# Effectiveness of brief interventions as part of the Screening, Brief Intervention and Referral to Treatment (SBIRT) model for reducing the nonmedical use of psychoactive substances: a systematic review

**DOI:** 10.1186/2046-4053-3-50

**Published:** 2014-05-24

**Authors:** Matthew M Young, Adrienne Stevens, James Galipeau, Tyler Pirie, Chantelle Garritty, Kavita Singh, Fatemeh Yazdi, Mohammed Golfam, Misty Pratt, Lucy Turner, Amy Porath-Waller, Cheryl Arratoon, Nancy Haley, Karen Leslie, Rhoda Reardon, Beth Sproule, Jeremy Grimshaw, David Moher

**Affiliations:** 1 Canadian Centre on Substance Abuse (CCSA), 75 Albert Street, Ottawa, Ontario K1P 5E7, Canada; 2 Department of Psychology, Carleton University, 1125 Colonel By Drive, Ottawa, ON K1S 5B6, Canada; 3 Ottawa Hospital Research Institute (OHRI), Ottawa Hospital - General Campus, 501 Smyth Road, Ottawa, ON K1H 8 L6, Canada; 4 Département de Pédiatrie, Hôpital Sainte-Justine, 3175 Ch DE LA Cote-Sainte-Cath, Montréal, QC H3T 1C5, Canada; 5 Direction de Santé Publique de Montréal, Faculté de Médecine, Université de Montréal, Montréal, QC H3C 3 J7, Canada; 6 Division of Adolescent Medicine, Department of Pediatrics, University of Toronto, 555 University Avenue, Toronto, ON M5G 1X8, Canada; 7 College of Physicians and Surgeons of Ontario, 80 College Street, Toronto, ON M5G 2E2, Canada

**Keywords:** brief intervention, drug use, psychoactive substance, SBIRT, screening, substance use, systematic review

## Abstract

**Background:**

The purpose of this systematic review is to assess the effectiveness of brief interventions (BIs) as part of the Screening, Brief Intervention, and Referral to Treatment (SBIRT) model for reducing the nonmedical use of psychoactive substances.

**Methods:**

Bibliographic databases (including MEDLINE, Embase, The Cochrane Library, CINAHL, and PsycINFO to April 2012) and gray literature sources were searched. We included randomized controlled trials that opportunistically screened adolescents or adults and then provided a one-to-one, verbal BI to those at risk of substance-use harm. Of interest was the nonmedical use of psychoactive substances (for example, drugs prohibited by international law), excluding alcohol, nicotine, and caffeine. Interventions comprised four or fewer sessions and were compared with no/delayed intervention or provision of information only. Studies were assessed for bias using the Cochrane risk of bias tool. Results were synthesized narratively. Evidence was interpreted according to the GRADE framework.

**Results:**

We identified 8,836 records. Of these, five studies met our inclusion criteria. Two studies compared BI with no BI, and three studies compared BI with information only. Studies varied in characteristics such as substances targeted, screening procedures, and BI administered. Outcomes were mostly reported by a single study, leading to limited or uncertain confidence in effect estimates.

**Conclusions:**

Insufficient evidence exists as to whether BIs, as part of SBIRT, are effective or ineffective for reducing the use of, or harms associated with nonmedical use of, psychoactive substances when these interventions are administered to nontreatment-seeking, screen-detected populations. Updating this review with emerging evidence will be important.

**Trial registration:**

CRD42012002414

## Background

Screening, Brief Intervention, and Referral to Treatment (SBIRT) is a comprehensive, integrated public health approach to the delivery of early intervention and treatment services for individuals experiencing substance use-related harms, as well as those who are at risk of experiencing such harms [1], who are not seeking, or unlikely to seek treatment. The SBIRT model is based on public health principles and procedures, and is designed to reduce the burden of injury, disease, and disability associated with the nonmedical use of psychoactive substances.

The protocol typically begins with a screening procedure that involves asking questions to evaluate whether the individual has experienced, or is at-risk of experiencing, substance use-related harms. Brief interventions (BIs) are typically delivered to those individuals at low to moderate risk of harms. Individuals identified as experiencing significant harm and/or having more serious signs of substance dependence warranting formal diagnosis may be referred to treatment services that are outside the scope of BIs.

Our interest in evaluating BIs was with respect to nonmedical use of psychoactive substances excluding alcohol, caffeine, or nicotine. For the purpose of this review, nonmedical psychoactive substances are drugs prohibited by international law [2], which include, but are not limited to: amphetamine-type stimulants, cannabis, cocaine, heroin, and MDMA (3,4-methylenedioxymethamphetamine); the nonmedical use of pharmaceuticals such as benzodiazepines, opioids, or dextromethorphan; and the use of substances such as solvents or inhalants (for example, gasoline, acetone, *etcetera*) when they are used for their intoxicating effects.

### Screening

SBIRT is intended to be implemented in healthcare settings or other community service settings frequented by the general population. In order to determine the likelihood that an individual is experiencing, or is at-risk of experiencing substance use-related harms, screening needs to be universal and opportunistic. By this, we mean that individuals are screened upon entering a program or organization (for example, hospital, primary care clinic, prison, or school program) as part of a standard intake procedure or process.

Screening may be conducted in a number of different ways. For example, intake staff may use psychometrically validated questionnaires or instruments that have been developed to accurately categorize users into low, moderate, or high risk categories. Psychometrically validated instruments have been developed for some types of substances, such as alcohol (Alcohol Use Disorders Identification Test, AUDIT [3]) or cannabis (the Cannabis Use Disorders Identification Test, CUDIT [4]). General drug screening instruments also exists (for example, Drug Abuse Screening Test, DAST [5]). However, screening instruments that reliably categorize users of other substances into low or moderate risk groups have not been developed (for example, heroin and cocaine). For those substances, screening may simply take the form of self-reported use or biological markers indicating use (for example, hair, urine, oral fluid, or blood). In the absence of validated instruments or biological markers, others may rely on even less rigorous screening methods such as the subjective judgment of the individual conducting the assessment. Regardless of the screening method employed, those deemed at risk of harms are typically provided a BI or referred to treatment.

### Brief interventions

In addition to the variability in screening procedures used, there is much variation in how BIs are defined and delivered. In general, BIs are in-person, time-limited efforts to provide information or advice, increase motivation to avoid substance use, or to teach behavior change skills with the aim of reducing substance use and the likelihood of experiencing negative consequences. This variation includes the number of conversations or meetings that take place during intervention delivery, as well as the amount of time spent conducting the BI. The systematic review conducted by Kaner *et al*. [6] defined ‘brief’ to mean four or fewer sessions and, in the context of BIs for alcohol in primary healthcare settings, are typically delivered within the normal consultation period of 5 to 30 minutes. In a review of interventions targeting alcohol, Bien *et al*. [7] suggested that successful BIs typically focus on the following elements, collectively referred to using the acronym FRAMES: Feedback on behavior and consequences, Responsibility to change, Advice, Menu of options to bring about change, Empathy, and Self-efficacy for change.

There is substantial scientific evidence of the benefits of the SBIRT model in primary health-care settings as a means of preventing and/or reducing the serious long-term harms associated with excessive alcohol use [8-10]. There is also accumulating evidence suggesting that BIs may be effective in reducing the nonmedical use of psychoactive substances, such as cannabis [11-15], ecstasy [16], cocaine [12,17,18], benzodiazepines [19], and opioids [3,17,20] among both youth and adults.

Although systematic reviews assessing the efficacy of BIs in reducing harms associated with risky alcohol have been conducted [6,21], there have been no published systematic reviews or meta-analyses that assess the effectiveness of BIs, among opportunistically screened populations, as part of the SBIRT model in reducing illicit drug use [22].

Our objective was to determine the effectiveness of BIs as part of the SBIRT model, compared with no BI or provision of information only, for reducing the nonmedical use of psychoactive substances among opportunistically screened populations identified as being at risk of harms and further, to determine whether any factors moderate the effect, using randomized evidence.

## Methods

We published our methods as a protocol before conducting the review [23] and registered the review with PROSPERO (Registration number CRD42012002414 [http://www.crd.york.ac.uk/PROSPERO/display_record.asp?ID=CRD42012002414]). This review is reported according to the PRISMA statement [24] and was conducted according to AMSTAR tool items for additional quality control [25] [see Additional file 1].

### Search strategy

We searched the following electronic databases: Ovid MEDLINE™ In-Process & Other Non-indexed Citations and Ovid MEDLINE™ (1946 to April 2012), Embase Classic + Embase (1947 to 6 April 2012), The Cochrane Library (searched 8 April 2012), Cumulative Index to Nursing and Allied Health Literature (CINAHL™) (searched 18 April 2012), PsycINFO™ (1806 to week 1 April 2012), Education Resources Information Center (ERIC) (searched 13 May 2012) and the CORK Database (searched 28 May 2012). All electronic search strategies were peer reviewed using the PRESS tool prior to implementation [26]; search strategies are presented in Additional file 2. We did not restrict the searches based on language, year of publication, or publication status.

For gray literature sources, numerous websites of relevant organizations, including those listed in ‘Grey Matters: a practical tool for evidence-based searching’ [27], were searched between 16 May and 22 May 2012 and are listed in Additional file 2. We scanned bibliographies of included articles and relevant systematic reviews. We searched clinicaltrials.gov and the WHO International Clinical Trials Registry Platform for ongoing studies.

### Selection criteria and process

We selected studies according to the following criteria:

Inclusion criteria:

• Study written in English or French.

• Randomized controlled trials (RCTs) or cluster RCTs.

• BIs administered to adolescents (12 to 18 years of age or equivalent by level of schooling), young adults (19 to 24 years of age), or adults (25 years and older) screened at risk of harms related to psychoactive substance use.

• Participants were identified by opportunistic screening regardless of setting (that is, the participants in the study were from a screen-detected population and not a population seeking treatment for substance abuse). We included studies with any screening procedure.

• Intervention was four sessions or less, included at least one of the FRAMES elements, and was delivered as a one-to-one verbal intervention to the individual.

• Intervention was compared with no/delayed intervention or provision of information only.

Exclusion criteria:

• Studies assessing alcohol, nicotine, or caffeine only.

• Group interventions or text-only online interventions.

• Studies addressing the effectiveness of the referral to treatment component of the SBIRT model only.

We uploaded the literature search results to systematic review software (Distiller SR^©^) for the study selection process. Our search was limited to systematic reviews published after 2009 because a recently conducted scoping review [22] did not locate earlier reviews on this topic. For all levels of study selection, we developed and pilot tested screening questions [see Additional file 3]. All titles and abstracts of records at Level 1 were screened once; those deemed not relevant were verified by a second person. Full-text reports of potentially relevant studies were assessed at Levels 2 and 3 by two independent reviewers; more than one level was used due to the complexity of applying the selection criteria to this literature. Disagreements were resolved by consensus or by a third reviewer. One reviewer tracked author responses regarding eligibility and identified multiple (companion) reports of the same study at Level 4.

### Data extraction and process

We extracted study and publication details, study design characteristics, inclusion and exclusion criteria, participant characteristics, details regarding the screening methods and personnel, details regarding the intervention and comparison groups and personnel, outcomes, and other additional information from included studies [see Additional file 4].

We identified the following as outcomes of interest:

#### Primary outcomes

• Substance use

• Frequency of use

• Quantity of use

• Use-related harms or negative consequences of use

• Changes in behavior likely to result in the reduction of negative substance use-related consequences (positive behavior change)

• Decision to attend treatment

#### Secondary outcom*e*s

• Use of different substances (including alcohol, caffeine, nicotine) from that for which the client received the intervention

• Intention to reduce substance use

• Other health measures

#### Adverse outcomes

• Any other reported adverse outcomes

These outcomes were extracted regardless of study follow-up time. We performed data calculations where needed (for example, change from baseline). For change-from-baseline calculations, we assumed a correlation coefficient of r = 0.25.

We developed and reviewed a data collection form in Distiller SR. One team member extracted information, and a second person verified all information.

### Risk of bias assessment

All RCTs were assessed using the Cochrane Risk of Bias tool (RoB tool) [28]. Other sources of potential bias that were assessed included fidelity (performance bias), recruitment bias for cluster trials [29], single versus multicenter studies [30], and study sponsorship bias. Some bias items were assessed at the study level (for example, randomization), while others were assessed at the outcome level (for example, selective reporting). We contacted corresponding authors of included studies regarding their consent procedures to inform the assessment of participant blinding. We assessed each study for the risk of bias for a given outcome and then determined a summary assessment across all studies for that outcome. Summary assessments were categorized into low, medium, and high risk of bias and incorporated into grading the quality of evidence.

One team member extracted risk of bias information and a second person verified all information.

### Evidence synthesis

Study characteristics were summarized narratively in the text and presented in tables. In order to assess whether meta-analyses of the data were possible, we assessed the quantity and methodological and clinical homogeneity of studies. We conducted narrative syntheses as meta-analysis was either not appropriate or possible. Where possible, we calculated and presented dichotomous outcomes as risk ratios (RR) and continuous outcomes as mean differences (MD), both with 95% confidence intervals. We contacted corresponding authors regarding inadequately reported data. Other analyses, such as subgroup analyses and funnel plot assessment, were pre-planned but not carried out due to few included studies.

The quality of evidence for all outcomes was evaluated using the GRADE methodology [31]. The quality of evidence was assessed across the domains of risk of bias, consistency, directness, precision, and publication bias. Each outcome was given a final adjudication of high, moderate, low, or very low.

### Protocol modifications

The pre-planned outcome ‘any standard/accepted biological markers of substance use’ represents a method for measuring use and is captured within other listed outcomes. Similarly, the pre-planned adverse outcome ‘self- or other reported use or increased use of different substances’ is captured as a secondary outcome. We included ‘composite’ outcomes where reported in studies as they captured relevant measures (for example, the Alcohol, Smoking and Substance Involvement Screening Test (ASSIST)). For feasibility we used a verification process for data extraction and risk of bias assessment rather than dual extraction. When grading the evidence, we felt ‘medium’ risk was more representative than ‘unclear’ risk for interpreting the risk of bias assessment across domains and studies for an outcome [32].

## Results

We located 9,631 bibliographic and 17 gray literature records. A flow diagram of the study selection process is shown in Additional file 5. Of the 8,836 records that remained after removing duplicates, 7,940 were excluded during title and abstract screening. Of the 896 full-text reports reviewed, 886 reports were excluded during two rounds of full text screening. Over 50% (n = 454) of the studies at this stage were excluded because the population was not opportunistically screened. A further 35% (n = 307) of the studies were excluded because they did not meet our study design criteria (that is, RCT, or cluster RCT). Remaining studies were excluded for a variety of reasons that occurred with a frequency of 5% or less, including full-text report was unavailable, intervention did not meet the definition of BI, *etcetera*. Of the ten remaining potentially eligible reports, eight reports detailing the results of five unique RCTs were included. Sixteen ongoing or completed trials were located [see Additional file 6]. A list of excluded studies is reported in Additional file 7.

### Other study designs

Given only five RCTs were located, we went back to determine if any excluded reports that employed other experimental designs (for example, non-RCTs, controlled before-after (CBA) or interrupted time series (ITS)) were relevant. Our search revealed 38 studies, but none of those studies met remaining selection criteria. These searches were mainly targeting RCT and non-RCT designs, for feasibility of resources; however, given the few studies meeting criteria of the 8,836 records screened, we felt it unlikely that additional studies would have been missed by our search.

### General characteristics of included trials

Of the five included studies, three were single-site RCTs [17,33,34], one was a multisite RCT [12,35,36], and one was a multisite, cluster RCT [37,38]. The single-site studies were conducted in the United States [17,34] and the cluster trial in Germany [37,38]. The other multisite study was conducted in Australia, Brazil, India, and the United States [12,35,36]. All studies were published after 2005 (Table 1). For the remainder of the review only the main report for each included study will be cited.

**Table 1:** Study and participant descriptive characteristics of included studies

**Author, year; design; funding**	**Summary of included participants; incentives**	**Summary of excluded participants**	**Demographics: mean age (range) of randomized participants; Sex (% female, intervention versus control)**	**Country**	**Setting**	**Number of participants screened**	**Randomized participants**	
							**I**	**C**
**BI versus no BI**								
Baer *et al*., 2007 [33]; Single site RCT; Government funding	127 participants who were homeless and with one or more binge drinking episodes or used illicit drugs four or more times in the past 30 days; Incentives offered for enrolling and attending BI and follow-up sessions	Those receiving alcohol or drug treatment in the past 30 days.	17.9 y (13 to 19 y); 44%^a^	United States	Nonprofit, faith-based drop-in center	254	75	52
Humeniuk *et al*., 2008 [35]; Multisite RCT; WHO and in-kind contributions and government grants from individual sites	731 (Australia n = 171, Brazil n = 165, India n = 177, USA n = 218) participants aged 16 to 62 y with a fixed address who scored in the moderate risk range for cannabis, cocaine, amphetamine stimulants, or opioids; Incentives offered for attending BI session and follow-up sessions	Those pending incarceration, with severe behavior, with past-month drug or alcohol treatment, or unable to attend the follow-up appointment.	31.4 y (16 to 62 y); 27.9%^a^	Australia, United States, Brazil, and India	One primary, urban general health outpatient hospital setting (Brazil); 31 primary, urban, general health-care units/clinics (Brazil, USA); one walk-in clinic associated with a drug treatment program (USA); several general medicine and dental urban clinics (USA); three clinics/centers specializing in sexually transmitted diseases (Australia, Brazil).	NR	372	359
**BI versus Written Information**								
Bernstein *et al*., 2009 [34]; Single site RCT; Government funding	210 participants who reported ‘3 to 5 days per month of cannabis use were included; Incentives offered for enrolling and attending follow-up sessions	Those institutionalized, in custody, in residential treatment, receiving a rape exam or were evaluated for suicide precautions.	Mean NR (14 to 21 y); 63.2% versus 67.6%	United States	Pediatric emergency department in an urban academic hospital.	7,804	68	71
Zahradnik *et al*., 2008 [37]; Cluster RCT (randomization by hospital ward); Government funding	126 participants (2 hospitals; 17 randomized wards) who consumed opioids, anxiolytics, hypnotics and sedatives, or caffeine with addiction potential for more than 60 days in the last 3 months or met criteria for DSM-IV dependence or abuse; Incentives offered for enrolling and attending BI and follow-up sessions	Those using opioids for cancer, with a terminal disease, with dependence on or use of illegal drugs, or receiving substance use treatment.	55.13 y (18 to 69 y); 64.9% versus 60%	Germany	Two hospitals (general and university); internal, surgical, and gynecological wards	6,042	NR	NR
Bernstein *et al*., 2005 [17]; Single-site RCT; Government funding	1,175 participants who self-reported use of cocaine and/or heroin in the last 30 days, and scored ≥3 on the DAST instrument; Incentives offered for enrolling and attending follow-up sessions	Those in drug use treatment or protective custody.	Mean NR (>18 y); 30.6% versus 28.2%	United States	Three walk-in clinics (urgent care, women’s clinic, homeless clinic) at an urban teaching hospital	23,669	590	585

^a^Percent female in total randomized population; information by group not reported.

BI, brief intervention; C, comparison group; DAST, drug abuse screening test; I*,* intervention group; NR, not reported; RCT, randomized controlled trial; *y*, year.

#### Participants and setting

One of the included studies assessed the effectiveness of BIs in homeless youth (13 to 18 years old) [33], one in youth and young adults (14 to 21 years old) [34], one in young adults and adults (16 to 62) [35], and two in adults only [17,37]. Four studies took place in a healthcare setting (primary care or hospital) [17,34,35,37] while one took place at a drop-in center [33].

#### Screening

There was considerable diversity in the screening instruments employed in the included studies (Table 2). Only one study [35] used a screening instrument whose psychometric properties have been well established. The other studies either used screening instruments of unknown/questionable validity or adapted versions of instruments whose psychometric properties had been published. Zahradnik *et al*. [37] used a combination of two screening instruments followed with a diagnostic interview to determine who would receive the intervention [37], while Bernstein *et al*. [17] employed a group of unspecified ‘substance abuse screening questions’ in conjunction with an instrument whose validity and reliability had been established for use in a clinical or research setting only. The two remaining studies used investigator developed, self-report screening instruments whose psychometric properties are unknown [33,34].

**Table 2:** Description of screening procedure and instruments of included trials

**Author, year**	**Screening procedure**	**Screening instrument(s)**	**Screening criteria**	**Validation of screening instrument**
**BI versus No BI**				
Baer *et al*. 2007 [33]	NR	Investigator developed instrument that included questions about binge drinking and past month use of illicit drugs	One or more binge drinking episodes or used illicit ‘street’ drugs four or more times in the past 30 days.	Not validated
Humeniuk *et al*. 2008 [35]	Questionnaires were either self-administered (Australia, USA) or by trained personnel (Brazil, India) with other demographic questions at primary care clinics in the various sites.	The Alcohol, Smoking and Substance Involvement Screening Test (ASSIST V3.0 [39])	ASSIST score in the moderate risk range (4 to 26) for cannabis, cocaine, amphetamine stimulants, or opioids. Those with scores in the low and high risk (except tobacco) ranges and those who frequently injected drugs were excluded.	The validity of the ASSIST has been assessed in primary healthcare settings and demonstrated good concurrent, construct, predictive, and discriminative validity [40]
			Among participants who scored moderate-risk for more than one substance, the focus of the intervention was the highest scoring or the substance of most concern to the participant.	
**BI versus Written Information**				
Bernstein *et al*. 2009 [34]	NR	Investigator developed instrument referred to by authors as the ‘Youth and Young Adult Health and Safety Needs Survey’. Included unspecified risk items from the USA Centers for Disease Control, Youth Behavioral Risk Factor Surveillance Survey (YBRFS; [41]).	Did not report ‘at risk alcohol use’; smoked cannabis ≥3 times in the last 30 days; reported risky behavior associated with cannabis use; reported ‘3 to 5 days per month’ of cannabis use.	Not validated
Zahradnik *et al*. 2008 [37]	Participants were asked to complete a self-report screening questionnaire. Those meeting screening criteria were given a diagnostic interview.	*Self-report questionnaire.* Assessed prescription drug intake by asking ‘have you been taking prescription drugs like hypnotics, sedatives, or analgesics regularly within the last four weeks?’ and screened for disorders using:	Participants were included if they:	SDS - scale’s psychometric properties published in Gossop *et al*. [42] However, psychometric properties of translated/adapted version unknown.
			1) met criteria for prescription drug dependence or abuse (3+ points on the adapted SDS and 5+ points on the QPM and were deemed depended via diagnostic interview) or,	
		1) German translation of the Severity of Dependence Scale (SDS [42,43]) adapted to assess prescription drug dependence.	2) consumed prescription drugs with addiction potential for at least 60 days in the last 3 months.	
				QPM - According to authors the QPM was validated; however, this was impossible to verify as results are published in a German study [44].
		2) A questionnaire for prescription drug misuse (QPM; [44])		
		*Diagnostic interview*. Section E of SCID-I (Structured Clinical Interview for DSM-IV for Axis I Disorders [45]).		
Bernstein *et al*. 2005 [17]	NR	‘Standard substance abuse screening questions for quantity and frequency in the last month’ that were integrated as part of a health needs history. Exact questions not reported.	Current use of drugs (as determined by the screening questions) and ≥3 on the 10-item DAST	‘Standard substance abuse screening questions’ - not validated
		Those screening positive were administered the 10-item Drug Abuse Severity Test (DAST-10 [46])		DAST-10 has satisfactory levels of validity and reliability for use in a clinical or research setting [47,48]

NR, not reported; USA, United States.

Humeniuk *et al*. [35] was the only study to provide BIs to those participants screened at a moderate risk level only and then refer to treatment those screened at high risk (as determined by the ASSIST). All other included studies assigned participants to control or intervention groups if they scored more than a specific threshold but did not specify any upper level threshold.

#### Brief intervention

There was also a considerable degree of heterogeneity in the characteristics of the BIs (see Table 3 for details regarding the BIs and how they were administered). Bernstein *et al*. [34] screened for and administered an intervention targeting a single, specific substance (cannabis). Bernstein *et al*. [17], Baer *et al*. [33], and Zahradnik *et al*. [37] screened for and administered interventions targeting a set of or a group of drugs: cocaine and/or heroin [17]; alcohol, cannabis, and other drugs [33]; or prescription drugs [37], respectively. Humeniuk *et al*. [35] screened for multiple drugs then targeted the BI at the substance that screening indicated was most problematic or the substance of most concern to the participant [35].

**Table 3:** Characteristics of brief interventions (BIs) and control groups

**Author and year**	**Target substance**	**Intervention**	**Individual delivering BI (training provided)**	**Intervention content**	**Treatment approach**	**Measure of intervention fidelity**	**Control group**
**BI versus No BI**							
Baer et al. 2007 [33]	Alcohol, cannabis, and other drugs	MI session (in-person, average 17 min)	Master’s level clinician or project director (all trained in MI techniques).	The interventions included feedback on behavior and consequences, self-efficacy for change, and advice (with permission). Youth provided feedback on the menu of options for discussion, and counselors addressed up to 6 topics in total across sessions. Visuals were also used to demonstrate risk relationships and normative comparisons.	Authors cite Miller et al. [49] regarding MI and the substance use check-up model.	Regular review of session audio tapes by supervisor. Extent of adherence NR.	*no BI*
							Showers and laundry facilities, meals, prayer, open social time, and brief counseling and case management if the youth desired it^a^.
		+					
		2nd MI session (in-person, average 32 min.)					
		+					
		3rd MI session (in-person, average 32 min.)					
		+					
		4th MI session (in-person, average 32 min.)					
		All four sessions scheduled within 4 weeks from first session.					
Humeniuk *et al*. 2008 [35]	Cannabis, cocaine, amphetamine-type stimulants, or opioids depending on ASSIST score and concern of participant	ASSIST-linked BI (in-person, 5 to 15 min.) and written information	Healthcare clinic staff (US, Australia, India); Clinicians and Researchers (Brazil); training was provided to all those conducting interventions.	Intervention session incorporated MI techniques and was adapted culturally within each country. The session included feedback on behavior and consequences and advice and used the ASSIST Feedback Report Card during the discussion. Participants left session with a copy of the Report Card, specific drug information booklets, and a take-home guide (Self-help Strategies for Cutting Down or Stopping Substance Use)	BI designed to move participants through Prochaska and DiClemente’s stages of change provided by [50]. Interventions incorporates FRAMES [7] elements as well as MI techniques [51].	Checklist of intervention details was used to maintain consistency across sites. Extent of adherence NR.	*no BI + Delayed intervention*
							Could contact the clinical interviewer if concerns regarding the study or their substance use. Intervention received after completing the ASSIST questionnaire at follow-up (3 months).
**BI versus Written Information**							
Bernstein *et al*. 2009 [34]	Cannabis	Structured conversation (in-person, 20 to 30 min.) and written information	Peer educators (<25y). Most completed undergraduate education (received one month of training).	Initial conversation included feedback on behavior and consequences, menu of options to bring about change, self-efficacy for change, and developing a behavior change plan. Questions from the CRAFFT [52] and a *Readiness to Change* ruler were used as part of initial conversation. Booster call included reviewing the change plan, inquiring as to progress, and offered referrals.	Intervention adapted from a previous study on adult cocaine and heroin use by the same author [17]. Content based MI techniques [51,53] and previous research [54-56].	Adherence to intervention was assessed weekly by investigators and the project coordinator. Taped recordings were scored against an adherence checklist of key intervention elements. All initial sessions met the required 80/100 points on the adherence checklist.	*no BI + Written Information* (risks of cannabis use, available community resources, and list of adolescent treatment facilities).
		+					
		telephone call (5 to 10 min.) 10 days later.					
Zahradnik *et al*. 2008 [37]	Prescription drugs (opioids, anxiolytics, hypnotics and sedatives, and caffeine)	MI session (in-person, 30 to 40 min)	Four psychologists, expertise in clinical treatment and research (two weeks of training in MI)	Verbal interventions were MI. Specific content not described.	MI as described by Hettema *et al*., [57] as well as the Transtheoretical Model of Behavior Change [50]	With participant consent, sessions were audio taped and coded for consistency by other researchers. Extent of adherence NR.	*no BI + Written Information* (booklet about prescription drugs).
		+		Feedback letter included strategies for improving self-efficacy and maintaining changes, where appropriate.			
		2nd MI (by telephone, 20 to 30 min) 4 weeks later					
		+					
				Throughout the intervention, psychologists communicated the necessity a medical professional supervision when discontinuing or reducing use of prescription medication.			
		feedback letter 8 weeks after first session					
Bernstein *et al*. 2005 [17]	Cocaine and/or heroin	MI session (in-person, average 20 min) and written information	Peer, experienced substance use outreach worker also in recovery (authors state training was intensive, systematic, and manual-driven).	*Initial session.* A semi-scripted motivational interview tailored to individual behavior, risks, culture and language. Intervention included self-efficacy for change and an action plan for behavior change. Participants received referrals, if desired, and written information (treatment options and harm reduction information about safe sex and needle exchange).	None provided. Intervention first developed for Project ASSERT in the emergency department [58] to help patients to recognize and change behaviors posing health risks.	Adherence determined through role plays with simulated patients, supervised patient interviews, and completion of a form per patient addressing 12 required elements. Extent of adherence NR.	*no BI + Written Information*
							Interventionist indicated to participants ‘based on your screening responses, you would benefit from help with your drug use’. Written information regarding treatment options (for example, detox, AA/NA, acupuncture, and residential treatment facilities) and harm reduction information about safe sex and needle exchange were provided.
		+					
		telephone call (5 to 10 min.) 10 days later					
				*Telephone call*. Reviewed the action plan and addressed alternative referrals, if needed.			

^a^Information provided by authors.

AA, alcoholics anonymous; ASSIST, alcohol, smoking, and substance involvement screening test; BI, brief intervention; FRAMES, feedback on behavior and consequences, Responsibility to change, Advice, Menu of options to bring about change, Empathy, Self-efficacy for change; MI, motivational interview; min, minutes; NA, narcotics anonymous; NR, not reported; y, years.

In addition to targeting different substances, interventions comprised diversity in the number and length of sessions. Humeniuk *et al*. [35] simply assessed the effect of a single verbal intervention and accompanying written information [35]. The BIs in Bernstein *et al*. [17] and Bernstein *et al*. [34] consisted of an initial verbal intervention, take-home written information, and a follow-up telephone call. Among the last two trials, Baer *et al*. [33] consisted of four sessions, and Zahradnik *et al*. [37] consisted of two sessions with a feedback letter mailed eight weeks later. Despite these variations, there was consistency in the treatment approach that informed the BIs in that all BIs either explicitly or implicitly incorporated a motivational interviewing approach.

All trials reported using techniques/strategies to ensure adherence to the planned BI. Though it was implied by all the studies that these strategies ensured that all interventions were administered as planned, only one of the included studies reported this explicitly [34].

#### Comparison groups

All three comparison groups of interest were encountered among included studies (Table 3). Three studies provided participants with written information about the risks of drug use [34,37] or list of local treatment options [17]. For the remaining two studies (no BI) Baer *et al*. [33] provided the care or service sought by the individual, and Humeniuk *et al*. [35] provided the care or service plus delayed intervention.

### Risk of bias assessments

Additional file 8 outlines the risk of bias assessments by domain for included studies. Supports for judgments are provided in Additional file 9. Overall, studies were deemed at medium or high risk of bias for outcomes.

Most studies reported an adequately randomized method, while two studies reported using a concealed method to implement randomization [17,35]. We assessed ‘blinding of participants and personnel’ across all outcomes as any systematic changes would have affected all outcomes. In all studies, it was not possible to blind personnel to what they were delivering to participants. In two studies, participants were aware of the study intent and what groups they could be allocated to; this information was unclear or likely to have occurred in remaining studies. Outcomes collected through objective means (biochemical analysis or use of database records) are at low-risk for assessor bias. Loss-to-follow-up (total amount or differential amount between groups), handling of missing data, and unknown/unreported reasons were important issues regarding attrition, and almost all studies were at unclear or high risk of bias. Studies were also at unclear or high risk for selective reporting bias. One study was at low risk of performance bias regarding fidelity of the intervention (other bias), while remaining studies were at unclear or high risk. The one cluster trial was at unclear risk of recruitment bias. Study sponsorship bias was not an issue in these studies.

### Effects of brief interventions

The effects of BIs in the included studies are described and analyzed based on comparison group: BI versus no intervention and BI versus written information only. Included in the former group is the Humeniuk *et al*. [35] study in which the delayed intervention was administered after outcome data were collected at follow-up. Results of included studies are presented in Tables 4 and 5. We were unable to fore-analyze unit of analysis errors in studies due to insufficient information, so we report the point estimate without confidence intervals.

**Table 4:** Evidence table for brief intervention (BI) versus no BI in participants screened for at-risk substance use

**Outcome**^**a**^	**Follow up**	**Event rates BI versus no BI**	**Effect Estimate (95% CI)**	**Studies (people)**	**Quality of evidence**	**Comments**
PRIMARY OUTCOMES						
**Substance use**						
*Not reported in any studies*	n/a	n/a	Not estimable	0 (0)	n/a	
**Frequency of use**						
**Change in days of use - Cannabis**	1 mo	-3.7 versus -6.1 d (fewer d at 1 mo)	MD 2.40 (-3.80 to 8.61)^c^	1 (89)	Very Low	(+) value for MD means fewer days of use with control
Self-report, past 30 d						
Change in mean from baseline^b^[33]						
	3 mo	-2.6 versus -5.9 d (fewer d at 3 mo)	MD 3.30 (-2.84 to 9.44)^c^	1 (89)	Very Low	
**Change in days of use - Other drugs**^d^	1 mo	-2.3 versus -3 d (fewer d at 1 mo)	MD 0.70 (-2.95 to 4.35)^c^	1 (89)	Very Low	
Self-report, past 30 d.						
	3 mo	-2.8 versus -2.3 d (fewer d at 3 mo)	MD -0.50 (-4.30 to 3.30)^c^	1 (89)	Very Low	
Change in mean from baseline^b^[33]						
**Change in days abstinent**^**e**^	1 mo	3.7 versus 6.4 d (more d at 1 mo)	MD -2.70 (-8.21 to 2.81)^c^	1 (89)	Very Low	(-) value for MD means more days abstinent with control
Self-report, past 30 d.						
Change in mean from baseline^b^[33]						
	3 mo	2.7 versus 6 d (more d at 3 mo)	MD -3.30 (-8.73 to 2.13)^c^	1 (89)	Very Low	
**Quantity of use**						
*Not reported in any studies*	n/a	n/a	Not estimable	0 (0)	n/a	
**User-related harms or negative consequences of use**						
*Not reported in any studies*	n/a	n/a	Not estimable	0 (0)	n/a	
**Positive behavior change**						
*Not reported in any studies*	n/a	n/a	Not estimable	0 (0)	n/a	
**Decision to attend treatment**						
*Not reported in any studies*	n/a	n/a	Not estimable	0 (0)	n/a	
**Composite outcome**						
**ASSIST Tool Score**^**f **^**- All substances**	3 mo	-7.8 versus -4.6 (fewer points at 3 mo)	MD -3.20, 95% CI (-6.77 to 0.37)	1 (628)	Low	Higher score = higher substance involvement.
Sum score, range 0 to 27+ points.						
						(-) value for MD means greater reduction in change score with BI
Change in means from baseline^b^[35]						
**SECONDARY OUTCOMES**						
**Use of different substances**						
*Not reported in any studies*	n/a	n/a	Not estimable	0 (0)	n/a	
**Intention to reduce use**						
*Not reported in any studies*	n/a	n/a	Not estimable	0 (0)	n/a	
**Other health measures**						
**Use of drop-in centre services**	1 mo	0.9 versus -0.2 d (more versus fewer d)	MD 1.10 (-1.88 to 4.08)^cg^	1 (89)	Very Low	(+) value for MD means greater use with BI
Objective, past 30 d.						
Change in means from baseline^b^[33]	3 mo	−1.1 versus -1 d (fewer d at 3 mo)	MD -0.10 (-3.23 to 3.03)^cg^	1 (89)	Very Low	
**Use of drop-in additional services**	1 mo	0 versus 0.1 d (more d at 1 mo)	MD -0.10 (-0.72 to 0.52)^cg^	1 (89)	Very Low	
Objective, past 30 d.						
Change in means from baseline^b^[33]	3 mo	0.5 versus -0.1 d (more versus fewer d)	MD 0.60 (-0.15 to 1.35)^cg^	1 (89)	Very Low	
**Use of other agency services**	1 mo	−2.4 versus -7 d (fewer d at 1 mo)	MD 4.60 (-5.05 to 14.25)^cg^	1 (89)	Very Low	
Self-report, past 30 d.						
Change in means from baseline^b^[33]	3 mo	−3.4 versus -8.2 d (fewer d at 3 mo)	MD 4.80 (-4.44 to 14.04)^cg^	1 (89)	Very Low	
**ADVERSE OUTCOMES**						
*Not reported in any studies*	n/a	n/a	Not estimable	0 (0)	n/a	

^a^For change from baseline data, means for baseline and follow-up timepoints are shown in Table S5, where possible.

^b^Change in mean analysis calculated as the reported mean at follow-up minus the mean at baseline.

^c^Calculated from authors’ data at baseline and follow-up, assuming a correlation coefficient of 0.25.

^d^Drugs other than tobacco, alcohol, cannabis were assessed.

^e^A few people with alcohol use were included in this analysis.

^f^Composite outcome: substance use, frequency of use, use-related harms or negatives consequences, intention to reduce substance use, another person concerned with use, use of drug by injection.

^g^Possible unit of analysis error.

ASSIST, alcohol, smoking and substance involvement screening test; CI, confidence interval; d, days; MD, mean difference; mo, months; RR, risk ratio.

**Table 5:** Evidence table for brief intervention (BI) versus written information in participants screened for at-risk substance use

**Outcome**^**a**^	**Follow up**	**Event rates BI versus info**	**Effect estimate (95% CI)**	**Studies (people)**	**Quality of evidence**	**Comments**
**PRIMARY OUTCOMES**						
**Substance use**						
**Abstinence - All substances**	3 mo	Range 14 to 18% versus 9 to 13%	RR 1.12 (0.41 to 3.09)	2 (223)	Very low	Two studies not statistically significant.
			RR 2.08^c^			
Cannabis (Self-report^b^, past 30 d, [34]).			See Comments			
Sedatives/hypnotics/opioids^d^ (NR, period not provided, [37])						
**Abstinence - Cocaine/heroin**	6 mo	17% versus 13%^f^	Adj RR 1.41 (0.98 to 1.95)^gh^	1 (778)	Low	
Objective^e^, past 30d [17]						
**Abstinence - All substances**	12 mo	Range 25 to 45% versus 20 to 22%	RR 2.05 (1.13 to 3.70)	2 (228)	Very low	Mixed results between studies.
			Adj RR 1.30^cgi^			
Cannabis (Self-report^b^, past 30d, [34])						
			See Comments			
Sedatives/hypnotics/opioids^d^ (NR, assessment period NR, [37])						
**High on cannabis**	3 mo	36/42 (86%) versus 46/55 (84%)^f^	Adj RR 1.05 (0.82 to 1.15)^gj^	1 (102)	Low	
Self-report^b^, past 30d [34]						
	12 mo	25/47 (53%) versus 41/55 (75%)^f^	Adj RR 0.72 (0.45 to 0.97)^gj^	1 (102)	Very low	
**Reducing use >25% - Sedatives/hypnotics/opioids**^**d**^	3 mo	29/56 (52%) versus 21/70 (30%)	RR 1.73^c^	1 (126)	Very low	Results favor BI over control.
NR, period of assessment not provided [37]	12 mo	28/56 (50%) versus 34/70 (49%)^f^	Adj RR 0.96^cgi^	1 (126)	Very low	Results NS
**Frequency of use**						
**Change in cannabis consumption.** Mean change from baseline^k^, self-report^b^, past 30 d [34]	3 mo	-5 versus -0.8 d (fewer d at 3 mo)	MD -4.2 (-8.1 to -0.3)	1 (95)	Low	(−) value for MD indicates fewer d consumption with BI
	12 mo	-7.1 versus -1.8 d (fewer d at 12 mo)	MD -5.3 (-0.6 to 10)	1 (102)	Low	
**Quantity of use**						
**Defined daily dosage - Sedatives/hypnotics/opioids**^**d**^	3 mo	0.42 versus 0.12 (dosage higher at 3 mo)	MD 0.30^c^	1 (126)	Very low	Results NS
Mean change from baseline^k^, NR						
Patient’s dose of a given prescription drug per day (in mg) divided by the product-specific WHO measure [37]	12 mo	Not provided	See Comment	1 (126)	Very low	Authors state no significant difference between groups, *P* = 0.330
**Change in drug level - Cocaine**	6 mo	-180 versus -21 ng/ 10 mg (less at 6 mo)	See Comment	1 (376)	Low	Authors state adjusted *P* = 0.058, likely representing multiple adjusted analyses^m^
Change in mean from baseline^l^, objective^e^[17]						
**Change in drug level - Opioids**	6 mo	-7.6 versus -7.8 ng/ 10 mg (less at 6 mo)	See Comment	1 (189)	Low	Authors state adjusted *P* = 0.186, likely representing multiple adjusted analyses^m^
Change in mean from baseline^l^, objective^e^[17]						
**Use-related harms or negative consequences of use**						
**Carried a weapon** (gun, knife, club)	3 mo	5/42 (12%) versus 17/55 (31%)^f^	Adj RR 0.44 (0.15 to 1.09)^gn^	1 (97)	Very Low	
Self-report, past 30 d [34]						
	12 mo	5/47 (11%) versus 11/55 (20%)^f^	Adj RR 0.62 (0.20 to 1.60)^gn^	1 (102)	Very Low	
**Drove a car after using cannabis.** Self-report, past 30 d [34]	3 mo	6/42 (14%) versus 10/55 (18%)^f^	Adj RR 0.85 (0.28 to 2.08)^gn^	1 (97)	Very Low	
	12 mo	8/47 (17%) versus 13/55 (24%)^f^	Adj RR 0.67 (0.26 to 1.48)^gn^	1 (102)	Very Low	
**Rode in a car with a person drunk/high after cannabis use.** Self-report, past 30 d [34]	3 mo	11/42 (26%) versus 13/55 (24%)^f^	Adj RR 1.01 (0.46 to 1.88)^gn^	1 (97)	Very Low	
	12 mo	10/47 (21%) versus 13/55 (24%)	Adj RR 0.85 (0.37 to 1.67)^gn^	1 (102)	Very Low	
**Physical fight.** Self-report, past 30 d [34]	3 mo	9/42 (21%) versus 14/55 (25%)^f^	Adj RR 0.91 (0.39 to 1.76)^gn^	1 (97)	Very Low	
	12 mo	6/47 (13%) versus 19/55 (35%)^f^	Adj RR 0.35 (0.12 to 0.87)^gn^	1 (102)	Low	
**Positive behavior change**						
**Tried to cut back on cannabis use.** Self-report, past 3 and 12 mo [34]	3 mo	29/42 (69%) versus 28/55 (51%)^f^	Adj RR 1.36 (0.96 to 1.64)^gn^	1 (97)	Low	
	12 mo	34/47 (72%) versus 33/55 (60%)^f^	Adj RR 0.96 (0.91 to 1.45)^gn^	1 (102)	Low	
**Tried to stop using cannabis.** Self-report, past 3 and 12 mo [34]	3 mo	23/42 (55%) versus 19/55 (35%)^f^	Adj RR 1.58 (1.01 to 2.12)^gn^	1 (97)	Low	
	12 mo	25/47 (53%) versus 21/55 (38%)^f^	Adj RR 1.42 (0.92 to 1.90)^gn^	1 (102)	Low	
**‘Tried to be careful about situations you got into when using marijuana’**	3 mo	32/42 (76%) versus 38/55 (69%)^f^	Adj RR 1.13 (0.84 to 1.30)^gn^	1 (97)	Low	
Self-report, past 3 and 12 mo [34]	12 mo	34/47 (72%) versus 38/55 (69%)^f^	Adj RR 1.05 (0.76 to 1.24)^gn^	1 (102)	Low	
**Decision to attend treatment**						
**Abstinence obtained by substance use treatment, including detox [**[17]**]**	6 mo	n/a	Not estimable	1 (118)	n/a	Data poorly reported, not provided by group.
**SECONDARY OUTCOMES**						
**Use of different substances**						
**Change in type of drug from baseline to follow-up - Cocaine/opioids**	6 mo	n/a	Not estimable	1 (118)	n/a	Poorly reported by authors.
Change from baseline [17]						
**Intention to reduce use**						
*Not reported in any studies*	n/a	n/a	Not estimable	0 (0)	n/a	
**Other health measures**						
**Felt unsafe**	3 mo	14/42 (33%) versus 25/55 (45%)^f^	Adj RR 0.67 (0.33 to 1.16)^gn^	1 (97)	Low	
Self-report, past 30 d [34]						
	12 mo	11/47 (23%) versus 29/55 (53%)^f^	Adj RR 0.35 (0.16 to 0.72)^gn^	1 (102)	Low	
**Change in ASI composite score from baseline - Cocaine and/or heroin**	3 mo	Not reported	Not estimable; See Comment	1 (854)	Low	Authors state not statistically significant
Change from baseline	Drug - 6 mo	49% versus 46% reduction from baseline	Not estimable; See Comment	1 (562)	Low	Authors state *P* = 0.06
Drug and medical subscales [17]						
	Med - 6 mo	56% versus 50% reduction from baseline	Not estimable; See Comment	1 (562)	Low	Authors state *P* = 0.055
**Other adverse outcomes**						
*Not reported in any studies*		n/a	Not estimable	0 (0)	n/a	

^a^For change from baseline data, means for baseline and follow-up timepoints are shown in Table S5, where possible.

^b^Self-report using Timeline Followback Calendar.

^c^Confidence interval not presented due to unit of analysis error.

^d^A small proportion (1.5%) of participants were assessed for caffeine use in this study.

^e^Objective measure by biochemical hair analysis.

^f^Unadjusted event rates.

^g^Adjusted RR calculated from authors’ adjusted OR.

^h^Adjusted for health insurance and homelessness.

^i^Adjusted for prescription drug dependence according to the Structured Clinical Interview for DSM-IV Axis I disorders.

^j^Unclear what variables were adjusted for.

^k^Mean change analysis first calculates the change between follow-up and baseline values for each participant and then computes the mean across those data.

^l^Change in mean analysis calculated the reported mean at follow-up minus the mean at baseline.

^m^Unclear but likely adjusts for gender, race, age, EuroQol scores, previous psychiatric history, randomization status, education level, drug route, drug problem severity (Drug Abuse Severity Test score at baseline, polydrug use, injection drug use, baseline Addiction Severity Index drug score, number of previous treatment episodes) and readiness to change.

^n^Unclear what variables adjusted for. adj, adjusted; ASI, addiction severity index; BI, brief intervention; CI, confidence interval; d, days; info, information; med, medical; MD, mean difference; mo, months; NS, not significant; RR, risk ratio.

#### Brief intervention versus no intervention

Two studies evaluated the BI with no or delayed intervention. Baer *et al*. [33] assessed a four-session BI targeting alcohol, cannabis, and other drug use among adolescents at a faith-based drop-in center. Humeniuk *et al*. [35] assessed a single-session intervention plus written materials targeting cannabis, cocaine, amphetamine-type stimulants, or opioids among those 16 years and older at healthcare settings across four countries.

Few outcomes were reported and by only one study each. Groups were not statistically significant for change in frequency of use measures [33], a composite score measure [35], and use of drop-in or other agency services from baseline to one and/or three months of follow-up [33] (Table 4). In Humeniuk *et al*. [35] general health outcomes were only assessed among participants in the intervention group and are therefore not included in Table 4. Remaining outcomes of interest were not assessed in either study.

#### Brief intervention versus written information only

Three studies evaluated the BI compared with the provision of written information only about the risks of drug use or local treatment services/options. Bernstein *et al*. [34] assessed one session plus written information and a telephone call targeting adolescents and young adults cannabis use at a pediatric emergency hospital department. Zahradnik *et al*. [37] assessed one session plus telephone call and a feedback letter targeting those 18 and older who regularly use prescription drugs with addiction potential or met DSM-IV criteria for dependence in a hospital setting. Finally, Bernstein *et al*. [17] assessed one session plus written information and a telephone call targeting those 18 years and older reporting heroin or cocaine use.

Most outcomes of interest were reported on but mainly by one study each. Some outcomes were assessed with multiple measures. Where authors reported two or more substances together, data for the individual substances are provided in Additional file 10. A few studies addressed abstinence as a measure of substance use for differing substances and with varied follow-up times: Two studies were not statistically significant at 3-months [34,37], one study not significant at 6 months [17], and two studies reported mixed results at 12-months [34,37] (Table 5). With few studies and important clinical and methodological heterogeneity between those studies, we did not meta-analyze. Remaining outcome measures (other substance use measures, frequency of use, quantity of use, use-related harms, positive behavior change, other health measures) were mostly not statistically significant [17,34,37] (Table 5, Additional file 10). A few measures (quantity of use, decision to attend treatment, and use of different substances) were poorly reported (Table 5). ‘Intention to reduce use’ was not reported in any study.

## Discussion

Few studies have assessed the effectiveness of BIs among opportunistically screened populations as part of the SBIRT model for reducing the nonmedical use of psychoactive substances. When comparing the intervention with no intervention or to written information only, most outcomes were not statistically significant. However, the overall quality of the evidence per outcome is limited or very uncertain [see Additional file 11]. Due to the few included studies, results are imprecise and largely could not be assessed for consistency. The literature has important methodologic limitations leading to medium or high risks of bias for outcomes. The body of evidence, therefore, is limited given the few included studies with mainly small sample sizes and the heterogeneity in study characteristics, including the measurement of outcomes.

### Practice implications

Insufficient evidence exists to make conclusions as to whether BIs are effective or ineffective at reducing the use of or harms associated with the nonmedical use of psychoactive substances other than alcohol, nicotine, or caffeine when these interventions are administered to nontreatment-seeking, screen-detected populations.

### Research implications

We are aware of 16 ongoing studies, some with large sample sizes, which are potentially relevant to this review. Since the current evidence base is inconclusive, updating this review when the results of the ongoing studies become available will be important.

Given the variation observed among characteristics of the included studies, future research in the area would benefit from modifications in scope and study design. Firstly, we propose a focus on primary care settings, to supplement the evidence base of four of our included studies, before evaluating other community service settings. Secondly, a standard, validated screening instrument with an acceptable sensitivity and specificity profile is an important first step in determining the effectiveness of the SBIRT model among nontreatment-seeking populations. The instrument should be designed to take a relatively short time to complete, as a pragmatic consideration for implementing the SBIRT model. Thirdly, interventions that are more clearly reported and with sufficient detail to be replicated in other trials would also help develop this body of evidence. Fidelity of the delivered intervention should be collected and reported on. Finally, agreement on a core set of defined outcomes, their measurements, and lengths of follow-up will be essential to ensuring relevance to practice and to allow meta-analysis of studies.

There are additional items that researchers could implement to increase internal validity. The consent process should be designed such that participants are unaware of the intent of the study or to the groups for which allocation is possible. Researchers should consider using a sham intervention as a control group that would address another aspect of wellbeing (for example, nutrition) to help blind participants to group allocation. In addition, if known, researchers should indicate reasons for client drop-out.

Incomplete reporting (for example, informed consent procedures and intervention details) was a general barrier in attempting to assess studies against inclusion criteria as well as to assess for risks of bias. Davidson *et al*. [59] provides detailed reporting guidance.

Future research incorporating these modifications will enable meaningful statements on SBIRT effectiveness.

Consult the protocol modifications section for possible limitations; our work has been conducted according to AMSTAR standards.

## Conclusions

Insufficient evidence exists as to whether BIs as part of SBIRT are effective or ineffective at reducing the use of or harms associated with the use of nonmedical use of psychoactive substances other than alcohol, nicotine, or caffeine when these interventions are administered to nontreatment-seeking, screen-detected populations. Given the evidence base is inconclusive, emerging evidence from existing ongoing studies may help to stabilize conclusions about the effectiveness of BI.

## Abbreviations

ASSIST: Alcohol, Smoking and Substance Involvement Screening Test; AUDIT: Alcohol Use Disorders Identification Test; BI: brief intervention; CBA: controlled before-after; CUDIT: Cannabis Use Disorders Identification Test; DAST: Drug Abuse Screening Test; FRAMES: Feedback on behavior and consequences, Responsibility to change, Advice, Menu of options to bring about change, Empathy, and Self-efficacy for change; ITS: interrupted time series; MD: mean difference; QPM: questionnaire for prescription drug misuse; RCT: randomized controlled trial; RoB: Cochrane Risk of Bias; RR: risk ratio; SBIRT: Screening, Brief Intervention, and Referral to Treatment model; SCID-I: Structured Clinical Interview for DSM-IV for Axis I Disorders; SDS: Severity of Dependence Scale; YBRFS: Youth Behavioral Risk Factor Surveillance Survey.

## Competing interests

The authors declare that they have no competing interests.

## Authors’ contributions

All authors made substantial contributions according the International Committee of Medical Journals Editors authorship criteria. MMY, AS, TP, CG, LT, APW, CA, NH, KL, RR, BS, JeG, and DM contributed to the concept and design. MMY, AS, JaG, TP, CG, KS, FY, MG, MP, LT, and APW contributed to the acquisition of data; MMY and AS drafted the manuscript while all other authors revised it critically for important content; all authors read and approved the final manuscript. Other contributors not meeting authorship requirements are acknowledged below.

## Supplementary Material

Additional file 1 Completed PRISMA checklist. Click here for file

Additional file 2 Database search strategies for Ovid MEDLINE™ In-Process and Other Non-indexed Citations and Ovid MEDLINE™ (1946 to April 2012), Embase Classic + Embase (1947 to 06 April 2012), The Cochrane Library (searched 08 April 2012), Cumulative Index to Nursing and Allied Health Literature (CINAHL™) (searched 18 April 2012), PsycINFO™ (1806 to week 1 April 2012), Education Resources Information Center (ERIC) (searched 13 May 2012) and the CORK Database (searched 28 May 2012), and gray literature sources. Click here for file

Additional file 3 Screening questions used by reviewers to decide if records met inclusion criteria. Click here for file

Additional file 4 Data extraction form. Click here for file

Additional file 5 PRISMA flow diagram of study selection. Click here for file

Additional file 6 Potentially relevant ongoing trials. Click here for file

Additional file 7 Excluded studies during full text screening. Click here for file

Additional file 8 Risk of bias assessments for included studies. Click here for file

Additional file 9 Supports for risk of bias assessments for included studies. Click here for file

Additional file 10 Additional data used for calculations or not reported in the main report. Click here for file

Additional file 11 Quality of evidence tables. Click here for file
