# Supplementary material for: Effectiveness of brief interventions as part of the Screening, Brief Intervention and Referral to Treatment (SBIRT) model for reducing the nonmedical use of psychoactive substances: a systematic review
Source: Syst Rev. 2014 May 24;3:50. doi: 10.1186/2046-4053-3-50 (PMC4042132; doi:10.1186/2046-4053-3-50)
Supplement: Additional file 2 — Database search strategies for Ovid MEDLINE™ In-Process and Other Non-indexed Citations and Ovid MEDLINE™ (1946 to April 2012), Embase Classic + Embase (1947 to 06 April 2012), The Cochrane Library (searched 08 April 2012), Cumulative Index to Nursing and Allied Health Literature (CINAHL™) (searched 18 April 2012), PsycINFO™ (1806 to week 1 April 2012), Education Resources Information Center (ERIC) (searched 13 May 2012) and the CORK Database (searched 28 May 2012), and gray literature sources. [file 2046-4053-3-50-S2.pdf]

Additional file 2. *Database search strategies for Ovid MEDLINE® In-Process & Other Non-indexed Citations and Ovid MEDLINE® (1946 to April 2012), Embase Classic + Embase (1947 to 2012 April 06), The Cochrane Library (searched 2012 April 08), Cumulative Index to Nursing and Allied Health Literature (CINAHL®) (searched 2012 April 18), PsycINFO®(1806 to April Week 1 2012), Education Resources Information Center (ERIC) (searched 2012 May 13) and the CORK Database (searched 2012 May 28), and GREY LITERATURE SOURCES.*

MEDLINE, Embase, PsychINFO – RCTs/CCTs

1 SBIRT.tw. (127)

2 (SBI or SBIs).tw. (1779)

3 1 or 2 (1899)

4 Substance-Related Disorders/ (114089)

5 exp Amphetamine-Related Disorders/ (57415)

6 exp Cocaine-Related Disorders/ (13105)

7 exp Marijuana Abuse/ or exp Marijuana Smoking/ (12242)

8 exp Opioid-Related Disorders/ (26294)

9 exp Phencyclidine Abuse/ (55895)

10 Psychoses, Substance-Induced/ (70039)

11 exp Substance Abuse, Intravenous/ (44099)

12 ((substance-related or substance-induced) adj3 (disorder* or psychosis or psychoses)).tw. (1943)

13 ((drug or drugs or substance* or opioid* or opiate* or amphetamine* or amfetamine* or methamphetamine* or methamfetamine or benzodiazepine* or morphine* or methadone* or prescription* or phencyclidine* or solvent* or barbiturate* or depressant* or stimulant* or psychotherap* or psycho-therap* or steroid*) adj3 (addict* or abuse* or abusing or abusive or misuse* or mis-use* or misusing or mis-using or non-medical use* or non-medical usage* or illicit* or illegal* or unlawful* or unsanction* or habit* or dependen* or disorder or disorders or relapse* or consumption or diversion*)).tw. (284390)

14 or/4-13 (487412)

15 Dextropropoxyphene/ (8343)

16 (Dextropropoxyphene or D-Propoxyphene or Propoxyphene or Darvon or Vicodin).tw. (4113)

17 exp Methadone/ (34314)

18 (Methadone or Amidone or Biodone or Dolophine or Metadol or Metasedin or Methaddict or Methadose or Methex or Phenadone or Phymet or Physeptone or Pinadone or Symoron).tw. (27635)

19 exp Meperidine/ (27670)

20 (Meperidine or Demerol or Dolantin or Dolargan or Dolcontral or Dolin or Dolosal or Dolsin or Isonipecain or Lidol or Lydol or Operidine or Pethidine or Promedol or Dimethylmeperidine or Isopromedol or Trimeperidine or Lomotil or Reasec).tw. (15690)

21 Pentobarbital/ (37102)

22 (Pentobarbital or Diabutal or Etaminal or Ethaminal or Mebubarbital or Mebumal or Nembutal or Pentobarbitone or Sagatal).tw. (41194)

23 exp Diazepam/ (83083)

24 (Diazepam or Apaurin or Diazemuls or Faustan or Relanium or Seduxen or Sibazon or Stesolid or Valium or Nordazepam or Calmday or Dealkylprazepam or Demethyldiazepam or Deoxydemoxepam or Desmethyldiazepam or Nordaz or Nordiazepam or Norprazepam or "Tranxilium N" or Vegesan).tw. (52131)

25 ("substance use" or "substance usage").tw. (47396)

26 Alprazolam/ (13744)

27 (Alprazolam or Alprazolan or Alprox or "Apo-Alpraz" or Cassadan or Esparon or Kalma or "Novo-Alprazol" or "Nu-Alpraz" or Ralozam or Tafil or Trankimazin or Xanax).tw. (6554)

28 exp Amphetamine/ (56197)

29 (Amphetamine or Amfetamine or Centramina or Desoxynorephedrin or Fenamine or "l-Amphetamine" or levo-Amphetamine or Levoamphetamine or Mydrial or Phenamine or Phenopromin or Thyramine or Dextroamphetamine or dextro-Amphetamine or Curban or "d-Amphetamine" or Dexamfetamine or Dexamphetamine or Dexedrine or dextro-Amphetamine or DextroStat or Oxydess or dextramethorphan or dextroamethorphan or DXM or methamphetamine or methamfetamine or Deoxyephedrine or Desoxyephedrine or Desoxyn or Madrine).tw. (68766)

30 Methylphenidate/ (22483)

31 (Methylphenidate or Centedrin or Daytrana or Dexmethylphenidate or Equasym or Focalin or Metadate or Methylin or Phenidylate or Ritalin* or Tsentedrin or Adderall or Obetrol).tw. (16202)

32 Narcotics/ (26314)

33 Narcotic*.tw. (31975)

34 Analgesics, Opioid/ (38888)

35 (opioid* or opiate* or opium).tw. (170936)

36 exp Morphine/ (130775)

37 (morphine* or morphia or "MS Contin" or Oramorph or codeine or Ardinex or Isocodeine or "N-Methylmorphine" or hydrocodon* or Hydrocon or Codinovo or Dicodid or Dihydrocodeinone or Hycodan or Hycon or Hydrocodeinonebitartrate or Robidone or coactified or ratio-codeine or tylenol or Oxycodone or Dihydrohydroxycodeinone or Dihydrone or Dinarkon or Eucodal or Oxiconum or Oxycodeinon or Oxycone or Oxycontin* or Pancodine or Theocodin).tw. (109967)

38 Hydromorphone/ (6320)

39 (Hydromorphon* or Dihydromorphinone or Dilaudid or Laudacon or Palladone).tw. (3104)

40 Phencyclidine/ (11364)

41 (Phencyclidine or Sernyl or Serylan).tw. (10253)

42 Buprenorphine/ (12995)

43 (Buprenorphine or Buprenex or Buprex or Prefin or Subutex or Temgesic).tw. (10487)

44 exp Fentanyl/ (54296)

45 (Fentanyl or Duragesic or Durogesic or Fentanest or Fentora or Phentanyl or Sublimaze).tw. (32210)

46 Pentazocine/ (10799)

47 (Pentazocine or Fortral or Lexir or Talwin).tw. (6612)

48 Tramadol/ (12577)

49 (Tramadol* or Adolonta or Amadol or Biodalgic or Biokanol or Contramal or Jutadol or Nobligan or Prontofort or Ralivia or Takadol or Theradol or Tiral or Topalgic or Tradol or Tradonal or Tralgiol or Tramacet or Tramabeta or Tramadin or Tramadoc or Tramadura or Tramagetic or Tramagit or Tramake or Tramal or Tramex or Tramundin or Trasedal or Tridural or Ultram or Zamudol or Zumalgic or Zydol or Zytram).tw. (7402)

50 or/15-49 (695473)

51 50 and (addict* or abuse* or abusing or abusive or misuse* or mis-use* or misusing or mis-using or illicit* or illegal* or unlawful* or unsanction* or non-medical*).tw. (89275)

52 14 or 51 (498615)

53 exp designer drugs/ or exp street drugs/ (11613)

54 (designer drug$1 or street drug$1 or recreational drug$1 or narcotic* or non-therapeutic drug$1 or non-medical drug$1 or club drug$1 or rave drug$1 or party pill$1).tw. (38125)

55 exp Cannabis/ (31463)

56 (Cannabi* or marijuana or marihuana or hemp or hash or hashish or ganja).tw. (67171)

57 exp Heroin/ (24255)

58 (Heroin or Diacetylmorphine or Diagesil or Diamorphine or Diamorf or speed).tw. (247697)

59 exp Cocaine/ (73086)

60 (cocaine or crack).tw. (81671)

61 exp Hallucinogens/ (77182)

62 (hallucinogen* or psychedelic*).tw. (8568)

63 exp n-methyl-3,4-methylenedioxyamphetamine/ or mescaline/ or ketamine/ (44671)

64 (methylenedioxymethamphetamine or MDMA or ecstasy or methamphetamine or "3,4-methylenedioxymethamphetamine" or crystal meth or crank or mescaline or mezcalin or peyote or trimethoxyphenethylamine or ketamine or Calipsol or Calypsol or "CI-581" or Kalipsol or Ketalar or Ketanest or Ketaset or "special k" or mushroom* or prilocybin or "gamma hydroxybutrate" or GHB or PCP or angel dust).tw. (100186)

65 ((sniff* or inhal* or snort*) adj3 (solvent* or glue or drug or drugs)).tw. (5295)

66 ("inhalant use" or "inhalant usage" or "inhalant abuse").tw. (951)

67 exp Lysergic Acid Diethylamide/ (14855)

68 (LSD or Lysergide or Lysergic Acid Diethylamide).tw. (12294)

69 ("drug use" or "drug usage" or (drug adj user*)).tw. (103447)

70 or/53-69 (662848)

71 52 or 70 (1003851)

72 Psychotherapy, Brief/ (82094)

73 Crisis Intervention/ (13363)

74 72 or 73 (94774)

75 exp Motivation/ (228739)

76 exp Psychotherapy/ (474799)

77 ((cogniti* or behavior* or behaviour* or motivat* or psychosocial* or psycho-social* or psychological) adj3 (therapy or therapies or therapeutic or interven* or interview* or session* or modify or modifies or modified or modification or chang* or conditioning)).tw. (280182)

78 or/75-77 (888453)

79 (brief* or short* or short-rang* or short-term or abbreviate* or concise or early or limited or time-limited or crisis or crises or emergency or emergencies or urgent or immediate* or minimal* or minimum or quick*).tw. (6768481)

80 78 and 79 (167176)

81 (((psycho-therap* or psychotherap* or counsel* or solution-focus?ed or interven* or prevent* or advice or advis*) adj5 (brief* or short* or short-rang* or short-term or

abbreviate* or concise or early or limited or time-limited or crisis or crises or emergency or emergencies or urgent or immediate* or minimal* or minimum or quick*)) or contingency management).tw. (186202)

82 80 or 81 (324375)

83 74 or 82 (397664)

84 exp "Referral and Consultation"/ (110407)

85 (refer or refer?ing or refer?ed or refers or referral*).tw. (519480)

86 (gatekeep* or gate-keep*).tw. (7328)

87 (specialist* or specialt* or professional*).tw. (665772)

88 or/84-87 (1207011)

89 71 and (83 or 88) (67973)

90 3 or 89 (69763)

91 (controlled clinical trial or randomized controlled trial).pt. (403488)

92 exp randomized controlled trials as topic/ or exp controlled clinical trials as topic/ or exp random allocation/ or exp double-blind method/ or exp single-blind method/ or exp placebos/ (643567)

93 "controlled clinical trial".tw. (17428)

94 (random* or RCT$1 or placebo*).tw. (1594700)

95 ((singl* or doubl* or trebl* or tripl*) and (mask* or blind* or dumm*)).tw. (310383)

96 or/91-95 (2006729)

97 90 and 96 (7720)

98 limit 97 to human (7019)

99 (publisher or in process or pubmednotmedline).st. (217078)

100 97 and 99 (50)

101 98 or 100 (7069)

102 (comment or editorial or letter or interview or news).pt. (2518111)

103 101 not 102 (7054)

104 review.pt. (3526535)

105 103 not 104 (5856)

106 105 use prmz (1992)

107 SBIRT.tw. (127)

108 (SBI or SBIs).tw. (1779)

109 107 or 108 (1899)

110 exp drug dependence/ (403746)

111 exp drug abuse/ (468186)

112 exp cocaine dependence/ (13105)

113 exp cannabis addiction/ or exp cannabis smoking/ (8775)

114 exp opiate addiction/ (26294)

115 exp substance abuse/ (445463)

116 ((substance-related or substance-induced) adj3 (disorder* or psychosis or psychoses)).tw. (1943)

117 ((drug or drugs or substance* or opioid* or opiate* or amphetamine* or amfetamine* or methamphetamine* or methamfetamine or benzodiazepine* or morphine* or methadone* or prescription* or phencyclidine* or solvent* or barbiturate* or depressant* or stimulant* or psychotherap* or psycho-therap* or steroid*) adj3 (addict* or abuse* or abusing or abusive or misuse* or mis-use* or misusing or mis-using or non-medical use* or non-medical usage* or illicit* or illegal* or unlawful* or unsanction* or habit* or dependen* or disorder or disorders or relapse* or consumption or diversion*)).tw. (284390)

118 or/110-117 (685592)

119 exp dextropropoxyphene/ (8343)

120 (Dextropropoxyphene or D-Propoxyphene or Propoxyphene or Darvon or Vicodin).tw. (4113)

121 exp methadone/ (34314)

122 (Methadone or Amidone or Biodone or Dolophine or Metadol or Metasedin or Methaddict or Methadose or Methex or Phenadone or Phymet or Physeptone or Pinadone or Symoron).tw. (27635)

123 exp pethidine/ (27591)

124 (Meperidine or Demerol or Dolantin or Dolargan or Dolcontral or Dolin or Dolosal or Dolsin or Isonipecain or Lidol or Lydol or Operidine or Pethidine or Promedol or Dimethylmeperidine or Isopromedol or Trimeperidine or Lomotil or Reasec).tw. (15690)

125 exp pentobarbital/ (37102)

126 (Pentobarbital or Diabutal or Etaminal or Ethaminal or Mebubarbital or Mebumal or Nembutal or Pentobarbitone or Sagatal).tw. (41194)

127 exp diazepam/ (83083)

128 (Diazepam or Apaurin or Diazemuls or Faustan or Relanium or Seduxen or Sibazon or Stesolid or Valium or Nordazepam or Calmday or Dealkylprazepam or Demethyldiazepam or Deoxydemoxepam or Desmethyldiazepam or Nordaz or Nordiazepam or Norprazepam or "Tranxilium N" or Vegesan).tw. (52131)

129 ("substance use" or "substance usage").tw. (47396)

130 exp alprazolam/ (13744)

131 (Alprazolam or Alprazolan or Alprox or "Apo-Alpraz" or Cassadan or Esparon or Kalma or "Novo-Alprazol" or "Nu-Alpraz" or Ralozam or Tafil or Trankimazin or Xanax).tw. (6554)

132 exp amphetamine/ (56197)

133 (Amphetamine or Amfetamine or Centramina or Desoxynorephedrin or Fenamine or "l-Amphetamine" or levo-Amphetamine or Levoamphetamine or Mydrial or Phenamine or Phenopromin or Thyramine or Dextroamphetamine or dextro-Amphetamine or Curban or "d-Amphetamine" or Dexamfetamine or Dexamphetamine or Dexedrine or dextro-Amphetamine or DextroStat or Oxydess or dextramethorphan or dextroamethorphan or DXM or methamphetamine or methamfetamine or Deoxyephedrine or Desoxyephedrine or Desoxyn or Madrine).tw. (68766)

134 exp methylphenidate/ (22483)

135 (Methylphenidate or Centedrin or Daytrana or Dexmethylphenidate or Equasym or Focalin or Metadate or Methylin or Phenidylate or Ritalin* or Tsentedrin or Adderall or Obetrol).tw. (16202)

136 exp narcotic agent/ (206544)

137 Narcotic*.tw. (31975)

138 exp narcotic analgesic agent/ (234924)

139 (opioid* or opiate* or opium).tw. (170936)

140 exp morphine/ (130775)

141 (morphine* or morphia or "MS Contin" or Oramorph or codeine or Ardinex or Isocodeine or "N-Methylmorphine" or hydrocodon* or Hydrocon or Codinovo or Dicodid or Dihydrocodeinone or Hycodan or Hycon or Hydrocodeinonebitartrate or Robidone or coactified or ratio-codeine or tylenol or Oxycodone or Dihydrohydroxycodeinone or Dihydrone or Dinarkon or Eucodal or Oxiconum or Oxycodeinon or Oxycone or Oxycontin* or Pancodine or Theocodin).tw. (109967)

142 exp hydromorphone/ (6320)

143 (Hydromorphon* or Dihydromorphinone or Dilaudid or Laudacon or Palladone).tw. (3104)

144 exp phencyclidine/ (11364)

145 (Phencyclidine or Sernyl or Serylan).tw. (10253)

146 exp buprenorphine/ (12995)

147 (Buprenorphine or Buprenex or Buprex or Prefin or Subutex or Temgesic).tw. (10487)

148 exp fentanyl/ (54296)

149 (Fentanyl or Duragesic or Durogesic or Fentanest or Fentora or Phentanyl or Sublimaze).tw. (32210)

150 exp pentazocine/ (10799)

151 (Pentazocine or Fortral or Lexir or Talwin).tw. (6612)

152 exp tramadol/ (12577)

153 (Tramadol* or Adolonta or Amadol or Biodalgic or Biokanol or Contramal or Jutadol or Nobligan or Prontofort or Ralivia or Takadol or Theradol or Tiral or Topalgic or Tradol or Tradonal or Tralgiol or Tramacet or Tramabeta or Tramadin or Tramadoc or Tramadura or Tramagetic or Tramagit or Tramake or Tramal or Tramex or Tramundin or Trasedal or Tridural or Ultram or Zamudol or Zumalgic or Zydol or Zytram).tw. (7402)

154 or/119-153 (724990)

155 154 and (addict* or abuse* or abusing or abusive or misuse* or mis-use* or misusing or mis-using or illicit* or illegal* or unlawful* or unsanction* or non-medical*).tw. (91993)

156 118 or 155 (694468)

157 exp designer drug/ or exp street drug/ (3784)

158 (designer drug$1 or street drug$1 or recreational drug$1 or narcotic* or non-therapeutic drug$1 or non-medical drug$1 or club drug$1 or rave drug$1 or party pill$1).tw. (38125)

159 exp cannabis/ (31463)

160 (Cannabi* or marijuana or marihuana or hemp or hash or hashish or ganja).tw. (67171)

161 exp diamorphine/ (22401)

162 (Heroin or Diacetylmorphine or Diagesil or Diamorphine or Diamorf or speed).tw. (247697)

163 exp cocaine/ (73086)

164 (cocaine or crack).tw. (81671)

165 exp psychedelic agent/ (77182)

166 (hallucinogen* or psychedelic*).tw. (8568)

167 exp 3,4 methylenedioxymethamphetamine/ (5951)

168 exp mescaline/ or exp ketamine/ (36299)

169 (methylenedioxymethamphetamine or MDMA or ecstasy or methamphetamine or "3,4-methylenedioxymethamphetamine" or crystal meth or crank or mescaline or mezcalin or peyote or trimethoxyphenethylamine or ketamine or Calipsol or Calypsol or "CI-581" or Kalipsol or Ketalar or Ketanest or Ketaset or "special k" or mushroom* or prilocybin or "gamma hydroxybutrate" or GHB or PCP or angel dust).tw. (100186)

170 ((sniff* or inhal* or snort*) adj3 (solvent* or glue or drug or drugs)).tw. (5295)

171 ("inhalant use" or "inhalant usage" or "inhalant abuse").tw. (951)

172 exp lysergide/ (13795)

173 (LSD or Lysergide or Lysergic Acid Diethylamide).tw. (12294)

174 ("drug use" or "drug usage" or (drug adj user*)).tw. (103447)

175 or/157-174 (660188)

176 156 or 175 (1190167)

177 exp crisis intervention/ (16222)

178 exp motivation/ (228739)

179 exp psychotherapy/ (474799)

180 ((cogniti* or behavior* or behaviour* or motivat* or psychosocial* or psycho-social* or psychological) adj3 (therapy or therapies or therapeutic or interven* or interview* or session* or modify or modifies or modified or modification or chang* or conditioning)).tw. (280182)

181 or/178-180 (888453)

182 (brief* or short* or short-rang* or short-term or abbreviate* or concise or early or limited or time-limited or crisis or crises or emergency or emergencies or urgent or immediate* or minimal* or minimum or quick*).tw. (6768481)

183 181 and 182 (167176)

184 (((psycho-therap* or psychotherap* or counsel* or solution-focus?ed or interven* or prevent* or advice or advis*) adj5 (brief* or short* or short-rang* or short-term or abbreviate* or concise or early or limited or time-limited or crisis or crises or emergency or emergencies or urgent or immediate* or minimal* or minimum or quick*)) or contingency management).tw. (186202)

185 183 or 184 (324375)

186 177 or 185 (333433)

187 exp patient referral/ (58835)

188 (refer or refer?ing or refer?ed or refers or referral*).tw. (519480)

189 (gatekeep* or gate-keep*).tw. (7328)

190 (specialist* or specialt* or professional*).tw. (665772)

191 or/187-190 (1177259)

192 176 and (186 or 191) (66345)

193 limit 192 to (randomized controlled trial or controlled clinical trial) [Limit not valid in PsycINFO; records were retained] (20923)

194 exp "randomized controlled trial (topic)"/ (14643)

195 exp "controlled clinical trial (topic)"/ (15252)

196 exp randomization/ (131645)

197 double blind procedure/ (112772)

198 exp placebo/ (215881)

199 "controlled clinical trial".tw. (17428)

200 (random* or RCT$1 or placebo*).tw. (1594700)

201 ((singl* or doubl* or trebl* or tripl*) and (mask* or blind* or dumm*)).tw. (310383)

202 or/194-201 (1830861)

203 192 and 202 (7331)

204 193 or 203 (24196)

205 limit 204 to human (22894)

206 (editorial or letter or note).pt. (2765265)

207 205 not 206 (22883)

208 (review or short survey).pt. (3812553)

209 207 not 208 (21923)

210 209 use emczd (2154)

211 SBIRT.tw. (127)

212 (SBI or SBIs).tw. (1779)

213 211 or 212 (1899)

214 exp Drug Abuse/ (468186)

215 exp Marijuana Usage/ (1733)

216 ((substance-related or substance-induced) adj3 (disorder* or psychosis or psychoses)).tw. (1943)

217 ((drug or drugs or polydrug$1 or substance* or opioid* or opiate* or amphetamine* or amfetamine* or methamphetamine* or methamfetamine or benzodiazepine* or morphine* or methadone* or prescription* or phencyclidine* or solvent* or barbiturate* or depressant* or stimulant* or psychotherap* or psycho-therap* or steroid*) adj3 (addict* or abuse* or abusing or abusive or misuse* or mis-use* or misusing or mis-using or non-medical use* or non-medical usage* or illicit* or illegal* or unlawful* or unsanction* or habit* or dependen* or disorder or disorders or relapse* or consumption or diversion*)).tw. (284730)

218 or/214-217 (643416)

219 (Dextropropoxyphene or D-Propoxyphene or Propoxyphene or Darvon or Vicodin).tw. (4113)

220 exp Methadone/ (34314)

221 (Methadone or Amidone or Biodone or Dolophine or Metadol or Metasedin or Methaddict or Methadose or Methex or Phenadone or Phymet or Physeptone or Pinadone or Symoron).tw. (27635)

222 exp Meperidine/ (27670)

223 (Meperidine or Demerol or Dolantin or Dolargan or Dolcontral or Dolin or Dolosal or Dolsin or Isonipecain or Lidol or Lydol or Operidine or Pethidine or Promedol or Dimethylmeperidine or Isopromedol or Trimeperidine or Lomotil or Reasec).tw. (15690)

224 exp Pentobarbital/ (37102)

225 (Pentobarbital or Diabutal or Etaminal or Ethaminal or Mebubarbital or Mebumal or Nembutal or Pentobarbitone or Sagatal).tw. (41194)

226 exp Diazepam/ (83083)

227 (Diazepam or Apaurin or Diazemuls or Faustan or Relanium or Seduxen or Sibazon or Stesolid or Valium or Nordazepam or Calmday or Dealkylprazepam or Demethyldiazepam or Deoxydemoxepam or Desmethyldiazepam or Nordaz or Nordiazepam or Norprazepam or "Tranxilium N" or Vegesan).tw. (52131)

228 ("substance use" or "substance usage").tw. (47396)

229 exp Alprazolam/ (13744)

230 (Alprazolam or Alprazolan or Alprox or "Apo-Alpraz" or Cassadan or Esparon or Kalma or "Novo-Alprazol" or "Nu-Alpraz" or Ralozam or Tafil or Trankimazin or Xanax).tw. (6554)

231 exp Amphetamine/ (56197)

232 (Amphetamine or Amfetamine or Centramina or Desoxynorephedrin or Fenamine or "l-Amphetamine" or levo-Amphetamine or Levoamphetamine or Mydrial or Phenamine or Phenopromin or Thyramine or Dextroamphetamine or dextro-Amphetamine or Curban or "d-Amphetamine" or Dexamfetamine or Dexamphetamine or Dexedrine or dextro-Amphetamine or DextroStat or Oxydess or dextramethorphan or dextroamethorphan or DXM or methamphetamine or methamfetamine or Deoxyephedrine or Desoxyephedrine or Desoxyn or Madrine).tw. (68766)

233 exp Methylphenidate/ (22483)

234 (Methylphenidate or Centedrin or Daytrana or Dexmethylphenidate or Equasym or Focalin or Metadate or Methylin or Phenidylate or Ritalin* or Tsentedrin or Adderall or Obetrol).tw. (16202)

235 exp Narcotic Drugs/ (20093)

236 Narcotic*.tw. (31975)

237 (opioid* or opiate* or opium).tw. (170936)

238 exp Morphine/ (130775)

239 (morphine* or morphia or "MS Contin" or Oramorph or codeine or Ardinex or Isocodeine or "N-Methylmorphine" or hydrocodon* or Hydrocon or Codinovo or Dicodid or Dihydrocodeinone or Hycodan or Hycon or Hydrocodeinonebitartrate or Robidone or coactified or ratio-codeine or tylenol or Oxycodone or Dihydrohydroxycodeinone or Dihydrone or Dinarkon or Eucodal or Oxiconum or Oxycodeinon or Oxycone or Oxycontin* or Pancodine or Theocodin).tw. (109967)

240 (Hydromorphon* or Dihydromorphinone or Dilaudid or Laudacon or Palladone).tw. (3104)

241 exp Phencyclidine/ (11364)

242 (Phencyclidine or Sernyl or Serylan).tw. (10253)

243 (Buprenorphine or Buprenex or Buprex or Prefin or Subutex or Temgesic).tw. (10487)

244 exp Fentanyl/ (54296)

245 (Fentanyl or Duragesic or Durogesic or Fentanest or Fentora or Phentanyl or Sublimaze).tw. (32210)

246 exp Pentazocine/ (10799)

247 (Pentazocine or Fortral or Lexir or Talwin).tw. (6612)

248 exp Tramadol/ (12577)

249 (Tramadol* or Adolonta or Amadol or Biodalgic or Biokanol or Contramal or Jutadol or Nobligan or Prontofort or Ralivia or Takadol or Theradol or Tiral or Topalgic or Tradol or Tradonal or Tralgiol or Tramacet or Tramabeta or Tramadin or Tramadoc or Tramadura or Tramagetic or Tramagit or Tramake or Tramal or Tramex or Tramundin or Trasedal or Tridural or Ultram or Zamudol or Zumalgic or Zydol or Zytram).tw. (7402)

250 or/219-249 (681460)

251 250 and (addict* or abuse* or abusing or abusive or misuse* or mis-use* or misusing or mis-using or illicit* or illegal* or unlawful* or unsanction* or non-medical*).tw. (88298)

252 218 or 251 (655243)

253 (designer drug$1 or street drug$1 or recreational drug$1 or narcotic* or non-therapeutic drug$1 or non-medical drug$1 or club drug$1 or rave drug$1 or party pill$1).tw. (38125)

254 exp Cannabis/ (31463)

255 (Cannabi* or marijuana or marihuana or hemp or hash or hashish or ganja).tw. (67171)

256 exp Heroin/ (24255)

257 (Heroin or Diacetylmorphine or Diagesil or Diamorphine or Diamorf or speed).tw. (247697)

258 exp cocaine/ (73086)

259 (cocaine or crack).tw. (81671)

260 exp hallucinogenic drugs/ (23375)

261 (hallucinogen* or psychedelic*).tw. (8568)

262 exp methylenedioxymethamphetamine/ (10424)

263 exp mescaline/ or exp ketamine/ (36299)

264 (methylenedioxymethamphetamine or MDMA or ecstasy or methamphetamine or "3,4-methylenedioxymethamphetamine" or crystal meth or crank or mescaline or mezcalin or peyote or trimethoxyphenethylamine or ketamine or Calipsol or Calypsol or "CI-581" or Kalipsol or Ketalar or Ketanest or Ketaset or "special k" or mushroom* or prilocybin or "gamma hydroxybutrate" or GHB or PCP or angel dust).tw. (100186)

265 ((sniff* or inhal* or snort*) adj3 (solvent* or glue or drug or drugs)).tw. (5295)

266 ("inhalant use" or "inhalant usage" or "inhalant abuse").tw. (951)

267 exp Lysergic Acid Diethylamide/ (14855)

268 (LSD or Lysergide or Lysergic Acid Diethylamide).tw. (12294)

269 ("drug use" or "drug usage" or (drug adj user*)).tw. (103447)

270 or/253-269 (648721)

271 252 or 270 (1157219)

272 exp brief psychotherapy/ (6979)

273 exp Crisis Intervention/ (16222)

274 272 or 273 (23013)

275 exp motivation/ or exp motivation training/ (228911)

276 exp psychotherapy/ (474799)

277 ((cogniti* or behavior* or behaviour* or motivat* or psychosocial* or psycho-social* or psychological) adj3 (therapy or therapies or therapeutic or interven* or interview* or session* or modify or modifies or modified or modification or chang* or conditioning)).tw. (280182)

278 or/275-277 (888533)

279 (brief* or short* or short-rang* or short-term or abbreviate* or concise or early or limited or time-limited or crisis or crises or emergency or emergencies or urgent or immediate* or minimal* or minimum or quick*).tw. (6768481)

280 278 and 279 (167186)

281 (((psycho-therap* or psychotherap* or counsel* or solution-focus?ed or interven* or prevent* or advice or advis*) adj5 (brief* or short* or short-rang* or short-term or abbreviate* or concise or early or limited or time-limited or crisis or crises or emergency or emergencies or urgent or immediate* or minimal* or minimum or quick*)) or contingency management).tw. (186202)

282 280 or 281 (324382)

283 274 or 282 (334216)

284 exp professional referral/ (2878)

285 (refer or refer?ing or refer?ed or refers or referral*).tw. (519480)

286 (gatekeep* or gate-keep*).tw. (7328)

287 (specialist* or specialt* or professional*).tw. (665772)

288 or/284-287 (1149894)

289 271 and (283 or 288) (64106)

290 213 or 289 (65807)

291 limit 290 to "2000 treatment outcome/randomized clinical trial" [Limit not valid in Embase,Ovid MEDLINE(R),Ovid MEDLINE(R) In-Process; records were retained] (48204)

292 exp Random Sampling/ (531)

293 exp Placebo/ (215881)

294 "controlled clinical trial".tw. (17428)

295 (random* or RCT$1 or placebo*).tw. (1594700)

296 ((singl* or doubl* or trebl* or tripl*) and (mask* or blind* or dumm*)).tw. (310383)

297 or/292-296 (1759189)

298 290 and 297 (7253)

299 291 or 298 (49527)

300 limit 299 to human (41757)

301 300 use psyc1 (2)

302 300 use psyc2 (41)

303 300 use psyc3 (374)

304 300 use psyc4 (258)

305 300 use psyc5 (450)

306 300 use psyc6 (462)

307 300 use psyc7 (230)

308 or/301-307 (1817)

309 106 or 210 or 308 (5963)

310 remove duplicates from 309 (3715)

311 310 use prmz (1930) **MEDLINE records**

312 310 use emczd (766) **Embase records**

313 310 not (311 or 312) (1019) **PsycINFO records**

MEDLINE, Embase, PsychINFO – Reviews

1 SBIRT.tw. (127)

2 (SBI or SBIs).tw. (1779)

3 1 or 2 (1899)

4 Substance-Related Disorders/ (114089)

5 exp Amphetamine-Related Disorders/ (57415)

6 exp Cocaine-Related Disorders/ (13105)

7 exp Marijuana Abuse/ or exp Marijuana Smoking/ (12242)

8 exp Opioid-Related Disorders/ (26294)

9 exp Phencyclidine Abuse/ (55895)

10 Psychoses, Substance-Induced/ (70039)

11 exp Substance Abuse, Intravenous/ (44099)

12 ((substance-related or substance-induced) adj3 (disorder* or psychosis or psychoses)).tw. (1943)

13 ((drug or drugs or substance* or opioid* or opiate* or amphetamine* or amfetamine* or methamphetamine* or methamfetamine or benzodiazepine* or morphine* or methadone* or prescription* or phencyclidine* or solvent* or barbiturate* or depressant* or stimulant* or psychotherap* or psycho-therap* or steroid*) adj3 (addict* or abuse* or abusing or abusive or misuse* or mis-use* or misusing or mis-using or non-medical use* or non-medical usage* or illicit* or illegal* or unlawful* or unsanction* or habit* or dependen* or disorder or disorders or relapse* or consumption or diversion*)).tw. (284390)

14 or/4-13 (487412)

15 Dextropropoxyphene/ (8343)

16 (Dextropropoxyphene or D-Propoxyphene or Propoxyphene or Darvon or Vicodin).tw. (4113)

17 exp Methadone/ (34314)

18 (Methadone or Amidone or Biodone or Dolophine or Metadol or Metasedin or Methaddict or Methadose or Methex or Phenadone or Phymet or Physeptone or Pinadone or Symoron).tw. (27635)

19 exp Meperidine/ (27670)

20 (Meperidine or Demerol or Dolantin or Dolargan or Dolcontral or Dolin or Dolosal or Dolsin or Isonipecain or Lidol or Lydol or Operidine or Pethidine or Promedol or Dimethylmeperidine or Isopromedol or Trimeperidine or Lomotil or Reasec).tw. (15690)

21 Pentobarbital/ (37102)

22 (Pentobarbital or Diabutal or Etaminal or Ethaminal or Mebubarbital or Mebumal or Nembutal or Pentobarbitone or Sagatal).tw. (41194)

23 exp Diazepam/ (83083)

24 (Diazepam or Apaurin or Diazemuls or Faustan or Relanium or Seduxen or Sibazon or Stesolid or Valium or Nordazepam or Calmday or Dealkylprazepam or Demethyldiazepam or Deoxydemoxepam or Desmethyldiazepam or Nordaz or Nordiazepam or Norprazepam or "Tranxilium N" or Vegesan).tw. (52131)

25 ("substance use" or "substance usage").tw. (47396)

26 Alprazolam/ (13744)

27 (Alprazolam or Alprazolan or Alprox or "Apo-Alpraz" or Cassadan or Esparon or Kalma or "Novo-Alprazol" or "Nu-Alpraz" or Ralozam or Tafil or Trankimazin or Xanax).tw. (6554)

28 exp Amphetamine/ (56197)

29 (Amphetamine or Amfetamine or Centramina or Desoxynorephedrin or Fenamine or "l-Amphetamine" or levo-Amphetamine or Levoamphetamine or Mydrial or Phenamine or Phenopromin or Thyramine or Dextroamphetamine or dextro-Amphetamine or Curban or "d-Amphetamine" or Dexamfetamine or Dexamphetamine or Dexedrine or dextro-Amphetamine or DextroStat or Oxydess or dextramethorphan or dextroamethorphan or DXM or methamphetamine or methamfetamine or Deoxyephedrine or Desoxyephedrine or Desoxyn or Madrine).tw. (68766)

30 Methylphenidate/ (22483)

31 (Methylphenidate or Centedrin or Daytrana or Dexmethylphenidate or Equasym or Focalin or Metadate or Methylin or Phenidylate or Ritalin* or Tsentedrin or Adderall or Obetrol).tw. (16202)

32 Narcotics/ (26314)

33 Narcotic*.tw. (31975)

34 Analgesics, Opioid/ (38888)

35 (opioid* or opiate* or opium).tw. (170936)

36 exp Morphine/ (130775)

37 (morphine* or morphia or "MS Contin" or Oramorph or codeine or Ardinex or Isocodeine or "N-Methylmorphine" or hydrocodon* or Hydrocon or Codinovo or Dicodid or Dihydrocodeinone or Hycodan or Hycon or Hydrocodeinonebitartrate or Robidone or coactified or ratio-codeine or tylenol or Oxycodone or Dihydrohydroxycodeinone or Dihydrone or Dinarkon or Eucodal or Oxiconum or Oxycodeinon or Oxycone or Oxycontin* or Pancodine or Theocodin).tw. (109967)

38 Hydromorphone/ (6320)

39 (Hydromorphon* or Dihydromorphinone or Dilaudid or Laudacon or Palladone).tw. (3104)

40 Phencyclidine/ (11364)

41 (Phencyclidine or Sernyl or Serylan).tw. (10253)

42 Buprenorphine/ (12995)

43 (Buprenorphine or Buprenex or Buprex or Prefin or Subutex or Temgesic).tw. (10487)

44 exp Fentanyl/ (54296)

45 (Fentanyl or Duragesic or Durogesic or Fentanest or Fentora or Phentanyl or Sublimaze).tw. (32210)

46 Pentazocine/ (10799)

47 (Pentazocine or Fortral or Lexir or Talwin).tw. (6612)

48 Tramadol/ (12577)

49 (Tramadol* or Adolonta or Amadol or Biodalgic or Biokanol or Contramal or Jutadol or Nobligan or Prontofort or Ralivia or Takadol or Theradol or Tiral or Topalgic or Tradol or Tradonal or Tralgiol or Tramacet or Tramabeta or Tramadin or Tramadoc or Tramadura or Tramagetic or Tramagit or Tramake or Tramal or Tramex or Tramundin or Trasedal or Tridural or Ultram or Zamudol or Zumalgic or Zydol or Zytram).tw. (7402)

50 or/15-49 (695473)

51 50 and (addict* or abuse* or abusing or abusive or misuse* or mis-use* or misusing or mis-using or illicit* or illegal* or unlawful* or unsanction* or non-medical*).tw. (89275)

52 14 or 51 (498615)

53 exp designer drugs/ or exp street drugs/ (11613)

54 (designer drug$1 or street drug$1 or recreational drug$1 or narcotic* or non-therapeutic drug$1 or non-medical drug$1 or club drug$1 or rave drug$1 or party pill$1).tw. (38125)

55 exp Cannabis/ (31463)

56 (Cannabi* or marijuana or marihuana or hemp or hash or hashish or ganja).tw. (67171)

57 exp Heroin/ (24255)

58 (Heroin or Diacetylmorphine or Diagesil or Diamorphine or Diamorf or speed).tw. (247697)

59 exp Cocaine/ (73086)

60 (cocaine or crack).tw. (81671)

61 exp Hallucinogens/ (77182)

62 (hallucinogen* or psychedelic*).tw. (8568)

63 exp n-methyl-3,4-methylenedioxyamphetamine/ or mescaline/ or ketamine/ (44671)

64 (methylenedioxymethamphetamine or MDMA or ecstasy or methamphetamine or "3,4-methylenedioxymethamphetamine" or crystal meth or crank or mescaline or mezcalin or peyote or trimethoxyphenethylamine or ketamine or Calipsol or Calypsol or "CI-581" or Kalipsol or Ketalar or Ketanest or Ketaset or "special k" or mushroom* or prilocybin or "gamma hydroxybutrate" or GHB or PCP or angel dust).tw. (100186)

65 ((sniff* or inhal* or snort*) adj3 (solvent* or glue or drug or drugs)).tw. (5295)

66 ("inhalant use" or "inhalant usage" or "inhalant abuse").tw. (951)

67 exp Lysergic Acid Diethylamide/ (14855)

68 (LSD or Lysergide or Lysergic Acid Diethylamide).tw. (12294)

69 ("drug use" or "drug usage" or (drug adj user*)).tw. (103447)

70 or/53-69 (662848)

71 52 or 70 (1003851)

72 Psychotherapy, Brief/ (82094)

73 Crisis Intervention/ (13363)

74 72 or 73 (94774)

75 exp Motivation/ (228739)

76 exp Psychotherapy/ (474799)

77 ((cogniti* or behavior* or behaviour* or motivat* or psychosocial* or psycho-social* or psychological) adj3 (therapy or therapies or therapeutic or interven* or interview* or session* or modify or modifies or modified or modification or chang* or conditioning)).tw. (280182)

78 or/75-77 (888453)

79 (brief* or short* or short-rang* or short-term or abbreviate* or concise or early or limited or time-limited or crisis or crises or emergency or emergencies or urgent or immediate* or minimal* or minimum or quick*).tw. (6768481)

80 78 and 79 (167176)

81 (((psycho-therap* or psychotherap* or counsel* or solution-focus?ed or interven* or prevent* or advice or advis*) adj5 (brief* or short* or short-rang* or short-term or abbreviate* or concise or early or limited or time-limited or crisis or crises or emergency or emergencies or urgent or immediate* or minimal* or minimum or quick*)) or contingency management).tw. (186202)

82 80 or 81 (324375)

83 74 or 82 (397664)

84 exp "Referral and Consultation"/ (110407)

85 (refer or refer?ing or refer?ed or refers or referral*).tw. (519480)

86 (gatekeep* or gate-keep*).tw. (7328)

87 (specialist* or specialt* or professional*).tw. (665772)

88 or/84-87 (1207011)

89 71 and (83 or 88) (67973)

90 3 or 89 (69763)

91 limit 90 to systematic reviews [Limit not valid in Embase; records were retained] (37183)

92 meta analysis.pt. (32859)

93 exp meta-analysis as topic/ (15377)

94 (meta-analy* or metanaly* or metaanaly* or met analy* or integrative research or integrative review* or integrative overview* or research integration or research overview* or collaborative review*).tw. (115383)

95 (systematic review* or systematic overview* or evidence-based review* or evidence-based overview* or (evidence adj3 (review* or overview*)) or meta-review* or meta-overview* or "review of reviews" or technology assessment* or HTA or HTAs).tw. (143440)

96 exp Technology assessment, biomedical/ (19883)

97 health technology assessment winchester england.jn. (1115)

98 (evidence report technology assessment or evidence report technology assessment summary).jn. (383)

99 or/92-98 (265347)

100 90 and 99 (1321)

101 91 or 100 (37477)

102 limit 101 to human (29620)

103 (publisher or in process or pubmednotmedline).st. (217078)

104 101 and 103 (19)

105 102 or 104 (29639)

106 (comment or letter or editorial or interview or news).pt. (2518111)

107 105 not 106 (29113)

108 107 (29113)

109 limit 108 to yr="2010 -Current" (5522)

110 109 use prmz (169)

111 SBIRT.tw. (127)

112 (SBI or SBIs).tw. (1779)

113 111 or 112 (1899)

114 exp drug dependence/ (403746)

115 exp drug abuse/ (468186)

116 exp cocaine dependence/ (13105)

117 exp cannabis addiction/ or exp cannabis smoking/ (8775)

118 exp opiate addiction/ (26294)

119 exp substance abuse/ (445463)

120 ((substance-related or substance-induced) adj3 (disorder* or psychosis or psychoses)).tw. (1943)

121 ((drug or drugs or substance* or opioid* or opiate* or amphetamine* or amfetamine* or methamphetamine* or methamfetamine or benzodiazepine* or morphine* or methadone* or prescription* or phencyclidine* or solvent* or barbiturate* or depressant* or stimulant* or psychotherap* or psycho-therap* or steroid*) adj3 (addict* or abuse* or abusing or abusive or misuse* or mis-use* or misusing or mis-using or non-medical use* or non-medical usage* or illicit* or illegal* or unlawful* or unsanction* or habit* or dependen* or disorder or disorders or relapse* or consumption or diversion*)).tw. (284390)

122 or/114-121 (685592)

123 exp dextropropoxyphene/ (8343)

124 (Dextropropoxyphene or D-Propoxyphene or Propoxyphene or Darvon or Vicodin).tw. (4113)

125 exp methadone/ (34314)

126 (Methadone or Amidone or Biodone or Dolophine or Metadol or Metasedin or Methaddict or Methadose or Methex or Phenadone or Phymet or Physeptone or Pinadone or Symoron).tw. (27635)

127 exp pethidine/ (27591)

128 (Meperidine or Demerol or Dolantin or Dolargan or Dolcontral or Dolin or Dolosal or Dolsin or Isonipecain or Lidol or Lydol or Operidine or Pethidine or Promedol or Dimethylmeperidine or Isopromedol or Trimeperidine or Lomotil or Reasec).tw. (15690)

129 exp pentobarbital/ (37102)

130 (Pentobarbital or Diabutal or Etaminal or Ethaminal or Mebubarbital or Mebumal or Nembutal or Pentobarbitone or Sagatal).tw. (41194)

131 exp diazepam/ (83083)

132 (Diazepam or Apaurin or Diazemuls or Faustan or Relanium or Seduxen or Sibazon or Stesolid or Valium or Nordazepam or Calmday or Dealkylprazepam or Demethyldiazepam or Deoxydemoxepam or Desmethyldiazepam or Nordaz or Nordiazepam or Norprazepam or "Tranxilium N" or Vegesan).tw. (52131)

133 ("substance use" or "substance usage").tw. (47396)

134 exp alprazolam/ (13744)

135 (Alprazolam or Alprazolan or Alprox or "Apo-Alpraz" or Cassadan or Esparon or Kalma or "Novo-Alprazol" or "Nu-Alpraz" or Ralozam or Tafil or Trankimazin or Xanax).tw. (6554)

136 exp amphetamine/ (56197)

137 (Amphetamine or Amfetamine or Centramina or Desoxynorephedrin or Fenamine or "l-Amphetamine" or levo-Amphetamine or Levoamphetamine or Mydrial or Phenamine or Phenopromin or Thyramine or Dextroamphetamine or dextro-Amphetamine or Curban or "d-Amphetamine" or Dexamfetamine or Dexamphetamine or Dexedrine or dextro-Amphetamine or DextroStat or Oxydess or dextramethorphan or dextroamethorphan or DXM or methamphetamine or methamfetamine or Deoxyephedrine or Desoxyephedrine or Desoxyn or Madrine).tw. (68766)

138 exp methylphenidate/ (22483)

139 (Methylphenidate or Centedrin or Daytrana or Dexmethylphenidate or Equasym or Focalin or Metadate or Methylin or Phenidylate or Ritalin* or Tsentedrin or Adderall or Obetrol).tw. (16202)

140 exp narcotic agent/ (206544)

141 Narcotic*.tw. (31975)

142 exp narcotic analgesic agent/ (234924)

143 (opioid* or opiate* or opium).tw. (170936)

144 exp morphine/ (130775)

145 (morphine* or morphia or "MS Contin" or Oramorph or codeine or Ardinex or Isocodeine or "N-Methylmorphine" or hydrocodon* or Hydrocon or Codinovo or Dicodid or Dihydrocodeinone or Hycodan or Hycon or Hydrocodeinonebitartrate or Robidone or coactified or ratio-codeine or tylenol or Oxycodone or Dihydrohydroxycodeinone or Dihydrone or Dinarkon or Eucodal or Oxiconum or Oxycodeinon or Oxycone or Oxycontin* or Pancodine or Theocodin).tw. (109967)

146 exp hydromorphone/ (6320)

147 (Hydromorphon* or Dihydromorphinone or Dilaudid or Laudacon or Palladone).tw. (3104)

148 exp phencyclidine/ (11364)

149 (Phencyclidine or Sernyl or Serylan).tw. (10253)

150 exp buprenorphine/ (12995)

151 (Buprenorphine or Buprenex or Buprex or Prefin or Subutex or Temgesic).tw. (10487)

152 exp fentanyl/ (54296)

153 (Fentanyl or Duragesic or Durogesic or Fentanest or Fentora or Phentanyl or Sublimaze).tw. (32210)

154 exp pentazocine/ (10799)

155 (Pentazocine or Fortral or Lexir or Talwin).tw. (6612)

156 exp tramadol/ (12577)

157 (Tramadol* or Adolonta or Amadol or Biodalgic or Biokanol or Contramal or Jutadol or Nobligan or Prontofort or Ralivia or Takadol or Theradol or Tiral or Topalgic or Tradol or Tradonal or Tralgiol or Tramacet or Tramabeta or Tramadin or Tramadoc or Tramadura or Tramagetic or Tramagit or Tramake or Tramal or Tramex or Tramundin or Trasedal or Tridural or Ultram or Zamudol or Zumalgic or Zydol or Zytram).tw. (7402)

158 or/123-157 (724990)

159 158 and (addict* or abuse* or abusing or abusive or misuse* or mis-use* or misusing or mis-using or illicit* or illegal* or unlawful* or unsanction* or non-medical*).tw. (91993)

160 122 or 159 (694468)

161 exp designer drug/ or exp street drug/ (3784)

162 (designer drug$1 or street drug$1 or recreational drug$1 or narcotic* or non-therapeutic drug$1 or non-medical drug$1 or club drug$1 or rave drug$1 or party pill$1).tw. (38125)

163 exp cannabis/ (31463)

164 (Cannabi* or marijuana or marihuana or hemp or hash or hashish or ganja).tw. (67171)

165 exp diamorphine/ (22401)

166 (Heroin or Diacetylmorphine or Diagesil or Diamorphine or Diamorf or speed).tw. (247697)

167 exp cocaine/ (73086)

168 (cocaine or crack).tw. (81671)

169 exp psychedelic agent/ (77182)

170 (hallucinogen* or psychedelic*).tw. (8568)

171 exp 3,4 methylenedioxymethamphetamine/ (5951)

172 exp mescaline/ or exp ketamine/ (36299)

173 (methylenedioxymethamphetamine or MDMA or ecstasy or methamphetamine or "3,4-methylenedioxymethamphetamine" or crystal meth or crank or mescaline or mezcalin or peyote or trimethoxyphenethylamine or ketamine or Calipsol or Calypsol or "CI-581" or Kalipsol or Ketalar or Ketanest or Ketaset or "special k" or mushroom* or prilocybin or "gamma hydroxybutrate" or GHB or PCP or angel dust).tw. (100186)

174 ((sniff* or inhal* or snort*) adj3 (solvent* or glue or drug or drugs)).tw. (5295)

175 ("inhalant use" or "inhalant usage" or "inhalant abuse").tw. (951)

176 exp lysergide/ (13795)

177 (LSD or Lysergide or Lysergic Acid Diethylamide).tw. (12294)

178 ("drug use" or "drug usage" or (drug adj user*)).tw. (103447)

179 or/161-178 (660188)

180 160 or 179 (1190167)

181 exp crisis intervention/ (16222)

182 exp motivation/ (228739)

183 exp psychotherapy/ (474799)

184 ((cogniti* or behavior* or behaviour* or motivat* or psychosocial* or psycho-social* or psychological) adj3 (therapy or therapies or therapeutic or interven* or interview* or session* or modify or modifies or modified or modification or chang* or conditioning)).tw. (280182)

185 or/182-184 (888453)

186 (brief* or short* or short-rang* or short-term or abbreviate* or concise or early or limited or time-limited or crisis or crises or emergency or emergencies or urgent or immediate* or minimal* or minimum or quick*).tw. (6768481)

187 185 and 186 (167176)

188 (((psycho-therap* or psychotherap* or counsel* or solution-focus?ed or interven* or prevent* or advice or advis*) adj5 (brief* or short* or short-rang* or short-term or abbreviate* or concise or early or limited or time-limited or crisis or crises or emergency or emergencies or urgent or immediate* or minimal* or minimum or quick*)) or contingency management).tw. (186202)

189 187 or 188 (324375)

190 181 or 189 (333433)

191 exp patient referral/ (58835)

192 (refer or refer?ing or refer?ed or refers or referral*).tw. (519480)

193 (gatekeep* or gate-keep*).tw. (7328)

194 (specialist* or specialt* or professional*).tw. (665772)

195 or/191-194 (1177259)

196 180 and (190 or 195) (66345)

197 limit 196 to "systematic review" [Limit not valid in Ovid MEDLINE(R),Ovid MEDLINE(R) In-Process; records were retained] (23736)

198 exp "meta analysis (topic)"/ (3368)

199 (meta-analy* or metanaly* or metaanaly* or met analy* or integrative research or integrative review* or integrative overview* or research integration or research overview* or collaborative review*).tw. (115383)

200 (systematic review* or systematic overview* or evidence-based review* or evidence-based overview* or (evidence adj3 (review* or overview*)) or meta-review* or meta-overview* or "review of reviews" or technology assessment* or HTA or HTAs).tw. (143440)

201 exp biomedical technology assessment/ (19883)

202 or/198-201 (251084)

203 196 and 202 (1239)

204 197 or 203 (24324)

205 limit 204 to human (21762)

206 (editorial or letter or note).pt. (2765265)

207 205 not 206 (21606)

208 207 (21606)

209 limit 208 to yr="2010 -Current" (3179)

210 209 use emczd (131)

211 SBIRT.tw. (127)

212 (SBI or SBIs).tw. (1779)

213 211 or 212 (1899)

214 exp Drug Abuse/ (468186)

215 exp Marijuana Usage/ (1733)

216 ((substance-related or substance-induced) adj3 (disorder* or psychosis or psychoses)).tw. (1943)

217 ((drug or drugs or polydrug$1 or substance* or opioid* or opiate* or amphetamine* or amfetamine* or methamphetamine* or methamfetamine or benzodiazepine* or morphine* or methadone* or prescription* or phencyclidine* or solvent* or barbiturate* or depressant* or stimulant* or psychotherap* or psycho-therap* or steroid*) adj3 (addict* or abuse* or abusing or abusive or misuse* or mis-use* or misusing or mis-using or non-medical use* or non-medical usage* or illicit* or illegal* or unlawful* or unsanction* or habit* or dependen* or disorder or disorders or relapse* or consumption or diversion*)).tw. (284730)

218 or/214-217 (643416)

219 (Dextropropoxyphene or D-Propoxyphene or Propoxyphene or Darvon or Vicodin).tw. (4113)

220 exp Methadone/ (34314)

221 (Methadone or Amidone or Biodone or Dolophine or Metadol or Metasedin or Methaddict or Methadose or Methex or Phenadone or Phymet or Physeptone or Pinadone or Symoron).tw. (27635)

222 exp Meperidine/ (27670)

223 (Meperidine or Demerol or Dolantin or Dolargan or Dolcontral or Dolin or Dolosal or Dolsin or Isonipecain or Lidol or Lydol or Operidine or Pethidine or Promedol or Dimethylmeperidine or Isopromedol or Trimeperidine or Lomotil or Reasec).tw. (15690)

224 exp Pentobarbital/ (37102)

225 (Pentobarbital or Diabutal or Etaminal or Ethaminal or Mebubarbital or Mebumal or Nembutal or Pentobarbitone or Sagatal).tw. (41194)

226 exp Diazepam/ (83083)

227 (Diazepam or Apaurin or Diazemuls or Faustan or Relanium or Seduxen or Sibazon or Stesolid or Valium or Nordazepam or Calmday or Dealkylprazepam or Demethyldiazepam or Deoxydemoxepam or Desmethyldiazepam or Nordaz or Nordiazepam or Norprazepam or "Tranxilium N" or Vegesan).tw. (52131)

228 ("substance use" or "substance usage").tw. (47396)

229 exp Alprazolam/ (13744)

230 (Alprazolam or Alprazolan or Alprox or "Apo-Alpraz" or Cassadan or Esparon or Kalma or "Novo-Alprazol" or "Nu-Alpraz" or Ralozam or Tafil or Trankimazin or Xanax).tw. (6554)

231 exp Amphetamine/ (56197)

232 (Amphetamine or Amfetamine or Centramina or Desoxynorephedrin or Fenamine or "l-Amphetamine" or levo-Amphetamine or Levoamphetamine or Mydrial or Phenamine or Phenopromin or Thyramine or Dextroamphetamine or dextro-Amphetamine or Curban or "d-Amphetamine" or Dexamfetamine or Dexamphetamine or Dexedrine or dextro-Amphetamine or DextroStat or Oxydess or dextramethorphan or dextroamethorphan or DXM or methamphetamine or methamfetamine or Deoxyephedrine or Desoxyephedrine or Desoxyn or Madrine).tw. (68766)

233 exp Methylphenidate/ (22483)

234 (Methylphenidate or Centedrin or Daytrana or Dexmethylphenidate or Equasym or Focalin or Metadate or Methylin or Phenidylate or Ritalin* or Tsentedrin or Adderall or Obetrol).tw. (16202)

235 exp Narcotic Drugs/ (20093)

236 Narcotic*.tw. (31975)

237 (opioid* or opiate* or opium).tw. (170936)

238 exp Morphine/ (130775)

239 (morphine* or morphia or "MS Contin" or Oramorph or codeine or Ardinex or Isocodeine or "N-Methylmorphine" or hydrocodon* or Hydrocon or Codinovo or Dicodid or Dihydrocodeinone or Hycodan or Hycon or Hydrocodeinonebitartrate or Robidone or coactified or ratio-codeine or tylenol or Oxycodone or Dihydrohydroxycodeinone or Dihydrone or Dinarkon or Eucodal or Oxiconum or Oxycodeinon or Oxycone or Oxycontin* or Pancodine or Theocodin).tw. (109967)

240 (Hydromorphon* or Dihydromorphinone or Dilaudid or Laudacon or Palladone).tw. (3104)

241 exp Phencyclidine/ (11364)

242 (Phencyclidine or Sernyl or Serylan).tw. (10253)

243 (Buprenorphine or Buprenex or Buprex or Prefin or Subutex or Temgesic).tw. (10487)

244 exp Fentanyl/ (54296)

245 (Fentanyl or Duragesic or Durogesic or Fentanest or Fentora or Phentanyl or Sublimaze).tw. (32210)

246 exp Pentazocine/ (10799)

247 (Pentazocine or Fortral or Lexir or Talwin).tw. (6612)

248 exp Tramadol/ (12577)

249 (Tramadol* or Adolonta or Amadol or Biodalgic or Biokanol or Contramal or Jutadol or Nobligan or Prontofort or Ralivia or Takadol or Theradol or Tiral or Topalgic or Tradol or Tradonal or Tralgiol or Tramacet or Tramabeta or Tramadin or Tramadoc or Tramadura or Tramagetic or Tramagit or Tramake or Tramal or Tramex or Tramundin or Trasedal or Tridural or Ultram or Zamudol or Zumalgic or Zydol or Zytram).tw. (7402)

250 or/219-249 (681460)

251 250 and (addict* or abuse* or abusing or abusive or misuse* or mis-use* or misusing or mis-using or illicit* or illegal* or unlawful* or unsanction* or non-medical*).tw. (88298)

252 218 or 251 (655243)

253 (designer drug$1 or street drug$1 or recreational drug$1 or narcotic* or non-therapeutic drug$1 or non-medical drug$1 or club drug$1 or rave drug$1 or party pill$1).tw. (38125)

254 exp Cannabis/ (31463)

255 (Cannabi* or marijuana or marihuana or hemp or hash or hashish or ganja).tw. (67171)

256 exp Heroin/ (24255)

257 (Heroin or Diacetylmorphine or Diagesil or Diamorphine or Diamorf or speed).tw. (247697)

258 exp cocaine/ (73086)

259 (cocaine or crack).tw. (81671)

260 exp hallucinogenic drugs/ (23375)

261 (hallucinogen* or psychedelic*).tw. (8568)

262 exp methylenedioxymethamphetamine/ (10424)

263 exp mescaline/ or exp ketamine/ (36299)

264 (methylenedioxymethamphetamine or MDMA or ecstasy or methamphetamine or "3,4-methylenedioxymethamphetamine" or crystal meth or crank or mescaline or mezcalin or peyote or trimethoxyphenethylamine or ketamine or Calipsol or Calypsol or "CI-581" or Kalipsol or Ketalar or Ketanest or Ketaset or "special k" or mushroom* or prilocybin or "gamma hydroxybutrate" or GHB or PCP or angel dust).tw. (100186)

265 ((sniff* or inhal* or snort*) adj3 (solvent* or glue or drug or drugs)).tw. (5295)

266 ("inhalant use" or "inhalant usage" or "inhalant abuse").tw. (951)

267 exp Lysergic Acid Diethylamide/ (14855)

268 (LSD or Lysergide or Lysergic Acid Diethylamide).tw. (12294)

269 ("drug use" or "drug usage" or (drug adj user*)).tw. (103447)

270 or/253-269 (648721)

271 252 or 270 (1157219)

272 exp brief psychotherapy/ (6979)

273 exp Crisis Intervention/ (16222)

274 272 or 273 (23013)

275 exp motivation/ or exp motivation training/ (228911)

276 exp psychotherapy/ (474799)

277 ((cogniti* or behavior* or behaviour* or motivat* or psychosocial* or psycho-social* or psychological) adj3 (therapy or therapies or therapeutic or interven* or interview* or session* or modify or modifies or modified or modification or chang* or conditioning)).tw. (280182)

278 or/275-277 (888533)

279 (brief* or short* or short-rang* or short-term or abbreviate* or concise or early or limited or time-limited or crisis or crises or emergency or emergencies or urgent or immediate* or minimal* or minimum or quick*).tw. (6768481)

280 278 and 279 (167186)

281 (((psycho-therap* or psychotherap* or counsel* or solution-focus?ed or interven* or prevent* or advice or advis*) adj5 (brief* or short* or short-rang* or short-term or abbreviate* or concise or early or limited or time-limited or crisis or crises or emergency or emergencies or urgent or immediate* or minimal* or minimum or quick*)) or contingency management).tw. (186202)

282 280 or 281 (324382)

283 274 or 282 (334216)

284 exp professional referral/ (2878)

285 (refer or refer?ing or refer?ed or refers or referral*).tw. (519480)

286 (gatekeep* or gate-keep*).tw. (7328)

287 (specialist* or specialt* or professional*).tw. (665772)

288 or/284-287 (1149894)

289 271 and (283 or 288) (64106)

290 213 or 289 (65807)

291 limit 290 to "reviews (maximizes specificity)" (1096)

292 limit 290 to ("0830 systematic review" or 1200 meta analysis) [Limit not valid in Embase,Ovid MEDLINE(R),Ovid MEDLINE(R) In-Process; records were retained] (47817)

293 exp meta analysis/ (98098)

294 (meta-analy* or metanaly* or metaanaly* or met analy* or integrative research or integrative review* or integrative overview* or research integration or research overview* or collaborative review*).tw. (115383)

295 (systematic review* or systematic overview* or evidence-based review* or evidence-based overview* or (evidence adj3 (review* or overview*)) or meta-review* or meta-overview* or "review of reviews" or technology assessment* or HTA or HTAs).tw. (143440)

296 or/293-295 (268591)

297 290 and 296 (1297)

298 291 or 292 or 297 (48283)

299 limit 298 to human (40520)

300 limit 299 to yr="2010 -Current" (6725)

301 300 use psyc6 (50)

302 300 use psyc7 (68)

303 110 or 210 or 301 or 302 (418)

304 remove duplicates from 303 (313)

305 304 use prmz (161) **MEDLINE records**

306 304 use emczd (74) **Embase records**

307 304 use psyc6 (34) **PsycINFO records**

308 304 use psyc7 (44) **PsycINFO records**

The Cochrane Library

#1 (sbirt or sbi or sbis):ti,ab,kw (83)

#2 MeSH descriptor Substance-Related Disorders, this term only (2037)

#3 MeSH descriptor Amphetamine-Related Disorders explode all trees (114)

#4 MeSH descriptor Cocaine-Related Disorders explode all trees (501)

#5 MeSH descriptor Marijuana Abuse explode all trees (174)

#6 MeSH descriptor Marijuana Smoking explode all trees (141)

#7 MeSH descriptor Opioid-Related Disorders explode all trees (1042)

#8 MeSH descriptor Phencyclidine Abuse explode all trees (5)

#9 MeSH descriptor Psychoses, Substance-Induced explode all trees (166)

#10 MeSH descriptor Substance Abuse, Intravenous explode all trees (302)

#11 ((substance-related or substance-induced) NEAR/3 (disorder* or psychosis or psychoses)):ti,ab,kw (2227)

#12 ((drug or drugs or substance* or opioid* or opiate* or amphetamine* or amfetamine* or methamphetamine* or methamfetamine or benzodiazepine* or morphine* or methadone* or prescription* or phencyclidine* or solvent* or barbiturate* or depressant* or stimulant* or psychotherap* or psycho-therap* or steroid*) NEAR/3 (addict* or abuse* or abusing or abusive or misuse* or mis-use* or misusing or mis-using or non-medical use* or non-medical usage* or illicit* or illegal* or unlawful* or unsanction* or habit* or dependen* or disorder or disorders or relapse* or consumption or diversion*)):ti,ab,kw (23668)

#13 (#2 OR #3 OR #4 OR #5 OR #6 OR #7 OR #8 OR #9 OR #10 OR #11 OR #12) (24108)

#14 MeSH descriptor Dextropropoxyphene explode all trees (178)

#15 (Dextropropoxyphene or D-Propoxyphene or Propoxyphene or Darvon or Vicodin):ti,ab,kw (348)

#16 (Dextropropoxyphene or D-Propoxyphene or Propoxyphene or Darvon or Vicodin):ti,ab,kw (348)

#17 MeSH descriptor Methadone explode all trees (816)

#18 (Methadone or Amidone or Biodone or Dolophine or Metadol or Metasedin or Methaddict or Methadose or Methex or Phenadone or Phymet or Physeptone or Pinadone or Symoron):ti,ab,kw (1506)

#19 MeSH descriptor Meperidine explode all trees (1000)

#20 (Meperidine or Demerol or Dolantin or Dolargan or Dolcontral or Dolin or Dolosal or Dolsin or Isonipecain or Lidol or Lydol or Operidine or Pethidine or Promedol or Dimethylmeperidine or Isopromedol or Trimeperidine or Lomotil or Reasec):ti,ab,kw (2037)

#21 MeSH descriptor Pentobarbital explode all trees (132)

#22 (Pentobarbital or Diabutal or Etaminal or Ethaminal or Mebubarbital or Mebumal or Nembutal or Pentobarbitone or Sagatal):ti,ab,kw (232)

#23 MeSH descriptor Diazepam explode all trees (1830)

#24 (Diazepam or Apaurin or Diazemuls or Faustan or Relanium or Seduxen or Sibazon or Stesolid or Valium or Nordazepam or Calmday or Dealkylprazepam or Demethyldiazepam or

Deoxydemoxepam or Desmethyldiazepam or Nordaz or Nordiazepam or Norprazepam or "Tranxilium N" or Vegesan):ti,ab,kw (3476)

#25 ("substance use" or "substance usage"):ti,ab,kw (971)

#26 MeSH descriptor Alprazolam explode all trees (427)

#27 (Alprazolam or Alprazolan or Alprox or "Apo-Alpraz" or Cassadan or Esparon or Kalma or "Novo-Alprazol" or "Nu-Alpraz" or Ralozam or Tafil or Trankimazin or Xanax):ti,ab,kw (782)

#28 MeSH descriptor Amphetamine explode all trees (625)

#29 (Amphetamine or Amfetamine or Centramina or Desoxynorephedrin or Fenamine or "l-Amphetamine" or levo-Amphetamine or Levoamphetamine or Mydrial or Phenamine or Phenopromin or Thyramine or Dextroamphetamine or dextro-Amphetamine or Curban or "d-Amphetamine" or Dexamfetamine or Dexamphetamine or Dexedrine or dextro-Amphetamine or DextroStat or Oxydess or dextramethorphan or dextroamethorphan or DXM or methamphetamine or methamfetamine or Deoxyephedrine or Desoxyephedrine or Desoxyn or Madrine):ti,ab,kw (1491)

#30 MeSH descriptor Methylphenidate explode all trees (936)

#31 (Methylphenidate or Centedrin or Daytrana or Dexmethylphenidate or Equasym or Focalin or Metadate or Methylin or Phenidylate or Ritalin* or Tsentedrin or Adderall or Obetrol):ti,ab,kw (1368)

#32 MeSH descriptor Narcotics explode all trees (667)

#33 Narcotic*:ti,ab,kw (3988)

#34 MeSH descriptor Analgesics, Opioid explode all trees (4525)

#35 (opioid* or opiate* or opium):ti,ab,kw (9896)

#36 MeSH descriptor Morphine explode all trees (4395)

#37 (morphine* or morphia or "MS Contin" or Oramorph or codeine or Ardinex or Isocodeine or "N-Methylmorphine" or hydrocodon* or Hydrocon or Codinovo or Dicodid or Dihydrocodeinone or Hycodan or Hycon or Hydrocodeinonebitartrate or Robidone or coactified or ratio-codeine or tylenol or Oxycodone or Dihydrohydroxycodeinone or Dihydrone or Dinarkon or Eucodal or Oxiconum or Oxycodeinon or Oxycone or Oxycontin* or Pancodine or Theocodin):ti,ab,kw (7370)

#38 MeSH descriptor Hydromorphone explode all trees (154)

#39 (Hydromorphon* or Dihydromorphinone or Dilaudid or Laudacon or Palladone):ti,ab,kw (258)

#40 MeSH descriptor Phencyclidine explode all trees (9)

#41 (Phencyclidine or Sernyl or Serylan):ti,ab,kw (41)

#42 MeSH descriptor Buprenorphine explode all trees (560)

#43 (Buprenorphine or Buprenex or Buprex or Prefin or Subutex or Temgesic):ti,ab,kw (1053)

#44 MeSH descriptor Fentanyl explode all trees (3683)

#45 (Fentanyl or Duragesic or Durogesic or Fentanest or Fentora or Phentanyl or Sublimaze):ti,ab,kw (6388)

#46 MeSH descriptor Pentazocine explode all trees (269)

#47 (Pentazocine or Fortral or Lexir or Talwin):ti,ab,kw (523)

#48 MeSH descriptor Tramadol explode all trees (614)

#49 (Tramadol* or Adolonta or Amadol or Biodalgic or Biokanol or Contramal or Jutadol or Nobligan or Prontofort or Ralivia or Takadol or Theradol or Tiral or Topalgic or Tradol or Tradonal or Tralgiol or Tramacet or Tramabeta or Tramadin or Tramadoc or Tramadura or Tramagetic or Tramagit or Tramake or Tramal or Tramex or Tramundin or Trasedal or Tridural or Ultram or Zamudol or Zumalgic or Zydol or Zytram):ti,ab,kw (1259)

#50 (#14 OR #15 OR #16 OR #17 OR #18 OR #19 OR #20 OR #21 OR #22 OR #23 OR #24 OR #25 OR #26 OR #27 OR #28 OR #29 OR #30 OR #31 OR #32 OR #33 OR #34 OR #35 OR #36 OR #37 OR #38 OR #39 OR #40 OR #41 OR #42 OR #43 OR #44 OR #45 OR #46 OR #47 OR #48 OR #49) (30145)

#51 (addict* or abuse* or abusing or abusive or misuse* or mis-use* or misusing or mis-using or illicit* or illegal* or unlawful* or unsanction* or non-medical*):ti,ab,kw (6894)

#52 (#50 AND #51) (2295)

#53 MeSH descriptor Designer Drugs explode all trees (4)

#54 MeSH descriptor Street Drugs explode all trees (191)

#55 ((designer NEXT drug*) or (street NEXT drug*) or (recreational NEXT drug*) or narcotic* or (non-therapeutic NEXT drug*) or (non-medical NEXT drug*) or (club NEXT drug*) or (rave NEXT drug*) or (party NEXT pill*)):ti,ab,kw (4144)

#56 MeSH descriptor Cannabis explode all trees (240)

#57 (Cannabi* or marijuana or marihuana or hemp or hash or hashish or ganja):ti,ab,kw (1302)

#58 MeSH descriptor Heroin explode all trees (235)

#59 (Heroin or Diacetylmorphine or Diagesil or Diamorphine or Diamorf or speed):ti,ab,kw (6196)

#60 MeSH descriptor Cocaine explode all trees (572)

#61 (cocaine or crack):ti,ab,kw (1925)

#62 MeSH descriptor Hallucinogens explode all trees (118)

#63 (hallucinogen* or psychedelic*):ti,ab,kw (181)

#64 MeSH descriptor N-Methyl-3,4-methylenedioxyamphetamine explode all trees (83)

#65 MeSH descriptor Mescaline explode all trees (7)

#66 MeSH descriptor Ketamine explode all trees (898)

#67 (methylenedioxymethamphetamine or MDMA or ecstasy or methamphetamine or "3,4-methylenedioxymethamphetamine" or "crystal meth" or crank or mescaline or mezcalin or peyote or trimethoxyphenethylamine or ketamine or Calipsol or Calypsol or "CI-581" or Kalipsol or Ketalar or Ketanest or Ketaset or "special k" or mushroom* or prilocybin or "gamma hydroxybutrate" or GHB or PCP or "angel dust"):ti,ab,kw (2692)

#68 ((sniff* or inhal* or snort*) adj3 (solvent* or glue or drug or drugs)):ti,ab,kw (0)

#69 ("inhalant use" or "inhalant usage" or "inhalant abuse"):ti,ab,kw (9)

#70 MeSH descriptor Lysergic Acid Diethylamide explode all trees (48)

#71 (LSD or Lysergide or "Lysergic Acid Diethylamide"):ti,ab,kw (168)

#72 ("drug use" or "drug usage" or (drug NEXT user*)):ti,ab,kw (2269)

#73 (#53 OR #54 OR #55 OR #56 OR #57 OR #58 OR #59 OR #60 OR #61 OR #62 OR #63 OR #64 OR #65 OR #66 OR #67 OR #68 OR #69 OR #70 OR #71 OR #72) (17004)

#74 (#13 OR #52 OR #73) (37164)

#75 MeSH descriptor Psychotherapy, Brief explode all trees (562)

#76 MeSH descriptor Crisis Intervention explode all trees (127)

#77 (#75 OR #75) (562)

#78 MeSH descriptor Motivation explode all trees (4063)

#79 MeSH descriptor Psychotherapy explode all trees (12750)

#80 ((cogniti* or behavior* or behaviour* or motivat* or psychosocial* or psycho-social* or psychological) NEAR/3 (therapy or therapies or therapeutic or interven* or interview* or session* or modify or modifies or modified or modification or chang* or conditioning)):ti,ab,kw (20660)

#81 (#78 OR #79 OR #80) (27900)

#82 (brief* or short* or short-rang* or short-term or abbreviate* or concise or early or limited or time-limited or crisis or crises or emergency or emergencies or urgent or immediate* or minimal* or minimum or quick*):ti,ab,kw (149482)

#83 (#81 AND #82) (8054)

#84 (((psycho-therap* or psychotherap* or counsel* or (solution* NEAR/2 focus*) or interven* or prevent* or advice or advis*) NEAR/5 (brief* or short* or short-rang* or short-term or abbreviate* or concise or early or limited or time-limited or crisis or crises or emergency or emergencies or urgent or immediate* or minimal* or minimum or quick*)) or "contingency management"):ti,ab,kw (8811)

#85 (#83 OR #84) (14381)

#86 (#77 OR #85) (14381)

#87 MeSH descriptor Referral and Consultation explode all trees (1490)

#88 (refer or refering or referring or refered or referred or refers or referral*):ti,ab,kw (8187)

#89 (gatekeep* or gate-keep*):ti,ab,kw (66)

#90 (specialist* or specialt* or professional*):ti,ab,kw (7689)

#91 (#87 OR #88 OR #89 OR 90) (55599)

#92 (#74 AND (#86 OR #91)) (5122)

#93 (#1 OR #92) (5201)

CENTRAL only – 4500 records

Reviews (DSR, DARE, HTA; yr 2010-2012) – 288 records

Project Cork Database

Abstract: (clinical trial* OR random* OR RCT* OR placebo* OR blind* OR dumm* OR mask*)

AND

Keywords: (brief or short or concise or time-limited or minimal* or minimum or quick* or immediate* or minimal* or minimum or quick) AND (drug OR substance) AND (drug misuse OR abuse OR misuse)

167 results

CINAHL (Ebsco platform) – RCTs

#	Query	Limiters/Expanders	Results
S101	S99 NOT S100	Expanders - Apply related words<br>Search modes - Boolean/Phrase	**3158**
S100	S95 or S96	Limiters - Publication Type: Review, Systematic Review	320

		Expanders - Apply related words<br>Search modes - Boolean/Phrase	
S99	S97 NOT S98	Expanders - Apply related words<br>Search modes - Boolean/Phrase	3477
S98	S95 or S96	Limiters - Publication Type: Anecdote, Commentary, Editorial, Interview, Letter<br>Expanders - Apply related words<br>Search modes - Boolean/Phrase	166
S97	S95 or S96	Expanders - Apply related words<br>Search modes - Boolean/Phrase	3643
S96	S1 OR S87 OR S88	Limiters - Publication Type: Randomized Controlled Trial<br>Expanders - Apply related words<br>Search modes - Boolean/Phrase	94
S95	S89 and S94	Expanders - Apply related words<br>Search modes - Boolean/Phrase	3643
S94	S90 or S91 or S92 or S93	Expanders - Apply related words<br>Search modes - Boolean/Phrase	606822
S93	TX "controlled clinical trial" OR TX (random* or RCT OR RCTs or placebo*) OR TX (((singl* or doubl* or trebl*	Expanders - Apply related words	606822

	or tripl*) N3 (mask* or blind* or dumm*)))	Search modes - Boolean/Phrase	
S92	(MH "Randomized Controlled Trials") OR (MH "Triple-Blind Studies") OR (MH "Double-Blind Studies") OR (MH "Single-Blind Studies")	Expanders - Apply related words Search modes - Boolean/Phrase	29632
S91	(MH "Randomized Controlled Trials") OR (MH "Triple-Blind Studies") OR (MH "Double-Blind Studies") OR (MH "Single-Blind Studies")	Expanders - Apply related words Search modes - Boolean/Phrase	29632
S90	(MH "Randomized Controlled Trials") OR (MH "Triple-Blind Studies") OR (MH "Double-Blind Studies")	Expanders - Apply related words Search modes - Boolean/Phrase	25149
S89	S1 OR S87 OR S88	Expanders - Apply related words Search modes - Boolean/Phrase	12144
S88	S68 and S86	Expanders - Apply related words Search modes - Boolean/Phrase	9415
S87	S68 and S81	Expanders - Apply related words Search modes - Boolean/Phrase	3454
S86	S82 or S83 or S84 or S85	Expanders - Apply related words Search modes - Boolean/Phrase	282590
S85	TX specialist* or specialt* or professional*	Expanders - Apply related words Search modes - Boolean/Phrase	249922
S84	TX gatekeep* or gate-keep*	Expanders - Apply related words	868

		Search modes - Boolean/Phrase	
S83	TX refer or refering or referring or refered or referred or refers or referral*	Expanders - Apply related words Search modes - Boolean/Phrase	41773
S82	(MH "Referral and Consultation+")	Expanders - Apply related words Search modes - Boolean/Phrase	14620
S81	S71 or S80	Expanders - Apply related words Search modes - Boolean/Phrase	40907
S80	S77 or S78 or S79	Expanders - Apply related words Search modes - Boolean/Phrase	40907
S79	TX "contingency management"	Expanders - Apply related words Search modes - Boolean/Phrase	221
S78	TX (psycho-therap* or psychotherap* or counsel* or solution-focus* or interven* or prevent* or advice or advis*) N5 (brief* or short* or short-rang* or short-term or abbreviate* or concise or early or limited or time-limited or crisis or crises or emergency or emergencies or urgent or immediate* or minimal* or minimum or quick*)	Expanders - Apply related words Search modes - Boolean/Phrase	25659
S77	S75 and S76	Expanders - Apply related words Search modes - Boolean/Phrase	20645
S76	brief* or short* or short-rang* or short-term or abbreviate* or concise or early or limited or time-limited or crisis or crises or emergency or emergencies or urgent or immediate* or minimal* or minimum or	Expanders - Apply related words Search modes - Boolean/Phrase	329597

	quick*		
S75	S72 or S73 or S74	Expanders - Apply related words<br>Search modes - Boolean/Phrase	124138
S74	TX ((cogniti* or behavior* or behaviour* or motivat* or psychosocial* or psycho-social* or psychological) N3 (therapy or therapies or therapeutic or interven* or interview* or session* or modify or modifies or modified or modification or chang* or conditioning))	Expanders - Apply related words<br>Search modes - Boolean/Phrase	37108
S73	(MH "Psychotherapy+")	Expanders - Apply related words<br>Search modes - Boolean/Phrase	72956
S72	(MH "Motivation+")	Expanders - Apply related words<br>Search modes - Boolean/Phrase	32900
S71	S69 or S70	Expanders - Apply related words<br>Search modes - Boolean/Phrase	2262
S70	(MH "Crisis Intervention")	Expanders - Apply related words<br>Search modes - Boolean/Phrase	1978
S69	(MH "Psychotherapy, Brief")	Expanders - Apply related words<br>Search modes - Boolean/Phrase	284
S68	S9 or S46 or S67	Expanders - Apply related words<br>Search modes - Boolean/Phrase	77698
S67	S47 or S48 or S49 or S50 or S51 or S52 or S53 or S54 or S55 or S56 or S57 or S58 or S59 or S60 or S61 or S62 or	Expanders - Apply related words	38984

	S63 or S64 or S65 or S66	Search modes - Boolean/Phrase	
S66	TX "drug use" or "drug usage" or (drug W1 user*)	Expanders - Apply related words Search modes - Boolean/Phrase	10192
S65	TX LSD or Lysergide or Lysergic Acid Diethylamide	Expanders - Apply related words Search modes - Boolean/Phrase	241
S64	(MH "Lysergic Acid Diethylamide")	Expanders - Apply related words Search modes - Boolean/Phrase	79
S63	TX "inhalant use" or "inhalant usage" or "inhalant abuse"	Expanders - Apply related words Search modes - Boolean/Phrase	285
S62	TX ((sniff* or inhal* or snort*) N3 (solvent* or glue or drug or drugs))	Expanders - Apply related words Search modes - Boolean/Phrase	367
S61	TX methylenedioxymethamphetamine or MDMA or ecstasy or methamphetamine or "3,4-methylenedioxymethamphetamine" or crystal meth or crank or mescaline or mezcalin or peyote or trimethoxyphenethylamine or ketamine or Calipsol or Calypsol or "CI-581" or Kalipsol or Ketalar or Ketanest or Ketaset or "special k" or mushroom* or prilocybin or "gamma hydroxybutrate" or GHB or PCP or "angel dust"	Expanders - Apply related words Search modes - Boolean/Phrase	4374
S60	(MH "Ketamine")	Expanders - Apply related words Search modes - Boolean/Phrase	901
S59	(MH "Mescaline")	Expanders - Apply related words	10

		Search modes - Boolean/Phrase	
S58	(MH "Methylenedioxymethamphetamine")	Expanders - Apply related words Search modes - Boolean/Phrase	504
S57	TX hallucinogen* or psychedelic*	Expanders - Apply related words Search modes - Boolean/Phrase	413
S56	(MH "Hallucinogens+")	Expanders - Apply related words Search modes - Boolean/Phrase	759
S55	TX cocaine or crack	Expanders - Apply related words Search modes - Boolean/Phrase	4581
S54	(MH "Cocaine+")	Expanders - Apply related words Search modes - Boolean/Phrase	2413
S53	TX Heroin or Diacetylmorphine or Diagesil or Diamorphine or Diamorf or speed	Expanders - Apply related words Search modes - Boolean/Phrase	12497
S52	(MH "Heroin")	Expanders - Apply related words Search modes - Boolean/Phrase	1240
S51	TX Cannabi* or marijuana or marihuana or hemp or hash or hashish or ganja	Expanders - Apply related words Search modes - Boolean/Phrase	4727
S50	(MH "Cannabis")	Expanders - Apply related words	2736

		Search modes - Boolean/Phrase	
S49	TX designer drug* or street drug* or recreational drug* or narcotic* or non-therapeutic drug* or non-medical drug* or club drug* or rave drug* or party pill*	Expanders - Apply related words Search modes - Boolean/Phrase	9593
S48	(MH "Street Drugs+")	Expanders - Apply related words Search modes - Boolean/Phrase	2177
S47	(MH "Designer Drugs")	Expanders - Apply related words Search modes - Boolean/Phrase	98
S46	(S10 or S11 or S12 or S13 or S14 or S15 or S16 or S17 or S18 or S19 or S20 or S21 or S22 or S23 or S24 or S25 or S26 or S27 or S28 or S29 or S30 or S31 or S32 or S33 or S34 or S35 or S36 or S37 or S38 or S39 or S40 or S41 or S42 or S43 or S44) AND TX (addict* or abuse* or abusing or abusive or misuse* or mis-use* or misusing or mis-using or illicit* or illegal* or unlawful* or unsanction* or non-medical*)	Expanders - Apply related words Search modes - Boolean/Phrase	15623
S45	S10 or S11 or S12 or S13 or S14 or S15 or S16 or S17 or S18 or S19 or S20 or S21 or S22 or S23 or S24 or S25 or S26 or S27 or S28 or S29 or S30 or S31 or S32 or S33 or S34 or S35 or S36 or S37 or S38 or S39 or S40 or S41 or S42 or S43 or S44	Expanders - Apply related words Search modes - Boolean/Phrase	36684
S44	TX Tramadol* or Adolonta or Amadol or Biodalgic or Biokanol or Contramal or Jutadol or Nobligan or Prontofort or Ralivia or Takadol or Theradol or Tiral or Topalgic or Tradol or Tradonal or Tralgiol or Tramacet or Tramabeta or Tramadin or Tramadoc or Tramadura or Tramagetic or Tramagit or Tramake or Tramal or Tramex or Tramundin or Trasedal or Tridural or Ultram or Zamudol or Zumalgic or Zydol or Zytram	Expanders - Apply related words Search modes - Boolean/Phrase	494
S43	(MH "Tramadol")	Expanders - Apply related words	349

		Search modes - Boolean/Phrase	
S42	TX Pentazocine or Fortral or Lexir or Talwin	Expanders - Apply related words Search modes - Boolean/Phrase	58
S41	(MH "Pentazocine")	Expanders - Apply related words Search modes - Boolean/Phrase	25
S40	TX Fentanyl or Duragesic or Durogesic or Fentanest or Fentora or Phentanyl or Sublimaze	Expanders - Apply related words Search modes - Boolean/Phrase	2038
S39	(MH "Fentanyl+")	Expanders - Apply related words Search modes - Boolean/Phrase	1758
S38	TX Buprenorphine or Buprenex or Buprex or Prefin or Subutex or Temgesic	Expanders - Apply related words Search modes - Boolean/Phrase	914
S37	Buprenorphine	Expanders - Apply related words Search modes - Boolean/Phrase	877
S36	TX Phencyclidine or Sernyl or Serylan	Expanders - Apply related words Search modes - Boolean/Phrase	129
S35	(MH "Phencyclidine")	Expanders - Apply related words Search modes - Boolean/Phrase	72
S34	TX Hydromorphon* or Dihydromorphinone or Dilaudid or Laudacon or Palladone	Expanders - Apply related words	316

		Search modes - Boolean/Phrase	
S33	(MH "Dihydromorphinone")	Expanders - Apply related words Search modes - Boolean/Phrase	190
S32	TX morphine* or morphia or "MS Contin" or Oramorph or codeine or Ardinex or Isocodeine or "N-Methylmorphine" or hydrocodon* or Hydrocon or Codinovo or Dicodid or Dihydrocodeinone or Hycodan or Hycon or Hydrocodeinonebitartrate or Robidone or coactified or ratio-codeine or tylenol or Oxycodone or Dihydrohydroxycodeinone or Dihydrone or Dinarkon or Eucodal or Oxiconum or Oxycodeinon or Oxycone or Oxycontin* or Pancodine or Theocodin	Expanders - Apply related words Search modes - Boolean/Phrase	4856
S31	(MH "Morphine+")	Expanders - Apply related words Search modes - Boolean/Phrase	5819
S30	TX opioid* or opiate* or opium	Expanders - Apply related words Search modes - Boolean/Phrase	9800
S29	(MH "Analgesics, Opioid")	Expanders - Apply related words Search modes - Boolean/Phrase	3894
S28	TX Narcotic*	Expanders - Apply related words Search modes - Boolean/Phrase	7314
S27	(MH "Narcotics")	Expanders - Apply related words Search modes - Boolean/Phrase	4731
S26	TX Methylphenidate or Centedrin or Daytrana or Dexmethylphenidate or Equasym or Focalin or	Expanders - Apply related words	1206

	Metadate or Methylin or Phenidylate or Ritalin* or Tsentedrin or Adderall or Obetrol	Search modes - Boolean/Phrase	
S25	(MH "Methylphenidate")	Expanders - Apply related words Search modes - Boolean/Phrase	952
S24	TX Amphetamine or Amfetamine or Centramina or Desoxynorephedrin or Fenamine or "l-Amphetamine" or levo-Amphetamine or Levoamphetamine or Mydrial or Phenamine or Phenopromin or Thyramine or Dextroamphetamine or dextro-Amphetamine or Curban or "d-Amphetamine" or Dexamfetamine or Dexamphetamine or Dexedrine or dextro-Amphetamine or DextroStat or Oxydess or dextramethorphan or dextroamethorphan or DXM or methamphetamine or methamfetamine or Deoxyephedrine or Desoxyephedrine or Desoxyn or Madrine	Expanders - Apply related words Search modes - Boolean/Phrase	2348
S23	(MH "Amphetamine+")	Expanders - Apply related words Search modes - Boolean/Phrase	194
S22	TX Alprazolam or Alprazolan or Alprox or "Apo-Alpraz" or Cassadan or Esparon or Kalma or "Novo-Alprazol" or "Nu-Alpraz" or Ralozam or Tafil or Trankimazin or Xanax	Expanders - Apply related words Search modes - Boolean/Phrase	173
S21	(MH "Alprazolam")	Expanders - Apply related words Search modes - Boolean/Phrase	84
S20	TX "substance use" or "substance usage"	Expanders - Apply related words Search modes - Boolean/Phrase	14107
S19	TX Diazepam or Apaurin or Diazemuls or Faustan or Relanium or Seduxen or Sibazon or Stesolid or Valium or Nordazepam or Calmday or Dealkylprazepam or	Expanders - Apply related words Search modes -	730

	Demethyldiazepam or Deoxydemoxepam or Desmethyldiazepam or Nordaz or Nordiazepam or Norprazepam or "Tranxilium N" or Vegesan	Boolean/Phrase	
S18	(MH "Diazepam")	Expanders - Apply related words Search modes - Boolean/Phrase	448
S17	TX Pentobarbital or Diabutal or Etaminal or Ethaminal or Mebubarbital or Mebumal or Nembutal or Pentobarbitone or Sagatal	Expanders - Apply related words Search modes - Boolean/Phrase	274
S16	(MH "Pentobarbital")	Expanders - Apply related words Search modes - Boolean/Phrase	108
S15	TX Meperidine or Demerol or Dolantin or Dolargan or Dolcontral or Dolin or Dolosal or Dolsin or Isonipecain or Lidol or Lydol or Operidine or Pethidine or Promedol or Dimethylmeperidine or Isopromedol or Trimeperidine or Lomotil or Reasec	Expanders - Apply related words Search modes - Boolean/Phrase	736
S14	(MH "Meperidine")	Expanders - Apply related words Search modes - Boolean/Phrase	466
S13	TX Methadone or Amidone or Biodone or Dolophine or Metadol or Metasedin or Methaddict or Methadose or Methex or Phenadone or Phymet or Physeptone or Pinadone or Symoron	Expanders - Apply related words Search modes - Boolean/Phrase	2470
S12	(MH "Methadone")	Expanders - Apply related words Search modes - Boolean/Phrase	1856
S11	TX Dextropropoxyphene or D-Propoxyphene or Propoxyphene or Darvon or Vicodin	Expanders - Apply related words Search modes - Boolean/Phrase	198

S10	(MH "Propoxyphene")	Expanders - Apply related words<br>Search modes - Boolean/Phrase	97
S9	S2 or S3 or S4 or S5 or S6 or S7 or S8	Expanders - Apply related words<br>Search modes - Boolean/Phrase	53353
S8	TX ((drug or drugs or substance* or opioid* or opiate* or amphetamine* or amfetamine* or methamphetamine* or methamfetamine or benzodiazepine* or morphine* or methadone* or prescription* or phencyclidine* or solvent* or barbiturate* or depressant* or stimulant* or psychotherap* or psycho-therap* or steroid*) N3 (addict* or abuse* or abusing or abusive or misuse* or mis-use* or misusing or mis-using or non-medical use* or non-medical usage* or illicit* or illegal* or unlawful* or unsanction* or habit* or dependen* or disorder or disorders or relapse* or consumption or diversion*))	Expanders - Appliquer les mots connexes<br>Search modes - Boolean/Phrase	53169
S7	TX ((substance-related or substance-induced) N3 (disorder* or psychosis or psychoses))	Expanders - Appliquer les mots connexes<br>Search modes - Boolean/Phrase	303
S6	(MH "Psychoses, Substance-Induced")	Expanders - Appliquer les mots connexes<br>Search modes - Boolean/Phrase	158
S5	(MH "Inhalant Abuse")	Expanders - Appliquer les mots connexes<br>Search modes - Boolean/Phrase	221
S4	(MH "Substance Abuse, Perinatal")	Expanders - Appliquer les mots connexes<br>Search modes - Boolean/Phrase	1088
S3	(MH "Substance Abuse, Intravenous")	Expanders - Appliquer	2089

		les mots connexes<br>Search modes - Boolean/Phrase	
S2	(MH "Substance Use Disorders")	Expanders - Appliquer les mots connexes<br>Search modes - Boolean/Phrase	8151
S1	TX SBIRT OR SBI OR SBIs	Expanders - Appliquer les mots connexes<br>Search modes - Boolean/Phrase	238

CINAHL (Ebsco platform) – Reviews

#	Query	Limiters/Expanders	Results
S101	S95 or S96	Limiters - Published Date from: 20100101-20121231; Publication Type: Review, Systematic Review<br>Expanders - Apply related words<br>Search modes - Boolean/Phrase	**62**
S100	S95 or S96	Limiters - Publication Type: Review, Systematic Review<br>Expanders - Apply related words<br>Search modes - Boolean/Phrase	320
S99	S97 NOT S98	Expanders - Apply related words<br>Search modes - Boolean/Phrase	3477
S98	S95 or S96	Limiters - Publication	166

		Type: Anecdote, Commentary, Editorial, Interview, Letter<br>Expanders - Apply related words<br>Search modes - Boolean/Phrase	
S97	S95 or S96	Expanders - Apply related words<br>Search modes - Boolean/Phrase	3643
S96	S1 OR S87 OR S88	Limiters - Publication Type: Randomized Controlled Trial<br>Expanders - Apply related words<br>Search modes - Boolean/Phrase	94
S95	S89 and S94	Expanders - Apply related words<br>Search modes - Boolean/Phrase	3643
S94	S90 or S91 or S92 or S93	Expanders - Apply related words<br>Search modes - Boolean/Phrase	606822
S93	TX "controlled clinical trial" OR TX (random* or RCT OR RCTs or placebo*) OR TX (((singl* or doubl* or trebl* or tripl*) N3 (mask* or blind* or dumm*)))	Expanders - Apply related words<br>Search modes - Boolean/Phrase	606822
S92	(MH "Randomized Controlled Trials") OR (MH "Triple-Blind Studies") OR (MH "Double-Blind Studies") OR (MH "Single-Blind Studies")	Expanders - Apply related words<br>Search modes - Boolean/Phrase	29632
S91	(MH "Randomized Controlled Trials") OR (MH "Triple-Blind Studies") OR (MH "Double-Blind Studies") OR (MH "Single-Blind Studies")	Expanders - Apply related words<br>Search modes -	29632

		Boolean/Phrase	
S90	(MH "Randomized Controlled Trials") OR (MH "Triple-Blind Studies") OR (MH "Double-Blind Studies")	Expanders - Apply related words Search modes - Boolean/Phrase	25149
S89	S1 OR S87 OR S88	Expanders - Apply related words Search modes - Boolean/Phrase	12144
S88	S68 and S86	Expanders - Apply related words Search modes - Boolean/Phrase	9415
S87	S68 and S81	Expanders - Apply related words Search modes - Boolean/Phrase	3454
S86	S82 or S83 or S84 or S85	Expanders - Apply related words Search modes - Boolean/Phrase	282590
S85	TX specialist* or specialt* or professional*	Expanders - Apply related words Search modes - Boolean/Phrase	249922
S84	TX gatekeep* or gate-keep*	Expanders - Apply related words Search modes - Boolean/Phrase	868
S83	TX refer or refering or referring or refered or referred or refers or referral*	Expanders - Apply related words Search modes - Boolean/Phrase	41773
S82	(MH "Referral and Consultation+")	Expanders - Apply related words Search modes -	14620

		Boolean/Phrase	
S81	S71 or S80	Expanders - Apply related words Search modes - Boolean/Phrase	40907
S80	S77 or S78 or S79	Expanders - Apply related words Search modes - Boolean/Phrase	40907
S79	TX "contingency management"	Expanders - Apply related words Search modes - Boolean/Phrase	221
S78	TX (psycho-therap* or psychotherap* or counsel* or solution-focus* or interven* or prevent* or advice or advis*) N5 (brief* or short* or short-rang* or short-term or abbreviate* or concise or early or limited or time-limited or crisis or crises or emergency or emergencies or urgent or immediate* or minimal* or minimum or quick*)	Expanders - Apply related words Search modes - Boolean/Phrase	25659
S77	S75 and S76	Expanders - Apply related words Search modes - Boolean/Phrase	20645
S76	brief* or short* or short-rang* or short-term or abbreviate* or concise or early or limited or time-limited or crisis or crises or emergency or emergencies or urgent or immediate* or minimal* or minimum or quick*	Expanders - Apply related words Search modes - Boolean/Phrase	329597
S75	S72 or S73 or S74	Expanders - Apply related words Search modes - Boolean/Phrase	124138
S74	TX ((cogniti* or behavior* or behaviour* or motivat* or psychosocial* or psycho-social* or psychological) N3 (therapy or therapies or therapeutic or interven* or interview* or session* or modify or modifies or	Expanders - Apply related words Search modes - Boolean/Phrase	37108

	modified or modification or chang* or conditioning))		
S73	(MH "Psychotherapy+")	Expanders - Apply related words<br>Search modes - Boolean/Phrase	72956
S72	(MH "Motivation+")	Expanders - Apply related words<br>Search modes - Boolean/Phrase	32900
S71	S69 or S70	Expanders - Apply related words<br>Search modes - Boolean/Phrase	2262
S70	(MH "Crisis Intervention")	Expanders - Apply related words<br>Search modes - Boolean/Phrase	1978
S69	(MH "Psychotherapy, Brief")	Expanders - Apply related words<br>Search modes - Boolean/Phrase	284
S68	S9 or S46 or S67	Expanders - Apply related words<br>Search modes - Boolean/Phrase	77698
S67	S47 or S48 or S49 or S50 or S51 or S52 or S53 or S54 or S55 or S56 or S57 or S58 or S59 or S60 or S61 or S62 or S63 or S64 or S65 or S66	Expanders - Apply related words<br>Search modes - Boolean/Phrase	38984
S66	TX "drug use" or "drug usage" or (drug W1 user*)	Expanders - Apply related words<br>Search modes - Boolean/Phrase	10192
S65	TX LSD or Lysergide or Lysergic Acid Diethylamide	Expanders - Apply related words<br>Search modes -	241

		Boolean/Phrase	
S64	(MH "Lysergic Acid Diethylamide")	Expanders - Apply related words Search modes - Boolean/Phrase	79
S63	TX "inhalant use" or "inhalant usage" or "inhalant abuse"	Expanders - Apply related words Search modes - Boolean/Phrase	285
S62	TX ((sniff* or inhal* or snort*) N3 (solvent* or glue or drug or drugs))	Expanders - Apply related words Search modes - Boolean/Phrase	367
S61	TX methylenedioxymethamphetamine or MDMA or ecstasy or methamphetamine or "3,4-methylenedioxymethamphetamine" or crystal meth or crank or mescaline or mezcalin or peyote or trimethoxyphenethylamine or ketamine or Calipsol or Calypsol or "CI-581" or Kalipsol or Ketalar or Ketanest or Ketaset or "special k" or mushroom* or prilocybin or "gamma hydroxybutrate" or GHB or PCP or "angel dust"	Expanders - Apply related words Search modes - Boolean/Phrase	4374
S60	(MH "Ketamine")	Expanders - Apply related words Search modes - Boolean/Phrase	901
S59	(MH "Mescaline")	Expanders - Apply related words Search modes - Boolean/Phrase	10
S58	(MH "Methylenedioxymethamphetamine")	Expanders - Apply related words Search modes - Boolean/Phrase	504
S57	TX hallucinogen* or psychedelic*	Expanders - Apply related words Search modes -	413

		Boolean/Phrase	
S56	(MH "Hallucinogens+")	Expanders - Apply related words Search modes - Boolean/Phrase	759
S55	TX cocaine or crack	Expanders - Apply related words Search modes - Boolean/Phrase	4581
S54	(MH "Cocaine+")	Expanders - Apply related words Search modes - Boolean/Phrase	2413
S53	TX Heroin or Diacetylmorphine or Diagesil or Diamorphine or Diamorf or speed	Expanders - Apply related words Search modes - Boolean/Phrase	12497
S52	(MH "Heroin")	Expanders - Apply related words Search modes - Boolean/Phrase	1240
S51	TX Cannabi* or marijuana or marihuana or hemp or hash or hashish or ganja	Expanders - Apply related words Search modes - Boolean/Phrase	4727
S50	(MH "Cannabis")	Expanders - Apply related words Search modes - Boolean/Phrase	2736
S49	TX designer drug* or street drug* or recreational drug* or narcotic* or non-therapeutic drug* or non-medical drug* or club drug* or rave drug* or party pill*	Expanders - Apply related words Search modes - Boolean/Phrase	9593
S48	(MH "Street Drugs+")	Expanders - Apply related words Search modes -	2177

		Boolean/Phrase	
S47	(MH "Designer Drugs")	Expanders - Apply related words Search modes - Boolean/Phrase	98
S46	(S10 or S11 or S12 or S13 or S14 or S15 or S16 or S17 or S18 or S19 or S20 or S21 or S22 or S23 or S24 or S25 or S26 or S27 or S28 or S29 or S30 or S31 or S32 or S33 or S34 or S35 or S36 or S37 or S38 or S39 or S40 or S41 or S42 or S43 or S44) AND TX (addict* or abuse* or abusing or abusive or misuse* or mis-use* or misusing or mis-using or illicit* or illegal* or unlawful* or unsanction* or non-medical*)	Expanders - Apply related words Search modes - Boolean/Phrase	15623
S45	S10 or S11 or S12 or S13 or S14 or S15 or S16 or S17 or S18 or S19 or S20 or S21 or S22 or S23 or S24 or S25 or S26 or S27 or S28 or S29 or S30 or S31 or S32 or S33 or S34 or S35 or S36 or S37 or S38 or S39 or S40 or S41 or S42 or S43 or S44	Expanders - Apply related words Search modes - Boolean/Phrase	36684
S44	TX Tramadol* or Adolonta or Amadol or Biodalgic or Biokanol or Contramal or Jutadol or Nobligan or Prontofort or Ralivia or Takadol or Theradol or Tiral or Topalgic or Tradol or Tradonal or Tralgiol or Tramacet or Tramabeta or Tramadin or Tramadoc or Tramadura or Tramagetic or Tramagit or Tramake or Tramal or Tramex or Tramundin or Trasedal or Tridural or Ultram or Zamudol or Zumalgic or Zydol or Zytram	Expanders - Apply related words Search modes - Boolean/Phrase	494
S43	(MH "Tramadol")	Expanders - Apply related words Search modes - Boolean/Phrase	349
S42	TX Pentazocine or Fortral or Lexir or Talwin	Expanders - Apply related words Search modes - Boolean/Phrase	58
S41	(MH "Pentazocine")	Expanders - Apply related words Search modes -	25

		Boolean/Phrase	
S40	TX Fentanyl or Duragesic or Durogesic or Fentanest or Fentora or Phentanyl or Sublimaze	Expanders - Apply related words Search modes - Boolean/Phrase	2038
S39	(MH "Fentanyl+")	Expanders - Apply related words Search modes - Boolean/Phrase	1758
S38	TX Buprenorphine or Buprenex or Buprex or Prefin or Subutex or Temgesic	Expanders - Apply related words Search modes - Boolean/Phrase	914
S37	Buprenorphine	Expanders - Apply related words Search modes - Boolean/Phrase	877
S36	TX Phencyclidine or Sernyl or Serylan	Expanders - Apply related words Search modes - Boolean/Phrase	129
S35	(MH "Phencyclidine")	Expanders - Apply related words Search modes - Boolean/Phrase	72
S34	TX Hydromorphon* or Dihydromorphinone or Dilaudid or Laudacon or Palladone	Expanders - Apply related words Search modes - Boolean/Phrase	316
S33	(MH "Dihydromorphinone")	Expanders - Apply related words Search modes - Boolean/Phrase	190
S32	TX morphine* or morphia or "MS Contin" or Oramorph or codeine or Ardinex or Isocodeine or "N-Methylmorphine" or hydrocodon* or Hydrocon or	Expanders - Apply related words Search modes -	4856

	Codinovo or Dicodid or Dihydrocodeinone or Hycodan or Hycon or Hydrocodeinonebitartrate or Robidone or coactified or ratio-codeine or tylenol or Oxycodone or Dihydrohydroxycodeinone or Dihydrone or Dinarkon or Eucodal or Oxiconum or Oxycodeinon or Oxycone or Oxycontin* or Pancodine or Theocodin	Boolean/Phrase	
S31	(MH "Morphine+")	Expanders - Apply related words Search modes - Boolean/Phrase	5819
S30	TX opioid* or opiate* or opium	Expanders - Apply related words Search modes - Boolean/Phrase	9800
S29	(MH "Analgesics, Opioid")	Expanders - Apply related words Search modes - Boolean/Phrase	3894
S28	TX Narcotic*	Expanders - Apply related words Search modes - Boolean/Phrase	7314
S27	(MH "Narcotics")	Expanders - Apply related words Search modes - Boolean/Phrase	4731
S26	TX Methylphenidate or Centedrin or Daytrana or Dexmethylphenidate or Equasym or Focalin or Metadate or Methylin or Phenidylate or Ritalin* or Tsentedrin or Adderall or Obetrol	Expanders - Apply related words Search modes - Boolean/Phrase	1206
S25	(MH "Methylphenidate")	Expanders - Apply related words Search modes - Boolean/Phrase	952
S24	TX Amphetamine or Amfetamine or Centramina or Desoxynorephedrin or Fenamine or "l-Amphetamine" or levo-Amphetamine or Levoamphetamine or	Expanders - Apply related words Search modes -	2348

	Mydrial or Phenamine or Phenopromin or Thyramine or Dextroamphetamine or dextro-Amphetamine or Curban or "d-Amphetamine" or Dexamfetamine or Dexamphetamine or Dexedrine or dextro-Amphetamine or DextroStat or Oxydess or dextramethorphan or dextroamethorphan or DXM or methamphetamine or methamfetamine or Deoxyephedrine or Desoxyephedrine or Desoxyn or Madrine	Boolean/Phrase	
S23	(MH "Amphetamine+")	Expanders - Apply related words Search modes - Boolean/Phrase	194
S22	TX Alprazolam or Alprazolan or Alprox or "Apo-Alpraz" or Cassadan or Esparon or Kalma or "Novo-Alprazol" or "Nu-Alpraz" or Ralozam or Tafil or Trankimazin or Xanax	Expanders - Apply related words Search modes - Boolean/Phrase	173
S21	(MH "Alprazolam")	Expanders - Apply related words Search modes - Boolean/Phrase	84
S20	TX "substance use" or "substance usage"	Expanders - Apply related words Search modes - Boolean/Phrase	14107
S19	TX Diazepam or Apaurin or Diazemuls or Faustan or Relanium or Seduxen or Sibazon or Stesolid or Valium or Nordazepam or Calmday or Dealkylprazepam or Demethyldiazepam or Deoxydemoxepam or Desmethyldiazepam or Nordaz or Nordiazepam or Norprazepam or "Tranxilium N" or Vegesan	Expanders - Apply related words Search modes - Boolean/Phrase	730
S18	(MH "Diazepam")	Expanders - Apply related words Search modes - Boolean/Phrase	448
S17	TX Pentobarbital or Diabutal or Etaminal or Ethaminal or Mebubarbital or Mebumal or Nembutal or	Expanders - Apply related words	274

	Pentobarbitone or Sagatal	Search modes - Boolean/Phrase	
S16	(MH "Pentobarbital")	Expanders - Apply related words Search modes - Boolean/Phrase	108
S15	TX Meperidine or Demerol or Dolantin or Dolargan or Dolcontral or Dolin or Dolosal or Dolsin or Isonipecain or Lidol or Lydol or Operidine or Pethidine or Promedol or Dimethylmeperidine or Isopromedol or Trimeperidine or Lomotil or Reasec	Expanders - Apply related words Search modes - Boolean/Phrase	736
S14	(MH "Meperidine")	Expanders - Apply related words Search modes - Boolean/Phrase	466
S13	TX Methadone or Amidone or Biodone or Dolophine or Metadol or Metasedin or Methaddict or Methadose or Methex or Phenadone or Phymet or Physeptone or Pinadone or Symoron	Expanders - Apply related words Search modes - Boolean/Phrase	2470
S12	(MH "Methadone")	Expanders - Apply related words Search modes - Boolean/Phrase	1856
S11	TX Dextropropoxyphene or D-Propoxyphene or Propoxyphene or Darvon or Vicodin	Expanders - Apply related words Search modes - Boolean/Phrase	198
S10	(MH "Propoxyphene")	Expanders - Apply related words Search modes - Boolean/Phrase	97
S9	S2 or S3 or S4 or S5 or S6 or S7 or S8	Expanders - Apply related words Search modes - Boolean/Phrase	53353
S8	TX ((drug or drugs or substance* or opioid* or opiate*	Expanders - Appliquer	53169

	or amphetamine* or amfetamine* or methamphetamine* or methamfetamine or benzodiazepine* or morphine* or methadone* or prescription* or phencyclidine* or solvent* or barbiturate* or depressant* or stimulant* or psychotherap* or psycho-therap* or steroid*) N3 (addict* or abuse* or abusing or abusive or misuse* or mis-use* or misusing or mis-using or non-medical use* or non-medical usage* or illicit* or illegal* or unlawful* or unsanction* or habit* or dependen* or disorder or disorders or relapse* or consumption or diversion*))	les mots connexes Search modes - Boolean/Phrase	
S7	TX ((substance-related or substance-induced) N3 (disorder* or psychosis or psychoses))	Expanders - Appliquer les mots connexes Search modes - Boolean/Phrase	303
S6	(MH "Psychoses, Substance-Induced")	Expanders - Appliquer les mots connexes Search modes - Boolean/Phrase	158
S5	(MH "Inhalant Abuse")	Expanders - Appliquer les mots connexes Search modes - Boolean/Phrase	221
S4	(MH "Substance Abuse, Perinatal")	Expanders - Appliquer les mots connexes Search modes - Boolean/Phrase	1088
S3	(MH "Substance Abuse, Intravenous")	Expanders - Appliquer les mots connexes Search modes - Boolean/Phrase	2089
S2	(MH "Substance Use Disorders")	Expanders - Appliquer les mots connexes Search modes - Boolean/Phrase	8151
S1	TX SBIRT OR SBI OR SBIs	Expanders - Appliquer	238

		les mots connexes<br>Search modes - Boolean/Phrase	

ERIC Database - Studies

(Keywords:SBIRT) and (Publication Type:"Journal Articles" OR Publication Type:"Collected Works Proceedings" OR Publication Type:"Dissertations/Theses" OR Publication Type:"Dissertations/Theses Doctoral Dissertations" OR Publication Type:"Dissertations/Theses Masters Theses" OR Publication Type:"Dissertations/Theses Practicum Papers" OR Publication Type:"ERIC Publications" OR Publication Type:"Reports Evaluative" OR Publication Type:"Reports Research") – 9 hits

((Keywords:"Substance-Related Disorders" or Keywords:"substance abuse" OR Keywords:"drug abuse" OR Keywords:"illicit drug" OR Keywords:"illicit drugs" OR Keywords:marijuana OR Keywords:cannabis OR Keywords:cocaine OR Keywords:crack OR Keywords:heroin OR Keywords:opioid* OR Keywords:amphetamine* OR Keywords:narcotic* OR Keywords:hallucinogen* OR Keywords:"street drug" OR Keywords:"street drugs" OR Keywords:"designer drug" OR Keywords:"designer drugs" OR Keywords:rave OR Keywords:"non-medical use") and (Keywords:brief* or Keywords:short* or Keywords:short-rang* or Keywords:short-term or Keywords:abbreviate* or Keywords:concise or Keywords:early or Keywords:limited or Keywords:time-limited or Keywords:contingency or Keywords:crisis or Keywords:crises or Keywords:emergency or Keywords:emergencies or Keywords:urgent or Keywords:immediate* or Keywords:minimal* or Keywords:minimum or Keywords:quick*) and (Keywords:random* or Keywords:controlled OR Keywords:controled or Keywords:RCT OR Keywords:RCTs or Keywords:placebo)) and (Publication Type:"Journal Articles" OR Publication Type:"Collected Works Proceedings" OR Publication Type:"Dissertations/Theses" OR Publication Type:"Dissertations/Theses Doctoral Dissertations" OR Publication Type:"Dissertations/Theses Masters Theses" OR Publication Type:"Dissertations/Theses Practicum Papers" OR Publication Type:"ERIC Publications" OR Publication Type:"Reports Evaluative" OR Publication Type:"Reports Research") – 167 hits

*These two searches were tagged and downloaded together – **170 records** with overlap*

ERIC Database - Reviews

((Keywords:"Substance-Related Disorders" or Keywords:"substance abuse" OR Keywords:"drug abuse" OR Keywords:"illicit drug" OR Keywords:"illicit drugs" OR Keywords:marijuana OR Keywords:cannabis OR Keywords:cocaine OR Keywords:crack OR Keywords:heroin OR Keywords:opioid* OR Keywords:amphetamine* OR Keywords:narcotic* OR Keywords:hallucinogen* OR Keywords:"street drug" OR Keywords:"street drugs" OR

Keywords:"designer drug" OR Keywords:"designer drugs" OR Keywords:rave OR Keywords:"non-medical use") and (Keywords:brief* or Keywords:short* or Keywords:short-rang* or Keywords:short-term or Keywords:abbreviate* or Keywords:concise or Keywords:early or Keywords:limited or Keywords:time-limited or Keywords:contingency or Keywords:crisis or Keywords:crises or Keywords:emergency or Keywords:emergencies or Keywords:urgent or Keywords:immediate* or Keywords:minimal* or Keywords:minimum or Keywords:quick*) and ((Keywords:"systematic review" or Keywords:"systematic overivew" or Keywords:meta-analy* or Keywords:metaanaly* or Keywords:metanaly* or Keywords:"technology assessment" and Keywords:' or Keywords:"technology assessments" or Keywords:HTA or Keywords:HTAs) NOT (Keywords:random* or Keywords:controlled OR Keywords:controled or Keywords:RCT OR Keywords:RCTs or Keywords:placebo))) and (Publication Type:"Journal Articles" OR Publication Type:"Collected Works Proceedings" OR Publication Type:"Dissertations/Theses" OR Publication Type:"Dissertations/Theses Doctoral Dissertations" OR Publication Type:"Dissertations/Theses Masters Theses" OR Publication Type:"Dissertations/Theses Practicum Papers" OR Publication Type:"ERIC Publications" OR Publication Type:"Reports Evaluative" OR Publication Type:"Reports Research") - **6 hits** *with overlap from CCT search removed*

GREY LITERATURE SOURCES

PsycEXTRA
https://my.apa.org/apa/idm/login.seam?ERIGHTS_TARGET=http%3A%2F%2Fpsycnet.apa.org%2F%3F&cid=518020

Canadian Centre on Substance Abuse library
http://www.ccsa.ca/Eng/KnowledgeCentre/OurDatabases/LibraryCollection/Pages/default.aspx

Centre for Addiction and Mental Health http://www.camh.net/

Substance Abuse and Mental Health Services Administration http://www.samhsa.gov/

National Institute on Drug Abuse http://www.drugabuse.gov/

Centre for Addictions Research of BC http://www.carbc.ca/

College of Physicians and Surgeons of Ontario http://www.cpso.on.ca/

Campbell Collaboration http://www.campbellcollaboration.org/

Australian Drug Information Network http://www.adin.com.au/content.asp?Document_ID=1

European gateway on alcohol, drugs and addictions http://www.addictionsinfo.eu/

European Monitoring Centre for Drugs and Drug Addiction http://www.emcdda.europa.eu/

DrugScope http://www.drugscope.org.uk/

Sources searched from CADTH Grey Matters checklist

- Agence d'évaluation des technologies et des modes d'intervention en santé (AETMIS), Québec http://www.aetmis.gouv.qc.ca/
- Centre for Health Services and Policy Research (CHSPR), University of British Columbia http://www.chspr.ubc.ca/
- Institute for Clinical Evaluative Sciences (ICES), Ontario http://www.ices.on.ca/
- World Health Organization Regional Office for Europe (WHO). Health Evidence Network (HEN) http://www.euro.who.int/en/what-we-do/data-and-evidence/health-evidence-network-hen/publications
- Australian Government Department of Health and Ageing. Medical Services Advisory Committee (MSAC) http://www.msac.gov.au/
- Joanna Briggs Institute http://www.joannabriggs.edu.au/
- Southern Health. Centre for Clinical Effectiveness (CCE) http://www.southernhealth.org.au/page/Health_Professionals/CCE/
- Belgian Health Care Knowledge Centre (KCE) http://www.kce.fgov.be/index_en.aspx?SGREF=5212
- Haute Autorité de santé (HAS) / French National Authority for Health http://www.has-sante.fr/portail/jcms/c_946986/english-toutes-nos-publications-ligne-principale?portal=c_226237
- Health Service Executive (HSE) / Feidhmeannacht na Seirbhíse Sláinte http://www.hse.ie/eng/services/Publications/
- De Gezondheidsraad / Health Council of the Netherlands http://www.gezondheidsraad.nl/en
- Norwegian Knowledge Centre for the Health Services / Nasjonalt kunnskapssenter for helsetjenesten http://www.kunnskapssenteret.no/Forsiden
- University of Birmingham. Aggressive Research Intelligence Facility (ARIF) http://www.arif.bham.ac.uk
- National Health Service for Wales http://www.attract.wales.nhs.uk
- Intute http://www.intute.ac.uk/search.html
- NHS National Institute for Clinical Excellence (NICE) http://www.nice.org.uk/
- NHS Quality Improvement Scotland http://www.nhshealthquality.org/nhsqis/1816.140.144.html
- Agency for Healthcare Research and Quality (AHRQ). Technology Assessments http://www.ahrq.gov/clinic/techix.htm, Evidence-based Practice Centers' evidence reports and technology assessments http://www.ahrq.gov/clinic/epcquick.htm, EPC Topics in Process http://www.ahrq.gov/clinic/epc/epcprogress.htm, Effective Health Care Reports http://effectivehealthcare.ahrq.gov/healthInfo.cfm?infotype=all
- Department of Veterans Affairs Research & Development General publications http://www.research.va.gov/resources/pubs/default.cfm, VA Technology Assessment Program (VATAP) http://www4.va.gov/VATAP/index.asp
- ECRI Institute http://www.ecri.org/
- University HealthSystem Consortium (UHC) http://www.uhc.edu/
- McMaster University. Centre for Health Economics and Policy Analysis (CHEPA) http://www.chepa.org/

- Institute of Health Economics (IHE) http://www.ihe.ca
- Centre for Health Economics Research and Evaluation (CHERE) http://datasearch.uts.edu.au/chere/research/research_reports.cfm
- European Network of Health Economic Evaluation Databases (EURONHEED) http://infodoc.inserm.fr/euronheed/Publication.nsf
- Brunel University, UK. Health Economics Research Group (HERG) http://www.brunel.ac.uk/about/acad/herg
- University of Aberdeen. Health Economics Research Unit (HERU) http://www.abdn.ac.uk/heru/
- Canadian Medical Association. CMA Infobase. http://www.cma.ca/index.php/ci_id/54316/la_id/1.htm
- Ontario Guidelines Advisory Committee. (GAC) http://www.gacguidelines.ca/index.cfm?pagepath=GAC_Endorsed_Guidelines&id=21080
- British Columbia Ministry of Health Services http://www.bcguidelines.ca/gpac/alphabetical.html
- Aetna, Inc. Clinical Policy Bulletins http://www.aetna.com/healthcare-professionals/policies-guidelines/medical_clinical_policy_bulletins.html (medical), http://www.aetna.com/healthcare-professionals/policies-guidelines/pcpb_menu.html (pharmaceutical)
- Institute for Clinical Systems Improvement (ICSI) http://www.icsi.org/guidelines_and_more/index.aspx?catID=12
- National Guideline Clearinghouse, US http://www.guideline.gov
- New Zealand Guidelines Group (NZGG) http://www.nzgg.org.nz/index.cfm
- NHS National Institute for Clinical Excellence (NICE). NICE guidelines http://www.nice.org.uk/page.aspx?o=guidelines.completed
- TRIP database http://www.tripdatabase.com/index.html
- ClinicalStudiesResults.org. PhRMA Clinical Study Results Database http://www.clinicalstudyresults.org (ongoing trials)
- Thomson Centerwatch. CenterWatch Clinical Trials Listing Service http://www.centerwatch.com (ongoing trials)
- U.S. National Institutes of Health. ClinicalTrials.gov http://clinicaltrials.gov/ct/gui (ongoing trials)
- World Health Organization (WHO). International Clinical Trials Registry Platform Search Portal http://apps.who.int/trialsearch/ (ongoing trials)
- Bandolier (Oxford, UK) http://www.medicine.ox.ac.uk/bandolier/
- New Zealand Complementary and Alternative Therapies Evidence-based Summaries. Complementary and Alternative Medicine www.cam.org.nz/
- UK National Health Service. National electronic Library for Medicines (NeLM). http://www.nelm.nhs.uk/en/NeLM-Area/Evidence/
- National Library of Medicine (NLM). NLM Gateway. http://gateway.nlm.nih.gov/gw/Cmd

- University of Queensland. OTseeker – Occupational Therapy Systematic Evaluation of Evidence. http://www.otseeker.com
- University of York. Centre for Reviews and Dissemination (CRD), http://www.crd.york.ac.uk/crdweb/
- National Institute for Clinical Excellence (NICE). Evidence in Health and Social Care. http://www.evidence.nhs.uk/
