# Supplementary material for: Effectiveness of brief interventions as part of the Screening, Brief Intervention and Referral to Treatment (SBIRT) model for reducing the nonmedical use of psychoactive substances: a systematic review
Source: Syst Rev. 2014 May 24;3:50. doi: 10.1186/2046-4053-3-50 (PMC4042132; doi:10.1186/2046-4053-3-50)
Supplement: Additional file 3 — Screening questions used by reviewers to decide if records met inclusion criteria. [file 2046-4053-3-50-S3.pdf]

Additional file 3: *Screening questions used by reviewers to decide if records met inclusion criteria*

Level 1 questions (title and abstract)

1. Study is written in:
   - English
   - French
   - Other than English or French
   - Unclear

2. Does this study evaluate a brief intervention (BI) targeting the non-medical use of psychoactive substances (including the non-medical use of pharmaceuticals or the use of legal inhalants or solvents for their intoxicating effects)?
   - Yes/unclear
   - No, other (eg, any alcohol, nicotine, caffeine only study; animal or laboratory study; other treatment)

3. Was the intervention provided to those NOT seeking or receiving treatment for substance use?
   - Yes, not seeking or receiving treatment / Unclear
   - No, they are seeking or receiving treatment / Not applicable

4. Is this possibly a primary study design of interest?
   - Yes/unclear
   - No, a BI protocol
   - No, a systematic review
   - No, other (e.g. editorial, comment, letter, retrospective, case report)

Level 2 questions (full text articles)

1. The full-text report for this record is not available.
   - Yes

2. Study is written in:
   - English
   - French
   - Other than English or French

3. Is this a primary study design of interest?
   - Yes, RCT or cluster RCT
   - Yes, conference abstract (try to seek full text)

- Yes, is nonRCT, CBA, ITS
- No, is a protocol
- No, is a systematic review/meta-analysis
- No, is a secondary analysis of a study (query to locate original article)
- No, other (eg, editorial, comment, letter, case report, case series, qualitative only, survey only, retrospective design, no control group)

4. Population: Are individuals included by means of recruitment or asked to volunteer to take part in the study (before screening takes place)?
- Yes, recruited/voluntary
- No
- Unsure
- Not applicable (ONLY for "No, other" or "No, systematic review" answers in Q2)

5. Population: Are individuals seeking/receiving treatment for ANY substance, from or in a treatment facility/community treatment program, or eligibility for participation is that they have been previously diagnosed with a substance use disorder?
- Yes, treatment seeking / receiving / facility / program OR previously diagnosed
- No
- Unsure

6. Population: Are individuals being universally screened upon entering a program or organization (e.g., hospital, primary care clinic, prison, school program, etc.) as part of a standard intake procedure or process? (Note: Screening may be explicitly stated or simply implied)
- Yes, universally screened
- No
- Unsure

7. Is this study assessing a brief intervention (BI)?
- Yes, BI explicitly defined and has 4 or less sessions
- Yes, BI explicitly defined but has >4 sessions
- Yes, BI explicitly defined but unclear number of sessions (contact authors)
- No, does not meet the BI definition (e.g., other intervention)
- Unclear whether a BI (contact authors)

8. Is this intervention targeting the non-medical use of psychoactive substances and presents those results separately)?
- Yes/presents those results separately
- No, does not present results separately
- No, other (eg, alcohol, nicotine, caffeine only)

9. Is the BI delivered in a one-to-one, verbal format, directed to the indivdual at risk?
- Yes
- No, not directed to the indivdual at risk
- No, group (2+ people receiving) or online/computer intervention without video (text only)
- No, other (e.g., provision of written materials without accompanying discussion)
- Unclear, does not state format but in-person presumed

Level 3 questions (full text articles)

1. Population: Are individuals being universally screened?
- Yes
- No
- Unclear, contact author. Related text from article:

2. Population: Is the study population 12 years (or equivalent based on schooling) or older?
- Yes
- No
- Unsure, contact author

3. Intervention: Is this study assessing a brief intervention (BI)?
- Yes, BI explicitly defined and has 4 or less sessions
- Yes, BI explicitly defined but has >4 sessions
- Yes, BI explicitly defined but unclear number of sessions (contact authors)
- No, does not meet the BI definition (e.g., other intervention)
- Unclear whether a BI (contact authors)

4. Intervention: Does the BI have at least one of the following FRAMES elements? (feedback on behaviour and consequences, responsibility to change, advice, menu of options to bring about change, empathy, self-efficacy for change)
- Yes
- No
- Unclear, contact authors

5. Intervention: Is this intervention targeting the non-medical use of psychoactive substances and presents those results separately)
- Yes/presents those results separately
- No, does not present results separately
- No, other
- Unclear % of baseline population screened positive for substance not of interest only (contact authors)

6. Intervention: Is the BI delivered in a one-to-one, verbal format, directed to the individual at risk?
- Yes
- No, not directed to the individual at risk
- No, group (2+ people receiving) or online/computer intervention (text only)
- No, other (e.g., provision of written materials without accompanying discussion)
- Unclear, does not state format but in-person presumed

7. Comparison: Is there a comparison group of interest? (e.g., no BI, pamphlets or other info only, delayed intervention)
- Yes
- No, not comparison of interest
- Unclear comparison group (e.g., 'control group'), contact author

8. Should this study be excluded for another reason?
- Yes (specify reason)

Level 4 – Contact author

Can this study now be included after contacting the author?
- Yes, because the following eligibility criteria are now met (specify)
- Yes, did not obtain information from authors but interpretation of information is reasonable to consider eligible
- No, was not able to obtain a response from one or more authors
- No, is a conference abstract that should be tracked for future available data
- Yes but is the companion to refid (specify)

Level 4 – Collate multiple reports

Is this study included in the review?
- Yes
- Yes but is a companion report of refid (specify)
