# Supplementary material for: Effectiveness of brief interventions as part of the Screening, Brief Intervention and Referral to Treatment (SBIRT) model for reducing the nonmedical use of psychoactive substances: a systematic review
Source: Syst Rev. 2014 May 24;3:50. doi: 10.1186/2046-4053-3-50 (PMC4042132; doi:10.1186/2046-4053-3-50)
Supplement: Additional file 4 — Data extraction form. [file 2046-4053-3-50-S4.pdf]

Additional file 4: *Data extraction form*

Publication details
- Citation
- Author contact information
- Language of publication
- Publication type (full report, journal; full, non-journal; report not in public domain; conference abstract)

Study characteristics
- Country of study conduct
- Study funding and other author conflict of interest
- Timelines (study conduct, recruitment/screening period, other, not reported)
- Study design (single site RCT, multisite RCT, cluster RCT)
- Additional information

Population characteristics
- Study inclusion and exclusion criteria
- Number of participants screening
- Number and reasons for exclusions/withdrawals
- Number of participants/centres randomized
- Incentives offered to participate in the study
- Age, sex, race/ethnicity, and other baseline demographic information
- Screening: tool/method and description, whether validated, setting, administration method, professional conducting screening, training received to administer screening, additional information

Intervention characteristics
- Number randomized
- Provider delivering intervention, training provided to administer intervention, format/mode of delivery, setting, intervention content/elements, theoretical basis, substance(s) targeted, whether co-intervention provided, length of session, duration of intervention, fidelity assessment and results, additional information

Comparator groups
- Number randomized
- Details on comparator

Risk of bias items
- Items (refer to Appendix F)
- Judgement of risk of bias (low, high, or unclear)
- Information from report or correspondence that supports the judgement

Outcomes

- Outcome measured and definition
- Data type (dichotomous, continuous, ordinal)
- Follow-up time(s)
- Unit of analysis
- Dichotomous: Participants with event and total analyzed per arm
- Continuous: unit of measurement, interpretation of measurement, mean/median/mean change from baseline and measure of dispersion (SD, SE, IQR low/high, range). Text box to report ordinal data.
- Statistical tests conducted and results
- Other data or additional information
