# Supplementary material for: Effectiveness of brief interventions as part of the Screening, Brief Intervention and Referral to Treatment (SBIRT) model for reducing the nonmedical use of psychoactive substances: a systematic review
Source: Syst Rev. 2014 May 24;3:50. doi: 10.1186/2046-4053-3-50 (PMC4042132; doi:10.1186/2046-4053-3-50)
Supplement: Additional file 6 — Potentially relevant ongoing trials. [file 2046-4053-3-50-S6.pdf]

Additional file 6: *Potentially relevant ongoing trials*

**Identifier, Start date** (estimated completion date)	**Study Name**	**Methods**	**Participants**	**Intervention**	**Control**	**Outcomes**	**Contact Information**	**Notes**
HSRP20081113<br><br>July 2007 (June 2010)	Brief intervention to reduce substance use in South African primary care	RCT, South Africa	18-24 y. Public primary health care clinic.	Brief motivational intervention	NR	Reduce hazardous drug and alcohol use	Jennifer Mertens jennifer.mertens@ kp.org	Source: NLM Gateway
NCT01501318<br><br>February 2012 (Completed November 2012 – no published reports as of December 2013)	Brief education to reduce health care consumers' risky substance use behaviors: How brief is brief?	RCT, United States	40 participants 18 y or older. Emergency room.	Brief motivational interview, one session (5-15 min), plus personalized feedback report	Personalized feedback report	Substance use behaviors, consequences, intentions	Matthew G Hile, PhD matthew.hile@ mimh.edu	Source: clincal trials.gov
HSRP20101044<br><br>September 2009 (September 2012)	Comparative effectiveness and feasibility of screening, brief intervention and referral services (SBIRT) in a general dental clinic	2y RCT, United States	1175 participants who are smokers and at-risk substance users. General, academic dentistry clinic.	1) Brief intervention for smokers only<br>2) Brief intervention for smoking + other substance use	Wait-list that includes general dental health counselling	Not specified	TF Babor babor@nso.uchc.edu	Source: NLM Gateway

**Identifier, Start date** (estimated completion date)	**Study Name**	**Methods**	**Participants**	**Intervention**	**Control**	**Outcomes**	**Contact Information**	**Notes**
NCT01207791<br><br>October 2010 (Completed June 2012 - no published reports as of December 2013)	Screening, motivational assessment, referral, and treatment in emergency departments (SMART-ED)	Multisite RCT, United States	1285 participants 18 y or older screened positive for use of a non-alcohol, non-nicotine substance. Emergency department.	(1) Screening, assessment, referral to treatment, and written information (2) Brief intervention (30-min) and written information + two telephone boosters (15-min each)	Minimal sreening only	Frequency of use, quantity of use, change in substance, negative consequences of use, decision to attend treatment, other health care use	Alyssa A Forcehimes, Ph.D. aforcehimes@ comcast.net	Source: Clinical Trials.gov, Bogenshutz 2011 {92}
NCT00876941<br><br>April 2009 (March 2013)	Assessing screening plus brief interventions's resulting efficacy (ASPIRE) to stop drug use	RCT, United States	450 participants 18y or older with ASSIST score of >4 for drug use. 200 participants with ASSIST score of 2-3 will be included in secondary and exploratory analyses. Hospital-based	(1) Enhanced brief intervention. One in-perison session plus optional telephone booster call, each 30-45 min. (2) Standard brief intervention. One in-person session, 10-15 min.	Informed that at risk of substance use hazards + written list of available resources.	Substance use, substance use consequences, HIV risk behaviours, substance use treatement use, costs.	Richard Saitz, MD Boston Medical Center	Source: Clinical trials.gov

**Identifier, Start date** (estimated completion date)	**Study Name**	**Methods**	**Participants**	**Intervention**	**Control**	**Outcomes**	**Contact Information**	**Notes**
			primary care practice.					
HSRP20101435<br><br>September 2008 (June 2013)	Computer versus therapist-delivered brief intervention for drug abuse in primary care	RCT, United States	680 participants at risk for heavy or problem alcohol and drug use. Primary care setting assumed.	(1) Therapist-delivered brief intervention (2) Computer-delivered brief intervention	(1) Computer-delivered health screen (2) Assessment only	Substance use, economic outcomes	Svikis DS dssvikis@vcu.edu	Source: NLM Gateway
NCT00913770<br><br>September 2008 (June 2013)	Models of screening, brief intervention with a facilitated referral to treatment (SBIRT) for opioid patients in the emergency department	RCT, United States	360 participants 18 y or older with opioid dependence and positive urine screen. Emergency department.	(1) Brief intervention with facilitated referral to treatment (2) Brief intervention plus buprenorphine treatment	Handout (local substance use treatment centres)	Substance use treatment program, opioid use, HIV risk behaviours, health care service utilization.	Gail D'Onofrio, MD, MS gail.donofrio@yale. edu	Source: Clinical Trials.gov

**Identifier, Start date** (estimated completion date)	**Study Name**	**Methods**	**Participants**	**Intervention**	**Control**	**Outcomes**	**Contact Information**	**Notes**
NCT01003834<br><br>November 2010 (September 2013)	Computerized screening, brief intervention, and referral to treatment in primary care (SBIRT)	RCT, United States	680 participants 18-65 y with substance use. Primary care clinic.	1) Computer-delivered brief intervention based on motivational interviewing 2) Therapist-delivered motivational intervention	(1) Computerized health screening only (standard care) (2) Computerized health screening and substance use assessment only	Substance use, medical and psychosocial functioning, economic outcomes.	Pam Dillon, PharmD 804-827-1519 pmdillon@vcu.edu<br><br>Lynn Hull, PhD 804-828-5596 hulllc@vcu.edu	Source: Clinical trials.gov
HSRP20101141<br><br>Aug 2009 (May 2014)	Optimizing screening, brief interventions, and referral to treatment (SBIRT) for drug-using patients in an inner-city emergency department	Factorial RCT, United States	900 patients 18-60y, screen positive for drug use. Emergency department.	(1) Computer-delivered brief intervention + written information (2) Intervener-delivered brief intervention Intervener brief intervention	Enhance usual care + written information<br><br>Second phase randomization to adapted motivational enhance	Drug use, other health measures (including physical and mental health, HIV risk behaviour)	Blow FC fredblow@umich. edu	Source: NLM Gateway

**Identifier, Start date** (estimated completion date)	**Study Name**	**Methods**	**Participants**	**Intervention**	**Control**	**Outcomes**	**Contact Information**	**Notes**
				+ written information<br><br>Second phase randomization to adapted motivational enhancement therapy or enhanced usual care to be delivered two months following initial allocation.	ment therapy or enhanced usual care to be delivered two months following initial allocation.			
NCT01433692<br><br>March 2012 (July 2014)	CANABIC: CANnabis and Adolescents, a Brief Intervention to reduce their Consumption	Cluster RCT, 3 regions in France.	750 participant 15-25 y with cannabis consumption. Primary care practices.	Brief intervention	No intervention	Cannabis, tobacco, alcohol use.	Patrick Lacarin placarin@chu-clermontferrand.fr	Source: Clinical Trials.gov
NCT01532934<br><br>August 2009 (July 2014)	The impact of psychopathic traits on the efficacy of a brief intervention for substance use	RCT, United States	120 participants 18 y or older who are offenders in a jail diversion program	Brief motivational enhancement, four sessions (45-min each)	Standard Care	Criminal recidivism, substance use	Marc T. Swogger, Ph.D. marc_swogger@urmc.rochester.edu	Source: Clinical Trials.gov

**Identifier, Start date** (estimated completion date)	**Study Name**	**Methods**	**Participants**	**Intervention**	**Control**	**Outcomes**	**Contact Information**	**Notes**
NCT00877331<br><br>April 2009 (September 2014)	Brief intervention in primary care for problem drug use and abuse	RCT, United States	1000 participants 18 y and older using recreational drugs or prescription drugs not as prescribed in past 3 mo	Brief intervention (motivational interviewing). One in-person session, 30-45 min, plus 1-wk brief telephone call.	Enhanced care as usual.	Substance use, treatment program attendance, intervention fidelity, negative consequences of use, other health measures, costs.	Principal Investigator: Peter P Roy-Byrne, MD University of Washington	Source: Clinical Trials.gov
NCT01124591<br><br>June 2010 (December 2014)	Brief intervention for drug misuse in the emergency department (BIDMED)	Multisite RCT, United States	1100 participants 18-64 y with subcritical illness or injury and illicit or prescription drug misuse. Academic trauma centre and community hospital.	Brief motivational intervention, two sessions	Screening only	Drug misuse, behaviours associated with drug misuse, physical and psychosocial health, socioeconomic consequences, uptake of HIV and hepatitus B/C screening	Roland C Merchant, MD, ScD rmerchant@lifespan.org<br><br>Janette Baird, PhD jbaird@lifespan.org	Source: Clinical Trials.gov

**Identifier, Start date** (estimated completion date)	**Study Name**	**Methods**	**Participants**	**Intervention**	**Control**	**Outcomes**	**Contact Information**	**Notes**
NCT01597856<br><br>December 2012 (December 2016)	Evaluation and treatment of substance use in veterans with PTSD disability claims	RCT, United States	640 military veterans 18-65 y applying for post-traumatic stress disorder disability claim	Brief intervention	No intervention	Attending treatment, substance use, severity of post-traumatic stress disorder	Marc I Rosen, MD marc.rosen@va.gov<br><br>Theodore Speroff, PhD ted.speroff@va.gov	Source: Clinical Trials.gov
ACTRN12611000136909<br><br>March 30, 2010 (none provided)	The Ecstasy Check-up: A multi-site trial of a brief intervention for ecstasy use	Multisite RCT	175 participants 16 y or older, ectasy use	Brief intervention (motivational interviewing), one session plus written information	Written information (educational factsheet about ecstasy)	Ecstasy use, Client satisfaction of E Check Up discussion.	Dr Laila Khawar l.khawar@unsw.edu.au<br><br>Dr Melissa Norberg m.norberg@unsw. edu.au	Source: Australian New Zealand Clinical Trials Registry
NR<br><br>NR	Health services interventions for patients in the emergency department with alcohol and/or drug use disorders: findings from Project Health Link	RCT	1141 patients, abuse or dependence on alcohol, cannabis, stimulants, or opioids. Emergency department.	(1) 5-session case management intervention (2) 2-session brief motivational intervention	Brochure	Linkage to substance use assessment, referral, and treatment entry	Not reported	Abstract [A]

Abbreviations: HIV= human immunodeficiency virus; min=minutes; mo= months; NLM=National Library of Medicine; NR=Not reported; PTSD=post-traumatic stress disorder; RCT=randomized controlled trial; wk; weeks; y=year.

[A] Identified as a conference abstract: Massey, L. S., Walton, M. A., Booth, B. M., Barry, K. L., Cunningham, R. M., Chermack, S. T., and Blow, F. C. Alcoholism: Clinical and Experimental Research 2011;35:144A. Abstract no 0534. Supported by NIAAA #AA014665 and NIDA #DA016591
