# Supplementary material for: Effectiveness of brief interventions as part of the Screening, Brief Intervention and Referral to Treatment (SBIRT) model for reducing the nonmedical use of psychoactive substances: a systematic review
Source: Syst Rev. 2014 May 24;3:50. doi: 10.1186/2046-4053-3-50 (PMC4042132; doi:10.1186/2046-4053-3-50)
Supplement: Additional file 7 — Excluded studies during full text screening. [file 2046-4053-3-50-S7.pdf]

Additional file 7. *Excluded studies during full text screening*

Level 1: Language not English or French

These articles were assessed based on the abstract and determined as possibility relevant for content but excluded for language; readers would need to consult the full text report to determine whether the reports meet all eligibility criteria.

RefID:3812. Harada, T.. [A new strategy for drug abuse treatment: the rationale and practice of the Matrix Model]. [Japanese]. Nihon Arukoru Yakubutsu Igakkai Zasshi 2010. 45 (6) 557-568. **Keywords:** Multifile Reviews/Cognitive Therapy/mt [Methods]/Counseling/Evidence-Based Medicine/Humans/Models/Psychological/Substance-Related Disorders/th [Therapy].

RefID:7428. Pirskanen, Marjatta and Pietilä, Anna Maija. Phased development process of an early intervention model to promote adolescents' abstinence from substance use: dialog between research and practice [Finnish]. Hoitotiede 2010. 22 (3) 206-217. **Keywords:** CINAHL/early intervention/Substance Abuse -- Prevention and Control -- In Adolescence/Theory Construction/adolescence/human/Theory Validation.

RefID:10781. Isela Mart+¡nez Mart+¡nez, Kalina, Leticia Salazar Garza, Martha, Javier Pedroza Cabrera, Francisco, Mariana Ruiz Torres, Gabriela, and Enrique Ayala Vel+ízquez, H. +. Resultados preliminares del Programa de Intervenci+¦n Breve para Adolescentes que Inician el Consumo de Alcohol y otras Drogas. (Spanish). Salud Mental 2008. 31 (2) 119-127. **Keywords:** INTERVENTION (Social services)/TEENAGERS/substance abuse/Research/Drugs/alcohol/AGGRESSION (Psychology) in adolescence/Drug Utilization/COMPULSIVE behavior/Mental Health/drug use/Adolescent/alcohol and drug abuse/brief intervention/cognitive-behavioral treatment/abuso de alcohol y otras drogas/Adolescencia/intervenci+¦n breve/tratamiento cognoscitivo-conductual.

Level 2: Full-text report unavailable

RefID:2095. Moon, K. T.. Targeted interventions for adolescents to prevent drug use. American family physician 2010. 81 (11) 1369-1374. **Keywords:** Adolescent/article/*character state/*Child Behavior/Cognitive Therapy/coping behavior/*drug use/*education program/health program/high risk behavior/human/impulsiveness/intervention study/*prevention study/Preventive Medicine/Program Development/psychoeducation/substance abuse/Cannabis/Cocaine.

RefID:2938. Demyan, Amy L.. The effects of a brief, mass-media intervention on attitude and intention to seek professional psychological treatment. Dissertation Abstracts International: Section B: The Sciences and Engineering 2009. 70 (4-B) 2570-#End Page#. **Keywords:** *Mass

Media/*Mental Health Services/*Psychological Assessment/*Public Service Announcements/Intervention.

RefID:2984. Ball, Samuel A., Martino, Steve, Nich, Charla, Frankforter, Tami L., Van Horn, Deborah, Crits-Christoph, Paul, Woody, George E., Obert, Jeanne L., Farentinos, Christiane, and Carroll, Kathleen M.. "Site matters: Multisite randomized trial of motivational enhancement therapy in community drug abuse clinics": Correction. Journal of consulting and clinical psychology 2009. 77 (2) 336-#End Page#. **Keywords:** *Brief Psychotherapy/*drug abuse/*Drug Rehabilitation/*Intervention/*Motivation Training/Interviewing.

RefID:5641. Deren, S., Davis, W. R., Beardsley, M., Tortu, S., and Clatts, M.. Outcomes of a risk-reduction intervention with high-risk populations: the Harlem AIDS project. AIDS education and prevention : official publication of the International Society for AIDS Education 1995. 7 (5) 379-390. **Keywords:** Central/Acquired Immunodeficiency Syndrome [prevention & control]/Community-Institutional Relations/Follow-Up Studies/Health Behavior/Health Education/New York City/Outcome Assessment (Health Care)/Risk Factors/Sexual Behavior/Socioeconomic Factors/Substance Abuse,Intravenous/Urban Health/Adult/Female/Humans/Male.

RefID:5736. Gonzales, G., Feingold, A., Oliveto, A., Gonsai, K., and Kosten, T. R.. Depression and gender predict outcome for desipramine and contingency management in cocaine and opiate dependence. Drug and alcohol dependence 2001. 63 Suppl 1 (#Issue#) 58-#End Page#. **Keywords:** Central.

RefID:5764. Kuo, I., Brady, J., Schwartz, R., Butler, C., Brooner, R., Vlahov, D., and Strathdee, S.. Feastibilty of mobile laam treatment among injection drug users refered from a needle exchange program. Proceedings of the 63rd Annual Scientific Meeting of College on Problems of Drug Dependence; 2001 June 12 17; Scottsdale, Arizona, USA 2001. #volume# (#Issue#) S119-#End Page#. **Keywords:** Central.

RefID:5828. Huber, A., Shoptaw, S., Gulati, V., and Gonzales, R.. SErtraline and contingency management as treatment for methamphetamine dependence. Proceedings of the 63rd Annual Scientific Meeting of College on Problems of Drug Dependence; 2001 June 12 17; Scottsdale, Arizona, USA 2001. #volume# (#Issue#) S95-#End Page#. **Keywords:** Central.

RefID:5905. Carroll, K. M., Bryce, L., and Sheehan, J. P.. Child welfare referral to substance abuse treatment. 153rd Annual Meeting of the American Psychiatric Association; 2000 May 13 18; Chicago, IL 2000. #volume# (#Issue#) No-#End Page#. **Keywords:** Central.

RefID:5919. Kosten, T. R., Oliveto, A., and Feingold, A.. Desipramine and contingency management for cocaine abuse. Proceedings of the 39th Annual Meeting of the American College of Neuropsychopharmacology; 2000 Dec 10 14; San Juan; Puerto Rico 2000. #volume# (#Issue#) #Start Page#-#End Page#. **Keywords:** Central.

RefID:6005. Rawson, R. A., McCann, M., and Ling, W.. Relapse prevention and contingency management aproaches for the treatment of cocaine abuse disordes. Proceedings of the 65th Annual Scientific Meeting of the College on Problems of Drug Dependence; 2003 June; Bal Harbour, Florida.USA 2000. #volume# (#Issue#) S178-#End Page#. **Keywords:** Central.

RefID:6095. Stotts, A., Schmitz, J., Sayre, S., Schwebel, A., and Grabowski, J.. Brief motivational enhancement therapy prior to relapse prevention for cocaine dependent patients. Proceedings of the 61st Annual Scientific Meeting of the College on Problems of Drug Dependence; 1999 June; Acapulco, Messico. 1999. #volume# (#Issue#) 218-#End Page#. **Keywords:** Central.

RefID:6158. Petry, N.. Low cost, community-baed contingency management treatment for cocaine dependence. Proceedings of the 61st Annual Scientific Meeting of the College on Problems of Drug Dependence; 1999 June; Acapulco, Messico. 1999. #volume# (#Issue#) 32-#End Page#. **Keywords:** Central.

RefID:6202. Rawson, R. A.. Relapse prevention and contingency management of cocaine abuse. Proceedings of the 152nd Annual Meeting of the American Psychiatric Association; 1999 May 15 20th; Washington, DC, USA 1999. #volume# (#Issue#) #Start Page#-#End Page#. **Keywords:** Central.

RefID:7060. Rani, Shobha and Byrne, Hanora. Dual diagnosis training: a six step approach to curriculum development. International Journal of Nursing Education 2012. 4 (1) 73-76. **Keywords:** CINAHL/Curriculum Development/Diagnosis,Dual (Psychiatry)/Education,Nursing/psychoeducation/Behavioral Objectives/Needs Assessment/Problem Identification/Teaching Methods.

RefID:8654. Abstracts: American Society of Addiction Medicine, 34th Annual Medical-Scientific Conference, May 1-4, 2003, Toronto, Ontario, Canada. Journal of addictive diseases 2003. 22 (2) 105-149. **Keywords:** CINAHL/substance abuse/Substance Dependence/Medical Organizations/Congresses and Conferences -- Ontario/Ontario.

RefID:8765. Chitthathairatt, S., Lapvongwatana, P., Tongwichian, S., Nontasorn, T., and Tongprateep, T.. Effects of the cognitive-behavioral approach and Buddhist practice on amphetamine prevention among adolescents. Thai Journal of Nursing Research 2004. 8 (3; The evidence from the study revealed that the CBABP program affected on amphetamine preventive competence among school adolescent students. An effort to develop a booster CBABP program for sustainable competence is recommended
) 182-191. **Keywords:** CINAHL/Adolescent Behavior/Amphetamines -- In Adolescence/Buddhism -- In Adolescence/Cognitive Therapy -- In Adolescence/Substance Abuse -- Prevention and Control -- In Adolescence/adolescence/chi square test/Clinical Assessment Tools/Coefficient Alpha/Comparative Studies/Content Validity/Control (Psychology)/Descriptive Statistics/Evaluation Research/Family Relations/Fisher's Exact Test/Kuder-Richardson Coefficient/multivariate analysis of variance/One-Way Analysis of Variance/outcome assessment/P-Value/Parenting/Power Analysis/Pretest-Posttest Design/Prospective

Studies/Quasi-Experimental Studies/Questionnaires/Repeated Measures/Risk Taking Behavior/Scales/Schools,Middle/Self Concept/Self Report/Students,Middle School/Summated Rating Scaling/T-Tests/Thailand/human.

RefID:8984. Program abstracts: 55th Annual Scientific Meeting, The Gerontological Society of America, November 22-26, 2002, Boston, MA, "Relationships in a changing world: from aging cells to aging societies". The Gerontologist 2-10-2002. 42 (#Issue#) 1-435. **Keywords:** CINAHL/Aged/Aging/Geriatrics/Congresses and Conferences -- Massachusetts/Massachusetts.

RefID:8988. Screening for drug abuse. #journal name# 1989. 2 ed (#Issue#) 583-594. **Keywords:** CINAHL/Health Screening/Preventive Health Care/Substance Abuse -- Prevention and Control.

RefID:9250. 3rd World Conference on Neurorehabilitation, April 2-6, 2002, Venice, Italy. Neurorehabilitation & Neural Repair 2001. 15 (4) 255-366. **Keywords:** CINAHL/rehabilitation/neurology/Congresses and Conferences -- Italy/Italy.

RefID:9251. 54 Annual Scientific Meeting "2001 -- "A Gerontological Odyssey: Exploring Science, Society, and Spirituality," November 15-18, 2001, Chicago, Illinois. The Gerontologist 2-10-2001. 41 (#Issue#) 1-399. **Keywords:** CINAHL/Geriatrics/Gerontologic Care/Congresses and Conferences -- Illinois/Illinois.

RefID:9257. Abstracts and presentations from "Making Waves," the College of Occupational Therapists 26th Annual Conference & Exhibition, 11th-14th June 2002, Brighton Centre. #journal name# 2002. #volume# (#Issue#) #Start Page#-133. **Keywords:** CINAHL/occupational therapy/Congresses and Conferences -- England/England/Occupational Therapists -- Organizations.

RefID:9270. Health Disparities: Meeting the Challenge... 35th Annual Communicating Nursing Research Conference/16th Annual WIN Assembly, "Health Disparities: Meeting the Challenge," held April 18-20, 2002, Palm Springs, California. Communicating Nursing Research 2002. 35 (#Issue#) xv-#End Page#. **Keywords:** CINAHL/Special Populations/Research,Nursing/Congresses and Conferences -- California/California/Research,Nursing -- Organizations.

RefID:9280. Orthopaedic proceedings. Journal of Bone & Joint Surgery, British Volume 2002. 84B (#Issue#) 109-197. **Keywords:** CINAHL/orthopedic surgery/orthopedics/Congresses and Conferences.

RefID:9358. Counseling to prevent household and recreational injuries. #journal name# 1989. 2 ed (#Issue#) 659-685. **Keywords:** CINAHL/Counseling/Accidents,Home -- Prevention and Control/patient education/Preventive Health Care/Health Promotion.

RefID:9371. Making waves: Brighton 2002, College of Occupational Therapists 26th Annual Conference & Exhibition, 11th-14th June 2002, Brighton Centre. #journal name# 2001. #volume# (#Issue#) #Start Page#-135. **Keywords:** CINAHL/occupational therapy/Congresses and

Conferences -- United Kingdom/United Kingdom/Occupational Therapists -- Organizations.

RefID:9375. Orthopaedic Section abstracts: platform presentations (abstracts OPL1-OPL64). Journal of Orthopaedic & Sports Physical Therapy 2006. 36 (1) A1-25. **Keywords:** CINAHL/Physical Therapy/orthopedics/Congresses and Conferences -- California/California/American Physical Therapy Association.

RefID:10710. Deck, Dennis D.. addressing adolescent substance abuse: an evaluation of washington's prevention and intervention services program. 2001-03 final report. #journal name# 2004. #volume# (#Issue#) #Start Page#-#End Page#. **Keywords:** State Programs/Program Implementation/Program Evaluation/Program Effectiveness/prevention/early intervention/substance abuse/Comprehensive School Health Education/Resistance (Psychology)/Counseling/ERIC RCTS.

RefID:10760. a study of children's attitudes and perceptions about drugs and alcohol. #journal name# 1983. #volume# (#Issue#) #Start Page#-#End Page#. **Keywords:** Adolescents/Alcoholic Beverages/Children/drinking/drug use/Elementary Secondary Education/Marihuana/Peer Influence/Perception/Rural Urban Differences/Sex Differences/Student Attitudes/ERIC RCTS.

RefID:10763. Malvin, Janet and And, Others. evaluation of two alternatives programs for junior high school students. #journal name# 1982. #volume# (#Issue#) #Start Page#-#End Page#. **Keywords:** Affective Objectives/Cross Age Teaching/drug abuse/Junior High School Students/Junior High Schools/Observation/Program Evaluation/Questionnaires/Self Evaluation (Individuals)/Service Learning/Summative Evaluation/Tutoring/ERIC RCTS.

RefID:10766. drug treatment in new york city and washington, d.c.: followup studies. #journal name# 1977. #volume# (#Issue#) #Start Page#-#End Page#. **Keywords:** academic achievement/behavior change/Criminals/Drug Addiction/Drug Rehabilitation/Employment/Followup Studies/Narcotics/Prosocial Behavior/recidivism/Rehabilitation Centers/ERIC RCTS.

Level 2: Language not in English or French

RefID:1060. De Micheli, Denise, Fisberg, Mauro, and Formigoni, Maria Lucia. [Study on the effectiveness of brief intervention for alcohol and other drug use directed to adolescents in a primary health care unit]. Revista da Associacao Medica Brasileira (1992) 2004. 50 (3) 305-313. **Keywords:** Adolescent/Adult/Alcohol-Related Disorders/ep [Epidemiology]/Alcohol-Related Disorders/pc [Prevention & Control]/Brazil/ep [Epidemiology]/Child/Epidemiologic Methods/Female/Humans/Male/Marijuana Abuse/ep [Epidemiology]/Marijuana Abuse/pc [Prevention & Control]/*Patient Education as Topic/st [Standards]/Primary Health Care/st [Standards]/*Process Assessment (Health Care)/st [Standards]/Psychotherapy,Brief/Socioeconomic Factors/Substance-Related Disorders/ep [Epidemiology]/*Substance-Related Disorders/pc [Prevention & Control].

RefID:2394. Van Den Bosch, L. M. C.. Efficacy of dialectical behaviour therapy in the treatment of female borderline patients with and without substance abuse problems: Result of a Dutch study. Tijdschrift voor psychiatrie 2005. 47 (3) 127-137. **Keywords:** alcohol abuse/article/automutilation/*Behavior Therapy/*borderline state/th [Therapy]/Comorbidity/Female/follow up/high risk patient/human/impulsiveness/major clinical study/Netherlands/risk reduction/*substance abuse/suicide attempt/Treatment Outcome.

RefID:2481. Insua, P. and Moncada, S.. Cognition, attitudes and behavior: Changing towards harm reduction associated with drug use among injection drug users. Adicciones 2002. 14 (2) 161-176. **Keywords:** acquired immune deficiency syndrome/Adult/article/behavior modification/controlled study/Female/Health Education/human/*intravenous drug abuse/major clinical study/Male/medical education/Motivation/Patient Satisfaction/*preventive health service.

RefID:3238. Bechdolf, A., Pohlmann, B., Geyer, C., Ferber, C., Klosterkotter, J., and Gouzoulis-Mayfrank, E.. Motivational Interviewing for Patients with Comorbid Schizophrenia and Substance Abuse Disorders: A Review. Fortschritte der Neurologie, Psychiatrie 2005. 73 (12) 728-735. **Keywords:** *drug abuse/*Experimentation/*Interviewing/*Motivation Training/*Schizophrenia/Comorbidity/Dual Diagnosis/methodology/Motivational Interviewing/Readiness to Change.

RefID:4619. Waechter, H. M. and Pudel, V.. Controlled examination of an extremely brief psychotherapy (15 hours) with guided affective imagery. Psychotherapie, Psychosomatik, medizinische Psychologie 1980. 30 (5) 193-205. **Keywords:** Central/guided affective imagery with neurotic & psychosomatic patients/Treatment Effectiveness Evaluation/ENNR & MAS & Raven SPM & GBL/Directed Reverie Therapy/Outpatient Treatment/neurosis/Psychosomatic Disorder/Katathymes Bilderleben/Therapieerfolgskontrolle/Ambulante Behandlung/Neurose/Psychosomatische Stoerungen/Psychotherapy and Psychotherapeutic Counseling/Psychotherapie und psychotherapeutische Beratung/Neurotic Disorder/Somatoform Disorder.

RefID:5058. Witt, K. and Hector, O.. [Reduction of the use of hypnotics by health education. Results from a controlled intervention study in a local community]. Ugeskrift for laeger 1991. 153 (49) 3460-3463. **Keywords:** Central/Denmark/Drug Utilization/Health Education [methods]/Hypnotics and Sedatives [administration & dosage] [adverse effects]/Intervention Studies/Newspapers/Radio/Sleep Disorders [drug therapy]/Humans.

Level 2, Not study design of interest

Non Randomized Controlled Trial, Controlled Before-After,

Interrupted Time Series

RefID:241. Wu, Elwin, El-Bassel, Nabila, Gilbert, Louisa, Chang, Mingway, and Sanders, Glorice. Effects of receiving additional off-site services on abstinence from illicit drug use among men on methadone: a longitudinal study. Evaluation and program planning 2010. 33 (4) 403-409. **Keywords:** Adult/*Health Services/ut [Utilization]/Humans/Interviews as Topic/Longitudinal Studies/Male/*Methadone/tu [Therapeutic Use]/Middle Aged/New York City/Opioid-Related Disorders/*Program Evaluation/mt [Methods]/*Street Drugs.

RefID:300. Berman, Anne H., Forsberg, Lars, Durbeej, Natalie, Kallmen, Hakan, and Hermansson, Ulric. Single-session motivational interviewing for drug detoxification inpatients: effects on self-efficacy, stages of change and substance use. Substance use & misuse 2010. 45 (3) 384-402. **Keywords:** Adult/Female/Humans/Inpatients/*Interview,Psychological/Male/Models,Psychological/*Motivation/*Psychotherapy,Brief/mt [Methods]/*Self Efficacy/*Substance-Related Disorders/th [Therapy]/Treatment Outcome.

RefID:337. Allahverdipour, Hamid, Bazargan, Mohsen, Farhadinasab, Abdollah, Hidarnia, Alireza, and Bashirian, Saeed. Effectiveness of skill-based substance abuse intervention among male adolescents in an Islamic country: case of the Islamic Republic of Iran. Journal of drug education 2009. 39 (2) 211-222. **Keywords:** Adolescent/*Adolescent Behavior/*Health Knowledge,Attitudes,Practice/Humans/Iran/*Islam/Longitudinal Studies/Male/Primary Prevention/mt [Methods]/*Smoking/pc [Prevention & Control]/*Substance-Related Disorders/pc [Prevention & Control].

RefID:384. Wiggins, Meg, Bonell, Chris, Sawtell, Mary, Austerberry, Helen, Burchett, Helen, Allen, Elizabeth, and Strange, Vicki. Health outcomes of youth development programme in England: prospective matched comparison study. BMJ (Clinical research ed.) 2009. 339 (#Issue#) b2534-#End Page#. **Keywords:** Adolescent/*Adolescent Health Services/og [Organization & Administration]/England/Female/Humans/Male/Pregnancy/Pregnancy in Adolescence/pc [Prevention & Control]/Prognosis/Program Evaluation/Prospective Studies/Socioeconomic Factors/Substance-Related Disorders/pc [Prevention & Control]/Unsafe Sex/pc [Prevention & Control]/Vulnerable Populations.

RefID:483. Kirby, Kimberly C., Kerwin, Marylouise E., Carpenedo, Carolyn M., Rosenwasser, Beth J., and Gardner, Robert S.. Interdependent group contingency management for cocaine-dependent methadone maintenance patients. Journal of applied behavior analysis 2008. 41 (4) 579-595. **Keywords:** Adult/Behavior Therapy/mt [Methods]/Choice Behavior/Cocaine-Related Disorders/px [Psychology]/*Cocaine-Related Disorders/rh [Rehabilitation]/Community Mental Health Services/Feasibility Studies/Female/Humans/Male/*Methadone/tu [Therapeutic Use]/Middle Aged/Motivation/*Narcotics/tu [Therapeutic Use]/Outcome and Process Assessment (Health Care)/Patient Compliance/px [Psychology]/*Psychotherapy,Group/mt [Methods]/Reinforcement Schedule/Substance Abuse Detection/*Token Economy.

RefID:951. Gray, Emily, McCambridge, Jim, and Strang, John. The effectiveness of motivational interviewing delivered by youth workers in reducing drinking, cigarette and cannabis smoking among young people: quasi-experimental pilot study. Alcohol and alcoholism (Oxford, Oxfordshire) 2005. 40 (6) 535-539. **Keywords:** Adolescent/Adult/*Alcohol Drinking/pc [Prevention & Control]/Female/Follow-Up Studies/Humans/*Interview,Psychological/London/Male/*Marijuana Abuse/pc [Prevention & Control]/*Motivation/*Nondirective Therapy/Outcome and Process Assessment (Health Care)/Pilot Projects/*Psychotherapy,Brief/*Smoking/pc [Prevention & Control]/Smoking/px [Psychology]/Student Health Services.

RefID:1070. Womack, Sharon, Compton, Wilson M., Dennis, Michael, McCormick, Scott, Fraser, James, Horton, Joseph C., Spitznagel, Edward L., and Cottler, Linda B.. Improving treatment services for substance abusers with comorbid depression. The American journal on addictions / American Academy of Psychiatrists in Alcoholism and Addictions 2004. 13 (3) 295-304. **Keywords:** Adult/*Case Management/*Depression/co [Complications]/*Depression/di [Diagnosis]/Depression/th [Therapy]/Diagnosis,Dual (Psychiatry)/Female/Humans/Intervention Studies/Male/Middle Aged/Referral and Consultation/Substance-Related Disorders/co [Complications]/*Substance-Related Disorders/th [Therapy]/Treatment Outcome.

RefID:1082. Hien, Denise A., Cohen, Lisa R., Miele, Gloria M., Litt, Lisa Caren, and Capstick, Carrie. Promising treatments for women with comorbid PTSD and substance use disorders. The American journal of psychiatry 2004. 161 (8) 1426-1432. **Keywords:** Adult/*Cognitive Therapy/mt [Methods]/Community Mental Health Services/mt [Methods]/Comorbidity/Diagnosis,Dual (Psychiatry)/Female/Humans/Poverty/Psychiatric Status Rating Scales/Recurrence/pc [Prevention & Control]/Sex Factors/Stress Disorders,Post-Traumatic/di [Diagnosis]/*Stress Disorders,Post-Traumatic/ep [Epidemiology]/*Stress Disorders,Post-Traumatic/th [Therapy]/Substance-Related Disorders/di [Diagnosis]/*Substance-Related Disorders/ep [Epidemiology]/*Substance-Related Disorders/th [Therapy]/Treatment Outcome/Urban Population.

RefID:1170. Hershberger, Scott L., Wood, Michele M., and Fisher, Dennis G.. A cognitive-behavioral intervention to reduce HIV risk behaviors in crack and injection drug users. AIDS and behavior 2003. 7 (3) 229-243. **Keywords:** Adult/*Cocaine-Related Disorders/px [Psychology]/*Cognitive Therapy/Condoms/*Crack Cocaine/Female/*HIV Infections/pc [Prevention & Control]/*HIV Infections/px [Psychology]/Humans/Male/*Risk-Taking/Sexual Behavior/*Substance Abuse,Intravenous/px [Psychology]/Treatment Outcome.

RefID:2182. Wiggins, M., Bonell, C., Sawtell, M., Austerberry, H., Burchett, H., Allen, E., and Strange, V.. Health outcomes of youth development programme in England: Prospective matched comparison study. BMJ (Clinical research ed.) 2009. 339 (7713) 148-151. **Keywords:** academic achievement/Adolescent/adolescent pregnancy/article/cannabis addiction/Child/*child welfare/clinical trial/controlled clinical trial/controlled study/drunkenness/Female/Health Education/*health program/heterosexual female/high risk

population/human/juvenile delinquency/major clinical study/Male/outcome assessment/parenthood/priority journal/school child/sexual education/sexual intercourse/substance abuse/United Kingdom.

RefID:2337. Soyez, V., Broekaert, E., and Rosseel, Y.. Social network involvement during therapeutic community treatment: Is there an impact on success?. Therapeutic Communities: the International Journal for Therapeutic and Supportive Organizations 2006. 27 (1) 45-67. **Keywords:** article/clinical trial/cohort analysis/controlled clinical trial/controlled study/Crisis Intervention/*drug dependence treatment/family counseling/Female/field study/follow up/hospital admission/human/interview/long term care/major clinical study/Male/multicenter study/Patient Participation/prediction/Regression Analysis/residential care/sex difference/social interaction/social network/Social Support/*Therapeutic Community/Treatment Outcome.

RefID:2428. Roll, J. M., Chermack, S. T., and Chudzynski, J. E.. Investigating the use of contingency management in the treatment of cocaine abuse among individuals with schizophrenia: A feasibility study. Psychiatry research 2004. 125 (1) 61-64. **Keywords:** Adult/article/case report/clinical trial/*cocaine dependence/th [Therapy]/controlled clinical trial/controlled study/*drug dependence treatment/drug withdrawal/feasibility study/human/Male/priority journal/psychosocial care/*reinforcement/*Schizophrenia/Social Work/substance abuse/Treatment Outcome/*Cocaine.

RefID:2534. Jelovac, N., Milas, M., and Golik-Gruber, V.. Naltrexone is efficient in maintaining heroin abstinence of selected groups of addicts. Alcoholism 2000. 36 (1) 73-77. **Keywords:** article/clinical article/clinical trial/controlled clinical trial/controlled study/drug activity/drug detoxification/drug indication/drug mechanism/drug withdrawal/*Heroin Dependence/dt [Drug Therapy]/*Heroin Dependence/th [Therapy]/human/Patient Selection/Psychotherapy/superego/Treatment Outcome/*naltrexone/dt [Drug Therapy].

RefID:2947. Singh, Chanpreet K.. Role of vocational counseling and education in work attitudes and job placement of recovering substance abusers. Dissertation Abstracts International Section A: Humanities and Social Sciences 2009. 69 (12-A) 4644-#End Page#. **Keywords:** *drug abuse/*Occupational Guidance/*Occupations/*Vocational Counselors/*Vocational Education/employment status/Higher Education/vocational rehabilitation.

RefID:3137. O'Farrell, Timothy J., Murphy, Marie, Alter, Jane, and Fals-Stewart, William. Brief family treatment intervention to promote aftercare among male substance abusing patients in inpatient detoxification: A quasi-experimental pilot study. Addictive behaviors 2007. 32 (8) 1681-1691. **Keywords:** *Aftercare/*detoxification/*Drug Rehabilitation/*Family Therapy/*Intervention/Family Members.

RefID:3169. Ruglass, Lesia M.. Ethnocultural differences in therapeutic alliance and outcome for women with comorbid posttraumatic stress disorder and substance use disorder. Dissertation Abstracts International: Section B: The Sciences and Engineering 2006. 66 (8-B) 4499-#End

Page#. **Keywords:** *comorbidity/*drug abuse/*Posttraumatic Stress Disorder/*Therapeutic Alliance/*Treatment Outcomes/Human Females/Racial and Ethnic Differences.

RefID:3298. Caldeira, Nathilee A.. Dissociation and treatment outcome in urban women with comorbid PTSD and substance use disorders. Dissertation Abstracts International: Section B: The Sciences and Engineering 2004. 65 (3-B) 1540-#End Page#. **Keywords:** *Cognitive Behavior Therapy/*drug abuse/*Posttraumatic Stress Disorder/*Therapeutic Alliance/Mental Disorders/Treatment Outcomes.

RefID:3551. Kirby, Kris N., Petry, Nancy M., and Bickel, Warren K.. Heroin addicts have higher discount rates for delayed rewards than non-drug-using controls. Journal of Experimental Psychology: General 1999. 128 (1) 78-87. **Keywords:** *Choice Behavior/*Delay of Gratification/*Heroin Addiction/*Impulsiveness/*Rewards/Monetary Incentives.

RefID:4523. Piper, W. E., Debbane, E. G., Bienvenu, J. P., and Garant, J.. A comparative study of four forms of psychotherapy. Journal of consulting and clinical psychology 1984. 52 (2) 268-279. **Keywords:** Central/Adolescent/Cost-Benefit Analysis/Mental Disorders [therapy]/Psychotherapy [methods]/Psychotherapy,Brief [methods]/Psychotherapy,Group [methods]/Adult/Female/Humans/Male/Middle Aged.

RefID:4553. Budman, S. H., Demby, A., Feldstein, M., and Gold, M.. The effects of time-limited group psychotherapy: a controlled study. International Journal of Group Psychotherapy 1984. 34 (4) 587-603. **Keywords:** Central/Adult/Follow Up Studies/Neurotic Disorder Therapy/Personality Disorder Therapy/Psychotherapy,Brief Methods/Psychotherapy,Group Methods.

RefID:4719. Tansella, M., Zimmermann-Tansella, C., and Lader, M.. The residual effects of N-desmethyldiazepam in patients. Psychopharmacologia 1974. 38 (1) 81-90. **Keywords:** Central/Adolescent/Amobarbital [therapeutic use]/Anxiety Disorders [complications] [drug therapy]/Clinical Trials as Topic/Dealkylation/Diazepam [adverse effects] [therapeutic use]/Placebos/Psychiatric Status Rating Scales/Psychological Tests/Self Concept/Sleep Disorders [drug therapy] [etiology]/Time Factors/Adult/Child/Female/Humans/Male/Middle Aged.

RefID:4726. Solyom, L., Heseltine, G. F., McClure, D. J., Solyom, C., Ledwidge, B., and Steinberg, G.. Behaviour therapy versus drug therapy in the treatment of phobic neurosis. Canadian Psychiatric Association journal 1973. 18 (1) 25-32. **Keywords:** Central/Anxiety/Aversive Therapy/Behavior Therapy/Electroshock/Evaluation Studies as Topic/Personality Inventory/Phenelzine [therapeutic use]/Phobic Disorders [diagnosis] [drug therapy] [therapy]/Placebos/Psychotherapy,Brief/Recurrence/Self Concept/Adult/Female/Humans/Male.

RefID:5209. Daley, D. C., Salloum, I. M., Zuckoff, A., Kirisci, L., and Thase, M. E.. Increasing treatment adherence among outpatients with depression and cocaine dependence: Results of a

pilot study. American Journal of Psychiatry 1998. 155 (11) 1611-1613. **Keywords:** Central/Depression /Th [Therapy]/Drug Dependence /Th [Therapy]/Psychotherapy/Patient Compliance/outpatient care/Motivation/Hospitalization/patient counseling/Treatment Outcome/human/Male/Female/clinical article/controlled study/Adult/article/priority journal/Cocaine/antidepressant agent.

RefID:5844. Johnson, J. D.. The effects of a brief cognitive-behavioral group intervention on the depression and hopelessness of drug dependent, human immunodeficiency virus-positive, African-American women (immune deficiency). Dissertation Abstracts International 2001. 62 (1-B) 551-#End Page#. **Keywords:** Central.

RefID:7664. Allahverdipour, H., Bazargan, M., Farhadinasab, A., Hidarnia, A., and Bashirian, S.. Effectiveness of skill-based substance abuse intervention among male adolescents in an Islamic country: case of the Islamic Republic of Iran. Journal of drug education 2009. 39 (2) 211-222. **Keywords:** CINAHL/Crisis Intervention -- In Adolescence -- Iran/substance abuse/adolescence/Attitude/chi square test/Coefficient Alpha/Data Analysis Software/Funding Source/Iran/Knowledge/Male/Mann-Whitney U Test/One-Way Analysis of Variance/Panel Studies/Peer Group/Prospective Studies/Quasi-Experimental Studies/Role Models/Self-Efficacy/T-Tests/Wilcoxon Rank Sum Test/human.

RefID:7793. Liddle, H. A., Dakof, G. A., Turner, R. M., Henderson, C. E., and Greenbaum, P. E.. Treating adolescent drug abuse: a randomized trial comparing multidimensional family therapy and cognitive behavior therapy. Addiction (Abingdon, England) 2008. 103 (10) 1660-1670. **Keywords:** CINAHL/Cognitive Therapy -- In Adolescence/Family Therapy -- In Adolescence/Substance Abuse -- Therapy -- In Adolescence/adolescence/Adult/Analysis of Variance/Behavior Rating Scales/chi square test/Child/Clinical Trials/Coding/Descriptive Statistics/Female/Interview Guides/Interviews/Male/Maximum Likelihood/Middle Age/New England/Pretest-Posttest Design/Prospective Studies/Psychological Tests/Repeated Measures/Retrospective Design/Self Report/Substance Dependence -- Therapy/Treatment Outcomes/videorecording/human.

RefID:8081. Patterson, D. A.. Motivational interviewing: does it increase clients' retention in intensive outpatient treatment?. Substance Abuse 2008. 29 (1) 17-23. **Keywords:** CINAHL/Motivational Interviewing -- Methods/Outpatient Service/Patient Compliance/Substance Dependence -- Rehabilitation/Adult/Analysis of Variance/Bivariate Statistics/Blacks/chi square test/Descriptive Statistics/effect size/Female/Funding Source/Hispanics/Male/Middle Age/Multivariate Analysis/Native Americans/Pretest-Posttest Control Group Design/Professional-Client Relations/Program Development/Psychological Tests/Psychotherapist Attitudes/Random Assignment/Regression/Research Priorities/Severity of Illness Indices/T-Tests/Treatment Outcomes/Two-Tailed Test/Whites/human.

RefID:8209. Domino, M. E., Morrissey, J. P., Chung, S., and Nadlicki, T.. Changes in service use during a trauma-informed intervention for women. Women & health 2006. 44 (3) 105-122. **Keywords:** CINAHL/Battered Women/Patient Compliance/Stress Disorders,Post-Traumatic --

Rehabilitation/Women's Health/Adult/Comorbidity/Descriptive Statistics/Female/Focus Groups/Funding Source/Mental Health/Middle Age/multiple regression/Multivariate Analysis/outcome assessment/P-Value/Process Assessment (Health Care)/Questionnaires/Stress Disorders,Post-Traumatic -- Epidemiology/Substance Use Disorders -- Epidemiology/United States/human.

RefID:8449. Cohen, L. R. and Hien, D. A.. Treatment outcomes for women with substance abuse and PTSD who have experienced complex trauma. Psychiatric Services 2006. 57 (1) 100-106. **Keywords:** CINAHL/Cognitive Therapy/Stress Disorders,Post-Traumatic -- Diagnosis/Stress Disorders,Post-Traumatic -- Therapy/Substance Use Disorders -- Therapy/Adult/Analysis of Variance/Behavior,Addictive/Comorbidity/Descriptive Statistics/Female/Funding Source/Hamilton Rating Scale for Depression/Middle Age/Psychological Tests/Scales/Socioeconomic Factors/Unstructured Interview/Urban Areas/human.

RefID:9268. Heckathorn, D. D., Semaan, S., Broadhead, R., and Hughes, J. J.. Extensions of respondent-driven sampling: a new approach to the study of injection drug users aged 18-25. AIDS & Behavior 2002. 6 (1) 55-67. **Keywords:** CINAHL/Intravenous Drug Users -- Connecticut/Sampling Methods -- Evaluation/Research Subject Recruitment -- Methods/Descriptive Statistics/chi square test/Age Factors/Interviews/Whites/Blacks/Hispanics/Volunteer Workers/Bias (Research)/Connecticut/Interpersonal Relations/adolescence/Adult/Epidemiological Research/Funding Source/human.

RefID:9320. Harrington, N. G., Giles, S. M., Hoyle, R. H., Feeney, G. J., and Yungbluth, S. C.. Evaluation of the All Stars character education and problem behavior prevention program: effects on mediator and outcome variables for middle school students. Health Education & Behavior 2001. 28 (5) 533-#End Page#. **Keywords:** CINAHL/Adolescent Behavior/Character -- Education -- In Adolescence/School Health Education -- In Adolescence/Prospective Studies/Pretest-Posttest Design/Midwestern United States/Descriptive Statistics/Questionnaires/Observational Methods/Analysis of Variance/Coefficient Alpha/multiple regression/correlation coefficient/Violence/sexuality/substance abuse/convenience sample/Repeated Measures/Education,Continuing (Credit)/School Health Education -- Evaluation/adolescence/Funding Source/human.

RefID:9362. Rotheram-Borus, M. J., Lee, M. B., Murphy, D. A., Futterman, D., Duan, N., Birnbaum, J. M., and Lightfoot, M.. Efficacy of a preventive intervention for youths living with HIV. American journal of public health 2001. 91 (3) 400-405. **Keywords:** CINAHL/HIV Infections -- Prevention and Control -- In Adolescence/HIV Infections -- Transmission/Health Behavior -- In Adolescence/Behavioral Changes -- Evaluation/HIV Education -- Evaluation/Funding Source/Intervention Trials/Interviews/Coping/Risk Taking Behavior -- Prevention and Control/Quality Assurance/Data Collection,Computer Assisted/Health Status Indicators/Brief Symptom Inventory/Psychological Tests/Coefficient Alpha/substance abuse/analysis of covariance/Logistic Regression/Descriptive Statistics/adolescence/Adult/Male/Female/P-Value/Evaluation Research/Program Evaluation/Sexuality -- In Adolescence/human.

RefID:10698. Slater, Michael D., Kelly, Kathleen J., Edwards, Ruth W., Thurman, Pamela J., Plested, Barbara A., Keefe, Thomas J., Lawrence, Frank R., and Henry, Kimberly L.. Combining In-School and Community-Based Media Efforts: Reducing Marijuana and Alcohol Uptake among Younger Adolescents. Health education research 2006. 21 (1) 157-167. **Keywords:** Marijuana/prevention/Marketing/Mass Media Effects/drug abuse/drinking/Early Adolescents/Community Programs/Health Education/School Community Relationship/Middle School Students/Comparative Analysis/Longitudinal Studies/Smoking/Program Effectiveness/ERIC RCTS.

RefID:10718. Dent, Clyde W., Sussman, Steve, Hennesy, Michael, Galaif, Elisha R., Stacy, Alan W., Moss, Maryann, and Craig, Sande. Implementation and Process Evaluation of a School-Based Drug Abuse Prevention Program: Project Towards No Drug Abuse. Journal of drug education 1998. 28 (4) 361-375. **Keywords:** drug abuse/High Risk Students/High School Students/High Schools/Outcomes of Education/prevention/Program Evaluation/ERIC RCTS.

RefID:10790. Bond, L., Patton, G., Glover, S., Carlin, J. B., Butler, H., Thomas, L., and Bowes, G.. The Gatehouse Project: can a multilevel school intervention affect emotional wellbeing and health risk behaviours?. J Epidemiol.Community Health 2004. 58 (12) 997-1003. **Keywords:** Adolescent/Adolescent Behavior/Female/Health Behavior/Health Promotion/Methods/Humans/Male/Mental Health/Risk-Taking/School Health Services/organization & administration/Social Environment/Substance-Related Disorders/prevention & control/Teaching Materials/Victoria/from SR bibliographies.

RefID:10795. Piper, D. L., Moberg, D. P., and King, M. J.. The healthy for life project: Behavioral outcomes. The journal of primary prevention 2000. 21 (1) 47-73. **Keywords:** from SR bibliographies.

Protocol

RefID:92. Bogenschutz, Michael P., Donovan, Dennis M., Adinoff, Bryon, Crandall, Cameron, Forcehimes, Alyssa A., Lindblad, Robert, Mandler, Raul N., Oden, Neal L., Perl, Harold I., and Walker, Robrina. Design of NIDA CTN Protocol 0047: screening, motivational assessment, referral, and treatment in emergency departments (SMART-ED). The American journal of drug and alcohol abuse 2011. 37 (5) 417-425. **Keywords:** Data Interpretation,Statistical/*Emergency Service,Hospital/og [Organization & Administration]/Humans/*Mass Screening/mt [Methods]/Motivation/National Institute on Drug Abuse (U.S.)/Prospective Studies/Psychotherapy,Brief/mt [Methods]/*Research Design/*Substance-Related Disorders/di [Diagnosis]/Substance-Related Disorders/th [Therapy]/United States.

RefID:122. Vicens, Caterina, Socias, Isabel, Mateu, Catalina, Leiva, Alfonso, Bejarano, Ferran, Sempere, Ermengol, Basora, Josep, Palop, Vicente, Mengual, Marta, Beltran, Jose Luis,

Aragones, Enric, Lera, Guillem, Folch, Silvia, Pinol, Josep Lluis, Esteva, Magdalena, Roca, Miguel, Arenas, Arturo, Del Mar Sureda, Maria, Campoamor, Francisco, and Fiol, Francisca. Comparative efficacy of two primary care interventions to assist withdrawal from long term benzodiazepine use: a protocol for a clustered, randomized clinical trial. BMC family practice 2011. 12 (#Issue#) 23-#End Page#. **Keywords:** Adolescent/Adult/Aged/Aged,80 and over/Anxiety/ep [Epidemiology]/*Benzodiazepines/ae [Adverse Effects]/Depression/ep [Epidemiology]/Humans/Interviews as Topic/Middle Aged/*Patient Education as Topic/*Primary Health Care/mt [Methods]/Research Design/Single-Blind Method/*Substance Withdrawal Syndrome/pc [Prevention & Control]/*Substance-Related Disorders/th [Therapy]/Young Adult.

RefID:711. Whitford, David L. and Chan, Wai Sun. A randomised controlled trial of a lengthened and multi-disciplinary consultation model in a socially deprived community: a study protocol. BMC family practice 2007. 8 (#Issue#) 38-#End Page#. **Keywords:** Anxiety/ep [Epidemiology]/Cultural Deprivation/Depression/ep [Epidemiology]/*Family Practice/og [Organization & Administration]/Humans/Ireland/Models,Organizational/Mothers/px [Psychology]/*Patient Care Team/og [Organization & Administration]/*Poverty Areas/*Primary Health Care/og [Organization & Administration]/Program Evaluation/*Referral and Consultation/og [Organization & Administration]/Residence Characteristics/Social Problems/Time Factors/Vulnerable Populations.

RefID:1039. Feaster, Daniel J., Robbins, Michael S., Horigian, Viviana, and Szapocznik, Jose. Statistical issues in multisite effectiveness trials: the case of brief strategic family therapy for adolescent drug abuse treatment. Clinical trials (London, England) 2004. 1 (5) 428-439. **Keywords:** Adolescent/*Adolescent Health Services/*Community Health Centers/Data Interpretation,Statistical/*Family Therapy/Humans/Models,Statistical/*Multicenter Studies as Topic/mt [Methods]/Patient Selection/Randomized Controlled Trials as Topic/mt [Methods]/Sample Size/*Substance-Related Disorders/th [Therapy]/*Treatment Outcome.

RefID:3750. Robbins, M. S., Alonso, E., Horigian, V. E., Bachrach, K., Burlew, K., Carrion, I. S., Hodgkins, C. C., Miller, M., Schindler, E., VanDeMark, N., Henderson, C., and Szapocznik, J.. Transporting clinical research to community settings: designing and conducting a multisite trial of brief strategic family therapy. Addiction Science & Clinical Practice 2010. 5 (2) 54-61. **Keywords:** Multifile Reviews/Adolescent/Community Health Centers/og [Organization & Administration]/Evidence-Based Medicine/Family Therapy/mt [Methods]/Health Personnel/Health Services Research/Humans/Multicenter Studies as Topic/Program Development/Psychotherapy/Brief/mt [Methods]/Research Design/Substance Abuse Treatment Centers/Substance-Related Disorders/di [Diagnosis]/Substance-Related Disorders/px [Psychology]/Substance-Related Disorders/rh [Rehabilitation].

RefID:3752. Bogenschutz, M. P., Donovan, D. M., Adinoff, B., Crandall, C., Forcehimes, A. A., Lindblad, R., Mandler, R. N., Oden, N. L., Perl, H. I., and Walker, R.. Design of NIDA CTN Protocol 0047: screening, motivational assessment, referral, and treatment in emergency departments (SMART-ED). American Journal of Drug & Alcohol Abuse 2011. 37 (5) 417-425. **Keywords:**

Multifile Reviews/Data Interpretation/Statistical/Emergency Service/Hospital/og [Organization & Administration]/Humans/Mass Screening/mt [Methods]/Motivation/National Institute on Drug Abuse (U.S.)/Prospective Studies/Psychotherapy/Brief/mt [Methods]/Research Design/Substance-Related Disorders/di [Diagnosis]/Substance-Related Disorders/th [Therapy]/United States.

RefID:4160. Ferri, Marica, Burkhart, Gregor, Allara, Elias, Bo, Alessandra, Gyarmathy, Anna, V, Faggiano, Fabrizio, and Cochrane Database of Systematic Reviews. Media Campaigns for the prevention of illicit drug use in young people. #journal name# 2011. #volume# (9) #Start Page#-#End Page#. **Keywords:** CDSR.

RefID:5702. Shift, W., Copeland, J., Howard, J., Roffman, R. A., Stephens, R. S., and Berghuis, J.. Adolescent cannabis check-up and intervention trial. Drug and alcohol dependence 2001. 63 Suppl 1 (#Issue#) 156-#End Page#. **Keywords:** Central.

Systematic review/meta-analysis

RefID:100. Wong, Suzanne, Ordean, Alice, Kahan, Meldon, and Society of Obstetricians and Gynecologists of Canada. SOGC clinical practice guidelines: Substance use in pregnancy: no. 256, April 2011. International journal of gynaecology and obstetrics: the official organ of the International Federation of Gynaecology and Obstetrics 2011. 114 (2) 190-202. **Keywords:** Canada/Female/Health Services Accessibility/Humans/Infant Mortality/Infant,Newborn/Maternal Health Services/ec [Economics]/Maternal Mortality/Pregnancy/*Pregnancy Complications/di [Diagnosis]/*Pregnancy Complications/th [Therapy]/Randomized Controlled Trials as Topic/*Substance-Related Disorders/di [Diagnosis]/*Substance-Related Disorders/th [Therapy].

RefID:135. Wong, Suzanne, Ordean, Alice, Kahan, Meldon, Maternal Fetal Medicine Committee, Family Physicians Advisory Committee, Medico-Legal Committee, and Society of Obstetricians and Gynaecologists of Canada. Substance use in pregnancy. Journal of obstetrics and gynaecology Canada : JOGC = Journal d'obstetrique et gynecologie du Canada : JOGC 2011. 33 (4) 367-384. **Keywords:** Breast Feeding/Female/Humans/Infant,Newborn/Maternal Health Services/Neonatal Abstinence Syndrome/di [Diagnosis]/Neonatal Abstinence Syndrome/th [Therapy]/Pain/dt [Drug Therapy]/Pregnancy/*Pregnancy Complications/di [Diagnosis]/*Pregnancy Complications/th [Therapy]/Professional-Patient Relations/Smoking Cessation/mt [Methods]/Substance Abuse Detection/Substance Withdrawal Syndrome/th [Therapy]/*Substance-Related Disorders/di [Diagnosis]/*Substance-Related Disorders/th [Therapy].

RefID:219. Petry, Nancy M. and Alessi, Sheila M.. Prize-based contingency management is efficacious in cocaine-abusing patients with and without recent gambling participation. Journal of substance abuse treatment 2010. 39 (3) 282-288. **Keywords:** Adult/Cocaine-Related

Disorders/co [Complications]/*Cocaine-Related Disorders/rh [Rehabilitation]/Female/Gambling/co [Complications]/*Gambling/px [Psychology]/Humans/Male/Middle Aged/Randomized Controlled Trials as Topic/Retrospective Studies/*Reward/Treatment Outcome.

RefID:247. Weinstock, Jeremiah, Rash, Carla J., and Petry, Nancy M.. Contingency management for cocaine use in methadone maintenance patients: when does abstinence happen?. Psychology of addictive behaviors : journal of the Society of Psychologists in Addictive Behaviors 2010. 24 (2) 282-291. **Keywords:** Adult/Analysis of Variance/*Behavior Therapy/mt [Methods]/Chi-Square Distribution/*Cocaine-Related Disorders/th [Therapy]/Female/Humans/Male/*Methadone/tu [Therapeutic Use]/Narcotics/tu [Therapeutic Use]/*Opioid-Related Disorders/rh [Rehabilitation]/*Substance Abuse Detection/Treatment Outcome.

RefID:377. Baker, Amanda, Turner, Alyna, Kay-Lambkin, Frances J., and Lewin, Terry J.. The long and the short of treatments for alcohol or cannabis misuse among people with severe mental disorders. Addictive behaviors 2009. 34 (10) 852-858. **Keywords:** Adult/Alcohol Drinking/ep [Epidemiology]/*Alcohol Drinking/th [Therapy]/Alcohol-Related Disorders/ep [Epidemiology]/*Alcohol-Related Disorders/th [Therapy]/Cognitive Therapy/Comorbidity/Diagnosis,Dual (Psychiatry)/Female/Humans/Male/Marijuana Abuse/ep [Epidemiology]/*Marijuana Abuse/th [Therapy]/Mental Disorders/ep [Epidemiology]/*Mental Disorders/th [Therapy]/Middle Aged/Motivation/New South Wales/ep [Epidemiology]/Treatment Outcome/Young Adult.

RefID:389. Magill, Molly and Ray, Lara A.. Cognitive-behavioral treatment with adult alcohol and illicit drug users: a meta-analysis of randomized controlled trials. Journal of studies on alcohol and drugs 2009. 70 (4) 516-527. **Keywords:** Adult/*Alcoholism/th [Therapy]/Cognitive Therapy/sn [Statistics & Numerical Data]/*Cognitive Therapy/Female/Humans/Male/Randomized Controlled Trials as Topic/Sex Characteristics/*Substance-Related Disorders/th [Therapy]/Time Factors/Treatment Outcome.

RefID:446. Field, Craig A., Adinoff, Bryon, Harris, T. Robert, Ball, Samuel A., and Carroll, Kathleen M.. Construct, concurrent and predictive validity of the URICA: data from two multi-site clinical trials. Drug and alcohol dependence 2009. 101 (1-2) 115-123. **Keywords:** Adult/*Alcoholism/px [Psychology]/Alcoholism/rh [Rehabilitation]/Female/Humans/Male/Motivation/Predictive Value of Tests/Prognosis/*Psychological Tests/st [Standards]/Psychotherapy/Reproducibility of Results/*Substance-Related Disorders/px [Psychology]/Substance-Related Disorders/rh [Rehabilitation]/Treatment Outcome.

RefID:535. Rash, Carla J., Alessi, Sheila M., and Petry, Nancy M.. Cocaine abusers with and without alcohol dependence respond equally well to contingency management treatments. Experimental and clinical psychopharmacology 2008. 16 (4) 275-281. **Keywords:** Adult/Alcoholics Anonymous/Alcoholism/ep [Epidemiology]/Alcoholism/px [Psychology]/*Alcoholism/rh [Rehabilitation]/*Behavior Therapy/mt [Methods]/Cocaine-

Related Disorders/ep [Epidemiology]/Cocaine-Related Disorders/px [Psychology]/*Cocaine-Related Disorders/rh [Rehabilitation]/Combined Modality Therapy/Comorbidity/Female/Follow-Up Studies/Humans/Male/Middle Aged/Patient Education as Topic/Psychotherapy,Group/Randomized Controlled Trials as Topic/Recurrence/pc [Prevention & Control]/Reinforcement (Psychology)/Substance Abuse Detection/*Token Economy/Treatment Outcome.

RefID:561. Weinstock, Jeremiah, Barry, Danielle, and Petry, Nancy M.. Exercise-related activities are associated with positive outcome in contingency management treatment for substance use disorders. Addictive behaviors 2008. 33 (8) 1072-1075. **Keywords:** Adult/*Exercise Therapy/mt [Methods]/Female/Humans/Male/Motivation/Patient Compliance/Reinforcement Schedule/Substance Abuse Treatment Centers/Substance-Related Disorders/rh [Rehabilitation]/*Substance-Related Disorders/th [Therapy]/Treatment Outcome.

RefID:607. Dutra, Lissa, Stathopoulou, Georgia, Basden, Shawnee L., Leyro, Teresa M., Powers, Mark B., and Otto, Michael W.. A meta-analytic review of psychosocial interventions for substance use disorders. The American journal of psychiatry 2008. 165 (2) 179-187. **Keywords:** Adolescent/Adult/Behavior Therapy/mt [Methods]/Cocaine-Related Disorders/px [Psychology]/Cocaine-Related Disorders/th [Therapy]/Effect Modifier,Epidemiologic/Female/Humans/Male/Marijuana Abuse/px [Psychology]/Marijuana Abuse/th [Therapy]/Opioid-Related Disorders/px [Psychology]/Opioid-Related Disorders/th [Therapy]/Patient Dropouts/sn [Statistics & Numerical Data]/*Psychotherapy/mt [Methods]/Psychotherapy/sn [Statistics & Numerical Data]/Publication Bias/Randomized Controlled Trials as Topic/sn [Statistics & Numerical Data]/Recurrence/pc [Prevention & Control]/Substance-Related Disorders/px [Psychology]/*Substance-Related Disorders/th [Therapy]/Treatment Outcome.

RefID:669. Schumacher, Joseph E., Milby, Jesse B., Wallace, Dennis, Meehan, Dawna Cricket, Kertesz, Stefan, Vuchinich, Rudy, Dunning, Jonathan, and Usdan, Stuart. Meta-analysis of day treatment and contingency-management dismantling research: Birmingham Homeless Cocaine Studies (1990-2006). Journal of consulting and clinical psychology 2007. 75 (5) 823-828. **Keywords:** Alabama/Cocaine-Related Disorders/px [Psychology]/*Cocaine-Related Disorders/rh [Rehabilitation]/Combined Modality Therapy/*Crack Cocaine/*Day Care/*Homeless Persons/px [Psychology]/Humans/Randomized Controlled Trials as Topic/*Token Economy.

RefID:2128. Goldstein, B. I. and Bukstein, O. G.. Comorbid substance use disorders among youth with bipolar disorder: Opportunities for early identification and prevention. Journal of Clinical Psychiatry 2010. 71 (3) 348-358. **Keywords:** *adolescent disease/dt [Drug Therapy]/Alcoholism/dt [Drug Therapy]/article/*Bipolar Disorder/dt [Drug Therapy]/cannabis addiction/*childhood disease/dt [Drug Therapy]/clinical trial/Cognitive Therapy/Comorbidity/conduct disorder/family history/human/lithium blood level/major depression/Medline/posttraumatic stress disorder/Pregnancy/priority journal/sexual abuse/*substance abuse/Suicide/suicide attempt/systematic review/tobacco dependence/aripiprazole/ct [Clinical Trial]/aripiprazole/dt [Drug Therapy]/atypical

antipsychotic agent/dt [Drug Therapy]/lamotrigine/ct [Clinical Trial]/lamotrigine/dt [Drug Therapy]/lithium carbonate/cr [Drug Concentration]/lithium carbonate/dt [Drug Therapy]/placebo/psychotropic agent/quetiapine/ct [Clinical Trial]/quetiapine/cb [Drug Combination]/quetiapine/dt [Drug Therapy]/topiramate/ct [Clinical Trial]/topiramate/cb [Drug Combination]/topiramate/dt [Drug Therapy]/valproic acid/ct [Clinical Trial]/valproic acid/dt [Drug Therapy].

RefID:2236. Becker, S. J. and Curry, J. F.. Outpatient Interventions for Adolescent Substance Abuse: A Quality of Evidence Review. Journal of consulting and clinical psychology 2008. 76 (4) 531-543. **Keywords:** *addiction/th [Therapy]/Adolescent/article/clinical trial/Cognitive Therapy/evidence based medicine/Family Therapy/human/intervention study/Motivation/*substance abuse.

RefID:2976. Vuchinich, Rudy, Wallace, Dennis, Milby, Jesse B., Schumacher, Joseph E., Mennemeyer, Stephen, and Kertesz, Stefan. Relations between in-treatment and follow-up abstinence among cocaine-dependent homeless persons in three clinical trials. Experimental and clinical psychopharmacology 2009. 17 (3) 165-172. **Keywords:** *contingency management/*Drug Abstinence/*Drug Dependency/*Drug Rehabilitation/*Homeless/Cocaine/Relapse (Disorders).

RefID:3295. Hanlon, Joseph T., Lindblad, Catherine I., and Gray, Shelly L.. Can Clinical Pharmacy Services Have a Positive Impact on Drug-Related Problems and Health Outcomes in Community-Based Older Adults?. American Journal of Geriatric Pharmacotherapy (AJGP) 2004. 2 (1) 3-13. **Keywords:** *aging/*Drug Therapy/*Health Care Services/*Side Effects (Drug)/*Treatment Outcomes.

RefID:3341. Copeland, Jan and Martin, Greg. Web-based interventions for substance use disorders: A qualitative review. Journal of substance abuse treatment 2004. 26 (2) 109-116. **Keywords:** *Alcohol Rehabilitation/*Drug Rehabilitation/*Internet/*Intervention/*Treatment Effectiveness Evaluation.

RefID:3718. Jackson, C., Geddes, R., Haw, S., and Frank, J.. Interventions to prevent substance use and risky sexual behaviour in young people: a systematic review. Addiction (Abingdon, England) 2012. 107 (4) 733-747. **Keywords:** Multifile Reviews.

RefID:3722. Hedrich, D., Alves, P., Farrell, M., Stover, H., Moller, L., and Mayet, S.. The effectiveness of opioid maintenance treatment in prison settings: a systematic review. Addiction (Abingdon, England) 2012. 107 (3) 501-517. **Keywords:** Multifile Reviews.

RefID:3725. MacLean, S., Cameron, J., Harney, A., and Lee, N. K.. Psychosocial therapeutic interventions for volatile substance use: a systematic review. Addiction (Abingdon, England) 2012. 107 (2) 278-288. **Keywords:** Multifile Reviews.

RefID:3731. Finfgeld-Connett, D. and Johnson, E. D.. Substance abuse treatment for women

who are under correctional supervision in the community: a systematic review of qualitative findings. Issues in Mental Health Nursing 2011. 32 (10) 640-648. **Keywords:** Multifile Reviews.

RefID:3758. Amato, L., Minozzi, S., Davoli, M., and Vecchi, S.. Psychosocial combined with agonist maintenance treatments versus agonist maintenance treatments alone for treatment of opioid dependence. [Review][Update of Cochrane Database Syst Rev. 2008;(4):CD004147; PMID: 18843654]. Cochrane Database of Systematic Reviews 2011. #volume# (10) CD004147-#End Page#. **Keywords:** Multifile Reviews/Combined Modality Therapy/mt [Methods]/Humans/Narcotics/tu [Therapeutic Use]/Opioid-Related Disorders/px [Psychology]/Opioid-Related Disorders/rh [Rehabilitation]/Psychotherapy/mt [Methods]/Randomized Controlled Trials as Topic.

RefID:3790. Wong, S., Ordean, A., Kahan, M., Maternal Fetal Medicine Committee, Family Physicians Advisory Committee, Medico-Legal Committee, and Society of Obstetricians and Gynaecologists of Canada.. Substance use in pregnancy. Journal of Obstetrics & Gynaecology Canada: JOGC 2011. 33 (4) 367-384. **Keywords:** Multifile Reviews/Breast Feeding/Female/Humans/Infant/newborn/Maternal Health Services/Neonatal Abstinence Syndrome/di [Diagnosis]/Neonatal Abstinence Syndrome/th [Therapy]/Pain/dt [Drug Therapy]/Pregnancy/Pregnancy Complications/di [Diagnosis]/Pregnancy Complications/th [Therapy]/Professional-Patient Relations/Smoking Cessation/mt [Methods]/Substance Abuse Detection/Substance Withdrawal Syndrome/th [Therapy]/Substance-Related Disorders/di [Diagnosis]/Substance-Related Disorders/th [Therapy].

RefID:3828. Corrigan, J. D., Bogner, J., Hungerford, D. W., and Schomer, K.. Screening and brief intervention for substance misuse among patients with traumatic brain injury. [Review] [52 refs]. Journal of Trauma-Injury Infection & Critical Care 2010. 69 (3) 722-726. **Keywords:** Multifile Reviews/Brain Injuries/co [Complications]/Brain Injuries/px [Psychology]/Consciousness Disorders/co [Complications]/Emergency Service/hospital/Humans/Informed Consent/Substance-Related Disorders/co [Complications]/Substance-Related Disorders/di [Diagnosis].

RefID:3851. Altena, A. M., Brilleslijper-Kater, S. N., and Wolf, J. L.. Effective interventions for homeless youth: a systematic review. [Review] [53 refs]. American journal of preventive medicine 2010. 38 (6) 637-645. **Keywords:** Multifile Reviews/Adolescent/Child/Cognitive Therapy/mt [Methods]/Female/Health Services/Homeless Persons/Humans/Intervention Studies/Male/Quality of Life/Substance-Related Disorders/rh [Rehabilitation]/Young Adult.

RefID:3937. Walling, A. D.. Which treatments are effective for reducing adolescent alcohol abuse?. American family physician 2010. 82 (5) 532-534. **Keywords:** Multifile Reviews/Adolescent/Adult/alcohol abuse/alcohol consumption/article/Child/Cognitive Therapy/Drinking Behavior/Family Therapy/human/intervention study/meta analysis (topic)/outcome assessment/preschool child/school child/systematic review.

RefID:4040. Turnbull, Catherine, Osborn, David A., and Cochrane Database of Systematic

Reviews. Home visits during pregnancy and after birth for women with an alcohol or drug problem. #journal name# 2012. #volume# (1) #Start Page#-#End Page#. **Keywords:** CDSR/Female .checkword/Humans .checkword/Pregnancy .checkword/House Calls .utilization/Pregnancy Complications/Substance-Related Disorders/Alcohol-Related Disorders/Postnatal Care/Postpartum Period/Pregnancy Outcome/Prenatal Care.

RefID:4051. Amato, Laura, Minozzi, Silvia, Davoli, Marina, Vecchi, Simona, and Cochrane Database of Systematic Reviews. Psychosocial combined with agonist maintenance treatments versus agonist maintenance treatments alone for treatment of opioid dependence. #journal name# 2011. #volume# (10) #Start Page#-#End Page#. **Keywords:** CDSR/Humans .checkword/Combined Modality Therapy .methods/Narcotics .therapeutic use/Opioid-Related Disorders .psychology .rehabilitation/Psychotherapy .methods/Randomized Controlled Trials as Topic.

RefID:4083. Amato, Laura, Minozzi, Silvia, Davoli, Marina, Vecchi, Simona, and Cochrane Database of Systematic Reviews. Psychosocial and pharmacological treatments versus pharmacological treatments for opioid detoxification. #journal name# 2011. #volume# (9) #Start Page#-#End Page#. **Keywords:** CDSR/Adult .checkword/Female .checkword/Humans .checkword/Male .checkword/Buprenorphine .therapeutic use/Combined Modality Therapy .methods/Methadone .therapeutic use/Narcotics .therapeutic use/Opioid-Related Disorders .rehabilitation .therapy/Psychotherapy .methods/Randomized Controlled Trials as Topic.

RefID:10529. Thomas, R. E., Lorenzetti, D., and Spragins, W.. Mentoring adolescents to prevent drug and alcohol use. Cochrane Database of Systematic Reviews 2011. #volume# (11) #Start Page#-#End Page#. **Keywords:** 2012 Apr 18/adolescence/Alcohol Abuse -- Prevention and Control/Cochrane Library/Confidence Intervals/Embase/human/Mentorship -- In Adolescence/meta analysis/Outcomes (Health Care)/Relative Risk/Substance Abuse -- Prevention and Control/CINAHL Reviews.

RefID:10541. Amato, L., Minozzi, S., Davoli, M., and Vecchi, S.. Psychosocial and pharmacological treatments versus pharmacological treatments for opioid detoxification. Cochrane Database of Systematic Reviews 2011. #volume# (9) #Start Page#-#End Page#. **Keywords:** 2012 Apr 18/Buprenorphine -- Therapeutic Use/Clinical Trials/Combined Modality Therapy/Confidence Intervals/human/Methadone -- Therapeutic Use/Narcotics -- Adverse Effects/Psychotherapy/Relative Risk/Substance Withdrawal Syndrome -- Therapy/Treatment Outcomes/CINAHL Reviews.

RefID:10773. Cheon, Jeong Woong. Best Practices in Community-Based Prevention for Youth Substance Reduction: Towards Strengths-Based Positive Development Policy. Journal of Community Psychology 2008. 36 (6) 761-779. **Keywords:** ERIC Reviews/prevention/Public Health/Criticism/Youth/Learner Engagement/substance abuse/community/Psychology/School Districts/Educational Change/Federal Programs/State Programs/Local Issues.

Secondary analysis of a study

RefID:475. Madras, Bertha K., Compton, Wilson M., Avula, Deepa, Stegbauer, Tom, Stein, Jack B., and Clark, H. Westley. Screening, brief interventions, referral to treatment (SBIRT) for illicit drug and alcohol use at multiple healthcare sites: comparison at intake and 6 months later. Drug and alcohol dependence 2009. 99 (1-3) 280-295. **Keywords:** Adult/Age Factors/*Alcoholism/di [Diagnosis]/Alcoholism/ep [Epidemiology]/*Alcoholism/rh [Rehabilitation]/Community Health Centers/Crime/sn [Statistics & Numerical Data]/Data Interpretation,Statistical/Employment/sn [Statistics & Numerical Data]/Ethnic Groups/Female/Follow-Up Studies/Homeless Persons/sn [Statistics & Numerical Data]/Hospitals/Humans/Male/Middle Aged/Outpatient Clinics,Hospital/Rural Population/Sex Factors/*Street Drugs/*Substance-Related Disorders/di [Diagnosis]/Substance-Related Disorders/ep [Epidemiology]/*Substance-Related Disorders/rh [Rehabilitation]/Trauma Centers/Treatment Outcome/Urban Population/Young Adult.

RefID:620. Neufeld, Karin J., Kidorf, Michael S., Kolodner, Kenneth, King, Van L., Clark, Michael, and Brooner, Robert K.. A behavioral treatment for opioid-dependent patients with antisocial personality. Journal of substance abuse treatment 2008. 34 (1) 101-111. **Keywords:** Adult/*Antisocial Personality Disorder/ep [Epidemiology]/*Cognitive Therapy/mt [Methods]/Demography/Female/Humans/Male/*Methadone/tu [Therapeutic Use]/*Narcotics/tu [Therapeutic Use]/*Opioid-Related Disorders/ep [Epidemiology]/*Opioid-Related Disorders/th [Therapy]/Patient Compliance/sn [Statistics & Numerical Data]/Treatment Outcome.

RefID:625. Hogue, Aaron, Dauber, Sarah, Stambaugh, Leyla Faw, Cecero, John J., and Liddle, Howard A.. Early therapeutic alliance and treatment outcome in individual and family therapy for adolescent behavior problems. Journal of consulting and clinical psychology 2006. 74 (1) 121-129. **Keywords:** Adolescent/*Cognitive Therapy/Comorbidity/*Family Therapy/Female/Follow-Up Studies/Humans/*Internal-External Control/Male/Mental Disorders/px [Psychology]/*Mental Disorders/rh [Rehabilitation]/*Professional-Family Relations/*Professional-Patient Relations/Psychological Theory/Substance-Related Disorders/px [Psychology]/*Substance-Related Disorders/rh [Rehabilitation]/*Trust/px [Psychology].

RefID:783. Sindelar, Jody, Elbel, Brian, and Petry, Nancy M.. What do we get for our money? Cost-effectiveness of adding contingency management. Addiction (Abingdon, England) 2007. 102 (2) 309-316. **Keywords:** Adolescent/Adult/Aged/Cocaine-Related Disorders/ec [Economics]/*Cocaine-Related Disorders/rh [Rehabilitation]/Cost-Benefit Analysis/Female/Humans/Male/*Methadone/ec [Economics]/Methadone/tu [Therapeutic Use]/Middle Aged/Motivation/*Narcotics/ec [Economics]/Narcotics/tu [Therapeutic Use].

RefID:788. Neuner, Bruno, Fleming, Michael, Born, Rike, Weiss-Gerlach, Edith, Neumann, Tim, Rettig, Jordan, Lau, Alexandra, Schoenfeld, Helge, Kallischnigg, Gerd, and Spies, Claudia.

Predictors of loss to follow-up in young patients with minor trauma after screening and written intervention for alcohol in an urban emergency department. Journal of studies on alcohol and drugs 2007. 68 (1) 133-140. **Keywords:** Adolescent/Adult/Aged/Aged,80 and over/*Alcohol-Related Disorders/ep [Epidemiology]/*Alcohol-Related Disorders/rh [Rehabilitation]/*Communication/Educational Status/*Emergency Medical Services/sn [Statistics & Numerical Data]/Female/Follow-Up Studies/Humans/Male/Mass Screening/mt [Methods]/Middle Aged/*Patient Dropouts/sn [Statistics & Numerical Data]/Prospective Studies/Socioeconomic Factors/*Urban Population/sn [Statistics & Numerical Data]/*Verbal Behavior/*Wounds and Injuries/ep [Epidemiology].

RefID:1601. Siegler, E. L., Capezuti, E., Maislin, G., Baumgarten, M., Evans, L., and Strumpf, N.. Effects of a restraint reduction intervention and OBRA '87 regulations on psychoactive drug use in nursing homes. Journal of the American Geriatrics Society 1997. 45 (7) 791-796. **Keywords:** Aged/Aged,80 and over/Anti-Anxiety Agents/ad [Administration & Dosage]/Antidepressive Agents/ad [Administration & Dosage]/Antipsychotic Agents/ad [Administration & Dosage]/Benzodiazepines/Drug Utilization/Female/Humans/Male/Middle Aged/*Nursing Homes/lj [Legislation & Jurisprudence]/Nursing Staff/ed [Education]/*Psychotropic Drugs/ad [Administration & Dosage]/Referral and Consultation/*Restraint,Physical/lj [Legislation & Jurisprudence].

RefID:2055. Andrade, L. F. and Petry, N. M.. The impact of contingency managemet on quality of life among cocaine abusers with and without alcohol dependence. Alcoholism: Clinical and Experimental Research 2011. 35 (#Issue#) 142A-#End Page#. **Keywords:** *Alcoholism/*society/*Quality of Life/human/patient/substance abuse/clinical trial (topic)/outpatient/Analysis of Variance/univariate analysis/analysis of covariance/multivariate analysis of covariance/Treatment Outcome/Health Status/Comorbidity/alcohol consumption/population/treatment duration/*Cocaine.

RefID:2363. Naeem, F., Kingdon, D., and Turkington, D.. Cognitive behaviour therapy for Schizophrenia in patients with mild to moderate substance misuse problems. Cognitive Behaviour Therapy 2005. 34 (4) 207-215. **Keywords:** article/*Behavior Therapy/clinical feature/clinical trial/*Cognitive Therapy/controlled clinical trial/controlled study/Depression/disease association/disease severity/*drug misuse/di [Diagnosis]/*drug misuse/th [Therapy]/human/major clinical study/outcomes research/population distribution/randomized controlled trial/rating scale/Sample Size/*Schizophrenia/di [Diagnosis]/*Schizophrenia/th [Therapy]/scoring system/statistical analysis/statistical significance/*substance abuse/symptomatology.

RefID:2703. Curry, John, Silva, Susan, Rohde, Paul, Ginsburg, Golda, Kennard, Betsy, Kratochvil, Christopher, Simons, Anne, Kirchner, Jerry, May, Diane, Mayes, Taryn, Feeny, Norah, Albano, Anne Marie, Lavanier, Sarah, Reinecke, Mark, Jacobs, Rachel, Becker-Weidman, Emily, Weller, Elizabeth, Emslie, Graham, Walkup, John, Kastelic, Elizabeth, Burns, Barbara, Wells, Karen, and March, John. Onset of alcohol or substance use disorders following treatment for adolescent depression. Journal of consulting and clinical psychology 2012. 80 (2) 299-312. **Keywords:**

*Adolescent Psychopathology/*Alcoholism/*drug abuse/*major depression/*Onset (Disorders).

RefID:2885. Marsiglia, Flavio F., Pena, Veronica, Nieri, Tanya, and Nagoshi, Julie L.. Real groups: The design and immediate effects of a prevention intervention for Latino children. Social Work with Groups: A Journal of Community and Clinical Practice 2010. 33 (2-3) 103-121. **Keywords:** *Drug Abuse Prevention/*Program Development/*School Based Intervention/*Group Intervention/Latinos/Latinas.

RefID:3309. Malcolm, Barris P.. Evaluating the Effects of Self-Esteem on Substance Abuse among Homeless Men. Journal of Alcohol and Drug Education 2004. 48 (3) 39-61. **Keywords:** *alcohol abuse/*drug abuse/*Homeless/*Human Males/*Self Esteem/Personality Correlates.

RefID:5609. Baker, A., Kochan, N., Dixon, J., Wodak, A., and Heather, N.. HIV risk-taking behaviour among injecting drug users currently, previously and never enrolled in methadone treatment. Addiction (Abingdon, England) 1995. 90 (4) 545-554. **Keywords:** Central.

RefID:8816. Malcolm, B. P.. Evaluating the effects of self-esteem on substance abuse among homeless men. Journal of Alcohol & Drug Education 2004. 48 (3) 39-61. **Keywords:** CINAHL/Homeless Persons/Self Concept/substance abuse/Analysis of Variance/chi square test/Descriptive Statistics/Experimental Studies/Interviews/Male/Multivariate Analysis/multivariate analysis of covariance/Open-Ended Questionnaires/P-Value/Pearson's Correlation Coefficient/Psychological Tests/Random Assignment/Repeated Measures/Rosenberg Self Esteem Scale/Scales/secondary analysis/Self Report/human.

RefID:9378. McCusker, J, Bigelow, C, Vickers, Lahti, Spotts, D, Garfield, F, and Frost, R. Planned duration of residential drug abuse treatment: efficacy versus effectiveness. Addiction (Abingdon, England) 1997. 92 (11) 1467-1478. **Keywords:** CINAHL/Socioenvironmental Therapy/Substance Use Disorders -- Therapy/Adult/Female/human/Length of Stay/Male/Nursing Care Plans/Prospective Studies/Psychological Tests/Randomized Controlled Trials/Recurrence/Treatment Outcomes/United States.

RefID:10629. Subramaniam, Geetha A., Warden, Diane, Minhajuddin, Abu, Fishman, Marc J., Stitzer, Maxine L., Adinoff, Bryon, Trivedi, Madhukar, Weiss, Roger, Potter, Jennifer, Poole, Sabrina A., and Woody, George E.. Predictors of Abstinence: National Institute of Drug Abuse Multisite Buprenorphine/Naloxone Treatment Trial in Opioid-Dependent Youth. Journal of the American Academy of Child & Adolescent Psychiatry 2011. 50 (11) 1120-1128. **Keywords:** drug abuse/Drug Rehabilitation/Predictor Variables/Drug Addiction/drug therapy/Counseling/Regression (Statistics)/Mental Disorders/Health/Outcomes of Treatment/ERIC RCTS.

RefID:10694. Rowland, Melisa D., Chapman, Jason E., and Henggeler, Scott W.. Sibling Outcomes from a Randomized Trial of Evidence-Based Treatments with Substance Abusing Juvenile Offenders. Journal of Child & Adolescent Substance Abuse 2008. 17 (3) 11-26.

Keywords: Siblings/delinquency/Adolescents/substance abuse/Outcomes of Treatment/Program Effectiveness/Crime/Youth Programs/Contingency Management/Probability/Predictor Variables/ERIC RCTS.

Other design (eg, editorial, comment, letter)

RefID:3. Tamm, Leanne, Adinoff, Bryon, Nakonezny, Paul A., Winhusen, Theresa, and Riggs, Paula. Attention-deficit/hyperactivity disorder subtypes in adolescents with comorbid substance-use disorder. The American journal of drug and alcohol abuse 2012. 38 (1) 93-100. **Keywords:** #Keywords#.

RefID:14. Campbell, Aimee N. C., Nunes, Edward V., Miele, Gloria M., Matthews, Abigail, Polsky, Daniel, Ghitza, Udi E., Turrigiano, Eva, Bailey, Genie L., VanVeldhuisen, Paul, Chapdelaine, Rita, Froias, Autumn, Stitzer, Maxine L., Carroll, Kathleen M., Winhusen, Theresa, Clingerman, Sara, Perez, Livangelie, McClure, Erin, Goldman, Bruce, and Crowell, A. Rebecca. Design and methodological considerations of an effectiveness trial of a computer-assisted intervention: an example from the NIDA Clinical Trials Network. Contemporary clinical trials 2012. 33 (2) 386-395. **Keywords:** #Keywords#.

RefID:73. Alessi, Sheila M., Rash, Carla, and Petry, Nancy M.. Contingency management is efficacious and improves outcomes in cocaine patients with pretreatment marijuana use. Drug and alcohol dependence 2011. 118 (1) 62-67. **Keywords:** Adult/Ambulatory Care/*Behavior Therapy/mt [Methods]/Central Nervous System Stimulants/ur [Urine]/Cocaine/ur [Urine]/Cocaine-Related Disorders/di [Diagnosis]/*Cocaine-Related Disorders/rh [Rehabilitation]/Cocaine-Related Disorders/th [Therapy]/Female/Follow-Up Studies/Humans/Male/*Marijuana Smoking/px [Psychology]/Marijuana Smoking/ur [Urine]/Psychiatric Status Rating Scales/Reinforcement (Psychology)/*Substance Abuse Detection/Substance-Related Disorders/di [Diagnosis]/*Substance-Related Disorders/rh [Rehabilitation]/Substance-Related Disorders/ur [Urine]/Time Factors/Treatment Outcome.

RefID:80. Petry, Nancy M., Rash, Carla J., and Easton, Caroline J.. Contingency management treatment in substance abusers with and without legal problems. The journal of the American Academy of Psychiatry and the Law 2011. 39 (3) 370-378. **Keywords:** Adult/*Drug Users/lj [Legislation & Jurisprudence]/Female/Humans/Male/New England/*Outcome Assessment (Health Care)/*Substance-Related Disorders/th [Therapy].

RefID:115. Petry, Nancy M., Ford, Julian D., and Barry, Danielle. Contingency management is especially efficacious in engendering long durations of abstinence in patients with sexual abuse histories. Psychology of addictive behaviors : journal of the Society of Psychologists in Addictive Behaviors 2011. 25 (2) 293-300. **Keywords:** Adult/*Behavior Therapy/mt [Methods]/Female/Humans/Male/Middle Aged/*Sex Offenses/px [Psychology]/Substance Abuse Detection/Substance-Related Disorders/px [Psychology]/*Substance-Related

Disorders/th [Therapy]/Substance-Related Disorders/ur [Urine]/Treatment Outcome.

RefID:121. Unger, Annemarie, Jagsch, Reinhold, Jones, Hendree, Arria, Amelia, Leitich, Harald, Rohrmeister, Klaudia, Aschauer, Constantin, Winklbaur, Berndadette, Bawert, Andjela, and Fischer, Gabriele. Randomized controlled trials in pregnancy: scientific and ethical aspects. Exposure to different opioid medications during pregnancy in an intra-individual comparison. Addiction (Abingdon, England) 2011. 106 (7) 1355-1362. **Keywords:** Adult/*Analgesics,Opioid/tu [Therapeutic Use]/Birth Weight/de [Drug Effects]/*Buprenorphine/tu [Therapeutic Use]/Double-Blind Method/Female/Humans/Infant,Newborn/Male/*Methadone/tu [Therapeutic Use]/*Neonatal Abstinence Syndrome/ep [Epidemiology]/Neonatal Abstinence Syndrome/et [Etiology]/Neonatal Abstinence Syndrome/pc [Prevention & Control]/Opiate Substitution Treatment/mt [Methods]/Opioid-Related Disorders/co [Complications]/*Opioid-Related Disorders/dt [Drug Therapy]/Pregnancy/*Pregnancy Complications/dt [Drug Therapy]/Severity of Illness Index/Smoking/ep [Epidemiology]/Treatment Outcome/Young Adult.

RefID:137. Weiss, Lindsay M. and Petry, Nancy M.. Interaction effects of age and contingency management treatments in cocaine-dependent outpatients. Experimental and clinical psychopharmacology 2011. 19 (2) 173-181. **Keywords:** Adolescent/Adult/*Age Factors/Cocaine-Related Disorders/pp [Physiopathology]/*Cocaine-Related Disorders/th [Therapy]/Female/Humans/Male/*Outpatients/Treatment Outcome/Young Adult.

RefID:138. Byrne, Shannon A. and Petry, Nancy M.. Concurrent alcohol dependence among methadone-maintained cocaine abusers is associated with greater abstinence. Experimental and clinical psychopharmacology 2011. 19 (2) 116-122. **Keywords:** Adult/*Alcohol-Related Disorders/co [Complications]/*Cocaine-Related Disorders/dt [Drug Therapy]/Female/Follow-Up Studies/Humans/Male/*Methadone/tu [Therapeutic Use]/Middle Aged/*Substance Withdrawal Syndrome/Treatment Outcome.

RefID:160. Dierst-Davies, Rhodri, Reback, Cathy J., Peck, James A., Nuno, Miriam, Kamien, Jonathan B., and Amass, Leslie. Delay-discounting among homeless, out-of-treatment, substance-dependent men who have sex with men. The American journal of drug and alcohol abuse 2011. 37 (2) 93-97. **Keywords:** Adult/Case-Control Studies/Choice Behavior/*Homeless Persons/sn [Statistics & Numerical Data]/Homosexuality,Male/Humans/*Impulsive Behavior/ep [Epidemiology]/Impulsive Behavior/et [Etiology]/Male/Middle Aged/*Reward/*Substance-Related Disorders/px [Psychology]/Time Factors.

RefID:170. Garcia-Fernandez, Gloria, Secades-Villa, Roberto, Garcia-Rodriguez, Olaya, Alvarez-Lopez, Heli, Sanchez-Hervas, Emilio, Fernandez-Hermida, Jose Ramon, and Fernandez-Artamendi, Sergio. Individual characteristics and response to Contingency Management treatment for cocaine addiction. Psicothema 2011. 23 (1) 114-118. **Keywords:** Adult/Ambulatory Care/*Behavior Therapy/*Cocaine-Related Disorders/th [Therapy]/Comorbidity/Female/Humans/*Individuality/Male/Mental Disorders/ep [Epidemiology]/Motivation/Patient Compliance/Prognosis/Randomized Controlled Trials as

Topic/sn [Statistics & Numerical Data]/Recurrence/Severity of Illness Index/Socioeconomic Factors/Substance-Related Disorders/ep [Epidemiology]/*Token Economy/Treatment Outcome.

RefID:171. Gaudiano, Brandon A., Weinstock, Lauren M., and Miller, Ivan W.. Improving treatment adherence in patients with bipolar disorder and substance abuse: rationale and initial development of a novel psychosocial approach. Journal of psychiatric practice 2011. 17 (1) 5-20. **Keywords:** Adult/Bipolar Disorder/ep [Epidemiology]/Bipolar Disorder/px [Psychology]/*Bipolar Disorder/th [Therapy]/Comorbidity/Female/Follow-Up Studies/Humans/Male/Middle Aged/Patient Compliance/px [Psychology]/*Patient Compliance/sn [Statistics & Numerical Data]/Pilot Projects/Program Evaluation/mt [Methods]/*Psychotherapy/mt [Methods]/Questionnaires/Rhode Island/ep [Epidemiology]/Substance-Related Disorders/ep [Epidemiology]/Substance-Related Disorders/px [Psychology]/*Substance-Related Disorders/th [Therapy]/Treatment Outcome.

RefID:175. Dickerson, Daniel L., Spear, Suzanne, Marinelli-Casey, Patricia, Rawson, Richard, Li, Libo, and Hser, Yih Ing. American indians/alaska natives and substance abuse treatment outcomes: positive signs and continuing challenges. Journal of addictive diseases 2011. 30 (1) 63-74. **Keywords:** Adult/Alaska/*American Native Continental Ancestry Group/px [Psychology]/Counseling/mt [Methods]/Female/Humans/*Indians,North American/px [Psychology]/Male/*Psychotherapy/mt [Methods]/Randomized Controlled Trials as Topic/Risk Factors/*Substance-Related Disorders/th [Therapy]/Treatment Outcome.

RefID:216. Gibbons, Carly J., Nich, Charla, Steinberg, Karen, Roffman, Roger A., Corvino, Joanne, Babor, Thomas F., and Carroll, Kathleen M.. Treatment process, alliance and outcome in brief versus extended treatments for marijuana dependence. Addiction (Abingdon, England) 2010. 105 (10) 1799-1808. **Keywords:** Adult/Ambulatory Care/st [Standards]/Ambulatory Care/sn [Statistics & Numerical Data]/Analysis of Variance/Clinical Competence/st [Standards]/*Clinical Competence/sn [Statistics & Numerical Data]/Clinical Protocols/Cognitive Therapy/mt [Methods]/Data Interpretation,Statistical/Female/Guideline Adherence/st [Standards]/*Guideline Adherence/sn [Statistics & Numerical Data]/Humans/Male/Marijuana Abuse/px [Psychology]/*Marijuana Abuse/rh [Rehabilitation]/Motivation/*Outcome and Process Assessment (Health Care)/*Physician-Patient Relations/Psychometrics/Psychotherapy,Brief/mt [Methods]/Regression Analysis/Time Factors.

RefID:267. Smout, Matthew F., Longo, Marie, Harrison, Sonia, Minniti, Rinaldo, Cahill, Sharon, Wickes, Wendy, and White, Jason M.. The Psychostimulant Check-Up: A pilot study of a brief intervention to reduce illicit stimulant use. Drug and alcohol review 2010. 29 (2) 169-176. **Keywords:** Adult/*Amphetamine-Related Disorders/rh [Rehabilitation]/Female/Follow-Up Studies/Humans/Male/*Methamphetamine/ae [Adverse Effects]/Patient Acceptance of Health Care/Pilot Projects/*Psychotherapy,Brief/mt [Methods]/Substance Abuse,Intravenous/rh [Rehabilitation]/Young Adult.

RefID:272. Nilsen, H. K., Stiles, T. C., Landro, N. I., Fors, E. A., Kaasa, S., and Borchgrevink, P. C..

Patients with problematic opioid use can be weaned from codeine without pain escalation. Acta anaesthesiologica Scandinavica 2010. 54 (5) 571-579. **Keywords:** Adult/*Analgesics,Opioid/ad [Administration & Dosage]/Analysis of Variance/Chronic Disease/*Codeine/ad [Administration & Dosage]/Cognition/de [Drug Effects]/*Cognitive Therapy/mt [Methods]/Female/Follow-Up Studies/Health Status/Humans/Male/Middle Aged/Neuropsychological Tests/*Pain/dt [Drug Therapy]/Pain Measurement/Quality of Life/px [Psychology]/*Substance-Related Disorders/pc [Prevention & Control]/Young Adult.

RefID:298. Horigian, Viviana E., Robbins, Michael S., Dominguez, Roberto, Ucha, Jessica, and Rosa, Carmen L.. Principles for defining adverse events in behavioral intervention research: lessons from a family-focused adolescent drug abuse trial. Clinical trials (London, England) 2010. 7 (1) 58-68. **Keywords:** Adolescent/Adolescent Behavior/px [Psychology]/*Behavior Therapy/*Behavioral Research/*Conduct Disorder/cl [Classification]/*Family Therapy/mt [Methods]/Humans/Multicenter Studies as Topic/Substance-Related Disorders/pp [Physiopathology]/*Substance-Related Disorders/px [Psychology].

RefID:361. Dean, Andy C., London, Edythe D., Sugar, Catherine A., Kitchen, Christina M. R., Swanson, Aimee Noelle, Heinzerling, Keith G., Kalechstein, Ari D., and Shoptaw, Steven. Predicting adherence to treatment for methamphetamine dependence from neuropsychological and drug use variables. Drug and alcohol dependence 2009. 105 (1-2) 48-55. **Keywords:** Adult/Affect/*Amphetamine-Related Disorders/px [Psychology]/*Amphetamine-Related Disorders/rh [Rehabilitation]/Analysis of Variance/Antidepressive Agents,Second-Generation/tu [Therapeutic Use]/Bupropion/tu [Therapeutic Use]/Cognitive Therapy/Crime/Female/Humans/Male/Memory,Short-Term/de [Drug Effects]/*Methamphetamine/Neuropsychological Tests/*Patient Compliance/Predictive Value of Tests/Reaction Time/de [Drug Effects]/Reaction Time/ph [Physiology]/Smoking/px [Psychology]/Social Environment/Socioeconomic Factors/Treatment Outcome.

RefID:434. Haller, Dagmar M., Meynard, Anne, Lefebvre, Daniele, Tylee, Andre, Narring, Francoise, and Broers, Barbara. Brief intervention addressing excessive cannabis use in young people consulting their GP: a pilot study. The British journal of general practice : the journal of the Royal College of General Practitioners 2009. 59 (560) 166-172. **Keywords:** Adolescent/*Cannabis/ae [Adverse Effects]/*Family Practice/Feasibility Studies/Female/Humans/Male/*Marijuana Abuse/pc [Prevention & Control]/*Physician's Role/Pilot Projects/Questionnaires/Switzerland/Young Adult.

RefID:468. Sigmon, Stacey C., Dunn, Kelly E., Badger, Gary J., Heil, Sarah H., and Higgins, Stephen T.. Brief buprenorphine detoxification for the treatment of prescription opioid dependence: a pilot study. Addictive behaviors 2009. 34 (3) 304-311. **Keywords:** Adult/Behavior Therapy/*Buprenorphine/tu [Therapeutic Use]/Epidemiologic Methods/Feasibility Studies/Female/Humans/Male/*Naltrexone/tu [Therapeutic Use]/*Narcotic Antagonists/tu [Therapeutic Use]/*Opioid-Related Disorders/rh [Rehabilitation]/Opioid-Related Disorders/ur [Urine]/*Prescription Drugs/ae [Adverse Effects]/Substance Abuse Detection/Time Factors.

RefID:488. Ledgerwood, David M., Alessi, Sheila M., Hanson, Tressa, Godley, Mark D., and Petry, Nancy M.. Contingency management for attendance to group substance abuse treatment administered by clinicians in community clinics. Journal of applied behavior analysis 2008. 41 (4) 517-526. **Keywords:** Adult/*Alcoholism/rh [Rehabilitation]/Community Mental Health Services/Female/Humans/Male/Middle Aged/Motivation/*Patient Compliance/px [Psychology]/Patient Dropouts/px [Psychology]/*Psychotherapy,Group/Substance-Related Disorders/px [Psychology]/*Substance-Related Disorders/rh [Rehabilitation]/*Token Economy.

RefID:550. Godley, Susan H., Godley, Mark D., Wright, Kelli L., Funk, Rodney R., and Petry, Nancy M.. Contingent reinforcement of personal goal activities for adolescents with substance use disorders during post-residential continuing care. The American journal on addictions / American Academy of Psychiatrists in Alcoholism and Addictions 2008. 17 (4) 278-286. **Keywords:** Adolescent/*Aftercare/Comorbidity/Educational Status/Family Relations/Female/Follow-Up Studies/*Goals/Humans/Male/*Motivation/Parenting/px [Psychology]/Patient Acceptance of Health Care/Patient Compliance/px [Psychology]/Rehabilitation,Vocational/*Reinforcement (Psychology)/*Residential Treatment/Social Adjustment/Social Behavior/Substance-Related Disorders/px [Psychology]/*Substance-Related Disorders/rh [Rehabilitation]/Temperance/px [Psychology].

RefID:626. Wilburn, Sharon T., Wilburn, Kenneth T., Weaver, Dax M., and Bowles, Kathy. Pearls and pitfalls in evaluating a Student Assistance Program: a five-year case study. Journal of drug education 2007. 37 (4) 447-467. **Keywords:** Adolescent/Child/Demography/Female/*Health Education/og [Organization & Administration]/Health Knowledge,Attitudes,Practice/Humans/Male/Minority Groups/Organizational Case Studies/Program Evaluation/Risk Factors/*School Health Services/og [Organization & Administration]/*Substance-Related Disorders/pc [Prevention & Control]/*Substance-Related Disorders/th [Therapy].

RefID:627. Galloway, Gantt P., Polcin, Douglas, Kielstein, Anousheh, Brown, Michelle, and Mendelson, John. A nine session manual of motivational enhancement therapy for methamphetamine dependence: adherence and efficacy. Journal of psychoactive drugs 2007. Suppl 4 (#Issue#) 393-400. **Keywords:** Adolescent/Adult/Amphetamine-Related Disorders/px [Psychology]/*Amphetamine-Related Disorders/th [Therapy]/Amphetamine-Related Disorders/ur [Urine]/California/ep [Epidemiology]/*Central Nervous System Stimulants/Data Interpretation,Statistical/Female/Follow-Up Studies/Humans/Male/*Methamphetamine/Patient Acceptance of Health Care/Patient Compliance/Psychiatric Status Rating Scales/*Psychotherapy,Brief/Sex Factors/Substance Abuse Detection/Treatment Outcome.

RefID:638. D'Amico, Elizabeth J. and Edelen, Maria Orlando. Pilot test of Project CHOICE: a voluntary afterschool intervention for middle school youth. Psychology of addictive behaviors : journal of the Society of Psychologists in Addictive Behaviors 2007. 21 (4) 592-598. **Keywords:** Adolescent/Child/*Choice Behavior/Female/Humans/Male/Pilot Projects/*School Health Services/*Substance-Related Disorders/pc [Prevention & Control].

RefID:714. Suarez-Morales, Lourdes, Matthews, Julie, Martino, Steve, Ball, Samuel A., Rosa, Carmen, Farentinos, Christine, Szapocznik, Jose, and Carroll, Kathleen M.. Issues in designing and implementing a Spanish-language multi-site clinical trial. The American journal on addictions / American Academy of Psychiatrists in Alcoholism and Addictions 2007. 16 (3) 206-215. **Keywords:** *Communication Barriers/Culture/Emigration and Immigration/*Ethnic Groups/Humans/*Multicenter Studies as Topic/mt [Methods]/Multilingualism/Patient Dropouts/Patient Selection/*Randomized Controlled Trials as Topic/mt [Methods]/Research Design/Research Personnel/*Substance-Related Disorders/th [Therapy]/United States.

RefID:743. Petry, Nancy M., Alessi, Sheila M., and Hanson, Tressa. Contingency management improves abstinence and quality of life in cocaine abusers. Journal of consulting and clinical psychology 2007. 75 (2) 307-315. **Keywords:** Adult/*Cocaine-Related Disorders/ep [Epidemiology]/*Cocaine-Related Disorders/pc [Prevention & Control]/Demography/Female/Humans/Male/*Mental Health Services/og [Organization & Administration]/Patient Selection/Prevalence/*Quality of Life/px [Psychology]/Questionnaires/Treatment Outcome.

RefID:987. Lewis, Marilyn W. and Petry, Nancy M.. Contingency management treatments that reinforce completion of goal-related activities: participation in family activities and its association with outcomes. Drug and alcohol dependence 2005. 79 (2) 267-271. **Keywords:** Adult/Cocaine-Related Disorders/px [Psychology]/*Cocaine-Related Disorders/th [Therapy]/*Family/Female/*Goals/Humans/*Leisure Activities/Male/*Patient Compliance/*Reinforcement Schedule/Sex Factors/Treatment Outcome.

RefID:1121. Kirby, Kris N. and Petry, Nancy M.. Heroin and cocaine abusers have higher discount rates for delayed rewards than alcoholics or non-drug-using controls. Addiction (Abingdon, England) 2004. 99 (4) 461-471. **Keywords:** Adult/*Alcoholism/px [Psychology]/Choice Behavior/*Cocaine-Related Disorders/px [Psychology]/Female/*Heroin Dependence/px [Psychology]/Humans/Impulsive Behavior/px [Psychology]/Male/Middle Aged/Motivation/Questionnaires/*Reward/Time Factors.

RefID:1349. Pollack, H. A.. Cost-effectiveness of harm reduction in preventing hepatitis C among injection drug users. Medical decision making : an international journal of the Society for Medical Decision Making 2001. 21 (5) 357-367. **Keywords:** *Blood-Borne Pathogens/Cost-Benefit Analysis/Effect Modifier,Epidemiologic/HIV Infections/ep [Epidemiology]/HIV Infections/et [Etiology]/HIV Infections/pc [Prevention & Control]/Health Care Costs/Hepatitis C/ep [Epidemiology]/*Hepatitis C/et [Etiology]/*Hepatitis C/pc [Prevention & Control]/Humans/Models,Econometric/Needle Sharing/ec [Economics]/*Needle-Exchange Programs/ec [Economics]/Outcome Assessment (Health Care)/Prevalence/Risk-Taking/Substance Abuse,Intravenous/ep [Epidemiology]/*Substance Abuse,Intravenous/vi [Virology].

RefID:1374. Petry, N. M., Petrakis, I., Trevisan, L., Wiredu, G., Boutros, N. N., Martin, B., and

Kosten, T. R.. Contingency management interventions: from research to practice. The American journal of psychiatry 2001. 158 (5) 694-702. **Keywords:** Adult/*Behavior Therapy/mt [Methods]/Female/Humans/Male/Middle Aged/Patient Compliance/Randomized Controlled Trials as Topic/*Reinforcement (Psychology)/Reward/Substance Abuse Treatment Centers/Substance-Related Disorders/rh [Rehabilitation]/*Substance-Related Disorders/th [Therapy]/Token Economy/Treatment Outcome.

RefID:1404. Tuttle, J., Bidwell-Cerone, S., Campbell-Heider, N., Richeson, G., and Collins, S.. Teen Club: a nursing intervention for reducing risk-taking behavior and improving well-being in female African American adolescents. Journal of pediatric health care : official publication of National Association of Pediatric Nurse Associates & Practitioners 2000. 14 (3) 103-108. **Keywords:** Adolescent/*Adolescent Behavior/px [Psychology]/Adolescent Psychology/*African Americans/px [Psychology]/Attitude to Health/*Community Health Nursing/og [Organization & Administration]/*Community Health Workers/og [Organization & Administration]/Female/*Health Behavior/*Health Promotion/og [Organization & Administration]/Humans/New York/Nursing Evaluation Research/Pilot Projects/Program Evaluation/Questionnaires/Retrospective Studies/*Risk-Taking/*Self-Help Groups/og [Organization & Administration].

RefID:1455. Roll, J. M. and Higgins, S. T.. A within-subject comparison of three different schedules of reinforcement of drug abstinence using cigarette smoking as an exemplar. Drug and alcohol dependence 2000. 58 (1-2) 103-109. **Keywords:** Adult/Analysis of Variance/Behavior Therapy/Chi-Square Distribution/Female/Humans/Male/*Reinforcement Schedule/*Smoking/th [Therapy]/*Substance-Related Disorders/th [Therapy].

RefID:1465. Rounsaville, B. J., Weiss, R., and Carroll, K.. Options for managing psychotropic medications in drug-abusing patients participating in behavioral therapies clinical trials. The American journal on addictions / American Academy of Psychiatrists in Alcoholism and Addictions 1999. 8 (3) 178-189. **Keywords:** *Behavior Therapy/Humans/Psychotropic Drugs/ad [Administration & Dosage]/*Psychotropic Drugs/tu [Therapeutic Use]/*Randomized Controlled Trials as Topic/Reproducibility of Results/Research Design/*Substance-Related Disorders/th [Therapy].

RefID:1483. Craig, A., Hancock, K., and Dickson, H.. Improving the long-term adjustment of spinal cord injured persons. Spinal cord 1999. 37 (5) 345-350. **Keywords:** Adolescent/Adult/Chi-Square Distribution/*Cognitive Therapy/Female/Humans/Interpersonal Relations/Longitudinal Studies/Male/Middle Aged/Patient Admission/sn [Statistics & Numerical Data]/Prejudice/Psychometrics/*Quality of Life/Questionnaires/*Social Adjustment/*Spinal Cord Injuries/px [Psychology]/*Spinal Cord Injuries/rh [Rehabilitation]/Substance-Related Disorders/ep [Epidemiology].

RefID:1667. Bell, D. C., Williams, M. L., Nelson, R., and Spence, R. T.. A pilot study of client progress in short-term drug abuse treatment. Journal of psychoactive drugs 1995. 27 (3) 211-221. **Keywords:** Adolescent/Adult/Ambulatory Care

Facilities/Cognition/Emotions/Female/Humans/Male/Middle Aged/Pilot Projects/*Psychotherapy,Brief/Residential Treatment/Social Support/Substance-Related Disorders/px [Psychology]/*Substance-Related Disorders/rh [Rehabilitation]/Treatment Outcome.

RefID:1682. Bell, J., Chan, J., and Kuk, A.. Investigating the influence of treatment philosophy on outcome of methadone maintenance. Addiction (Abingdon, England) 1995. 90 (6) 823-830. **Keywords:** Adult/Aftercare/Cohort Studies/Dose-Response Relationship,Drug/Female/Heroin Dependence/px [Psychology]/*Heroin Dependence/rh [Rehabilitation]/Humans/Long-Term Care/Male/*Methadone/tu [Therapeutic Use]/New South Wales/*Organizational Policy/Recurrence/Substance Abuse Treatment Centers/Substance Withdrawal Syndrome/pc [Prevention & Control]/Substance Withdrawal Syndrome/px [Psychology]/Treatment Outcome.

RefID:1845. Pentz, M. A., Dwyer, J. H., MacKinnon, D. P., Flay, B. R., Hansen, W. B., Wang, E. Y., and Johnson, C. A.. A multicommunity trial for primary prevention of adolescent drug abuse. Effects on drug use prevalence. JAMA : the journal of the American Medical Association 1989. 261 (22) 3259-3266. **Keywords:** Adolescent/*Adolescent Behavior/Child/Cohort Studies/*Community Health Services/og [Organization & Administration]/Cross-Sectional Studies/Female/Humans/Kansas/Male/Missouri/*Primary Prevention/Substance-Related Disorders/ep [Epidemiology]/*Substance-Related Disorders/pc [Prevention & Control].

RefID:1883. Kirmil-Gray, K., Eagleston, J. R., Thoresen, C. E., and Zarcone, V. P. J.. Brief consultation and stress management treatments for drug-dependent insomnia: effects on sleep quality, self-efficacy, and daytime stress. Journal of behavioral medicine 1985. 8 (1) 79-99. **Keywords:** Adult/Aged/Alcohol Drinking/*Behavior Therapy/Electroencephalography/Female/Follow-Up Studies/Humans/*Hypnotics and Sedatives/Middle Aged/*Sleep Initiation and Maintenance Disorders/th [Therapy]/Sleep Stages/*Stress,Psychological/co [Complications]/Substance Withdrawal Syndrome/th [Therapy]/*Substance-Related Disorders/th [Therapy].

RefID:1889. Stephenson, J. N., Moberg, P., Daniels, B. J., and Robertson, J. F.. Treating the intoxicated adolescent. A need for comprehensive services. JAMA : the journal of the American Medical Association 1984. 252 (14) 1884-1888. **Keywords:** Adolescent/Adolescent Behavior/Alcohol Drinking/Alcoholic Intoxication/co [Complications]/*Alcoholic Intoxication/th [Therapy]/*Alcoholism/rh [Rehabilitation]/Child/Emergency Service,Hospital/Female/Humans/Male/Patient Compliance/Prospective Studies/Referral and Consultation/Substance-Related Disorders/co [Complications].

RefID:1906. Bigelow, G. E., Stitzer, M. L., Griffiths, R. R., and Liebson, I. A.. Contingency management approaches to drug self-administration and drug abuse: efficacy and limitations. Addictive behaviors 1981. 6 (3) 241-252. **Keywords:** Alcoholism/px [Psychology]/Animals/Behavior Therapy/*Conditioning,Operant/Ethanol/ad [Administration & Dosage]/Humans/Macaca mulatta/Papio/Reinforcement (Psychology)/Reinforcement Schedule/Self Administration/Self Medication/*Substance-Related Disorders/px

[Psychology]/Substance-Related Disorders/th [Therapy]/Tobacco Use Disorder/px [Psychology].

RefID:1914. Desmond, D. P.. Effectiveness of psychotherapeutic counseling in methadone maintenance. Drug and alcohol dependence 1979. 4 (6) 439-447. **Keywords:** Counseling/Double-Blind Method/Humans/*Methadone/tu [Therapeutic Use]/Methadyl Acetate/tu [Therapeutic Use]/Narcotics/ur [Urine]/Opioid-Related Disorders/rh [Rehabilitation]/*Psychotherapy/mt [Methods]/Psychotherapy,Group.

RefID:1928. Casswell, S.. Cannabis intoxication: effects of monetary incentive on performance, a controlled investigation of behavioural tolerance in moderate users of cannabis. Perceptual and motor skills 1975. 41 (2) 423-434. **Keywords:** Adult/Cannabis/ad [Administration & Dosage]/*Cannabis/*Cognition/de [Drug Effects]/Dose-Response Relationship,Drug/Educational Status/Female/Goals/Humans/Male/Memory,Short-Term/de [Drug Effects]/Motivation/de [Drug Effects]/Phytotherapy/Placebos/*Reaction Time/de [Drug Effects]/*Reward/Sex Factors/*Substance-Related Disorders/co [Complications].

RefID:1952. Bagoien, G., Bjorngaard, J., Ostensen, C., Romundstad, P., and Morken, G.. Motivational interviewing to patients with comorbid substance use admitted to an acute psychiatric department. Psychiatrische Praxis 2011. 38 (#Issue#) #Start Page#-#End Page#. **Keywords:** *human/*patient/*psychiatric department/*mental health service/randomized controlled trial/drug use/questionnaire/psychosis/hospital/Counseling/health service/population/Length of Stay/registration/diagnosis/university hospital/randomization/Education/social interaction/behavior change/alcohol/benzodiazepine derivative.

RefID:1953. Latimer, E. and Rabouin, D.. The at Home/Chez Soi Canadian Study of Housing First for people who are homeless and mentally ill: Study design and baseline data for the Montreal site. Psychiatrische Praxis 2011. 38 (#Issue#) #Start Page#-#End Page#. **Keywords:** *Housing/*human/*mental health service/*mental disease/*therapy/*homelessness/*study design/United States/city/substance abuse/Mental Health/adaptation/model/diagnosis/information processing/follow up/screening/algorithm/Male/community/Quality of Life/employment status/Income/Health/justice/group therapy/experimental study/Case Management/Canada/randomization.

RefID:1961. Moore, R. A. and Aubin, H.-J.. Do placebo response rates from cessation trials inform on strength of addictions?. International Journal of Environmental Research and Public Health 2012. 9 (1) 192-211. **Keywords:** abstinence/*addiction/Alcoholism/dt [Drug Therapy]/article/cannabis addiction/th [Therapy]/cocaine dependence/dt [Drug Therapy]/cocaine dependence/th [Therapy]/Cognitive Therapy/disease severity/drug dependence/dt [Drug Therapy]/group therapy/human/intermethod comparison/major clinical study/meta analysis/nicotine replacement therapy/opiate addiction/dt [Drug Therapy]/outcome assessment/*placebo effect/Smoking Cessation/systematic review/tobacco dependence/dt [Drug Therapy]/tobacco dependence/th [Therapy]/acamprosate/ct [Clinical

Trial]/acamprosate/dt [Drug Therapy]/amantadine/ct [Clinical Trial]/amantadine/dt [Drug Therapy]/amfebutamone/ct [Clinical Trial]/amfebutamone/dt [Drug Therapy]/anticonvulsive agent/ct [Clinical Trial]/anticonvulsive agent/dt [Drug Therapy]/antidepressant agent/ct [Clinical Trial]/antidepressant agent/dt [Drug Therapy]/buprenorphine/ct [Clinical Trial]/buprenorphine/cm [Drug Comparison]/buprenorphine/dt [Drug Therapy]/carbamazepine/ct [Clinical Trial]/carbamazepine/dt [Drug Therapy]/clonidine/ct [Clinical Trial]/clonidine/cm [Drug Comparison]/clonidine/dt [Drug Therapy]/dexamphetamine/ct [Clinical Trial]/dexamphetamine/dt [Drug Therapy]/dopamine receptor stimulating agent/ct [Clinical Trial]/dopamine receptor stimulating agent/dt [Drug Therapy]/mazindol/ct [Clinical Trial]/mazindol/dt [Drug Therapy]/methadone/ct [Clinical Trial]/methadone/dt [Drug Therapy]/methylphenidate/ct [Clinical Trial]/methylphenidate/dt [Drug Therapy]/modafinil/ct [Clinical Trial]/modafinil/dt [Drug Therapy]/naltrexone/ct [Clinical Trial]/naltrexone/dt [Drug Therapy]/neuroleptic agent/ct [Clinical Trial]/neuroleptic agent/dt [Drug Therapy]/opiate antagonist/ct [Clinical Trial]/opiate antagonist/dt [Drug Therapy]/placebo/varenicline/ct [Clinical Trial]/varenicline/dt [Drug Therapy].

RefID:2085. Martini, D., Kaplan, B., Hopkins, E., and Broderick, K.. Emergency physician utilization of alcohol/substance screening and discharge tools. Academic Emergency Medicine 2011. 18 (5 SUPPL. 1) S66-#End Page#. **Keywords:** *screening/*emergency physician/*Emergency Medicine/*society/human/patient/abuse/physician/Risk/population/Education/substance abuse/United States/money/emergency ward/emergency/college/alcohol.

RefID:2086. Broderick, K. B. and Richmond, M.. Evaluation of denver health's new brief screen tool for detection of risky substance use. Academic Emergency Medicine 2011. 18 (5 SUPPL. 1) S29-#End Page#. **Keywords:** *health/*Emergency Medicine/*society/human/patient/tobacco/screening/health educator/Adult/Sensitivity and Specificity/confidence interval/standard/Safety/community/hospital/Smoking/screening test/ethnicity/information processing/Sample Size/emergency ward/alcohol consumption/population/drug use/Public Health/nurse/Cannabis/alcohol/street drug.

RefID:2102. Bickel, W. K., Jones, B. A., Landes, R. D., Christensen, D. R., Jackson, L., and Mancino, M.. Hypothetical Intertemporal Choice and Real Economic Behavior: Delay Discounting Predicts Voucher Redemptions During Contingency-Management Procedures. Experimental and clinical psychopharmacology 2010. 18 (6) 546-552. **Keywords:** Adult/Age Distribution/article/*Decision Making/Female/human/Income/major clinical study/Male/money/opiate addiction/dt [Drug Therapy]/randomized controlled trial/*reinforcement/Urinalysis/buprenorphine/dt [Drug Therapy]/buprenorphine/li [Sublingual Drug Administration].

RefID:2124. Bucci, S., Baker, A., Halpin, S. A., Hides, L., Lewin, T. J., Carr, V. J., and Startup, M.. Intervention for cannabis use in young people at ultra high risk for psychosis and in early psychosis. Mental Health and Substance Use: Dual Diagnosis 2010. 3 (1) 66-73. **Keywords:** Adolescent/Adult/Age Distribution/article/*cannabis addiction/Child/Cognitive Therapy/drug

use/drug use scale of the opiate treatment index/*early intervention/feasibility study/Female/follow up/functional assessment/global assessment of functioning/*high risk population/human/major clinical study/Male/medical service/mental health service/Motivation/outcome assessment/priority journal/psychological rating scale/*psychosis/th [Therapy]/Risk Assessment/school child/scoring system/*cannabis/do [Drug Dose]/*cannabis/to [Drug Toxicity].

RefID:2177. Sysko, R. and Hildebrandt, T.. Cognitive-behavioural therapy for individuals with Bulimia nervosa and a co-occurring substance use disorder. European Eating Disorders Review 2009. 17 (2) 89-100. **Keywords:** alcohol withdrawal/Alcoholism/dt [Drug Therapy]/Alcoholism/th [Therapy]/ambivalence/article/Behavior Therapy/borderline state/th [Therapy]/*bulimia/dt [Drug Therapy]/*Bulimia/th [Therapy]/clinical effectiveness/clinical feature/clinical trial/*Cognitive Therapy/Comorbidity/conflict/*drug dependence/dt [Drug Therapy]/drug efficacy/eating disorder/dt [Drug Therapy]/human/human relation/impulsiveness/Motivation/opiate addiction/dt [Drug Therapy]/patient attitude/Psychotherapy/substance abuse/acamprosate/dt [Drug Therapy]/fluoxetine/dt [Drug Therapy]/naltrexone/dt [Drug Therapy]/placebo.

RefID:2258. Davey, J., Leal, N., and Freeman, J.. Screening for drugs in oral fluid: Illicit drug use and drug driving in a sample of Queensland motorists. Drug and alcohol review 2007. 26 (3) 301-307. **Keywords:** *addiction/di [Diagnosis]/*addiction/ep [Epidemiology]/Adolescent/Adult/Aged/article/Australia/breath analysis/*car driving/chemistry/cross-sectional study/Female/human/legal aspect/Male/*Mass Screening/methodology/Middle Aged/*saliva/Sensitivity and Specificity/*substance abuse/amphetamine/an [Drug Analysis]/cocaine/an [Drug Analysis]/diamorphine/an [Drug Analysis]/*street drug/an [Drug Analysis]/tetrahydrocannabinol/an [Drug Analysis].

RefID:2317. Feeney, G. F. X., Connor, J. P., Young, R. M., Tucker, J., and McPherson, A.. Improvement in measures of psychological distress amongst amphetamine misusers treated with brief cognitive-behavioural therapy (CBT). Addictive behaviors 2006. 31 (10) 1833-1843. **Keywords:** Adult/anxiety disorder/co [Complication]/anxiety disorder/th [Therapy]/article/*Behavior Therapy/clinical trial/*Cognitive Therapy/controlled clinical trial/controlled study/coping behavior/depression/co [Complication]/Depression/th [Therapy]/Discriminant Analysis/disease severity/*distress syndrome/co [Complication]/*distress syndrome/th [Therapy]/*drug dependence/drug withdrawal/Female/General Health Questionnaire/human/major clinical study/Male/mental stress/Patient Compliance/Problem Solving/psychosomatic disorder/co [Complication]/psychosomatic disorder/th [Therapy]/rating scale/Self Concept/sociopathy/co [Complication]/sociopathy/th [Therapy]/*amphetamine.

RefID:2425. Pantalon, M. V., Ferro, G., Chawarski, M. C., LaPaglia, D. M., Pakes, J. P., and Schottenfeld, R. S.. Voucher Purchases in Contingency Management Interventions for Women Cocaine Dependence. Addictive Disorders and their Treatment 2004. 3 (1) 27-35. **Keywords:** abstinence/Adult/article/*Behavior Therapy/clinical trial/*cocaine dependence/dm [Disease

Management]/*cocaine dependence/th [Therapy]/community care/community reinforcement approach/*contingency management/controlled clinical trial/controlled study/drug dependence treatment/evaluation/Female/*health care financing/household/human/intermethod comparison/major clinical study/practice guideline/priority journal/randomized controlled trial/recreation/reinforcement/Reward/Social Behavior.

RefID:2462. Breslin, C., Sdao-Jarvie, K., Li, S., Tupker, E., and Ittig-Deland, V.. Brief treatment for young substance abusers: A pilot study in an addiction treatment setting. Psychology of Addictive Behaviors 2002. 16 (1) 10-16. **Keywords:** *addiction/th [Therapy]/Adolescent/Adult/article/clinical trial/controlled clinical trial/controlled study/Female/follow up/human/long term care/major clinical study/Male/outpatient care/Psychotherapy/*substance abuse/treatment planning.

RefID:2543. Romach, M. K., Otton, S. V., Somer, G., Tyndale, R. F., and Sellers, E. M.. Cytochrome P450 2D6 and treatment of codeine dependence. Journal of clinical psychopharmacology 2000. 20 (1) 43-45. **Keywords:** Adult/article/clinical article/clinical trial/controlled clinical trial/controlled study/dose response/*drug mechanism/drug metabolism/enzyme activity/enzyme polymorphism/enzyme substrate/Female/human/Male/*opiate addiction/priority journal/reinforcement/*codeine/*cytochrome P450 2D6/ec [Endogenous Compound]/*cytochrome P450 isoenzyme/ec [Endogenous Compound]/*fluoxetine/ct [Clinical Trial]/*fluoxetine/do [Drug Dose]/*fluoxetine/pd [Pharmacology]/morphine/opiate/unclassified drug.

RefID:2544. Eisen, M., Keyser-Smith, J., Dampeer, J., and Sambrano, S.. Evaluation of substance use outcomes in demonstration projects for pregnant and postpartum women and their infants: Findings from a quasi-experiment. Addictive behaviors 2000. 25 (1) 123-129. **Keywords:** Adult/article/clinical trial/controlled clinical trial/controlled study/drug abuse/drug use/Female/health care delivery/*Health Education/human/major clinical study/Pregnancy/*prevention/puerperium/Self Report/statistical analysis/alcohol/Cannabis/Cocaine/illicit drug.

RefID:2549. Booth, R. E., Zhang, Y., and Kwiatkowski, C. F.. The challenge of changing drug and sex risk behaviors of runaway and homeless adolescents. Child Abuse and Neglect 1999. 23 (12) 1295-1306. **Keywords:** Adolescent/Adult/article/clinical trial/controlled clinical trial/*drug abuse/Female/Health Education/*homelessness/human/Human immunodeficiency virus infection/ep [Epidemiology]/Human immunodeficiency virus infection/et [Etiology]/Human immunodeficiency virus infection/pc [Prevention]/major clinical study/Male/randomized controlled trial/Regression Analysis/Risk Assessment/*runaway reaction/*Sexual Behavior/sexual education/statistical model/training/cocaine/to [Drug Toxicity]/diamorphine/to [Drug Toxicity].

RefID:2552. Sitharthan, T., Singh, S., Kranitis, P., Currie, J., Freeman, P., Murugesan, G., and

Ludowici, J.. Integrated drug and alcohol intervention: Development of an opportunistic intervention program to reduce alcohol and other substance use among psychiatric patients. Australian and New Zealand Journal of Psychiatry 1999. 33 (5) 676-683. **Keywords:** *alcohol abuse/article/*Behavior Therapy/*comorbidity/*drug misuse/pc [Prevention]/human/*mental disease/mental health service/Motivation/staff training/alcohol/amphetamine derivative/Cannabis/diamorphine/nicotine.

RefID:2591. Galanter, M., Keller, D. S., and Dermatis, H.. Network therapy for addiction: Assessment of the clinical outcome of training. American Journal of Drug and Alcohol Abuse 1997. 23 (3) 355-367. **Keywords:** Adult/article/Behavior Therapy/clinical article/clinical trial/Cognitive Therapy/controlled clinical trial/controlled study/*drug dependence/th [Therapy]/*drug dependence treatment/Female/human/Male/residency education/Treatment Outcome/*cocaine/to [Drug Toxicity].

RefID:2611. Loneck, B., Garrett, J. A., and Banks, S. M.. The Johnson Intervention and relapse during outpatient treatment. American Journal of Drug and Alcohol Abuse 1996. 22 (3) 363-375. **Keywords:** Adult/Alcoholism/article/clinical trial/controlled clinical trial/controlled study/*drug dependence treatment/Female/human/intermethod comparison/major clinical study/Male/*outpatient care/patient referral/*relapse.

RefID:2829. Anderson, Kristen G., Ramo, Danielle E., Cummins, Kevin M., and Brown, Sandra A.. Alcohol and drug involvement after adolescent treatment and functioning during emerging adulthood. Drug and alcohol dependence 2010. 107 (2-3) 171-181. **Keywords:** *Adolescent Development/*Alcohol Drinking Patterns/*Drug Rehabilitation/*Drug Usage/*Treatment Outcomes/drug abuse/Human Development/Involvement/Relapse (Disorders).

RefID:2883. Tobler, Amy L. and Komro, Kelli A.. Trajectories or parental monitoring and communication and effects on drug use among urban young adolescents. Journal of Adolescent Health 2010. 46 (6) 560-568. **Keywords:** *Drug Usage/*Monitoring/*Parent Child Communication/*Parenting Style/*Urban Environments/Adolescent Development.

RefID:2996. Bowser, Benjamin P., Ryan, Lisa, Smith, Carla Dillard, and Lockett, Gloria. Outreach-based drug treatment for sex trading women: The Cal-Pep risk-reduction demonstration project. International Journal of Drug Policy 2008. 19 (6) 492-495. **Keywords:** *Drug Rehabilitation/*Harm Reduction/*HIV/*Outreach Programs/*Prostitution.

RefID:3121. Belt, Ritva and Punamaki, Raija Leena. Mother-infant group psychotherapy as an intensive treatment in early interaction among mothers with substance abuse problems. Journal of Child Psychotherapy 2007. 33 (2) 202-220. **Keywords:** *drug abuse/*Drug Rehabilitation/*Family Therapy/*Group Psychotherapy/*Mother Child Relations/Mothers.

RefID:3178. Sullivan, Maria A., Rothenberg, Jami L., Vosburg, Suzanne K., Church, Sarah H., Feldman, Shara J., Epstein, Eva M., Kleber, Herbert D., and Nunes, Edward V.. Predictors of Retention in Naltrexone Maintenance for Opioid Dependence: Analysis of a Stage I Trial. The

American Journal on Addictions 2006. 15 (2) 150-159. **Keywords:** *Drug Rehabilitation/*naltrexone/*Opiates/*Treatment Compliance/*Treatment Dropouts/Drug Dependency/Retention.

RefID:3180. Christensen, Helen, Low, Lee Fay, and Anstey, Kaarin J.. Prevalence, risk factors and treatment for substance abuse in older adults. Current Opinion in Psychiatry 2006. 19 (6) 587-592. **Keywords:** *aging/*Alcohol Drinking Patterns/*drug abuse/*Risk Factors/*Treatment/Cognitive Processes/Drug Dependency/epidemiology/Mental Health.

RefID:3274. Boyd, Mary R., Moneyham, Linda, Murdaugh, Carolyn, Phillips, Kenneth D., Tavakoli, Abbas, Jackwon, Kirby, Jackson, Natalie, and Vyavaharkar, Medha. A Peer-Based Substance Abuse Intervention for HIV+ Rural Women: A Pilot Study. Archives of psychiatric nursing 2005. 19 (1) 10-17. **Keywords:** *drug abuse/*Drug Rehabilitation/*HIV/*Peer Counseling/*Rural Environments/Human Females/Intervention.

RefID:3321. Waldron, Holly Barrett and Kaminer, Yifrah. On the learning curve: The emerging evidence supporting cognitive-behavioral therapies for adolescent substance abuse. Addiction (Abingdon, England) 2004. 99 (Suppl2) 93-105. **Keywords:** *Adolescent Psychology/*Cognitive Behavior Therapy/*drug abuse/*Drug Rehabilitation/*Treatment Outcomes.

RefID:3334. Copeland, Jan. Developments in the treatment of cannabis use disorder. Current Opinion in Psychiatry 2004. 17 (3) 161-167. **Keywords:** *Cannabis/*Cognitive Therapy/*drug abuse/*Drug Dependency/*Drug Rehabilitation.

RefID:3354. Mullaney, Donald K.. Antisocial personality disorder (ASPD) and substance abuse treatment outcomes in a post-prison population. Dissertation Abstracts International Section A: Humanities and Social Sciences 2003. 63 (9-A) 3358-#End Page#. **Keywords:** *Antisocial Personality Disorder/*drug abuse/*Group Psychotherapy/*Relapse Prevention/*Treatment Outcomes.

RefID:3382. Rowe, Cynthia, Parker-Sloat, Elizabeth, Schwartz, Seth, and Liddle, Howard. Family therapy for early adolescent substance abuse. #journal name# 2003. #volume# (#Issue#) 105-132. **Keywords:** *drug abuse/*Drug Rehabilitation/*Family Therapy/*Treatment Effectiveness Evaluation/Adolescent Psychology/Family.

RefID:3437. Lyketsos, Constantine G., Fishman, Marc, Hutton, Heidi, Cox, Todd, Hobbs, Susan, Spoler, Charles, Hunt, Wayne, Driscoll, Jeannine, and Treisman, Glenn. The effectiveness of psychiatric treatment for HIV-infected patients. Psychosomatics: Journal of Consultation Liaison Psychiatry 1997. 38 (5) 423-432. **Keywords:** *drug abuse/*Drug Therapy/*HIV/*Individual Psychotherapy/*Mental Disorders/Followup Studies/Outpatients/Treatment Effectiveness Evaluation.

RefID:3451. Mcclanahan, Terry Michael. A comparative evaluation of cognitive-behavioral therapy and insight-oriented psychotherapy in the treatment of comorbid substance abuse,

anxiety, and depression in substance abusing females. Dissertation Abstracts International: Section B: The Sciences and Engineering 2001. 62 (3-B) 1587-#End Page#. **Keywords:** *Cognitive Therapy/*comorbidity/*drug abuse/*Insight (Psychotherapeutic Process)/*major depression/Anxiety/Human Females.

RefID:3481. Van Horn, Deborah H. A. and Bux, Donald A. J.. A pilot test of motivational interviewing groups for dually diagnosed inpatients. Journal of substance abuse treatment 2001. 20 (2) 191-195. **Keywords:** *Brief Psychotherapy/*Dual Diagnosis/*Interviewing/*Intrinsic Motivation/*Motivation Training/drug abuse/Mental Disorders/Motivational Interviewing.

RefID:3506. Becker, Les R., Hall, Margruetta, Fisher, Deborah A., and Miller, Ted R.. Methods for evaluating a mature substance abuse prevention/early intervention program. The journal of behavioral health services & research 2000. 27 (2) 166-177. **Keywords:** *Business Organizations/*Drug Abuse Prevention/*early intervention/*Employee Assistance Programs/Health Care Psychology/managed care/Program Evaluation.

RefID:3517. Lang, Ernie, Engelander, Marnie, and Brooke, Tracey. Report of an integrated brief intervention with self-defined problem cannabis users. Journal of substance abuse treatment 2000. 19 (2) 111-116. **Keywords:** *Brief Psychotherapy/*Cannabis/*drug abuse/*Treatment Outcomes/Treatment Effectiveness Evaluation.

RefID:3532. Fuger, Kathryn Lu Magnuson. Defining the relationship between the perceived social support of cocaine-using mothers and two case management approaches. (prenatally exposed, family focused intervention). Dissertation Abstracts International Section A: Humanities and Social Sciences 1999. 60 (5-A) 1767-#End Page#. **Keywords:** *Case Management/*Cocaine/*Mothers/*Prenatal Exposure/*Social Support.

RefID:3584. Ligon, Jan Howard. Crisis psychiatric and substance abuse services: Evaluation of a community program in an urban setting. Dissertation Abstracts International Section A: Humanities and Social Sciences 1997. 58 (3-A) 1093-#End Page#. **Keywords:** *Community Mental Health Services/*Crisis Intervention/*Drug Rehabilitation/Client Satisfaction/Psychiatric Symptoms/Treatment Outcomes/Urban Environments.

RefID:3602. Greber, Roberta A., Allen, Karen M., Soeken, Karen L., and Solounias, Bernadette L.. Outcome of trauma patients after brief intervention by a substance abuse consultation service. The American Journal on Addictions 1997. 6 (1) 38-47. **Keywords:** *Drug Abuse Prevention/*Injuries/*Psychotherapeutic Outcomes/Followup Studies/Treatment Outcomes.

RefID:4451. Rounsaville, B. J., Kosten, T. R., Weissmann, M. M., and Kleber, H. D.. A 2.5-year follow-up of short-term interpersonal psychotherapy in methadone-maintained opiate addicts. Comprehensive psychiatry 1986. 27 (3) 201-210. **Keywords:** Central/Combined Modality Therapy/Follow-Up Studies/Humans/Methadone: therapeutic use/Opioid-Related Disorders: rehabilitation: therapy/Psychotherapy,Brief/Research Support,U.S.Gov't,P.H.S..

RefID:4730. Moadel, Y., Schnee, J., and Rubins, J. L.. The role of psychotherapy in the medical management of acute psychiatric problems. American journal of psychoanalysis 1973. 33 (1) 80-93. **Keywords:** Central/Acute Disease/Affective Symptoms [therapy]/Anxiety/Attitude of Health Personnel/Attitude to Health/Crisis Intervention/Defense Mechanisms/Depression [etiology]/Mental Disorders [therapy]/Personality/Physician-Patient Relations/Placebos/Psychophysiologic Disorders [therapy]/Psychotherapy/Self Concept/Stress,Psychological/Unconscious (Psychology)/Humans.

RefID:4757. Eick, T. J. and Kofoed, L.. An unusual indication for a single-subject clinical trial. The Journal of nervous and mental disease 1994. 182 (10) 587-590. **Keywords:** Central/1-Naphthylamine [analogs & derivatives] [therapeutic use]/Clinical Trials as Topic [methods]/Combined Modality Therapy/Cross-Over Studies/Depressive Disorder [drug therapy] [psychology] [therapy]/Physician-Patient Relations/Placebos/Psychotherapy/Self Concept/Serotonin Uptake Inhibitors [therapeutic use]/Sertraline/Transference (Psychology)/Treatment Outcome/Humans/Male/Middle Aged.

RefID:5159. Evenson, R. C., Binner, P. R., Cho, D. W., Schicht, W. W., and Topolski, J. M.. An outcome study of Missouri's CSTAR alcohol and drug abuse programs. Journal of substance abuse treatment 1998. 15 (2) 143-150. **Keywords:** Central/Activities of Daily Living/Adolescent/Alcoholism [psychology] [rehabilitation]/Consumer Satisfaction/Crime/Employment/Missouri/Parenting/Psychiatric Status Rating Scales/Questionnaires/Sampling Studies/Substance-Related Disorders [psychology] [rehabilitation]/Treatment Outcome/Adult/Female/Humans/Male.

RefID:5576. Westerman, M. A., Foote, J. P., and Winston, A.. Change in coordination across phases of psychotherapy and outcome: two mechanisms for the role played by patients' contribution to the alliance. Journal of consulting and clinical psychology 1995. 63 (4) 672-675. **Keywords:** Central/Mental Disorders [therapy]/Psychotherapy/Treatment Outcome/Adult/Female/Humans/Male/Middle Aged.

RefID:5590. Hawthorne, G., Garrard, J., and Dunt, D.. Does Life Education's drug education programme have a public health benefit?. Addiction (Abingdon, England) 1995. 90 (2) 205-215. **Keywords:** Central/Alcohol Drinking [epidemiology] [prevention & control]/Analgesics/Australia [epidemiology]/Cross-Sectional Studies/Health Education/Incidence/Sex Factors/Smoking [epidemiology] [prevention & control]/Substance-Related Disorders [epidemiology] [prevention & control]/Child/Female/Humans/Male.

RefID:5670. Tunis, S. L., Delucchi, K. L., Schwartz, K., Banys, P., and Sees, K. L.. The relationship of counselor and peer alliance to drug use and HIV risk behaviors in a six-month methadone detoxification program. Addictive behaviors 1995. 20 (3) 395-405. **Keywords:** Central/HIV Seropositivity [transmission]/Metabolic Detoxication,Drug/Methadone [therapeutic use]/Peer Group/Professional-Patient Relations/Risk-Taking/Sexual Behavior/Substance-Related Disorders [drug therapy]/Treatment Outcome/Humans.

RefID:5733. Rowan-Szal GA, Simpson DD. Contingency management and treatment engagement in a sample of cocaine-using methadone patients. Proceedings of the 62nd Annual Scientific Meeting of the College on problems of Drug Dependence; 2000 June 17 22; San Juan, Puerto Rico; USA 2001. #volume# (#Issue#) S189-#End Page#. **Keywords:** Central.

RefID:6007. Lang, E., Engelander, M., and Brook, T.. Report of an integrated brief intervention with self-defined problem cannabis users. Journal of substance abuse treatment 2000. 19 (2) 111-116. **Keywords:** Central.

RefID:7042. Penny, Robyn and Pratt, Jan. The trial and evaluation of a clinical pathway for parents with substance use issues. Neonatal, Paediatric & Child Health Nursing 2011. 14 (3) 14-20. **Keywords:** CINAHL/Child Health Services/Children of Impaired Parents/Critical Path/substance abuse/Audit/Australia/convenience sample/Descriptive Statistics/Female/Focus Groups/human/Infant/Male/Parent-Infant Relations/Parenting/Prospective Studies/Quasi-Experimental Studies.

RefID:7056. Alessi, S. M., Rash, C., and Petry, N. M.. Contingency management is efficacious and improves outcomes in cocaine patients with pretreatment marijuana use. Drug & Alcohol Dependence 1-10-2011. 118 (1) 62-67. **Keywords:** CINAHL/Behavior Therapy -- Methods/Substance Use Disorders -- Rehabilitation/Smoking -- Psychosocial Factors/Substance Abuse Detection/Adult/Ambulatory Care/Central Nervous System Stimulants -- Urine/Cocaine -- Urine/Substance Use Disorders -- Diagnosis/Substance Use Disorders -- Therapy/Female/Prospective Studies/human/Male/Smoking -- Urine/Psychological Tests/Reinforcement (Psychology)/Substance Use Disorders -- Urine/Time Factors/Treatment Outcomes/Clinical Trials.

RefID:7059. Steiker, Lori, Powell, Tara, Goldbach, Jeremy, and Hopson, Laura. Dissonance-Based Interventions for Substance Using Alternative High School Youth. Practice (09503153) 2011. 23 (4) 235-252. **Keywords:** CINAHL/Substance Abuse -- Therapy -- In Adolescence/Consumer Participation/Substance Abuse -- Prevention and Control -- In Adolescence/human/Funding Source/adolescence/Treatment Outcomes/Curriculum Development/Substance Abusers/Community Health Services/Institute of Medicine (U.S.)/Substance Abuse -- Risk Factors/Preventive Health Care/Students,High School/juvenile delinquency/Schools,Secondary/conceptual framework/Cognitive Dissonance/Transtheoretical Stages of Change Model/Professional Practice,Evidence-Based/Hardiness/Texas/Multicenter Studies/Life Experiences/Risk Assessment/Ethnographic Research/Quantitative Studies/Surveys/Survey Research/videorecording/Attitude/Health Beliefs/Descriptive Statistics/Questionnaires/Internal Consistency/Coefficient Alpha/Focus Groups/Qualitative Studies/Social Workers/Coding/Professional Role/Social Work Practice -- Trends.

RefID:7065. Fitzpatrick, Ben and Cook, Neal. Empowering decision-making in cannabis use in multiple sclerosis. British Journal of Neuroscience Nursing 2011. 7 (6) 707-712. **Keywords:** CINAHL/Cannabis -- Therapeutic Use/Multiple Sclerosis -- Drug Therapy/Decision

Making,Patient/Empowerment/Middle Age/Male/Coping/Multiple Sclerosis -- Psychosocial Factors/patient education/Cannabis -- Adverse Effects/Privacy and Confidentiality/Motivational Interviewing/Consent/professional development/Psychiatric Nursing/Health Beliefs.

RefID:7131. O'Malley, P. A.. Prescription opioid abuse update for the clinical nurse specialist. Clinical Nurse Specialist: The Journal for Advanced Nursing Practice 2012. 26 (1) 19-21. **Keywords:** CINAHL/Advanced Nursing Practice/Clinical Nurse Specialists/Drugs,Prescription/Narcotics/Pain -- Drug Therapy/Substance Abuse -- Epidemiology -- United States/Substance Abuse -- Prevention and Control/adolescence/Adult/Drug and Narcotic Control/Emergency Care -- Utilization/Narcotics -- Poisoning/Overdose -- Mortality/patient education/Substance Abuse -- Risk Factors/United States.

RefID:7132. Denisco, Richard, Kenna, George, O'Neil, Michael, Kulich, Ronald, Moore, Paul, Kane, William, Mehta, Noshir, Hersh, Elliot, V, and Katz, Nathaniel. Prevention of prescription opioid abuse: The role of the dentist. Journal of the American Dental Association (JADA) 2011. 142 (7) 800-810. **Keywords:** CINAHL/Drugs,Prescription/Narcotics/Substance Abuse -- Prevention and Control/Dentists/Professional Role/Education,Continuing (Credit)/Prescriptions,Drug.

RefID:7192. Korthuis, P., Saha, Somnath, Chander, Geetanjali, McCarty, Dennis, Moore, Richard, Cohn, Jonathan, Sharp, Victoria, and Beach, Mary. Substance Use and the Quality of Patient-Provider Communication in HIV Clinics. AIDS & Behavior 2011. 15 (4) 832-841. **Keywords:** CINAHL/substance abuse/Communication/HIV Infections/Professional-Patient Relations/human/Interviews/Audiorecording/Bivariate Statistics/Linear Regression/Poisson Distribution/Multivariate Analysis/chi square test/Data Analysis Software/Male/Female/Adult/Middle Age/Odds Ratio/Funding Source/Center for Epidemiological Studies Depression Scale/Clinical Assessment Tools/Scales.

RefID:7227. Chandler, D.. Fidelity and outcomes in six integrated dual disorders treatment programs. Community mental health journal 2011. 47 (1) 82-89. **Keywords:** CINAHL/Mental Disorders -- Rehabilitation/Substance Use Disorders -- Rehabilitation/Diagnosis,Dual (Psychiatry)/Professional Practice,Evidence-Based/Program Evaluation/Instrument Validation/California/Regression/Outcomes (Health Care)/Scales/Descriptive Statistics.

RefID:7262. Miles, Maureen, Francis, Karen, and Chapman, Ysanne. Challenges for midwives: pregnant women and illicit drug use. Australian Journal of Advanced Nursing 2010. 28 (1; The purpose of the paper is to introduce illicit drug use as a societal problem and describes the response of the Australian Government. Specifically the paper examines the use of illicit drugs by pregnant women and the role of midwives in supporting these women throughout pregnancy and birth
; Setting
; Maternity services, specifically antenatal care clinics
; Conclusion
; In Australia the rate of pregnant women who use illicit drugs is escalating. These pregnancies

are high obstetric risk with potential for harm to both the mother and the baby. Pregnancy however is seen as 'window of opportunity'; a time to provide education, choices and support. The literature describes that for health professionals working with pregnant women who are illicit drug users is challenging and for some health professionals their interaction can be negative. Australia advocates harm minimisation and encourages harm reduction strategies. Midwives are in a position to implement these strategies within the maternity setting. Further research is recommended as well as professional development programs for midwives to upgrade knowledge and cultivate engagement skills to enable appropriate and positive interaction with pregnant women who use illicit drugs
) 83-90. **Keywords:** CINAHL/Midwifery/Prenatal Care/Substance Abuse,Perinatal/Attitude of Health Personnel/Australia/Female/Pregnancy/Pregnancy Outcomes/Substance Abuse -- Epidemiology -- Australia.

RefID:7284. Ager, R., Roahen-Harrison, S., Toriello, P. J., Kissinger, P., Morse, P., Morse, E., Carney, L., and Rice, J.. Predictors of adopting motivational enhancement therapy. Research on Social Work Practice 2011. 21 (1) 65-76. **Keywords:** CINAHL/Counselors/Drug Rehabilitation Programs -- Manpower/Motivational Interviewing -- Utilization/Diffusion of Innovation/human/Professional Practice,Evidence-Based/Predictive Validity/Surveys/Descriptive Statistics/Step-Wise Multiple Regression/Social Worker Attitudes/professional knowledge/Age Factors/Cultural Competence/Self-Efficacy/Workload/Organizational Structure/Organizational Objectives/Motivational Interviewing -- Education/Random Assignment/control group/Pretest-Posttest Design/Linear Regression/Logistic Regression/Funding Source.

RefID:7352. Doran, D., Paterson, J., Clark, C., Srivastava, R., Goering, P. N., Kushniruk, A. W., Bajnok, I., Nagle, L., Almost, J., and Carryer, J.. A pilot study of an electronic interprofessional evidence-based care planning tool for clients with mental health problems and addictions. Worldviews on Evidence-Based Nursing 2010. 7 (3) 174-184. **Keywords:** CINAHL/Instrument Validation/Patient Centered Care -- Evaluation/Professional Practice,Evidence-Based/Psychiatric Patients/Schizophrenia -- Therapy/Substance Abusers/Activities of Daily Living/Aggression/Allied Health Personnel/conceptual framework/construct validity/convenience sample/correlation coefficient/Depression/Descriptive Statistics/Diffusion of Innovation/Funding Source/Health Services Accessibility/Hospitals,Psychiatric/human/Inpatients/Interprofessional Relations/Interviews/Models,Theoretical/Nonprobability Sample/Observational Methods/outcome assessment/Pain/Paired T-Tests/Physicians/Pilot Studies/Pretest-Posttest Design/professional knowledge/Psychiatric Nursing/Questionnaires/Scales/Schizophrenia -- Symptoms/Semi-Structured Interview/substance abuse.

RefID:7353. Hennessy, K. D. and Green-Hennessy, S.. A Review of Mental Health Interventions in SAMHSA's National Registry of Evidence-Based Programs and Practices. Psychiatric Services 2011. 62 (3) 303-305. **Keywords:** CINAHL/Professional Practice,Evidence-Based/Mental Health Services/Data Collection/Substance Abuse and Mental Health Services Administration/human/United States.

RefID:7369. Polcin, D. L., Korcha, R., Bond, J., and Galloway, G.. Eighteen-month outcomes for clients receiving combined outpatient treatment and sober living houses. Journal of Substance Use 2010. 15 (5) 352-366. **Keywords:** CINAHL/Outcomes (Health Care)/Residential Facilities/Substance Use Rehabilitation Programs/Analysis of Variance/Brief Symptom Inventory/California/Checklists/Clinical Assessment Tools/Confidence Intervals/Descriptive Statistics/Educational Status/employment status/Ethnic Groups/Female/human/Male/Marital Status/Odds Ratio/outcome assessment/Outpatients/P-Value/Prospective Studies/Repeated Measures/Severity of Illness Indices/Social Networks/structured interview/substance abuse/Support Groups.

RefID:7375. Heun, D.. Is it noncompliance, or are the barriers insurmountable?. Professional case management 2010. 15 (5) 290-293. **Keywords:** CINAHL/Diabetes Mellitus -- Prevention and Control/Health Services Accessibility/Patient Compliance/Self Care/Adult/Alcoholism/Burnout,Professional/Case Management/Depression/Female/Gastroparesis/homelessness/Male/Mental Disorders/patient assessment/Readmission/Substance Dependence.

RefID:7486. Schonfeld, L., King-Kallimanis, B. L., Duchene, D. M., Etheridge, R. L., Herrera, J. R., Barry, K. L., and Lynn, N.. Screening and brief intervention for substance misuse among older adults: the Florida BRITE project. American journal of public health 2010. 100 (1) 108-114. **Keywords:** CINAHL/Health Screening -- In Middle Age/Health Screening -- In Old Age/Substance Use Disorders -- Diagnosis -- In Middle Age/Substance Use Disorders -- Diagnosis -- In Old Age/Substance Use Disorders -- Therapy -- In Middle Age/Substance Use Disorders -- Therapy -- In Old Age/Aged/Alcoholism -- Diagnosis -- In Middle Age/Alcoholism -- Diagnosis -- In Old Age/Analysis of Variance/Clinical Assessment Tools/Data Analysis Software/Descriptive Statistics/Female/Florida/Geriatric Depression Scale/human/Interviews/Male/Middle Age/Paired T-Tests/Pilot Studies/post hoc analysis/Program Evaluation/Psychological Tests/Psychotherapy,Brief/Repeated Measures/Scales/Substance Dependence -- Diagnosis -- In Middle Age/Substance Dependence -- Diagnosis -- In Old Age/Two-Tailed Test.

RefID:7516. Tran, D. T., Stone, A. M., Fernandez, R. S., Griffiths, R. D., and Johnson, M.. Does implementation of clinical practice guidelines change nurses' screening for alcohol and other substance use?. Contemporary Nurse: A Journal for the Australian Nursing Profession 2009. 33 (1) 13-19. **Keywords:** CINAHL/Health Screening/Medical-Surgical Nursing/Nursing Practice/Nursing Staff,Hospital/Practice Guidelines/Substance Abuse -- Prevention and Control/alcohol abuse/chi square test/Clinical Assessment Tools/Data Analysis Software/Descriptive Statistics/Hospital Policies/Hospitals,Urban/Inpatients/New South Wales/Nursing Assessment/Nursing Audit/Nursing Interventions/Nursing Units/P-Value/Patient Admission/Patient History Taking/Pretest-Posttest Design/Record Review/Referral and Consultation/Smoking/Staff Development/human.

RefID:7608. Sigmon, S. C., Dunn, K. E., Badger, G. J., Heil, S. H., and Higgins, S. T.. Brief buprenorphine detoxification for the treatment of prescription opioid dependence: a pilot

study. Addictive behaviors 2009. 34 (3) 304-311. **Keywords:** CINAHL/Buprenorphine -- Therapeutic Use/Drugs,Prescription -- Adverse Effects/Naltrexone -- Therapeutic Use/Narcotic Antagonists -- Therapeutic Use/Substance Use Disorders -- Rehabilitation/Adult/Behavior Therapy/Epidemiological Research/Female/Male/Pilot Studies/Substance Abuse Detection/Substance Use Disorders -- Urine/Time Factors/human.

RefID:7618. . In brief. Nurse Prescribing 2011. 9 (11) 528-528. **Keywords:** CINAHL/Dermatology Nursing/Narcotics/Nurse Practitioners/Office Nursing/Practice Guidelines/Prescriptive Authority/Professional Compliance/Psoriasis -- Therapy/United Kingdom.

RefID:7639. Middleton, Jenni. Role model. Addicted to equality. Nursing Times 31-5-2011. 107 (21) 24-25. **Keywords:** CINAHL/Clinical Nurse Specialists/Substance Use Disorders -- Nursing/Nurse Attitudes/Role Models/Staff Development.

RefID:7655. Gaylin, W.. Behavior control: from the brain to the mind. Hastings Center Report 2009. 39 (3) 13-16. **Keywords:** CINAHL/Behavior/Control (Psychology)/Ethics,Medical/Autonomy/Child Behavior Disorders -- Drug Therapy/electroconvulsive therapy/Methylphenidate -- Therapeutic Use/Professional Organizations/Psychiatry -- Methods/Psychosurgery.

RefID:7689. Winters, K. C.. Screening and assessing adolescents for substance use disorders. #journal name# 2001. #volume# (#Issue#) #Start Page#-136. **Keywords:** CINAHL/Substance Use Disorders -- Risk Factors -- In Adolescence/Substance Use Disorders -- Diagnosis -- In Adolescence/Substance Use Disorders -- Prevention and Control -- In Adolescence/Research Instruments/Child/adolescence/Adult.

RefID:7722. Marvel, F., Rowe, C. L., Colon-Perez, L., DiClemente, R. J., and Liddle, H. A.. Multidimensional family therapy HIV/STD risk-reduction intervention: an integrative family-based model for drug-involved juvenile offenders. Family process 2009. 48 (1) 69-84. **Keywords:** CINAHL/Family Therapy -- Methods/HIV Infections -- Prevention and Control -- In Adolescence/Juvenile Offenders -- Psychosocial Factors/Sexually Transmitted Diseases -- Prevention and Control -- In Adolescence/Substance Abusers/adolescence/Communication/Cultural Competence/Cultural Sensitivity/Female/Male/Mothers -- Psychosocial Factors/Parent-Child Relations/Professional Practice,Evidence-Based/Program Development/Protocols/Risk Taking Behavior -- Prevention and Control/Vignettes.

RefID:7790. Swan, M., Schwartz, S., Berg, B., Walker, D., Stephens, R., and Roffman, R.. The teen marijuana check-up: an in-school protocol for eliciting voluntary self-assessment of marijuana use. Journal of Social Work Practice in the Addictions 2008. 8 (3) 284-302. **Keywords:** CINAHL/Behavior Therapy -- In Adolescence/Cannabis/Self Assessment -- In Adolescence/Substance Abuse -- Therapy -- In Adolescence/adolescence/Behavioral Changes/Clinical Trials/Counseling/Feedback/Funding Source/Interviews/Male/Motivational Interviewing/Pretest-Posttest Design/Privacy and

Confidentiality/Protocols/Questionnaires/Random Assignment/School Health Services/Smoking/Substance Abuse -- Prevention and Control/trust/human.

RefID:7854. Petersen, T.. Advising young people about drugs and alcohol. Practice Nursing 2008. 19 (6) 287-291. **Keywords:** CINAHL/Substance Abuse -- In Adolescence/adolescence/alcohol abuse/Child/Female/Health Screening/Information Resources/Male/Motivation/Office Nursing/Referral and Consultation/Self Care/Social Identity/Street Drugs/Substance Abuse -- Prevention and Control/World Wide Web.

RefID:7964. Burton-Macleo, S. and Fainsinger, R. L.. Cancer pain control in the setting of substance use: establishing goals of care. Journal of Palliative Care 2008. 24 (2) 122-125. **Keywords:** CINAHL/Analgesics,Opioid -- Therapeutic Use/Carcinoma,Non-Small-Cell Lung -- Complications/Lung Neoplasms -- Complications/Pain -- Drug Therapy/Palliative Care/Substance Use Disorders -- Complications/Analgesics,Opioid -- Adverse Effects/Carcinoma,Non-Small-Cell Lung -- Therapy/Lung Neoplasms -- Therapy/Male/Middle Age/Nursing Care Plans/Patient Compliance/Professional-Patient Relations/Substance Use Disorders -- Rehabilitation.

RefID:7980. Neushotz, L. A. and Fitzpatrick, J. J.. Improving substance abuse screening and intervention in a primary care clinic. Archives of psychiatric nursing 2008. 22 (2) 78-86. **Keywords:** CINAHL/Health Knowledge/Outpatient Service -- Administration/Primary Health Care -- Administration/Substance Abuse Detection/Substance Use Disorders -- Education/Substance Use Disorders -- Therapy/Academic Medical Centers/Addictions Nursing/Descriptive Statistics/Diffusion of Innovation/Focus Groups/Health Personnel/New York/Process Assessment (Health Care)/Self Report/human.

RefID:8052. Agley, J. and Gassman, R.. A state-based model of prevention: Indiana's example. Health Promotion Practice 2008. 9 (2) 199-204. **Keywords:** CINAHL/Preventive Health Care/Public Health/Substance Abuse -- Prevention and Control -- In Adolescence/adolescence/Child/Community Programs/Health Personnel/Indiana.

RefID:8084. Martin, L. M., Bliven, M., and Boisvert, R.. Occupational performance, self-esteem, and quality of life in substance addictions recovery. OTJR: Occupation, Participation & Health 2008. 28 (2) 81-88. **Keywords:** CINAHL/Activities of Daily Living/Functional Assessment -- Methods/occupational therapy/Recovery/Self Concept/substance abuse/adolescence/Adult/Blacks/Clinical Assessment Tools/Coefficient Alpha/Data Analysis Software/Female/Hispanics/Male/Mental Disorders/Middle Age/Native Americans/Paired T-Tests/Pretest-Posttest Design/residential care/Rosenberg Self Esteem Scale/Semi-Structured Interview/Summated Rating Scaling/Whites/Wilcoxon Rank Sum Test/human.

RefID:8086. Wilburn, S. T., Wilburn, K. T., Weaver, D. M., and Bowles, K.. Pearls and Pitfalls in Evaluating a Student Assistance Program: A Five-Year Case Study. Journal of drug education 2007. 37 (4) 447-467. **Keywords:** CINAHL/Health Education -- Administration/School Health Services -- Administration/Substance Use Disorders -- Prevention and Control/Substance Use

Disorders -- Therapy/adolescence/Attitude to Health/Child/Demography/Female/Male/Minority Groups/Program Evaluation/Quality of Health Care/Risk Factors.

RefID:8178. . New approaches seek to expand naltrexone use in heroin treatment. Nida Notes 2003. 17 (6) 8-10. **Keywords:** CINAHL/Heroin/Naltrexone -- Therapeutic Use/Substance Dependence -- Drug Therapy.

RefID:8183. Kaminer, Y.. Pharmacotherapy for adolescents with psychoactive substance use disorders. #journal name# 1995. #volume# (#Issue#) 291-324. **Keywords:** CINAHL/Substance Abuse -- Drug Therapy -- In Adolescence/Diagnosis,Dual (Psychiatry) -- In Adolescence/Affective Disorders/Anxiety Disorders/adolescence.

RefID:8189. Talbert, J. J.. Substance abuse among nurses. Clinical Journal of Oncology Nursing 2009. 13 (1) 17-19. **Keywords:** CINAHL/Impairment,Health Professional/Nurses/substance abuse/Substance Abuse -- Symptoms/work environment.

RefID:8231. . Preventing drug use among children and adolescents: a research-based guide. #journal name# 1997. #volume# (#Issue#) #Start Page#-38. **Keywords:** CINAHL/Substance Abuse -- Prevention and Control/Child/adolescence.

RefID:8239. James, Philip. The Evidence-Based Guide to Antipsychotic Medications. Journal of Psychiatric & Mental Health Nursing 2011. 18 (2) e4-e4. **Keywords:** CINAHL/Antipsychotic Agents.

RefID:8241. Meadows, M.. The FDA's drug review process: ensuring drugs are safe and effective. FDA Consumer 2002. 36 (4) 19-24. **Keywords:** CINAHL/United States Food and Drug Administration/Drug Approval.

RefID:8243. D'Onofrio, C. N.. The prevention of alcohol use by rural youth. #journal name# 1997. #volume# (#Issue#) 250-363. **Keywords:** CINAHL/Alcohol Drinking -- Prevention and Control/Environment/School Health Education/Prevalence/Risk Factors/Health Promotion/Program Development/Alcohol Drinking -- Epidemiology/Demography/Rural Areas/Child/adolescence.

RefID:8245. Stathis, S., Letters, P., Dacre, E., Doolan, I., Health, K., and Litchfield, B.. The role of an Indigenous Health Worker in contributing to equity of access to a mental health and substance abuse service for Indigenous young people in a youth detention centre. Australian e-Journal for the Advancement of Mental Health 2007. 6 (1) 1-10. **Keywords:** CINAHL/Health Services Accessibility -- Australia/Indigenous Peoples -- Australia/Juvenile Offenders -- Australia/Mental Health Services -- Australia/Substance Use Disorders -- Therapy/Australia/chi square test/Health Services Research/Mental Health Personnel/Referral and Consultation/human.

RefID:8253. Grabowski, J., Arnoni, G., Elk, R., Rhoades, H., and Schmitz, J.. Baseline assessment, study entry, and stabilization: double-blind clinical trials in drug dependence. #journal name# 1997. #volume# (#Issue#) 158-181. **Keywords:** CINAHL/Clinical Trials -- Methods/psychopharmacology/Substance Abuse Detection/Research Subject Recruitment/substance abuse.

RefID:8294. . Treating tobacco use and dependence. Kansas Nurse 2001. 76 (9) 1-8. **Keywords:** CINAHL/tobacco/Smoking Cessation Programs/Practice Guidelines/Motivation/Counseling/nicotine replacement therapy.

RefID:8297. Knapp, M.. Webhealth topics. Post-Katrina stress disorder. Journal of Consumer Health on the Internet 2007. 11 (2) 61-73. **Keywords:** CINAHL/Information Resources/Natural Disasters/Stress,Psychological/World Wide Web/Consumer Health Information/Mental Disorders.

RefID:8298. Taylor, H., Stuttaford, M., Broad, B., and Vostanis, P.. Why a 'roof' is not enough: the characteristics of young homeless people referred to a designated mental health service. Journal of Mental Health 2006. 15 (4) 491-501. **Keywords:** CINAHL/Health Services Accessibility/Health Services Needs and Demand/Homelessness -- Psychosocial Factors/Mental Disorders -- Prevention and Control/Mental Health/Mental Health Services -- Utilization/Referral and Consultation/adolescence/Adult/Anxiety/Checklists/Clinical Assessment Tools/content analysis/Cross Sectional Studies/Data Analysis Software/Depression/descriptive research/Descriptive Statistics/Female/Funding Source/Male/Mental Disorders -- Symptoms/National Health Programs/Psychological Tests/Research,Mental Health/Risk Assessment/Risk Factors/Scales/Severity of Illness/Stress Disorders,Post-Traumatic/United Kingdom/human.

RefID:8310. Des Jarlais, D. C., Sloboda, Z., Friedman, S. R., Tempalski, B., McKnight, C., and Braine, N.. Diffusion of the D.A.R.E and syringe exchange programs. American journal of public health 2006. 96 (8) 1354-1358. **Keywords:** CINAHL/Community Programs -- Evaluation/Diffusion of Innovation/Health Education -- Methods/HIV Infections -- Prevention and Control/Needle Exchange Programs/Substance Abuse -- Prevention and Control -- In Adolescence/adolescence/Child/Community Programs -- History/Decision Making,Organizational/Financing,Government/Professional Practice,Evidence-Based/Program Development/Public Health.

RefID:8315. Liddle, H. A. and Dakof, G. A.. Family-based treatment for adolescent drug use: state of the science. #journal name# 1995. #volume# (#Issue#) 218-254. **Keywords:** CINAHL/Substance Abuse -- Therapy -- In Adolescence/Family Therapy/adolescence.

RefID:8337. Pentz, M. A.. Preventing drug abuse through the community: multicomponent programs make the difference. #journal name# 1998. #volume# (#Issue#) 73-86. **Keywords:** CINAHL/Substance Abuse -- Prevention and Control -- In Adolescence/Substance Abuse -- Education -- In Adolescence/Community Programs/Health

Education/Models,Theoretical/Education,Theory-Based/Literature Review/Students,Middle School/Students,High School/random sample/Kansas/Indiana/Prospective Studies/Surveys/Experimental Studies/School Health Education/Parents -- Education/Questionnaires/Repeated Measures/Linear Regression/Logistic Regression/Causal Modeling/Blacks/Whites/Child/adolescence/human.

RefID:8411. Stubben, J.. Culturally competent substance abuse prevention research among rural Native American communities. #journal name# 1997. #volume# (#Issue#) 459-483. **Keywords:** CINAHL/Substance Abuse -- Prevention and Control/Rural Areas/Native Americans -- United States/Community Health Services/United States/Research Methodology.

RefID:8475. Shumaker, R. and Hickey, P.. Patient safety first. Medication diversion in the perioperative setting. AORN Journal 2006. 83 (3) 745-#End Page#. **Keywords:** CINAHL/Drugs,Prescription/Operating Room Personnel/patient safety/substance abuse/Theft/Drug and Narcotic Control/Impairment,Health Professional/Perioperative Nursing/Theft -- Prevention and Control.

RefID:8491. Mueller, M. D. and Wyman, J. R.. Study sheds new light on the state of drug abuse treatment nationwide. Nida Notes 1997. 12 (5) 1-#End Page#. **Keywords:** CINAHL/Substance Abuse -- Prevention and Control/Drug Rehabilitation Programs/Treatment Outcomes/Research.

RefID:8543. Stimson, G. V., Fitch, C., Des Jarlais, D., Poznyak, V., Perlis, T., Oppenheimer, E., and Rhodes, T.. Scaling up promising interventions. Rapid assessment and response studies of injection drug use: knowledge gain, capacity building, and intervention development in a multisite study. American journal of public health 2006. 96 (2) 288-295. **Keywords:** CINAHL/Health and Welfare Planning -- Methods/Multimethod Studies/Needs Assessment -- Methods/Needs Assessment -- Standards/Public Health Administration/substance abuse/World Health Organization -- Standards/Community Role/Evaluation Research/Funding Source/Information Management/naturalistic inquiry/Organizational Development/outcome assessment/Planning Techniques/Practice Guidelines/professional knowledge/Program Development/Prospective Studies/Questionnaires/Reports/Research Personnel/Thematic Analysis/World Health/human.

RefID:8598. Glider, P., Hughes, P., Mullen, R., Coletti, S., Sechrest, L., Neri, R., Renner, B., and Sicilian, D.. Two therapeutic communities for substance-abusing women and their children. #journal name# 1996. #volume# (#Issue#) 32-51. **Keywords:** CINAHL/Substance Abuse -- Rehabilitation/Drug Rehabilitation Programs/Socioenvironmental Therapy/Program Development/Mother-Child Relations/Clinical Trials/Pregnancy/Female.

RefID:8601. Fok, M. S. and Wong, D. Y.. A pilot study on enhancing positive coping behaviour in early adolescents using a school-based project. Journal of Child Health Care 2005. 9 (4) 301-313. **Keywords:** CINAHL/Coping -- In Adolescence/Stress,Psychological -- In Adolescence/adolescence/Audiorecording/Child/content analysis/Diaries/Female/Focus Groups/Hong Kong/Interrater Reliability/Male/Parents/Schools,Elementary/Semi-Structured

Interview/human.

RefID:8658. Chasnoff, I. J., Marques, P. R., Strantz, I. H., Farrow, J., and Davis, S.. Building bridges: treatment research partnerships in the community. #journal name# 1996. #volume# (#Issue#) 6-21. **Keywords:** CINAHL/Substance Abuse,Perinatal -- Therapy/Community Health Services/Health Services Accessibility/Drug Rehabilitation Programs/Program Development/Research/Pregnancy/Female.

RefID:8702. Boyd, M. R., Moneyham, L., Murdaugh, C., Phillips, K. D., Tavakoli, A., Jackwon, K., Jackson, N., and Vyavaharkar, M.. A peer-based substance abuse intervention for HIV+ rural women: a pilot study. Archives of psychiatric nursing 2005. 19 (1) 10-17. **Keywords:** CINAHL/Peer Counseling/Substance Abusers -- Psychosocial Factors/Substance Use Disorders -- Psychosocial Factors/Substance Use Disorders -- Rehabilitation/Women/Attitude to Change/Center for Epidemiological Studies Depression Scale/Descriptive Statistics/Female/Interviews/Lazarus Theory of Stress and Coping/Models,Psychological/Motivation/Pilot Studies/Pretest-Posttest Design/Psychological Tests/Questionnaires/Rural Areas/Sample Size/Scales/Sensitivity and Specificity/human.

RefID:8795. Stephens, R. S., Roffman, R. A., Fearer, S. A., Williams, C., Picciano, J. F., and Burke, R. S.. The marijuana check-up: reaching users who are ambivalent about change. Addiction (Abingdon, England) 2004. 99 (10) 1323-1332. **Keywords:** CINAHL/Cannabis/Motivation/Substance Abuse -- Prevention and Control/Substance Abusers/Adult/chi square test/Coefficient Alpha/Experimental Studies/Female/Funding Source/Interview Guides/Interviews/Male/Questionnaires/Random Assignment/Scales/Self Report/T-Tests/Washington/human.

RefID:8850. Hershberger, S. L., Wood, M. M., and Fisher, D. G.. A cognitive-behavioral intervention to reduce HIV risk behaviors in crack and injection drug users. AIDS & Behavior 2003. 7 (3) 229-243. **Keywords:** CINAHL/Cognitive Therapy/Risk Taking Behavior -- Prevention and Control/Substance Abuse -- Rehabilitation/Adult/Asians/Behavior Rating Scales/Blacks/California/Cocaine/conceptual framework/Demography/Descriptive Statistics/Evaluation Research/Funding Source/Hispanics/Interviews/Intravenous Drug Users/Random Assignment/Research Subject Recruitment/Self Report/Sexual Partners/sexuality/Substance Abusers/Substance Use Rehabilitation Programs/Support,Psychosocial/Two-Way Analysis of Variance/videorecording/Whites/human.

RefID:8865. Bohman, T. M., Barker, E. D., Bell, M. L., Lewis, C. M., Holleran, L., and Pomeroy, E.. Early intervention for alcohol use prevention and vehicle safety skills: evaluating the Protecting You/Protecting Me curriculum. Journal of Child & Adolescent Substance Abuse 2004. 14 (1) 17-40. **Keywords:** CINAHL/Curriculum/Motor Vehicles/Program Development/Safety/Attitude to Health/Data Analysis,Statistical/Descriptive Statistics/Evaluation Research/Scales/Texas/Time Factors/Funding Source/human.

RefID:8914. Lind, J., Kouimtsidis, C., Reynolds, M., Hunt, M., Drummond, C., and Ghodse, H..

Drug misuse among patients admitted to a general hospital. Journal of Substance Use 2003. 8 (3) 186-190. **Keywords:** CINAHL/Substance Abuse -- Epidemiology -- England/Academic Medical Centers/Age Factors/chi square test/Data Analysis Software/Descriptive Statistics/Drugs,Non-Prescription/Drugs,Prescription/employment status/England/Epidemiological Research/Female/Fisher's Exact Test/Hypnotics and Sedatives/Inpatients/Interviews/Male/Mann-Whitney U Test/Middle Age/One-Way Analysis of Variance/P-Value/Patient Admission/patient assessment/Prospective Studies/Questionnaires/random sample/Record Review/Self Report/Sex Factors/Street Drugs/Surveys/T-Tests/human.

RefID:8937. Bartu, A., Freeman, N. C., Gawthorne, G. S., Codde, J. P., and Holman, C. D. J.. Psychiatric comorbidity in a cohort of heroin and amphetamine users in Perth, Western Australia. Journal of Substance Use 2003. 8 (3) 150-154. **Keywords:** CINAHL/Diagnosis,Dual (Psychiatry)/Drug Rehabilitation Programs/Mental Health Services -- Utilization/Patient Admission/Substance Abusers/Adult/Age Factors/Amphetamines/Comparative Studies/Confidence Intervals/Counseling/Cox Proportional Hazards Model/Female/Funding Source/Hospitals,Psychiatric/Hospitals,Urban/Inpatients/Male/Mental Disorders/Methadone -- Therapeutic Use/Narcotics/outcome assessment/Outpatients/P-Value/patient assessment/Patient Dropouts/Probability Sample/Prospective Studies/Record Review/Regression/Resource Databases,Health/Sex Factors/Support Groups/Treatment Outcomes/Western Australia/human.

RefID:8945. Scott, R. G. A., Keaney, F., Marshall, E. J., Strang, J., Sinha, J., and Peters, T. J.. The feasibility of substance misuse screening in referrals from accident and emergency to an inner-city fracture clinic: results from a pilot study. Journal of Substance Use 2002. 7 (2) 65-70. **Keywords:** CINAHL/Substance Abuse -- Epidemiology -- England/Referral and Consultation/Ambulatory Care/Prospective Studies/Epidemiological Research/Pilot Studies/Questionnaires/Self Report/Alcohol Abuse -- Epidemiology/Smoking -- Epidemiology/Cannabis/Fractures -- Etiology/Research Instruments/Data Analysis Software/Confidence Intervals/Fisher's Exact Test/T-Tests/One-Way Analysis of Variance/chi square test/Descriptive Statistics/P-Value/Marital Status/Age Factors/Sex Factors/Male/Female/Adult/Outpatients/Outpatient Service/Emergency Service/Hospitals,Urban/England/human.

RefID:8961. Tayar, S. G.. Description of a substance abuse relapse prevention programme conducted by occupational therapy and psychology graduate students in a United States women's prison. British Journal of Occupational Therapy 2004. 67 (4; The psychology students taught the inmates the theoretical bases for the programme, trigger identification and coping skills, social support development and assertive communication. The occupational therapy students used activities to help the inmates to identify their values and interests, develop ideas and resources for leisure and jobs, structure and manage their time, practise assertiveness and identify strengths. Although programme effectiveness was not formally measured, it appeared to both the students and the faculty members that the inmates experienced personal growth and that the student therapists gained professional skills in working with another discipline and

in leading groups
) 159-166. **Keywords:** CINAHL/Recurrence -- Prevention and Control/Drug Rehabilitation Programs -- Methods/occupational therapy/Psychotherapy/Students,Occupational Therapy/Correctional Facilities/Psychological Theory.

RefID:8973. Olfson, M., Tobin, J. N., Cassells, A., and Weissman, M.. Improving the detection of drug abuse, alcohol abuse, and depression in community health centers. Journal of Health Care for the Poor & Underserved 2003. 14 (3) 386-402. **Keywords:** CINAHL/Substance Abuse -- Diagnosis/Depression -- Diagnosis/Alcohol Abuse -- Diagnosis/Community Health Centers/Primary Health Care/New Jersey/New York/Urban Areas/Pilot Studies/Surveys/Sensitivity and Specificity/Predictive Value of Tests/Psychological Tests/Record Review/Sampling Methods/Interviews/T-Tests/chi square test/random sample/Questionnaires/Funding Source/human.

RefID:8978. Wyman, J. R.. Multifaceted prevention programs reach at-risk children through their families. Nida Notes 1997. 12 (3) 5-7. **Keywords:** CINAHL/Substance Abuse -- Prevention and Control/Family Relations/Parents -- Education/Research/Child,Preschool/Child/adolescence/Adult.

RefID:9042. Good, G.. Sick to death. Journal of Clinical Ethics 2006. 17 (1) 80-82. **Keywords:** CINAHL/Empathy/Multidisciplinary Care Team/Paraplegia -- Complications/Pressure Ulcer -- Nursing/Professional-Patient Relations/Terminal Care/Treatment Refusal/Adult/Bacteremia -- Drug Therapy/Bacteremia -- Etiology/Chronic Disease/Family/Female/Pain -- Drug Therapy/Pain -- Etiology/Pain -- Nursing/Paraplegia -- Etiology/Patient Compliance/Pressure Ulcer -- Complications/Pressure Ulcer -- Etiology/Resuscitation Orders/Smoking/Substance Use Disorders -- Etiology/trust.

RefID:9074. Dimond, B.. Legal aspects of medicines. Accountability and medicinal products 4: professional issues. British Journal of Nursing (BJN) 25-3-2004. 13 (6) 337-340. **Keywords:** CINAHL/Committees/Licensure/Malpractice/Medication Errors/Analgesics,Opioid -- Poisoning/Clinical Competence/Female/Heroin -- Poisoning/Social Responsibility/United Kingdom.

RefID:9103. Vaughan, B. L., Levy, S., and Knight, K. R.. Adolescent substance use: prevention and management by primary care clinicians. Journal of Clinical Outcomes Management 2003. 10 (3) 166-175. **Keywords:** CINAHL/Pediatric Care/Primary Health Care/Substance Abuse -- Prevention and Control -- In Adolescence/Substance Abuse -- Therapy -- In Adolescence/adolescence/Education,Continuing (Credit)/Male/Patient History Taking/Physical Examination/Physician-Patient Relations/Research Instruments.

RefID:9127. . Nurse Practice Act -- Oregon revised Statutes (as amended 1995) and Administrative Rules Regulating the Practice of Nursing. Oregon State Board of Nursing. #journal name# 1996. #volume# (#Issue#) various-#End Page#. **Keywords:** CINAHL/Nursing Practice -- Legislation and Jurisprudence -- Oregon/State Boards of Nursing -- Administration --

Oregon/Oregon/Education,Nursing -- Legislation and Jurisprudence -- Oregon/Practical Nurses -- Legislation and Jurisprudence -- Oregon/Registered Nurses -- Legislation and Jurisprudence -- Oregon/Accreditation -- Legislation and Jurisprudence -- Oregon/Schools,Nursing -- Legislation and Jurisprudence -- Oregon/Schools,Nursing -- Administration -- Oregon/Mandatory Reporting -- Legislation and Jurisprudence -- Oregon/Faculty,Nursing -- Legislation and Jurisprudence -- Oregon/Curriculum -- Legislation and Jurisprudence -- Oregon/State Board Examinations -- Legislation and Jurisprudence -- Oregon/NCLEX Examination -- Legislation and Jurisprudence -- Oregon/Licensure,Nursing -- Legislation and Jurisprudence -- Oregon/Foreign Nurses -- Legislation and Jurisprudence -- Oregon/Refresher Courses -- Legislation and Jurisprudence -- Oregon/Scope of Practice -- Legislation and Jurisprudence -- Oregon/Nursing Process -- Legislation and Jurisprudence -- Oregon/Professional Misconduct -- Legislation and Jurisprudence -- Oregon/Jurisprudence -- Legislation and Jurisprudence -- Oregon/Drug Rehabilitation Programs -- Legislation and Jurisprudence -- Oregon/Disabled -- Legislation and Jurisprudence -- Oregon/Delegation of Authority -- Legislation and Jurisprudence -- Oregon/Drug Administration -- Legislation and Jurisprudence -- Oregon/Nurse Practitioners -- Legislation and Jurisprudence -- Oregon/Certification -- Legislation and Jurisprudence -- Oregon/Prescriptive Authority -- Legislation and Jurisprudence -- Oregon/Nursing Assistants -- Legislation and Jurisprudence -- Oregon/Professional Discipline -- Legislation and Jurisprudence -- Oregon/License Revocation -- Legislation and Jurisprudence -- Oregon.

RefID:9170. Clark, J. J., Leukefeld, C., Godlaski, T., Brown, C., Garrity, J., and Hays, L.. Developing, implementing, and evaluating a treatment protocol for rural substance abusers. Journal of Rural Health 2002. 18 (3) 396-406. **Keywords:** CINAHL/Substance Abuse -- Therapy/Rural Health Services -- Evaluation/Substance Use Rehabilitation Programs -- Trends/Kentucky/Semi-Structured Interview/Focus Groups/Step-Wise Multiple Regression/Descriptive Statistics/Thematic Analysis/Adult/Middle Age/Male/Female/Funding Source/human.

RefID:9183. Angres, D. H., McGovern, M. P., Rawal, P., and Shaw, M.. Psychiatric comorbidity and physicians with substance use disorders: clinical characteristics, treatment experiences, and post-treatment functioning. Addictive Disorders & Their Treatment 2002. 1 (3; Treatment of substance use disorders in physicians has become a highly specialized clinical practice. The recognition of comorbid psychiatric disorders for physicians with substance use disorders has been slower to evolve than with the general field of addiction treatment and service research. This study examines the role of psychiatric comorbidity for physicians with substance use disorders along a number of dimensions
) 89-98. **Keywords:** CINAHL/Comorbidity/Substance Use Disorders/Physicians -- Psychosocial Factors/Mental Disorders/Impairment,Health Professional/Severity of Illness/Diagnosis,Dual (Psychiatry)/Retrospective Design/Record Review/Prospective Studies/Surveys/random sample/T-Tests/Pearson's Correlation Coefficient/Interrater Reliability/Questionnaires/Patient Satisfaction/Psychological Tests/Factor Analysis/Length of Stay/Marital Status/employment status/Mental Disorders -- Therapy/Self Report/Substance Use Disorders -- Therapy/Male/Adult/Middle Age/Funding Source/human.

RefID:9184. Woldeguiorguis, I. M.. Racism and sexism in child welfare: effects on women of color as mothers and practitioners. Child Welfare 2003. 82 (2) 273-288. **Keywords:** CINAHL/Child Welfare/Racism/Sexism/Domestic Violence/Massachusetts/Female/Mothers/Adult/substance abuse/Social Workers/child care/Supervisors and Supervision/Interprofessional Relations/Professional-Client Relations/Women,Working.

RefID:9222. . Fluoxetine with CBT effective for comorbid disorders. Brown University Child & Adolescent Psychopharmacology Update 2008. 10 (1; Controlled trial of 126 adolescents (mean age 17 years) with major depressive disorder (MDD), CD and SUD, randomized to fluoxetine (20 mg/day)+CBT (N=63) or placebo+CBT (N=63) for 16 weeks
; Adverse events were mild and transient; however, 4 patients in the fluoxetine+CBT group and one patient in the placebo+ CBT group were hospitalized or evaluated in an emergency department for worsening suicidality
) 5-6. **Keywords:** CINAHL/Behavior Therapy -- In Adolescence/Comorbidity/Depression -- Therapy -- In Adolescence/Fluoxetine -- Therapeutic Use -- In Adolescence/adolescence/Combined Modality Therapy/Depression -- Drug Therapy/Social Behavior Disorders -- Drug Therapy/Social Behavior Disorders -- Therapy/Substance Dependence -- Drug Therapy/Substance Dependence -- Therapy.

RefID:9267. Osorio, R., McCusker, M., and Salazar, C.. Evaluation of a women-only service for substance misusers. Journal of Substance Use 2002. 7 (1) 41-49. **Keywords:** CINAHL/Substance Use Rehabilitation Programs/Women's Health Services/Program Evaluation/Evaluation Research/Questionnaires/Semi-Structured Interview/Patient Satisfaction/Health Status/Health Services Accessibility/Health Resource Utilization/Process Assessment (Health Care)/outcome assessment/Research Instruments/chi square test/T-Tests/P-Value/Sex Factors/Age Factors/Race Factors/Female/adolescence/Adult/Middle Age/Aged/Whites/Asians/Blacks/human.

RefID:9272. Ward, J. A., Chester, J., Bates, S., and Richards, J.. Identifying risks and responding to overdose: piloting of an overdose prevention programme. Journal of Substance Use 2002. 7 (1) 6-14. **Keywords:** CINAHL/Overdose -- Prevention and Control/Substance Abusers -- Education/substance abuse/Health Education/Program Development/Evaluation Research/Pilot Studies/Semi-Structured Interview/Questionnaires/Substance Abusers/Street Drugs/Stress,Psychological/Attitude to Health/Clinical Assessment Tools/Information Needs/Teaching Methods/Outcomes of Education/content analysis/Data Analysis Software/P-Value/Age Factors/Sex Factors/Male/Female/Adult/Middle Age/England/human.

RefID:9277. Sowers, K. M., Ellis, R. A., Washington, T. A., and Currant, M.. Optimizing treatment effects for substance-abusing women with children: an evaluation of the Susan B. Anthony Center. Research on Social Work Practice 2002. 12 (1) 143-158. **Keywords:** CINAHL/Substance Abuse -- Therapy/Mothers/outcome assessment/Evaluation Research/convenience sample/Female/Adult/Middle Age/Research Instruments/Consumer Satisfaction/Substance Abuse -- Epidemiology/Retrospective Design/Blacks/Hispanics/Native Americans/Whites/Race

Factors/outcomes research/chi square test/Descriptive Statistics/human.

RefID:9281. Gonzalez, G. and Rosenheck, R. A.. Outcomes and service use among homeless persons with serious mental illness and substance abuse. Psychiatric Services 2002. 53 (4) 437-446. **Keywords:** CINAHL/Mental Health Services -- Utilization/Homeless Persons/Mental Disorders,Chronic/Health Services Accessibility/Diagnosis,Dual (Psychiatry)/Descriptive Statistics/Support Groups/Substance Use Disorders -- Epidemiology/Substance Use Disorders -- Therapy/Time Factors/Treatment Outcomes/United States/P-Value/Psychological Tests/T-Tests/Adult/Male/Female/Funding Source/human.

RefID:9293. Haack, M. R. and Yocom, C. J.. State policies and nurses with substance use disorders. Journal of Nursing Scholarship 2002. 34 (1) 89-94. **Keywords:** CINAHL/Professional Discipline/Impairment,Health Professional/Registered Nurses/substance abuse/Practical Nurses/Government Regulations -- United States/State Boards of Nursing/Funding Source/Prospective Studies/Comparative Studies/descriptive research/Descriptive Statistics/Questionnaires/Mail/United States/conceptual framework/Sampling Methods/chi square test/Analysis of Variance/Employment/Recurrence/theory/Adult/Middle Age/Male/Female/human.

RefID:9294. Gregoire, K. A. and Schultz, D. J.. Substance-abusing child welfare parents: treatment and child placement outcomes. Child Welfare 2001. 80 (4) 433-452. **Keywords:** CINAHL/substance abuse/Parents/Child Welfare/Funding Source/Pennsylvania/Referral and Consultation/Treatment Outcomes/Interview Guides/chi square test/Social Workers/Female/Male/Adult/Descriptive Statistics/Sex Factors/Substance Use Rehabilitation Programs/Child Custody/human.

RefID:9322. Buppert, C.. Legal limits. How a seemingly innocuous act can lead to loss of license. Journal for Nurse Practitioners 2008. 4 (4) 251-252. **Keywords:** CINAHL/Advanced Nursing Practice/Nurse Practitioners/Professional Discipline/Alcohol Drinking/Analgesics,Opioid/State Boards of Nursing/Substance Abuse -- Rehabilitation.

RefID:9328. . Nurse practice act: title 58, chapter 31; nurse practice act rules R156-31; health care providers immunity from liability act: title 58, chapter 13; division of occupational and professional licensing act: title 58, chapter 1; general rules of the division of occupational and professional licensing R156-1... Utah. #journal name# 1995. #volume# (#Issue#) various-#End Page#. **Keywords:** CINAHL/Nursing Practice -- Legislation and Jurisprudence -- Utah/State Boards of Nursing -- Administration -- Utah/Utah/Registered Nurses -- Legislation and Jurisprudence -- Utah/Practical Nursing -- Legislation and Jurisprudence -- Utah/Advanced Nursing Practice -- Legislation and Jurisprudence -- Utah/Licensure,Nursing -- Legislation and Jurisprudence -- Utah/State Board Examinations -- Legislation and Jurisprudence -- Utah/Professional Discipline -- Legislation and Jurisprudence -- Utah/Prescriptive Authority -- Legislation and Jurisprudence -- Utah/Professional Misconduct -- Legislation and Jurisprudence -- Utah/Delegation of Authority -- Legislation and Jurisprudence -- Utah/Schools,Nursing -- Legislation and Jurisprudence -- Utah/Scope of Nursing Practice -- Legislation and Jurisprudence

-- Utah/Liability,Legal -- Legislation and Jurisprudence -- Utah/Emergency Care -- Legislation and Jurisprudence -- Utah/Philanthropy -- Utah/Legal Procedure -- Legislation and Jurisprudence -- Utah.

RefID:9356. Bisson, J.. Clinical evidence concise. Post-traumatic stress disorder. American family physician 1-1-2006. 73 (1) 120-#End Page#. **Keywords:** CINAHL/Stress Disorders,Post-Traumatic -- Prevention and Control/Stress Disorders,Post-Traumatic -- Therapy/Cognitive Therapy/Desensitization,Psychologic/Education,Continuing (Credit)/Eye Movements/Medical Practice,Evidence-Based/Stress Disorders,Post-Traumatic -- Drug Therapy/stress management.

RefID:9360. Griffin, G.. Drug and alcohol liaison nursing service to a general medical ward: a six-month project. Journal of Substance Use 1999. 4 (2) 109-116. **Keywords:** CINAHL/Substance Abuse -- Nursing/Addictions Nursing/Nurse Liaison/Hospital Units/Substance Abuse -- Prevention and Control/Nursing Role/Evaluation Research/Pilot Studies/Program Evaluation/Nursing Assessment/Clinical Assessment Tools/Visual Analog Scaling/Questionnaires/Referral and Consultation/Staff Development/patient education/Teaching Materials/Male/Female/Adult/Middle Age/Aged/Aged,80 and over/Inpatients/Academic Medical Centers/Wales/human.

RefID:9361. Ross, M. W., Simon Rosser, B. R., Bauer, G. R., Bockting, W. O., Robinson, B. E., Rugg, D. L., and Coleman, E.. Drug use, unsafe sexual behavior, and internalized homonegativity in men who have sex with men. AIDS & Behavior 2001. 5 (1) 97-103. **Keywords:** CINAHL/Homosexuals,Male -- Psychosocial Factors/Risk Taking Behavior/substance abuse/sexuality/Research Subject Recruitment/Male/Adult/Questionnaires/Demography/hypothesis/Summated Rating Scaling/Scales/Alcohol Drinking/Clinical Assessment Tools/Psychological Tests/chi square test/Fisher's Exact Test/statistical significance/Confidence Intervals/Odds Ratio/Logistic Regression/Life Style/Homophobia/Funding Source/human.

RefID:9363. Semp, D. and Madgeskind, S.. Gay practices for harm reduction: inviting lesbians, gay men, and takataapui to be part of alcohol and drug harm reduction. Journal of Substance Use 2000. 5 (2) 92-98. **Keywords:** CINAHL/Homosexuals/substance abuse/Health Personnel/Substance Use Rehabilitation Programs -- New Zealand/Quality Assurance/conceptual framework/Social Environment/Counseling/Professional-Patient Relations/Patient Attitudes/Attitude of Health Personnel/Survey Research/Questionnaires/Thematic Analysis/Affirmative Action/New Zealand/human.

RefID:9372. Copello, A., Orford, J., Velleman, R., Templeton, L., and Krishnan, M.. Methods for reducing alcohol and drug related family harm in non-specialist settings. Journal of Mental Health 2000. 9 (3) 329-343. **Keywords:** CINAHL/Substance Abuse -- Psychosocial Factors/Family Coping/Family Therapy/Professional Practice,Evidence-Based/Alcohol Abuse -- Psychosocial Factors/Counseling/Support,Psychosocial/Funding Source/Adult/Middle Age/Male/Female.

RefID:9374. Commons, D. S.. Nonoccupational medical factors impacting workers'

compensation. Professional case management 2007. 12 (3) 187-188. **Keywords:** CINAHL/Job Re-Entry/Worker's Compensation/Cardiovascular Diseases/Depression/diabetes mellitus/Obesity/Smoking/Substance Dependence.

RefID:9406. Martin, K. R.. Adolescent treatment programs reduce drug abuse, produce other improvements. Nida Notes 2002. 17 (1) 11-12. **Keywords:** CINAHL/Drug Rehabilitation Programs -- Evaluation/Substance Abuse -- Rehabilitation -- In Adolescence/Treatment Outcomes/adolescence.

RefID:9454. Marcus, M. T.. An interdisciplinary team model for substance abuse prevention in communities. Journal of Professional Nursing 2000. 16 (3) 158-168. **Keywords:** CINAHL/Substance Abuse -- Prevention and Control -- Texas/Interinstitutional Relations/Community Mental Health Services -- Texas/Funding Source/Goals and Objectives/Collaboration/conceptual framework/Faculty Development/Models,Theoretical/Faculty,Nursing/Schools,Nursing -- Texas/Texas/Cultural Competence/teamwork.

RefID:9464. Aristeiguieta, C. A.. Culture and medicine. Screening patients for alcohol, tobacco, and other drug misuse: the role of brief interventions. WJM: Western Journal of Medicine 2000. 172 (1) 53-57. **Keywords:** CINAHL/Health Screening/substance abuse/Primary Health Care/Substance Abuse -- Therapy/Smoking/Alcohol Drinking.

RefID:9509. Holland, M.. Clinical topics in addiction. Mental Health Practice 2008. 12 (4) 20-20. **Keywords:** CINAHL/Professional Practice/Substance Dependence -- Therapy.

RefID:9576. Gibson, A. and Gibson, T.. Motivational interviewing. Practising Midwife 2000. 3 (1) 32-35. **Keywords:** CINAHL/Counseling -- Methods/Interviews -- Methods/Motivation -- In Pregnancy/Expectant Mothers -- Psychosocial Factors/Behavioral Changes -- In Pregnancy/Pregnancy/Female/Midwifery/Professional-Client Relations/Smoking -- In Pregnancy/Pregnancy,Unplanned -- Psychosocial Factors/Substance Abuse,Perinatal -- Psychosocial Factors.

RefID:9590. Trinkoff, A. M. and Storr, C. L.. Substance use among nurses: differences between specialties... reprinted with permission from Am J Public Health 1998;88:581-585. Journal of Addictions Nursing 1998. 10 (2) 77-84. **Keywords:** CINAHL/Specialties,Nursing/Coping/Nurses -- Psychosocial Factors/Funding Source/Mail/Prevalence/Surveys/Stratified Random Sample/Odds Ratio/Confidence Intervals/Questionnaires/chi square test/P-Value/Logistic Regression/exploratory research/human.

RefID:9622. Shaner, A., Roberts, L. J., Eckman, T. A., Tucker, D. E., Tsuang, J. W., Wilkins, J. N., and Mintz, J.. Monetary reinforcement of abstinence from cocaine among mentally ill patients with cocaine dependence... this paper was presented at the annual scientific meeting of the College on Problems of Drug Dependence held June 22-27, 1996, in San Juan, Puerto Rico. Psychiatric Services 1997. 48 (6) 807-810. **Keywords:** CINAHL/Behavior Modification --

Methods/schizophrenia/Substance Dependence/Diagnosis,Differential/Cannabis/Analysis of Variance/Repeated Measures/Pretest-Posttest Design/Summated Rating Scaling/Adult/Outpatients/Male/Funding Source/human.

RefID:9674. Duffin, C.. 'Let us help young sex workers get off drugs'. Nursing Standard 10-1-2007. 21 (18) 12-13. **Keywords:** CINAHL/Prescriptive Authority/Specialties,Nursing/Substance Abuse -- Nursing/Attitude of Health Personnel/Female/Male/Mobile Health Units/Sex Factors/United Kingdom.

RefID:9675. Hohman, M. M.. Motivational interviewing: an intervention tool for child welfare case workers working with substance-abusing parents. Child Welfare 1998. 77 (3) 275-289. **Keywords:** CINAHL/Interviews -- Methods/Professional-Client Relations/Parents/substance abuse/Motivation/Social Work/Psychological Theory.

RefID:9708. Howe, E. G.. At the bedside. Deadly sins, continued: treating patients with addictions. Journal of Clinical Ethics 1996. 7 (3) 195-204. **Keywords:** CINAHL/Behavior,Addictive/Substance Abusers -- Psychosocial Factors/Substance Dependence/Attitude of Health Personnel/Control (Psychology)/Refusal to Treat -- Psychosocial Factors/Professional-Patient Relations/Decision Making,Clinical/Perception/Empathy/ethics/Accountability.

RefID:9715. Winn, L. and de Ruiter, M.. Evaluating a pregnancy liaison service for drug-using women. Nursing Times 10-10-2006. 102 (41) 30-31. **Keywords:** CINAHL/Substance Abuse,Perinatal -- Therapy/adolescence/Adult/Data Analysis Software/Female/Pregnancy/Pregnancy Outcomes/Questionnaires/Record Review/human.

RefID:9723. . Identifying and treating the substance abuser... part one of two. JAAPA: Journal of the American Academy of Physician Assistants 1996. 9 (12) 20-#End Page#. **Keywords:** CINAHL/Substance Abuse Detection/Substance Abuse -- Epidemiology/Street Drugs/Risk Factors/Clinical Assessment Tools/Substance Abuse -- Complications/Substance Abuse -- Economics.

RefID:9729. Mathias, R.. NIDA's Clinical Trials Network marks progress toward improved drug abuse treatment. Nida Notes 2002. 16 (6) 11-12. **Keywords:** CINAHL/Clinical Trials/Collaboration/Drug Rehabilitation Programs/Substance Abuse -- Prevention and Control/National Institute on Drug Abuse (U.S.)/Research Personnel/Research Priorities/Research Protocols.

RefID:9797. Nation, J. M., Benshoff, J. J., and Malkin, M. M.. Therapeutic recreation programs for adolescents in substance abuse treatment facilities. Journal of Rehabilitation 1996. 62 (4) 10-16. **Keywords:** CINAHL/Recreational Therapy -- In Adolescence/Substance Abuse -- Therapy -- In Adolescence/Rehabilitation -- Methods -- In Adolescence/Questionnaires/Personnel Shortage/Goals and Objectives/adolescence/human.

RefID:9831. Hahn, E. J.. Predictors of parent involvement in drug prevention. #journal name# 1992. #volume# (#Issue#) #Start Page#-286. **Keywords:** CINAHL/Substance Abuse -- Prevention and Control/Parental Attitudes/Parenting/Correlational Studies/Discriminant Analysis/Interviews/Self Report/convenience sample/Health Belief Model/human.

RefID:9836. Oda, D. S. and O'Grady, R.. Public health nursing services for drug-exposed infants and mothers: a pilot study [corrected] [published erratum appears in J COMMUNITY HEALTH NURS 1994;11(4):251]. Journal of community health nursing 1994. 11 (3) 165-175. **Keywords:** CINAHL/Community Health Nursing/Maternal-Child Nursing/Substance Abuse,Perinatal -- Nursing/Child Health Services -- Utilization/Maternal Health Services -- Utilization/exploratory research/Pilot Studies/descriptive research/Cross Sectional Studies/Role Theory/Case Management/Content Validity/Infant,Newborn/Infant/adolescence/Adult/Female/human.

RefID:9893. Gropper, M.. The many faces of cocaine: the importance of psychosocial assessment in diagnosing and treating cocaine abuse. Social work in health care 1991. 16 (2) 97-112. **Keywords:** CINAHL/Diagnosis,Psychosocial/Substance Abuse -- Psychosocial Factors/Substance Abuse -- Diagnosis/Cocaine -- Adverse Effects/Pregnancy,High Risk/DSM/Social Work/Denial (Psychology)/Substance Abuse -- Therapy/Referral and Consultation.

RefID:9935. Dodge, V. H.. Relaxation training: a nursing intervention for substance abusers. Archives of psychiatric nursing 1991. 5 (2) 99-104. **Keywords:** CINAHL/Relaxation Techniques -- Utilization/substance abuse/Substance Abuse -- Therapy/Research Instruments/Psychological Tests/Psychophysiology/Substance Abuse -- Etiology/Psychiatric Nursing/Clinical Nurse Specialists.

RefID:9942. Faugier, J.. Substance abuse: a misuse of ability. Nursing Times 23-1-1991. 87 (4) 26-29. **Keywords:** CINAHL/substance abuse/United Kingdom/HIV Infections/Specialties,Nursing.

RefID:9967. Robinson, T. M. S.. Education in the NICU. Perinatal substance abuse: working with neonates and families. Neonatal Network 1999. 18 (2) 68-70. **Keywords:** CINAHL/Substance Abuse,Perinatal -- Complications -- In Infancy and Childhood/Infant,Drug-Exposed/Neonatal Abstinence Syndrome/Neonatal Nursing/Infant,Newborn/fetus/Fetal Alcohol Syndrome/Pregnancy/Female/Pregnancy Outcomes/Cocaine -- Adverse Effects -- In Infancy and Childhood/Mothers -- Psychosocial Factors/Family -- Psychosocial Factors/Professional-Family Relations.

RefID:10061. . Brief counseling can reduce or stop drug use. AORN Journal 2005. 81 (5) 1026-1026. **Keywords:** CINAHL/Counseling/Substance Abuse -- Prevention and Control.

RefID:10118. Reid, D. L.. Healthy Start PLUS. Kansas Nurse 1996. 71 (7) 17-17. **Keywords:** CINAHL/Child Welfare -- Kansas/Child Health Services -- Kansas/Kansas.

RefID:10120. Albaugh, J. A. and Kellogg-Spadt, S.. Intimacy issues. Man's search for ultimate

sex: VIAGRA abuse. Urologic Nursing 2003. 23 (1) 75-76. **Keywords:** CINAHL/Sildenafil -- Adverse Effects/substance abuse/Substance Abuse -- Prevention and Control/Male/Street Drugs.

RefID:10151. Robbins, C. E.. A monitored treatment program for impaired health care professionals. Journal of Nursing Administration 1987. 17 (2) 17-21. **Keywords:** CINAHL/Impairment,Health Professional/Substance Dependence -- Rehabilitation/Occupational Health Services/Contracts/Nursing Staff,Hospital/Nurses.

RefID:10192. Dillmann, J. M.. Substance abuse in the perioperative setting. AORN Journal 1995. 62 (1) 111-112. **Keywords:** CINAHL/Perioperative Nursing/substance abuse/Impairment,Health Professional.

RefID:10198. Turner, T.. Think again. Forcing people to give up drugs is not the answer. Nursing Times 16-9-2003. 99 (37) 19-19. **Keywords:** CINAHL/Substance Abuse -- Prevention and Control/Professional-Client Relations/Substance Abusers.

RefID:10213. Clark, M. D.. Drug use and nursing students: a program for prevention... part 2. Nurse Educator 1988. 13 (6) 22-24. **Keywords:** Impairment,Health Professional/Students,Nursing/substance abuse/Substance Abuse -- Education/Substance Dependence/Teaching Methods/Ethics,Nursing/Risk Factors/Self Medication/Student Attitudes.

RefID:10244. Richenbaugh, F.. The chemically dependent nurse and intervention. South Carolina Nurse 1996. 3 (4) 14-14. **Keywords:** Substance Dependence/Impairment,Health Professional/Nurses.

RefID:10245. Hoffmeister, R.. The chemically dependent nurse: identification, intervention & reintegration into practice. Kansas Nurse 1989. 64 (5) 5-6. **Keywords:** Impairment,Health Professional/Nurses/substance abuse.

RefID:10246. Reed, M. T.. The dependent nurse... drugs or alcohol. Nursing Times 19-1-1983. 79 (3) 12-13. **Keywords:** Impairment,Health Professional/Alcoholism/Substance Dependence.

RefID:10249. Abbott, C. A.. The impaired nurse: management strategies... part 2. AORN Journal 1987. 46 (6) 1104-#End Page#. **Keywords:** Hospital Policies/Impairment,Health Professional/Job Interviews/Nurses/Occupational Health Services/Personnel Management/Substance Dependence -- Psychosocial Factors/Substance Dependence -- Rehabilitation/Employee Discipline.

RefID:10609. Barnes, Lisa B.. An Evaluation of the Right Choices Program to Determine Effectiveness in Delivering Constructive Interventions and Providing an Early Support Program in Order to Modify Behavior of First-Time Student Offenders Who Commit Drug and Violent Acts. ProQuest LLC 2010. #volume# (#Issue#) #Start Page#-#End Page#. **Keywords:** Control Groups/Urban Schools/Behavior Problems/Student Behavior/Program Evaluation/Focus

Groups/Intervention/Violence/Interviews/drug abuse/Law Enforcement/Surveys/Suspension/Low Achievement/School Districts/Dropouts/Teacher Student Relationship/Classroom Techniques/Parent Participation/Program Effectiveness/Student Attitudes/Crime/ERIC RCTS.

RefID:10615. Gonzales, Arturo, Westerberg, Verner S., Peterson, Thomas R., Moseley, Ana, Gryczynski, Jan, Mitchell, Shannon Gwin, Buff, Gary, and Schwartz, Robert P.. Implementing a Statewide Screening, Brief Intervention, and Referral to Treatment (SBIRT) Service in Rural Health Settings: New Mexico SBIRT. Substance Abuse 2012. 33 (2) 114-123. **Keywords:** Medical Services/substance abuse/Intervention/drug use/Mental Health Programs/Referral/alcohol abuse/Screening Tests/Adults/National Programs/Eligibility/ERIC RCTS.

RefID:10617. Babor, Thomas F., McRee, Bonnie G., Kassebaum, Patricia A., Grimaldi, Paul L., Ahmed, Kazi, and Bray, Jeremy. Screening, Brief Intervention, and Referral to Treatment (SBIRT): Toward a Public Health Approach to the Management of Substance Abuse. Substance Abuse 2007. 28 (3) 7-30. **Keywords:** Delivery Systems/substance abuse/early intervention/Screening Tests/Demonstration Programs/Primary Health Care/Cost Effectiveness/Referral/At Risk Persons/Test Construction/Intervention/alcohol abuse/Elementary Schools/School Health Services/Hospitals/drug abuse/Smoking/Health Promotion/Public Health/Counseling Techniques/drug therapy/Adolescents/ERIC RCTS.

RefID:10632. Hettema, Jennifer E., Sorensen, James L., Uy, Manelisa, and Jain, Sharad. Motivational Enhancement Therapy to Increase Resident Physician Engagement in Substance Abuse Education. Substance Abuse 2009. 30 (3) 244-247. **Keywords:** substance abuse/Intervention/Physicians/drug use/Internal Medicine/statistical significance/Educational Opportunities/therapy/Referral/Screening Tests/alcohol abuse/Motivation Techniques/Measures (Individuals)/Attitude Change/Attitude Measures/behavior change/ERIC RCTS.

RefID:10713. . rosebud sioux tribal education department & tribal education code: external evaluation. final report. #journal name# 1999. #volume# (#Issue#) #Start Page#-#End Page#. **Keywords:** American Indian Education/Dropout Rate/Educational Administration/Elementary Secondary Education/School Law/Sioux (Tribe)/Tribal Government/Tribally Controlled Education/ERIC RCTS.

RefID:10715. Nakonezny, Paul A. and Ojeda, Michael. Health Services Utilization between Older and Younger Homeless Adults.(author Abstract). The Gerontologist 1-4-2005. 45 (2) 249-#End Page#. **Keywords:** Regression (Statistics)/Primary Health Care/Patients/Medical Services/substance abuse/Quasiexperimental Design/Multivariate Analysis/Mental Disorders/Hospitals/Homeless People/ERIC RCTS.

RefID:10725. Staines, Graham L., McKendrick, Karen, Perlis, Theresa, Sacks, Stanley, and De Leon, George. Sequential Assignment and Treatment-as-Usual: Alternatives to Standard Experimental Designs in Field Studies of Treatment Efficacy. Evaluation review 1999. 23 (1) 47-

76. **Keywords:** Field Studies/Outcomes of Treatment/Program Effectiveness/Program Evaluation/Research Methodology/Statistical Bias/ERIC RCTS.

RefID:10754. Cowart, Virginia S.. Would Controlled Substance Status Affect Steroid Trafficking?. Physician and Sportsmedicine 1987. 15 (5) 151-52,154. **Keywords:** Athletes/drug abuse/Federal Government/Government Role/prevention/ERIC RCTS.

RefID:10757. Lohr, Mary Jane and And, Others. Substance Use during Pregnancy in Adolescence. #journal name# 1990. #volume# (#Issue#) #Start Page#-#End Page#. **Keywords:** Adolescents/behavior change/drug use/Early Parenthood/Pregnancy/substance abuse/ERIC RCTS.

RefID:10762. Wilson, Geraldine S. and And, Others. The Development of Preschool Children of Heroin-Addicted Mothers: A Controlled Study. Pediatrics 1979. 63 (1) 135-141. **Keywords:** child development/Drug Addiction/Early Childhood Education/Exceptional Child Research/Mothers/Parent Influence/Perinatal Influences/Prenatal Influences/ERIC RCTS.

RefID:10776. World Health Organization. Validation of the Alcohol, Smoking and Substance Involvement Screening Test (ASSIST) and Pilot Brief Intervention: A Technical Report of Phase II Findings of the WHO ASSIST Project. #journal name# 2006. #volume# (#Issue#) #Start Page#-#End Page#. **Keywords:** #Keywords#.

RefID:10777. Washington State Department of Social and Health Services. Washington State Screening, Brief Intervention, and Referral to Treatment Program: Final Program Performance Report. #journal name# 2010. #volume# (#Issue#) #Start Page#-#End Page#. **Keywords:** #Keywords#.

RefID:10778. Estee, Sharon, He, Lijian, and Yang, Summer. Substance Use Outcomes: All WASBIRT Hospitals: Six-Month Follow-Up Survey of WASBIRT Patients. #journal name# 2009. #volume# (#Issue#) #Start Page#-#End Page#. **Keywords:** #Keywords#.

RefID:10779. Office of National Drug Control Policy. Screening and Brief Intervention. #journal name# 2007. #volume# (#Issue#) #Start Page#-#End Page#. **Keywords:** #Keywords#.

RefID:10797. Smith, E. A., Palen, L. A., Caldwell, L. L., Flisher, A. J., Graham, J. W., Mathews, C., Wegner, L., and Vergnani, T.. Substance use and sexual risk prevention in Cape Town, South Africa: an evaluation of the HealthWise program. Prev.Sci 2008. 9 (4) 311-321. **Keywords:** Adolescent/Adolescent Behavior/Condoms/utilization/Female/Health Promotion/Humans/Male/School Health Services/Social Marketing/South Africa/Substance-Related Disorders/prevention & control/Unsafe Sex/from SR bibliographies.

RefID:10799. Wiggins, M., Bonell, C., Sawtell, M., Austerberry, H., Burchett, H., Allen, E., and Strange, V.. Health outcomes of youth development programme in England: prospective matched comparison study. BMJ (Clinical research ed.) 2009. 339 (#Issue#) b2534-#End Page#.

Keywords: Adolescent/Adolescent Health Services/organization & administration/England/Female/Humans/Male/Pregnancy/Pregnancy in Adolescence/prevention & control/Prognosis/Program Evaluation/Prospective Studies/Socioeconomic Factors/Substance-Related Disorders/Unsafe Sex/Vulnerable Populations/from SR bibliographies.

RefID:10801. Fors, S. W. and Jarvis, S.. Evaluation of a peer-led drug abuse risk reduction project for runaway/homeless youths. J Drug Educ. 1995. 25 (4) 321-333. **Keywords:** Adolescent/Adult/Analysis of Variance/Child/Health Knowledge,Attitudes,Practice/Homeless Youth/Humans/Least-Squares Analysis/Peer Group/Program Evaluation/Runaway Behavior/Self-Help Groups/organization & administration/Substance-Related Disorders/prevention & control.

Level 2: Population recruited/voluntary

RefID:22. Barnett, Elizabeth, Spruijt-Metz, Donna, Unger, Jennifer B., Sun, Ping, Rohrbach, Louise Ann, and Sussman, Steve. Boosting a teen substance use prevention program with motivational interviewing. Substance use & misuse 2012. 47 (4) 418-428. **Keywords:** #Keywords#.

RefID:51. de Dios, Marcel A., Herman, Debra S., Britton, Willoughby B., Hagerty, Claire E., Anderson, Bradley J., and Stein, Michael D.. Motivational and mindfulness intervention for young adult female marijuana users. Journal of substance abuse treatment 2012. 42 (1) 56-64. **Keywords:** Adolescent/Adult/Feasibility Studies/Female/Follow-Up Studies/Humans/*Interview,Psychological/mt [Methods]/*Marijuana Abuse/rh [Rehabilitation]/Marijuana Smoking/ep [Epidemiology]/*Marijuana Smoking/pc [Prevention & Control]/*Meditation/mt [Methods]/Motivation/Pilot Projects/Regression Analysis/Treatment Outcome/Young Adult.

RefID:70. Stanger, Catherine, Ryan, Stacy R., Fu, Hongyun, and Budney, Alan J.. Parent training plus contingency management for substance abusing families: a Complier Average Causal Effects (CACE) analysis. Drug and alcohol dependence 2011. 118 (2-3) 119-126. **Keywords:** Adult/Child/*Child Behavior/px [Psychology]/Child Behavior Disorders/px [Psychology]/*Child of Impaired Parents/px [Psychology]/Child,Preschool/Female/Humans/Male/Parent-Child Relations/*Parenting/px [Psychology]/*Parents/ed [Education]/*Substance-Related Disorders/px [Psychology].

RefID:74. Walker, Denise D., Stephens, Robert, Roffman, Roger, Demarce, Josephine, Lozano, Brian, Towe, Sheri, and Berg, Belinda. Randomized controlled trial of motivational enhancement therapy with nontreatment-seeking adolescent cannabis users: a further test of the teen marijuana check-up. Psychology of addictive behaviors : journal of the Society of Psychologists in Addictive Behaviors 2011. 25 (3) 474-484. **Keywords:** Adolescent/*Behavior Therapy/mt [Methods]/Female/Humans/Interview,Psychological/Male/Marijuana Abuse/px [Psychology]/*Marijuana Abuse/th [Therapy]/Marijuana Smoking/px [Psychology]/*Marijuana

Smoking/th [Therapy]/*Motivation/*Psychotherapy,Brief/mt [Methods]/Treatment Outcome.

RefID:78. Mason, Michael, Pate, Patricia, Drapkin, Michelle, and Sozinho, Kayilu. Motivational interviewing integrated with social network counseling for female adolescents: a randomized pilot study in urban primary care. Journal of substance abuse treatment 2011. 41 (2) 148-155. **Keywords:** Adolescent/Evidence-Based Practice/Female/Humans/Interview,Psychological/Motivation/Pilot Projects/*Primary Health Care/Psychiatric Status Rating Scales/*Psychotherapy,Brief/mt [Methods]/Questionnaires/Risk-Taking/*Sexual Behavior/px [Psychology]/*Social Support/*Substance-Related Disorders/pc [Prevention & Control]/Substance-Related Disorders/px [Psychology]/Treatment Outcome/Urban Population.

RefID:134. McCambridge, Jim, Day, Maria, Thomas, Bonnita A., and Strang, John. Fidelity to Motivational Interviewing and subsequent cannabis cessation among adolescents. Addictive behaviors 2011. 36 (7) 749-754. **Keywords:** Adolescent/Female/Humans/*Interview,Psychological/London/Male/*Marijuana Abuse/px [Psychology]/Marijuana Abuse/rh [Rehabilitation]/*Motivation/Outcome and Process Assessment (Health Care)/Treatment Outcome/Young Adult.

RefID:162. Stormshak, Elizabeth A., Connell, Arin M., Veronneau, Marie Helene, Myers, Michael W., Dishion, Thomas J., Kavanagh, Kathryn, and Caruthers, Allison S.. An ecological approach to promoting early adolescent mental health and social adaptation: family-centered intervention in public middle schools. Child development 2011. 82 (1) 209-225. **Keywords:** Adolescent/Alcoholism/eh [Ethnology]/*Alcoholism/pc [Prevention & Control]/Alcoholism/px [Psychology]/Antisocial Personality Disorder/eh [Ethnology]/*Antisocial Personality Disorder/pc [Prevention & Control]/Antisocial Personality Disorder/px [Psychology]/Ethnic Groups/px [Psychology]/*Family Therapy/mt [Methods]/Female/*Health Promotion/mt [Methods]/Humans/Male/Motivation/Oregon/*School Health Services/Sex Factors/*Social Adjustment/*Social Environment/Substance-Related Disorders/eh [Ethnology]/*Substance-Related Disorders/pc [Prevention & Control]/Substance-Related Disorders/px [Psychology]/Urban Population.

RefID:173. Werch, Chudley E., Bian, Hui, Carlson, Joan M., Moore, Michele J., Diclemente, Carlo C., Huang, I. Chan, Ames, Steven C., Thombs, Dennis, Weiler, Robert M., and Pokorny, Steven B.. Brief integrative multiple behavior intervention effects and mediators for adolescents. Journal of behavioral medicine 2011. 34 (1) 3-12. **Keywords:** Adolescent/Alcohol Drinking/pc [Prevention & Control]/*Behavior Therapy/Female/Florida/Food Habits/Fruit/*Health Behavior/*Health Promotion/mt [Methods]/Humans/Logistic Models/Male/*Psychotherapy,Brief/Relaxation/Risk Factors/Vegetables.

RefID:176. Martino, Steve, Ball, Samuel A., Nich, Charla, Canning-Ball, Monica, Rounsaville, Bruce J., and Carroll, Kathleen M.. Teaching community program clinicians motivational interviewing using expert and train-the-trainer strategies. Addiction (Abingdon, England) 2011. 106 (2) 428-441. **Keywords:** Adult/*Clinical

Competence/Connecticut/*Education,Medical,Continuing/mt [Methods]/Educational Measurement/mt [Methods]/Female/Humans/*Interview,Psychological/Male/Middle Aged/Motivation/Program Development/Program Evaluation/Regression Analysis/Substance Abuse Treatment Centers/*Substance-Related Disorders/th [Therapy]/*Teaching/mt [Methods]/Teaching Materials/Technology Transfer.

RefID:179. Ondersma, Steven J., Grekin, Emily R., and Svikis, Dace. The potential for technology in brief interventions for substance use, and during-session prediction of computer-delivered brief intervention response. Substance use & misuse 2011. 46 (1) 77-86. **Keywords:** Adult/African Americans/Computers/Female/Hair/ch [Chemistry]/*Health Promotion/mt [Methods]/Humans/*Internet/Postpartum Period/Poverty/Pregnancy/ROC Curve/Randomized Controlled Trials as Topic/Risk Factors/*Substance-Related Disorders/pc [Prevention & Control]/Substance-Related Disorders/px [Psychology]/*Substance-Related Disorders/th [Therapy]/Substance-Related Disorders/ur [Urine]/User-Computer Interface/Young Adult.

RefID:200. Grossbard, Joel R., Mastroleo, Nadine R., Kilmer, Jason R., Lee, Christine M., Turrisi, Rob, Larimer, Mary E., and Ray, Anne. Substance use patterns among first-year college students: secondary effects of a combined alcohol intervention. Journal of substance abuse treatment 2010. 39 (4) 384-390. **Keywords:** Alcohol Drinking/ep [Epidemiology]/*Alcohol Drinking/pc [Prevention & Control]/Analysis of Variance/Female/Follow-Up Studies/Humans/Longitudinal Studies/Male/Marijuana Smoking/ep [Epidemiology]/Marijuana Smoking/pc [Prevention & Control]/Motivation/Parents/*Psychotherapy,Brief/mt [Methods]/Smoking/ep [Epidemiology]/Smoking/pc [Prevention & Control]/*Students/px [Psychology]/Substance-Related Disorders/ep [Epidemiology]/*Substance-Related Disorders/pc [Prevention & Control]/Treatment Outcome/United States/Universities.

RefID:202. Cornelius, Jack R., Bukstein, Oscar G., Douaihy, Antoine B., Clark, Duncan B., Chung, Tammy A., Daley, Dennis C., Wood, D. Scott, and Brown, Sandra J.. Double-blind fluoxetine trial in comorbid MDD-CUD youth and young adults. Drug and alcohol dependence 2010. 112 (1-2) 39-45. **Keywords:** Adolescent/Adult/Antidepressive Agents,Second-Generation/ae [Adverse Effects]/*Antidepressive Agents,Second-Generation/tu [Therapeutic Use]/*Cognitive Therapy/Combined Modality Therapy/Comorbidity/*Depressive Disorder,Major/dt [Drug Therapy]/Depressive Disorder,Major/th [Therapy]/Double-Blind Method/Female/Fluoxetine/ae [Adverse Effects]/*Fluoxetine/tu [Therapeutic Use]/Humans/Male/*Marijuana Abuse/dt [Drug Therapy]/Placebos/Treatment Outcome/Young Adult.

RefID:204. Fang, Lin, Schinke, Steven P., and Cole, Kristin C. A.. Preventing substance use among early Asian-American adolescent girls: initial evaluation of a web-based, mother-daughter program. The Journal of adolescent health : official publication of the Society for Adolescent Medicine 2010. 47 (5) 529-532. **Keywords:** Adolescent/Adult/*Asian Americans/Child/Data Collection/Female/Humans/*Internet/Middle Aged/*Nuclear Family/*Substance-Related Disorders/pc [Prevention & Control]/United States/User-Computer Interface.

RefID:205. Freudenberg, Nicholas, Ramaswamy, Megha, Daniels, Jessie, Crum, Martha, Ompad,

Danielle C., and Vlahov, David. Reducing drug use, human immunodeficiency virus risk, and recidivism among young men leaving jail: evaluation of the REAL MEN re-entry program. The Journal of adolescent health : official publication of the Society for Adolescent Medicine 2010. 47 (5) 448-455. **Keywords:** Adolescent/Community Networks/*HIV Infections/pc [Prevention & Control]/Humans/Male/New York City/*Prisoners/Program Evaluation/Sexual Behavior/*Substance-Related Disorders/pc [Prevention & Control]/Young Adult.

RefID:217. Brooks, Adam C., Ryder, Deanna, Carise, Deni, and Kirby, Kimberly C.. Feasibility and effectiveness of computer-based therapy in community treatment. Journal of substance abuse treatment 2010. 39 (3) 227-235. **Keywords:** Adaptation,Psychological/Adult/Cocaine-Related Disorders/rh [Rehabilitation]/Evidence-Based Medicine/mt [Methods]/Feasibility Studies/Female/*Health Knowledge,Attitudes,Practice/Humans/Internet/Male/Middle Aged/Pilot Projects/Referral and Consultation/sn [Statistics & Numerical Data]/Reinforcement (Psychology)/*Substance-Related Disorders/rh [Rehabilitation]/*Therapy,Computer-Assisted/mt [Methods].

RefID:218. Reback, Cathy J., Peck, James A., Dierst-Davies, Rhodri, Nuno, Miriam, Kamien, Jonathan B., and Amass, Leslie. Contingency management among homeless, out-of-treatment men who have sex with men. Journal of substance abuse treatment 2010. 39 (3) 255-263. **Keywords:** Adult/Alcohol Drinking/ep [Epidemiology]/Alcohol Drinking/pc [Prevention & Control]/Central Nervous System Stimulants/ad [Administration & Dosage]/Central Nervous System Stimulants/ae [Adverse Effects]/Feasibility Studies/Follow-Up Studies/HIV Infections/ep [Epidemiology]/*HIV Infections/pc [Prevention & Control]/*Health Promotion/mt [Methods]/*Homeless Persons/Homosexuality,Male/Humans/Male/Mental Disorders/ep [Epidemiology]/Methamphetamine/ad [Administration & Dosage]/Methamphetamine/ae [Adverse Effects]/Middle Aged/Risk-Taking/Substance-Related Disorders/ep [Epidemiology]/*Substance-Related Disorders/rh [Rehabilitation].

RefID:221. Rosenberg, Stanley D., Goldberg, Richard W., Dixon, Lisa B., Wolford, George L., Slade, Eric P., Himelhoch, Seth, Gallucci, Gerard, Potts, Wendy, Tapscott, Stephanie, and Welsh, Christopher J.. Assessing the STIRR model of best practices for blood-borne infections of clients with severe mental illness. Psychiatric services (Washington, D.C.) 2010. 61 (9) 885-891. **Keywords:** Adult/*Blood-Borne Pathogens/ip [Isolation & Purification]/Evidence-Based Medicine/Female/HIV Seropositivity/di [Diagnosis]/Hepatitis C/di [Diagnosis]/Humans/Male/Mass Screening/*Mental Disorders/Middle Aged/Models,Theoretical/Patient Acceptance of Health Care/Referral and Consultation/*Severity of Illness Index/Urban Population.

RefID:223. Bisaga, Adam, Aharonovich, Efrat, Cheng, Wendy Y., Levin, Frances R., Mariani, John J., Raby, Wilfrid N., and Nunes, Edward V.. A placebo-controlled trial of memantine for cocaine dependence with high-value voucher incentives during a pre-randomization lead-in period. Drug and alcohol dependence 2010. 111 (1-2) 97-104. **Keywords:** Adult/*Behavior Therapy/Clinical Trials as Topic/*Cocaine-Related Disorders/th [Therapy]/Excitatory Amino Acid Antagonists/tu [Therapeutic Use]/Female/Humans/Kaplan-Meier Estimate/Logistic

Models/Male/*Memantine/tu [Therapeutic Use]/Middle Aged/Sex Factors/Single-Blind Method/Substance Abuse Detection/Treatment Outcome.

RefID:228. Coviello, Donna M., Cornish, James W., Lynch, Kevin G., Alterman, Arthur I., and O'Brien, Charles P.. A randomized trial of oral naltrexone for treating opioid-dependent offenders. The American journal on addictions / American Academy of Psychiatrists in Alcoholism and Addictions 2010. 19 (5) 422-432. **Keywords:** Administration,Oral/Adolescent/Adult/*Criminals/px [Psychology]/Female/Humans/Male/Middle Aged/Naltrexone/ad [Administration & Dosage]/*Naltrexone/tu [Therapeutic Use]/*Narcotic Antagonists/ad [Administration & Dosage]/*Opioid-Related Disorders/dt [Drug Therapy]/*Opioid-Related Disorders/th [Therapy]/*Psychotherapy/mt [Methods].

RefID:230. Jamison, Robert N., Ross, Edgar L., Michna, Edward, Chen, Li Q., Holcomb, Caroline, and Wasan, Ajay D.. Substance misuse treatment for high-risk chronic pain patients on opioid therapy: a randomized trial. Pain 2010. 150 (3) 390-400. **Keywords:** Adult/*Analgesics,Opioid/ae [Adverse Effects]/*Back Pain/dt [Drug Therapy]/Chronic Disease/Diagnosis,Dual (Psychiatry)/Disability Evaluation/Discriminant Analysis/Drug Users/sn [Statistics & Numerical Data]/Electronic Health Records/sn [Statistics & Numerical Data]/Female/Humans/Male/Middle Aged/Opioid-Related Disorders/di [Diagnosis]/*Opioid-Related Disorders/px [Psychology]/Opioid-Related Disorders/ur [Urine]/Pain Measurement/Patient Compliance/sn [Statistics & Numerical Data]/Questionnaires/Risk Factors/Young Adult.

RefID:248. Lee, Christine M., Neighbors, Clayton, Kilmer, Jason R., and Larimer, Mary E.. A brief, web-based personalized feedback selective intervention for college student marijuana use: a randomized clinical trial. Psychology of addictive behaviors : journal of the Society of Psychologists in Addictive Behaviors 2010. 24 (2) 265-273. **Keywords:** Adolescent/*Feedback,Psychological/Health Surveys/Humans/Intention to Treat Analysis/*Internet/*Marijuana Abuse/th [Therapy]/Marijuana Smoking/Patient Selection/Questionnaires/Sex Factors/*Students/Treatment Outcome/Universities/Young Adult.

RefID:259. Wechsberg, Wendee M., Novak, Scott P., Zule, William A., Browne, Felicia A., Kral, Alex H., Ellerson, Rachel Middlesteadt, and Kline, Tracy. Sustainability of intervention effects of an evidence-based HIV prevention intervention for African American women who smoke crack cocaine. Drug and alcohol dependence 2010. 109 (1-3) 205-212. **Keywords:** Adolescent/Adult/African Americans/Alcohol Drinking/ep [Epidemiology]/Anxiety/co [Complications]/Anxiety/px [Psychology]/*Cocaine-Related Disorders/co [Complications]/*Crack Cocaine/Depressive Disorder/co [Complications]/Depressive Disorder/px [Psychology]/Employment/Evidence-Based Medicine/Female/Follow-Up Studies/*HIV Infections/co [Complications]/*HIV Infections/pc [Prevention & Control]/Homeless Persons/Humans/Middle Aged/North Carolina/ep [Epidemiology]/Patient Dropouts/Predictive Value of Tests/Risk Factors/Socioeconomic Factors/Treatment

Outcome/Unsafe Sex/Young Adult.

RefID:265. Fernandes, Simone, Ferigolo, Maristela, Benchaya, Mariana Canellas, Moreira, Tais de Campos, Pierozan, Pollianna Sangalli, Mazoni, Claudia Galvao, and Barros, Helena Maria Tannhauser. Brief Motivational Intervention and telemedicine: a new perspective of treatment to marijuana users. Addictive behaviors 2010. 35 (8) 750-755. **Keywords:** Adolescent/Adult/Brazil/Child/Female/Humans/Male/*Marijuana Abuse/th [Therapy]/*Motivation/*Psychotherapy,Brief/mt [Methods]/Questionnaires/Regression Analysis/Socioeconomic Factors/*Telemedicine/mt [Methods]/Time Factors/Young Adult.

RefID:266. Rigter, Henk, Pelc, Isidore, Tossmann, Peter, Phan, Olivier, Grichting, Esther, Hendriks, Vincent, and Rowe, Cindy. INCANT: a transnational randomized trial of multidimensional family therapy versus treatment as usual for adolescents with cannabis use disorder. BMC psychiatry 2010. 10 (#Issue#) 28-#End Page#. **Keywords:** Adolescent/Adult/Checklist/Cognitive Therapy/mt [Methods]/Community Mental Health Services/mt [Methods]/Cross-Cultural Comparison/Europe/ep [Epidemiology]/*Family Therapy/mt [Methods]/Female/Humans/*International Cooperation/Male/Marijuana Abuse/ep [Epidemiology]/*Marijuana Abuse/th [Therapy]/Pilot Projects/Psychiatric Status Rating Scales/sn [Statistics & Numerical Data]/Questionnaires/Research Design/Treatment Outcome.

RefID:275. Milby, Jesse B., Schumacher, Joseph E., Wallace, Dennis, Vuchinich, Rudy, Mennemeyer, Stephen T., and Kertesz, Stefan G.. Effects of sustained abstinence among treated substance-abusing homeless persons on housing and employment. American journal of public health 2010. 100 (5) 913-918. **Keywords:** Adult/Alabama/*Cocaine-Related Disorders/th [Therapy]/*Employment/Female/*Homeless Persons/*Housing/Humans/Male/Middle Aged/*Patient Compliance/Time Factors/Treatment Outcome.

RefID:283. Werch, Chudley E Chad, Bian, Hui, Diclemente, Carlo C., Moore, Michelle J., Thombs, Dennis, Ames, Steven C., Huang, I. Chan, and Pokorny, Steven. A brief image-based prevention intervention for adolescents. Psychology of addictive behaviors : journal of the Society of Psychologists in Addictive Behaviors 2010. 24 (1) 170-175. **Keywords:** Adolescent/Female/Humans/Male/*Psychotherapy,Brief/Referral and Consultation/*Self Concept/*Smoking/pc [Prevention & Control]/*Smoking Cessation/mt [Methods]/*Substance-Related Disorders/pc [Prevention & Control].

RefID:287. Tetzlaff, John, Collins, Gregory B., Brown, David L., Leak, Byron C., Pollock, Greg, and Popa, Daniela. A strategy to prevent substance abuse in an academic anesthesiology department. Journal of clinical anesthesia 2010. 22 (2) 143-150. **Keywords:** *Academic Medical Centers/Anesthesiology/ed [Education]/*Anesthesiology/st [Standards]/Contracts/Humans/Mass Screening/mt [Methods]/*Program Development/mt [Methods]/*Substance-Related Disorders/pc [Prevention & Control]/Third-Party Consent.

RefID:314. Hien, Denise A., Jiang, Huiping, Campbell, Aimee N. C., Hu, Mei Chen, Miele, Gloria

M., Cohen, Lisa R., Brigham, Gregory S., Capstick, Carrie, Kulaga, Agatha, Robinson, James, Suarez-Morales, Lourdes, and Nunes, Edward V.. Do treatment improvements in PTSD severity affect substance use outcomes? A secondary analysis from a randomized clinical trial in NIDA's Clinical Trials Network. The American journal of psychiatry 2010. 167 (1) 95-101. **Keywords:** Adult/Alcoholism/di [Diagnosis]/Alcoholism/ep [Epidemiology]/Alcoholism/th [Therapy]/*Cognitive Therapy/mt [Methods]/Comorbidity/Diagnosis,Dual (Psychiatry)/sn [Statistics & Numerical Data]/Female/Follow-Up Studies/Humans/Life Change Events/Longitudinal Studies/Markov Chains/*National Institute on Drug Abuse (U.S.)/*Patient Education as Topic/mt [Methods]/Psychotherapy,Group/mt [Methods]/Severity of Illness Index/Stress Disorders,Post-Traumatic/di [Diagnosis]/Stress Disorders,Post-Traumatic/ep [Epidemiology]/*Stress Disorders,Post-Traumatic/th [Therapy]/Substance-Related Disorders/di [Diagnosis]/Substance-Related Disorders/ep [Epidemiology]/*Substance-Related Disorders/th [Therapy]/Treatment Outcome/United States/ep [Epidemiology].

RefID:323. Jaffee, William B., Bailey, Genie L., Lohman, Michelle, Riggs, Paula, McDonald, Leah, and Weiss, Roger D.. Methods of recruiting adolescents with psychiatric and substance use disorders for a clinical trial. The American journal of drug and alcohol abuse 2009. 35 (5) 381-384. **Keywords:** Adolescent/*Attention Deficit Disorder with Hyperactivity/di [Diagnosis]/Attention Deficit Disorder with Hyperactivity/th [Therapy]/Clinical Trials as Topic/Cognitive Therapy/Community Mental Health Centers/Diagnosis,Dual (Psychiatry)/Humans/Methylphenidate/tu [Therapeutic Use]/Multicenter Studies as Topic/*Patient Selection/Randomized Controlled Trials as Topic/Referral and Consultation/Social Work/Substance Abuse Treatment Centers/*Substance-Related Disorders/di [Diagnosis].

RefID:325. Henderson, Craig E., Rowe, Cindy L., Dakof, Gayle A., Hawes, Sam W., and Liddle, Howard A.. Parenting practices as mediators of treatment effects in an early-intervention trial of multidimensional family therapy. The American journal of drug and alcohol abuse 2009. 35 (4) 220-226. **Keywords:** Adolescent/*Adolescent Behavior/px [Psychology]/Child/Ethnic Groups/*Family Therapy/mt [Methods]/Female/Florida/ep [Epidemiology]/Humans/Interviews as Topic/Male/*Parent-Child Relations/Parenting/px [Psychology]/*Parenting/Peer Group/Poverty/Psychometrics/Substance-Related Disorders/ep [Epidemiology]/*Substance-Related Disorders/th [Therapy]/Treatment Outcome.

RefID:338. Naar-King, Sylvie, Parsons, Jeffrey T., Murphy, Debra A., Chen, Xinguang, Harris, D. Robert, and Belzer, Marvin E.. Improving health outcomes for youth living with the human immunodeficiency virus: a multisite randomized trial of a motivational intervention targeting multiple risk behaviors. Archives of pediatrics & adolescent medicine 2009. 163 (12) 1092-1098. **Keywords:** Adolescent/Adolescent Behavior/Anti-HIV Agents/ad [Administration & Dosage]/Female/*HIV Seropositivity/*Health Behavior/*Health Education/Humans/Intervention Studies/Interviews as Topic/Male/Motivation/Patient Compliance/Regression Analysis/Risk-Taking/Sexual Behavior/Substance-Related Disorders/ep [Epidemiology]/United States/Viral Load/Young Adult.

RefID:352. Schmitz, Joy M., Lindsay, Jan A., Green, Charles E., Herin, David V., Stotts, Angela L., and Moeller, F. Gerard. High-dose naltrexone therapy for cocaine-alcohol dependence. The American journal on addictions / American Academy of Psychiatrists in Alcoholism and Addictions 2009. 18 (5) 356-362. **Keywords:** Adult/Alcoholism/co [Complications]/*Alcoholism/dt [Drug Therapy]/Alcoholism/th [Therapy]/Cocaine-Related Disorders/co [Complications]/*Cocaine-Related Disorders/dt [Drug Therapy]/Cocaine-Related Disorders/th [Therapy]/Cognitive Therapy/mt [Methods]/Combined Modality Therapy/Dose-Response Relationship,Drug/Female/Humans/Male/*Naltrexone/ad [Administration & Dosage]/Naltrexone/ae [Adverse Effects]/*Narcotic Antagonists/ad [Administration & Dosage]/Narcotic Antagonists/ae [Adverse Effects]/*Patient Compliance/sn [Statistics & Numerical Data].

RefID:354. Otto, Christiane, Crackau, Brit, Lohrmann, Ira, Zahradnik, Anne, Bischof, Gallus, John, Ulrich, and Rumpf, Hans Jurgen. Brief intervention in general hospital for problematic prescription drug use: 12-month outcome. Drug and alcohol dependence 2009. 105 (3) 221-226. **Keywords:** Adolescent/Adult/Aged/Female/Hospitals,General/Humans/Male/Middle Aged/*Prescription Drugs/ae [Adverse Effects]/*Psychotherapy,Brief/mt [Methods]/*Substance-Related Disorders/th [Therapy]/Time Factors/Treatment Outcome.

RefID:355. Stanger, Catherine, Budney, Alan J., Kamon, Jody L., and Thostensen, Jeff. A randomized trial of contingency management for adolescent marijuana abuse and dependence. Drug and alcohol dependence 2009. 105 (3) 240-247. **Keywords:** Adolescent/*Adolescent Behavior/px [Psychology]/Adult/*Behavior,Addictive/th [Therapy]/Child/*Cognitive Therapy/mt [Methods]/*Family Therapy/mt [Methods]/Female/Humans/Male/*Marijuana Abuse/th [Therapy]/Patient Compliance/Treatment Outcome.

RefID:373. Nagel, Tricia, Robinson, Gary, Condon, John, and Trauer, Tom. Approach to treatment of mental illness and substance dependence in remote Indigenous communities: results of a mixed methods study. The Australian journal of rural health 2009. 17 (4) 174-182. **Keywords:** Adult/Chronic Disease/Cultural Competency/Diagnosis,Dual (Psychiatry)/Female/*Health Services,Indigenous/og [Organization & Administration]/Humans/Male/Mental Disorders/eh [Ethnology]/Mental Disorders/et [Etiology]/*Mental Disorders/th [Therapy]/Northern Territory/Oceanic Ancestry Group/*Psychotherapy,Brief/mt [Methods]/Substance-Related Disorders/eh [Ethnology]/*Substance-Related Disorders/th [Therapy].

RefID:375. Barrowclough, Christine, Haddock, Gillian, Beardmore, Ruth, Conrod, Patricia, Craig, Tom, Davies, Linda, Dunn, Graham, Lewis, Shon, Moring, Jan, Tarrier, Nick, and Wykes, Til. Evaluating integrated MI and CBT for people with psychosis and substance misuse: recruitment, retention and sample characteristics of the MIDAS trial. Addictive behaviors 2009. 34 (10) 859-866. **Keywords:** Adolescent/Adult/Aged/Cognitive Therapy/mt [Methods]/Female/Follow-Up Studies/Great Britain/Humans/Male/Mental Health Services/Middle Aged/*Motivation/*Patient Selection/*Psychotic Disorders/th [Therapy]/*Randomized Controlled Trials as Topic/Single-Blind Method/*Substance-Related Disorders/th

[Therapy]/Young Adult.

RefID:387. Alemagno, Sonia A., Stephens, Richard C., Stephens, Peggy, Shaffer-King, Peggy, and White, Patrick. Brief motivational intervention to reduce HIV risk and to increase HIV testing among offenders under community supervision. Journal of correctional health care : the official journal of the National Commission on Correctional Health Care 2009. 15 (3) 210-221. **Keywords:** Community-Institutional Relations/Computer-Assisted Instruction/*Counseling/Criminal Law/mt [Methods]/Female/*HIV Infections/di [Diagnosis]/*HIV Infections/pc [Prevention & Control]/HIV Infections/px [Psychology]/Harm Reduction/*Health Education/mt [Methods]/Health Knowledge,Attitudes,Practice/Humans/Male/*Motivation/Pilot Projects/*Prisoners/*Sexual Behavior/Substance Abuse Treatment Centers/mt [Methods]/Substance Abuse,Intravenous/px [Psychology]/Substance Abuse,Intravenous/vi [Virology].

RefID:390. Wechsberg, Wendee M., Wu, Li Tzy, Zule, William A., Parry, Charles D., Browne, Felicia A., Luseno, Winnie K., Kline, Tracy, and Gentry, Amanda. Substance abuse, treatment needs and access among female sex workers and non-sex workers in Pretoria, South Africa. Substance abuse treatment, prevention, and policy 2009. 4 (#Issue#) 11-#End Page#. **Keywords:** Adolescent/Adult/Age of Onset/Cross-Sectional Studies/Female/*Health Services Accessibility/Humans/Middle Aged/*Needs Assessment/Prevalence/*Prostitution/px [Psychology]/Randomized Controlled Trials as Topic/South Africa/ep [Epidemiology]/*Substance-Related Disorders/ep [Epidemiology]/*Substance-Related Disorders/px [Psychology]/*Unsafe Sex/px [Psychology].

RefID:396. Crits-Christoph, Paul, Gibbons, Mary Beth Connolly, Ring-Kurtz, Sarah, Gallop, Robert, and Present, Julie. A pilot study of community-friendly manual-guided drug counseling. Journal of substance abuse treatment 2009. 37 (1) 8-16. **Keywords:** Adult/*Cocaine-Related Disorders/rh [Rehabilitation]/Community Mental Health Services/mt [Methods]/*Directive Counseling/mt [Methods]/Female/Follow-Up Studies/Guideline Adherence/Humans/Male/Manuals as Topic/Middle Aged/Pilot Projects/Professional Competence/*Psychotherapy,Group/mt [Methods]/Treatment Outcome.

RefID:408. Kidorf, Michael, King, Van L., Neufeld, Karin, Peirce, Jessica, Kolodner, Ken, and Brooner, Robert K.. Improving substance abuse treatment enrollment in community syringe exchangers. Addiction (Abingdon, England) 2009. 104 (5) 786-795. **Keywords:** Adult/Baltimore/ep [Epidemiology]/Drug Users/Female/*Harm Reduction/Humans/Male/Motivation/*Needle-Exchange Programs/og [Organization & Administration]/*Opioid-Related Disorders/th [Therapy]/*Reward/*Substance Abuse Treatment Centers/og [Organization & Administration]/Substance Abuse,Intravenous/ep [Epidemiology]/*Substance Abuse,Intravenous/th [Therapy].

RefID:452. Kay-Lambkin, Frances J., Baker, Amanda L., Lewin, Terry J., and Carr, Vaughan J.. Computer-based psychological treatment for comorbid depression and problematic alcohol and/or cannabis use: a randomized controlled trial of clinical efficacy. Addiction (Abingdon,

England) 2009. 104 (3) 378-388. **Keywords:** Adolescent/Adult/Alcohol-Related Disorders/px [Psychology]/*Alcohol-Related Disorders/th [Therapy]/*Cognitive Therapy/mt [Methods]/Depressive Disorder/px [Psychology]/*Depressive Disorder/th [Therapy]/Diagnosis,Dual (Psychiatry)/Female/Harm Reduction/Humans/Male/Marijuana Abuse/px [Psychology]/*Marijuana Abuse/th [Therapy]/Middle Aged/Motivation/New South Wales/Psychotherapy,Brief/mt [Methods]/Telemedicine/mt [Methods]/*Therapy,Computer-Assisted/mt [Methods]/Treatment Outcome/Young Adult.

RefID:461. Liddle, Howard A., Rowe, Cynthia L., Dakof, Gayle A., Henderson, Craig E., and Greenbaum, Paul E.. Multidimensional family therapy for young adolescent substance abuse: twelve-month outcomes of a randomized controlled trial. Journal of consulting and clinical psychology 2009. 77 (1) 12-25. **Keywords:** Adolescent/*Family Therapy/mt [Methods]/Female/Health Services Needs and Demand/Humans/Juvenile Delinquency/sn [Statistics & Numerical Data]/Male/Parent-Child Relations/Parenting/Questionnaires/Substance-Related Disorders/ep [Epidemiology]/*Substance-Related Disorders/th [Therapy]/Time Factors/Treatment Outcome.

RefID:484. Marlowe, Douglas B., Festinger, David S., Dugosh, Karen L., Arabia, Patricia L., and Kirby, Kimberly C.. An effectiveness trial of contingency management in a felony preadjudication drug court. Journal of applied behavior analysis 2008. 41 (4) 565-577. **Keywords:** Adolescent/Adult/Alcoholism/px [Psychology]/*Alcoholism/rh [Rehabilitation]/Case Management/*Crime/lj [Legislation & Jurisprudence]/Drug and Narcotic Control/lj [Legislation & Jurisprudence]/Female/Humans/Male/Motivation/*Prisoners/lj [Legislation & Jurisprudence]/Prisoners/px [Psychology]/Punishment/Reinforcement Schedule/*Street Drugs/Substance-Related Disorders/px [Psychology]/*Substance-Related Disorders/rh [Rehabilitation]/*Token Economy/Young Adult.

RefID:485. Preston, Kenzie L., Ghitza, Udi E., Schmittner, John P., Schroeder, Jennifer R., and Epstein, David H.. Randomized trial comparing two treatment strategies using prize-based reinforcement of abstinence in cocaine and opiate users. Journal of applied behavior analysis 2008. 41 (4) 551-563. **Keywords:** Adult/Cocaine-Related Disorders/px [Psychology]/*Cocaine-Related Disorders/rh [Rehabilitation]/Combined Modality Therapy/Community Mental Health Services/Female/Heroin Dependence/px [Psychology]/*Heroin Dependence/rh [Rehabilitation]/Humans/Male/Middle Aged/*Motivation/Reinforcement Schedule/Substance Abuse Detection/Substance Withdrawal Syndrome/di [Diagnosis]/Substance Withdrawal Syndrome/rh [Rehabilitation]/*Token Economy/Treatment Outcome.

RefID:501. McCambridge, Jim, Slym, Renee L., and Strang, John. Randomized controlled trial of motivational interviewing compared with drug information and advice for early intervention among young cannabis users. Addiction (Abingdon, England) 2008. 103 (11) 1809-1818. **Keywords:** Adolescent/Alcohol Drinking/px [Psychology]/*Alcohol Drinking/th [Therapy]/Counseling/mt [Methods]/Female/Follow-Up Studies/Humans/Interview,Psychological/mt [Methods]/Male/Marijuana Smoking/px [Psychology]/*Marijuana Smoking/th [Therapy]/*Motivation/*Patient Education as

Topic/*Psychotherapy,Brief/*Smoking Cessation/px [Psychology]/Treatment Outcome/Young Adult.

RefID:502. Werch, Chudley E., Bian, Hui, Moore, Michele J., Ames, Steven C., Diclemente, Carlo C., Thombs, Dennis, and Pokorny, Steven B.. Brief multiple behavior health interventions for older adolescents. American journal of health promotion : AJHP 2008. 23 (2) 92-96. **Keywords:** Adolescent/Age Factors/Alcohol Drinking/Analysis of Variance/Cannabis/Female/*Health Behavior/Health Promotion/Health Surveys/Humans/Male/Models,Psychological/Nutritional Status/*Program Development/Psychometrics/Smoking.

RefID:507. Werch, Chudley E., Moore, Michele J., Bian, Hui, Diclemente, Carlo C., Ames, Steven C., Weiler, Robert M., Thombs, Dennis, Pokorny, Steven B., and Huang, I. Chan. Efficacy of a brief image-based multiple-behavior intervention for college students. Annals of behavioral medicine : a publication of the Society of Behavioral Medicine 2008. 36 (2) 149-157. **Keywords:** Adolescent/Adult/Alcohol Drinking/pc [Prevention & Control]/Analysis of Variance/*Behavior Therapy/mt [Methods]/Combined Modality Therapy/Female/Follow-Up Studies/*Health Promotion/mt [Methods]/Humans/Imagery (Psychotherapy)/Male/Patient Education as Topic/Physical Fitness/px [Psychology]/*Risk Reduction Behavior/*Risk-Taking/*Students/px [Psychology]/Substance-Related Disorders/pc [Prevention & Control]/Treatment Outcome/Universities.

RefID:517. Liddle, Howard A., Dakof, Gayle A., Turner, Ralph M., Henderson, Craig E., and Greenbaum, Paul E.. Treating adolescent drug abuse: a randomized trial comparing multidimensional family therapy and cognitive behavior therapy. Addiction (Abingdon, England) 2008. 103 (10) 1660-1670. **Keywords:** Adolescent/Adolescent Behavior/px [Psychology]/Ambulatory Care/mt [Methods]/*Cognitive Therapy/mt [Methods]/*Family Therapy/mt [Methods]/Female/Humans/Male/Patient Compliance/Substance-Related Disorders/ep [Epidemiology]/Substance-Related Disorders/px [Psychology]/*Substance-Related Disorders/th [Therapy]/United States/ep [Epidemiology].

RefID:521. Shoptaw, Steven, Reback, Cathy J., Larkins, Sherry, Wang, Pin Chieh, Rotheram-Fuller, Erin, Dang, Jeff, and Yang, Xiaowei. Outcomes using two tailored behavioral treatments for substance abuse in urban gay and bisexual men. Journal of substance abuse treatment 2008. 35 (3) 285-293. **Keywords:** Adult/Amphetamine-Related Disorders/px [Psychology]/Amphetamine-Related Disorders/rh [Rehabilitation]/*Bisexuality/*Cognitive Therapy/mt [Methods]/Follow-Up Studies/*Homosexuality,Male/Humans/Male/Methamphetamine/ae [Adverse Effects]/Middle Aged/Risk Reduction Behavior/*Risk-Taking/*Sexual Behavior/px [Psychology]/Social Support/Substance-Related Disorders/px [Psychology]/*Substance-Related Disorders/rh [Rehabilitation]/Time Factors/Treatment Outcome/Urban Population.

RefID:568. Bickel, Warren K., Marsch, Lisa A., Buchhalter, August R., and Badger, Gary J.. Computerized behavior therapy for opioid-dependent outpatients: a randomized controlled trial. Experimental and clinical psychopharmacology 2008. 16 (2) 132-143. **Keywords:**

Adult/Alcohols/bl [Blood]/Alcohols/ur [Urine]/*Behavior Therapy/mt [Methods]/Buprenorphine/tu [Therapeutic Use]/*Computer-Aided Design/Double-Blind Method/Female/Humans/Male/Narcotic Antagonists/tu [Therapeutic Use]/Opioid-Related Disorders/px [Psychology]/*Opioid-Related Disorders/th [Therapy]/*Reinforcement (Psychology)/Severity of Illness Index/Treatment Outcome.

RefID:575. Martin, Greg and Copeland, Jan. The adolescent cannabis check-up: randomized trial of a brief intervention for young cannabis users. Journal of substance abuse treatment 2008. 34 (4) 407-414. **Keywords:** Adolescent/Adult/Feedback/Female/Humans/Male/*Marijuana Abuse/th [Therapy]/*Psychotherapy,Brief/mt [Methods]/Questionnaires/Treatment Outcome.

RefID:579. Poduska, Jeanne M., Kellam, Sheppard G., Wang, Wei, Brown, C. Hendricks, Ialongo, Nicholas S., and Toyinbo, Peter. Impact of the Good Behavior Game, a universal classroom-based behavior intervention, on young adult service use for problems with emotions, behavior, or drugs or alcohol. Drug and alcohol dependence 2008. 95 Suppl 1 (#Issue#) S29-S44. **Keywords:** Achievement/Adolescent/Adult/Affective Symptoms/ep [Epidemiology]/*Affective Symptoms/pc [Prevention & Control]/*Aggression/px [Psychology]/Alcoholism/ep [Epidemiology]/*Alcoholism/pc [Prevention & Control]/Attention Deficit and Disruptive Behavior Disorders/ep [Epidemiology]/*Attention Deficit and Disruptive Behavior Disorders/th [Therapy]/Baltimore/*Behavior Therapy/mt [Methods]/Child/Cohort Studies/Conduct Disorder/ep [Epidemiology]/*Conduct Disorder/pc [Prevention & Control]/Female/Follow-Up Studies/Humans/Male/*Mental Health Services/ut [Utilization]/*Schools/Sex Factors/*Socialization/Substance-Related Disorders/ep [Epidemiology]/*Substance-Related Disorders/pc [Prevention & Control]/*Urban Population/Utilization Review/sn [Statistics & Numerical Data].

RefID:584. Wechsberg, Wendee M., Luseno, Winnie K., Karg, Rhonda S., Young, Siobhan, Rodman, Nathaniel, Myers, Bronwyn, and Parry, Charles D. H.. Alcohol, cannabis, and methamphetamine use and other risk behaviours among Black and Coloured South African women: a small randomized trial in the Western Cape. The International journal on drug policy 2008. 19 (2) 130-139. **Keywords:** Adolescent/Adult/African Continental Ancestry Group/sn [Statistics & Numerical Data]/Alcohol Drinking/ep [Epidemiology]/Alcohol Drinking/eh [Ethnology]/Condoms/ut [Utilization]/Evidence-Based Medicine/mt [Methods]/Female/Focus Groups/*HIV Infections/pc [Prevention & Control]/HIV Infections/tm [Transmission]/Humans/Marijuana Abuse/ep [Epidemiology]/Marijuana Abuse/eh [Ethnology]/Methamphetamine/ae [Adverse Effects]/Middle Aged/Pilot Projects/*Risk-Taking/*Sexual Behavior/eh [Ethnology]/Sexual Partners/South Africa/eh [Ethnology]/*Substance-Related Disorders/ep [Epidemiology]/Substance-Related Disorders/eh [Ethnology]/Unsafe Sex/eh [Ethnology]/*Unsafe Sex/sn [Statistics & Numerical Data].

RefID:585. Litt, Mark D., Kadden, Ronald M., Kabela-Cormier, Elise, and Petry, Nancy M.. Coping skills training and contingency management treatments for marijuana dependence: exploring mechanisms of behavior change. Addiction (Abingdon, England) 2008. 103 (4) 638-648. **Keywords:** *Adaptation,Psychological/ph [Physiology]/Adult/*Cognitive Therapy/mt

[Methods]/Cognitive Therapy/st [Standards]/Female/Humans/Male/Marijuana Abuse/px [Psychology]/*Marijuana Abuse/rh [Rehabilitation]/Motivation/Self Efficacy/Treatment Outcome.

RefID:589. French, Michael T., Zavala, Silvana K., McCollister, Kathryn E., Waldron, Holly B., Turner, Charles W., and Ozechowski, Timothy J.. Cost-effectiveness analysis of four interventions for adolescents with a substance use disorder. Journal of substance abuse treatment 2008. 34 (3) 272-281. **Keywords:** Adolescent/Adult/Cognitive Therapy/ec [Economics]/Cost-Benefit Analysis/*Family Therapy/ec [Economics]/Female/*Health Care Costs/Humans/Male/*Substance-Related Disorders/ec [Economics]/*Substance-Related Disorders/rh [Rehabilitation]/Treatment Outcome.

RefID:597. Rapp, Richard C., Otto, Amy L., Lane, D. Timothy, Redko, Cristina, McGatha, Sue, and Carlson, Robert G.. Improving linkage with substance abuse treatment using brief case management and motivational interviewing. Drug and alcohol dependence 2008. 94 (1-3) 172-182. **Keywords:** Adolescent/Adult/*Case Management/Female/*Genetic Linkage/Humans/*Interviews as Topic/Male/Middle Aged/*Motivation/*Substance-Related Disorders/ep [Epidemiology].

RefID:608. Ghitza, Udi E., Epstein, David H., and Preston, Kenzie L.. Contingency management reduces injection-related HIV risk behaviors in heroin and cocaine using outpatients. Addictive behaviors 2008. 33 (4) 593-604. **Keywords:** Adolescent/Adult/Aged/*Cocaine-Related Disorders/rh [Rehabilitation]/Counseling/Female/*HIV Infections/pc [Prevention & Control]/*Heroin Dependence/rh [Rehabilitation]/Humans/Male/*Methadone/tu [Therapeutic Use]/Middle Aged/*Narcotics/tu [Therapeutic Use]/*Reward/Risk Factors/Substance Abuse Treatment Centers/Substance Abuse,Intravenous/rh [Rehabilitation].

RefID:611. Smock, Sara A., Trepper, Terry S., Wetchler, Joseph L., McCollum, Eric E., Ray, Rose, and Pierce, Kent. Solution-focused group therapy for level 1 substance abusers. Journal of marital and family therapy 2008. 34 (1) 107-120. **Keywords:** Aged/*Cognitive Therapy/mt [Methods]/*Family Therapy/mt [Methods]/Female/Humans/Interpersonal Relations/Male/Middle Aged/Reproducibility of Results/Research Design/*Self Efficacy/*Substance-Related Disorders/th [Therapy]/Treatment Outcome.

RefID:622. Scott, Christy K., Dennis, Michael L., and Foss, Mark A.. Utilizing Recovery Management Checkups to shorten the cycle of relapse, treatment reentry, and recovery. Drug and alcohol dependence 2005. 78 (3) 325-338. **Keywords:** Cognition Disorders/di [Diagnosis]/Cognition Disorders/ep [Epidemiology]/Environment/Humans/Motivation/*Periodicity/*Recovery of Function/Recurrence/Substance-Related Disorders/ep [Epidemiology]/*Substance-Related Disorders/th [Therapy]/Time Factors/Treatment Outcome.

RefID:624. Hogue, Aaron, Dauber, Sarah, Samuolis, Jessica, and Liddle, Howard A.. Treatment techniques and outcomes in multidimensional family therapy for adolescent behavior

problems. Journal of family psychology : JFP : journal of the Division of Family Psychology of the American Psychological Association (Division 43) 2006. 20 (4) 535-543. **Keywords:** Adolescent/Alcoholism/px [Psychology]/*Alcoholism/rh [Rehabilitation]/Child Behavior Disorders/px [Psychology]/*Child Behavior Disorders/rh [Rehabilitation]/Conflict (Psychology)/Family Relations/*Family Therapy/mt [Methods]/Female/Follow-Up Studies/Humans/Internal-External Control/Male/Substance-Related Disorders/px [Psychology]/*Substance-Related Disorders/rh [Rehabilitation].

RefID:635. Petry, Nancy M., Alessi, Sheila M., Hanson, Tressa, and Sierra, Sean. Randomized trial of contingent prizes versus vouchers in cocaine-using methadone patients. Journal of consulting and clinical psychology 2007. 75 (6) 983-991. **Keywords:** Adolescent/Cocaine-Related Disorders/ep [Epidemiology]/*Cocaine-Related Disorders/rh [Rehabilitation]/Female/Humans/Incidence/Male/*Methadone/tu [Therapeutic Use]/*Narcotics/tu [Therapeutic Use]/Reinforcement (Psychology)/*Reward/Time Factors/*Token Economy.

RefID:648. Kemp, Rosalind, Harris, Anthony, Vurel, Erdal, and Sitharthan, Thiagarajan. Stop Using Stuff: trial of a drug and alcohol intervention for young people with comorbid mental illness and drug and alcohol problems. Australasian psychiatry : bulletin of Royal Australian and New Zealand College of Psychiatrists 2007. 15 (6) 490-493. **Keywords:** Adult/*Alcohol Drinking/ep [Epidemiology]/Alcohol Drinking/pc [Prevention & Control]/*Alcohol Drinking/th [Therapy]/Cognitive Therapy/mt [Methods]/Comorbidity/Diagnosis,Dual (Psychiatry)/Diagnostic and Statistical Manual of Mental Disorders/Female/Humans/Male/Motivation/*Opioid-Related Disorders/ep [Epidemiology]/Opioid-Related Disorders/pc [Prevention & Control]/*Opioid-Related Disorders/th [Therapy]/Psychotherapy,Group/mt [Methods]/Psychotic Disorders/di [Diagnosis]/*Psychotic Disorders/ep [Epidemiology]/*Psychotic Disorders/px [Psychology]/Questionnaires/Severity of Illness Index/Time Factors.

RefID:651. Werch, Chudley E Chad, Bian, Hui, Moore, Michele J., Ames, Steve, Diclemente, Carlo C., and Weiler, Robert M.. Brief multiple behavior interventions in a college student health care clinic. The Journal of adolescent health : official publication of the Society for Adolescent Medicine 2007. 41 (6) 577-585. **Keywords:** Adolescent/Adult/Analysis of Variance/Body Image/Contracts/Counseling/Diet/Exercise/Female/*Health Behavior/*Health Promotion/mt [Methods]/Humans/Male/*Physical Fitness/Program Evaluation/Psychotherapy,Brief/Referral and Consultation/Risk Reduction Behavior/*Risk-Taking/Sleep/Southeastern United States/*Student Health Services/Substance-Related Disorders/pc [Prevention & Control]/Treatment Outcome/Universities.

RefID:671. Purcell, David W., Garfein, Richard S., Latka, Mary H., Thiede, Hanne, Hudson, Sharon, Bonner, Sebastian, Golub, Elizabeth T., Ouellet, Lawrence J., and DUIT Study Team. Development, description, and acceptability of a small-group, behavioral intervention to prevent HIV and hepatitis C virus infections among young adult injection drug users. Drug and alcohol dependence 2007. 91 Suppl 1 (#Issue#) S73-S80. **Keywords:**

Adolescent/Adult/*Cognitive Therapy/Data Interpretation,Statistical/Female/Follow-Up Studies/*HIV Infections/pc [Prevention & Control]/HIV Infections/px [Psychology]/*Health Education/*Hepatitis C/pc [Prevention & Control]/Hepatitis C/px [Psychology]/Humans/Male/Patient Acceptance of Health Care/Quality Assurance,Health Care/Research Design/Sexual Behavior/*Substance Abuse,Intravenous/co [Complications]/Substance Abuse,Intravenous/px [Psychology]/Unsafe Sex/px [Psychology].

RefID:672. Bailey, Susan L., Ouellet, Lawrence J., Mackesy-Amiti, Mary Ellen, Golub, Elizabeth T., Hagan, Holly, Hudson, Sharon M., Latka, Mary H., Gao, Weihua, Garfein, Richard S., and DUIT Study Team. Perceived risk, peer influences, and injection partner type predict receptive syringe sharing among young adult injection drug users in five U.S. cities. Drug and alcohol dependence 2007. 91 Suppl 1 (#Issue#) S18-S29. **Keywords:** Adolescent/Adult/Cross-Sectional Studies/Data Interpretation,Statistical/Female/Humans/Longitudinal Studies/Male/*Needle Sharing/px [Psychology]/Needle Sharing/sn [Statistics & Numerical Data]/*Peer Group/Proportional Hazards Models/Questionnaires/Recurrence/*Risk-Taking/Socioeconomic Factors/Substance Abuse,Intravenous/ep [Epidemiology]/*Substance Abuse,Intravenous/px [Psychology]/United States/ep [Epidemiology].

RefID:683. McKee, Sherry A., Carroll, Kathleen M., Sinha, Rajita, Robinson, Jane E., Nich, Charla, Cavallo, Dana, and O'Malley, Stephanie. Enhancing brief cognitive-behavioral therapy with motivational enhancement techniques in cocaine users. Drug and alcohol dependence 2007. 91 (1) 97-101. **Keywords:** Adult/*Cocaine-Related Disorders/th [Therapy]/*Cognitive Therapy/mt [Methods]/Demography/Female/Humans/Male/*Motivation/Psychotherapy,Brief/mt [Methods]/*Psychotherapy,Brief/Treatment Outcome.

RefID:686. Ford, Julian D., Hawke, Josephine, Alessi, Sheila, Ledgerwood, David, and Petry, Nancy. Psychological trauma and PTSD symptoms as predictors of substance dependence treatment outcomes. Behaviour research and therapy 2007. 45 (10) 2417-2431. **Keywords:** Adult/Female/Humans/Male/Multivariate Analysis/Prognosis/Psychotherapy/*Stress Disorders,Post-Traumatic/co [Complications]/Stress Disorders,Post-Traumatic/px [Psychology]/Stress Disorders,Post-Traumatic/th [Therapy]/*Substance Abuse,Intravenous/px [Psychology]/*Substance Abuse,Intravenous/th [Therapy]/Treatment Outcome.

RefID:687. Lester, Kristin M., Milby, Jesse B., Schumacher, Joseph E., Vuchinich, Rudolph, Person, Sharina, and Clay, Olivio J.. Impact of behavioral contingency management intervention on coping behaviors and PTSD symptom reduction in cocaine-addicted homeless. Journal of traumatic stress 2007. 20 (4) 565-575. **Keywords:** *Adaptation,Psychological/Adult/*Behavior Therapy/mt [Methods]/Cocaine-Related Disorders/di [Diagnosis]/Cocaine-Related Disorders/px [Psychology]/*Cocaine-Related Disorders/rh [Rehabilitation]/*Cognitive Therapy/mt [Methods]/Combined Modality Therapy/Comorbidity/Day Care/Diagnosis,Dual (Psychiatry)/Female/Follow-Up Studies/Goals/*Homeless Persons/px [Psychology]/Humans/Male/Mental Disorders/di [Diagnosis]/Mental Disorders/px [Psychology]/Mental Disorders/rh [Rehabilitation]/Middle Aged/Rehabilitation,Vocational/Stress Disorders,Post-Traumatic/di [Diagnosis]/Stress

Disorders,Post-Traumatic/px [Psychology]/*Stress Disorders,Post-Traumatic/rh [Rehabilitation]/*Token Economy.

RefID:694. Jungerman, Flavia S., Andreoni, Solange, and Laranjeira, Ronaldo. Short term impact of same intensity but different duration interventions for cannabis users. Drug and alcohol dependence 2007. 90 (2-3) 120-127. **Keywords:** Adolescent/Adult/Brazil/ep [Epidemiology]/Female/Follow-Up Studies/Humans/Male/*Marijuana Abuse/ep [Epidemiology]/*Marijuana Abuse/rh [Rehabilitation]/Marijuana Abuse/ur [Urine]/Middle Aged/*Periodicity/Professional Competence/Psychotherapy,Brief/mt [Methods]/Time Factors/Treatment Outcome.

RefID:697. Olmstead, Todd A., Sindelar, Jody L., Easton, Caroline J., and Carroll, Kathleen M.. The cost-effectiveness of four treatments for marijuana dependence. Addiction (Abingdon, England) 2007. 102 (9) 1443-1453. **Keywords:** Adolescent/Adult/Cognitive Therapy/ec [Economics]/*Cognitive Therapy/mt [Methods]/Connecticut/ep [Epidemiology]/Cost-Benefit Analysis/Counseling/ec [Economics]/*Counseling/mt [Methods]/Female/Humans/Male/Marijuana Abuse/ec [Economics]/*Marijuana Abuse/rh [Rehabilitation]/*Motivation/Treatment Outcome.

RefID:705. Ball, Samuel A., Martino, Steve, Nich, Charla, Frankforter, Tami L., Van Horn, Deborah, Crits-Christoph, Paul, Woody, George E., Obert, Jeanne L., Farentinos, Christiane, Carroll, Kathleen M., and National Institute on Drug Abuse Clinical Trials Network. Site matters: multisite randomized trial of motivational enhancement therapy in community drug abuse clinics. Journal of consulting and clinical psychology 2007. 75 (4) 556-567. **Keywords:** Adult/*Ambulatory Care Facilities/*Community Mental Health Services/ut [Utilization]/Female/Follow-Up Studies/Humans/Male/*Motivation/Retention (Psychology)/*Substance-Related Disorders/rh [Rehabilitation].

RefID:709. White, Helene R., Mun, Eun Young, Pugh, Lisa, and Morgan, Thomas J.. Long-term effects of brief substance use interventions for mandated college students: sleeper effects of an in-person personal feedback intervention. Alcoholism, clinical and experimental research 2007. 31 (8) 1380-1391. **Keywords:** Adolescent/Adult/Body Mass Index/Central Nervous System Depressants/bl [Blood]/Ethanol/bl [Blood]/Feedback/Female/Follow-Up Studies/Humans/Interview,Psychological/Male/Marijuana Smoking/px [Psychology]/Motivation/*Psychotherapy,Brief/Questionnaires/Smoking/px [Psychology]/Students/*Substance-Related Disorders/rh [Rehabilitation].

RefID:713. Moeller, F. Gerard, Schmitz, Joy M., Steinberg, Joel L., Green, Charles M., Reist, Christopher, Lai, Lingo Y., Swann, Alan C., and Grabowski, John. Citalopram combined with behavioral therapy reduces cocaine use: a double-blind, placebo-controlled trial. The American journal of drug and alcohol abuse 2007. 33 (3) 367-378. **Keywords:** Adult/*Behavior Therapy/mt [Methods]/Citalopram/ae [Adverse Effects]/*Citalopram/tu [Therapeutic Use]/Cocaine-Related Disorders/px [Psychology]/*Cocaine-Related Disorders/rh [Rehabilitation]/Double-Blind Method/Female/Humans/Male/Middle Aged/Patient

Compliance/px [Psychology]/Serotonin Uptake Inhibitors/ae [Adverse Effects]/*Serotonin Uptake Inhibitors/tu [Therapeutic Use]/Substance Abuse Detection/px [Psychology]/*Token Economy.

RefID:722. Winters, Ken C. and Leitten, Willia. Brief intervention for drug-abusing adolescents in a school setting. Psychology of addictive behaviors : journal of the Society of Psychologists in Addictive Behaviors 2007. 21 (2) 249-254. **Keywords:** Adolescent/Female/*Health Promotion/mt [Methods]/Humans/Male/Parents/Program Development/*Program Evaluation/*School Health Services/*Students/px [Psychology]/Substance-Related Disorders/ep [Epidemiology]/*Substance-Related Disorders/th [Therapy]/United States/ep [Epidemiology].

RefID:723. Jaffe, Adi, Shoptaw, Steven, Stein, Judith, Reback, Cathy J., and Rotheram-Fuller, Erin. Depression ratings, reported sexual risk behaviors, and methamphetamine use: latent growth curve models of positive change among gay and bisexual men in an outpatient treatment program. Experimental and clinical psychopharmacology 2007. 15 (3) 301-307. **Keywords:** Adult/Ambulatory Care/px [Psychology]/*Amphetamine-Related Disorders/px [Psychology]/Amphetamine-Related Disorders/th [Therapy]/*Bisexuality/px [Psychology]/Cognitive Therapy/mt [Methods]/Depression/ep [Epidemiology]/*Depression/px [Psychology]/*Homosexuality,Male/px [Psychology]/Humans/Male/Methamphetamine/ch [Chemistry]/Methamphetamine/ur [Urine]/Models,Psychological/Risk-Taking/*Sexual Behavior/px [Psychology]/Substance Abuse Treatment Centers/Token Economy.

RefID:724. Alessi, Sheila M., Hanson, Tressa, Wieners, Mary, and Petry, Nancy M.. Low-cost contingency management in community clinics: delivering incentives partially in group therapy. Experimental and clinical psychopharmacology 2007. 15 (3) 293-300. **Keywords:** Adult/Cross-Over Studies/Feasibility Studies/Female/Humans/Male/Motivation/Patient Dropouts/Patient Participation/*Psychotherapy,Group/mt [Methods]/*Substance Abuse Treatment Centers/mt [Methods]/*Substance-Related Disorders/rh [Rehabilitation]/Substance-Related Disorders/ur [Urine]/*Token Economy/Treatment Outcome.

RefID:746. Srisurapanont, Manit, Sombatmai, Sangworn, and Boripuntakul, Theerarat. Brief intervention for students with methamphetamine use disorders: a randomized controlled trial. The American journal on addictions / American Academy of Psychiatrists in Alcoholism and Addictions 2007. 16 (2) 111-116. **Keywords:** Adolescent/Adult/Female/Humans/Male/*Methamphetamine/*Psychotherapy,Brief/*Students/px [Psychology]/Students/sn [Statistics & Numerical Data]/Substance-Related Disorders/ep [Epidemiology]/*Substance-Related Disorders/th [Therapy].

RefID:749. Brooner, Robert K., Kidorf, Michael S., King, Van L., Stoller, Kenneth B., Neufeld, Karin J., and Kolodner, Ken. Comparing adaptive stepped care and monetary-based voucher interventions for opioid dependence. Drug and alcohol dependence 2007. 88 Suppl 2 (#Issue#) S14-S23. **Keywords:** *Adaptation,Psychological/Adult/Behavior Therapy/Counseling/Demography/Drug Administration Schedule/Economics/Female/Follow-Up Studies/Humans/Male/*Methadone/tu [Therapeutic Use]/*Motivation/*Narcotics/tu

[Therapeutic Use]/*Opioid-Related Disorders/rh [Rehabilitation]/Patient Acceptance of Health Care/sn [Statistics & Numerical Data]/*Patient-Centered Care/mt [Methods]/Reinforcement (Psychology)/*Token Economy.

RefID:768. Ondersma, Steven J., Svikis, Dace S., and Schuster, Charles R.. Computer-based brief intervention a randomized trial with postpartum women. American journal of preventive medicine 2007. 32 (3) 231-238. **Keywords:** Adult/African Americans/px [Psychology]/*Computers/Female/*Health Promotion/mt [Methods]/Humans/Marijuana Abuse/eh [Ethnology]/Marijuana Abuse/pc [Prevention & Control]/Mothers/px [Psychology]/Motivation/*Postpartum Period/Prospective Studies/Risk Factors/Self Disclosure/Self Efficacy/*Street Drugs/Substance-Related Disorders/eh [Ethnology]/*Substance-Related Disorders/pc [Prevention & Control]/United States.

RefID:775. Rosen, Marc I., Dieckhaus, Kevin, McMahon, Thomas J., Valdes, Barbara, Petry, Nancy M., Cramer, Joyce, and Rounsaville, Bruce. Improved adherence with contingency management. AIDS patient care and STDs 2007. 21 (1) 30-40. **Keywords:** Adult/*Anti-HIV Agents/tu [Therapeutic Use]/*Counseling/mt [Methods]/Female/*HIV Infections/dt [Drug Therapy]/*HIV Infections/px [Psychology]/Humans/Male/Middle Aged/Motivation/*Patient Compliance/Substance Abuse,Intravenous/*Treatment Refusal/Viral Load.

RefID:811. Sullivan, Lynn E., Barry, Declan, Moore, Brent A., Chawarski, Marek C., Tetrault, Jeanette M., Pantalon, Michael V., O'Connor, Patrick G., Schottenfeld, Richard S., and Fiellin, David A.. A trial of integrated buprenorphine/naloxone and HIV clinical care. Clinical infectious diseases : an official publication of the Infectious Diseases Society of America 2006. 43 Suppl 4 (#Issue#) S184-S190. **Keywords:** Administration,Sublingual/Adult/Antiretroviral Therapy,Highly Active/*Buprenorphine/ad [Administration & Dosage]/Dose-Response Relationship,Drug/Drug Administration Schedule/Female/Follow-Up Studies/HIV Infections/co [Complications]/*HIV Infections/di [Diagnosis]/HIV Infections/dt [Drug Therapy]/Humans/Male/Middle Aged/*Naloxone/ad [Administration & Dosage]/*Narcotic Antagonists/ad [Administration & Dosage]/Opioid-Related Disorders/co [Complications]/Opioid-Related Disorders/di [Diagnosis]/*Opioid-Related Disorders/dt [Drug Therapy]/Pilot Projects/Probability/Reference Values/Risk Factors/Treatment Outcome.

RefID:815. Roll, John M., Petry, Nancy M., Stitzer, Maxine L., Brecht, Mary L., Peirce, Jessica M., McCann, Michael J., Blaine, Jack, MacDonald, Marilyn, DiMaria, Joan, Lucero, Leroy, and Kellogg, Scott. Contingency management for the treatment of methamphetamine use disorders. The American journal of psychiatry 2006. 163 (11) 1993-1999. **Keywords:** Adult/Amphetamine-Related Disorders/px [Psychology]/*Amphetamine-Related Disorders/th [Therapy]/Amphetamine-Related Disorders/ur [Urine]/*Behavior Therapy/mt [Methods]/Behavior,Addictive/px [Psychology]/Behavior,Addictive/th [Therapy]/Behavior,Addictive/ur [Urine]/Female/Humans/Male/Reinforcement Schedule/Substance Abuse Detection/Substance Abuse Treatment Centers/*Token Economy/Treatment Outcome.

RefID:819. Lozano, Brian E., Stephens, Robert S., and Roffman, Roger A.. Abstinence and moderate use goals in the treatment of marijuana dependence. Addiction (Abingdon, England) 2006. 101 (11) 1589-1597. **Keywords:** Adult/Analysis of Variance/*Cognitive Therapy/mt [Methods]/Female/Follow-Up Studies/*Goals/Humans/Male/Marijuana Abuse/px [Psychology]/*Marijuana Abuse/rh [Rehabilitation]/Self Efficacy/Treatment Outcome.

RefID:820. Carroll, Kathleen M., Easton, Caroline J., Nich, Charla, Hunkele, Karen A., Neavins, Tara M., Sinha, Rajita, Ford, Haley L., Vitolo, Sally A., Doebrick, Cheryl A., and Rounsaville, Bruce J.. The use of contingency management and motivational/skills-building therapy to treat young adults with marijuana dependence. Journal of consulting and clinical psychology 2006. 74 (5) 955-966. **Keywords:** Adolescent/Adult/*Cognitive Therapy/mt [Methods]/Criminal Law/Female/Humans/Male/*Marijuana Abuse/th [Therapy]/*Motivation/Social Facilitation.

RefID:832. Martino, Steve, Carroll, Kathleen M., Nich, Charla, and Rounsaville, Bruce J.. A randomized controlled pilot study of motivational interviewing for patients with psychotic and drug use disorders. Addiction (Abingdon, England) 2006. 101 (10) 1479-1492. **Keywords:** Adult/Diagnosis,Dual (Psychiatry)/Female/Follow-Up Studies/Humans/*Interview,Psychological/mt [Methods]/Male/*Motivation/Patient Compliance/Pilot Projects/*Psychotic Disorders/di [Diagnosis]/*Substance-Related Disorders/di [Diagnosis]/Treatment Outcome.

RefID:836. Shoptaw, Steven, Huber, Alice, Peck, James, Yang, Xiaowei, Liu, Juanmei, Jeff, Dang, Roll, John, Shapiro, Benjamin, Rotheram-Fuller, Erin, and Ling, Walter. Randomized, placebo-controlled trial of sertraline and contingency management for the treatment of methamphetamine dependence. Drug and alcohol dependence 2006. 85 (1) 12-18. **Keywords:** Adult/*Continuity of Patient Care/sn [Statistics & Numerical Data]/Demography/Depression/di [Diagnosis]/Depression/ep [Epidemiology]/Depression/px [Psychology]/Diagnostic and Statistical Manual of Mental Disorders/Double-Blind Method/Drug Administration Schedule/Female/Humans/Impulse Control Disorders/di [Diagnosis]/Impulse Control Disorders/ep [Epidemiology]/Impulse Control Disorders/px [Psychology]/Length of Stay/sn [Statistics & Numerical Data]/Male/Methamphetamine/ae [Adverse Effects]/Methamphetamine/ur [Urine]/*Methamphetamine/Questionnaires/*Serotonin Uptake Inhibitors/tu [Therapeutic Use]/*Sertraline/tu [Therapeutic Use]/Substance Withdrawal Syndrome/di [Diagnosis]/Substance Withdrawal Syndrome/ep [Epidemiology]/Substance Withdrawal Syndrome/et [Etiology]/*Substance-Related Disorders/rh [Rehabilitation]/Treatment Outcome.

RefID:838. Spoth, Richard L., Clair, Scott, Shin, Chungyeol, and Redmond, Cleve. Long-term effects of universal preventive interventions on methamphetamine use among adolescents. Archives of pediatrics & adolescent medicine 2006. 160 (9) 876-882. **Keywords:** Adolescent/Adolescent Behavior/Child/Female/Humans/Intervention Studies/Iowa/Longitudinal Studies/Male/*Methamphetamine/ad [Administration & Dosage]/Outcome Assessment (Health Care)/Rural Population/*Substance-Related Disorders/pc [Prevention & Control].

RefID:839. Peterson, Peggy L., Baer, John S., Wells, Elizabeth A., Ginzler, Joshua A., and Garrett, Sharon B.. Short-term effects of a brief motivational intervention to reduce alcohol and drug risk among homeless adolescents. Psychology of addictive behaviors : journal of the Society of Psychologists in Addictive Behaviors 2006. 20 (3) 254-264. **Keywords:** Adolescent/Adult/*Alcoholism/pc [Prevention & Control]/Female/*Homeless Persons/sn [Statistics & Numerical Data]/Humans/Male/*Motivation/*Psychotherapy,Brief/mt [Methods]/Substance-Related Disorders/pc [Prevention & Control]/Time Factors.

RefID:851. Fiellin, David A., Pantalon, Michael V., Chawarski, Marek C., Moore, Brent A., Sullivan, Lynn E., O'Connor, Patrick G., and Schottenfeld, Richard S.. Counseling plus buprenorphine-naloxone maintenance therapy for opioid dependence. The New England journal of medicine 2006. 355 (4) 365-374. **Keywords:** Adult/*Buprenorphine/tu [Therapeutic Use]/Cocaine-Related Disorders/co [Complications]/Cocaine-Related Disorders/ep [Epidemiology]/Combined Modality Therapy/*Counseling/Female/Humans/Male/*Naloxone/tu [Therapeutic Use]/*Narcotic Antagonists/tu [Therapeutic Use]/Opioid-Related Disorders/co [Complications]/Opioid-Related Disorders/dt [Drug Therapy]/*Opioid-Related Disorders/th [Therapy].

RefID:864. Gonzalez, Vivian M., Schmitz, Joy M., and DeLaune, Katherine A.. The role of homework in cognitive-behavioral therapy for cocaine dependence. Journal of consulting and clinical psychology 2006. 74 (3) 633-637. **Keywords:** Adult/*Cocaine-Related Disorders/th [Therapy]/*Cognitive Therapy/mt [Methods]/Female/Humans/Male/*Patient Compliance/sn [Statistics & Numerical Data]/Retention (Psychology)/*Workload.

RefID:865. Walker, Denise D., Roffman, Roger A., Stephens, Robert S., Wakana, Kim, Berghuis, James, and Kim, Wakana. Motivational enhancement therapy for adolescent marijuana users: a preliminary randomized controlled trial. Journal of consulting and clinical psychology 2006. 74 (3) 628-632. **Keywords:** Adolescent/Female/Humans/Male/*Marijuana Abuse/th [Therapy]/*Motivation/*Psychotherapy/mt [Methods]/School Health Services/Time Factors.

RefID:866. Petry, Nancy M., Alessi, Sheila M., Carroll, Kathleen M., Hanson, Tressa, MacKinnon, Stephen, Rounsaville, Bruce, and Sierra, Sean. Contingency management treatments: Reinforcing abstinence versus adherence with goal-related activities. Journal of consulting and clinical psychology 2006. 74 (3) 592-601. **Keywords:** Adult/*Cocaine-Related Disorders/pc [Prevention & Control]/Community Mental Health Services/Female/*Goals/Humans/Male/*Patient Compliance/*Reinforcement (Psychology)/*Social Behavior/Substance-Related Disorders/pc [Prevention & Control].

RefID:875. Spoth, Richard, Shin, Chungyeol, Guyll, Max, Redmond, Cleve, and Azevedo, Kari. Universality of effects: an examination of the comparability of long-term family intervention effects on substance use across risk-related subgroups. Prevention science : the official journal of the Society for Prevention Research 2006. 7 (2) 209-224. **Keywords:** Adolescent/Adolescent Behavior/Alcohol Drinking/*Family/Humans/Risk Factors/Substance-Related Disorders/pc

[Prevention & Control]/*Substance-Related Disorders/th [Therapy].

RefID:879. Marsden, John, Stillwell, Garry, Barlow, Helen, Boys, Annabel, Taylor, Colin, Hunt, Neil, and Farrell, Michael. An evaluation of a brief motivational intervention among young ecstasy and cocaine users: no effect on substance and alcohol use outcomes. Addiction (Abingdon, England) 2006. 101 (7) 1014-1026. **Keywords:** Adolescent/Adult/*Alcohol Drinking/pc [Prevention & Control]/Alcohol Drinking/px [Psychology]/Behavior Therapy/mt [Methods]/Cocaine-Related Disorders/px [Psychology]/Cocaine-Related Disorders/th [Therapy]/England/Female/*Hallucinogens/Humans/Interview,Psychological/mt [Methods]/Male/Motivation/*N-Methyl-3,4-methylenedioxyamphetamine/*Psychotherapy,Brief/mt [Methods]/Substance-Related Disorders/px [Psychology]/*Substance-Related Disorders/th [Therapy].

RefID:884. Ledgerwood, David M. and Petry, Nancy M.. Does contingency management affect motivation to change substance use?. Drug and alcohol dependence 2006. 83 (1) 65-72. **Keywords:** Adult/Alcoholism/px [Psychology]/*Alcoholism/rh [Rehabilitation]/Ambulatory Care/*Behavior Therapy/mt [Methods]/Cocaine-Related Disorders/px [Psychology]/*Cocaine-Related Disorders/rh [Rehabilitation]/Community Mental Health Services/Female/Heroin Dependence/px [Psychology]/*Heroin Dependence/rh [Rehabilitation]/Humans/Logistic Models/Male/Middle Aged/*Motivation/Outcome and Process Assessment (Health Care)/sn [Statistics & Numerical Data]/Psychotherapy,Group/*Token Economy.

RefID:888. Baker, Amanda, Bucci, Sandra, Lewin, Terry J., Kay-Lambkin, Frances, Constable, Paul M., and Carr, Vaughan J.. Cognitive-behavioural therapy for substance use disorders in people with psychotic disorders: Randomised controlled trial. The British journal of psychiatry : the journal of mental science 2006. 188 (#Issue#) 439-448. **Keywords:** Adolescent/Adult/*Cognitive Therapy/mt [Methods]/Female/Humans/Male/Middle Aged/New South Wales/Psychotic Disorders/co [Complications]/*Psychotic Disorders/th [Therapy]/Substance-Related Disorders/co [Complications]/*Substance-Related Disorders/th [Therapy]/Treatment Outcome.

RefID:902. Naar-King, Sylvie, Wright, Kathryn, Parsons, Jeffrey T., Frey, Maureen, Templin, Thomas, Lam, Phebe, and Murphy, Debra. Healthy choices: motivational enhancement therapy for health risk behaviors in HIV-positive youth. AIDS education and prevention : official publication of the International Society for AIDS Education 2006. 18 (1) 1-11. **Keywords:** Adolescent/Adult/Child/Choice Behavior/Female/*HIV Infections/pc [Prevention & Control]/Humans/Male/*Motivation/Questionnaires/*Risk-Taking/Substance-Related Disorders/United States.

RefID:909. Fishbein, Diana H., Hyde, Christopher, Eldreth, Diana, Paschall, Mallie J., Hubal, Robert, Das, Abhik, Tarter, Ralph, Ialongo, Nick, Hubbard, Scott, and Yung, Betty. Neurocognitive skills moderate urban male adolescents' responses to preventive intervention materials. Drug and alcohol dependence 2006. 82 (1) 47-60. **Keywords:** Adolescent/Affect/Attitude to Health/*Brain/ph [Physiology]/*Cognition/ph [Physiology]/Decision Making/*Health Promotion/Humans/*Intelligence/Male/*Preventive

Health Services/Psychology/Social Behavior/*Substance-Related Disorders/pc [Prevention & Control]/*Urban Population/sn [Statistics & Numerical Data]/User-Computer Interface.

RefID:965. Rosenblum, Andrew, Magura, Stephen, Kayman, Deborah J., and Fong, Chunki. Motivationally enhanced group counseling for substance users in a soup kitchen: a randomized clinical trial. Drug and alcohol dependence 2005. 80 (1) 91-103. **Keywords:** Adult/Alcoholism/px [Psychology]/*Alcoholism/rh [Rehabilitation]/*Cognitive Therapy/mt [Methods]/Combined Modality Therapy/Community-Institutional Relations/Female/Follow-Up Studies/Food Services/*Homeless Persons/px [Psychology]/Humans/Male/Middle Aged/*Motivation/New York City/Outcome Assessment (Health Care)/Peer Group/*Psychotherapy,Group/mt [Methods]/Referral and Consultation/Social Support/Substance-Related Disorders/px [Psychology]/*Substance-Related Disorders/rh [Rehabilitation]/*Urban Population.

RefID:971. Ball, Samuel A., Cobb-Richardson, Patricia, Connolly, Adrian J., Bujosa, Cesar T., and O'neall, Thomas W.. Substance abuse and personality disorders in homeless drop-in center clients: symptom severity and psychotherapy retention in a randomized clinical trial. Comprehensive psychiatry 2005. 46 (5) 371-379. **Keywords:** Adaptation,Psychological/Adult/*Ambulatory Care/Crime/sn [Statistics & Numerical Data]/Female/*Homeless Persons/sn [Statistics & Numerical Data]/Humans/Interpersonal Relations/Male/Mass Screening/mt [Methods]/Middle Aged/Personality Disorders/di [Diagnosis]/*Personality Disorders/ep [Epidemiology]/*Personality Disorders/th [Therapy]/Psychology/*Psychotherapy/mt [Methods]/Questionnaires/*Retention (Psychology)/Severity of Illness Index/Social Behavior/Substance-Related Disorders/di [Diagnosis]/*Substance-Related Disorders/ep [Epidemiology].

RefID:982. Werch, Chudley E Chad, Moore, Michele M., Diclemente, Carlo C., Owen, Deborah M., Carlson, Joan M., and Jobli, Edessa. Single vs. multiple drug prevention: is more always better?: a pilot study. Substance use & misuse 2005. 40 (8) 1085-1101. **Keywords:** Adolescent/Child/Data Collection/Female/*Health Promotion/mt [Methods]/Humans/Male/Pilot Projects/Program Evaluation/Rural Population/*School Health Services/og [Organization & Administration]/Substance-Related Disorders/ep [Epidemiology]/*Substance-Related Disorders/pc [Prevention & Control]/United States/ep [Epidemiology]/Urban Population.

RefID:1002. Kamon, Jody, Budney, Alan, and Stanger, Catherine. A contingency management intervention for adolescent marijuana abuse and conduct problems. Journal of the American Academy of Child and Adolescent Psychiatry 2005. 44 (6) 513-521. **Keywords:** Adolescent/*Adolescent Behavior/Case Management/*Child Behavior Disorders/th [Therapy]/Cognitive Therapy/mt [Methods]/Combined Modality Therapy/*Family Therapy/mt [Methods]/Feasibility Studies/Female/Follow-Up Studies/Humans/Male/*Marijuana Abuse/th [Therapy]/Motivation/Substance Abuse Detection/*Token Economy/Treatment Outcome.

RefID:1010. Shoptaw, Steven, Reback, Cathy J., Peck, James A., Yang, Xiaowei, Rotheram-Fuller, Erin, Larkins, Sherry, Veniegas, Rosemary C., Freese, Thomas E., and Hucks-Ortiz, Christopher.

Behavioral treatment approaches for methamphetamine dependence and HIV-related sexual risk behaviors among urban gay and bisexual men. Drug and alcohol dependence 2005. 78 (2) 125-134. **Keywords:** Acquired Immunodeficiency Syndrome/pc [Prevention & Control]/*Acquired Immunodeficiency Syndrome/px [Psychology]/Acquired Immunodeficiency Syndrome/tm [Transmission]/Adolescent/Adult/Aged/*Amphetamine-Related Disorders/th [Therapy]/Amphetamine-Related Disorders/ur [Urine]/Bisexuality/*Central Nervous System Stimulants/*Cognitive Therapy/mt [Methods]/Combined Modality Therapy/Homosexuality,Male/Humans/Los Angeles/Male/Methamphetamine/ur [Urine]/*Methamphetamine/Risk-Taking/*Sexual Behavior/px [Psychology]/*Substance Abuse Treatment Centers/Urban Population.

RefID:1019. McCambridge, Jim and Strang, John. Deterioration over time in effect of Motivational Interviewing in reducing drug consumption and related risk among young people. Addiction (Abingdon, England) 2005. 100 (4) 470-478. **Keywords:** Adolescent/Adult/Cluster Analysis/Extinction,Psychological/Female/Humans/*Interview,Psychological/mt [Methods]/Interview,Psychological/st [Standards]/Male/*Motivation/*Psychotherapy,Brief/mt [Methods]/Psychotherapy,Brief/st [Standards]/Questionnaires/Risk/Substance-Related Disorders/pc [Prevention & Control]/*Substance-Related Disorders/th [Therapy].

RefID:1024. Gardner, Lytt I., Metsch, Lisa R., Anderson-Mahoney, Pamela, Loughlin, Anita M., del Rio, Carlos, Strathdee, Steffanie, Sansom, Stephanie L., Siegal, Harvey A., Greenberg, Alan E., Holmberg, Scott D., and Antiretroviral Treatment and Access Study Study Group. Efficacy of a brief case management intervention to link recently diagnosed HIV-infected persons to care. AIDS (London, England) 2005. 19 (4) 423-431. **Keywords:** Adolescent/Adult/Anti-Retroviral Agents/tu [Therapeutic Use]/Case Management/ec [Economics]/*Case Management/og [Organization & Administration]/Female/Follow-Up Studies/*HIV Infections/dt [Drug Therapy]/HIV Infections/ec [Economics]/HIV Infections/vi [Virology]/HIV-1/ip [Isolation & Purification]/Health Care Costs/Health Services/ut [Utilization]/Health Services Accessibility/Health Services Research/Humans/Male/Medical Records/Middle Aged/Multivariate Analysis/RNA,Viral/bl [Blood]/Socioeconomic Factors/United States/Viral Load.

RefID:1026. Baker, Amanda, Lee, Nicole K., Claire, Melissa, Lewin, Terry J., Grant, Tanya, Pohlman, Sonja, Saunders, John B., Kay-Lambkin, Frances, Constable, Paul, Jenner, Linda, and Carr, Vaughan J.. Brief cognitive behavioural interventions for regular amphetamine users: a step in the right direction. Addiction (Abingdon, England) 2005. 100 (3) 367-378. **Keywords:** Adult/*Amphetamine-Related Disorders/th [Therapy]/*Cognitive Therapy/mt [Methods]/Female/Follow-Up Studies/Humans/Male/*Psychotherapy,Brief/mt [Methods]/Treatment Outcome.

RefID:1076. Tucker, Thamizan, Fry, Craig L., Lintzeris, Nick, Baldwin, Simon, Ritter, Alison, Donath, Susan, and Whelan, Greg. Randomized controlled trial of a brief behavioural intervention for reducing hepatitis C virus risk practices among injecting drug users. Addiction (Abingdon, England) 2004. 99 (9) 1157-1166. **Keywords:** Adolescent/Adult/*Behavior

Therapy/mt [Methods]/Female/*Hepatitis C/pc [Prevention & Control]/Hepatitis C/px [Psychology]/Humans/Male/Middle Aged/Patient Satisfaction/Questionnaires/*Risk-Taking/Substance Abuse,Intravenous/px [Psychology]/*Substance Abuse,Intravenous/th [Therapy].

RefID:1084. Marijuana Treatment Project Research Group. Brief treatments for cannabis dependence: findings from a randomized multisite trial. Journal of consulting and clinical psychology 2004. 72 (3) 455-466. **Keywords:** Adult/Female/Humans/Male/*Marijuana Abuse/th [Therapy]/*Motivation/*Psychotherapy,Brief/mt [Methods].

RefID:1090. Strang, John and McCambridge, Jim. Can the practitioner correctly predict outcome in motivational interviewing?. Journal of substance abuse treatment 2004. 27 (1) 83-88. **Keywords:** Adolescent/Adult/Forecasting/Humans/*Interview,Psychological/*Marijuana Abuse/rh [Rehabilitation]/*Motivation/Treatment Outcome.

RefID:1112. Booth, Robert E., Corsi, Karen F., and Mikulich-Gilbertson, Susan K.. Factors associated with methadone maintenance treatment retention among street-recruited injection drug users. Drug and alcohol dependence 2004. 74 (2) 177-185. **Keywords:** Adult/Female/Follow-Up Studies/Humans/Male/*Methadone/tu [Therapeutic Use]/Motivation/*Narcotics/tu [Therapeutic Use]/Patient Selection/*Retention (Psychology)/*Substance Abuse,Intravenous/rh [Rehabilitation]/Treatment Outcome.

RefID:1128. Petry, Nancy M., Tedford, Jacqueline, Austin, Mark, Nich, Charla, Carroll, Kathleen M., and Rounsaville, Bruce J.. Prize reinforcement contingency management for treating cocaine users: how low can we go, and with whom?. Addiction (Abingdon, England) 2004. 99 (3) 349-360. **Keywords:** Adult/Analysis of Variance/Cocaine-Related Disorders/px [Psychology]/*Cocaine-Related Disorders/rh [Rehabilitation]/Costs and Cost Analysis/Female/Humans/Male/Patient Compliance/*Reinforcement Schedule/Token Economy/Treatment Outcome.

RefID:1136. McCambridge, Jim and Strang, John. The efficacy of single-session motivational interviewing in reducing drug consumption and perceptions of drug-related risk and harm among young people: results from a multi-site cluster randomized trial. Addiction (Abingdon, England) 2004. 99 (1) 39-52. **Keywords:** Adolescent/Adult/Alcohol Drinking/pc [Prevention & Control]/Alcohol Drinking/px [Psychology]/Attitude to Health/Female/Follow-Up Studies/Humans/*Interview,Psychological/mt [Methods]/Male/Marijuana Smoking/pc [Prevention & Control]/Marijuana Smoking/px [Psychology]/*Motivation/Psychotherapy,Brief/mt [Methods]/Smoking/pc [Prevention & Control]/Smoking/px [Psychology]/Substance-Related Disorders/px [Psychology]/*Substance-Related Disorders/th [Therapy].

RefID:1143. Leukefeld, Carl, Roberto, Heather, Hiller, Matthew, Webster, Matthew, Logan, T. K., and Staton-Tindall, Michele. HIV prevention among high-risk and hard-to-reach rural residents. Journal of psychoactive drugs 2003. 35 (4) 427-434. **Keywords:** Adult/Aged/Female/*HIV

Infections/ep [Epidemiology]/*HIV Infections/pc [Prevention & Control]/Humans/Male/Middle Aged/*Risk-Taking/*Rural Population/sn [Statistics & Numerical Data]/Sexual Behavior/sn [Statistics & Numerical Data].

RefID:1210. Schumacher, Joseph E., Milby, Jesse B., Wallace, Dennis, Simpson, Cathy, Frison, Sonja, McNamara, Cecelia, and Usdan, Stuart. Diagnostic compared with abstinence outcomes of day treatment and contingency management among cocaine-dependent homeless persons. Experimental and clinical psychopharmacology 2003. 11 (2) 146-157. **Keywords:** Adult/Ambulatory Care/mt [Methods]/Ambulatory Care/px [Psychology]/Ambulatory Care/sn [Statistics & Numerical Data]/*Ambulatory Care/Chi-Square Distribution/*Cocaine-Related Disorders/di [Diagnosis]/Cocaine-Related Disorders/px [Psychology]/*Cocaine-Related Disorders/th [Therapy]/Confidence Intervals/Disease Management/Female/Follow-Up Studies/Homeless Persons/px [Psychology]/Homeless Persons/sn [Statistics & Numerical Data]/*Homeless Persons/Humans/Male/Middle Aged/Odds Ratio/Treatment Outcome.

RefID:1220. Messina, Nena, Farabee, David, and Rawson, Richard. Treatment responsivity of cocaine-dependent patients with antisocial personality disorder to cognitive-behavioral and contingency management interventions. Journal of consulting and clinical psychology 2003. 71 (2) 320-329. **Keywords:** Adult/*Antisocial Personality Disorder/co [Complications]/*Cocaine-Related Disorders/co [Complications]/*Cocaine-Related Disorders/th [Therapy]/*Cognitive Therapy/mt [Methods]/Female/Follow-Up Studies/Humans/Male/Treatment Outcome.

RefID:1224. Santisteban, Daniel A., Coatsworth, J. Douglas, Perez-Vidal, Angel, Kurtines, William M., Schwartz, Seth J., LaPerriere, Arthur, and Szapocznik, Jose. Efficacy of brief strategic family therapy in modifying Hispanic adolescent behavior problems and substance use. Journal of family psychology : JFP : journal of the Division of Family Psychology of the American Psychological Association (Division 43) 2003. 17 (1) 121-133. **Keywords:** Adolescent/Aggression/px [Psychology]/Child Behavior Disorders/px [Psychology]/*Child Behavior Disorders/rh [Rehabilitation]/*Family Therapy/Female/*Hispanic Americans/px [Psychology]/Humans/Male/Marijuana Abuse/px [Psychology]/*Marijuana Abuse/rh [Rehabilitation]/*Psychotherapy,Brief/Psychotherapy,Group/Treatment Outcome.

RefID:1233. Davis, Tania M., Baer, John S., Saxon, Andrew J., and Kivlahan, Daniel R.. Brief motivational feedback improves post-incarceration treatment contact among veterans with substance use disorders. Drug and alcohol dependence 2003. 69 (2) 197-203. **Keywords:** Feedback,Psychological/Female/Humans/*Interview,Psychological/mt [Methods]/Male/Middle Aged/*Motivation/Office Visits/sn [Statistics & Numerical Data]/*Prisoners/px [Psychology]/Severity of Illness Index/*Substance-Related Disorders/th [Therapy]/*Veterans/px [Psychology].

RefID:1259. Dishion, Thomas J., Kavanagh, Kathryn, Schneiger, Alison, Nelson, Sarah, and Kaufman, Noah K.. Preventing early adolescent substance use: a family-centered strategy for the public middle school. Prevention science : the official journal of the Society for Prevention Research 2002. 3 (3) 191-201. **Keywords:** Adolescent/Child/*Family

Therapy/Female/Humans/Logistic Models/Male/Outcome and Process Assessment (Health Care)/Parenting/px [Psychology]/Risk Factors/*School Health Services/og [Organization & Administration]/*Substance-Related Disorders/pc [Prevention & Control]/Substance-Related Disorders/px [Psychology].

RefID:1293. Eggert, Leona L., Thompson, Elaine A., Randell, Brooke P., and Pike, Kenneth C.. Preliminary effects of brief school-based prevention approaches for reducing youth suicide--risk behaviors, depression, and drug involvement. Journal of child and adolescent psychiatric nursing : official publication of the Association of Child and Adolescent Psychiatric Nurses, Inc 2002. 15 (2) 48-64. **Keywords:** Adolescent/Adult/*Crisis Intervention/mt [Methods]/Depression/pc [Prevention & Control]/Female/Humans/Male/Multivariate Analysis/Northwestern United States/Regression Analysis/*School Health Services/Substance-Related Disorders/pc [Prevention & Control]/*Suicide/pc [Prevention & Control].

RefID:1298. D'Amico, Elizabeth J. and Fromme, Kim. Brief prevention for adolescent risk-taking behavior. Addiction (Abingdon, England) 2002. 97 (5) 563-574. **Keywords:** Adolescent/*Adolescent Behavior/Adult/Alcohol Drinking/pc [Prevention & Control]/Automobile Driving/px [Psychology]/Female/Follow-Up Studies/*Health Promotion/mt [Methods]/Humans/Male/Peer Group/*Risk-Taking.

RefID:1305. Fernandes, Leona C., Kilicarslan, Tansel, Kaplan, Howard L., Tyndale, Rachel F., Sellers, Edward M., and Romach, Myroslava K.. Treatment of codeine dependence with inhibitors of cytochrome P450 2D6. Journal of clinical psychopharmacology 2002. 22 (3) 326-329. **Keywords:** Adult/Aged/*Analgesics,Opioid/ae [Adverse Effects]/*Codeine/ae [Adverse Effects]/*Cytochrome P-450 CYP2D6/ai [Antagonists & Inhibitors]/Cytochrome P-450 CYP2D6/me [Metabolism]/Double-Blind Method/*Enzyme Inhibitors/tu [Therapeutic Use]/Female/Fluoxetine/tu [Therapeutic Use]/Humans/Male/Middle Aged/Pilot Projects/Quinidine/tu [Therapeutic Use]/Serotonin Uptake Inhibitors/tu [Therapeutic Use]/*Substance-Related Disorders/dt [Drug Therapy]/Substance-Related Disorders/en [Enzymology]/Substance-Related Disorders/px [Psychology].

RefID:1329. Carroll, K. M., Libby, B., Sheehan, J., and Hyland, N.. Motivational interviewing to enhance treatment initiation in substance abusers: an effectiveness study. The American journal on addictions / American Academy of Psychiatrists in Alcoholism and Addictions 2001. 10 (4) 335-339. **Keywords:** Connecticut/Female/Humans/Interview,Psychological/Male/*Motivation/*Patient Acceptance of Health Care/px [Psychology]/Persuasive Communication/*Substance-Related Disorders/px [Psychology]/Substance-Related Disorders/th [Therapy].

RefID:1330. Morgenstern, J., Blanchard, K. A., Morgan, T. J., Labouvie, E., and Hayaki, J.. Testing the effectiveness of cognitive-behavioral treatment for substance abuse in a community setting: within treatment and posttreatment findings. Journal of consulting and clinical psychology 2001. 69 (6) 1007-1017. **Keywords:** Adult/*Cognitive Therapy/mt [Methods]/Community Mental Health Services/Female/Humans/Male/Program

Evaluation/Questionnaires/Severity of Illness Index/Substance-Related Disorders/di [Diagnosis]/Substance-Related Disorders/ep [Epidemiology]/*Substance-Related Disorders/th [Therapy]/Time Factors/Treatment Outcome.

RefID:1334. Liddle, H. A., Dakof, G. A., Parker, K., Diamond, G. S., Barrett, K., and Tejeda, M.. Multidimensional family therapy for adolescent drug abuse: results of a randomized clinical trial. The American journal of drug and alcohol abuse 2001. 27 (4) 651-688. **Keywords:** Adolescent/Alcoholism/ep [Epidemiology]/*Alcoholism/rh [Rehabilitation]/*Family Therapy/mt [Methods]/Female/Follow-Up Studies/Humans/Incidence/Juvenile Delinquency/pc [Prevention & Control]/Male/Marijuana Abuse/ep [Epidemiology]/*Marijuana Abuse/rh [Rehabilitation]/Peer Group/Psychotherapy,Group/mt [Methods]/Risk Factors/Treatment Outcome.

RefID:1339. Waldron, H. B., Slesnick, N., Brody, J. L., Turner, C. W., and Peterson, T. R.. Treatment outcomes for adolescent substance abuse at 4- and 7-month assessments. Journal of consulting and clinical psychology 2001. 69 (5) 802-813. **Keywords:** Adolescent/*Cognitive Therapy/mt [Methods]/Female/Follow-Up Studies/Humans/Male/*Substance-Related Disorders/th [Therapy]/Treatment Outcome.

RefID:1342. Baker, A., Boggs, T. G., and Lewin, T. J.. Randomized controlled trial of brief cognitive-behavioural interventions among regular users of amphetamine. Addiction (Abingdon, England) 2001. 96 (9) 1279-1287. **Keywords:** Adult/*Amphetamine-Related Disorders/rh [Rehabilitation]/Analysis of Variance/*Cognitive Therapy/mt [Methods]/Female/Humans/Male/New South Wales/Patient Participation/*Psychotherapy,Brief/mt [Methods]/Treatment Outcome.

RefID:1352. Copeland, J., Swift, W., Roffman, R., and Stephens, R.. A randomized controlled trial of brief cognitive-behavioral interventions for cannabis use disorder. Journal of substance abuse treatment 2001. 21 (2) 55-56. **Keywords:** Adult/Australia/*Cognitive Therapy/Female/Humans/Male/*Marijuana Abuse/px [Psychology]/*Marijuana Abuse/th [Therapy]/Patient Compliance/px [Psychology]/Self-Assessment/Time Factors/Treatment Outcome.

RefID:1354. Spoth, R. L., Redmond, C., and Shin, C.. Randomized trial of brief family interventions for general populations: adolescent substance use outcomes 4 years following baseline. Journal of consulting and clinical psychology 2001. 69 (4) 627-642. **Keywords:** Adolescent/Adult/Child/*Family Therapy/Female/Follow-Up Studies/Humans/Male/*Psychotherapy,Brief/Substance-Related Disorders/px [Psychology]/*Substance-Related Disorders/rh [Rehabilitation].

RefID:1385. Copeland, J., Swift, W., and Rees, V.. Clinical profile of participants in a brief intervention program for cannabis use disorder. Journal of substance abuse treatment 2001. 20 (1) 45-52. **Keywords:** Adolescent/Adult/*Cannabis/ae [Adverse Effects]/*Cognitive Therapy/Female/Humans/Male/Marijuana Abuse/px [Psychology]/*Marijuana Abuse/th

[Therapy]/Middle Aged/New South Wales/Psychiatric Status Rating Scales/*Psychotherapy,Brief/Recurrence/pc [Prevention & Control]/Severity of Illness Index/Treatment Outcome.

RefID:1425. Conrod, P. J., Stewart, S. H., Pihl, R. O., Cote, S., Fontaine, V., and Dongier, M.. Efficacy of brief coping skills interventions that match different personality profiles of female substance abusers. Psychology of addictive behaviors : journal of the Society of Psychologists in Addictive Behaviors 2000. 14 (3) 231-242. **Keywords:** *Adaptation,Psychological/Adult/Alcoholism/px [Psychology]/*Alcoholism/rh [Rehabilitation]/*Cognitive Therapy/mt [Methods]/Female/Humans/Middle Aged/Motivation/*Personality Inventory/*Psychotherapy,Brief/mt [Methods]/Substance-Related Disorders/px [Psychology]/*Substance-Related Disorders/rh [Rehabilitation]/Treatment Outcome.

RefID:1428. Schuler, M. E., Nair, P., Black, M. M., and Kettinger, L.. Mother-infant interaction: effects of a home intervention and ongoing maternal drug use. Journal of clinical child psychology 2000. 29 (3) 424-431. **Keywords:** Adult/Child Abuse/px [Psychology]/Female/*Home Care Services/Humans/Infant,Newborn/Intervention Studies/Male/*Mother-Child Relations/*Parenting/Pregnancy/Random Allocation/*Substance-Related Disorders/co [Complications]/Substance-Related Disorders/th [Therapy].

RefID:1440. Downey, K. K., Helmus, T. C., and Schuster, C. R.. Treatment of heroin-dependent poly-drug abusers with contingency management and buprenorphine maintenance. Experimental and clinical psychopharmacology 2000. 8 (2) 176-184. **Keywords:** Adolescent/Adult/Breath Tests/*Buprenorphine/tu [Therapeutic Use]/Cocaine-Related Disorders/co [Complications]/Cocaine-Related Disorders/px [Psychology]/Cocaine-Related Disorders/rh [Rehabilitation]/*Cognitive Therapy/Combined Modality Therapy/Female/Heroin Dependence/co [Complications]/Heroin Dependence/px [Psychology]/*Heroin Dependence/rh [Rehabilitation]/Humans/Male/Middle Aged/*Narcotic Antagonists/tu [Therapeutic Use]/Psychiatric Status Rating Scales/Substance Abuse Detection/Substance-Related Disorders/co [Complications]/Substance-Related Disorders/px [Psychology]/*Substance-Related Disorders/rh [Rehabilitation]/Time Factors/Treatment Outcome.

RefID:1441. Milby, J. B., Schumacher, J. E., McNamara, C., Wallace, D., Usdan, S., McGill, T., and Michael, M.. Initiating abstinence in cocaine abusing dually diagnosed homeless persons. Drug and alcohol dependence 2000. 60 (1) 55-67. **Keywords:** Adult/Analysis of Variance/Cocaine-Related Disorders/px [Psychology]/*Cocaine-Related Disorders/th [Therapy]/Cocaine-Related Disorders/ur [Urine]/Diagnosis,Dual (Psychiatry)/px [Psychology]/Employment/px [Psychology]/Female/*Homeless Persons/px [Psychology]/Housing/Humans/Male/Mental Disorders/px [Psychology]/*Mental Disorders/th [Therapy]/Mental Disorders/ur [Urine]/Middle Aged/Statistics,Nonparametric/Treatment Outcome.

RefID:1460. Volpicelli, J. R., Markman, I., Monterosso, J., Filing, J., and O'Brien, C. P.. Psychosocially enhanced treatment for cocaine-dependent mothers: evidence of efficacy.

Journal of substance abuse treatment 2000. 18 (1) 41-49. **Keywords:** Adult/Case Management/Cocaine-Related Disorders/px [Psychology]/*Cocaine-Related Disorders/th [Therapy]/Female/Humans/*Social Support.

RefID:1476. Longshore, D., Grills, C., and Annon, K.. Effects of a culturally congruent intervention on cognitive factors related to drug-use recovery. Substance use & misuse 1999. 34 (9) 1223-1241. **Keywords:** Adult/*African Americans/px [Psychology]/*Behavior Therapy/mt [Methods]/Cognitive Therapy/mt [Methods]/*Crisis Intervention/mt [Methods]/*Culture/European Continental Ancestry Group/px [Psychology]/Female/Humans/Male/*Substance-Related Disorders/eh [Ethnology]/*Substance-Related Disorders/th [Therapy]/Treatment Outcome/United States.

RefID:1497. Farrow, J. A., Watts, D. H., Krohn, M. A., and Olson, H. C.. Pregnant adolescents in chemical dependency treatment. Description and outcomes. Journal of substance abuse treatment 1999. 16 (2) 157-161. **Keywords:** Adolescent/Adult/Age Factors/Ambulatory Care/Female/Humans/Infant,Newborn/Marital Status/Obstetric Labor Complications/ep [Epidemiology]/Pregnancy/Pregnancy Complications/ep [Epidemiology]/Pregnancy Outcome/Pregnancy in Adolescence/px [Psychology]/*Pregnancy in Adolescence/sn [Statistics & Numerical Data]/Residential Treatment/Sex Factors/Smoking/ep [Epidemiology]/Substance Abuse Treatment Centers/Substance-Related Disorders/ep [Epidemiology]/*Substance-Related Disorders/th [Therapy]/Treatment Outcome.

RefID:1544. Booth, R. E., Kwiatkowski, C., Iguchi, M. Y., Pinto, F., and John, D.. Facilitating treatment entry among out-of-treatment injection drug users. Public health reports (Washington, D.C.: 1974) 1998. 113 Suppl 1 (#Issue#) 116-128. **Keywords:** Adolescent/Adult/Aged/*Behavior Therapy/Colorado/Female/*Health Promotion/*Health Services Accessibility/Humans/Male/Middle Aged/*Motivation/Needles/Substance Abuse Treatment Centers/*Substance-Related Disorders/px [Psychology]/*Substance-Related Disorders/th [Therapy].

RefID:1553. Rhoades, H. M., Creson, D., Elk, R., Schmitz, J., and Grabowski, J.. Retention, HIV risk, and illicit drug use during treatment: methadone dose and visit frequency. American journal of public health 1998. 88 (1) 34-39. **Keywords:** Adult/Behavior Therapy/Female/HIV Infections/Humans/Male/*Methadone/ad [Administration & Dosage]/Middle Aged/Opioid-Related Disorders/ep [Epidemiology]/*Opioid-Related Disorders/th [Therapy]/Patient Dropouts/sn [Statistics & Numerical Data]/Risk Factors/Risk-Taking/*Street Drugs/Substance Abuse Detection/Survival Analysis.

RefID:1658. Henggeler, S. W., Pickrel, S. G., Brondino, M. J., and Crouch, J. L.. Eliminating (almost) treatment dropout of substance abusing or dependent delinquents through home-based multisystemic therapy. The American journal of psychiatry 1996. 153 (3) 427-428. **Keywords:** Adolescent/Combined Modality Therapy/Community Mental Health Services/ut [Utilization]/Family Therapy/Female/*Home Care Services/Humans/Juvenile Delinquency/rh [Rehabilitation]/Male/Patient Dropouts/Substance-Related Disorders/rh

[Rehabilitation]/*Substance-Related Disorders/th [Therapy].

RefID:1666. Nunes, E. V., McGrath, P. J., Quitkin, F. M., Ocepek-Welikson, K., Stewart, J. W., Koenig, T., Wager, S., and Klein, D. F.. Imipramine treatment of cocaine abuse: possible boundaries of efficacy. Drug and alcohol dependence 1995. 39 (3) 185-195. **Keywords:** Adolescent/Adult/Aged/Antidepressive Agents,Tricyclic/ae [Adverse Effects]/*Antidepressive Agents,Tricyclic/tu [Therapeutic Use]/Cocaine/ae [Adverse Effects]/*Cocaine/Combined Modality Therapy/Comorbidity/Depressive Disorder/px [Psychology]/Depressive Disorder/rh [Rehabilitation]/Double-Blind Method/Euphoria/de [Drug Effects]/Female/Humans/Imipramine/ae [Adverse Effects]/*Imipramine/tu [Therapeutic Use]/Male/Middle Aged/Motivation/Substance Withdrawal Syndrome/px [Psychology]/Substance Withdrawal Syndrome/rh [Rehabilitation]/Substance-Related Disorders/px [Psychology]/*Substance-Related Disorders/rh [Rehabilitation]/Treatment Outcome.

RefID:1804. Higgins, S. T., Delaney, D. D., Budney, A. J., Bickel, W. K., Hughes, J. R., Foerg, F., and Fenwick, J. W.. A behavioral approach to achieving initial cocaine abstinence. The American journal of psychiatry 1991. 148 (9) 1218-1224. **Keywords:** Adult/Ambulatory Care/Attitude to Health/*Behavior Therapy/mt [Methods]/*Cocaine/Counseling/Evaluation Studies as Topic/Female/Humans/Male/Patient Dropouts/Pilot Projects/Reinforcement (Psychology)/Reinforcement,Social/Substance-Related Disorders/pc [Prevention & Control]/Substance-Related Disorders/px [Psychology]/*Substance-Related Disorders/th [Therapy]/Time Factors.

RefID:1807. Weddington, W. W. J., Brown, B. S., Haertzen, C. A., Hess, J. M., Mahaffey, J. R., Kolar, A. F., and Jaffe, J. H.. Comparison of amantadine and desipramine combined with psychotherapy for treatment of cocaine dependence. The American journal of drug and alcohol abuse 1991. 17 (2) 137-152. **Keywords:** Adult/*Amantadine/ad [Administration & Dosage]/Amantadine/pk [Pharmacokinetics]/*Cocaine/Combined Modality Therapy/*Desipramine/ad [Administration & Dosage]/Desipramine/pk [Pharmacokinetics]/Dose-Response Relationship,Drug/Female/Follow-Up Studies/Humans/Male/*Psychotherapy/Single-Blind Method/Substance-Related Disorders/bl [Blood]/Substance-Related Disorders/px [Psychology]/*Substance-Related Disorders/rh [Rehabilitation].

RefID:1808. Halikas, J. A., Crosby, R. D., Carlson, G. A., Crea, F., Graves, N. M., and Bowers, L. D.. Cocaine reduction in unmotivated crack users using carbamazepine versus placebo in a short-term, double-blind crossover design. Clinical pharmacology and therapeutics 1991. 50 (1) 81-95. **Keywords:** Administration,Oral/Adult/Carbamazepine/bl [Blood]/*Carbamazepine/tu [Therapeutic Use]/Chromatography,High Pressure Liquid/*Cocaine/Double-Blind Method/Educational Status/Humans/Male/Marriage/Middle Aged/Motivation/*Substance-Related Disorders/dt [Drug Therapy]/Substance-Related Disorders/px [Psychology]/Substance-Related Disorders/ur [Urine].

RefID:1823. McAuliffe, W. E.. A randomized controlled trial of recovery training and self-help for opioid addicts in New England and Hong Kong. Journal of psychoactive drugs 1990. 22 (2) 197-209. **Keywords:** Crime/Employment/Hong Kong/Humans/Length of Stay/New England/*Opioid-Related Disorders/th [Therapy]/Outpatients/Randomized Controlled Trials as Topic.

RefID:1874. Nathan, R. G., Robinson, D., Cherek, D. R., Sebastian, C. S., Hack, M., and Davison, S.. Alternative treatments for withdrawing the long-term benzodiazepine user: a pilot study. The International journal of the addictions 1986. 21 (2) 195-211. **Keywords:** Adult/*Anti-Anxiety Agents/Benzodiazepines/Biofeedback,Psychology/Combined Modality Therapy/Counseling/Electromyography/Female/Galvanic Skin Response/Humans/Long-Term Care/Middle Aged/Muscle Relaxation/Pilot Projects/Skin Temperature/Substance Withdrawal Syndrome/rh [Rehabilitation]/*Substance-Related Disorders/rh [Rehabilitation].

RefID:1935. Milburn, N. G., Iribarren, F. J., Rice, E., Lightfoot, M., Solorio, R., Rotheram-Borus, M. J., Desmond, K., Lee, A., Alexander, K., Maresca, K., Eastmen, K., Arnold, E. M., and Duan, N.. A family intervention to reduce sexual risk behavior, substance use, and delinquency among newly homeless youth. Journal of Adolescent Health 2012. 50 (4) 358-364. **Keywords:** Adolescent/article/cannabis addiction/th [Therapy]/controlled study/Family Therapy/Female/high risk behavior/homelessness/human/*juvenile delinquency/th [Therapy]/major clinical study/Male/priority journal/randomized controlled trial/Self Report/*Sexual Behavior/*substance abuse.

RefID:1946. Winters, K. C., Fahnhorst, T., Botzet, A., Lee, S., and Lalone, B.. Brief intervention for drug-abusing adolescents in a school setting: Outcomes and mediating factors. Journal of substance abuse treatment 2012. 42 (3) 279-288. **Keywords:** Adolescent/Adult/alcohol abstinence/*alcohol abuse/alcohol consumption/Alcoholism/article/*brief intervention/Child/child parent relation/controlled study/*Counseling/*drug abuse/Female/follow up/Health Behavior/human/major clinical study/Male/outcome assessment/parental behavior/patient assessment/priority journal/Problem Solving/randomized controlled trial/school/*alcohol/*Cannabis.

RefID:2027. Tyrer, P., Miloseska, K., Whittington, C., Ranger, M., Khaleel, I., Crawford, M., North, B., and Barrett, B.. Nidotherapy in the treatment of substance misuse, psychosis and personality disorder: Secondary analysis of a controlled trial. Psychiatrist 2011. 35 (1) 9-14. **Keywords:** *alternative medicine/article/clinical article/Comorbidity/controlled study/Cost Control/health care cost/hospital bed utilization/human/*nidotherapy/outcome assessment/personality disorder/th [Therapy]/*psychosis/th [Therapy]/randomization/secondary analysis/social status/*substance abuse/Treatment Outcome.

RefID:2088. Sherman, S. G., Sutcliffe, C. G., Srirojn, B., German, D., Thomson, N., Aramrattana, A., and Celentano, D. D.. Predictors and consequences of incarceration among a sample of young Thai methamphetamine users. Drug and alcohol review 2010. 29 (4) 399-405. **Keywords:** Adolescent/Adult/age/article/case control study/clinical trial/comparative study/controlled

clinical trial/controlled study/*drug abuse/co [Complication]/ethnology/Female/follow up/Forecasting/high risk behavior/human/longitudinal study/Male/*prisoner/psychological aspect/randomized controlled trial/Thailand/*Methamphetamine.

RefID:2111. Martin, G. and Copeland, J.. Brief intervention for regular ecstasy (MDMA) users: Pilot randomized trial of a check-up model. Journal of Substance Use 2010. 15 (2) 131-142. **Keywords:** abstinence/Adult/article/clinical article/clinical trial/*Cognitive Therapy/controlled clinical trial/controlled study/Diagnostic and Statistical Manual of Mental Disorders/*drug dependence treatment/*Ecstasy Check up/Female/human/intervention study/Male/*Motivation/priority journal/randomized controlled trial/scoring system/structured interview/symptom/therapy delay/Treatment Outcome/*3,4 methylenedioxymethamphetamine/alcohol/amphetamine/Cannabis/Cocaine.

RefID:2114. Rigter, H., Pelc, I., Tossmann, P., Phan, O., Grichting, E., Hendriks, V., and Rowe, C.. INCANT: A transnational randomized trial of Multidimensional Family Therapy versus treatment as usual for adolescents with cannabis use disorder. BMC psychiatry 2010. 10 (#Issue#) #Start Page#-#End Page#. **Keywords:** Adolescent/Adult/article/behavior disorder/Belgium/*cannabis addiction/th [Therapy]/clinical trial/Cognitive Therapy/controlled clinical trial/controlled study/Europe/family functioning/*Family Therapy/France/Germany/government/human/intermethod comparison/intervention study/leisure/major clinical study/mental function/multicenter study/Peer Group/phase 3 clinical trial/randomized controlled trial/Sample Size/social psychology/substance abuse/Switzerland.

RefID:2125. Chad Werch, C. E., Bian, H., DiClemente, C. C., Moore, M. J., Thombs, D., Ames, S. C., Huang, I.-C., and Pokorny, S.. A brief image-based prevention intervention for adolescents. Psychology of Addictive Behaviors 2010. 24 (1) 170-175. **Keywords:** Adolescent/alcohol consumption/alcoholism/pc [Prevention]/article/*child psychiatry/cigarette smoking/clinical trial/controlled clinical trial/controlled study/drug dependence/pc [Prevention]/Female/Health Behavior/*Health Education/high school student/human/major clinical study/Male/*Preventive Medicine/randomized controlled trial/*substance abuse.

RefID:2129. Werch, C. E., Moore, M. J., Bian, H., DiClemente, C. C., Huang, I.-C., Ames, S. C., Thombs, D., Weiler, R. M., and Pokorny, S. B.. Are effects from a brief multiple behavior intervention for college students sustained over time?. Preventive medicine 2010. 50 (1-2) 30-34. **Keywords:** Adult/*Alcoholism/th [Therapy]/article/*Behavior Therapy/*cannabis addiction/th [Therapy]/car driving/clinical trial/college student/controlled clinical trial/controlled study/Drinking Behavior/Exercise/Female/follow up/habit/Health Behavior/human/major clinical study/Male/medical record/priority journal/Quality of Life/randomized controlled trial/religion/Self Concept/Social Behavior/Treatment Outcome/United States.

RefID:2130. Koblin, B. A., Bonner, S., Hoover, D. R., Xu, G., Lucy, D., Fortin, P., Putnam, S., and Latka, M. H.. A randomized trial of enhanced HIV risk-reduction and vaccine trial education

interventions among HIV-negative, high-risk women who use noninjection drugs: The UNITY study. Journal of Acquired Immune Deficiency Syndromes 2010. 53 (3) 378-387. **Keywords:** Adult/article/clinical trial/controlled clinical trial/controlled study/*drug abuse/drug efficacy/drug safety/Female/follow up/high risk patient/human/*Human immunodeficiency virus infection/dt [Drug Therapy]/*Human immunodeficiency virus infection/ep [Epidemiology]/*Human immunodeficiency virus infection/pc [Prevention]/infection risk/intervention study/major clinical study/patient education/priority journal/randomized controlled trial/risk reduction/Unsafe Sex/*Human immunodeficiency virus vaccine/ct [Clinical Trial]/*Human immunodeficiency virus vaccine/dt [Drug Therapy].

RefID:2206. Stice, E., Rohde, P., Seeley, J. R., and Gau, J. M.. Brief Cognitive-Behavioral Depression Prevention Program for High-Risk Adolescents Outperforms Two Alternative Interventions: A Randomized Efficacy Trial. Journal of consulting and clinical psychology 2008. 76 (4) 595-606. **Keywords:** Adolescent/Adult/article/clinical trial/*Cognitive Therapy/controlled clinical trial/controlled study/*depression/pc [Prevention]/*depression/rh [Rehabilitation]/*Depression/th [Therapy]/Female/follow up/high risk patient/human/intervention study/major clinical study/Male/Psychotherapy/randomization/randomized controlled trial/rehabilitation care/social adaptation/substance abuse.

RefID:2217. Burleson, J. A. and Kaminer, Y.. Does temperament moderate treatment response in adolescent substance use disorders?. Substance Abuse 2008. 29 (2) 89-95. **Keywords:** adaptive behavior/Adolescent/alcohol consumption/article/Behavior Therapy/Circadian Rhythm/clinical assessment/Cognitive Therapy/*drug dependence/th [Therapy]/Female/human/major clinical study/Male/mental function/mood/patient education/physical activity/Principal Component Analysis/psychoeducation/rating scale/Social Behavior/*Temperament/*treatment response.

RefID:2237. Hjorthoj, C., Fohlmann, A., Larsen, A.-M., Madsen, M. T. R., Vesterager, L., Gluud, C., Arendt, M. C., and Nordentoft, M.. Design paper: The CapOpus trial: A randomized, parallel-group, observer-blinded clinical trial of specialized addiction treatment versus treatment as usual for young patients with cannabis abuse and psychosis. Trials 2008. 9 (#Issue#) #Start Page#-#End Page#. **Keywords:** article/*cannabis addiction/di [Diagnosis]/*cannabis addiction/th [Therapy]/case manager/clinical trial/Cognition/Cognitive Therapy/community mental health center/controlled clinical trial/controlled study/cost benefit analysis/disease association/disease severity/Early Diagnosis/Family/follow up/group therapy/health program/human/International Classification of Diseases/interview/Motivation/parallel design/psychoeducation/*psychosis/di [Diagnosis]/*psychosis/dt [Drug Therapy]/*psychosis/th [Therapy]/Quality of Life/randomized controlled trial/single blind procedure/social adaptation/social interaction/treatment response/neuroleptic agent/ct [Clinical Trial]/neuroleptic agent/dt [Drug Therapy].

RefID:2238. Barber, J. P., Gallop, R., Crits-Christoph, P., Barrett, M. S., Klostermann, S., McCarthy, K. S., and Sharpless, B. A.. THE ROLE OF THE ALLIANCE AND TECHNIQUES IN

PREDICTING OUTCOME OF SUPPORTIVE-EXPRESSIVE DYNAMIC THERAPY FOR COCAINE DEPENDENCE. Psychoanalytic Psychology 2008. 25 (3) 461-482. **Keywords:** article/*cocaine dependence/th [Therapy]/Counseling/human/psychodynamics/Psychotherapy/supportive expressive therapy/Treatment Outcome.

RefID:2294. Li, S., Armstrong, M. S., Chaim, G., Kelly, C., and Shenfeld, J.. Group and individual couple treatment for substance abuse clients: A pilot study. American Journal of Family Therapy 2007. 35 (3) 221-233. **Keywords:** Adult/Aged/article/Brief Symptom Inventory/clinical effectiveness/clinical trial/Cognitive Therapy/controlled clinical trial/controlled study/*drug dependence/th [Therapy]/Dyadic Adjustment Scale/Family Therapy/Female/follow up/human/individualization/intermethod comparison/major clinical study/Male/outcome assessment/pilot study/randomized controlled trial/substance abuse.

RefID:2300. Kadden, R. M., Litt, M. D., Kabela-Cormier, E., and Petry, N. M.. Abstinence rates following behavioral treatments for marijuana dependence. Addictive behaviors 2007. 32 (6) 1220-1236. **Keywords:** *abstinence/Adult/article/*Behavior Therapy/*cannabis addiction/th [Therapy]/clinical trial/*Cognitive Therapy/*contingency management/controlled clinical trial/controlled study/coping behavior/drug dependence/th [Therapy]/drug dependence treatment/drug withdrawal/Female/follow up/human/major clinical study/Male/*motivational enhancement therapy/reinforcement/Treatment Outcome.

RefID:2421. Babor, T. F., Carroll, K., Christiansen, K., Donaldson, J., Herrell, J., Kadden, R., Litt, M., McRee, B., Miller, M., Roffman, R., Solowji, N., Steinberg, K., Stephens, R., and Vendetti, J.. Brief treatments for cannabis dependence: Findings from a randomized multisite trial. Journal of consulting and clinical psychology 2004. 72 (3) 455-466. **Keywords:** Adult/article/Behavior Therapy/*cannabis addiction/et [Etiology]/clinical feature/clinical trial/controlled clinical trial/controlled study/Female/follow up/human/major clinical study/Male/medical assessment/Motivation/motivational enhancement therapy/multicenter study/psychiatric diagnosis/randomization/randomized controlled trial/Treatment Outcome/*Cannabis.

RefID:2435. Dishion, T. J., Nelson, S. E., and Kavanagh, K.. The Family Check-Up with high-risk young adolescents: Preventing early-onset substance use by parent monitoring. Behavior Therapy 2003. 34 (4) 553-571. **Keywords:** Adolescent/article/Behavior Therapy/*Child Behavior/child development/clinical article/clinical trial/Cognition/controlled clinical trial/controlled study/*family check up/*family counseling/Family Therapy/Female/follow up/*high risk population/home/human/juvenile/Male/onset age/randomized controlled trial/skill/*substance abuse/videorecording.

RefID:2560. Crits-Christoph, P., Siqueland, L., Blaine, J., Frank, A., Luborsky, L., Onken, L. S., Muenz, L. R., Thase, M. E., Weiss, R. D., Gastfriend, D. R., Woody, G. E., Barber, J. P., Butler, S. F., Daley, D., Salloum, I., Bishop, S., Najavits, L. M., Lis, J., Mercer, D., Griffin, M. L., Moras, K., and Beck, A. T.. Psychosocial treatments for cocaine dependence: National Institute on Drug Abuse Collaborative Cocaine Treatment Study. Archives of general psychiatry 1999. 56 (6) 493-502. **Keywords:** article/*cocaine dependence/th [Therapy]/human/major clinical study/patient

counseling/psychosocial care/Psychotherapy/scoring system/Treatment Outcome.

RefID:2574. Sussman, S., Dent, C. W., Stacy, A. W., and Craig, S.. One-year outcomes of project towards no drug abuse. Preventive medicine 1998. 27 (4) 632-642. **Keywords:** Adolescent/Adult/alcohol consumption/article/cigarette smoking/clinical trial/controlled clinical trial/controlled study/Curriculum/*drug abuse/Female/Health Promotion/high school/human/major clinical study/Male/Motivation/*Preventive Medicine/priority journal/randomized controlled trial/social adaptation/Treatment Outcome/*Cannabis.

RefID:2584. Corby, E. A. and Curtis, Russell J.. Substance abuse risk reduction: Verbal mediational training for children by parental and nonparental models. Substance Abuse 1997. 18 (4) 145-164. **Keywords:** article/Child/*child parent relation/clinical article/clinical trial/Cognitive Therapy/controlled clinical trial/controlled study/Family/Female/human/Male/Problem Solving/randomized controlled trial/risk management/social adaptation/*substance abuse/*verbal communication.

RefID:2585. Nuttbrock, L., Rahav, M., Rivera, J., Ng-Mak, D., and Pepper, B.. Stability of psychiatric symptoms among mentally ill chemical abusers in long-term residential treatment programs. Journal of Drug Issues 1997. 27 (4) 795-806. **Keywords:** Adult/anxiety neurosis/dt [Drug Therapy]/anxiety neurosis/th [Therapy]/article/clinical trial/controlled clinical trial/controlled study/Depression/dt [Drug Therapy]/Depression/th [Therapy]/human/major clinical study/*mental disease/dt [Drug Therapy]/*mental disease/th [Therapy]/patient counseling/psychosis/dt [Drug Therapy]/psychosis/th [Therapy]/randomized controlled trial/*residential care/*substance abuse/symptomatology/*antidepressant agent/dt [Drug Therapy]/*anxiolytic agent/dt [Drug Therapy]/*psychotropic agent/dt [Drug Therapy].

RefID:2590. Carey, M. P., Maisto, S. A., Kalichman, S. C., Forsyth, A. D., Wright, E. M., and Johnson, B. T.. Enhancing motivation to reduce the risk of HIV infection for economically disadvantaged urban women. Journal of consulting and clinical psychology 1997. 65 (4) 531-541. **Keywords:** acquired immune deficiency syndrome/Adult/article/Awareness/Behavior/disease transmission/Female/high risk population/human/*Human immunodeficiency virus infection/*infection risk/major clinical study/Motivation/Public Health/socioeconomics/substance abuse/urban area/vaginitis.

RefID:2649. Mandell, W., Vlahov, D., Latkin, C. A., Carran, D., Oziemkowska, M. J., and Reedt, L.. Changes in HIV risk behaviors among counseled injecting drug users. Journal of Drug Issues 1994. 24 (3) 555-567. **Keywords:** article/*drug dependence/ep [Epidemiology]/Health Behavior/Health Education/human/*Human immunodeficiency virus infection/ep [Epidemiology]/major clinical study/Male/Risk Assessment/Self Report.

RefID:2699. Kay, Lambkin, Baker, Amanda, Lewin, Terry, and Carr, Vaughan. Acceptability of a clinician-assisted computerized psychological intervention for comorbid mental health and substance use problems: Treatment adherence data from a randomized controlled trial. Journal of medical Internet research 2011. 13 (1) 339-349. **Keywords:** *comorbidity/*drug

abuse/*Intervention/*Treatment Compliance/*Computer Assisted Therapy/Clinical Trials/Clinicians/Mental Health.

RefID:2701. Henggeler, Scott W., McCart, Michael R., Cunningham, Phillippe B., and Chapman, Jason E.. Enhancing the effectiveness of juvenile drug courts by integrating evidence-based practices. Journal of consulting and clinical psychology 2012. 80 (2) 264-275. **Keywords:** *Adjudication/*contingency management/*drug abuse/*evidence based practice/*juvenile delinquency.

RefID:2749. Begun, Audrey L., Rose, Susan J., and LeBel, Thomas P.. Intervening with women in jail around alcohol and substance abuse during preparation for community reentry. Alcoholism Treatment Quarterly 2011. 29 (4) 453-478. **Keywords:** *alcohol abuse/*drug abuse/*Intervention/*Motivational Interviewing/*rehabilitation/Alcohol Rehabilitation/Drug Rehabilitation/Human Females.

RefID:2757. Bodin, Maria and Leifman, Hakan. A randomized effectiveness trial of an adult-to-youth mentoring program in Sweden. Addiction Research & Theory 2011. 19 (5) 438-447. **Keywords:** *Drug Abuse Prevention/*Mental Health Program Evaluation/*Mentor/Psychosocial Development.

RefID:2826. Mbilinyi, Lyungai F., Neighbors, Clayton, Walker, Denise D., Roffman, Roger A., Zegree, Joan, Edleson, Jeffrey, and O'Rourke, Allison. A telephone intervention for substance-using adult male perpetrators of intimate partner violence. Research on Social Work Practice 2011. 21 (1) 43-56. **Keywords:** *drug abuse/*Intervention/*Intimate Partner Violence/*telemedicine/Perpetrators/Telephone Systems.

RefID:2838. Gollub, Erica L., Morrow, Kathleen M., Mayer, Kenneth H., Koblin, Beryl A., Peterside, Pamela Brown, Husnik, Marla J., and Metzger, David S.. Three city feasibility study of a body empowerment and HIV prevention intervention among women with drug use histories: Women FIT. Journal of Women's Health 2010. 19 (9) 1705-1713. **Keywords:** *AIDS Prevention/*Drug Usage/*HIV/*Intervention/Empowerment/History/Human Body.

RefID:2890. Burns, Michelle Nicole, Lehman, Kenneth A., Milby, Jesse B., Wallace, Dennis, and Schumacher, Joseph E.. Do PTSD symptoms and course predict continued substance use for homeless individuals in contingency management for cocaine dependence?. Behaviour research and therapy 2010. 48 (7) 588-598. **Keywords:** *Cocaine/*contingency management/*Drug Dependency/*Drug Usage/*Posttraumatic Stress Disorder/Comorbidity/Homeless.

RefID:2911. Wechsberg, Wendee M., Wu, Li Tzy, Zule, William A., Parry, Charles D., Browne, Felicia A., Luseno, Winnie K., Kline, Tracy, and Gentry, Amanda. Substance abuse, treatment needs and access among female sex workers and non-sex workers in Pretoria, South Africa. Substance abuse treatment, prevention, and policy 2009. 4 (#Issue#) #Start Page#-#End Page#. **Keywords:** *drug abuse/*Health Service Needs/*Prostitution/health care utilization/Human

Females/Treatment.

RefID:2937. Romo, Lucia, Le Strat, Yann, Aubry, Caroline, Marquez, Sonia, Houdeyer, Karine, Batel, Philippe, Ades, Jean, and Gorwood, Philip. The role of brief motivational intervention on self-efficacy and abstinence in a cohort of patients with alcohol dependence. International journal of psychiatry in medicine 2009. 39 (3) 313-323. **Keywords:** *Alcohol Rehabilitation/*Alcoholism/*Intervention/*Self Efficacy/Drug Abstinence/Drug Dependency/Motivation/Patients.

RefID:3031. Callahan, Catina. The association of criminal justice system involvement on change in high-risk behaviors among urban, crack-cocaine using women. Dissertation Abstracts International Section A: Humanities and Social Sciences 2008. 69 (2-A) 752-#End Page#. **Keywords:** *Crack Cocaine/*criminal behavior/*criminal justice/*Involvement/*Urban Environments/HIV/Human Females.

RefID:3076. Wechsberg, Wendee M., Zule, William A., Riehman, Kara S., Luseno, Wminnie K., and Lam, Wendy K. K.. African-American crack abusers and drug treatment initiation: Barriers and effects of a pretreatment intervention. Substance abuse treatment, prevention, and policy 2007. 2 (#Issue#) #Start Page#-#End Page#. **Keywords:** *Blacks/*Crack Cocaine/*drug abuse/*Drug Rehabilitation/*Treatment Barriers/Health Care Seeking Behavior/Intervention.

RefID:3087. Sharp, William Garry. Help-seeking and mental health education: An evaluation of a classroom-based strategy to modify help-seeking for mental health problems. Dissertation Abstracts International: Section B: The Sciences and Engineering 2007. 68 (6-B) 4143-#End Page#. **Keywords:** *Health Education/*Help Seeking Behavior/*Mental Disorders/*Mental Health/Classrooms.

RefID:3096. Gauthier-Faille, Gail. Outpatient treatment for substance dependence: Using empirical findings about retention and substance use outcomes to shape treatment services. Dissertation Abstracts International: Section B: The Sciences and Engineering 2007. 68 (3-B) 1925-#End Page#. **Keywords:** *Drug Abuse Prevention/*Employment Status/*health/*Outpatients/Retention.

RefID:3116. Ghitza, Udi E., Epstein, David H., Schmittner, John, Vahabzadeh, Massoud, Lin, Jia Ling, and Preston, Kenzie L.. Randomized trial of prize-based reinforcement density for simultaneous abstinence from cocaine and heroin. Journal of consulting and clinical psychology 2007. 75 (5) 765-774. **Keywords:** *Cocaine/*Drug Abstinence/*Heroin/*Methadone Maintenance/*reinforcement/Contingency Management/Drug Rehabilitation.

RefID:3227. Marsden, John, Stillwell, Garry, Barlow, Helen, Taylor, Colin, Boys, Annabel, and Hunt, Neil. An evaluation of a brief intervention model for use with young non-injecting stimulant users. Drugs: Education, Prevention & Policy 2005. 12 (Suppl1) 90-93. **Keywords:** *drug abuse/*Intervention/*Models/*Motivation/*Treatment/Cocaine/Crack Cocaine/Methylenedioxymethamphetamine.

RefID:3235. Gaiton, Lana R.. Investigation of therapeutic alliance in a treatment study with substance-abusing women with PTSD. Dissertation Abstracts International: Section B: The Sciences and Engineering 2005. 65 (9-B) 4828-#End Page#. **Keywords:** *Cognitive Behavior Therapy/*drug abuse/*Posttraumatic Stress Disorder/*Therapeutic Alliance/*Treatment Outcomes.

RefID:3339. Campbell, Aimee N. C., Fisher, Douglass S., Picciano, Joseph F., Orlando, Matthew J., Stephens, Robert S., and Roffman, Roger A.. Marketing Effectiveness in Reaching the Nontreatment-Seeking Marijuana Smoker. Journal of Social Work Practice in the Addictions 2004. 4 (1) 39-59. **Keywords:** *Marijuana Usage/*Marketing/*Social Marketing.

RefID:3425. Siqueland, Lynne, Crits-Christoph, Paul, Gallop, Robert, Barber, Jacques P., Griffin, Margaret L., Thase, Michael E., Daley, Denis, Frank, Arlene, Gastfriend, David R., Blaine, Jack, Connolly, Mary Beth, and Gladis, Madeline. Retention in psychosocial treatment of cocaine dependence: Predictors and impact on outcome. The American Journal on Addictions 2002. 11 (1) 24-40. **Keywords:** *Client Characteristics/*Cocaine/*Drug Rehabilitation/*Treatment Dropouts/*Treatment Outcomes/Brief Psychotherapy/Cognitive Therapy/Comorbidity/Counseling/Drug Dependency/Psychiatric Symptoms/Severity (Disorders)/treatment duration.

RefID:3492. Nwakeze, Peter C., Magura, Stephen, Rosenblum, Andrew, and Joseph, Herman. Service outcomes of peer consumer advocacy for soup kitchens guests. Journal of Social Service Research 2000. 27 (2) 19-38. **Keywords:** *Case Management/*Models/*Program Evaluation/*Social Services.

RefID:3513. Longshore, Douglas and Grills, Cheryl. Motivating illegal drug use recovery: Evidence for a culturally congruent intervention. Journal of Black Psychology 2000. 26 (3) 288-301. **Keywords:** *Blacks/*drug abuse/*Drug Rehabilitation/*Sociocultural Factors.

RefID:3557. Aubrey, Lauren Lawendowski. Motivational interviewing with adolescents presenting for outpatient substance abuse treatment. Dissertation Abstracts International: Section B: The Sciences and Engineering 1998. 59 (3-B) 1357-#End Page#. **Keywords:** *Brief Psychotherapy/*drug abuse/*Drug Rehabilitation/*Interviewing/*Motivational Interviewing.

RefID:3830. Brooks, A. C., Ryder, D., Carise, D., and Kirby, K. C.. Feasibility and effectiveness of computer-based therapy in community treatment. Journal of substance abuse treatment 2010. 39 (3) 227-235. **Keywords:** Multifile Reviews/adaptation/Psychological/Adult/Cocaine-Related Disorders/rh [Rehabilitation]/Evidence-Based Medicine/mt [Methods]/Feasibility Studies/Female/Health Knowledge/Attitudes/Practice/Humans/Internet/Male/Middle Aged/Pilot Projects/Referral and Consultation/sn [Statistics & Numerical Data]/Reinforcement (Psychology)/Substance-Related Disorders/rh [Rehabilitation]/therapy/Computer-Assisted/mt [Methods].

RefID:4383. Schinke, S. P., Orlandi, M. A., Botvin, G. J., and Gilchrist, L. D.. Preventing substance abuse among American-Indian adolescents: A bicultural competence skills approach. Journal of consulting and clinical psychology 1988. 35 (#Issue#) 87-90. **Keywords:** Central.

RefID:4389. Johnson, R., Fudala, P., and Krieter, N.. Effects of daily versus alternate-day dosing of buprenorphine in heroin-dependent volunteers. NIDA Research Monograph Series 1988. 90 (#Issue#) 332-333. **Keywords:** Central/Drug Dependence /Dt [Drug Therapy]/Adult/drug intermittent therapy/major clinical study/human/conference paper/diamorphine/Buprenorphine /Dt [Drug Therapy].

RefID:4731. Soskin, R. A.. The use of LSD in time-limited psychotherapy. The Journal of nervous and mental disease 1973. 157 (6) 410-419. **Keywords:** Central/Analysis of Variance/Clinical Trials as Topic/Conversion Disorder [therapy]/Defense Mechanisms/Evaluation Studies as Topic/Follow-Up Studies/Lysergic Acid Diethylamide [therapeutic use]/MMPI/Personality Assessment/Placebos/Psychiatric Status Rating Scales/Psychophysiologic Disorders [drug therapy] [therapy]/Psychotherapy/Time Factors/Adult/Humans/Male.

RefID:5152. Gottheil, E., Weinstein, S. P., Sterling, R. C., Lundy, A., and Serota, R. D.. A randomized controlled study of the effectiveness of intensive outpatient treatment for cocaine dependence. Psychiatric services (Washington, D.C.) 1998. 49 (6) 782-787. **Keywords:** Central/Ambulatory Care/Cocaine-Related Disorders [rehabilitation]/Follow-Up Studies/Philadelphia/Psychotherapy/Psychotherapy,Group/Substance Abuse Treatment Centers/Treatment Outcome/Urban Population/Adult/Female/Humans/Male.

RefID:5247. Kirby, K. C., Marlowe, D. B., Festinger, D. S., Lamb, R. J., and Platt, J. J.. Schedule of voucher delivery influences initiation of cocaine abstinence. Journal of consulting and clinical psychology 1998. 66 (5) 761-767. **Keywords:** Central/Analysis of Variance/Behavior Therapy [methods] [standards]/Chi-Square Distribution/Cocaine-Related Disorders [therapy]/Longitudinal Studies/Reinforcement Schedule/Substance Abuse Detection [psychology]/Survival Analysis/Token Economy/Treatment Outcome/Adult/Female/Humans/Male/Middle Aged.

RefID:5303. Grenyer, B. F., Solowij, N., and Peters, R.. Brief versus intensive psychotherapy for cannabis dependence. NIDA research monograph 1997. 174 (#Issue#) 108-#End Page#. **Keywords:** Central.

RefID:5306. Chan, M., Sorense, J. L., Guydish, J., Tajima, B., and Acampora, A.. Client satisfaction with drug abuse day treatment versus residential care. Journal of Drug Issues 1997. 27 (2) 367-377. **Keywords:** Central/Drug Dependence /Day Hospital /Home Care /Patient Satisfaction /Disease Severity /Treatment Outcome /Mental Health Service /Human /Article.

RefID:5570. Lam, J. A., Jekel, J. F., Thompson, K. S., Leaf, P. J., Hartwell, S. W., and Florio, L.. Assessing the value of a short-term residential drug treatment program for homeless men. Journal of addictive diseases 1995. 14 (4) 21-39. **Keywords:** Central/Cocaine/Connecticut/Day

Care/Follow-Up Studies/Homeless Persons [psychology]/Length of Stay/Public Housing/Substance Abuse Treatment Centers/Substance-Related Disorders [psychology] [rehabilitation]/Treatment Outcome/Adult/Humans/Male.

RefID:5746. Oliveto, A., Sevarino, K., Feingold, A., Gonsai, K., McCance-Katz, E., Stein, S., and Kosten, T. R.. Effectiveness of LAAM dose and contingency management procedures for facilitating drug abstinence in opioid dependent cocaine abusers. Drug and alcohol dependence 2001. 63 Suppl 1 (#Issue#) 117-#End Page#. **Keywords:** Central.

RefID:6027. Kwiatkowski, C. F., Booth, R. E., and Lloyd, L. V.. The effects of offering free treatment to street-recruited opioid injectors. Addiction (Abingdon, England) 2000. 95 (5) 697-704. **Keywords:** Central/Adolescent/Methadone [economics] [therapeutic use]/Narcotics [economics] [therapeutic use]/Opioid-Related Disorders [economics] [rehabilitation]/Patient Compliance [psychology]/Street Drugs/Substance Abuse,Intravenous [economics] [rehabilitation]/Adult/Aged/Female/Humans/Male/Middle Aged.

RefID:6152. Rosenthal, R. N., Muran, J. C., Pinsker, H., Hellerstein, D., and Winston, A.. Interpersonal change in brief supportive psychotherapy. Journal of Psychotherapy Practice & Research 1999. 8 (1) 55-63. **Keywords:** Central/Adult/case report/Female/human/Interpersonal Relations/Male/Middle Age/Personality Disorders/th [Therapy]/Psychotherapy.

RefID:6193. Hoffman, J. A., Klein, H., Crosby, H., and Clark, D. C.. Project neighborhoods in action: an HIV-related intervention project targeting drug abusers in Washington, DC. Journal of urban health : bulletin of the New York Academy of Medicine 1999. 76 (4) 419-434. **Keywords:** Central/Community-Institutional Relations/District of Columbia/HIV Infections [prevention & control]/Intervention Studies/Risk-Taking/Substance Abuse Treatment Centers/Substance Abuse,Intravenous [prevention & control]/Adult/Female/Humans/Male/Middle Aged.

RefID:7000. Smelson, David, Kalman, David, Losonczy, Miklos, Kline, Anna, Sambamoorthi, Usha, Hill, Lauren, Castles-Fonseca, Kathy, and Ziedonis, Douglas. A Brief Treatment Engagement Intervention for Individuals with Co-occurring Mental Illness and Substance Use Disorders: Results of a Randomized Clinical Trial. Community mental health journal 2012. 48 (2) 127-132. **Keywords:** CINAHL.

RefID:7015. Otiashvili, D., Kirtadze, I., O'Grady, K. E., and Jones, H. E.. Drug use and HIV risk outcomes in opioid-injecting men in the Republic of Georgia: Behavioral treatment+naltrexone compared to usual care. Drug & Alcohol Dependence 1-1-2012. 120 (1-3) 14-21. **Keywords:** CINAHL.

RefID:7030. Stanger, C., Ryan, S. R., Fu, H., and Budney, A. J.. Parent training plus contingency management for substance abusing families: A Complier Average Causal Effects (CACE) analysis. Drug & Alcohol Dependence 1-11-2011. 118 (2-3) 119-126. **Keywords:** CINAHL/Child Behavior/Children of Impaired Parents -- Psychosocial Factors/Parenting/Parents -- Education/Substance Use Disorders -- Psychosocial Factors/Adult/Child/Child Behavior

Disorders -- Psychosocial Factors/Child,Preschool/Female/human/Male/Parent-Child Relations/Clinical Trials/Child Behavior Checklist.

RefID:7119. McCambridge, J., Day, M., Thomas, B. A., and Strang, J.. Fidelity to Motivational Interviewing and subsequent cannabis cessation among adolescents. Addictive behaviors 2011. 36 (7) 749-754. **Keywords:** CINAHL/Diagnosis,Psychosocial/Motivation/Substance Use Disorders -- Psychosocial Factors/adolescence/England/Female/human/Male/Outcomes (Health Care)/Substance Use Disorders -- Rehabilitation/Treatment Outcomes/Young Adult.

RefID:7205. Mbilinyi, L. F., Neighbors, C., Walker, D. D., Roffman, R. A., Zegree, J., Edleson, J., and O'Rourke, A.. A telephone intervention for substance-using adult male perpetrators of intimate partner violence. Research on Social Work Practice 2011. 21 (1) 43-56. **Keywords:** CINAHL/Intimate Partner Violence -- Therapy/Public Offenders/Men/Telehealth/human/Funding Source/Motivational Interviewing/Substance Abusers/help seeking behavior/Male/Random Assignment/control group/Comparative Studies/Feedback/Treatment Outcomes/Reference Values/Psychotherapy,Brief/Randomized Controlled Trials/Patient Compliance/Repeated Measures/Descriptive Statistics/Scales/Interview Guides/chi square test/Correlational Studies/Poisson Distribution/Prospective Studies/Wilcoxon Rank Sum Test/Analysis of Variance/Social Work Practice.

RefID:7236. Fang, L., Schinke, S. P., and Cole, K. C.. Preventing Substance Use Among Early Asian–American Adolescent Girls: Initial Evaluation of a Web-based, Mother–Daughter Program. Journal of Adolescent Health 2010. 47 (5) 529-532. **Keywords:** CINAHL/Substance Abuse -- Prevention and Control -- In Adolescence/Asians -- In Adolescence -- United States/Mother-Child Relations/Internet/United States/human/Daughters/Mothers/Female/adolescence/Child/Adult/convenience sample/Random Assignment/Family Theory/conceptual framework/Pretest-Posttest Design/Family Centered Care/Descriptive Statistics/Demography/Interpersonal Relations/Depression/Communication/Parenting/T-Tests/chi square test/Poisson Distribution/Scales/Summated Rating Scaling/Alcohol Drinking/Smoking/Cannabis/Street Drugs.

RefID:7287. Freudenberg, N., Ramaswamy, M., Daniels, J., Crum, M., Ompad, D. C., and Vlahov, D.. Reducing Drug Use, Human Immunodeficiency Virus Risk, and Recidivism Among Young Men Leaving Jail: Evaluation of the REAL MEN Re-entry Program. Journal of Adolescent Health 2010. 47 (5) 448-455. **Keywords:** CINAHL/Repeat Offenders -- In Adolescence/Substance Abuse -- Prevention and Control -- In Adolescence/HIV Infections -- Prevention and Control -- In Adolescence/Risk Taking Behavior -- Prevention and Control -- In Adolescence/Juvenile Offenders/human/Blacks/Hispanics/Male/adolescence/New York/Descriptive Statistics/convenience sample/Random Assignment/Interviews/Logistic Regression/Regression/employment status/Educational Status/sexuality/Multicenter Studies/exploratory research/Demography/Health Knowledge/Attitude/T-Tests/Data Analysis Software/Organizations,Nonprofit/outcome assessment.

RefID:7457. Kay-Lambkin, F. J., Baker, A. L., Lewin, T. J., and Carr, V. J.. Computer-based psychological treatment for comorbid depression and problematic alcohol and/or cannabis use: a randomized controlled trial of clinical efficacy. Addiction (Abingdon, England) 2009. 104 (3) 378-388. **Keywords:** CINAHL/Depression -- Therapy/Psychotherapy -- Methods/Substance Use Disorders -- Therapy/Therapy,Computer Assisted/adolescence/Adult/Analysis of Variance/Audiorecording/chi square test/Cognitive Therapy/Combined Modality Therapy/Comorbidity/Confidence Intervals/Data Analysis Software/Descriptive Statistics/Evaluation Research/Female/Funding Source/Harm Reduction/human/Interview Guides/Logistic Regression/Male/Middle Age/Motivational Interviewing/New South Wales/Odds Ratio/Pretest-Posttest Design/Prospective Studies/Psychological Tests/Psychotherapy,Brief/Random Assignment/Randomized Controlled Trials/Repeated Measures/structured interview/Treatment Outcomes.

RefID:7502. Weiss, R. D., Griffin, M. L., Jaffee, W. B., Bender, R. E., Graff, F. S., Gallop, R. J., and Fitzmaurice, G. M.. A "community-friendly" version of integrated group therapy for patients with bipolar disorder and substance dependence: a randomized controlled trial. Drug & Alcohol Dependence 1-10-2009. 104 (3) 212-219. **Keywords:** CINAHL/Bipolar Disorder -- Therapy/Cognitive Therapy -- Methods/Community Mental Health Services -- Methods/Psychotherapy,Group -- Methods/Substance Use Disorders -- Therapy/Adult/Antidepressive Agents -- Therapeutic Use/Antimanic Agents -- Therapeutic Use/Bipolar Disorder -- Complications/Randomized Controlled Trials/Counseling/Diagnosis,Dual (Psychiatry)/Female/Male/Medication Compliance/Substance Use Disorders -- Complications/Time Factors/human.

RefID:7505. Stanger, C., Budney, A. J., Kamon, J. L., and Thostensen, J.. A randomized trial of contingency management for adolescent marijuana abuse and dependence. Drug & Alcohol Dependence 1-12-2009. 105 (3) 240-247. **Keywords:** CINAHL/Adolescent Behavior/Behavior,Addictive -- Therapy/Cognitive Therapy -- Methods/Family Therapy -- Methods/Substance Use Disorders -- Therapy/adolescence/Adult/Child/Female/human/Male/Patient Compliance/Treatment Outcomes/Clinical Trials.

RefID:7526. Kidorf, M., King, V. L., Neufeld, K., Peirce, J., Kolodner, K., and Brooner, R. K.. Improving substance abuse treatment enrollment in community syringe exchangers. Addiction (Abingdon, England) 2009. 104 (5) 786-795. **Keywords:** CINAHL/Intravenous Drug Users -- Psychosocial Factors/Motivation/Needle Exchange Programs/Referral and Consultation/Substance Abuse,Intravenous -- Therapy/Adult/Analysis of Variance/chi square test/Clinical Trials/Combined Modality Therapy/Confidence Intervals/Contingency Management/Descriptive Statistics/Economics/Evaluation Research/Female/Funding Source/Harm Reduction/Interview Guides/Logistic Regression/Male/Odds Ratio/Psychological Tests/Semi-Structured Interview/T-Tests/Treatment Outcomes/human.

RefID:7544. Baker, A., Turner, A., Kay-Lambkin, F. J., and Lewin, T. J.. The long and the short of

treatments for alcohol or cannabis misuse among people with severe mental disorders. Addictive behaviors 2009. 34 (10) 852-858. **Keywords:** CINAHL/Alcohol Drinking/Alcohol-Related Disorders -- Therapy/Mental Disorders -- Therapy/Substance Use Disorders -- Therapy/Adult/Alcohol Drinking -- Epidemiology/Alcohol-Related Disorders -- Epidemiology/Cognitive Therapy/Comorbidity/Diagnosis,Dual (Psychiatry)/Female/human/Male/Mental Disorders -- Epidemiology/Middle Age/Motivation/New South Wales/Substance Use Disorders -- Epidemiology/Treatment Outcomes/Young Adult.

RefID:7607. Fals-Stewart, W. and Lam, W. K. K.. Brief behavioral couples therapy for drug abuse: a randomized clinical trial examining clinical efficacy and cost-effectiveness. Families, Systems & Health: The Journal of Collaborative Family HealthCare 2008. 26 (4) 377-392. **Keywords:** CINAHL/cost benefit analysis/Couples Counseling/Psychotherapy,Brief/Substance Abuse -- Therapy/Treatment Outcomes -- Evaluation/Adaptation,Psychological/Adult/Audiorecording/Randomized Controlled Trials/Comparative Studies/Couples Counseling -- Economics/Descriptive Statistics/Dyadic Adjustment Scale/Female/Interview Guides/Interviews/Male/Prospective Studies/psychoeducation/Psychological Tests/Psychotherapy,Brief -- Economics/Psychotherapy,Group/Questionnaires/human.

RefID:7616. Mooney, M. E., Herin, D. V., Schmitz, J. M., Moukaddam, N., Green, C. E., and Grabowski, J.. Effects of oral methamphetamine on cocaine use: A randomized, double-blind, placebo-controlled trial. Drug & Alcohol Dependence 1-4-2009. 101 (1-2) 34-41. **Keywords:** CINAHL/Central Nervous System Stimulants -- Therapeutic Use/Methamphetamine -- Therapeutic Use/Substance Use Disorders -- Rehabilitation/adolescence/Adult/Affect -- Drug Effects/Blood Pressure -- Drug Effects/Body Weight -- Drug Effects/Central Nervous System Stimulants -- Administration and Dosage/Delayed-Action Preparations/Double-Blind Studies/Female/Heart Rate -- Drug Effects/Male/Methamphetamine -- Administration and Dosage/Middle Age/Riboflavin -- Diagnostic Use/Substance Abuse Detection/Substance Use Disorders -- Psychosocial Factors/Treatment Outcomes/human.

RefID:7651. Gordon, M. S., Kinlock, T. W., Schwartz, R. P., and O'Grady, K. E.. A randomized clinical trial of methadone maintenance for prisoners: findings at 6 months post-release. Addiction (Abingdon, England) 2008. 103 (8) 1333-1342. **Keywords:** CINAHL/Counseling/Methadone -- Therapeutic Use/Prisoners/Substance Dependence -- Drug Therapy/Adult/chi square test/Randomized Controlled Trials/Combined Modality Therapy/Community Mental Health Services/Correctional Health Services/Descriptive Statistics/Drug Rehabilitation Programs/Funding Source/Interviews/Logistic Regression/Male/Maryland/Pretest-Posttest Design/Prospective Studies/psychoeducation/Psychological Tests/Record Review/Self Report/Severity of Illness Indices/Substance Abuse Detection/Treatment Outcomes/Urinalysis/human.

RefID:7718. Morgenstern, J., Neighbors, C. J., Kuerbis, A., Riordan, A., Blanchard, K. A., McVeigh, K. H., Morgan, T. J., and McCrady, B.. Improving 24-month abstinence and employment

outcomes for substance-dependent women receiving temporary assistance for needy families with intensive case management. American journal of public health 2009. 99 (2) 328-333. **Keywords:** CINAHL/Case Management -- Utilization/Employment/Public Assistance/Substance Abuse -- Therapy/Women/Comparative Studies/Confidence Intervals/control group/Drug Rehabilitation Programs -- Methods/Female/Funding Source/Interviews/Mandatory Testing/Models,Statistical/New Jersey/Odds Ratio/Patient Compliance/Program Evaluation/Prospective Studies/Random Assignment/Self Report/statistical significance/Time Factors/Treatment Outcomes/human.

RefID:7756. Werch, C. E., Bian, H., Moore, M. J., Ames, S. C., DiClemente, C. C., Thombs, D., and Pokorny, S. B.. Brief multiple behavior health interventions for older adolescents. American Journal of Health Promotion 2008. 23 (2) 92-96. **Keywords:** CINAHL/Career Planning and Development/Colleges and Universities/Crisis Intervention -- In Adolescence/Health Behavior/Models,Theoretical/Success/adolescence/Alcohol Drinking/Analysis of Variance/Cannabis/Clinical Assessment Tools/Exercise/Female/Florida/Funding Source/Health Promotion/Male/multivariate analysis of variance/nutrition/outcome assessment/Surveys/human.

RefID:7761. Barry, D., Weinstock, J., and Petry, N. M.. Ethnic differences in HIV risk behaviors among methadone-maintained women receiving contingency management for cocaine use disorders. Drug & Alcohol Dependence 1-11-2008. 98 (1-2) 144-153. **Keywords:** CINAHL/Contingency Management/Ethnic Groups -- Psychosocial Factors/HIV Infections -- Risk Factors/HIV Infections -- Transmission/Methadone -- Therapeutic Use/Risk Taking Behavior/Substance Use Disorders -- Rehabilitation/Adult/Blacks -- Psychosocial Factors/Clinical Trials/Female/Hispanics -- Psychosocial Factors/HIV Infections -- Ethnology/HIV Infections -- Prevention and Control/Middle Age/Motivation/Prospective Studies/Reward/Substance Use Disorders -- Ethnology/Treatment Outcomes/Unsafe Sex/Whites -- Psychosocial Factors/human.

RefID:7855. Wechsberg, W. M., Luseno, W. K., Karg, R. S., Young, S., Rodman, N., Myers, B., and Parry, C. D.. Alcohol, cannabis, and methamphetamine use and other risk behaviours among Black and Coloured South African women: a small randomized trial in the Western Cape. International Journal of Drug Policy 2008. 19 (2) 130-139. **Keywords:** CINAHL/HIV Infections -- Prevention and Control/Risk Taking Behavior/Sexuality -- Ethnology/Substance Use Disorders -- Epidemiology/Unsafe Sex/adolescence/Adult/Alcohol Drinking -- Epidemiology/Alcohol Drinking -- Ethnology/Blacks -- Statistics and Numerical Data/Clinical Trials/Condoms -- Utilization/Female/Focus Groups/HIV Infections -- Transmission/Medical Practice,Evidence-Based -- Methods/Methamphetamine -- Adverse Effects/Middle Age/Pilot Studies/Sexual Partners/South Africa -- Ethnology/Substance Use Disorders -- Ethnology/Unsafe Sex -- Ethnology/human.

RefID:7857. Werch, C. E., Bian, H., Moore, M. J., Ames, S., DiClemente, C. C., and Weiler, R. M.. Brief multiple behavior interventions in a college student health care clinic. Journal of Adolescent Health 2007. 41 (6) 577-585. **Keywords:** CINAHL/Health Behavior/Health Promotion

-- Methods/Physical Fitness/Risk Taking Behavior/Student Health Services/adolescence/Adult/Analysis of Variance/Behavior/Body Image/Clinical Trials/Colleges and Universities/Contracts/Counseling/Diet/Exercise/Female/Male/Program Evaluation/Psychotherapy,Brief/Referral and Consultation/Scales/Sleep/Southeastern United States/Substance Use Disorders -- Prevention and Control/Treatment Outcomes/human.

RefID:7874. Festinger, D. S., Marlowe, D. B., Dugosh, K. L., Croft, J. R., and Arabia, P. L.. Higher magnitude cash payments improve research follow-up rates without increasing drug use or perceived coercion. Drug & Alcohol Dependence 1-7-2008. 96 (1-2) 128-135. **Keywords:** CINAHL/Coercion/Consumer Participation/Research,Mental Health -- Economics/Research,Mental Health -- Ethical Issues/Substance Abuse -- Trends/Adult/Analysis of Variance/Attitude Measures/chi square test/Chromatography,Gas/Coefficient Alpha/Descriptive Statistics/Female/Interviews/Logistic Regression/Male/Middle Age/Motivation/Outpatients/Pennsylvania/Pretest-Posttest Design/Psychological Tests/Questionnaires/Random Assignment/Self Report/Severity of Illness Indices/Mass Spectrometry/Summated Rating Scaling/Treatment Outcomes/Urban Areas/Urinalysis/human.

RefID:8010. Litt, M. D., Kadden, R. M., Kabela-Cormier, E., and Petry, N. M.. Coping skills training and contingency management treatments for marijuana dependence: exploring mechanisms of behavior change. Addiction (Abingdon, England) 2008. 103 (4) 638-648. **Keywords:** CINAHL/Behavioral Changes -- Evaluation/Cognitive Therapy -- Methods/Contingency Management/Coping -- Education/Substance Dependence -- Psychosocial Factors/Substance Dependence -- Therapy/Academic Medical Centers/Adult/Attitude to Change/Cannabis/Case Management/Clinical Trials/Coefficient Alpha/Combined Modality Therapy/Coping Strategies Questionnaire/Descriptive Statistics/Funding Source/Goodness of Fit Chi Square Test/Interview Guides/Male/Motivation/One-Way Analysis of Variance/Outpatients/Path Analysis/Patient Compliance/Pretest-Posttest Design/Prospective Studies/Psychological Tests/Regression/Self-Efficacy/structural equation modeling/Summated Rating Scaling/Test-Retest Reliability/Treatment Outcomes/Urinalysis/human.

RefID:8038. McGarry, J., McNicholas, F., Buckley, H., Kelly, B. D., Atkin, L., and Ross, N.. The clinical effectiveness of a brief consultation and advisory approach compared to treatment as usual in child and adolescent mental health services. Clinical Child Psychology & Psychiatry 2008. 13 (3) 365-376. **Keywords:** CINAHL/Mental Disorders -- Therapy -- In Adolescence/Mental Disorders -- Therapy -- In Infancy and Childhood/Mental Health Services -- In Adolescence/Mental Health Services -- In Infancy and Childhood/Referral and Consultation -- Methods/adolescence/Anxiety -- Therapy/chi square test/Child/Child Behavior Disorders -- Therapy/Child,Preschool/Clinical Trials/Depression -- Therapy/Descriptive Statistics/Evaluation Research/Female/Funding Source/Male/Mann-Whitney U Test/Models,Theoretical/Patient Centered Care/Pretest-Posttest Design/Prospective Studies/Psychological Tests/Questionnaires/Repeated Measures/Scales/T-Tests/Treatment Outcomes/human.

RefID:8176. Corrigan, J. D. and Bogner, J.. Interventions to promote retention in substance

abuse treatment. Brain Injury 2007. 21 (4) 343-356. **Keywords:** CINAHL/Brain Injuries -- Psychosocial Factors/Gift Giving/Health Services Accessibility/Motivation/Patient Compliance/Substance Use Disorders -- Rehabilitation/Adult/Aged/Brain Injuries -- Complications/Clinical Trials/Female/Male/Middle Age/Professional-Patient Relations/Socioeconomic Factors/Substance Use Disorders -- Complications/Substance Use Disorders -- Psychosocial Factors/human.

RefID:8184. Stotts, A. L., Potts, G. F., Ingersoll, G., George, M. R., and Martin, L. E.. Preliminary feasibility and efficacy of a brief motivational intervention with psychophysiological feedback for cocaine abuse. Substance Abuse 2006. 27 (4) 9-20. **Keywords:** CINAHL/Biofeedback -- Methods/Cocaine -- Adverse Effects/Motivational Interviewing -- Methods/Substance Use Disorders -- Therapy/adolescence/Adult/Analysis of Variance/chi square test/Descriptive Statistics/Electroencephalography -- Methods/Male/Middle Age/Patient Attitudes/Patient Satisfaction/Pilot Studies/Pretest-Posttest Control Group Design/Psychological Tests/Questionnaires/Random Assignment/Repeated Measures/Scales/Self Report/Sensory Stimulation/Severity of Illness Indices/Substance Abuse Detection/Summated Rating Scaling/Surveys/Texas/Treatment Outcomes/Univariate Statistics/human.

RefID:8190. Coviello, D. M., Zanis, D. A., Wesnoski, S. A., and Alterman, A. I.. The effectiveness of outreach case management in re-enrolling discharged methadone patients. Drug & Alcohol Dependence 15-10-2006. 85 (1) 56-65. **Keywords:** CINAHL/Case Management -- Methods/Methadone -- Therapeutic Use/Substance Abusers/Substance Dependence -- Drug Therapy/Adult/chi square test/Confidence Intervals/Experimental Studies/Female/Funding Source/HIV Infections/Logistic Regression/Male/Middle Age/Odds Ratio/Pennsylvania/post hoc analysis/Research Instruments/Risk Taking Behavior/Self Report/Substance Abuse Detection/T-Tests/human.

RefID:8197. Sindelar, J., Elbel, B., and Petry, N. M.. What do we get for our money? Cost-effectiveness of adding contingency management. Addiction (Abingdon, England) 2007. 102 (2) 309-316. **Keywords:** CINAHL/Behavior Modification -- Economics/Cocaine/Substance Abuse -- Therapy/chi square test/Comparative Studies/cost benefit analysis/Funding Source/Outpatients/Random Assignment/Reward/human.

RefID:8214. Ledgerwood, D. M. and Petry, N. M.. Does contingency management affect motivation to change substance use?. Drug & Alcohol Dependence 9-6-2006. 83 (1) 65-72. **Keywords:** CINAHL/Behavior Therapy/Motivation/Substance Dependence -- Therapy/Adult/analysis of covariance/chi square test/Confidence Intervals/Female/Internal Consistency/Interviews/Logistic Regression/Male/Middle Age/Odds Ratio/Outpatients/Pretest-Posttest Design/Random Assignment/Research Instruments/Self Report/Substance Abuse Detection/Substance Abusers/Transtheoretical Stages of Change Model/human.

RefID:8216. Strathdee, S. A., Ricketts, E. P., Huettner, S., Cornelius, L., Bishai, D., Havens, J. R., Beilenson, P., Rapp, C., Lloyd, J. J., and Latkin, C. A.. Facilitating entry into drug treatment among injection drug users referred from a needle exchange program: results from a

community-based behavioral intervention trial. Drug & Alcohol Dependence 27-7-2006. 83 (3) 225-232. **Keywords:** CINAHL/Case Management -- Evaluation/help seeking behavior/Intravenous Drug Users/Substance Abuse,Intravenous -- Drug Therapy/Adult/Case Managers/Center for Epidemiological Studies Depression Scale/chi square test/Clinical Trials/Confidence Intervals/Data Analysis Software/Experimental Studies/Female/Funding Source/Interview Guides/Interviews/Logistic Regression/Male/Maryland/Middle Age/Odds Ratio/Random Assignment/Scales/Transtheoretical Stages of Change Model/Wilcoxon Rank Sum Test/human.

RefID:8235. Morgenstern, J., Blanchard, K. A., McCrady, B. S., McVeigh, K. H., Morgan, T. J., and Pandina, R. J.. Research and practice. Effectiveness of intensive case management for substance-dependent women receiving temporary assistance for needy families. American journal of public health 2006. 96 (11) 2016-2023. **Keywords:** CINAHL/Case Management -- Administration/Economic and Social Security -- Utilization/Mothers -- Psychosocial Factors/Patient Compliance/Substance Use Disorders -- Prevention and Control/Adult/Blacks/Chronic Disease/Clinical Assessment Tools/Clinical Trials/Continuity of Patient Care/Critical Care/Data Analysis Software/Female/Funding Source/Hispanics/New Jersey/patient care/Program Evaluation/Random Assignment/Referral and Consultation/Social Welfare/Social Work/Substance Use Disorders -- Economics/T-Tests/United States/human.

RefID:8420. Naar-King, S., Wright, K., Parsons, J. T., Frey, M., Templin, T., Lam, P., and Murphy, D.. Healthy choices: motivational enhancement therapy for health risk behaviors in HIV-positive youth. AIDS Education & Prevention 2006. 18 (1) 1-11. **Keywords:** CINAHL/Cognitive Therapy/HIV Infections -- Prevention and Control/Motivation/Risk Taking Behavior/adolescence/Adult/Attitude to Health/Child/Clinical Trials/effect size/Female/Interviews/Male/One-Tailed Test/P-Value/Paired T-Tests/Pilot Studies/Pretest-Posttest Design/Questionnaires/Research Subject Retention/Sample Size/Substance Use Disorders/United States/Viral Load/Funding Source/human.

RefID:8458. Shoptaw, S., Reback, C. J., Peck, J. A., Yang, X., Rotheram-Fuller, E., Larkins, S., Veniegas, R. C., Freese, T. E., and Hucks-Ortiz, C.. Behavioral treatment approaches for methamphetamine dependence and HIV-related sexual risk behaviors among urban gay and bisexual men. Drug & Alcohol Dependence 9-5-2005. 78 (2) 125-134. **Keywords:** CINAHL/Behavior Therapy/Bisexuals/HIV Infections -- Prevention and Control/Homosexuals,Male/Methamphetamine/Risk Taking Behavior -- Prevention and Control/Substance Dependence -- Therapy/Adult/California/chi square test/Clinical Trials/Descriptive Statistics/Funding Source/Interview Guides/Interviews/McNemar's Test/Poisson Distribution/post hoc analysis/Questionnaires/Random Assignment/Self Report/Univariate Statistics/Urban Areas/Urinalysis/human.

RefID:8460. Baker, A., Lee, N. K., Claire, M., Lewin, T. J., Grant, T., Pohlman, S., Saunders, J. B., Kay-Lambkin, F., Constable, P., Jenner, L., and Carr, V. J.. Brief cognitive behavioural interventions for regular amphetamine users: a step in the right direction. Addiction (Abingdon, England) 2005. 100 (3) 367-378. **Keywords:** CINAHL/Amphetamines/Cognitive

Therapy/Substance Abuse -- Therapy/Adult/analysis of covariance/Brief Symptom Inventory/chi square test/Clinical Trials/Confidence Intervals/Counseling/Data Analysis Software/Descriptive Statistics/Female/Funding Source/Interview Guides/Interviews/Logistic Regression/Male/New South Wales/Odds Ratio/Psychological Tests/Queensland/Random Assignment/Repeated Measures/Scales/Urinalysis/human.

RefID:8472. Rosenblum, A., Magura, S., Kayman, D. J., and Fong, C.. Motivationally enhanced group counseling for substance users in a soup kitchen: a randomized clinical trial. Drug & Alcohol Dependence 1-10-2005. 80 (1) 91-103. **Keywords:** CINAHL/Cognitive Therapy/Homeless Persons/Motivational Interviewing/Psychotherapy,Group/Substance Abuse -- Therapy/Adult/analysis of covariance/Analysis of Variance/Bivariate Statistics/Center for Epidemiological Studies Depression Scale/chi square test/Randomized Controlled Trials/Confidence Intervals/Depression/Descriptive Statistics/Female/Interviews/Kappa Statistic/Logistic Regression/Male/Middle Age/Multivariate Analysis/New York/Odds Ratio/Psychotherapy,Group -- Methods/Random Assignment/Research Instruments/Scales/Self Report/Urinalysis/Funding Source/human.

RefID:8566. Gil, A. G., Wagner, E. F., and Tubman, J. G.. Culturally sensitive substance abuse intervention for Hispanic and African American adolescents: empirical examples from the Alcohol Treatment Targeting Adolescents in Need (ATTAIN) project. Addiction (Abingdon, England) 2-11-2004. 99 (#Issue#) 140-150. **Keywords:** CINAHL/Blacks -- In Adolescence -- United States/Cultural Sensitivity/Hispanics -- In Adolescence -- United States/Substance Abuse -- Therapy -- In Adolescence/adolescence/Clinical Trials/Coefficient Alpha/Funding Source/Interview Guides/Juvenile Offenders/multiple regression/Questionnaires/Random Assignment/Scales/Self Report/structured interview/T-Tests/United States/human.

RefID:8662. Baker, A., Lee, N. K., Claire, M., Lewin, T. J., Grant, T., Pohlman, S., Saunders, J. B., Kay-Lambkin, F., Constable, P., Jenner, L., and Carr, V. J.. Drug use patterns and mental health of regular amphetamine users during a reported 'heroin drought'. Addiction (Abingdon, England) 2004. 99 (7) 875-884. **Keywords:** CINAHL/Amphetamines/Mental Health/Substance Abuse -- Psychosocial Factors/adolescence/Adult/analysis of covariance/Analysis of Variance/Brief Symptom Inventory/chi square test/Cross Sectional Studies/Data Analysis Software/Descriptive Statistics/Female/Funding Source/Interviews/Male/Middle Age/New South Wales/Psychological Tests/Queensland/Questionnaires/Summated Rating Scaling/T-Tests/human.

RefID:8714. Tait, R. J., Hulse, G. K., and Robertson, S. I.. Effectiveness of a brief-intervention and continuity of care in enhancing attendance for treatment by adolescent substance users. Drug & Alcohol Dependence 11-6-2004. 74 (3) 289-296. **Keywords:** CINAHL/Outcomes (Health Care)/Substance Abuse -- Therapy -- In Adolescence/adolescence/chi square test/Clinical Trials/Confidence Intervals/Continuity of Patient Care/Descriptive Statistics/Female/Fisher's Exact Test/Funding Source/Interviews/Male/multiple regression/post hoc analysis/Questionnaires/Random Assignment/Western Australia/human.

RefID:8732. Hien, D. A., Cohen, L. R., Miele, G. M., Litt, L. C., and Capstick, C.. Promising treatments for women with comorbid PTSD and substance use disorders. American Journal of Psychiatry 2004. 161 (8) 1426-1432. **Keywords:** CINAHL/Cognitive Therapy -- Evaluation/Comorbidity/Stress Disorders,Post-Traumatic -- Therapy/Substance Abuse -- Therapy/Adult/Analysis of Variance/Audiorecording/Bivariate Statistics/Comparative Studies/Descriptive Statistics/Female/Hamilton Rating Scale for Depression/Impact of Events Scale/Middle Age/P-Value/post hoc analysis/Pretest-Posttest Design/Psychological Tests/Quasi-Experimental Studies/Recurrence -- Prevention and Control/Repeated Measures/Scales/structured interview/Funding Source/human.

RefID:8757. Brooner, R. K., Kidorf, M. S., King, V. L., Stoller, K. B., Neufeld, K. J., and Kolodner, K.. Comparing adaptive stepped care and monetary-based voucher interventions for opioid dependence. Drug & Alcohol Dependence 2-5-2007. 88 (#Issue#) S14-S23. **Keywords:** CINAHL/Behavior Modification -- Methods/Narcotics/Reinforcement (Psychology) -- Methods/Substance Dependence -- Therapy/Adult/Analysis of Variance/chi square test/Clinical Trials/Confidence Intervals/Counseling/Cox Proportional Hazards Model/Descriptive Statistics/Factorial Design/Female/Funding Source/Male/Maryland/Methadone -- Administration and Dosage/Methadone -- Therapeutic Use/Middle Age/Odds Ratio/Random Assignment/Substance Abuse Detection/Urinalysis/human.

RefID:8778. Krupski, L. A.. Promoting mindfulness and readiness to change: a comparison of teaching strategies for college students mandated to alcohol and drug education. #journal name# 2005. #volume# (#Issue#) #Start Page#-100. **Keywords:** CINAHL/Health Education/Students,College/Substance Abuse -- Education/Teaching Methods/Analysis of Variance/Attitude to Change/Experimental Studies/Repeated Measures/Student Satisfaction/human.

RefID:8830. Alterman, A. I., Koppenhaver, J. M., Mulholland, E., Ladden, L. J., and Baime, M. J.. Pilot trial of effectiveness of mindfulness meditation for substance abuse patients. Journal of Substance Use 2004. 9 (6) 259-268. **Keywords:** CINAHL/meditation/Substance Abuse -- Rehabilitation/Substance Abusers -- Psychosocial Factors/Clinical Assessment Tools/Descriptive Statistics/Female/Male/outcomes research/P-Value/Pennsylvania/Pilot Studies/Research Instruments/Scales/Short Form-36 Health Survey (SF-36)/Substance Abuse -- Urine/human.

RefID:8967. Baker, A., Lewin, T., Reichler, H., Clancy, R., Carr, V., Garrett, R., Sly, K., Devir, H., and Terry, M.. Evaluation of a motivational interview for substance use within psychiatric in-patient services. Addiction (Abingdon, England) 2002. 97 (10) 1329-1337. **Keywords:** CINAHL/Motivation -- Evaluation/Substance Abuse -- Therapy/Interviews -- Evaluation/Behavior Therapy -- Evaluation/Research Instruments/Inpatients/Mental Disorders/Psychiatric Patients/Comorbidity/Random Assignment/Self Care/Pamphlets -- Utilization/structured interview/New South Wales/Data Analysis Software/Repeated Measures/analysis of covariance/chi square test/Male/Adult/Treatment Outcomes/Prospective Studies/Brief Symptom Inventory/Diagnosis,Dual (Psychiatry)/Evaluation Research/Funding Source/human.

RefID:8977. Garfein, R. S., Swartzendruber, A., Ouellet, L. J., Kapadia, F., Hudson, S. M., Thiede, H., Strathdee, S. A., Williams, I. T., Bailey, S. L., Hagan, H., Golub, E. T., Kerndt, P., Hanson, D. L., and Latka, M. H.. Methods to recruit and retain a cohort of young-adult injection drug users for the Third Collaborative Injection Drug Users Study/Drug User Interventional Trial (CIDUS III/DUIT). Drug & Alcohol Dependence 2-11-2007. 91 (#Issue#) S4-17. **Keywords:** CINAHL/Patient Selection/Substance Abuse,Intravenous -- Complications/Substance Abuse,Intravenous -- Prevention and Control/Substance Abuse,Intravenous -- Psychosocial Factors/adolescence/Adult/Clinical Trials/Consent/Economics/Female/Hepatitis C -- Diagnosis/Hepatitis C -- Prevention and Control/HIV Infections -- Diagnosis/HIV Infections -- Prevention and Control/Male/Prospective Studies/Risk Taking Behavior/Socioeconomic Factors/study design/Unsafe Sex -- Prevention and Control/human.

RefID:8983. Petry, N. M., Tedford, J., Austin, M., Nich, C., Carroll, K. M., and Rounsaville, B. J.. Prize reinforcement contingency management for treating cocaine users: how low can we go, and with whom?. Addiction (Abingdon, England) 2004. 99 (3) 349-360. **Keywords:** CINAHL/Cocaine/Reward/Substance Use Rehabilitation Programs -- Methods/Adult/Analysis of Variance/chi square test/Clinical Trials/Community Health Services/Data Analysis Software/Descriptive Statistics/Female/Funding Source/Kruskal-Wallis Test/Male/Mann-Whitney U Test/post hoc analysis/Psychological Tests/Substance Abuse Detection/Urinalysis/human.

RefID:9008. Davis, T. M., Baer, J. S., Saxon, A. J., and Kivlahan, D. R.. Brief motivational feedback improves post-incarceration treatment contact among veterans with substance use disorders. Drug & Alcohol Dependence 1-3-2003. 69 (2) 197-203. **Keywords:** CINAHL/Substance Use Disorders -- Therapy/Motivation/Feedback/Behavior Therapy -- Methods/Veterans/Public Offenders/Funding Source/Clinical Trials/Questionnaires/Checklists/structured interview/Appointments and Schedules/Patient Compliance/Random Assignment/Data Analysis Software/Psychological Tests/Female/Male/Adult/Middle Age/chi square test/T-Tests/Logistic Regression/Treatment Outcomes/human.

RefID:9092. Saleh, S. S., Vaughn, T., Hall, J., Levey, S., Fuortes, L., and Uden-Holmen, T.. The effect of case management in substance abuse on health services use. Care Management Journals 2003. 4 (2) 82-87. **Keywords:** CINAHL/Case Management -- Evaluation/Case Management -- Methods/Health Services -- Utilization/Substance Abuse -- Therapy/Adult/Age Factors/Comorbidity/convenience sample/descriptive research/Descriptive Statistics/Emergency Care -- Utilization/Experimental Studies/Female/Length of Stay/Logistic Regression/Male/Mental Health Services -- Utilization/Middle Age/Office Visits -- Utilization/Patient Admission/Prospective Studies/Questionnaires/Random Assignment/Repeated Measures/Residential Care -- Utilization/Sex Factors/Social Workers/Substance Abusers/Univariate Statistics/human.

RefID:9106. D'Amico, E. J. and Fromme, K.. Brief prevention for adolescent risk-taking behavior. Addiction (Abingdon, England) 2002. 97 (5) 563-574. **Keywords:** CINAHL/Risk Taking Behavior -- Prevention and Control -- In Adolescence/Substance Abuse -- Prevention and Control -- In

Adolescence/Health Education -- In Adolescence/adolescence/Prospective Studies/Random Assignment/Alcohol Abuse -- Prevention and Control -- In Adolescence/Schools,Secondary/Questionnaires/Comparative Studies/Psychological Tests/Female/Male/Adult/multivariate analysis of variance/Peer Group/Behavioral Changes -- In Adolescence/School Health Education/Funding Source/human.

RefID:9119. Fearnow-Kenney, M. D., Wyrick, D. L., Jackson-Newsom, J., Wyrick, C. H., and Hansen, W. B.. Initial indicators of effectiveness for a high school drug prevention program. American Journal of Health Education 2003. 34 (2) 66-#End Page#. **Keywords:** CINAHL/Substance Abuse -- Prevention and Control -- In Adolescence/Risk Taking Behavior -- In Adolescence/Health Behavior -- In Adolescence/School Health Education/Education,Continuing (Credit)/Program Evaluation/Evaluation Research/exploratory research/Comparative Studies/Random Assignment/Pretest-Posttest Design/Surveys/Students,High School/Focus Groups/Student Attitudes/Attitude to Health/Outcomes of Education/Internal Consistency/Confidence Intervals/Descriptive Statistics/Coefficient Alpha/Criterion-Related Validity/Scales/P-Value/Discriminant Validity/multiple regression/chi square test/Race Factors/Age Factors/Sex Factors/Male/Female/adolescence/Adult/Whites/Blacks/Asians/Hispanics/Native Americans/Schools,Secondary/Suburban Areas/North Carolina/human.

RefID:9189. Wolchik, S. A., Sandler, I. N., Millsap, R. E., Plummer, B. A., Greene, S. M., Anderson, E. R., Dawson-McClure, S. R., Hipke, K., and Haine, R. A.. Six-year follow-up of preventive interventions for children of divorce: a randomized controlled trial. JAMA: Journal of the American Medical Association 16-10-2002. 288 (15) 1874-1881. **Keywords:** CINAHL/Divorce -- Psychosocial Factors/Mental Health -- In Adolescence/Mental Disorders -- Prevention and Control -- In Adolescence/early intervention/Risk Taking Behavior -- In Adolescence/Adolescent Behavior/Clinical Trials/Pretest-Posttest Design/Support,Psychosocial/Child Behavior Checklist/Diagnosis,Psychosocial/Sexual Partners/Substance Use Disorders/Interviews/Descriptive Statistics/analysis of covariance/Logistic Regression/chi square test/Confidence Intervals/Odds Ratio/Prospective Studies/adolescence/Child/Psychological Tests/Funding Source/human.

RefID:9192. Finke, L., Williams, J., Ritter, M., Kemper, D., Kersey, S., Nightenhauser, J., Autry, K., Going, C., Wulfman, G., and Hail, A.. Survival against drugs: education for school-age children. Journal of Child & Adolescent Psychiatric Nursing 2002. 15 (4) 163-169. **Keywords:** CINAHL/Family -- Psychosocial Factors/Peer Group -- In Infancy and Childhood/Substance Abuse -- Prevention and Control -- In Infancy and Childhood/Substance Abuse -- Psychosocial Factors -- In Infancy and Childhood/chi square test/Child/Descriptive Statistics/P-Value/Pretest-Posttest Design/Questionnaires/Scales/Self-Efficacy -- In Infancy and Childhood/Students,Elementary/T-Tests/Test-Retest Reliability/human.

RefID:9218. Saleh, S. S., Vaughn, T., Hall, J., Levey, S., Fuortes, L., and Uden-Holmen, T.. Effectiveness of case management in substance abuse treatment. Care Management Journals 2002. 3 (4) 172-177. **Keywords:** CINAHL/Case Management/substance abuse/Adult/Analysis of

Variance/Case Management -- Methods/Clinical Trials/Confidence Intervals/convenience sample/Descriptive Statistics/Employment/Female/Logistic Regression/Male/Middle Age/outcome assessment/outcomes research/Professional-Patient Relations/Repeated Measures/Research Subject Retention/Residential Facilities/Severity of Illness Indices/Social Workers/Telecommunications/human.

RefID:9283. Eggert, L. L., Thompson, E. A., Randell, B. P., and Pike, K. C.. Preliminary effects of brief school-based prevention approaches for reducing youth suicide -- risk behaviors, depression, and drug involvement. Journal of Child & Adolescent Psychiatric Nursing 2002. 15 (2) 48-64. **Keywords:** CINAHL/Suicide -- Prevention and Control -- In Adolescence/Substance Abuse -- Prevention and Control -- In Adolescence/Depression -- Prevention and Control -- In Adolescence/School Health Services/Crisis Intervention/Funding Source/Intervention Trials/Models,Theoretical/Repeated Measures/Random Assignment/Interviews/Questionnaires/chi square test/P-Value/Interrater Reliability/videorecording/Descriptive Statistics/Coefficient Alpha/Internal Consistency/Pearson's Correlation Coefficient/Analysis of Variance/Summated Rating Scaling/T-Tests/Factor Analysis/construct validity/Students,High School/Scales/adolescence/Adult/Male/Female/human.

RefID:9308. Williams, M., McCoy, H. V., Bowen, A., Saunders, L., Freeman, R., and Chen, D.. An evaluation of a brief HIV risk reduction intervention using empirically derived drug use and sexual risk indices. AIDS & Behavior 2001. 5 (1) 31-43. **Keywords:** CINAHL/Risk Taking Behavior/HIV Infections -- Prevention and Control/Health Promotion/Health Education/Behavioral Changes -- Evaluation/Funding Source/Cluster Analysis/Comparative Studies/United States/Puerto Rico/Research Subject Recruitment/Self Report/Urinalysis/Heroin -- Urine/Cocaine -- Urine/Research Subjects -- Economics/Interviews/Random Assignment/statistical significance/Pretest-Posttest Design/Questionnaires/Research Instruments/Intravenous Drug Users/substance abuse/Sexual Partners/Condoms -- Utilization/reliability/Test-Retest Reliability/Male/Female/Blacks/Whites/Hispanics/adolescence/Adult/Middle Age/Aged/Aged,80 and over/Descriptive Statistics/T-Tests/human.

RefID:9354. Williams, P. G., Allard, A., Sears, L., Dalrymple, N., and Bloom, A. S.. Brief report: case reports on naltrexone use in children with autism: controlled observations regarding benefits and practical issues of medication management. Journal of Autism & Developmental Disorders 2001. 31 (1) 103-108. **Keywords:** CINAHL/Autistic Disorder -- Drug Therapy/Naltrexone -- Therapeutic Use/Attention -- Drug Effects/Autistic Disorder -- Diagnosis/Case Control Studies/Child Behavior Disorders -- Diagnosis/Child Behavior Disorders -- Drug Therapy/Child/Child,Preschool/Double-Blind Studies/Male/Naltrexone -- Adverse Effects/Personality Assessment/Social Behavior/human.

RefID:10607. Werch, Chudley E., Moore, Michele J., and Diclemente, Carlo C.. Brief Image-Based Health Behavior Messages for Adolescents and Their Parents. Journal of Child & Adolescent Substance Abuse 2008. 17 (4) 19-40. **Keywords:** Parent Materials/Physical

Activities/Caregivers/Health Behavior/drug use/drinking/Adolescents/Parents/High School Students/Multivariate Analysis/Marijuana/substance abuse/information dissemination/ERIC RCTS.

RefID:10659. Stice, Eric, Rohde, Paul, Seeley, John R., and Gau, Jeff M.. Brief Cognitive-Behavioral Depression Prevention Program for High-Risk Adolescents Outperforms Two Alternative Interventions: A Randomized Efficacy Trial. Journal of consulting and clinical psychology 2008. 76 (4) 595-606. **Keywords:** Cognitive Restructuring/behavior modification/Intervention/prevention/Adolescents/Social Adjustment/Bibliotherapy/Depression (Psychology)/At Risk Persons/Symptoms (Individual Disorders)/Comparative Analysis/Counseling Techniques/behavior change/substance abuse/Pretests Posttests/Followup Studies/High School Students/group therapy/ERIC RCTS.

RefID:10796. Prado, G., Pantin, H., Briones, E., Schwartz, S. J., Feaster, D., Huang, S., Sullivan, S., Tapia, M. I., Sabillon, E., Lopez, B., and Szapocznik, J.. A randomized controlled trial of a parent-centered intervention in preventing substance use and HIV risk behaviors in Hispanic adolescents. J Consult.Clin Psychol. 2007. 75 (6) 914-926. **Keywords:** Adolescent/Adult/Female/HIV Infections/prevention & control/Hispanic Americans/statistics & numerical data/Humans/Male/Parent-Child Relations/Parenting/Parents/Education/Risk-Taking/Smoking Cessation/Substance-Related Disorders/from SR bibliographies.

RefID:10798. Stanton, B., Fang, X., Li, X., Feigelman, S., Galbraith, J., and Ricardo, I.. Evolution of risk behaviors over 2 years among a cohort of urban African American adolescents. Arch Pediatr Adolesc.Med 1997. 151 (4) 398-406. **Keywords:** Acquired Immunodeficiency Syndrome/prevention & control/Adolescent/Adolescent Behavior/African Americans/Alcohol Drinking/epidemiology/Child/Cohort Studies/Female/Humans/Longitudinal Studies/Male/Prevalence/Questionnaires/Risk-Taking/Sexual Behavior/statistics & numerical data/Smoking/Substance-Related Disorders/United States/Urban Population/from SR bibliographies.

Level 2: Population seeking or receiving treatment, previously diagnosed with a substance use disorder

RefID:2. Kirtadze, Irma, Otiashvili, David, O'Grady, Kevin E., and Jones, Hendree E.. Behavioral treatment + naltrexone reduces drug use and legal problems in the Republic of Georgia. The American journal of drug and alcohol abuse 2012. 38 (2) 171-175. **Keywords:** #Keywords#.

RefID:11. Killeen, Therese K., McRae-Clark, Aimee L., Waldrop, Angela E., Upadhyaya, Himanshu, and Brady, Kathleen T.. Contingency management in community programs treating adolescent substance abuse: a feasibility study. Journal of child and adolescent psychiatric nursing : official publication of the Association of Child and Adolescent Psychiatric Nurses, Inc 2012. 25 (1) 33-41. **Keywords:** #Keywords#.

RefID:18. Luoma, Jason B., Kohlenberg, Barbara S., Hayes, Steven C., and Fletcher, Lindsay. Slow and steady wins the race: a randomized clinical trial of acceptance and commitment therapy targeting shame in substance use disorders. Journal of consulting and clinical psychology 2012. 80 (1) 43-53. **Keywords:** #Keywords#.

RefID:39. Secades-Villa, Roberto, Sanchez-Hervas, Emilio, Zacares-Romaguera, Francisco, Garcia-Rodriguez, Olaya, Santonja-Gomez, Francisco J., and Garcia-Fernandez, Gloria. Community Reinforcement Approach (CRA) for cocaine dependence in the Spanish public health system: 1 year outcome. Drug and alcohol review 2011. 30 (6) 606-612. **Keywords:** #Keywords#.

RefID:72. Schottenfeld, Richard S., Moore, Brent, and Pantalon, Michael V.. Contingency management with community reinforcement approach or twelve-step facilitation drug counseling for cocaine dependent pregnant women or women with young children. Drug and alcohol dependence 2011. 118 (1) 48-55. **Keywords:** Adult/*Alcoholism/ep [Epidemiology]/Alcoholism/px [Psychology]/Ambulatory Care/*Behavior Therapy/mt [Methods]/Central Nervous System Stimulants/ur [Urine]/Cocaine/ur [Urine]/*Cocaine-Related Disorders/px [Psychology]/*Cocaine-Related Disorders/rh [Rehabilitation]/Cocaine-Related Disorders/th [Therapy]/Comorbidity/*Depressive Disorder,Major/ep [Epidemiology]/Depressive Disorder,Major/px [Psychology]/Female/Humans/Male/Maternal Behavior/px [Psychology]/Pregnancy/Pregnancy Complications/px [Psychology]/*Pregnancy Complications/rh [Rehabilitation]/Pregnancy Complications/th [Therapy]/Pregnant Women/px [Psychology]/Psychiatric Status Rating Scales/Reinforcement (Psychology)/Residence Characteristics/Reward/*Self-Help Groups/Time Factors/Treatment Outcome/Urban Population.

RefID:75. Fox, Court, Towe, Sheri L., Stephens, Robert S., Walker, Denise D., and Roffman, Roger A.. Motives for cannabis use in high-risk adolescent users. Psychology of addictive behaviors : journal of the Society of Psychologists in Addictive Behaviors 2011. 25 (3) 492-500. **Keywords:** Adaptation,Psychological/Adolescent/Female/Humans/Male/*Marijuana Abuse/px [Psychology]/Marijuana Abuse/th [Therapy]/*Marijuana Smoking/px [Psychology]/Marijuana Smoking/th [Therapy]/*Motivation/Risk Factors.

RefID:89. Hser, Yih Ing, Li, Jianhua, Jiang, Haifeng, Zhang, Ruimin, Du, Jiang, Zhang, Congbin, Zhang, Bo, Evans, Elizabeth, Wu, Fei, Chang, Yen Jung, Peng, Chinyi, Huang, David, Stitzer, Maxine L., Roll, John, and Zhao, Min. Effects of a randomized contingency management intervention on opiate abstinence and retention in methadone maintenance treatment in China. Addiction (Abingdon, England) 2011. 106 (10) 1801-1809. **Keywords:** Adult/Analysis of Variance/China/Female/HIV Infections/ep [Epidemiology]/HIV Infections/pc [Prevention & Control]/Humans/Male/Methadone/ad [Administration & Dosage]/*Methadone/tu [Therapeutic Use]/Narcotic Antagonists/ad [Administration & Dosage]/*Narcotic Antagonists/tu [Therapeutic Use]/*Opiate Substitution Treatment/mt [Methods]/Opioid-Related Disorders/px [Psychology]/*Opioid-Related Disorders/rh [Rehabilitation]/Opioid-

Related Disorders/ur [Urine]/*Patient Compliance/sn [Statistics & Numerical Data]/Patient Dropouts/sn [Statistics & Numerical Data]/Recurrence/Reward/Substance Abuse,Intravenous/px [Psychology]/*Substance Abuse,Intravenous/rh [Rehabilitation]/Substance Abuse,Intravenous/ur [Urine]/Time Factors/Treatment Outcome.

RefID:120. Schwartz, Robert P., Kelly, Sharon M., O'Grady, Kevin E., Gandhi, Devang, and Jaffe, Jerome H.. Interim methadone treatment compared to standard methadone treatment: 4-month findings. Journal of substance abuse treatment 2011. 41 (1) 21-29. **Keywords:** Adult/*Cocaine-Related Disorders/dt [Drug Therapy]/Counseling/Crime/Female/*Heroin Dependence/dt [Drug Therapy]/Humans/Male/*Methadone/ad [Administration & Dosage]/Methadone/tu [Therapeutic Use]/Middle Aged.

RefID:123. Ogel, Kultegin and Coskun, Sibel. Cognitive behavioral therapy-based brief intervention for volatile substance misusers during adolescence: a follow-up study. Substance use & misuse 2011. 46 Suppl 1 (#Issue#) 128-133. **Keywords:** Adolescent/*Cognitive Therapy/mt [Methods]/Follow-Up Studies/Humans/Inhalant Abuse/di [Diagnosis]/Inhalant Abuse/pc [Prevention & Control]/*Inhalant Abuse/rh [Rehabilitation]/Male/Outcome Assessment (Health Care)/Turkey/*Volatile Organic Compounds/ad [Administration & Dosage].

RefID:145. Jones, Hendree E., Tuten, Michelle, and O'Grady, Kevin E.. Treating the partners of opioid-dependent pregnant patients: feasibility and efficacy. The American journal of drug and alcohol abuse 2011. 37 (3) 170-178. **Keywords:** Adult/Counseling/Feasibility Studies/Female/Humans/Interpersonal Relations/*Interview,Psychological/Male/*Methadone/tu [Therapeutic Use]/Middle Aged/*Motivation/Opiate Substitution Treatment/*Opioid-Related Disorders/dt [Drug Therapy]/*Opioid-Related Disorders/px [Psychology]/Opioid-Related Disorders/rh [Rehabilitation]/Pregnancy/Pregnancy Complications/Prospective Studies/Questionnaires/Sexual Partners/px [Psychology]/Socioeconomic Factors/Treatment Outcome/Young Adult.

RefID:163. Stein, Michael D., Hagerty, Claire E., Herman, Debra S., Phipps, Maureen G., and Anderson, Bradley J.. A brief marijuana intervention for non-treatment-seeking young adult women. Journal of substance abuse treatment 2011. 40 (2) 189-198. **Keywords:** Adolescent/Female/Follow-Up Studies/Humans/*Marijuana Abuse/rh [Rehabilitation]/Marijuana Smoking/ep [Epidemiology]/Marijuana Smoking/pc [Prevention & Control]/*Motivation/*Psychotherapy,Brief/mt [Methods]/Time Factors/Young Adult.

RefID:168. Kay-Lambkin, Frances, Baker, Amanda, Lewin, Terry, and Carr, Vaughan. Acceptability of a clinician-assisted computerized psychological intervention for comorbid mental health and substance use problems: treatment adherence data from a randomized controlled trial. Journal of medical Internet research 2011. 13 (1) e11-#End Page#. **Keywords:** Adult/Alcoholism/ep [Epidemiology]/Alcoholism/th [Therapy]/Comorbidity/*Depression/ep [Epidemiology]/*Depression/th [Therapy]/Humans/Linear Models/Marijuana Abuse/ep [Epidemiology]/Marijuana Abuse/th [Therapy]/Middle Aged/*Patient Acceptance of Health

Care/Patient Compliance/*Psychological Techniques/*Substance-Related Disorders/ep [Epidemiology]/*Substance-Related Disorders/th [Therapy]/*Therapy,Computer-Assisted.

RefID:172. Robbins, Michael S., Feaster, Daniel J., Horigian, Viviana E., Puccinelli, Marc J., Henderson, Craig, and Szapocznik, Jose. Therapist adherence in brief strategic family therapy for adolescent drug abusers. Journal of consulting and clinical psychology 2011. 79 (1) 43-53. **Keywords:** Adolescent/Child/Family/px [Psychology]/*Family Therapy/Female/Humans/Male/*Patient Compliance/px [Psychology]/*Psychotherapy,Brief/Substance-Related Disorders/px [Psychology]/*Substance-Related Disorders/th [Therapy]/Treatment Outcome.

RefID:237. Olmstead, Todd A., Ostrow, Cary D., and Carroll, Kathleen M.. Cost-effectiveness of computer-assisted training in cognitive-behavioral therapy as an adjunct to standard care for addiction. Drug and alcohol dependence 2010. 110 (3) 200-207. **Keywords:** *Behavior,Addictive/th [Therapy]/*Cognitive Therapy/ec [Economics]/Cost-Benefit Analysis/Female/Humans/Male/*Substance-Related Disorders/th [Therapy]/Temperance/*Therapy,Computer-Assisted/ec [Economics]/Time Factors/Treatment Outcome.

RefID:260. Petry, Nancy M., Alessi, Sheila M., Ledgerwood, David M., and Sierra, Sean. Psychometric properties of the contingency management competence scale. Drug and alcohol dependence 2010. 109 (1-3) 167-174. **Keywords:** Adult/Clinical Competence/Cocaine-Related Disorders/px [Psychology]/Community Health Services/Data Interpretation,Statistical/Empathy/Factor Analysis,Statistical/Female/Humans/Male/Middle Aged/Observer Variation/Predictive Value of Tests/Principal Component Analysis/*Psychological Tests/*Psychometrics/Reproducibility of Results/Substance Abuse Treatment Centers/Substance-Related Disorders/ep [Epidemiology]/*Substance-Related Disorders/px [Psychology]/*Substance-Related Disorders/th [Therapy]/Treatment Outcome.

RefID:285. Stitzer, Maxine L., Petry, Nancy M., and Peirce, Jessica. Motivational incentives research in the National Drug Abuse Treatment Clinical Trials Network. Journal of substance abuse treatment 2010. 38 Suppl 1 (#Issue#) S61-S69. **Keywords:** Adult/Amphetamine-Related Disorders/px [Psychology]/*Amphetamine-Related Disorders/rh [Rehabilitation]/Cocaine-Related Disorders/px [Psychology]/*Cocaine-Related Disorders/rh [Rehabilitation]/Counseling/mt [Methods]/Female/Follow-Up Studies/Humans/Male/*Methadone/tu [Therapeutic Use]/Middle Aged/*Motivation/Narcotics/tu [Therapeutic Use]/National Institute on Drug Abuse (U.S.)/Randomized Controlled Trials as Topic/Recurrence/pc [Prevention & Control]/Reward/Substance Abuse Detection/Substance Abuse Treatment Centers/mt [Methods]/Time Factors/Treatment Outcome/United States.

RefID:310. Petry, Nancy M., Weinstock, Jeremiah, Alessi, Sheila M., Lewis, Marilyn W., and Dieckhaus, Kevin. Group-based randomized trial of contingencies for health and abstinence in HIV patients. Journal of consulting and clinical psychology 2010. 78 (1) 89-97. **Keywords:**

Adult/*Behavior Therapy/mt [Methods]/Chi-Square Distribution/Cocaine-Related Disorders/co [Complications]/Cocaine-Related Disorders/px [Psychology]/*Cocaine-Related Disorders/th [Therapy]/Female/HIV Infections/co [Complications]/*HIV Infections/px [Psychology]/*Health Behavior/*Health Promotion/mt [Methods]/Humans/Intention to Treat Analysis/Male/Middle Aged/Motivation/Opioid-Related Disorders/co [Complications]/Opioid-Related Disorders/px [Psychology]/*Opioid-Related Disorders/th [Therapy]/*Psychotherapy,Group/mt [Methods]/Questionnaires/Reinforcement Schedule/Severity of Illness Index/Substance Abuse Treatment Centers/Treatment Outcome/Viral Load.

RefID:351. Robbins, Michael S., Szapocznik, Jose, Horigian, Viviana E., Feaster, Daniel J., Puccinelli, Marc, Jacobs, Petra, Burlew, Kathy, Werstlein, Robert, Bachrach, Ken, and Brigham, Greg. Brief strategic family therapy for adolescent drug abusers: a multi-site effectiveness study. Contemporary clinical trials 2009. 30 (3) 269-278. **Keywords:** Adolescent/Combined Modality Therapy/Conduct Disorder/px [Psychology]/Conduct Disorder/rh [Rehabilitation]/Family Conflict/px [Psychology]/*Family Therapy/Female/Follow-Up Studies/Humans/Juvenile Delinquency/px [Psychology]/Juvenile Delinquency/rh [Rehabilitation]/Male/*Psychotherapy,Brief/*Street Drugs/Substance-Related Disorders/px [Psychology]/*Substance-Related Disorders/rh [Rehabilitation]/Treatment Outcome/Unsafe Sex/pc [Prevention & Control].

RefID:383. Garcia-Rodriguez, Olaya, Secades-Villa, Roberto, Higgins, Stephen T., Fernandez-Hermida, Jose R., Carballo, Jose L., Errasti Perez, Jose M., and Al-halabi Diaz, Susana. Effects of voucher-based intervention on abstinence and retention in an outpatient treatment for cocaine addiction: a randomized controlled trial. Experimental and clinical psychopharmacology 2009. 17 (3) 131-138. **Keywords:** Adult/Ambulatory Care/*Cocaine-Related Disorders/th [Therapy]/*Community Mental Health Services/og [Organization & Administration]/Female/Humans/Male/*Reinforcement (Psychology)/Reinforcement Schedule/*Retention (Psychology)/*Token Economy.

RefID:413. Olmstead, Todd A. and Petry, Nancy M.. The cost-effectiveness of prize-based and voucher-based contingency management in a population of cocaine- or opioid-dependent outpatients. Drug and alcohol dependence 2009. 102 (1-3) 108-115. **Keywords:** Ambulatory Care/ec [Economics]/Cocaine-Related Disorders/ec [Economics]/Cocaine-Related Disorders/px [Psychology]/*Cocaine-Related Disorders/rh [Rehabilitation]/Cost-Benefit Analysis/Counseling/ec [Economics]/Heroin Dependence/ec [Economics]/Heroin Dependence/px [Psychology]/Heroin Dependence/rh [Rehabilitation]/Humans/*Motivation/Opioid-Related Disorders/ec [Economics]/Opioid-Related Disorders/px [Psychology]/*Opioid-Related Disorders/rh [Rehabilitation]/Patient Education as Topic/ec [Economics]/Sample Size/Substance Abuse Detection/ec [Economics]/Substance Abuse Treatment Centers/Treatment Outcome.

RefID:489. Rash, Carla J., Alessi, Sheila M., and Petry, Nancy M.. Contingency management is efficacious for cocaine abusers with prior treatment attempts. Experimental and clinical psychopharmacology 2008. 16 (6) 547-554. **Keywords:** Adult/Age Factors/*Alcohol-Related

Disorders/co [Complications]/*Ambulatory Care/mt [Methods]/Cocaine-Related Disorders/co [Complications]/*Cocaine-Related Disorders/rh [Rehabilitation]/Employment/Female/Humans/Male/Recurrence/pc [Prevention & Control]/Severity of Illness Index/Time Factors/Treatment Outcome.

RefID:499. Kaminer, Yifrah and Burleson, Joseph A.. Does temperament moderate treatment response in adolescent substance use disorders?. Substance abuse : official publication of the Association for Medical Education and Research in Substance Abuse 2008. 29 (2) 89-95. **Keywords:** Adolescent/Cognitive Therapy/Female/Humans/Male/Questionnaires/Risk Factors/Severity of Illness Index/Substance-Related Disorders/di [Diagnosis]/*Substance-Related Disorders/ep [Epidemiology]/*Substance-Related Disorders/rh [Rehabilitation]/*Temperament.

RefID:526. Petry, Nancy M., Lewis, Marilyn W., and Ostvik-White, Elin M.. Participation in religious activities during contingency management interventions is associated with substance use treatment outcomes. The American journal on addictions / American Academy of Psychiatrists in Alcoholism and Addictions 2008. 17 (5) 408-413. **Keywords:** Adult/Combined Modality Therapy/Demography/Diagnostic and Statistical Manual of Mental Disorders/Female/Humans/Male/Reinforcement (Psychology)/*Religion/*Social Behavior/*Substance-Related Disorders/rh [Rehabilitation].

RefID:544. Henggeler, Scott W., Sheidow, Ashli J., Cunningham, Phillippe B., Donohue, Bradley C., and Ford, Julian D.. Promoting the implementation of an evidence-based intervention for adolescent marijuana abuse in community settings: testing the use of intensive quality assurance. Journal of clinical child and adolescent psychology : the official journal for the Society of Clinical Child and Adolescent Psychology, American Psychological Association, Division 53 2008. 37 (3) 682-689. **Keywords:** Adolescent/Adult/*Cognitive Therapy/ed [Education]/*Community Mental Health Services/*Evidence-Based Medicine/Female/Follow-Up Studies/Humans/*Inservice Training/Male/*Marijuana Abuse/rh [Rehabilitation]/Middle Aged/*Quality Assurance,Health Care/Recurrence/*Token Economy.

RefID:737. Corrigan, John D. and Bogner, Jennifer. Interventions to promote retention in substance abuse treatment. Brain injury : [BI] 2007. 21 (4) 343-356. **Keywords:** Adult/Aged/Brain Injuries/co [Complications]/*Brain Injuries/px [Psychology]/Female/*Gift Giving/*Health Services Accessibility/Humans/Male/Middle Aged/*Motivation/*Patient Compliance/px [Psychology]/Professional-Patient Relations/Socioeconomic Factors/Substance-Related Disorders/co [Complications]/Substance-Related Disorders/px [Psychology]/*Substance-Related Disorders/rh [Rehabilitation].

RefID:769. Weinstock, Jeremiah, Alessi, Sheila M., and Petry, Nancy M.. Regardless of psychiatric severity the addition of contingency management to standard treatment improves retention and drug use outcomes. Drug and alcohol dependence 2007. 87 (2-3) 288-296. **Keywords:** Alcoholism/px [Psychology]/Cocaine-Related Disorders/px [Psychology]/Ethnic Groups/Female/Humans/Male/Opioid-Related Disorders/px [Psychology]/Personality

Inventory/Randomized Controlled Trials as Topic/Severity of Illness Index/Socioeconomic Factors/Substance-Related Disorders/pp [Physiopathology]/*Substance-Related Disorders/px [Psychology]/*Substance-Related Disorders/rh [Rehabilitation]/Suicide,Attempted/sn [Statistics & Numerical Data]/Treatment Outcome.

RefID:797. Mensinger, Janell Lynn, Diamond, Guy S., Kaminer, Yifrah, and Wintersteen, Matthew B.. Adolescent and therapist perception of barriers to outpatient substance abuse treatment. The American journal on addictions / American Academy of Psychiatrists in Alcoholism and Addictions 2006. 15 Suppl 1 (#Issue#) 16-25. **Keywords:** Adolescent/*Ambulatory Care/Female/*Health Services Accessibility/Humans/Male/Marijuana Abuse/px [Psychology]/*Marijuana Abuse/rh [Rehabilitation]/Motivation/Multicenter Studies as Topic/*Patient Dropouts/px [Psychology]/*Psychotherapy,Brief/Questionnaires/Randomized Controlled Trials as Topic.

RefID:833. Roll, John M. and Shoptaw, Steve. Contingency management: schedule effects. Psychiatry research 2006. 144 (1) 91-93. **Keywords:** Adolescent/Adult/Amphetamine-Related Disorders/px [Psychology]/*Amphetamine-Related Disorders/rh [Rehabilitation]/Amphetamine-Related Disorders/ur [Urine]/Female/Humans/Male/Methamphetamine/ur [Urine]/*Methamphetamine/Middle Aged/Motivation/Pilot Projects/*Reinforcement Schedule/Substance Abuse Detection/*Token Economy/Treatment Outcome.

RefID:872. Petry, Nancy M., Kolodner, Ken B., Li, Rui, Peirce, Jessica M., Roll, John M., Stitzer, Maxine L., and Hamilton, John A.. Prize-based contingency management does not increase gambling. Drug and alcohol dependence 2006. 83 (3) 269-273. **Keywords:** Adult/Alcoholism/px [Psychology]/Alcoholism/rh [Rehabilitation]/Ambulatory Care/Amphetamine-Related Disorders/px [Psychology]/*Amphetamine-Related Disorders/rh [Rehabilitation]/*Central Nervous System Stimulants/Cocaine-Related Disorders/px [Psychology]/*Cocaine-Related Disorders/rh [Rehabilitation]/Female/Follow-Up Studies/*Gambling/px [Psychology]/Humans/Male/Marijuana Abuse/px [Psychology]/Marijuana Abuse/rh [Rehabilitation]/Methadone/tu [Therapeutic Use]/*Methamphetamine/Middle Aged/Narcotics/tu [Therapeutic Use]/Opioid-Related Disorders/px [Psychology]/Opioid-Related Disorders/rh [Rehabilitation]/*Street Drugs/Substance Abuse Treatment Centers/*Token Economy/United States.

RefID:886. Timko, Christine, DeBenedetti, Anna, and Billow, Rachel. Intensive referral to 12-Step self-help groups and 6-month substance use disorder outcomes. Addiction (Abingdon, England) 2006. 101 (5) 678-688. **Keywords:** Female/Follow-Up Studies/Humans/Male/Middle Aged/Patient Compliance/*Referral and Consultation/st [Standards]/*Self-Help Groups/ut [Utilization]/*Substance-Related Disorders/rh [Rehabilitation]/Treatment Outcome.

RefID:887. Schroeder, Jennifer R., Epstein, David H., Umbricht, Annie, and Preston, Kenzie L.. Changes in HIV risk behaviors among patients receiving combined pharmacological and behavioral interventions for heroin and cocaine dependence. Addictive behaviors 2006. 31 (5)

868-879. **Keywords:** Adult/Cocaine-Related Disorders/co [Complications]/Cocaine-Related Disorders/px [Psychology]/*Cocaine-Related Disorders/rh [Rehabilitation]/Cognitive Therapy/Combined Modality Therapy/Female/HIV Infections/pc [Prevention & Control]/HIV Infections/px [Psychology]/*HIV Infections/tm [Transmission]/Heroin Dependence/co [Complications]/Heroin Dependence/px [Psychology]/*Heroin Dependence/rh [Rehabilitation]/Humans/Male/Methadone/tu [Therapeutic Use]/Middle Aged/Needle Sharing/ae [Adverse Effects]/Prostitution/*Risk-Taking/Sexual Behavior/Substance Abuse,Intravenous/px [Psychology]/Substance Abuse,Intravenous/rh [Rehabilitation]/Unsafe Sex.

RefID:896. Bellack, Alan S., Bennett, Melanie E., Gearon, Jean S., Brown, Clayton H., and Yang, Ye. A randomized clinical trial of a new behavioral treatment for drug abuse in people with severe and persistent mental illness. Archives of general psychiatry 2006. 63 (4) 426-432. **Keywords:** Ambulatory Care/*Behavior Therapy/mt [Methods]/Community Mental Health Services/Comorbidity/Depressive Disorder,Major/di [Diagnosis]/Depressive Disorder,Major/ep [Epidemiology]/Depressive Disorder,Major/th [Therapy]/Diagnosis,Dual (Psychiatry)/Female/Humans/Male/Mental Disorders/di [Diagnosis]/*Mental Disorders/ep [Epidemiology]/Middle Aged/Outcome Assessment (Health Care)/Psychiatric Status Rating Scales/Psychotherapy,Group/Psychotic Disorders/di [Diagnosis]/Psychotic Disorders/ep [Epidemiology]/Psychotic Disorders/th [Therapy]/Quality of Life/Schizophrenia/di [Diagnosis]/Schizophrenia/ep [Epidemiology]/Schizophrenia/th [Therapy]/Severity of Illness Index/Substance Abuse Detection/Substance-Related Disorders/di [Diagnosis]/Substance-Related Disorders/ep [Epidemiology]/*Substance-Related Disorders/th [Therapy]/Treatment Outcome.

RefID:898. Saxon, Andrew J., Malte, Carol A., Sloan, Kevin L., Baer, John S., Calsyn, Donald A., Nichol, Paul, Chapko, Michael K., and Kivlahan, Daniel R.. Randomized trial of onsite versus referral primary medical care for veterans in addictions treatment. Medical care 2006. 44 (4) 334-342. **Keywords:** Adult/Comorbidity/Confidence Intervals/Continuity of Patient Care/og [Organization & Administration]/Female/*Hospitals,Veterans/og [Organization & Administration]/Hospitals,Veterans/ut [Utilization]/Humans/Internal Medicine/Male/Middle Aged/Odds Ratio/Outcome Assessment (Health Care)/Patient Compliance/Patient Satisfaction/sn [Statistics & Numerical Data]/Primary Health Care/ec [Economics]/*Primary Health Care/ut [Utilization]/*Referral and Consultation/Substance Abuse Treatment Centers/ec [Economics]/*Substance Abuse Treatment Centers/ut [Utilization]/Substance-Related Disorders/ec [Economics]/*Substance-Related Disorders/th [Therapy]/Treatment Outcome/*Veterans/px [Psychology]/Washington.

RefID:915. Poling, James, Oliveto, Alison, Petry, Nancy, Sofuoglu, Mehmet, Gonsai, Kishorchandra, Gonzalez, Gerardo, Martell, Bridget, and Kosten, Thomas R.. Six-month trial of bupropion with contingency management for cocaine dependence in a methadone-maintained population. Archives of general psychiatry 2006. 63 (2) 219-228. **Keywords:** Adult/*Behavior Therapy/mt [Methods]/*Bupropion/tu [Therapeutic Use]/Cocaine/ur [Urine]/Cocaine-Related Disorders/ep [Epidemiology]/*Cocaine-Related Disorders/rh [Rehabilitation]/Cocaine-Related

Disorders/ur [Urine]/Combined Modality Therapy/Comorbidity/*Dopamine Uptake Inhibitors/tu [Therapeutic Use]/Double-Blind Method/Female/*Heroin Dependence/ep [Epidemiology]/Heroin Dependence/rh [Rehabilitation]/Humans/Male/*Methadone/tu [Therapeutic Use]/Placebos/Psychiatric Status Rating Scales/Reward/Substance Abuse Detection.

RefID:919. Rawson, Richard A., McCann, Michael J., Flammino, Frank, Shoptaw, Steven, Miotto, Karen, Reiber, Chris, and Ling, Walter. A comparison of contingency management and cognitive-behavioral approaches for stimulant-dependent individuals. Addiction (Abingdon, England) 2006. 101 (2) 267-274. **Keywords:** Adult/*Amphetamine-Related Disorders/th [Therapy]/*Cocaine-Related Disorders/th [Therapy]/*Cognitive Therapy/mt [Methods]/*Conditioning,Operant/Female/Humans/Male/Methamphetamine/Treatment Outcome.

RefID:923. Schwartz, Robert P., Highfield, David A., Jaffe, Jerome H., Brady, Joseph V., Butler, Carol B., Rouse, Charles O., Callaman, Jason M., O'Grady, Kevin E., and Battjes, Robert J.. A randomized controlled trial of interim methadone maintenance. Archives of general psychiatry 2006. 63 (1) 102-109. **Keywords:** Adult/Analgesics,Opioid/ad [Administration & Dosage]/*Analgesics,Opioid/tu [Therapeutic Use]/Baltimore/Cocaine-Related Disorders/rh [Rehabilitation]/Crime/pc [Prevention & Control]/Drug Administration Schedule/Female/Follow-Up Studies/*Heroin Dependence/rh [Rehabilitation]/Humans/Male/Methadone/ad [Administration & Dosage]/*Methadone/tu [Therapeutic Use]/Outcome Assessment (Health Care)/Patient Acceptance of Health Care/*Substance Abuse Treatment Centers/og [Organization & Administration]/Treatment Outcome/Waiting Lists.

RefID:953. Petry, Nancy M., Peirce, Jessica M., Stitzer, Maxine L., Blaine, Jack, Roll, John M., Cohen, Allan, Obert, Jeanne, Killeen, Therese, Saladin, Michael E., Cowell, Mark, Kirby, Kimberly C., Sterling, Robert, Royer-Malvestuto, Charlotte, Hamilton, John, Booth, Robert E., MacDonald, Marilyn, Liebert, Marc, Rader, Linda, Burns, Raynetta, DiMaria, Joan, Copersino, Marc, Stabile, Patricia Quinn, Kolodner, Ken, and Li, Rui. Effect of prize-based incentives on outcomes in stimulant abusers in outpatient psychosocial treatment programs: a national drug abuse treatment clinical trials network study. Archives of general psychiatry 2005. 62 (10) 1148-1156. **Keywords:** Adult/Alcoholism/px [Psychology]/Alcoholism/th [Therapy]/Alcoholism/ur [Urine]/*Ambulatory Care/Amphetamine-Related Disorders/px [Psychology]/Amphetamine-Related Disorders/th [Therapy]/*Central Nervous System Stimulants/ae [Adverse Effects]/Cocaine-Related Disorders/px [Psychology]/Cocaine-Related Disorders/th [Therapy]/Female/Humans/Male/Methamphetamine/ae [Adverse Effects]/Methamphetamine/ur [Urine]/Motivation/*Psychotherapy/mt [Methods]/Substance Abuse Detection/*Substance Abuse Treatment Centers/mt [Methods]/Substance-Related Disorders/px [Psychology]/*Substance-Related Disorders/th [Therapy]/Substance-Related Disorders/ur [Urine]/*Token Economy/Treatment Outcome.

RefID:960. Heil, Sarah H., Sigmon, Stacey C., Mongeon, Joan A., and Higgins, Stephen T..

Characterizing and improving HIV/AIDS knowledge among cocaine-dependent outpatients. Experimental and clinical psychopharmacology 2005. 13 (3) 238-243. **Keywords:** *Cocaine-Related Disorders/px [Psychology]/Cross-Over Studies/Female/*HIV Infections/Humans/Male/*Outpatients/px [Psychology]/Patient Education as Topic/mt [Methods]/*Patient Education as Topic.

RefID:984. Corrigan, John D., Bogner, Jennifer, Lamb-Hart, Gary, Heinemann, Allen W., and Moore, Dennis. Increasing substance abuse treatment compliance for persons with traumatic brain injury. Psychology of addictive behaviors : journal of the Society of Psychologists in Addictive Behaviors 2005. 19 (2) 131-139. **Keywords:** Adolescent/Adult/Aged/*Brain Injuries/ep [Epidemiology]/Female/Financial Support/Humans/Male/Middle Aged/Motivation/*Patient Compliance/sn [Statistics & Numerical Data]/*Substance-Related Disorders/ep [Epidemiology]/*Substance-Related Disorders/rh [Rehabilitation].

RefID:988. Oliveto, Alison, Poling, James, Sevarino, Kevin A., Gonsai, Kishorchandra R., McCance-Katz, Elinore F., Stine, Susan M., and Kosten, Thomas R.. Efficacy of dose and contingency management procedures in LAAM-maintained cocaine-dependent patients. Drug and alcohol dependence 2005. 79 (2) 157-165. **Keywords:** Adult/Ambulatory Care/*Analgesics,Opioid/tu [Therapeutic Use]/Cocaine/ur [Urine]/*Cocaine-Related Disorders/dt [Drug Therapy]/Connecticut/Dose-Response Relationship,Drug/Double-Blind Method/Female/Humans/Male/*Methadyl Acetate/tu [Therapeutic Use]/Middle Aged/Narcotics/ur [Urine]/*Opioid-Related Disorders/dt [Drug Therapy]/Opioid-Related Disorders/ur [Urine]/Treatment Outcome.

RefID:989. Jones, Hendree E., Wong, Conrad J., Tuten, Michelle, and Stitzer, Maxine L.. Reinforcement-based therapy: 12-month evaluation of an outpatient drug-free treatment for heroin abusers. Drug and alcohol dependence 2005. 79 (2) 119-128. **Keywords:** Adult/Aftercare/*Ambulatory Care/Baltimore/*Behavior Therapy/Female/Heroin/ur [Urine]/*Heroin/Humans/Male/*Opioid-Related Disorders/th [Therapy]/Opioid-Related Disorders/ur [Urine]/*Outcome and Process Assessment (Health Care)/*Reinforcement (Psychology)/Residence Characteristics/*Substance Abuse Detection/Time Factors.

RefID:1000. Rowan-Szal, Grace A., Bartholomew, Norma G., Chatham, Lois R., and Simpson, D. Dwayne. A combined cognitive and behavioral intervention for cocaine-using methadone clients. Journal of psychoactive drugs 2005. 37 (1) 75-84. **Keywords:** Adult/*Cocaine-Related Disorders/dt [Drug Therapy]/*Cocaine-Related Disorders/px [Psychology]/*Cognitive Therapy/mt [Methods]/Female/Follow-Up Studies/Humans/Male/*Methadone/tu [Therapeutic Use]/*Reward.

RefID:1029. McKay, James R., Lynch, Kevin G., Shepard, Donald S., and Pettinati, Helen M.. The effectiveness of telephone-based continuing care for alcohol and cocaine dependence: 24-month outcomes. Archives of general psychiatry 2005. 62 (2) 199-207. **Keywords:** *Aftercare/mt [Methods]/Alcoholism/rh [Rehabilitation]/*Alcoholism/th [Therapy]/Ambulatory Care/Clinical Protocols/st [Standards]/Cocaine-Related Disorders/rh [Rehabilitation]/*Cocaine-

Related Disorders/th [Therapy]/Cognitive Therapy/Continuity of Patient Care/Counseling/mt [Methods]/Follow-Up Studies/Humans/Psychotherapy,Group/mt [Methods]/Substance Abuse Detection/Telephone/st [Standards]/*Telephone/ut [Utilization]/Temperance/Treatment Outcome.

RefID:1050. McKay, James R., Lynch, Kevin G., Shepard, Donald S., Ratichek, Sara, Morrison, Rebecca, Koppenhaver, Janelle, and Pettinati, Helen M.. The effectiveness of telephone-based continuing care in the clinical management of alcohol and cocaine use disorders: 12-month outcomes. Journal of consulting and clinical psychology 2004. 72 (6) 967-979. **Keywords:** Adolescent/Adult/Aged/*Alcoholism/th [Therapy]/*Cocaine-Related Disorders/th [Therapy]/*Continuity of Patient Care/*Counseling/mt [Methods]/Female/Humans/Male/Middle Aged/Recurrence/pc [Prevention & Control]/*Telephone.

RefID:1058. Dennis, Michael, Godley, Susan H., Diamond, Guy, Tims, Frank M., Babor, Thomas, Donaldson, Jean, Liddle, Howard, Titus, Janet C., Kaminer, Yifrah, Webb, Charles, Hamilton, Nancy, and Funk, Rod. The Cannabis Youth Treatment (CYT) Study: main findings from two randomized trials. Journal of substance abuse treatment 2004. 27 (3) 197-213. **Keywords:** Adolescent/Alcoholism/ec [Economics]/Alcoholism/rh [Rehabilitation]/*Ambulatory Care/ec [Economics]/Cognitive Therapy/ec [Economics]/Combined Modality Therapy/Family Therapy/ec [Economics]/Female/*Health Care Costs/sn [Statistics & Numerical Data]/Humans/Length of Stay/ec [Economics]/Male/Marijuana Abuse/ec [Economics]/*Marijuana Abuse/rh [Rehabilitation]/Motivation/Outcome and Process Assessment (Health Care)/sn [Statistics & Numerical Data]/*Psychotherapy,Brief/ec [Economics]/Randomized Controlled Trials as Topic/Reinforcement (Psychology)/Reinforcement,Social/United States.

RefID:1068. Doran, C. M., Shanahan, M., Bell, J., and Gibson, A.. A cost-effectiveness analysis of buprenorphine-assisted heroin withdrawal. Drug and alcohol review 2004. 23 (2) 171-175. **Keywords:** Adolescent/Adult/Aged/Buprenorphine/ad [Administration & Dosage]/*Buprenorphine/ec [Economics]/*Buprenorphine/tu [Therapeutic Use]/Community Mental Health Services/ec [Economics]/Cost-Benefit Analysis/Female/*Heroin/ae [Adverse Effects]/Humans/Male/Metabolic Detoxication,Drug/Middle Aged/Narcotic Antagonists/ad [Administration & Dosage]/*Narcotic Antagonists/ec [Economics]/*Narcotic Antagonists/tu [Therapeutic Use]/*Substance Withdrawal Syndrome/dt [Drug Therapy]/*Substance Withdrawal Syndrome/et [Etiology]/Urban Population/sn [Statistics & Numerical Data].

RefID:1069. Kavanagh, David J., Young, Ross, White, Angela, Saunders, John B., Wallis, Jeff, Shockley, Natalie, Jenner, Linda, and Clair, Anne. A brief motivational intervention for substance misuse in recent-onset psychosis. Drug and alcohol review 2004. 23 (2) 151-155. **Keywords:** Adolescent/Adult/Brief Psychiatric Rating Scale/Diagnostic and Statistical Manual of Mental Disorders/Female/Humans/Interviews as Topic/Male/*Motivation/Pilot Projects/*Psychotherapy,Brief/mt [Methods]/*Psychotic Disorders/et [Etiology]/Schizophrenia/et [Etiology]/Single-Blind Method/*Substance-Related Disorders/co [Complications]/Substance-Related Disorders/di [Diagnosis]/*Substance-Related Disorders/rh

[Rehabilitation]/Time Factors.

RefID:1091. Mullins, Sharon M., Suarez, Mariann, Ondersma, Steven J., and Page, Melanie C.. The impact of motivational interviewing on substance abuse treatment retention: a randomized control trial of women involved with child welfare. Journal of substance abuse treatment 2004. 27 (1) 51-58. **Keywords:** Adolescent/Adult/Analysis of Variance/Child/Child Welfare/Female/Humans/*Interview,Psychological/*Mandatory Programs/*Motivation/*Patient Compliance/Pregnancy/Pregnancy Complications/rh [Rehabilitation]/*Substance-Related Disorders/rh [Rehabilitation]/United States.

RefID:1108. Sweeney, Luann P., Samet, Jeffrey H., Larson, Mary Jo, and Saitz, Richard. Establishment of a multidisciplinary Health Evaluation and Linkage to Primary care (HELP) clinic in a detoxification unit. Journal of addictive diseases 2004. 23 (2) 33-45. **Keywords:** Adult/Appointments and Schedules/*Continuity of Patient Care/sn [Statistics & Numerical Data]/Counseling/ut [Utilization]/Diagnostic Tests,Routine/ut [Utilization]/Feasibility Studies/Female/Humans/Interprofessional Relations/Male/Middle Aged/Patient Compliance/sn [Statistics & Numerical Data]/*Primary Health Care/og [Organization & Administration]/Primary Health Care/ut [Utilization]/Referral and Consultation/ut [Utilization]/Reminder Systems/ut [Utilization]/Social Work/*Substance Abuse Treatment Centers/og [Organization & Administration]/Substance-Related Disorders/eh [Ethnology]/*Substance-Related Disorders/rh [Rehabilitation]/Urban Health Services/og [Organization & Administration].

RefID:1174. Higgins, Stephen T., Sigmon, Stacey C., Wong, Conrad J., Heil, Sarah H., Badger, Gary J., Donham, Robert, Dantona, Robert L., and Anthony, Stacey. Community reinforcement therapy for cocaine-dependent outpatients. Archives of general psychiatry 2003. 60 (10) 1043-1052. **Keywords:** Adult/*Ambulatory Care/*Behavior Therapy/mt [Methods]/Cocaine-Related Disorders/px [Psychology]/Cocaine-Related Disorders/rh [Rehabilitation]/*Cocaine-Related Disorders/th [Therapy]/Female/Follow-Up Studies/Humans/Male/Patient Compliance/*Reinforcement (Psychology)/Token Economy/Treatment Outcome.

RefID:1185. Sinha, Rajita, Easton, Caroline, Renee-Aubin, Lisa, and Carroll, Kathleen M.. Engaging young probation-referred marijuana-abusing individuals in treatment: a pilot trial. The American journal on addictions / American Academy of Psychiatrists in Alcoholism and Addictions 2003. 12 (4) 314-323. **Keywords:** Adolescent/Adult/Ambulatory Care/*Behavior Therapy/Combined Modality Therapy/Female/Humans/Male/Marijuana Abuse/px [Psychology]/*Marijuana Abuse/rh [Rehabilitation]/*Motivation/*Patient Acceptance of Health Care/px [Psychology]/*Prisoners/px [Psychology]/*Psychotherapy,Brief/*Referral and Consultation/Substance Abuse Treatment Centers/Treatment Outcome.

RefID:1192. Miller, William R., Yahne, Carolina E., and Tonigan, J. Scott. Motivational interviewing in drug abuse services: a randomized trial. Journal of consulting and clinical psychology 2003. 71 (4) 754-763. **Keywords:** Adult/Female/Humans/*Interview,Psychological/Male/*Mental Health Services/st [Standards]/*Motivation/Self Disclosure/*Substance-Related Disorders/di

[Diagnosis]/*Substance-Related Disorders/th [Therapy].

RefID:1262. Baker, Amanda, Lewin, Terry, Reichler, Heidi, Clancy, Richard, Carr, Vaughan, Garrett, Rachel, Sly, Ketrina, Devir, Holly, and Terry, Margarett. Evaluation of a motivational interview for substance use within psychiatric in-patient services. Addiction (Abingdon, England) 2002. 97 (10) 1329-1337. **Keywords:** Adult/Female/Follow-Up Studies/Hospitalization/Hospitals,Psychiatric/Humans/*Interview,Psychological/mt [Methods]/Male/Mental Disorders/co [Complications]/Middle Aged/*Motivation/Single-Blind Method/Substance-Related Disorders/co [Complications]/*Substance-Related Disorders/rh [Rehabilitation].

RefID:1275. Baker, Amanda, Lewin, Terry, Reichler, Heidi, Clancy, Richard, Carr, Vaughan, Garrett, Rachel, Sly, Ketrina, Devir, Holly, and Terry, Margarett. Motivational interviewing among psychiatric in-patients with substance use disorders. Acta psychiatrica Scandinavica 2002. 106 (3) 233-240. **Keywords:** Adolescent/Adult/Aged/Diagnosis,Dual (Psychiatry)/Feasibility Studies/Female/Humans/Inpatients/*Interview,Psychological/mt [Methods]/Interview,Psychological/st [Standards]/Male/*Mental Disorders/di [Diagnosis]/Middle Aged/Mood Disorders/di [Diagnosis]/*Motivation/Patient Acceptance of Health Care/Schizophrenia/di [Diagnosis]/*Substance-Related Disorders/di [Diagnosis]/Substance-Related Disorders/px [Psychology]/Substance-Related Disorders/th [Therapy].

RefID:1306. Scott, Christy K., Sherman, Richard E., Foss, Mark A., Godley, Mark, and Hristova, Lilia. Impact of centralized intake on case management services. Journal of psychoactive drugs 2002. 34 (1) 51-57. **Keywords:** Adult/*Case Management/og [Organization & Administration]/Chicago/Community Networks/Documentation/Eligibility Determination/Female/Humans/Male/*Patient Admission/Patient Dropouts/Psychometrics/*Substance Abuse Treatment Centers/og [Organization & Administration]/Substance-Related Disorders/px [Psychology]/*Substance-Related Disorders/rh [Rehabilitation]/Treatment Outcome.

RefID:1353. Preston, K. L., Umbricht, A., Wong, C. J., and Epstein, D. H.. Shaping cocaine abstinence by successive approximation. Journal of consulting and clinical psychology 2001. 69 (4) 643-654. **Keywords:** Adult/*Behavior Therapy/*Cocaine-Related Disorders/rh [Rehabilitation]/Female/Humans/Male/Methadone/tu [Therapeutic Use]/Middle Aged/*Motivation/Opioid-Related Disorders/rh [Rehabilitation]/Reinforcement Schedule/Substance Abuse Detection/Token Economy.

RefID:1435. Rohsenow, D. J., Monti, P. M., Martin, R. A., Michalec, E., and Abrams, D. B.. Brief coping skills treatment for cocaine abuse: 12-month substance use outcomes. Journal of consulting and clinical psychology 2000. 68 (3) 515-520. **Keywords:** *Adaptation,Psychological/Adult/*Behavior Therapy/mt [Methods]/*Cocaine-Related Disorders/pc [Prevention & Control]/*Cocaine-Related Disorders/rh [Rehabilitation]/Female/Follow-Up Studies/Humans/Male/Psychotherapy,Brief/mt

[Methods]/Recurrence/Relaxation Therapy/*Stress,Psychological/px [Psychology]/Time Factors/Treatment Outcome.

RefID:1437. Schumacher, J. E., Usdan, S., Milby, J. B., Wallace, D., and McNamara, C.. Abstinent-contingent housing and treatment retention among crack-cocaine-dependent homeless persons. Journal of substance abuse treatment 2000. 19 (1) 81-88. **Keywords:** Adult/Alabama/*Cocaine-Related Disorders/rh [Rehabilitation]/Cocaine-Related Disorders/th [Therapy]/*Crack Cocaine/Female/*Homeless Persons/px [Psychology]/Homeless Persons/sn [Statistics & Numerical Data]/*Housing/Humans/Male/Middle Aged/*Socioenvironmental Therapy/mt [Methods]/Treatment Outcome.

RefID:1474. Messina, N. P., Wish, E. D., and Nemes, S.. Therapeutic community treatment for substance abusers with antisocial personality disorder. Journal of substance abuse treatment 1999. 17 (1-2) 121-128. **Keywords:** Adolescent/Adult/Antisocial Personality Disorder/di [Diagnosis]/*Antisocial Personality Disorder/th [Therapy]/Diagnosis,Dual (Psychiatry)/Follow-Up Studies/Humans/Male/Patient Compliance/Recurrence/Substance-Related Disorders/di [Diagnosis]/*Substance-Related Disorders/th [Therapy]/*Therapeutic Community/Treatment Outcome.

RefID:1499. Avants, S. K., Margolin, A., Sindelar, J. L., Rounsaville, B. J., Schottenfeld, R., Stine, S., Cooney, N. L., Rosenheck, R. A., Li, S. H., and Kosten, T. R.. Day treatment versus enhanced standard methadone services for opioid-dependent patients: a comparison of clinical efficacy and cost. The American journal of psychiatry 1999. 156 (1) 27-33. **Keywords:** Adult/Cocaine-Related Disorders/ec [Economics]/Cocaine-Related Disorders/ep [Epidemiology]/Cocaine-Related Disorders/th [Therapy]/*Cost-Benefit Analysis/Day Care/ec [Economics]/*Day Care/Female/Follow-Up Studies/HIV Infections/ep [Epidemiology]/Health Care Costs/Humans/Male/Methadone/ec [Economics]/*Methadone/tu [Therapeutic Use]/Opioid-Related Disorders/ec [Economics]/Opioid-Related Disorders/ep [Epidemiology]/*Opioid-Related Disorders/rh [Rehabilitation]/Outcome Assessment (Health Care)/Risk-Taking/Severity of Illness Index/Substance Abuse Detection/Substance Abuse Treatment Centers/ec [Economics]/*Substance Abuse Treatment Centers/Treatment Outcome.

RefID:1515. Nemes, S., Wish, E. D., and Messina, N.. Comparing the impact of standard and abbreviated treatment in a therapeutic community. Findings from the district of Columbia treatment initiative experiment. Journal of substance abuse treatment 1999. 17 (4) 339-347. **Keywords:** Adult/Ambulatory Care/District of Columbia/Female/Humans/*Length of Stay/Male/Middle Aged/Rehabilitation,Vocational/Substance Abuse Detection/*Substance-Related Disorders/rh [Rehabilitation]/*Therapeutic Community/Treatment Outcome.

RefID:1533. Kaminer, Y., Burleson, J. A., Blitz, C., Sussman, J., and Rounsaville, B. J.. Psychotherapies for adolescent substance abusers: a pilot study. The Journal of nervous and mental disease 1998. 186 (11) 684-690. **Keywords:** Adolescent/Age Factors/*Cognitive Therapy/Diagnosis,Dual (Psychiatry)/Female/Humans/Male/Mental Disorders/di [Diagnosis]/*Mental Disorders/th [Therapy]/Pilot

Projects/*Psychotherapy/Psychotherapy,Group/Regression Analysis/Sex Factors/Substance Abuse Detection/Substance-Related Disorders/di [Diagnosis]/*Substance-Related Disorders/th [Therapy]/Treatment Outcome.

RefID:1574. McCusker, J., Bigelow, C., Vickers-Lahti, M., Spotts, D., Garfield, F., and Frost, R.. Planned duration of residential drug abuse treatment: efficacy versus effectiveness. Addiction (Abingdon, England) 1997. 92 (11) 1467-1478. **Keywords:** Adult/Female/Follow-Up Studies/Humans/Length of Stay/Male/Patient Care Planning/Recurrence/*Residential Treatment/*Substance-Related Disorders/th [Therapy]/Treatment Outcome/United States.

RefID:1575. Nuttbrock, L. H., Ng-Mak, D. S., Rahav, M., and Rivera, J. J.. Pre- and post-admission attrition of homeless, mentally ill chemical abusers referred to residential treatment programs. Addiction (Abingdon, England) 1997. 92 (10) 1305-1315. **Keywords:** Adult/*Community Mental Health Centers/sn [Statistics & Numerical Data]/Diagnosis,Dual (Psychiatry)/*Homeless Persons/sn [Statistics & Numerical Data]/Humans/Male/*Mental Disorders/th [Therapy]/New York City/*Patient Dropouts/sn [Statistics & Numerical Data]/*Residential Treatment/sn [Statistics & Numerical Data]/Substance-Related Disorders/px [Psychology]/*Substance-Related Disorders/th [Therapy]/Treatment Refusal.

RefID:1578. Simpson, D. D., Joe, G. W., Rowan-Szal, G. A., and Greener, J. M.. Drug abuse treatment process components that improve retention. Journal of substance abuse treatment 1997. 14 (6) 565-572. **Keywords:** Humans/Patient Acceptance of Health Care/Patient Compliance/*Substance-Related Disorders/th [Therapy].

RefID:1600. Gottheil, E., Thornton, C. C., and Weinstein, S. P.. Treatment structure, client coping methods, and response to brief individual counseling: preliminary findings in a substance dependent sample. Journal of addictive diseases 1997. 16 (3) 51-65. **Keywords:** *Adaptation,Psychological/Adult/Cognitive Therapy/*Counseling/mt [Methods]/Female/Field Dependence-Independence/Humans/Male/Middle Aged/Personality Inventory/Psychiatric Status Rating Scales/Questionnaires/Severity of Illness Index/Social Adjustment/Substance-Related Disorders/px [Psychology]/*Substance-Related Disorders/rh [Rehabilitation]/Treatment Outcome.

RefID:1602. Schmitz, J. M., Oswald, L. M., Jacks, S. D., Rustin, T., Rhoades, H. M., and Grabowski, J.. Relapse prevention treatment for cocaine dependence: group vs. individual format. Addictive behaviors 1997. 22 (3) 405-418. **Keywords:** Adaptation,Psychological/Adult/*Cocaine/*Cognitive Therapy/Female/Humans/Internal-External Control/Male/Middle Aged/Motivation/Personality Inventory/*Psychotherapy,Group/Recurrence/Substance Withdrawal Syndrome/px [Psychology]/Substance Withdrawal Syndrome/rh [Rehabilitation]/Substance-Related Disorders/px [Psychology]/*Substance-Related Disorders/rh [Rehabilitation].

RefID:1633. Fals-Stewart, W., Birchler, G. R., and O'Farrell, T. J.. Behavioral couples therapy for male substance-abusing patients: effects on relationship adjustment and drug-using behavior.

Journal of consulting and clinical psychology 1996. 64 (5) 959-972. **Keywords:** *Adaptation,Psychological/Adult/Alcoholism/px [Psychology]/Alcoholism/rh [Rehabilitation]/Ambulatory Care/*Behavior Therapy/mt [Methods]/Female/Humans/Male/*Marital Therapy/mt [Methods]/*Marriage/px [Psychology]/Middle Aged/*Psychotropic Drugs/*Street Drugs/Substance-Related Disorders/px [Psychology]/*Substance-Related Disorders/rh [Rehabilitation]/Treatment Outcome.

RefID:1636. Booth, R. E., Crowley, T. J., and Zhang, Y.. Substance abuse treatment entry, retention and effectiveness: out-of-treatment opiate injection drug users. Drug and alcohol dependence 1996. 42 (1) 11-20. **Keywords:** Adult/Ambulatory Care/Crime/pc [Prevention & Control]/Crime/px [Psychology]/Female/HIV Infections/pc [Prevention & Control]/HIV Infections/px [Psychology]/HIV Infections/tm [Transmission]/Humans/Male/Middle Aged/Opioid-Related Disorders/px [Psychology]/*Opioid-Related Disorders/rh [Rehabilitation]/*Outcome and Process Assessment (Health Care)/Patient Care Team/Patient Dropouts/px [Psychology]/Patient Education as Topic/Prospective Studies/Substance Abuse Detection/Substance Abuse,Intravenous/px [Psychology]/*Substance Abuse,Intravenous/rh [Rehabilitation].

RefID:1661. Stahler, G. J., Shipley, T. F. J., Bartelt, D., DuCette, J. P., and Shandler, I. W.. Evaluating alternative treatments for homeless substance-abusing men: outcomes and predictors of success. Journal of addictive diseases 1995. 14 (4) 151-167. **Keywords:** Adult/Aftercare/*Alcoholism/rh [Rehabilitation]/*Case Management/*Cocaine/Community Mental Health Services/*Homeless Persons/px [Psychology]/Humans/Male/*Patient Admission/Patient Care Team/Rehabilitation Centers/Rehabilitation,Vocational/px [Psychology]/Social Support/Substance-Related Disorders/px [Psychology]/*Substance-Related Disorders/rh [Rehabilitation]/Treatment Outcome.

RefID:1678. McCusker, J., Vickers-Lahti, M., Stoddard, A., Hindin, R., Bigelow, C., Zorn, M., Garfield, F., Frost, R., Love, C., and Lewis, B.. The effectiveness of alternative planned durations of residential drug abuse treatment. American journal of public health 1995. 85 (10) 1426-1429. **Keywords:** Follow-Up Studies/Humans/Length of Stay/New England/*Outcome Assessment (Health Care)/Patient Education as Topic/Program Evaluation/Recurrence/*Substance Abuse Treatment Centers/sn [Statistics & Numerical Data]/*Substance-Related Disorders/th [Therapy]/*Therapeutic Community/Time Factors.

RefID:1685. Vickers-Lahti, M., Garfield, F., McCusker, J., Hindin, R., Bigelow, C., Love, C., and Lewis, B.. The relationship between legal factors and attrition from a residential drug abuse treatment program. Journal of psychoactive drugs 1995. 27 (1) 17-25. **Keywords:** Adult/Crime/Depression/px [Psychology]/Female/Humans/Male/Multivariate Analysis/Patient Dropouts/px [Psychology]/*Patient Dropouts/sn [Statistics & Numerical Data]/Psychiatric Status Rating Scales/*Residential Treatment/lj [Legislation & Jurisprudence]/Residential Treatment/sn [Statistics & Numerical Data]/Socioeconomic Factors/Substance-Related Disorders/px [Psychology]/*Substance-Related Disorders/rh [Rehabilitation]/Treatment Outcome.

RefID:1693. Saunders, B., Wilkinson, C., and Phillips, M.. The impact of a brief motivational intervention with opiate users attending a methadone programme. Addiction (Abingdon, England) 1995. 90 (3) 415-424. **Keywords:** Adolescent/Adult/Combined Modality Therapy/Female/Follow-Up Studies/Heroin Dependence/px [Psychology]/*Heroin Dependence/rh [Rehabilitation]/Humans/Internal-External Control/Male/*Methadone/tu [Therapeutic Use]/Middle Aged/*Motivation/Patient Dropouts/px [Psychology]/*Psychotherapy,Brief/Self Concept/Treatment Outcome.

RefID:1706. Kidorf, M., Stitzer, M. L., Brooner, R. K., and Goldberg, J.. Contingent methadone take-home doses reinforce adjunct therapy attendance of methadone maintenance patients. Drug and alcohol dependence 1994. 36 (3) 221-226. **Keywords:** Adult/Combined Modality Therapy/Female/Follow-Up Studies/Heroin Dependence/px [Psychology]/*Heroin Dependence/rh [Rehabilitation]/Humans/Male/*Methadone/ad [Administration & Dosage]/Motivation/*Patient Compliance/px [Psychology]/*Psychotherapy/Reinforcement Schedule/Self Administration/px [Psychology]/Substance Abuse Detection/*Token Economy.

RefID:1719. Carroll, K. M., Rounsaville, B. J., Nich, C., Gordon, L. T., Wirtz, P. W., and Gawin, F.. One-year follow-up of psychotherapy and pharmacotherapy for cocaine dependence. Delayed emergence of psychotherapy effects. Archives of general psychiatry 1994. 51 (12) 989-997. **Keywords:** Adaptation,Psychological/Adult/Ambulatory Care/*Behavior Therapy/*Cocaine/Cognitive Therapy/*Desipramine/tu [Therapeutic Use]/Double-Blind Method/Female/Follow-Up Studies/Humans/Male/Placebos/Psychotherapy,Brief/Recurrence/Substance-Related Disorders/dt [Drug Therapy]/Substance-Related Disorders/pc [Prevention & Control]/*Substance-Related Disorders/th [Therapy]/Treatment Outcome.

RefID:1727. Hall, S. M., Tunis, S., Triffleman, E., Banys, P., Clark, H. W., Tusel, D., Stewart, P., and Presti, D.. Continuity of care and desipramine in primary cocaine abusers. The Journal of nervous and mental disease 1994. 182 (10) 570-575. **Keywords:** Adult/Ambulatory Care/*Cocaine/Continuity of Patient Care/ec [Economics]/*Continuity of Patient Care/Costs and Cost Analysis/Counseling/Desipramine/bl [Blood]/*Desipramine/tu [Therapeutic Use]/Female/Hospitalization/Humans/Length of Stay/Male/Patient Compliance/Placebos/Probability/Process Assessment (Health Care)/Psychotherapy/ec [Economics]/*Psychotherapy/mt [Methods]/Psychotherapy,Group/mt [Methods]/*Substance-Related Disorders/dt [Drug Therapy]/Substance-Related Disorders/rh [Rehabilitation]/Treatment Outcome.

RefID:1783. Lerner, A., Sigal, M., Bacalu, A., and Gelkopf, M.. Short term versus long term psychotherapy in opioid dependence: a pilot study. The Israel journal of psychiatry and related sciences 1992. 29 (2) 114-119. **Keywords:** Adult/Behavior Therapy/Counseling/Humans/Male/*Opioid-Related Disorders/th [Therapy]/Pilot Projects/*Psychotherapy/Time Factors.

RefID:1791. McCusker, J., Stoddard, A. M., Zapka, J. G., Morrison, C. S., Zorn, M., and Lewis, B. F.. AIDS education for drug abusers: evaluation of short-term effectiveness. American journal of public health 1992. 82 (4) 533-540. **Keywords:** Acquired Immunodeficiency Syndrome/ep [Epidemiology]/*Acquired Immunodeficiency Syndrome/pc [Prevention & Control]/Bias (Epidemiology)/Counseling/st [Standards]/Female/Follow-Up Studies/*Health Knowledge,Attitudes,Practice/Health Services Research/Humans/Male/Models,Psychological/Outcome Assessment (Health Care)/Patient Dropouts/Patient Education as Topic/mt [Methods]/*Patient Education as Topic/st [Standards]/Risk Factors/Substance Abuse,Intravenous/co [Complications]/*Substance Abuse,Intravenous/px [Psychology]/Substance Abuse,Intravenous/rh [Rehabilitation]/Time Factors.

RefID:1857. Hawton, K., McKeown, S., Day, A., Martin, P., O'Connor, M., and Yule, J.. Evaluation of out-patient counselling compared with general practitioner care following overdoses. Psychological medicine 1987. 17 (3) 751-761. **Keywords:** Adult/Aftercare/*Ambulatory Care/Attitude to Health/*Counseling/Female/Humans/Male/Outcome and Process Assessment (Health Care)/*Physicians,Family/*Poisoning/px [Psychology]/Prospective Studies/Sex Factors/*Substance-Related Disorders/th [Therapy]/*Suicide,Attempted/px [Psychology].

RefID:1912. Bale, R. N., Van Stone, W. W., Kuldau, J. M., Engelsing, T. M., Elashoff, R. M., and Zarcone, V. P. J.. Therapeutic communities vs methadone maintenance. A prospective controlled study of narcotic addiction treatment: design and one-year follow-up. Archives of general psychiatry 1980. 37 (2) 179-193. **Keywords:** Adult/Crime/Female/Follow-Up Studies/Heroin Dependence/px [Psychology]/*Heroin Dependence/rh [Rehabilitation]/Humans/Male/*Methadone/tu [Therapeutic Use]/Patient Acceptance of Health Care/Social Adjustment/*Therapeutic Community.

RefID:1937. Petry, N. M., Alessi, S. M., and Ledgerwood, D. M.. Contingency management delivered by community therapists in outpatient settings. Drug and alcohol dependence 2012. 122 (1-2) 86-92. **Keywords:** Adult/article/*Behavior Therapy/breath analysis/*clinical effectiveness/*community care/*contigency management/controlled study/*drug dependence/di [Diagnosis]/*drug dependence/th [Therapy]/drug withdrawal/*health care personnel/health program/human/intermethod comparison/major clinical study/medical education/outcome assessment/*outpatient care/Patient Compliance/Patient Participation/priority journal/Professional Competence/randomized controlled trial/Reward/Treatment Outcome/Urinalysis.

RefID:1971. Tetrault, J., Moore, B., Barry, D., O'Connor, P., Schottenfeld, R., Fiellin, D., and Sullivan, L.. Brief versus extended counseling for HIV clinic based buprenorphine treatment of opioid dependence. Journal of general internal medicine 2011. 26 (#Issue#) S133-S134. **Keywords:** *society/*Human immunodeficiency virus/*Counseling/*hospital/*Internal Medicine/human/virus load/abstinence/urine/clinical trial/Analysis of Variance/patient/population/drug therapy/Human immunodeficiency virus infected patient/physician/Disease Management/book/nurse/patient counseling/toxicology/log rank

test/outcome variable/Student t test/model/*opiate/*buprenorphine/CD4 antigen.

RefID:2330. D'Agostino, C. S., Barry, K. L., Blow, F. C., and Podgorski, C.. Community interventions for older adults with comorbid substance abuse: The Geriatric Addictions Program (GAP). Journal of Dual Diagnosis 2006. 2 (3) 31-45. **Keywords:** Adult/Aged/*Alcoholism/th [Therapy]/article/Comorbidity/conservative treatment/controlled study/*drug abuse/Female/Geriatric Assessment/geriatric care/health program/hospital discharge/hospital patient/human/major clinical study/Male/Mental Health/prescription/psychiatric diagnosis/substance abuse.

RefID:2562. Gibson, D. R., Lovelle-Drache, J., Young, M., Hudes, E. S., and Sorensen, J. L.. Effectiveness of brief counseling in reducing HIV risk behavior in injecting drug users: Final results of randomized trials of counseling with and without HIV testing. AIDS and behavior 1999. 3 (1) 3-12. **Keywords:** Adult/article/clinical trial/controlled clinical trial/controlled study/Counseling/drug detoxification/drug self administration/Female/follow up/Health Promotion/*heroin dependence/human/*Human immunodeficiency virus infection/injection/interview/major clinical study/Male/randomized controlled trial/Risk Assessment/screening test/*diamorphine/*Methadone.

RefID:2592. Mejta, C. L., Bokos, P. J., Mickenberg, J., Maslar, M. E., and Senay, E.. Improving substance abuse treatment access and retention using a case management approach. Journal of Drug Issues 1997. 27 (2) 329-340. **Keywords:** article/clinical trial/controlled clinical trial/controlled study/*drug dependence treatment/*health care access/health care facility/human/intravenous drug abuse/major clinical study/*opiate addiction/patient information/patient referral/randomized controlled trial/*substance abuse.

RefID:2704. Petry, Nancy M., Barry, Danielle, Alessi, Sheila M., Rounsaville, Bruce J., and Carroll, Kathleen M.. A randomized trial adapting contingency management targets based on initial abstinence status of cocaine-dependent patients. Journal of consulting and clinical psychology 2012. 80 (2) 276-285. **Keywords:** *Cocaine/*contingency management/*Drug Dependency/*Drug Rehabilitation/*Treatment Effectiveness Evaluation/Drug Abstinence/Outpatient Treatment.

RefID:2714. Sheidow, Ashli J., Jayawardhana, Jayani, Bradford, W. David, Henggeler, Scott W., and Shapiro, Steven B.. Money matters: Cost-effectiveness of juvenile drug court with and without evidence-based treatments. Journal of Child & Adolescent Substance Abuse 2012. 21 (1) 69-90. **Keywords:** *Adjudication/*Costs and Cost Analysis/*evidence based practice/*juvenile delinquency.

RefID:2727. Gauthier, Gail, Palacios-Boix, Jorge, Charney, Dara A., Negrete, Juan C., Pentney, Helen, and Gill, Kathryn J.. Comparison of brief and standard interventions for drug and alcohol dependence: Considerations for primary care service delivery. Canadian Journal of Community Mental Health 2011. 30 (1) 93-104. **Keywords:** *Alcoholism/*Drug Dependency/*health care delivery/*Intervention/*Primary Health Care.

RefID:2735. Garcia-Fernandez, Gloria, Secades-Villa, Roberto, Garcia-Rodriguez, Olaya, Alvarez-Lopez, Heli, Fernandez-Hermida, Jose R., Fernandez-Artamendi, Sergio, and Higgins, Stephen T.. Long-term benefits of adding incentives to the community reinforcement approach for cocaine dependence. European Addiction Research 2011. 17 (3) 139-145. **Keywords:** *Cocaine/*Drug Dependency/*Drug Rehabilitation/*Incentives/*reinforcement/Contingency Management/Drug Abstinence/Treatment Outcomes.

RefID:2745. Phan, Olivier, Henderson, Craig E., Angelidis, Tatiana, Weil, Patricia, van Toorn, Manja, Rigter, Renske, Soria, Cecilia, and Rigter, Henk. European youth care sites serve different populations of adolescents with cannabis use disorder. Baseline and referral data from the INCANT trial. BMC psychiatry 2011. 11 (#Issue#) #Start Page#-#End Page#. **Keywords:** *Cannabis/*Drug Addiction/*Family Therapy/*Outpatient Treatment/Demographic Characteristics/Morbidity.

RefID:2794. Rynes, Kristina N.. Demand-withdraw interaction in family therapy for adolescent drug abuse. Dissertation Abstracts International: Section B: The Sciences and Engineering 2011. 71 (11-B) 7103-#End Page#. **Keywords:** *Adolescent Attitudes/*drug abuse/*Family Therapy.

RefID:2825. Secades-Villa, Roberto, Garcia-Rodriguez, Olaya, Garcia-Fernandez, Gloria, Sanchez-Hervas, Emilio, Fernandez-Hermida, Jose R., and Higgins, Stephen T.. Community reinforcement approach plus vouchers among cocaine-dependent outpatients: Twelve-month outcomes. Psychology of Addictive Behaviors 2011. 25 (1) 174-179. **Keywords:** *Cocaine/*contingency management/*Drug Dependency/*Drug Rehabilitation/*Outpatient Treatment/Community Services/Treatment Outcomes.

RefID:2866. Fals-Stewart, William and Lam, Wendy K. K.. Brief behavioral couples therapy for drug abuse: A randomized clinical trial examining clinical efficacy and cost-effectiveness. Families, Systems, & Health 2008. 26 (4) 377-392. **Keywords:** *Costs and Cost Analysis/*Couples Therapy/*drug abuse/*Treatment Outcomes.

RefID:2891. Rigter, Henk, Pelc, Isidore, Tossmann, Peter, Phan, Olivier, Grichting, Esther, Hendriks, Vincent, and Rowe, Cindy. INCANT: A transnational randomized trial of Multidimensional Family Therapy versus treatment as usual for adolescents with cannabis use disorder. BMC psychiatry 2010. 10 (#Issue#) #Start Page#-#End Page#. **Keywords:** *Cannabis/*drug abuse/*Drug Dependency/*Drug Usage/*Family Therapy.

RefID:2965. Wesley, Martin C., Minatrea, Neresa B., and Watson, Joshua C.. Animal-assisted therapy in the treatment of substance dependence. Anthrozoos 2009. 22 (2) 137-148. **Keywords:** *Animal Assisted Therapy/*Drug Dependency/*Drug Rehabilitation/*Therapeutic Alliance.

RefID:2967. Ghee, Anna Cash, Johnson, Candace S., Burlew, Ann Kathleen, and Boiling, Lanny C.. Enhancing retention through a condensed trauma-integrated intervention for women with

chemical dependence. North American Journal of Psychology 2009. 11 (1) 157-172. **Keywords:** *Drug Dependency/*Intervention/*major depression/*Trauma/*Treatment Compliance.

RefID:3088. Anderson, Ann Warner. The effectiveness of group motivational interviewing for substance abusers with histories of childhood maltreatment. Dissertation Abstracts International: Section B: The Sciences and Engineering 2007. 68 (5-B) 3384-#End Page#. **Keywords:** *Child Abuse/*drug abuse/*Group Dynamics/*History/*Interviewing/Motivation.

RefID:3175. Barnett, Paul G., Masson, Carmen L., Sorensen, James L., Wong, Wynnie, and Hall, Sharon. Linking opioid-dependent hospital patients to drug treatment: Health care use and costs 6 months after randomization. Addiction (Abingdon, England) 2006. 101 (12) 1797-1804. **Keywords:** *Case Management/*Costs and Cost Analysis/*Drug Dependency/*Monetary Incentives/*Opiates/Drug Rehabilitation/drug therapy/Economics/evaluation.

RefID:3430. Hickman, Mark Ellsworth. The effects of personal feedback on alcohol intake in dually diagnosed clients: An empirical study of William R. Miller's motivational enhancement therapy. Dissertation Abstracts International: Section B: The Sciences and Engineering 1999. 60 (1-B) 0367-#End Page#. **Keywords:** *alcohol abuse/*Feedback/*Measurement/*Mental Disorders/*Motivation/Comorbidity.

RefID:3538. Barber, Jacques P., Luborsky, Lester, Crits-Christoph, Paul, Thase, Michael E., Weiss, Roger, Frank, Arlene, Onken, Lisa, and Gallop, Robert. Therapeutic alliance as a predictor of outcome in treatment of cocaine dependence. Psychotherapy Research 1999. 9 (1) 54-73. **Keywords:** *Cocaine/*Drug Usage/*major depression/*Psychotherapeutic Processes/*Therapeutic Alliance/Cognitive Therapy/Drug Dependency/Drug Rehabilitation/Psychotherapy/Treatment Outcomes.

RefID:3599. Longabaugh, Richard, Wirtz, Philip W., Beattie, Martha C., Noel, Nora, and Stout, Robert. Matching treatment focus to patient social investment and support: 18-month follow-up results. #journal name# 1997. #volume# (#Issue#) 602-628. **Keywords:** *Drug Abstinence/*Drug Addiction/*Drug Rehabilitation/*Therapeutic Processes/*Client Treatment Matching.

RefID:3639. Rowan-Szal, Grace, Joe, George W., Chatham, Lois R., and Simpson, D. Dwayne. A simple reinforcement system for methadone clients in a community-based treatment program. Journal of substance abuse treatment 1994. 11 (3) 217-223. **Keywords:** *Behavior Change/*contingency management/*Drug Addiction.

RefID:3667. Szapocznik, Jose, Santisteban, David, Rio, Arturo, and Perez-Vidal, Angel. Family Effectiveness Training: An intervention to prevent drug abuse and problem behaviors in Hispanic adolescents. Hispanic Journal of Behavioral Sciences 1989. 11 (1) 4-27. **Keywords:** *Behavior Problems/*drug abuse/*Family Therapy/*prevention/*Latinos/Latinas/At Risk Populations/Family Members/Followup Studies.

RefID:4329. Gibson, D. R., Wermuth, L., Lovelle-Drache, J., Ham, J., and Sorensen, J. L.. Brief counselling to reduce AIDS risk in intravenous drug users and their sexual partners: preliminary results. Counselling Psychology Quarterly 1989. 2 (#Issue#) 15-19. **Keywords:** Central.

RefID:4636. Hall, S. M., Bass, A., Hargreaves, W. A., and Loeb, P.. Contingency management and information feedback in outpatient heroin detoxifiction. Behavior Therapy 1979. 10 (#Issue#) 443-451. **Keywords:** Central.

RefID:4767. Kirby, K. C., Marlowe, D. B., Lamb, R. J., Husband, S. D., and Platt, J. J.. Cognitive behavioral cocaine treatment with and without contingency management. NIDA research monograph 1994. 153 (#Issue#) 346-#End Page#. **Keywords:** Central.

RefID:4928. Lehman, A. F., Herron, J. D., Schwartz, R. P., and Myers, C. P.. Rehabilitation for adults with severe mental illness and substance use disorders. A clinical trial. The Journal of nervous and mental disease 1993. 181 (2) 86-90. **Keywords:** Central/Adolescent/Community Mental Health Centers/Comorbidity/Counseling/Follow-Up Studies/Hospitalization/Mental Disorders [complications] [epidemiology] [rehabilitation]/Prevalence/Program Evaluation/Substance-Related Disorders [complications] [epidemiology] [rehabilitation]/Treatment Outcome/Adult/Female/Humans/Male.

RefID:5183. Guydish, J., Werdegar, D., Sorensen, J. L., Clark, W., and Acampora, A.. Drug abuse day treatment: a randomized clinical trial comparing day and residential treatment programs. Journal of consulting and clinical psychology 1998. 66 (2) 280-289. **Keywords:** Central/Day Care/Outcome and Process Assessment (Health Care)/Patient Admission/Prospective Studies/Substance-Related Disorders [psychology] [rehabilitation]/Therapeutic Community/Treatment Outcome/Adult/Female/Humans/Male.

RefID:5310. LaCour, F., Elk, R., Grabowski, J., Rhoades, H., Mackey, T., and Delclos, G.. Contingency management interventions in the treatment of cocaine-dependent patients infected with tubercolosis. NIDA research monograph 1997. 174 (#Issue#) 76-#End Page#. **Keywords:** Central.

RefID:5523. McCleary, P. M., Elk, R., Schmitz, J., Mangus, L., Rhoades, H., Grabowski, J., and Anders, R.. Prevention of relapse to cocaine use in post-partum cocainedependente women: contingency management interventions and cognitive-behavioral therapy compared to supportive counseling. Proceedings of the 58th Annual Scientific Meeting of the College on Problems of Drug Dependence; 1996 June; San Juan, Puerto Rico, USA 1996. #volume# (#Issue#) 262-#End Page#. **Keywords:** Central.

RefID:5566. Van, Der Hyde and Vincent, A. and And Others. Adatsa follow-up study of extended outpatient care: a comparison of 90 days versus 180 days of outpatient treatment for clients of washington state's alcoholism and drug addiction treatment and support act. Washington State Dept.of Social and Health Services, Olympia.Office of Research and Data Analysis. 1995. #volume# (#Issue#) 124-#End Page#. **Keywords:** Central/Alcohol-Abuse/Human-

Services/Program-Evaluation/Social-Services/State-Agencies/State-Aid/Alcoholism-/Drug-Addiction/Drug-Rehabilitation/Outcomes-of-Treatment/Substance-Abuse/Washington-.

RefID:5672. Alterman, A. I., McKay, J. R., Mulvaney, F. D., McLellan, A. T., and O'Brien. Thirteen month outcomes of day hospital versus inpatient treatments for cocaine dependence. NIDA research monograph 1995. 153 (#Issue#) 90-#End Page#. **Keywords:** Central.

RefID:5688. Dennis ML, Scott CK. A early Re-intervention (ERI) model for chronic substance abusers. Drug and alcohol dependence 2001. 63 Suppl 1 (#Issue#) 39-#End Page#. **Keywords:** Central.

RefID:5811. Petry, N.. Prize reinforcement contingency management for cocaine-abusing methadone patients. Drug and alcohol dependence 2001. 63 Suppl 1 (#Issue#) 122-#End Page#. **Keywords:** Central.

RefID:5914. Rowan-Szal GA, Simpson DD. Contingency management and relapse prevention training in a sample of cocaine-using methadone clients. NIDA research monograph 2000. 180 (#Issue#) 145-#End Page#. **Keywords:** Central.

RefID:6071. Guydish, J., Sorensen, J. L., Chan, M., Werdegar, D., Bostrom, A., and Acampora, A.. A randomized trial comparing day and residential drug abuse treatment: 18-month outcomes. Journal of consulting and clinical psychology 1999. 67 (3) 428-434. **Keywords:** Central/Analysis of Variance/Chi-Square Distribution/Day Care [standards]/Follow-Up Studies/Residential Treatment [standards]/Substance-Related Disorders [therapy]/Survival Analysis/Therapeutic Community/Treatment Outcome/Adult/Female/Humans/Male.

RefID:6075. PeñA, J. M., Franklin, R. R., Rice, J. C., Foulks, E. F., Bland, I. J., Shervington, D., and James, A.. A two-rate hypothesis for patterns of retention in psychosocial treatments of cocaine dependence: findings from a study of African-American men and a review of the published data. The American journal on addictions / American Academy of Psychiatrists in Alcoholism and Addictions 1999. 8 (4) 319-331. **Keywords:** Central/Adolescent/African Americans [psychology] [statistics & numerical data]/Cocaine-Related Disorders [ethnology] [psychology] [rehabilitation]/Patient Dropouts [psychology] [statistics & numerical data]/Psychotherapy [methods]/Survival Analysis/Adult/Humans/Male/Middle Aged.

RefID:6093. Rees, V., Copeland, J., Swift, W., Roffman, R., and Stephens, R.. Brief cognitive behavioral interventions for cannabis dependence. NIDA research monograph 1999. 179 (#Issue#) 79-#End Page#. **Keywords:** Central.

RefID:6114. McKay, J. R., Alterman, A. I., Cacciola, J. S., O'Brien, C. P., Koppenhaver, J. M., and Shepard, D. S.. Continuing care for cocaine dependence: comprehensive 2-year outcomes. Journal of consulting and clinical psychology 1999. 67 (3) 420-427. **Keywords:** Central/Aftercare [standards]/Cocaine-Related Disorders [therapy]/Longitudinal Studies/Psychotherapy [methods] [standards]/Recurrence [prevention & control]/Regression Analysis/Treatment

Outcome/Adult/Humans/Male.

RefID:6203. Rawson, R. A.. Relapse Prevention and Contingency Management of Cocaine Abuse in Methadone Patients. NIDA research monograph 1999. #volume# (#Issue#) 25-#End Page#. **Keywords:** Central.

RefID:7057. Schottenfeld, R. S., Moore, B., and Pantalon, M. V.. Contingency management with community reinforcement approach or twelve-step facilitation drug counseling for cocaine dependent pregnant women or women with young children. Drug & Alcohol Dependence 1-10-2011. 118 (1) 48-55. **Keywords:** CINAHL/Alcoholism -- Epidemiology/Behavior Therapy -- Methods/Substance Use Disorders -- Psychosocial Factors/Substance Use Disorders -- Rehabilitation/Depression -- Epidemiology/Pregnancy Complications -- Rehabilitation/Support Groups/Adult/Alcoholism -- Psychosocial Factors/Ambulatory Care/Central Nervous System Stimulants -- Urine/Cocaine -- Urine/Substance Use Disorders -- Therapy/Comorbidity/Depression -- Psychosocial Factors/Female/human/Male/Maternal Behavior/Pregnancy/Pregnancy Complications -- Psychosocial Factors/Pregnancy Complications -- Therapy/Expectant Mothers -- Psychosocial Factors/Psychological Tests/Reinforcement (Psychology)/Residence Characteristics/Reward/Time Factors/Treatment Outcomes/Urban Population/Clinical Trials.

RefID:7204. Bisaga, A., Aharonovich, E., Cheng, W. Y., Levin, F. R., Mariani, J. J., Raby, W. N., and Nunes, E. V.. A placebo-controlled trial of memantine for cocaine dependence with high-value voucher incentives during a pre-randomization lead-in period. Drug & Alcohol Dependence 1-9-2010. 111 (1-2) 97-104. **Keywords:** CINAHL/Behavior Therapy/Memantine -- Therapeutic Use/Substance Use Disorders -- Therapy/Adult/Clinical Trials/Excitatory Amino Acid Antagonists -- Therapeutic Use/Female/human/Kaplan-Meier Estimator/Logistic Regression/Male/Middle Age/Randomized Controlled Trials/Sex Factors/Single-Blind Studies/Substance Abuse Detection/Treatment Outcomes.

RefID:7405. Drebing, C. E., Van Ormer, E. A., Mueller, L., Hebert, M., Penk, W. E., Petry, N. M., Rosenheck, R., and Rounsaville, B.. Adding contingency management intervention to vocational rehabilitation: outcomes for dually diagnosed veterans. Journal of Rehabilitation Research & Development 2007. 44 (6) 851-865. **Keywords:** CINAHL/Diagnosis,Dual (Psychiatry)/Job Re-Entry/Mental Disorders -- Rehabilitation/Motivation/Patient Compliance/Rehabilitation,Vocational/Substance Dependence -- Rehabilitation/Adult/Asians/Checklists/chi square test/Clinical Assessment Tools/Coefficient Alpha/Comparative Studies/Descriptive Statistics/Educational Status/Employment,Supported/Female/Hospitals,Veterans/Interviews/Job Application/Job Interviews/Male/Massachusetts/Middle Age/Native Americans/One-Tailed Test/outcome assessment/Prospective Studies/Psychiatric Patients/Questionnaires/Random Assignment/Salaries and Fringe Benefits/Self Report/Short Form-36 Health Survey (SF-36)/Substance Abuse Detection/Survival Analysis/T-Tests/Veterans/Whites/human.

RefID:7432. Petry, N. M., Alessi, S. M., Ledgerwood, D. M., and Sierra, S.. Psychometric

properties of the contingency management competence scale. Drug & Alcohol Dependence 1-6-2010. 109 (1-3) 167-174. **Keywords:** CINAHL/Psychological Tests/Psychometrics/Substance Use Disorders -- Psychosocial Factors/Substance Use Disorders -- Therapy/Adult/Clinical Competence/Community Health Services/Data Analysis,Statistical/Empathy/Factor Analysis/Female/human/Male/Middle Age/Observer Bias/Predictive Value of Tests/Reproducibility of Results/Substance Use Rehabilitation Programs/Substance Use Disorders -- Epidemiology/Treatment Outcomes/Clinical Trials.

RefID:7460. Hien, D. A., Jiang, H., Campbell, A. N., Hu, M. C., Miele, G. M., Cohen, L. R., Brigham, G. S., Capstick, C., Kulaga, A., Robinson, J., Suarez-Morales, L., and Nunes, E. V.. Do treatment improvements in PTSD severity affect substance use outcomes? A secondary analysis from a randomized clinical trial in NIDA's Clinical Trials Network. American Journal of Psychiatry 2010. 167 (1) 95-101. **Keywords:** CINAHL/Cognitive Therapy -- Methods/National Institute on Drug Abuse (U.S.)/Patient Education -- Methods/Stress Disorders,Post-Traumatic -- Therapy/Substance Use Disorders -- Therapy/Adult/Alcoholism -- Diagnosis/Alcoholism -- Epidemiology/Alcoholism -- Therapy/Comorbidity/Diagnosis,Dual (Psychiatry) -- Statistics and Numerical Data/Female/human/Life Change Events/Probability/Prospective Studies/Psychotherapy,Group -- Methods/secondary analysis/Severity of Illness Indices/Stress Disorders,Post-Traumatic -- Diagnosis/Stress Disorders,Post-Traumatic -- Epidemiology/Substance Use Disorders -- Diagnosis/Substance Use Disorders -- Epidemiology/Treatment Outcomes/United States.

RefID:7498. Penk, W., Drebing, C. E., Rosenheck, R. A., Krebs, C., Van Ormer, A., and Mueller, L.. Veterans Health Administration transitional work experience vs. job placement in veterans with co-morbid substance use and non-psychotic psychiatric disorders. Psychiatric rehabilitation journal 2010. 33 (4) 297-307. **Keywords:** CINAHL/Diagnosis,Dual (Psychiatry)/employment status/Rehabilitation,Vocational/Veterans -- Psychosocial Factors/Adult/Clinical Trials/Female/Funding Source/human/Interview Guides/Interviews/Male/Middle Age/Random Assignment/Salaries and Fringe Benefits/Time Factors.

RefID:7561. Defulio, A., Donlin, W. D., Wong, C. J., and Silverman, K.. Employment-based abstinence reinforcement as a maintenance intervention for the treatment of cocaine dependence: a randomized controlled trial. Addiction (Abingdon, England) 2009. 104 (9) 1530-1538. **Keywords:** CINAHL/Contingency Management/Employment/Rehabilitation,Vocational/Reinforcement (Psychology)/Substance Dependence -- Prevention and Control/Substance Dependence -- Rehabilitation/Randomized Controlled Trials/Cocaine/Confidence Intervals/Data Analysis Software/Descriptive Statistics/Fisher's Exact Test/Funding Source/HIV Infections/Interview Guides/Interviews/Mann-Whitney U Test/Maryland/Odds Ratio/Power Analysis/Questionnaires/Risk Assessment/Risk Taking Behavior/Self Report/Substance Abuse Detection -- Methods/Treatment Outcomes/Two-Tailed Test/Urinalysis/human.

RefID:7564. Rash, C. J., Olmstead, T. A., and Petry, N. M.. Income does not affect response to contingency management treatments among community substance abuse treatment-seekers.

Drug & Alcohol Dependence 1-10-2009. 104 (3) 249-253. **Keywords:** CINAHL/Behavior Therapy -- Economics/Income/Substance Use Disorders -- Economics/Ambulatory Care Facilities/Models,Psychological/Psychotherapy,Group -- Economics/Substance Use Disorders -- Therapy/Substance Use Rehabilitation Programs/Treatment Outcomes/human.

RefID:7582. Calsyn, D. A., Crits-Christoph, P., Hatch-Maillette, M. A., Doyle, S. R., Song, Y. S., Coyer, S., and Pelta, S.. Reducing sex under the influence of drugs or alcohol for patients in substance abuse treatment. Addiction (Abingdon, England) 2010. 105 (1) 100-108. **Keywords:** CINAHL/Alcoholic Intoxication/HIV Infections -- Prevention and Control/Substance Abusers -- Psychosocial Factors/Substance Use Disorders -- Therapy/Substance Use Rehabilitation Programs/Unsafe Sex -- Prevention and Control/Adult/Behavior Therapy/chi square test/Clinical Trials/Descriptive Statistics/effect size/Funding Source/Harm Reduction/HIV Education/human/Interview Guides/Logistic Regression/Male/Methadone -- Therapeutic Use/Middle Age/Odds Ratio/P-Value/Pretest-Posttest Design/Prospective Studies/Psychological Tests/Repeated Measures/Severity of Illness Indices/T-Tests.

RefID:7611. Field, C. A., Adinoff, B., Harris, T. R., Ball, S. A., and Carroll, K. M.. Construct, concurrent and predictive validity of the URICA: Data from two multi-site clinical trials. Drug & Alcohol Dependence 1-4-2009. 101 (1-2) 115-123. **Keywords:** CINAHL/Alcoholism -- Psychosocial Factors/Psychological Tests -- Standards/Substance Use Disorders -- Psychosocial Factors/Adult/Alcoholism -- Rehabilitation/Clinical Trials/Female/Male/Motivation/Predictive Value of Tests/Prognosis/Psychotherapy/Reproducibility of Results/Substance Use Disorders -- Rehabilitation/Treatment Outcomes/human.

RefID:7637. Epstein, D. H., Schmittner, J., Umbricht, A., Schroeder, J. R., Moolchan, E. T., and Preston, K. L.. Promoting abstinence from cocaine and heroin with a methadone dose increase and a novel contingency. Drug & Alcohol Dependence 1-4-2009. 101 (1-2) 92-100. **Keywords:** CINAHL/Health Promotion/Methadone -- Therapeutic Use/Narcotics -- Therapeutic Use/Substance Use Disorders -- Rehabilitation/Adult/Breath Tests/Counseling/Data Analysis,Statistical/Dose-Response Relationship,Drug/Double-Blind Studies/Female/Male/Methadone -- Administration and Dosage/Middle Age/Motivation/Narcotics -- Administration and Dosage/Psychological Tests/Quality of Life/Socioeconomic Factors/Substance Abuse Detection/Substance Use Disorders -- Psychosocial Factors/Substance Use Disorders -- Urine/Treatment Outcomes/human.

RefID:7643. Olmstead, T. A. and Petry, N. M.. The cost-effectiveness of prize-based and voucher-based contingency management in a population of cocaine- or opioid-dependent outpatients. Drug & Alcohol Dependence 1-6-2009. 102 (1-3) 108-115. **Keywords:** CINAHL/Motivation/Substance Use Disorders -- Rehabilitation/Ambulatory Care -- Economics/Clinical Trials/cost benefit analysis/Counseling -- Economics/Patient Education -- Economics/Sample Size/Substance Abuse Detection -- Economics/Substance Use Disorders -- Economics/Substance Use Disorders -- Psychosocial Factors/Substance Use Rehabilitation Programs/Treatment Outcomes/human.

RefID:7818. Rapp, R. C., Otto, A. L., Lane, D. T., Redko, C., McGatha, S., and Carlson, R. G.. Improving linkage with substance abuse treatment using brief case management and motivational interviewing. Drug & Alcohol Dependence 2008. 94 (1-3) 172-182. **Keywords:** CINAHL/Case Management -- Utilization/Motivational Interviewing -- Utilization/Substance Use Disorders -- Therapy/adolescence/Adult/chi square test/Clinical Trials/Descriptive Statistics/Female/Funding Source/Interviews/Logistic Regression/Male/Middle Age/Odds Ratio/Ohio/Pretest-Posttest Design/Questionnaires/Referral and Consultation/human.

RefID:7868. McKee, S. A., Carroll, K. M., Sinha, R., Robinson, J. E., Nich, C., Cavallo, D., and O'Malley, S.. Enhancing brief cognitive-behavioral therapy with motivational enhancement techniques in cocaine users. Drug & Alcohol Dependence 2-11-2007. 91 (1) 97-101. **Keywords:** CINAHL/Cognitive Therapy -- Methods/Motivation/Psychotherapy,Brief -- Methods/Substance Use Disorders -- Therapy/Adult/Demography/Female/Male/Treatment Outcomes.

RefID:7892. Olmstead, T. A., Sindelar, J. L., Easton, C. J., and Carroll, K. M.. The cost-effectiveness of four treatments for marijuana dependence. Addiction (Abingdon, England) 2007. 102 (9) 1443-1453. **Keywords:** CINAHL/Cognitive Therapy -- Methods/Counseling -- Methods/Motivation/Substance Use Disorders -- Rehabilitation/adolescence/Adult/Clinical Trials/Cognitive Therapy -- Economics/Connecticut/cost benefit analysis/Counseling -- Economics/Female/Male/Substance Use Disorders -- Economics/Treatment Outcomes/human.

RefID:8037. Morgenstern, J., Blanchard, K. A., Kahler, C., Barbosa, K. M., McCrady, B. S., and McVeigh, K. H.. Testing mechanisms of action for intensive case management. Addiction (Abingdon, England) 2008. 103 (3) 469-477. **Keywords:** CINAHL/Case Management/Substance Abuse -- Prevention and Control/Adult/Descriptive Statistics/Female/Funding Source/Interviews/Motivation/outcome assessment/Prospective Studies/Public Assistance/human.

RefID:8159. Roll, J. M., Petry, N. M., Stitzer, M. L., Brecht, M. L., Peirce, J. M., McCann, M. J., Blaine, J., MacDonald, M., DiMaria, J., Lucero, L., and Kellogg, S.. Contingency management for the treatment of methamphetamine use disorders. American Journal of Psychiatry 2006. 163 (11) 1993-1999. **Keywords:** CINAHL/Behavior Therapy -- Methods/Reward/Substance Use Disorders -- Therapy/Adult/Behavior,Addictive -- Psychosocial Factors/Behavior,Addictive -- Therapy/Behavior,Addictive -- Urine/chi square test/Clinical Trials/Comparative Studies/Confidence Intervals/Cox Proportional Hazards Model/Descriptive Statistics/Female/Funding Source/Male/Multicenter Studies/Odds Ratio/P-Value/Random Assignment/Reinforcement (Psychology)/Substance Abuse Detection/Substance Use Disorders -- Psychosocial Factors/Substance Use Disorders -- Urine/Substance Use Rehabilitation Programs/T-Tests/Treatment Outcomes/human.

RefID:8221. Cheng, A., Lin, H., Kasprow, W., and Rosenheck, R. A.. Impact of supported housing on clinical outcomes: analysis of a randomized trial using multiple imputation technique. Journal of Nervous & Mental Disease 2007. 195 (1) 83-88. **Keywords:** CINAHL/Government Programs/Homeless Persons -- Psychosocial Factors/Mental Disorders -- Therapy/Public

Housing/Substance Use Disorders -- Therapy/Veterans -- Psychosocial Factors/Alcoholism -- Psychosocial Factors/Alcoholism -- Rehabilitation/Alcoholism -- Therapy/Appointments and Schedules/Brief Symptom Inventory/Case Management/Comparative Studies/Descriptive Statistics/Diagnosis,Dual (Psychiatry)/Funding Source/Government Agencies/Government Programs -- Utilization/Hospitals,Veterans/Mental Disorders -- Diagnosis/Mental Disorders -- Psychosocial Factors/Models,Statistical/Multicenter Studies/outcome assessment/P-Value/Prospective Studies/Repeated Measures/Scales/Social Adjustment/Socioenvironmental Therapy/Substance Use Disorders -- Diagnosis/Substance Use Disorders -- Psychosocial Factors/Substance Use Rehabilitation Programs/Two-Tailed Test/United States/United States Department of Veterans Affairs/human.

RefID:8234. Shoptaw, S., Huber, A., Peck, J., Yang, X., Liu, J., Dang, J., Roll, J., Shapiro, B., Rotheram-Fuller, E., and Ling, W.. Randomized, placebo-controlled trial of sertraline and contingency management for the treatment of methamphetamine dependence. Drug & Alcohol Dependence 15-10-2006. 85 (1) 12-18. **Keywords:** CINAHL/Behavior Modification -- Methods/Methamphetamine/Sertraline Hydrochloride -- Therapeutic Use/Substance Dependence -- Therapy/Adult/Analysis of Variance/chi square test/Data Analysis Software/Descriptive Statistics/Double-Blind Studies/Female/Fisher's Exact Test/Funding Source/Interview Guides/Kaplan-Meier Estimator/Male/Medication Compliance/Middle Age/Placebos/Psychological Tests/Random Assignment/Scales/Self Report/Sertraline Hydrochloride -- Administration and Dosage/Sertraline Hydrochloride -- Adverse Effects/Visual Analog Scaling/human.

RefID:8402. Rawson, R. A., McCann, M. J., Flammino, F., Shoptaw, S., Miotto, K., Reiber, C., and Ling, W.. A comparison of contingency management and cognitive-behavioral approaches for stimulant-dependent individuals. Addiction (Abingdon, England) 2006. 101 (2) 267-274. **Keywords:** CINAHL/Cocaine/Cognitive Therapy/Methamphetamine/Reinforcement (Psychology)/Substance Dependence -- Therapy/Analysis of Variance/chi square test/Clinical Trials/Funding Source/Interview Guides/Interviews/post hoc analysis/Psychological Tests/Random Assignment/Repeated Measures/Self Report/Treatment Outcomes/Urinalysis/human.

RefID:8404. Bisaga, A., Aharonovich, E., Garawi, F., Levin, F. R., Rubin, E., Raby, W. N., and Nunes, E. V.. A randomized placebo-controlled trial of gabapentin for cocaine dependence. Drug & Alcohol Dependence 28-2-2006. 81 (3) 267-274. **Keywords:** CINAHL/Cocaine/Gabapentin -- Therapeutic Use/Substance Dependence -- Drug Therapy/Adult/chi square test/Cognitive Therapy/Double-Blind Studies/Ethnic Groups/Female/Funding Source/Gabapentin -- Administration and Dosage/Gabapentin -- Adverse Effects/Interview Guides/Interviews/Kaplan-Meier Estimator/Log-Rank Test/Male/Medication Compliance/Middle Age/Motivational Interviewing/New York/Pearson's Correlation Coefficient/Placebos/Random Assignment/Self Report/Substance Abusers/human.

RefID:8414. Tait, R. J., Hulse, G. K., Robertson, S. I., and Sprivulis, P. C.. Emergency department-based intervention with adolescent substance users: 12-month outcomes. Drug & Alcohol

Dependence 1-9-2005. 79 (3) 359-363. **Keywords:** CINAHL/emergency care/Substance Abuse -- Therapy -- In Adolescence/adolescence/Adult/Analysis of Variance/chi square test/Child/Clinical Trials/Descriptive Statistics/Emergency Service/Emergency Service -- Utilization/Female/Fisher's Exact Test/Interviews/Male/Mann-Whitney U Test/Patient Compliance/Questionnaires/Random Assignment/Funding Source/Research Instruments/Self Report/Spearman's Rank Correlation Coefficient/Two-Tailed Test/Western Australia/human.

RefID:8424. Carroll, K. M., Ball, S. A., Nich, C., Martino, S., Frankforter, T. L., Farentinos, C., Kunkel, L. E., Mikulich-Gilbertson, S. K., Morgenstern, J., Obert, J. L., Polcin, D., Snead, N., and Woody, G. E.. Motivational interviewing to improve treatment engagement and outcome in individuals seeking treatment for substance abuse: a multisite effectiveness study. Drug & Alcohol Dependence 28-2-2006. 81 (3) 301-312. **Keywords:** CINAHL/Motivational Interviewing/Substance Abuse -- Therapy/Treatment Outcomes/Adult/Analysis of Variance/Audiorecording/chi square test/Clinical Trials/Counselors/Descriptive Statistics/Female/Funding Source/Interviews/Intraclass Correlation Coefficient/Male/Middle Age/Outpatients/Random Assignment/Repeated Measures/Research Instruments/Self Report/Substance Abuse Detection/Substance Abusers/Substance Use Rehabilitation Programs/Summated Rating Scaling/United States/human.

RefID:8464. Oliveto, A., Poling, J., Sevarino, K. A., Gonsai, K. R., McCance-Katz, E. F., Stine, S. M., and Kosten, T. R.. Efficacy of dose and contingency management procedures in LAAM-maintained cocaine-dependent patients. Drug & Alcohol Dependence 1-8-2005. 79 (2) 157-165. **Keywords:** CINAHL/Behavior Therapy/Methadone -- Therapeutic Use/Substance Dependence -- Therapy/Analysis of Variance/Behavior Therapy -- Methods/Center for Epidemiological Studies Depression Scale/chi square test/Cocaine/Data Analysis Software/Depression/Double-Blind Studies/Female/Funding Source/Hamilton Rating Scale for Depression/Interview Guides/Kruskal-Wallis Test/Male/Mann-Whitney U Test/Methadone -- Administration and Dosage/Narcotics/Outpatients/Psychological Tests/Random Assignment/Self Report/substance abuse/Substance Abuse Detection/Substance Dependence -- Drug Therapy/Substance Withdrawal Syndrome/Treatment Outcomes/Urinalysis/human.

RefID:8484. Jones, H. E., Wong, C. J., Tuten, M., and Stitzer, M. L.. Reinforcement-based therapy: 12-month evaluation of an outpatient drug-free treatment for heroin abusers. Drug & Alcohol Dependence 1-8-2005. 79 (2) 119-128. **Keywords:** CINAHL/Heroin/Reinforcement (Psychology)/Substance Dependence -- Therapy/Adult/Analysis of Variance/Clinical Trials/Concurrent Validity/Confidence Intervals/Counseling/Data Analysis Software/Female/Funding Source/Housing/Interview Guides/Male/Maryland/Odds Ratio/Predictive Validity/Random Assignment/Self Report/Semi-Structured Interview/Substance Abuse Detection/T-Tests/Treatment Outcomes/Urinalysis/human.

RefID:8510. Lewis, M. W. and Petry, N. M.. Contingency management treatments that reinforce completion of goal-related activities: participation in family activities and its association with outcomes. Drug & Alcohol Dependence 1-8-2005. 79 (2) 267-271. **Keywords:** CINAHL/Behavior Therapy/Family/Goals and Objectives/Reinforcement (Psychology)/Substance Dependence --

Therapy/Adult/chi square test/Confidence Intervals/Connecticut/Correlational Studies/Female/Funding Source/Logistic Regression/Male/Multivariate Analysis/Odds Ratio/Questionnaires/Research Instruments/Reward/Substance Abuse Detection/human.

RefID:9076. Swan, N.. Matching drug abuse treatment services to patient needs boosts outcome effectiveness. Nida Notes 1998. 13 (5) #Start Page#-3p. **Keywords:** CINAHL/Treatment Outcomes/Substance Abuse -- Rehabilitation/Research Instruments/Interviews/random sample/Prospective Studies.

RefID:9109. Kaminer, Y., Burleson, J. A., and Goldberger, R.. Cognitive-behavioral coping skills and psychoeducation therapies for adolescent substance abuse. Journal of Nervous & Mental Disease 2002. 190 (11) 737-745. **Keywords:** CINAHL/Alcoholism -- Rehabilitation -- In Adolescence/Cognitive Therapy -- In Adolescence/Patient Education -- In Adolescence/Psychotherapy,Group/Psychotropic Drugs/Substance Use Disorders -- Rehabilitation -- In Adolescence/adolescence/Alcoholism -- Psychosocial Factors -- In Adolescence/chi square test/Child Behavior Disorders -- Psychosocial Factors/Child Behavior Disorders -- Rehabilitation/Comorbidity/Comparative Studies/Descriptive Statistics/Female/Linear Regression/Outpatients/P-Value/Patient Dropouts -- Psychosocial Factors -- In Adolescence/Personality Assessment -- In Adolescence/Prospective Studies/Psychological Tests/Questionnaires/Scales/structured interview/T-Tests/Urinalysis/Funding Source/human.

RefID:9126. Friedman, A. S., Terras, A., and Glassman, K.. Multimodel substance use intervention program for male delinquents. Journal of Child & Adolescent Substance Abuse 2002. 11 (4) 43-65. **Keywords:** CINAHL/Drug Rehabilitation Programs -- In Adolescence/Random Assignment/Research Instruments/Comparative Studies/multivariate analysis of variance/Descriptive Statistics/T-Tests/structured interview/multiple regression/Violence -- In Adolescence/adolescence/Male/Funding Source/human.

RefID:9379. Nuttbrock, L H, Ng, Mak, Rahav, M, and Rivera, J. Pre- and post-admission attrition of homeless, mentally ill chemical abusers referred to residential treatment programs. Addiction (Abingdon, England) 1997. 92 (10) 1305-1315. **Keywords:** CINAHL/Ambulatory Care Facilities -- Statistics and Numerical Data/Homeless Persons -- Statistics and Numerical Data/Mental Disorders -- Therapy/Patient Dropouts -- Statistics and Numerical Data/Socioenvironmental Therapy -- Statistics and Numerical Data/Substance Use Disorders -- Therapy/Adult/Diagnosis,Dual (Psychiatry)/human/Male/New York/Randomized Controlled Trials/Substance Use Disorders -- Psychosocial Factors/Treatment Refusal.

RefID:9507. Monti, P M, Rohsenow, D J, Michalec, E, Martin, R A, and Abrams, D B. Brief coping skills treatment for cocaine abuse: substance use outcomes at three months. Addiction (Abingdon, England) 1997. 92 (12) 1717-1728. **Keywords:** CINAHL/Adaptation,Psychological/Cocaine/Psychotherapy -- Methods/Substance Use Disorders -- Therapy/Adult/Clinical Trials/Female/human/Male/Prospective Studies/Rural Health/Substance Use Disorders -- Psychosocial Factors/Treatment Outcomes/Urban Health.

RefID:9561. Brennan, P. I.. Cognitive-behavioral program vs. Twelve Step program: comparative effectiveness of two outpatient drug/alcohol treatment models. #journal name# 1998. #volume# (#Issue#) #Start Page#-98. **Keywords:** CINAHL/Alcoholics Anonymous -- Evaluation/Cognitive Therapy -- Evaluation/Substance Use Rehabilitation Programs -- Evaluation/Comparative Studies/Experimental Studies/Outpatients/Public Offenders/Random Assignment/Substance Abusers/Treatment Outcomes/human.

RefID:10602. Friedmann, Peter D., Katz, Elizabeth C., Rhodes, Anne G., Taxman, Faye S., O'Connell, Daniel J., Frisman, Linda K., Burdon, William M., Fletcher, Bennett W., Litt, Mark D., Clarke, Jennifer, and Martin, Steven S.. Collaborative Behavioral Management for Drug-Involved Parolees: Rationale and Design of the Step'n Out Study. Journal of Offender Rehabilitation 2008. 47 (3) 290-318. **Keywords:** drug abuse/behavior modification/Contingency Management/Drug Rehabilitation/Counseling Techniques/Prosocial Behavior/cooperation/Caseworkers/Counselors/Supervision/Program Evaluation/Research Design/ERIC RCTS.

RefID:10619. Kouimtsidis, Christos, Reynolds, Martina, Coulton, Simon, and Drummond, Colin. How Does Cognitive Behaviour Therapy Work with Opioid-Dependent Clients? Results of the UKCBTMM Study. Drugs: Education, Prevention & Policy 2012. 19 (3) 253-258. **Keywords:** Counseling Techniques/Self Efficacy/drug abuse/Coping/Psychotherapy/behavior modification/Cognitive Restructuring/drug therapy/Narcotics/Drug Addiction/Foreign Countries/Comparative Analysis/Cost Effectiveness/Outcomes of Treatment/prediction/ERIC RCTS.

RefID:10734. Titus, Janet C., Dennis, Michael L., Diamond, Guy, Godley, Susan H., Babor, Thomas, Donaldson, Jean, Herrell, James, Tims, Frank, and Webb, Charles. Treatment of Adolescent Marijuana Abuse: A Randomized Clinical Trial. Presentation 1: Structure of the Cannabis Youth Treatment Study. #journal name# 1999. #volume# (#Issue#) #Start Page#-#End Page#. **Keywords:** Adolescents/Counseling Techniques/drug abuse/Intervention/Marijuana/Models/Program Evaluation/ERIC RCTS.

RefID:10784. Liddle, H. A.. Advances in family-based therapy for adolescent substance abuse. Problems of drug dependence 2001. 63rd Annual Scientific Proceedings (#Issue#) 113-115. **Keywords:** #Keywords#.

Level 2: Population not universally screened upon entering a program or organization

RefID:45. Guo, Rui, He, Qian, Shi, Junxin, Gong, Jie, Wang, Hongxing, and Wang, Zengzhen. Short-term impact of cognition-motivation-emotional intelligence-resistance skills program on drug use prevention for school students in Wuhan, China. Journal of Huazhong University of

Science and Technology.Medical sciences = Hua zhong ke ji da xue xue bao.Yi xue Ying De wen ban = Huazhong keji daxue xuebao.Yixue Yingdewen ban 2010. 30 (6) 720-725. **Keywords:** #Keywords#.

RefID:124. O'neill, James M., Clark, Jeffrey K., and Jones, James A.. Promoting mental health and preventing substance abuse and violence in elementary students: a randomized control study of the Michigan Model for Health. The Journal of school health 2011. 81 (6) 320-330. **Keywords:** Aggression/Child/*Child Behavior/Female/*Health Education/Health Promotion/mt [Methods]/Humans/Male/*Mental Health/Questionnaires/Schools/Students/Substance-Related Disorders/ep [Epidemiology]/*Substance-Related Disorders/pc [Prevention & Control]/*Violence/pc [Prevention & Control].

RefID:580. Petras, Hanno, Kellam, Sheppard G., Brown, C. Hendricks, Muthen, Bengt O., Ialongo, Nicholas S., and Poduska, Jeanne M.. Developmental epidemiological courses leading to antisocial personality disorder and violent and criminal behavior: effects by young adulthood of a universal preventive intervention in first- and second-grade classrooms. Drug and alcohol dependence 2008. 95 Suppl 1 (#Issue#) S45-S59. **Keywords:** Achievement/Adolescent/Adult/*Aggression/px [Psychology]/Antisocial Personality Disorder/ep [Epidemiology]/*Antisocial Personality Disorder/pc [Prevention & Control]/*Attention Deficit and Disruptive Behavior Disorders/th [Therapy]/Baltimore/*Behavior Therapy/mt [Methods]/Child/Cohort Studies/Cross-Sectional Studies/Female/Humans/Juvenile Delinquency/pc [Prevention & Control]/Juvenile Delinquency/sn [Statistics & Numerical Data]/Male/Odds Ratio/*Schools/Sex Factors/*Socialization/Substance-Related Disorders/ep [Epidemiology]/*Substance-Related Disorders/pc [Prevention & Control]/*Urban Population/*Violence/pc [Prevention & Control]/Violence/sn [Statistics & Numerical Data].

RefID:637. Drebing, Charles E., Van Ormer, E Alice, Mueller, Lisa, Hebert, Marcie, Penk, Walter E., Petry, Nancy M., Rosenheck, Robert, and Rounsaville, Bruce. Adding contingency management intervention to vocational rehabilitation: outcomes for dually diagnosed veterans. Journal of rehabilitation research and development 2007. 44 (6) 851-865. **Keywords:** *Diagnosis,Dual (Psychiatry)/mt [Methods]/*Employment/px [Psychology]/Female/Humans/Male/Mental Disorders/co [Complications]/Mental Disorders/di [Diagnosis]/*Mental Disorders/rh [Rehabilitation]/Middle Aged/*Rehabilitation,Vocational/mt [Methods]/Reinforcement (Psychology)/Substance-Related Disorders/co [Complications]/Substance-Related Disorders/di [Diagnosis]/*Substance-Related Disorders/rh [Rehabilitation]/United States/*Veterans/px [Psychology]/*Workers' Compensation.

RefID:706. Bernat, Debra H., August, Gerald J., Hektner, Joel M., and Bloomquist, Michael L.. The Early Risers preventive intervention: testing for six-year outcomes and mediational processes. Journal of abnormal child psychology 2007. 35 (4) 605-617. **Keywords:** Aggression/Attention Deficit and Disruptive Behavior Disorders/di [Diagnosis]/*Attention Deficit and Disruptive Behavior Disorders/pc [Prevention & Control]/Attention Deficit and Disruptive Behavior Disorders/px [Psychology]/Camping/Child/Child,Preschool/Combined

Modality Therapy/Conduct Disorder/di [Diagnosis]/*Conduct Disorder/pc [Prevention & Control]/Conduct Disorder/px [Psychology]/*Early Intervention (Education)/Education/Educational Status/Female/Follow-Up Studies/Humans/Longitudinal Studies/Male/Mass Screening/Mentors/Minnesota/Outcome Assessment (Health Care)/Peer Group/Personality Assessment/Psychotherapy,Group/Social Behavior/Social Perception/Substance-Related Disorders/di [Diagnosis]/Substance-Related Disorders/pc [Prevention & Control]/Substance-Related Disorders/px [Psychology].

RefID:716. Spoth, Richard, Clair, Scott, Greenberg, Mark, Redmond, Cleve, and Shin, Chungyeol. Toward dissemination of evidence-based family interventions: maintenance of community-based partnership recruitment results and associated factors. Journal of family psychology : JFP : journal of the Division of Family Psychology of the American Psychological Association (Division 43) 2007. 21 (2) 137-146. **Keywords:** Adolescent/Child/*Child Behavior Disorders/pc [Prevention & Control]/Cohort Studies/*Community Mental Health Services/*Community-Institutional Relations/*Cooperative Behavior/*Evidence-Based Medicine/*Family Therapy/Female/Humans/*Information Dissemination/Iowa/*Juvenile Delinquency/pc [Prevention & Control]/Male/Patient Acceptance of Health Care/*Patient Care Team/Patient Selection/Pennsylvania/Quality Indicators,Health Care/Referral and Consultation/Schools/*Substance-Related Disorders/pc [Prevention & Control]/Universities.

RefID:736. Tracy, Kathlene, Babuscio, Theresa, Nich, Charla, Kiluk, Brian, Carroll, Kathleen M., Petry, Nancy M., and Rounsaville, Bruce J.. Contingency Management to reduce substance use in individuals who are homeless with co-occurring psychiatric disorders. The American journal of drug and alcohol abuse 2007. 33 (2) 253-258. **Keywords:** Adult/*Alcoholism/rh [Rehabilitation]/Analysis of Variance/*Behavior Therapy/mt [Methods]/*Cocaine-Related Disorders/rh [Rehabilitation]/Connecticut/Diagnosis,Dual (Psychiatry)/Female/*Homeless Persons/px [Psychology]/Humans/Male/*Mental Disorders/rh [Rehabilitation]/Pilot Projects/Reward.

RefID:741. Spoth, Richard, Redmond, Cleve, Shin, Chungyeol, Greenberg, Mark, Clair, Scott, and Feinberg, Mark. Substance-use outcomes at 18 months past baseline: the PROSPER Community-University Partnership Trial. American journal of preventive medicine 2007. 32 (5) 395-402. **Keywords:** Adolescent/Child/*Family/px [Psychology]/*Family Therapy/Female/Follow-Up Studies/Humans/*Intervention Studies/Iowa/ep [Epidemiology]/Male/Outcome Assessment (Health Care)/Pennsylvania/ep [Epidemiology]/*Students/px [Psychology]/Substance-Related Disorders/pc [Prevention & Control]/*Substance-Related Disorders/th [Therapy]/Time Factors/Treatment Outcome/Universities.

RefID:750. Wechsberg, Wendee M., Zule, William A., Riehman, Kara S., Luseno, Winnie K., and Lam, Wendy K. K.. African-American crack abusers and drug treatment initiation: barriers and effects of a pretreatment intervention. Substance abuse treatment, prevention, and policy 2007. 2 (#Issue#) 10-#End Page#. **Keywords:** Adult/*African Americans/*Cocaine-Related Disorders/rh [Rehabilitation]/*Communication Barriers/Community-Institutional

Relations/*Crack Cocaine/*Drug Users/Female/Follow-Up Studies/*Health Services Accessibility/Humans/Male/North Carolina/Outcome Assessment (Health Care)/Patient Participation/Program Evaluation.

RefID:1133. Furr-Holden, C. Debra, Ialongo, Nicholas S., Anthony, James C., Petras, Hanno, and Kellam, Sheppard G.. Developmentally inspired drug prevention: middle school outcomes in a school-based randomized prevention trial. Drug and alcohol dependence 2004. 73 (2) 149-158. **Keywords:** Child/Demography/Family/px [Psychology]/Female/Humans/Male/Outcome Assessment (Health Care)/Parent-Child Relations/Regression Analysis/*School Health Services/Schools/Students/*Substance-Related Disorders/ep [Epidemiology]/*Substance-Related Disorders/pc [Prevention & Control].

RefID:1438. Easton, C., Swan, S., and Sinha, R.. Motivation to change substance use among offenders of domestic violence. Journal of substance abuse treatment 2000. 19 (1) 1-5. **Keywords:** Adult/*Anger/Connecticut/ep [Epidemiology]/*Domestic Violence/pc [Prevention & Control]/Domestic Violence/px [Psychology]/Humans/Male/Middle Aged/*Motivation/Prevalence/*Psychotherapy,Group/mt [Methods]/Substance-Related Disorders/ep [Epidemiology]/*Substance-Related Disorders/pc [Prevention & Control]/Treatment Outcome.

RefID:1767. Rohrbach, L. A., Graham, J. W., and Hansen, W. B.. Diffusion of a school-based substance abuse prevention program: predictors of program implementation. Preventive medicine 1993. 22 (2) 237-260. **Keywords:** Alcoholism/pc [Prevention & Control]/Analysis of Variance/Child/Diffusion of Innovation/Discriminant Analysis/Female/Health Education/mt [Methods]/*Health Education/og [Organization & Administration]/*Health Plan Implementation/Health Promotion/Humans/Inservice Training/og [Organization & Administration]/Los Angeles/Male/Marijuana Abuse/pc [Prevention & Control]/Motivation/Multivariate Analysis/Program Development/Program Evaluation/Questionnaires/*School Health Services/og [Organization & Administration]/Social Support/*Substance-Related Disorders/pc [Prevention & Control]/Teaching/mt [Methods]/Teaching/og [Organization & Administration].

RefID:1822. Jones, R. T., McDonald, D. W., Fiore, M. F., Arrington, T., and Randall, J.. A primary preventive approach to children's drug refusal behavior: the impact of rehearsal-plus. Journal of pediatric psychology 1990. 15 (2) 211-223. **Keywords:** *Behavior Therapy/mt [Methods]/Child/Female/*Health Education/mt [Methods]/Humans/*Interpersonal Relations/Male/Risk Factors/*Substance-Related Disorders/pc [Prevention & Control].

RefID:3193. Voshaar, Richard C. O., Krabbe, Paul F. M., Gorgels, Wim J. M. J., Adang, Eddy M. M., van Balkom, Anton J. L. M., van de Lisdonk, Eloy H., and Zitman, Frans G.. Tapering Off Benzodiazepines in Long-Term Users: An Economic Evaluation. PharmacoEconomics 2006. 24 (7) 683-694. **Keywords:** *Benzodiazepines/*Costs and Cost Analysis/*Drug Therapy/*Health Care Costs/Economics.

RefID:3458. Barrett, Holly, Slesnick, Natasha, Brody, Janet L., Turner, Charles W., and Peterson, Thomas R.. Treatment outcomes for adolescent substance abuse at 4- and 7-month assessments. Journal of consulting and clinical psychology 2001. 69 (5) 802-813. **Keywords:** *Cognitive Therapy/*drug abuse/*Drug Rehabilitation/*Treatment Outcomes/Family Therapy/Group Psychotherapy.

RefID:5136. Abbey, Antonia, Oliansky, Denise, Stilianos, Krishna, Hohlstein, Leigh, Anne, and, Kaczynski, and Richard. Substance Abuse Prevention for Second Graders: Are They Too Young to Benefit?. Journal of Applied Developmental Psychology 1990. 11 (2) Apr-June, 149. **Keywords:** Central/Substance-Abuse (D840600)/Prevention- (D657300)/Elementary-School-Students (D252600)/substance abuse prevention program's effectiveness,second graders,experiment/sociology of health and medicine/substance use/abuse & compulsive behaviors (drug abuse,addiction,alcoholism,gambling,eating disorders,etc.) (2079).

RefID:7576. Riggs, N. R., Chou, C., and Pentz, M. A.. Preventing growth in amphetamine use: long-term effects of the Midwestern Prevention Project (MPP) from early adolescence to early adulthood. Addiction (Abingdon, England) 2009. 104 (10) 1691-1699. **Keywords:** CINAHL/Amphetamines/Substance Abuse -- Prevention and Control -- In Adolescence/Substance Abuse -- Prevention and Control -- In Adulthood/adolescence/Adult/Age Factors/Child/Clinical Trials/Data Analysis Software/Female/Funding Source/Male/Missouri/Pretest-Posttest Design/Prospective Studies/Schools,Middle/Self Report/structural equation modeling/Substance Abuse -- Epidemiology/Survival Analysis/human.

RefID:8434. Sun, W., Skara, S., Sun, P., Dent, C. W., and Sussman, S.. Project towards no drug abuse: long-term substance use outcomes evaluation. Preventive medicine 2006. 42 (3) 188-192. **Keywords:** CINAHL/School Health Education -- Evaluation/Substance Abuse -- Prevention and Control -- In Adolescence/adolescence/California/Descriptive Statistics/Female/Funding Source/Interviews/Intraclass Correlation Coefficient/Male/One-Tailed Test/outcomes research/Prospective Studies/Self Report/T-Tests/human.

RefID:9301. Dennison, C. R.. A brief substance abuse intervention for black men with high blood pressure. #journal name# 2000. #volume# (#Issue#) #Start Page#-271. **Keywords:** CINAHL/Substance Abuse -- Rehabilitation/Comorbidity/hypertension/Blacks/Male/Random Assignment/Pretest-Posttest Design/Descriptive Statistics/statistical significance/Time Factors/human.

RefID:10626. Kim, Hyoun K. and Leve, Leslie D.. Substance Use and Delinquency among Middle School Girls in Foster Care: A Three-Year Follow-Up of a Randomized Controlled Trial. Journal of consulting and clinical psychology 2011. 79 (6) 740-750. **Keywords:** Intervention/delinquency/Females/Caregivers/Early Adolescents/Adolescents/foster care/Middle School Students/substance abuse/prevention/Control Groups/Measures (Individuals)/Outcomes of Treatment/Prosocial Behavior/Behavior Problems/Symptoms (Individual Disorders)/ERIC RCTS.

RefID:10637. Redmond, Cleve, Spoth, Richard L., Shin, Chungyeol, Schainker, Lisa M., Greenberg, Mark T., and Feinberg, Mark. Long-Term Protective Factor Outcomes of Evidence-Based Interventions Implemented by Community Teams through a Community-University Partnership. Journal of Primary Prevention 2009. 30 (5) 513-530. **Keywords:** Intervention/Adolescents/Rural Areas/Technical Assistance/Partnerships in Education/Behavior Problems/School Community Programs/Community Services/Questionnaires/Participant Satisfaction/Outcomes of Treatment/Program Effectiveness/teamwork/behavior modification/ERIC RCTS.

RefID:10661. Connell, Arin M., Dishion, Thomas J., and Deater-Deckard, Kirby. Variable- and Person-Centered Approaches to the Analysis of Early Adolescent Substance Use: Linking Peer, Family, and Intervention Effects with Developmental Trajectories. Merrill-Palmer Quarterly: Journal of Developmental Psychology 2006. 52 (3) 421-448. **Keywords:** Crisis Intervention/prevention/Early Adolescents/Adolescents/substance abuse/Individual Development/At Risk Persons/ERIC RCTS.

RefID:10662. Spoth, Richard, Randall, G. Kevin, and Shin, Chungyeol. Increasing School Success through Partnership-Based Family Competency Training: Experimental Study of Long-Term Outcomes. School Psychology Quarterly 2008. 23 (1) 70-89. **Keywords:** Rural Schools/Intervention/Structural Equation Models/academic achievement/Grade 8/Grade 6/Grade 12/competence/Family School Relationship/Family Programs/Student Development/High Risk Students/Hypothesis Testing/High School Seniors/Parent Education/Parenting Skills/substance abuse/Student Participation/Student School Relationship/Adolescents/ERIC RCTS.

RefID:10700. Bond, Lyndal, Thomas, Lyndal, Coffey, Carolyn, Glover, Sara, Butler, Helen, Carlin, John B., and Patton, George. Long-Term Impact of the Gatehouse Project on Cannabis Use of 16-Year-Olds in Australia. Journal of School Health 1-1-2004. 74 (1) 23-#End Page#. **Keywords:** Program Effectiveness/Foreign Countries/Research Papers (Students)/Intervention/Educational Environment/Marijuana/Adolescents/ERIC RCTS.

RefID:10716. Brown, Eric C., Catalano, Richard F., Fleming, Charles B., Haggerty, Kevin P., and Abbott, Robert D.. Adolescent Substance Use Outcomes in the Raising Healthy Children Project: A Two-Part Latent Growth Curve Analysis. Journal of consulting and clinical psychology 2005. 73 (4) 699-710. **Keywords:** Longitudinal Studies/Adolescents/substance abuse/Public Schools/prevention/Intervention/Marijuana/Smoking/drinking/Program Effectiveness/Social Development/Youth Programs/Adolescent Development/ERIC RCTS.

RefID:10721. Ghosh-Dastidar, Bonnie, Longshore, Douglas L., Ellickson, Phyllis L., and McCaffrey, Daniel F.. Modifying Pro-Drug Risk Factors in Adolescents: Results from Project ALERT. Health Education & Behavior 2004. 31 (3) 318-334. **Keywords:** Self Efficacy/Middle School Students/prevention/drug use/Early Adolescents/effect size/Grade 8/Grade 7/Drug Education/At Risk Persons/Rural Areas/Program

Effectiveness/Smoking/drinking/Marijuana/Comprehensive School Health Education/Grade 9/Grade 10/High School Students/Student Characteristics/Beliefs/ERIC RCTS.

RefID:10761. Moskowitz, Joel M. and And, Others. an evaluation of an innovative drug education program: follow-up results. #journal name# 1981. #volume# (#Issue#) #Start Page#-#End Page#. **Keywords:** Age Differences/Decision Making/drug abuse/Drug Education/Educational Innovation/Formative Evaluation/Grade 7/Grade 8/Junior High Schools/Program Effectiveness/Program Evaluation/Sex Differences/Social Studies/Student Attitudes/ERIC RCTS.

RefID:10785. Heather, N., Bowie, A., Ashton, H., McAvoy, B., Spencer, I., Brodie, J., and Giddings, D.. Randomised controlled trial of two brief interventions against long-term benzodiazepine use: outcome of intervention. Addiction Research & Theory 2004. 12 (2) 141-154. **Keywords:** #Keywords#.

RefID:10787. Taylor, A. S., LoSciuto, L., Fox, M., Hilbert, S. M., and Sonkowsky, M.. The mentoring factor: Evaluation of the Across Ages' intergenerational approach to drug abuse. Child & Youth Services 1999. 20 (1-2) 77-99. **Keywords:** from SR bibliographies.

RefID:10791. Ellickson, P. L., McCaffrey, D. F., and Klein, D. J.. Long-term effects of drug prevention on risky sexual behavior among young adults. J Adolesc.Health 2009. 45 (2) 111-117. **Keywords:** Adolescent/Data Collection/Female/Follow-Up Studies/Humans/Male/Program Evaluation/Risk-Taking/Sexual Behavior/South Dakota/Substance-Related Disorders/prevention & control/Unsafe Sex/Young Adult/from SR bibliographies.

RefID:10792. Flay, B. R., Graumlich, S., Segawa, E., Burns, J. L., and Holliday, M. Y.. Effects of 2 prevention programs on high-risk behaviors among African American youth: a randomized trial. Arch Pediatr Adolesc.Med 2004. 158 (4) 377-384. **Keywords:** Adolescent/Adolescent Behavior/Psychology/African Continental Ancestry Group/statistics & numerical data/Agonistic Behavior/Chicago/epidemiology/Child/Condoms/utilization/Curriculum/Female/Health Behavior/Humans/juvenile delinquency/prevention & control/Male/Program Evaluation/Risk-Taking/Sex Factors/Sexual Behavior/Social Change/Substance-Related Disorders/Urban Population/Violence/from SR bibliographies.

RefID:10793. Griffin, K. W., Botvin, G. J., and Nichols, T. R.. Effects of a school-based drug abuse prevention program for adolescents on HIV risk behavior in young adulthood. Prev.Sci 2006. 7 (1) 103-112. **Keywords:** Adolescent/Adolescent Behavior/Adult/Chi-Square Distribution/Female/Follow-Up Studies/HIV Infections/prevention & control/Humans/Intervention Studies/Logistic Models/Male/Outcome Assessment (Health Care)/Randomized Controlled Trials as Topic/Risk-Taking/School Health Services/organization & administration/Substance-Related Disorders/from SR bibliographies.

RefID:10794. McNeal, R. B., Jr., Hansen, W. B., Harrington, N. G., and Giles, S. M.. How all stars works: an examination of program effects on mediating variables. Health Educ.Behav. 2004. 31

(2) 165-178. **Keywords:** Adolescent/Child/Female/Humans/Kentucky/Male/Program Development/Program Evaluation/Risk-Taking/School Health Services/organization & administration/Substance-Related Disorders/prevention & control/from SR bibliographies.

RefID:10800. Aseltine Jr, R. H., Dupre, M., and Lamlein, P.. Mentoring as a Drug Prevention Strategy: An Evaluation of" Across Ages.". Adolescent & Family Health 2000. 1 (1) 11-20. **Keywords:** #Keywords#.

Level 2: Brief intervention explicitly defined but has >4 sessions

RefID:37. Esposito-Smythers, Christianne, Spirito, Anthony, Kahler, Christopher W., Hunt, Jeffrey, and Monti, Peter. Treatment of co-occurring substance abuse and suicidality among adolescents: a randomized trial. Journal of consulting and clinical psychology 2011. 79 (6) 728-739. **Keywords:** #Keywords#.

RefID:119. Bonsack, Charles, Gibellini Manetti, Silvia, Favrod, Jerome, Montagrin, Yves, Besson, Jacques, Bovet, Pierre, and Conus, Philippe. Motivational intervention to reduce cannabis use in young people with psychosis: a randomized controlled trial. Psychotherapy and psychosomatics 2011. 80 (5) 287-297. **Keywords:** Adolescent/Adult/*Cognitive Therapy/Female/Humans/*Interview,Psychological/Male/*Marijuana Abuse/pc [Prevention & Control]/*Motivation/*Psychotherapy,Brief/mt [Methods]/*Psychotic Disorders/rh [Rehabilitation]/Schizophrenia/rh [Rehabilitation]/Switzerland.

RefID:146. Bradford, Andrea, Cully, Jeffrey, Rhoades, Howard, Kunik, Mark, Kraus-Schuman, Cynthia, Wilson, Nancy, and Stanley, Melinda. Early response to psychotherapy and long-term change in worry symptoms in older adults with generalized anxiety disorder. The American journal of geriatric psychiatry : official journal of the American Association for Geriatric Psychiatry 2011. 19 (4) 347-356. **Keywords:** Aged/*Anxiety Disorders/di [Diagnosis]/*Anxiety Disorders/th [Therapy]/*Cognitive Therapy/mt [Methods]/Female/Humans/Linear Models/Logistic Models/Male/Middle Aged/Self Report/Severity of Illness Index/Time Factors/Treatment Outcome.

RefID:1083. Spoth, Richard, Redmond, Cleve, Shin, Chungyeol, and Azevedo, Kari. Brief family intervention effects on adolescent substance initiation: school-level growth curve analyses 6 years following baseline. Journal of consulting and clinical psychology 2004. 72 (3) 535-542. **Keywords:** Adolescent/Child/*Family Therapy/mt [Methods]/Female/Humans/Incidence/Male/*Psychotherapy,Brief/mt [Methods]/Students/Substance-Related Disorders/ep [Epidemiology]/*Substance-Related Disorders/th [Therapy].

RefID:2140. Barrowclough, C., Lobbanl, F., Warburton, J., Choudhry, I., Gregg, L., Wood, H.,

Weavers, T., Holding, J., and Marshall, M.. HELPER ReCAP: Rethinking Choices after Psychosis - A phasespecific psychological therapy for people with problematic cannabis use following a first episode of psychosis. Early Intervention in Psychiatry 2010. 4 (#Issue#) 161-#End Page#. **Keywords:** *psychosis/*lifespan/*human/*therapy/relapse/follow up/Risk/homelessness/Cognitive Therapy/randomized controlled trial/early intervention/Mental Health/United Kingdom/diagnosis/patient/Violence/general aspects of disease/*Cannabis/reducing agent.

Level 2: Does not meet the definition of a BI

RefID:91. Liddle, Howard A., Dakof, Gayle A., Henderson, Craig, and Rowe, Cindy. Implementation outcomes of Multidimensional Family Therapy-Detention to Community: a reintegration program for drug-using juvenile detainees. International journal of offender therapy and comparative criminology 2011. 55 (4) 587-604. **Keywords:** Adolescent/Alcoholism/px [Psychology]/*Alcoholism/rh [Rehabilitation]/Antisocial Personality Disorder/px [Psychology]/*Antisocial Personality Disorder/rh [Rehabilitation]/Child/Combined Modality Therapy/*Community Mental Health Services/og [Organization & Administration]/Comorbidity/Conduct Disorder/px [Psychology]/Conduct Disorder/rh [Rehabilitation]/Cooperative Behavior/*Family Therapy/mt [Methods]/*Family Therapy/og [Organization & Administration]/Female/*Health Plan Implementation/Humans/Interdisciplinary Communication/Interview,Psychological/*Juvenile Delinquency/lj [Legislation & Jurisprudence]/Juvenile Delinquency/px [Psychology]/*Juvenile Delinquency/rh [Rehabilitation]/Male/Mental Disorders/px [Psychology]/Mental Disorders/rh [Rehabilitation]/*Prisoners/px [Psychology]/Residential Treatment/*Social Environment/*Socialization/Substance-Related Disorders/px [Psychology]/*Substance-Related Disorders/rh [Rehabilitation]/Systems Theory/Texas/Young Adult.

RefID:149. Spoth, Richard, Redmond, Cleve, Clair, Scott, Shin, Chungyeol, Greenberg, Mark, and Feinberg, Mark. Preventing substance misuse through community-university partnerships: randomized controlled trial outcomes 42-Jan years past baseline. American journal of preventive medicine 2011. 40 (4) 440-447. **Keywords:** Adolescent/Analysis of Variance/Child/*Community-Institutional Relations/Cooperative Behavior/Evidence-Based Medicine/Follow-Up Studies/Humans/Iowa/*Models,Organizational/Outcome and Process Assessment (Health Care)/Pennsylvania/Questionnaires/Rural Population/Students/sn [Statistics & Numerical Data]/*Substance-Related Disorders/pc [Prevention & Control]/Time Factors/*Universities/og [Organization & Administration].

RefID:188. Menza, Timothy W., Jameson, Damon R., Hughes, James P., Colfax, Grant N., Shoptaw, Steven, and Golden, Matthew R.. Contingency management to reduce methamphetamine use and sexual risk among men who have sex with men: a randomized controlled trial. BMC public health 2010. 10 (#Issue#) 774-#End Page#. **Keywords:** Adolescent/Adult/*Amphetamine-Related Disorders/rh [Rehabilitation]/*Behavior Therapy/mt [Methods]/Counseling/Crack Cocaine/ur [Urine]/HIV Infections/pc [Prevention &

Control]/*Homosexuality,Male/Humans/Interviews as Topic/Male/Methamphetamine/ur [Urine]/*Methamphetamine/Middle Aged/Patient Selection/*Reinforcement (Psychology)/Research Design/*Risk-Taking/Sexual Behavior/Sexually Transmitted Diseases/pc [Prevention & Control].

RefID:320. Riggs, Nathaniel R., Chou, Chih Ping, and Pentz, Mary Ann. Preventing growth in amphetamine use: long-term effects of the Midwestern Prevention Project (MPP) from early adolescence to early adulthood. Addiction (Abingdon, England) 2009. 104 (10) 1691-1699. **Keywords:** Adolescent/Adult/Age of Onset/Amphetamine-Related Disorders/ep [Epidemiology]/*Amphetamine-Related Disorders/pc [Prevention & Control]/*Amphetamines/ae [Adverse Effects]/*Central Nervous System Stimulants/ae [Adverse Effects]/Child/Female/Humans/Male/*Models,Statistical/*Preventive Health Services/Program Evaluation/Schools/Self Report/Survival Analysis/United States/ep [Epidemiology]/Urban Population/Young Adult.

RefID:420. Riggs, Nathaniel R. and Pentz, Mary Ann. Long-term effects of adolescent marijuana use prevention on adult mental health services utilization: the midwestern prevention project. Substance use & misuse 2009. 44 (5) 616-631. **Keywords:** Adolescent/Adult/Child/Cross-Sectional Studies/Female/*Health Education/Humans/Longitudinal Studies/Male/Marijuana Abuse/ep [Epidemiology]/*Marijuana Abuse/pc [Prevention & Control]/Mental Disorders/ep [Epidemiology]/Mental Disorders/pc [Prevention & Control]/*Mental Health Services/ut [Utilization]/Missouri/Models,Statistical/Outcome Assessment (Health Care)/Utilization Review/sn [Statistics & Numerical Data]/Young Adult.

RefID:542. Hogue, Aaron, Henderson, Craig E., Dauber, Sarah, Barajas, Priscilla C., Fried, Adam, and Liddle, Howard A.. Treatment adherence, competence, and outcome in individual and family therapy for adolescent behavior problems. Journal of consulting and clinical psychology 2008. 76 (4) 544-555. **Keywords:** Adolescent/Child Behavior Disorders/px [Psychology]/*Child Behavior Disorders/rh [Rehabilitation]/*Cognitive Therapy/Comorbidity/*Family Therapy/Female/Follow-Up Studies/Humans/Internal-External Control/Male/*Patient Compliance/px [Psychology]/*Professional Competence/Professional-Patient Relations/Substance-Related Disorders/px [Psychology]/*Substance-Related Disorders/rh [Rehabilitation].

RefID:605. Milby, Jesse B., Schumacher, Joseph E., Vuchinich, Rudy E., Freedman, Michelle J., Kertesz, Stefan, and Wallace, Dennis. Toward cost-effective initial care for substance-abusing homeless. Journal of substance abuse treatment 2008. 34 (2) 180-191. **Keywords:** Adult/*Cocaine-Related Disorders/rh [Rehabilitation]/*Cognitive Therapy/mt [Methods]/Cost-Benefit Analysis/Female/Health Care Costs/*Homeless Persons/Humans/Longitudinal Studies/Male/Middle Aged/Patient Compliance/Rehabilitation,Vocational/Reinforcement (Psychology)/Reward/*Substance-Related Disorders/rh [Rehabilitation].

RefID:641. Perry, Cheryl L., Lee, Susanne, Stigler, Melissa H., Farbakhsh, Kian, Komro, Kelli A., Gewirtz, Abigail H., and Williams, Carolyn L.. The impact of Project Northland on selected

MMPI-A problem behavior scales. The journal of primary prevention 2007. 28 (5) 449-465. **Keywords:** Adolescent/Adolescent Health Services/*Alcohol Drinking/pc [Prevention & Control]/Female/Health Education/Health Status Indicators/Humans/MMPI/Male/*Mental Disorders/Minnesota/*Preventive Health Services/*Preventive Medicine/Program Development/*Program Evaluation/Psychological Tests/Psychometrics/*Schools/*Students/*Substance-Related Disorders/pc [Prevention & Control].

RefID:657. Johnson, S., Thornicroft, G., Afuwape, S., Leese, M., White, I. R., Hughes, E., Wanigaratne, S., Miles, H., and Craig, T.. Effects of training community staff in interventions for substance misuse in dual diagnosis patients with psychosis (COMO study): cluster randomised trial. The British journal of psychiatry : the journal of mental science 2007. 191 (#Issue#) 451-452. **Keywords:** Clinical Competence/Community Mental Health Services/*Community Psychiatry/ed [Education]/Diagnosis,Dual (Psychiatry)/*Education,Medical,Continuing/mt [Methods]/Follow-Up Studies/Hospitalization/sn [Statistics & Numerical Data]/Humans/London/*Psychotic Disorders/th [Therapy]/*Substance-Related Disorders/th [Therapy]/Treatment Outcome.

RefID:862. Edwards, J., Elkins, K., Hinton, M., Harrigan, S. M., Donovan, K., Athanasopoulos, O., and McGorry, P. D.. Randomized controlled trial of a cannabis-focused intervention for young people with first-episode psychosis. Acta psychiatrica Scandinavica 2006. 114 (2) 109-117. **Keywords:** Adolescent/Adult/Community Mental Health Services/Female/Humans/Male/*Marijuana Abuse/ep [Epidemiology]/*Marijuana Abuse/th [Therapy]/*Psychotherapy/mt [Methods]/*Psychotic Disorders/ep [Epidemiology]/Single-Blind Method.

RefID:904. Sun, Wei, Skara, Silvana, Sun, Ping, Dent, Clyde W., and Sussman, Steve. Project Towards No Drug Abuse: long-term substance use outcomes evaluation. Preventive medicine 2006. 42 (3) 188-192. **Keywords:** Adolescent/*Adolescent Behavior/px [Psychology]/Adult/California/ep [Epidemiology]/Curriculum/Female/Follow-Up Studies/*Health Promotion/mt [Methods]/Health Promotion/og [Organization & Administration]/Humans/Interviews as Topic/Male/*Motivation/Program Evaluation/Regression Analysis/*School Health Services/og [Organization & Administration]/Substance-Related Disorders/ep [Epidemiology]/*Substance-Related Disorders/pc [Prevention & Control].

RefID:1057. Nuttbrock, Larry A., Rosenblum, Andrew, Magura, Stephen, Villano, Cherie, and Wallace, Joyce. Linking female sex workers with substance abuse treatment. Journal of substance abuse treatment 2004. 27 (3) 233-239. **Keywords:** Adolescent/Adult/*Community-Institutional Relations/Counseling/Data Interpretation,Statistical/Female/Follow-Up Studies/Heroin Dependence/ep [Epidemiology]/*Heroin Dependence/rh [Rehabilitation]/Hispanic Americans/px [Psychology]/Hispanic Americans/sn [Statistics & Numerical Data]/Humans/*Methadone/tu [Therapeutic Use]/*Mobile Health Units/Motivation/New York City/Outcome and Process Assessment (Health Care)/sn [Statistics

& Numerical Data]/Patient Acceptance of Health Care/sn [Statistics & Numerical Data]/Prostitution/px [Psychology]/*Prostitution/sn [Statistics & Numerical Data]/Referral and Consultation/sn [Statistics & Numerical Data]/*Street Drugs/Substance-Related Disorders/ep [Epidemiology]/*Substance-Related Disorders/rh [Rehabilitation]/Urban Population/sn [Statistics & Numerical Data]/*Urban Population.

RefID:1115. McNeal, Ralph B. J., Hansen, William B., Harrington, Nancy G., and Giles, Steven M.. How all stars works: an examination of program effects on mediating variables. Health education & behavior : the official publication of the Society for Public Health Education 2004. 31 (2) 165-178. **Keywords:** Adolescent/Child/Female/Humans/Kentucky/Male/Program Development/Program Evaluation/Risk-Taking/*School Health Services/og [Organization & Administration]/*Substance-Related Disorders/pc [Prevention & Control].

RefID:1356. Scheier, L. M., Botvin, G. J., and Griffin, K. W.. Preventive intervention effects on developmental progression in drug use: structural equation modeling analyses using longitudinal data. Prevention science : the official journal of the Society for Prevention Research 2001. 2 (2) 91-112. **Keywords:** Adolescent/Alcohol Drinking/ae [Adverse Effects]/*Cognitive Therapy/mt [Methods]/Female/Follow-Up Studies/Humans/Male/Marijuana Smoking/ae [Adverse Effects]/Models,Psychological/New England/*Psychotherapy,Brief/Questionnaires/Schools/Smoking/ae [Adverse Effects]/*Substance-Related Disorders/et [Etiology]/*Substance-Related Disorders/pc [Prevention & Control].

RefID:1424. Weiss, R. D., Griffin, M. L., Gallop, R., Luborsky, L., Siqueland, L., Frank, A., Onken, L. S., Daley, D. C., and Gastfriend, D. R.. Predictors of self-help group attendance in cocaine dependent patients. Journal of studies on alcohol 2000. 61 (5) 714-719. **Keywords:** Adult/Alcoholics Anonymous/Cocaine-Related Disorders/di [Diagnosis]/*Cocaine-Related Disorders/th [Therapy]/Female/Humans/Male/*Patient Compliance/sn [Statistics & Numerical Data]/Prospective Studies/*Self-Help Groups/Severity of Illness Index.

RefID:1509. McGillicuddy, N. B. and Blane, H. T.. Substance use in individuals with mental retardation. Addictive behaviors 1999. 24 (6) 869-878. **Keywords:** Adult/*Behavior Therapy/Female/Humans/*Intellectual Disability/px [Psychology]/Interpersonal Relations/Intervention Studies/Male/Personality/Social Behavior/Substance-Related Disorders/pc [Prevention & Control]/*Substance-Related Disorders/px [Psychology].

RefID:1568. Elk, R., Mangus, L., Rhoades, H., Andres, R., and Grabowski, J.. Cessation of cocaine use during pregnancy: effects of contingency management interventions on maintaining abstinence and complying with prenatal care. Addictive behaviors 1998. 23 (1) 57-64. **Keywords:** Adult/Cocaine-Related Disorders/ec [Economics]/*Cocaine-Related Disorders/rh [Rehabilitation]/Cost-Benefit Analysis/Female/Humans/*Maternal Exposure/pc [Prevention & Control]/Patient Compliance/Pregnancy/Pregnancy Complications/ec [Economics]/*Pregnancy Complications/rh [Rehabilitation]/Pregnancy Outcome/Prenatal Care/ec [Economics]/*Prenatal Care/Reward/Substance Abuse Detection.

RefID:1594. McLellan, A. T., Grissom, G. R., Zanis, D., Randall, M., Brill, P., and O'Brien, C. P.. Problem-service 'matching' in addiction treatment. A prospective study in 4 programs. Archives of general psychiatry 1997. 54 (8) 730-735. **Keywords:** Adult/Clinical Protocols/Female/Humans/Male/*Patient Selection/Prospective Studies/Substance Abuse Treatment Centers/*Substance-Related Disorders/th [Therapy]/Treatment Outcome.

RefID:1724. Black, M. M., Nair, P., Kight, C., Wachtel, R., Roby, P., and Schuler, M.. Parenting and early development among children of drug-abusing women: effects of home intervention. Pediatrics 1994. 94 (4 Pt 1) 440-448. **Keywords:** Adult/Child Advocacy/*Child Development/Child Health Services/ut [Utilization]/*Child of Impaired Parents/*Community Health Nursing/og [Organization & Administration]/Female/*Health Knowledge,Attitudes,Practice/Health Promotion/*Home Care Services/og [Organization & Administration]/Humans/Infant/Intervention Studies/Logistic Models/*Mothers/ed [Education]/*Mothers/px [Psychology]/Multivariate Analysis/*Parenting/px [Psychology]/Patient Compliance/Program Evaluation/Social Support/*Substance Abuse,Intravenous/px [Psychology].

RefID:1752. Kelly, J. A., Murphy, D. A., Bahr, G. R., Kalichman, S. C., Morgan, M. G., Stevenson, L. Y., Koob, J. J., Brasfield, T. L., and Bernstein, B. M.. Outcome of cognitive-behavioral and support group brief therapies for depressed, HIV-infected persons. The American journal of psychiatry 1993. 150 (11) 1679-1686. **Keywords:** Adult/*Cognitive Therapy/Depressive Disorder/et [Etiology]/Depressive Disorder/px [Psychology]/*Depressive Disorder/th [Therapy]/Follow-Up Studies/*HIV Seropositivity/co [Complications]/HIV Seropositivity/px [Psychology]/Humans/Male/*Psychotherapy,Brief/*Psychotherapy,Group/Social Support/Stress,Psychological/et [Etiology]/Stress,Psychological/px [Psychology]/Stress,Psychological/th [Therapy]/Treatment Outcome.

RefID:1764. Ellickson, P. L., Bell, R. M., and McGuigan, K.. Preventing adolescent drug use: long-term results of a junior high program. American journal of public health 1993. 83 (6) 856-861. **Keywords:** Adolescent/*Adolescent Behavior/Analysis of Variance/California/Follow-Up Studies/Health Promotion/mt [Methods]/*Health Promotion/Humans/Intervention Studies/Oregon/Schools/*Substance-Related Disorders/pc [Prevention & Control].

RefID:1809. Foote, A. and Erfurt, J. C.. Effects of EAP follow-up on prevention of relapse among substance abuse clients. Journal of studies on alcohol 1991. 52 (3) 241-248. **Keywords:** Adult/*Alcoholism/rh [Rehabilitation]/Female/Follow-Up Studies/Humans/Job Satisfaction/Male/*Occupational Health Services/Recurrence/Referral and Consultation/*Substance-Related Disorders/rh [Rehabilitation].

RefID:1873. Szapocznik, J., Kurtines, W. M., Foote, F., Perez-Vidal, A., and Hervis, O.. Conjoint versus one-person family therapy: further evidence for the effectiveness of conducting family therapy through one person with drug-abusing adolescents. Journal of consulting and clinical psychology 1986. 54 (3) 395-397. **Keywords:** Adolescent/*Family Therapy/mt

[Methods]/Humans/Psychotherapy,Brief/mt [Methods]/Substance-Related Disorders/px [Psychology]/*Substance-Related Disorders/th [Therapy].

RefID:1985. Kim, H. K. and Leve, L. D.. Substance use and delinquency among middle school girls in foster care: A three-year follow-up of a randomized controlled trial. Journal of consulting and clinical psychology 2011. 79 (6) 740-750. **Keywords:** alcohol consumption/article/cannabis addiction/pc [Prevention]/caregiver/Child/controlled study/Female/follow up/*foster care/*health program/health service/human/*juvenile delinquency/pc [Prevention]/major clinical study/*middle school student/outcome assessment/school child/scoring system/Smoking/Social Behavior/*substance abuse/summer/therapy effect.

RefID:2084. Ozhathil, D. K., Abar, B., Yeboah, K., Arjun, S., Gaylord, L., Baumann, B. M., Grissom, G., and Boudreaux, E. D.. The external validity of consecutive sampling protocols among emergency department patients. Academic Emergency Medicine 2011. 18 (5 SUPPL. 1) S77-#End Page#. **Keywords:** *human/*patient/*emergency ward/*Emergency Medicine/*sampling/*external validity/*society/cigarette smoking/population/African American/drug use/ethnicity/tobacco/randomized controlled trial/follow up/univariate analysis/Student t test/Demography/Adult/screening/alcohol/illicit drug.

RefID:3013. Sacks, Joann Y., Sacks, Stanley, McKendrick, Karen, Banks, Steven, Schoeneberger, Marlies, Hamilton, Zachary, Stommel, Joseph, and Shoemaker, Joanie. Prison therapeutic community treatment for female offenders: Profiles and preliminary findings for mental health and other variables (crime, substance use and HIV risk). Journal of Offender Rehabilitation 2008. 46 (3-4) 233-261. **Keywords:** *Criminal Rehabilitation/*Mental Disorders/*Severity (Disorders)/*Therapeutic Community/Cognitive Behavior Therapy/Crime/Criminals/diagnosis/drug abuse/HIV/Intervention/Mental Health/posttraumatic stress disorder/Prisons.

RefID:3170. Odom, Anna E.. A randomized study of integrated outpatient treatment and assertive community treatment for patients with comorbid mental illness and substance use disorders: Comparing treatment outcome for domiciled and homeless patients. Dissertation Abstracts International: Section B: The Sciences and Engineering 2006. 66 (8-B) 4495-#End Page#. **Keywords:** *comorbidity/*drug abuse/*Homeless/*Mental Disorders/*Outpatient Treatment/Communities/Patients/Quality of Life/schizophrenia/Treatment Outcomes.

RefID:3270. Haack, Mary R., Burda-Cohee, Charon, Alemi, Farrokh, Harge, Angela, and Nemes, Susanna. Facilitating Self-Management of Substance Use Disorders with Online Counseling: The Intervention and Study Design. Journal of Addictions Nursing 2005. 16 (1-2) 41-46. **Keywords:** *Counseling/*drug abuse/*Online Therapy/*Parents/*Self Management/Child Abuse/Child Neglect/Internet.

RefID:3373. Kinlock, Timothy W., O'Grady, Kevin E., and Hanlon, Thomas E.. The effects of drug treatment on institutional behavior. The Prison Journal 2003. 83 (3) 257-276. **Keywords:** *Brief Psychotherapy/*Cognitive Behavior Therapy/*Drug

Rehabilitation/*Incarceration/*Psychotherapeutic Outcomes/drug abuse/Perpetrators.

RefID:3376. Marlowe, Douglas B., Kirby, Kimberly C., Festinger, David S., Merikle, Elizabeth P., Tran, Giao Q., and Platt, Jerome J.. Day treatment for cocaine dependence: Incremental utility over outpatient counseling and voucher incentives. Addictive behaviors 2003. 28 (2) 387-398. **Keywords:** *Client Attitudes/*Cocaine/*drug abuse/*Drug Rehabilitation/*Partial Hospitalization/Client Participation/Cognitive Therapy/Contingency Management/Counseling/Drug Dependency/Monetary Incentives/Outpatient Treatment/urine.

RefID:3526. Linehan, Marsha M., Schmidt, Henry III, Dimeff, Linda A., Craft, J. Christopher, Kanter, Jonathan, and Comtois, Katherine A.. Dialectical behavior therapy for patients with borderline personality disorder and drug-dependence. The American Journal on Addictions 1999. 8 (4) 279-292. **Keywords:** *Behavior Therapy/*Borderline States/*Drug Rehabilitation/*Psychotherapeutic Outcomes/*Suicidal Ideation/Personality Disorders/Dialectical Behavior Therapy.

RefID:3795. Spoth, R., Redmond, C., Clair, S., Shin, C., Greenberg, M., and Feinberg, M.. Preventing substance misuse through community-university partnerships: randomized controlled trial outcomes 42-Jan years past baseline. American journal of preventive medicine 2011. 40 (4) 440-447. **Keywords:** Multifile Reviews/Adolescent/Analysis of Variance/Child/Community-Institutional Relations/Cooperative Behavior/Evidence-Based Medicine/Follow-Up Studies/Humans/Iowa/Models/Organizational/Outcome and Process Assessment (Health Care)/Pennsylvania/Questionnaires/Rural Population/Students/sn [Statistics & Numerical Data]/Substance-Related Disorders/pc [Prevention & Control]/Time Factors/Universities/og [Organization & Administration].

RefID:4722. Dasberg, H. and van Praag, H. M.. The therapeutic effect of short-term oral diazepam treatment on acute clinical anxiety in a crisis centre. Acta psychiatrica Scandinavica 1974. 50 (3) 326-340. **Keywords:** Central/Acute Disease/Administration,Oral/Anxiety Disorders [blood] [drug therapy]/Clinical Trials as Topic/Crisis Intervention/Diazepam [administration & dosage] [blood] [therapeutic use]/Placebos/Psychiatric Status Rating Scales/Questionnaires/Time Factors/Adult/Aged/Female/Humans/Male/Middle Aged.

RefID:5469. Platt, J. J., Kirby, K. C., Marlowe, D. B., Lamb, R. J., and Lidz, V.. Effectiveness of outpatient versus day treatment for cocaine users. NIDA research monograph 1996. 162 (#Issue#) 90-#End Page#. **Keywords:** Central.

RefID:7200. Black, A. C. and Rosen, M. I.. A money management-based substance use treatment increases valuation of future rewards. Addictive behaviors 2011. 36 (1-2) 125-128. **Keywords:** CINAHL/Alcohol-Related Disorders -- Economics/Behavior,Addictive -- Economics/Substance Use Disorders -- Economics/Adult/Alcohol-Related Disorders -- Psychosocial Factors/Alcohol-Related Disorders -- Rehabilitation/Behavior,Addictive -- Psychosocial Factors/Behavior,Addictive -- Rehabilitation/Budgets/Female/human/Male/Randomized Controlled Trials/Reward/Substance Use Disorders -- Psychosocial Factors/Substance Use

Disorders -- Rehabilitation.

RefID:7364. Lucas, G. M., Chaudhry, A., Hsu, J., Woodson, T., Lau, B., Olsen, Y., Keruly, J. C., Fiellin, D. A., Finkelstein, R., Barditch-Crovo, P., Cook, K., and Moore, R. D.. Clinic-based treatment of opioid-dependent HIV-infected patients versus referral to an opioid treatment program: A randomized trial. Annals of internal medicine 1-6-2010. 152 (11) 704-711. **Keywords:** CINAHL/Buprenorphine -- Therapeutic Use/Community Health Services -- Standards/HIV Infections -- Complications/Naloxone -- Therapeutic Use/Narcotic Antagonists -- Therapeutic Use/Substance Use Disorders -- Drug Therapy/Antiviral Agents -- Therapeutic Use/Maryland/Drug Therapy,Combination/HIV Infections -- Drug Therapy/human/Substance Use Disorders -- Complications/outcome assessment/Referral and Consultation/Substance Use Rehabilitation Programs -- Standards/Treatment Outcomes/Clinical Trials.

RefID:7368. Milby, J. B., Schumacher, J. E., Wallace, D., Vuchinich, R., Mennemeyer, S. T., and Kertesz, S. G.. Effects of sustained abstinence among treated substance-abusing homeless persons on housing and employment. American journal of public health 2010. 100 (5) 913-918. **Keywords:** CINAHL/Employment/Homeless Persons -- Alabama/Housing/Patient Compliance/Substance Use Rehabilitation Programs/Treatment Outcomes/Adult/Age Factors/Alabama/chi square test/Clinical Trials/Cocaine -- Drug Effects/Cognitive Therapy/Comparative Studies/Confidence Intervals/Contingency Management/Correlational Studies/Descriptive Statistics/Female/Funding Source/human/Logistic Regression/Male/Middle Age/Odds Ratio/Predictive Validity/Probability/Sex Factors/Time Factors.

RefID:7523. Mueser, K. T., Glynn, S. M., Cather, C., Zarate, R., Fox, L., Feldman, J., Wolfe, R., and Clark, R. E.. Family intervention for co-occurring substance use and severe psychiatric disorders: participant characteristics and correlates of initial engagement and more extended exposure in a randomized controlled trial. Addictive behaviors 2009. 34 (10) 867-877. **Keywords:** CINAHL/Family/Patient Education -- Methods/Schizophrenia -- Rehabilitation/Substance Use Disorders -- Rehabilitation/Support,Psychosocial/Adult/Aged/Alcohol-Related Disorders -- Epidemiology/Alcohol-Related Disorders -- Rehabilitation/California/Comorbidity/Diagnosis,Dual (Psychiatry)/Female/human/Male/Massachusetts/Middle Age/Motivation/Random Assignment/Randomized Controlled Trials/Schizophrenia -- Epidemiology/Substance Use Disorders -- Epidemiology/Young Adult.

RefID:8871. Bartels, S. J., Coakley, E. H., Zubritsky, C., Ware, J. H., Miles, K. M., Areán, P. A., Chen, H., Oslin, D. W., Llorente, M. D., Costantino, G., Quijano, L., McIntyre, J. S., Linkins, K. W., Oxman, T. E., Maxwell, J., and Levkoff, S. E.. Improving access to geriatric mental health services: a randomized trial comparing treatment engagement with integrated versus enhanced referral care for depression, anxiety, and at-risk alcohol use. American Journal of Psychiatry 2004. 161 (8) 1455-1462. **Keywords:** CINAHL/Alcohol Abuse -- Therapy -- In Old Age/Anxiety -- Therapy -- In Old Age/Depression -- Therapy -- In Old Age/Gerontologic Care -- Evaluation/Health Care Delivery,Integrated -- Evaluation -- In Old Age/Mental Health Services -- Evaluation -- In Old Age/Quality Improvement/Aged/Center for Epidemiological Studies

Depression Scale/chi square test/Clinical Trials/Confidence Intervals/Descriptive Statistics/Female/Male/Multiple Logistic Regression/Odds Ratio/P-Value/Psychological Tests/Scales/Short Form-36 Health Survey (SF-36)/Funding Source/human.

RefID:8971. McNeal, R. B., Jr., Hansen, W. B., Harrington, N. G., and Giles, S. M.. How All Stars works: an examination of program effects of mediating variables. Health Education & Behavior 2004. 31 (2) 165-#End Page#. **Keywords:** CINAHL/Risk Taking Behavior -- Prevention and Control -- In Adolescence/School Health Education/Substance Abuse -- Prevention and Control -- In Adolescence/adolescence/Clinical Trials/Coefficient Alpha/Female/Field Studies/Kentucky/Male/Pretest-Posttest Design/Prospective Studies/Random Assignment/Scales/Students,Middle School/Surveys/human.

RefID:9537. Swenson-Britt, E., Carrougher, G., Martin, B. W., and Brackley, M.. Project Hope: changing care delivery for the substance abuse patient. Clinical Nurse Specialist: The Journal for Advanced Nursing Practice 2000. 14 (2) 92-100. **Keywords:** CINAHL/Alcohol Rehabilitation Programs/Substance Abusers/Alcoholism/Alcoholism -- Prevention and Control/Nurse Attitudes/Inpatients/Clinical Nurse Specialists/Nursing Role/Quasi-Experimental Studies/Questionnaires/Solomon Four-Group Design/Scales/Staff Development/Attitude Measures/Program Evaluation/Research Instruments/Outcomes of Education/Course Content/Analysis of Variance/Alcoholism -- Nursing/Two-Way Analysis of Variance/human.

RefID:10786. Wu, Y., Stanton, B. F., Galbraith, J., Kaljee, L., Cottrell, L., Li, X., Harris, C. V., D'Alessandri, D., and Burns, J. M.. Sustaining and broadening intervention impact: a longitudinal randomized trial of 3 adolescent risk reduction approaches. Pediatrics 2003. 111 (1) e32-e38. **Keywords:** Adolescent/Adolescent Behavior/Psychology/Child/Cohort Studies/Communication/Female/Humans/Longitudinal Studies/Male/Parent-Child Relations/Risk Reduction Behavior/Risk-Taking/Substance-Related Disorders/epidemiology/prevention & control/from SR bibliographies.

RefID:10788. Slesnick, N., Prestopnik, J. L., Meyers, R. J., and Glassman, M.. Treatment outcome for street-living, homeless youth. Addict.Behav. 2007. 32 (6) 1237-1251. **Keywords:** Adolescent/Adult/Community Mental Health Services/organization & administration/Female/Homeless Youth/statistics & numerical data/Humans/Male/Questionnaires/Reinforcement (Psychology)/Social Behavior/Substance-Related Disorders/epidemiology/rehabilitation/Treatment Outcome/from SR bibliographies.

Level 2: Report does not present results separately for non-medical use of psychoactive substances of interest

RefID:246. Zanjani, Faika, Bush, Heather, and Oslin, David. Telephone-based psychiatric referral-care management intervention health outcomes. Telemedicine journal and e-health : the official journal of the American Telemedicine Association 2010. 16 (5) 543-550. **Keywords:**

Depressive Disorder/di [Diagnosis]/Depressive Disorder/th [Therapy]/Diagnosis,Dual (Psychiatry)/Female/Follow-Up Studies/Hospitals,Veterans/Humans/Interview,Psychological/Male/*Mental Health Services/og [Organization & Administration]/Middle Aged/Outcome Assessment (Health Care)/Philadelphia/Program Evaluation/*Psychiatry/og [Organization & Administration]/Psychotherapy,Brief/*Referral and Consultation/og [Organization & Administration]/Substance-Related Disorders/di [Diagnosis]/Substance-Related Disorders/th [Therapy]/*Telephone/Veterans.

RefID:491. Baer, John S., Beadnell, Blair, Garrett, Sharon B., Hartzler, Bryan, Wells, Elizabeth A., and Peterson, Peggy L.. Adolescent change language within a brief motivational intervention and substance use outcomes. Psychology of addictive behaviors : journal of the Society of Psychologists in Addictive Behaviors 2008. 22 (4) 570-575. **Keywords:** Adolescent/Alcoholism/px [Psychology]/*Alcoholism/rh [Rehabilitation]/Female/Follow-Up Studies/Health Education/*Homeless Youth/px [Psychology]/Humans/*Intention/Interview,Psychological/Male/*Motivation/*Psychotherapy,Brief/mt [Methods]/*Semantics/*Street Drugs/Substance-Related Disorders/px [Psychology]/*Substance-Related Disorders/rh [Rehabilitation]/Treatment Outcome/*Verbal Behavior.

RefID:1519. Swanson, A. J., Pantalon, M. V., and Cohen, K. R.. Motivational interviewing and treatment adherence among psychiatric and dually diagnosed patients. The Journal of nervous and mental disease 1999. 187 (10) 630-635. **Keywords:** Adult/Ambulatory Care/Attitude to Health/Comorbidity/Counseling/Diagnosis,Dual (Psychiatry)/Female/*Hospitalization/Humans/Male/Mental Disorders/di [Diagnosis]/Mental Disorders/ep [Epidemiology]/*Mental Disorders/th [Therapy]/Models,Psychological/*Motivation/*Patient Compliance/Psychotherapy,Brief/Substance-Related Disorders/di [Diagnosis]/Substance-Related Disorders/ep [Epidemiology]/*Substance-Related Disorders/th [Therapy]/Treatment Outcome.

RefID:2194. Stevens, J., Klima, J., Chisolm, D., and Kelleher, K. J.. A Trial of Telephone Services to Increase Adolescent Utilization of Health Care for Psychosocial Problems. Journal of Adolescent Health 2009. 45 (6) 564-570. **Keywords:** *adolescence/Adolescent/article/controlled study/Depression/Female/*health care utilization/human/interview/major clinical study/Male/mental health service/Motivation/outpatient department/primary medical care/priority journal/*psychosocial disorder/Self Report/substance abuse/Suicidal Ideation/*Telephone.

RefID:7351. Naar-King, S., Parsons, J. T., Murphy, D., Kolmodin, K., and Harris, D. R.. A multisite randomized trial of a motivational intervention targeting multiple risks in youth living with HIV: initial effects on motivation, self-efficacy, and depression. Journal of Adolescent Health 2010. 46 (5) 422-428. **Keywords:** CINAHL/Depression/HIV-Infected Patients -- In Adolescence/Motivation/Risk Taking Behavior -- Prevention and Control -- In Adolescence/Self-

Efficacy/adolescence/Analysis of Variance/Behavioral Changes/Brief Symptom Inventory/California/Clinical Trials/Coefficient Alpha/convenience sample/Data Collection,Computer Assisted/Descriptive Statistics/Female/Florida/Funding Source/HIV Infections -- Therapy/human/Interviews/Intraclass Correlation Coefficient/Male/Maryland/Medication Compliance/Michigan/Multicenter Studies/Multidisciplinary Care Team/Pennsylvania/Pretest-Posttest Design/Random Assignment/Repeated Measures/Scales/sexuality/substance abuse/Treatment Outcomes/Young Adult.

RefID:10601. Aalborg, Annette E., Miller, Brenda A., Husson, Gail, Byrnes, Hilary F., Bauman, Karl E., and Spoth, Richard L.. Implementation of Adolescent Family-Based Substance Use Prevention Programmes in Health Care Settings: Comparisons across Conditions and Programmes. Health Education Journal 2012. 71 (1) 53-61. **Keywords:** Family Programs/substance abuse/Control Groups/Health Services/prevention/Program Effectiveness/Comparative Analysis/Program Implementation/Children/Databases/Case Studies/ERIC RCTS.

RefID:10605. Joe, George W., Knight, Kevin, Simpson, D. Dwayne, Flynn, Patrick M., Morey, Janis T., Bartholomew, Norma G., Tindall, Michele Staton, Burdon, William M., Hall, Elizabeth A., Martin, Steve S., and O'Connell, Daniel J.. An Evaluation of Six Brief Interventions that Target Drug-Related Problems in Correctional Populations. Journal of Offender Rehabilitation 2012. 51 (1) 9-33. **Keywords:** sexuality/Health/Criminals/Multivariate Analysis/Social Networks/Institutionalized Persons/Correctional Institutions/drug abuse/Intervention/Psychological Patterns/Acquired Immunodeficiency Syndrome (AIDS)/Motivation/Cognitive Processes/Males/Knowledge Level/Attitude Change/Outcomes of Treatment/Mental Health/Attitude Measures/ERIC RCTS.

RefID:10789. Baer, J. S., Garrett, S. B., Beadnell, B., Wells, E. A., and Peterson, P. L.. Brief motivational intervention with homeless adolescents: evaluating effects on substance use and service utilization. Psychology of Addictive Behaviors 2007. 21 (4) 582-586. **Keywords:** Adolescent/Adult/Female/Homeless Persons/Humans/Male/Mental Health Services/utilization/Motivation/Substance-Related Disorders/epidemiology/rehabilitation/Time Factors/from SR bibliographies.

Level 2: BI targets other substances (eg, alcohol, nicotine, caffeine only)

RefID:2044. O'Leary-Barrett, M., Topper, L., MacKie, C. J., Castellanos-Ryan, N., Al-Khudhairy, N., and Conrod, P. J.. The adventure RCT: Effective delivery of personality-targeted interventions for substance misuse by educational professionals. Alcoholism: Clinical and Experimental Research 2011. 35 (#Issue#) 316A-#End Page#. **Keywords:**

*Personality/*Alcoholism/*society/human/drinking/school/Risk/student/Anxiety/cigarette smoking/Adolescent/United Kingdom/impulsiveness/substance abuse/Mental Health/high risk behavior/follow up/alcohol consumption/control group/Smoking/prevention/randomized controlled trial/teacher/sensation.

RefID:2047. Field, C., Caetano, R., Harris, T. R., Frankowski, F., and Roudsari, B.. Does subsequent treatment utilization among hispanics help explain improved drinking outcomes following brief intervention in the trauma department. Alcoholism: Clinical and Experimental Research 2011. 35 (#Issue#) 293A-#End Page#. **Keywords:** *Alcoholism/*drinking/*injury/*society/exposure/human/patient/substance abuse/drug dependence/hospital admission/follow up/ethnic group/population/randomized controlled trial/emergency health service/ethnic difference/Multivariate Analysis/alcohol.

RefID:2056. Clair, M., Martin, R., Stein, L. A. R., Lebeau, R., and Golembeske, C.. The impact of motivational interviewing on general and alcoholrelated predatory misbehaviors in incarcerated adolescents. Alcoholism: Clinical and Experimental Research 2011. 35 (#Issue#) 141A-#End Page#. **Keywords:** *human/*Adolescent/*Alcoholism/*society/Depression/Aggression/drinking/meditation/car/mood/prison/clinical trial/symptomatology/questionnaire/Center for Epidemiological Studies Depression Scale/high risk behavior/Crime/alcohol/Cannabis.

RefID:2167. Connell, A. M.. Employing complier average causal effect analytic methods to examine effects of randomized encouragement trials. American Journal of Drug and Alcohol Abuse 2009. 35 (4) 253-259. **Keywords:** Adolescent/Adult/article/Child/*complier average causal effect analysis/*data analysis/Family/family counseling/Family Therapy/Female/human/juvenile/major clinical study/Male/outcome assessment/outcomes research/Patient Compliance/prediction/sex difference/*substance abuse/*tobacco dependence.

RefID:2936. Cherpitel, Cheryl J., Bernstein, Edward, Bernstein, Judith, Moskalewicz, Jacek, and Swiatkiewicz, Grazyna. Screening, Brief Intervention and Referral to Treatment (SBIRT) in a Polish emergency room: Challenges in cultural translation of SBIRT. Journal of Addictions Nursing 2009. 20 (3) 127-131. **Keywords:** *alcohol abuse/*Drug Usage Screening/*Intervention/*nursing/*Sociocultural Factors/Emergency Services/Hospitals/Professional Referral.

RefID:3024. Godfrey, Christine, Heather, Nick, Bowie, Alison, Brodie, Jennifer, Parrott, Steve, Ashton, Heather, and McAvoy, Brian. Randomised controlled trial of two brief interventions against long-term benzodiazepine use: Cost-effectiveness. Addiction Research & Theory 2008. 16 (4) 309-317. **Keywords:** *Benzodiazepines/*Costs and Cost Analysis/*Intervention/*long term care/health care policy.

RefID:3613. Schoenwald, Sonja K., Ward, David M., Henggeler, Scott W., and Pickrel, Susan G.. Multisystemic therapy treatment of substance abusing or dependent adolescent offenders:

Costs of reducing incarceration, inpatient, and residential placement. Journal of Child and Family Studies 1996. 5 (4) 431-444. **Keywords:** *drug abuse/*Hospitalization/*Incarceration/*Residential Care Institutions/*Treatment/Followup Studies/Health Care Costs.

RefID:7290. Cherpitel, C. J., Korcha, R. A., Moskalewicz, J., Swiatkiewicz, G., Ye, Y., and Bond, J.. Screening, Brief Intervention, and Referral to Treatment (SBIRT): 12-Month Outcomes of a Randomized Controlled Clinical Trial in a Polish Emergency Department CHERPITEL ET AL. SCREENING, BRIEF INTERVENTION, AND REFERRAL TO TREATMENT. Alcoholism 2010. 34 (11) 1922-1928. **Keywords:** CINAHL/Alcoholism -- Prevention and Control/Alcoholism -- Therapy/Emergency Service/Clinical Trials/Poland/human/Motivational Interviewing/Treatment Outcomes/Referral and Consultation/Clinical Assessment Tools/T-Tests/Multivariate Statistics/Analysis of Variance/Male/Female/Adult/Paired T-Tests/Funding Source.

RefID:7760. Zanjani, F., Miller, B., Turiano, N., Ross, J., and Oslin, D.. Effectiveness of telephone-based referral care management, a brief intervention to improve psychiatric treatment engagement. Psychiatric Services 2008. 59 (7) 776-781. **Keywords:** CINAHL/Depression -- Therapy/Patient Compliance/Psychotherapy,Brief -- Methods/Substance Use Disorders -- Rehabilitation/Telephone/Adult/Aged/Aged,80 and over/chi square test/Clinical Trials/effect size/Female/Funding Source/Interviews/Male/Middle Age/P-Value/Pennsylvania/Power Analysis/Psychological Tests/Random Assignment/Record Review/human.

Level 2: BI delivered in a group (2+ people receiving) or online/computer intervention without video (text-only)

RefID:317. Conrod, Patricia J., Castellanos-Ryan, Natalie, and Strang, John. Brief, personality-targeted coping skills interventions and survival as a non-drug user over a 2-year period during adolescence. Archives of general psychiatry 2010. 67 (1) 85-93. **Keywords:** *Adaptation,Psychological/Adolescent/Cognitive Therapy/mt [Methods]/Female/Focus Groups/mt [Methods]/Follow-Up Studies/Humans/London/ep [Epidemiology]/Longitudinal Studies/Male/*Personality/cl [Classification]/Personality Inventory/Prevalence/*Psychotherapy,Group/mt [Methods]/Questionnaires/Risk Factors/Street Drugs/cl [Classification]/Substance Abuse Detection/Substance-Related Disorders/ep [Epidemiology]/*Substance-Related Disorders/pc [Prevention & Control]/Substance-Related Disorders/px [Psychology]/Survival Analysis..

RefID:997. Ondersma, Steven J., Chase, Sara K., Svikis, Dace S., and Schuster, Charles R.. Computer-based brief motivational intervention for perinatal drug use. Journal of substance abuse treatment 2005. 28 (4) 305-312. **Keywords:** Adult/Female/Follow-Up Studies/Humans/*Motivation/Pilot Projects/*Postpartum

Period/Pregnancy/Software/*Substance-Related Disorders/pc [Prevention & Control]/*Therapy,Computer-Assisted.

Level 3: Population not universally screened

RefID:226. Blow, Frederic C., Walton, Maureen A., Murray, Regan, Cunningham, Rebecca M., Chermack, Stephen T., Barry, Kristen L., Ilgen, Mark A., and Booth, Brenda M.. Intervention attendance among emergency department patients with alcohol- and drug-use disorders. Journal of studies on alcohol and drugs 2010. 71 (5) 713-719. **Keywords:** Adult/*Emergency Service,Hospital/td [Trends]/Female/Humans/Male/Middle Aged/*Patient Compliance/px [Psychology]/*Substance-Related Disorders/px [Psychology]/*Substance-Related Disorders/th [Therapy]/*Urban Population/td [Trends]/Young Adult.

RefID:566. D'Amico, Elizabeth J., Miles, Jeremy N. V., Stern, Stefanie A., and Meredith, Lisa S.. Brief motivational interviewing for teens at risk of substance use consequences: a randomized pilot study in a primary care clinic. Journal of substance abuse treatment 2008. 35 (1) 53-61. **Keywords:** Adolescent/Child/Female/Humans/Interviews as Topic/Male/*Motivation/Pilot Projects/Primary Health Care/*Substance-Related Disorders/et [Etiology]/Substance-Related Disorders/px [Psychology].

RefID:582. Naar-King, Sylvie, Lam, Phebe, Wang, Bo, Wright, Kathryn, Parsons, Jeffrey T., and Frey, Maureen A.. Brief report: maintenance of effects of motivational enhancement therapy to improve risk behaviors and HIV-related Health in a randomized controlled trial of youth living with HIV. Journal of pediatric psychology 2008. 33 (4) 441-445. **Keywords:** Adolescent/Adult/*Choice Behavior/Female/Follow-Up Studies/*HIV Infections/ep [Epidemiology]/*HIV Infections/px [Psychology]/*Health Behavior/Humans/Male/*Motivation/Patient Care Team/Questionnaires/*Risk-Taking/Substance-Related Disorders/ep [Epidemiology]/Substance-Related Disorders/pc [Prevention & Control]/Unsafe Sex/sn [Statistics & Numerical Data].

RefID:899. White, Helene R., Morgan, Thomas J., Pugh, Lisa A., Celinska, Katarzyna, Labouvie, Erich W., and Pandina, Robert J.. Evaluating two brief substance-use interventions for mandated college students. Journal of studies on alcohol 2006. 67 (2) 309-317. **Keywords:** Adolescent/Adult/Alcohol Drinking/lj [Legislation & Jurisprudence]/*Alcohol Drinking/pc [Prevention & Control]/Alcohol Drinking/px [Psychology]/Behavior Therapy/lj [Legislation & Jurisprudence]/*Behavior Therapy/Combined Modality Therapy/*Feedback/Female/Follow-Up Studies/Humans/*Interview,Psychological/Male/*Marijuana Abuse/pc [Prevention & Control]/Marijuana Abuse/px [Psychology]/*Motivation/Psychotherapy,Brief/lj [Legislation & Jurisprudence]/*Psychotherapy,Brief/Referral and Consultation/lj [Legislation & Jurisprudence]/*Referral and Consultation/Students/lj [Legislation & Jurisprudence]/*Students/px [Psychology].

RefID:1704. Baker, A., Kochan, N., Dixon, J., Heather, N., and Wodak, A.. Controlled evaluation of a brief intervention for HIV prevention among injecting drug users not in treatment. AIDS care 1994. 6 (5) 559-570. **Keywords:** Adolescent/Adult/*Cognitive Therapy/Female/Follow-Up Studies/*HIV Infections/pc [Prevention & Control]/HIV Infections/px [Psychology]/HIV Infections/tm [Transmission]/*Health Behavior/*Health Knowledge,Attitudes,Practice/Humans/Male/Middle Aged/Needle Sharing/ae [Adverse Effects]/Needle Sharing/px [Psychology]/*Psychotherapy,Brief/Sexual Behavior/Substance Abuse,Intravenous/px [Psychology]/*Substance Abuse,Intravenous/rh [Rehabilitation].

RefID:2934. Bradley, Elizabeth Gates. The effects of a school-based motivational intervention on adolescent substance abuse. Dissertation Abstracts International Section A: Humanities and Social Sciences 2009. 70 (6-A) 1917-#End Page#. **Keywords:** *drug abuse/*health/*Motivation/*School Based Intervention/*Schools/Intervention.

RefID:8169. Masson, C. L., Sorensen, J. L., Perlman, D. C., Shopshire, M. S., Delucchi, K. L., Chen, T., Sporer, K., Des Jarlais, D., and Hall, S. M.. Hospital- versus community-based syringe exchange: a randomized controlled trial. AIDS Education & Prevention 2007. 19 (2) 97-110. **Keywords:** CINAHL/Community Health Services/Hospitals,Community/Needle Exchange Programs -- Administration/Adult/California/chi square test/Clinical Assessment Tools/Randomized Controlled Trials/Confidence Intervals/Descriptive Statistics/Female/Funding Source/International Classification of Diseases/Male/Middle Age/Odds Ratio/P-Value/Pearson's Correlation Coefficient/Poisson Distribution/Questionnaires/Record Review/Short Form-36 Health Survey (SF-36)/human.

Level 3: Study population <12 years of age

RefID:9456. Marsiglia, F. F., Holleran, L., and Jackson, K. M.. Assessing the effect of external resources on school-based substance abuse prevention programs. Social Work in Education 2000. 22 (3) 145-161. **Keywords:** CINAHL/School Health Services/Substance Abuse -- Prevention and Control/Student Attitudes/Clinical Trials/P-Value/Experimental Studies/Surveys/Random Assignment/multivariate analysis of variance/T-Tests/Logistic Regression/Descriptive Statistics/Odds Ratio/Self Report/Child/adolescence/Adult/Male/Female/human.

Level 3: Does not assess BI

RefID:69. Stein, L. A. R., Clair, Mary, Lebeau, Rebecca, Colby, Suzanne M., Barnett, Nancy P., Golembeske, Charles, and Monti, Peter M.. Motivational interviewing to reduce substance-related consequences: effects for incarcerated adolescents with depressed mood. Drug and

alcohol dependence 2011. 118 (2-3) 475-478. **Keywords:** Adolescent/*Behavior Therapy/mt [Methods]/Depression/px [Psychology]/*Depression/th [Therapy]/Female/Humans/Male/*Motivation/Prisoners/px [Psychology]/*Relaxation Therapy/mt [Methods]/Substance-Related Disorders/px [Psychology]/*Substance-Related Disorders/th [Therapy]/Treatment Outcome/Young Adult.

RefID:133. Stein, L. A. R., Lebeau, Rebecca, Colby, Suzanne M., Barnett, Nancy P., Golembeske, Charles, and Monti, Peter M.. Motivational interviewing for incarcerated adolescents: effects of depressive symptoms on reducing alcohol and marijuana use after release. Journal of studies on alcohol and drugs 2011. 72 (3) 497-506. **Keywords:** Adolescent/Alcohol Drinking/ep [Epidemiology]/*Alcohol Drinking/pc [Prevention & Control]/Depression/et [Etiology]/*Depression/pp [Physiopathology]/Female/Follow-Up Studies/Humans/*Interview,Psychological/mt [Methods]/Male/*Marijuana Abuse/rh [Rehabilitation]/Motivation/Prisoners/px [Psychology]/*Relaxation Therapy/mt [Methods]/Treatment Outcome.

RefID:5917. Milby, J. B., Schumacher, J. E., McNamara, C., Wallace, D., and Usdan, S.. Contingency management in effective treatment for dually diagnosed, cocaine abusing homeless persons. NIDA research monograph 2000. 180 (#Issue#) 33-#End Page#. **Keywords:** Central.

Level 3: Results not presented separately for non-medical use of psychoactive substances of interest

RefID:975. Tait, Robert J., Hulse, Gary K., Robertson, Suzanne I., and Sprivulis, Peter C.. Emergency department-based intervention with adolescent substance users: 12-month outcomes. Drug and alcohol dependence 2005. 79 (3) 359-363. **Keywords:** Adolescent/Cohort Studies/Community Mental Health Services/mt [Methods]/Continuity of Patient Care/*Emergency Service,Hospital/Female/Health Status/Humans/Male/Outcome Assessment (Health Care)/Questionnaires/Referral and Consultation/Substance Abuse Treatment Centers/mt [Methods]/Substance-Related Disorders/di [Diagnosis]/Substance-Related Disorders/px [Psychology]/*Substance-Related Disorders/rh [Rehabilitation]/Treatment Outcome.

RefID:976. Tait, Robert J. and Hulse, Gary K.. Adolescent substance use and hospital presentations: a record linkage assessment of 12-month outcomes. Drug and alcohol dependence 2005. 79 (3) 365-371. **Keywords:** Adolescent/Age Factors/Australia/ep [Epidemiology]/Child/Cohort Studies/Community Mental Health Services/mt [Methods]/*Emergency Service,Hospital/Female/Follow-Up Studies/*Hospitalization/Humans/Male/Medical Record Linkage/Outcome Assessment (Health Care)/Patient Admission/Prevalence/Proportional Hazards Models/Referral and Consultation/Substance Abuse Treatment Centers/mt [Methods]/Substance-Related

Disorders/ep [Epidemiology]/*Substance-Related Disorders/rh [Rehabilitation]/Substance-Related Disorders/th [Therapy]/Survival Analysis/Treatment Outcome.

RefID:1098. Tait, Robert J., Hulse, Gary K., and Robertson, Suzanne I.. Effectiveness of a brief-intervention and continuity of care in enhancing attendance for treatment by adolescent substance users. Drug and alcohol dependence 2004. 74 (3) 289-296. **Keywords:** Adolescent/Chi-Square Distribution/Child/*Continuity of Patient Care/sn [Statistics & Numerical Data]/Female/Follow-Up Studies/Humans/Male/Patient Compliance/px [Psychology]/Patient Compliance/sn [Statistics & Numerical Data]/*Substance Abuse Treatment Centers/sn [Statistics & Numerical Data]/*Substance-Related Disorders/ep [Epidemiology]/Substance-Related Disorders/px [Psychology]/*Substance-Related Disorders/th [Therapy].

RefID:7213. Goti, J., Diaz, R., Serrano, L., Gonzalez, L., Calvo, R., Gual, A., and Castro, J.. Brief intervention in substance-use among adolescent psychiatric patients: a randomized controlled trial. European child & adolescent psychiatry 2010. 19 (6) 503-511. **Keywords:** CINAHL/Adolescent Psychiatry/Substance Use Disorders -- In Adolescence/adolescence/analysis of covariance/DSM/Epidemiological Research/Funding Source/human/Intervention Trials/Male/Outcomes (Health Care)/Questionnaires/Random Assignment/Semi-Structured Interview/Severity of Illness/Wilcoxon Signed Rank Test.

Level 3: Other reason for exclusion

Companion/duplicate report of excluded study

RefID:105. Shetty, Vivek, Murphy, Debra A., Zigler, Corwin, Yamashita, Dennis Duke, and Belin, Thomas R.. Randomized controlled trial of personalized motivational interventions in substance using patients with facial injuries. Journal of oral and maxillofacial surgery : official journal of the American Association of Oral and Maxillofacial Surgeons 2011. 69 (9) 2396-2411. **Keywords:** Adolescent/Adult/Alcoholism/pc [Prevention & Control]/Analysis of Variance/Chi-Square Distribution/Counseling/Cultural Competency/Ethnic Groups/*Facial Injuries/et [Etiology]/Facial Injuries/pc [Prevention & Control]/Feedback/Female/Humans/Male/*Motivation/*Patient Acceptance of Health Care/Patient Education as Topic/Prospective Studies/Psychiatric Status Rating Scales/Secondary Prevention/Self Report/Single-Blind Method/Socioeconomic Factors/Statistics,Nonparametric/*Substance-Related Disorders/co [Complications]/*Substance-Related Disorders/pc [Prevention & Control]/Substance-Related Disorders/px [Psychology]/Treatment Outcome/Young Adult.

RefID:257. Goti, Javier, Diaz, Rosa, Serrano, Lourdes, Gonzalez, Laura, Calvo, Rosa, Gual, Antoni, and Castro, Josefina. Brief intervention in substance-use among adolescent psychiatric patients: a randomized controlled trial. European child & adolescent psychiatry 2010. 19 (6) 503-511. **Keywords:** Adolescent/Alcoholism/di [Diagnosis]/Alcoholism/ep [Epidemiology]/Alcoholism/rh [Rehabilitation]/Amphetamine-Related Disorders/di [Diagnosis]/Amphetamine-Related Disorders/ep [Epidemiology]/Amphetamine-Related Disorders/rh [Rehabilitation]/Attitude to

Health/*Behavior Therapy/mt [Methods]/Child/Cocaine-Related Disorders/di [Diagnosis]/Cocaine-Related Disorders/ep [Epidemiology]/*Cocaine-Related Disorders/rh [Rehabilitation]/Comorbidity/Counseling/mt [Methods]/*Counseling/Cross-Sectional Studies/Designer Drugs/Diagnosis,Dual (Psychiatry)/Female/*Hospitalization/Humans/Intention/Male/Marijuana Abuse/di [Diagnosis]/Marijuana Abuse/ep [Epidemiology]/*Marijuana Abuse/rh [Rehabilitation]/Mental Disorders/di [Diagnosis]/Mental Disorders/ep [Epidemiology]/*Mental Disorders/rh [Rehabilitation]/*Motivation/*Psychotherapy,Brief/mt [Methods]/Self Efficacy/Substance-Related Disorders/di [Diagnosis]/Substance-Related Disorders/ep [Epidemiology]/*Substance-Related Disorders/rh [Rehabilitation]/Treatment Outcome.

RefID:2141. Bonsack, C.. Motivational intervention to reduce cannabis use in young people with psychosis: A randomized controlled trial. Early Intervention in Psychiatry 2010. 4 (#Issue#) 159-#End Page#. **Keywords:** *psychosis/*human/*substance abuse/*Schizophrenia/*randomized controlled trial/*lifespan/follow up/patient/*Cannabis.

RefID:7535. Zahradnik, A., Otto, C., Crackau, B., Löhrmann, I., Bischof, G., John, U., and Rumpf, H.. Randomized controlled trial of a brief intervention for problematic prescription drug use in non-treatment-seeking patients. Addiction (Abingdon, England) 2009. 104 (1) 109-117. **Keywords:** CINAHL/Drugs,Prescription/Motivational Interviewing/Psychotherapy,Brief/Substance Abuse -- Therapy/Substance Dependence -- Therapy/Academic Medical Centers/adolescence/Adult/Aged/chi square test/Data Analysis Software/Descriptive Statistics/effect size/Feedback/Female/Funding Source/Germany/Hospitals/human/Interview Guides/Interviews/Male/Mann-Whitney U Test/Middle Age/Prospective Studies/Psychological Tests/Questionnaires/Random Assignment/Randomized Controlled Trials/Scales/Severity of Illness Indices/Substance Abuse -- Epidemiology/Substance Dependence -- Epidemiology/Treatment Outcomes.

RefID:7985. White, aR, Mun, E. Y., Pugh, L., and Morgan, T. J.. Long-term effects of brief substance use interventions for mandated college students: sleeper effects of an in-person personal feedback intervention. Alcoholism 2007. 31 (8) 1380-1391. **Keywords:** CINAHL/Psychotherapy,Brief/Substance Use Disorders -- Rehabilitation/adolescence/Adult/Ethanol -- Blood/Body Mass Index/Central Nervous System Depressants -- Blood/Clinical Trials/Diagnosis,Psychosocial/Feedback/Female/Male/Motivation/Prospective Studies/Questionnaires/Smoking -- Psychosocial Factors/Students/human.

Study design (Secondary analysis or observational)

RefID:418. Magill, Molly, Barnett, Nancy P., Apodaca, Timothy R., Rohsenow, Damaris J., and Monti, Peter M.. The role of marijuana use in brief motivational intervention with young adult drinkers treated in an emergency department. Journal of studies on alcohol and drugs 2009. 70 (3) 409-413. **Keywords:** Adolescent/*Alcohol Drinking/th [Therapy]/*Behavior Therapy/Emergency Service,Hospital/Female/Humans/Male/*Marijuana Smoking/th [Therapy]/Young Adult.

RefID:2586. Oliansky, D. M., Wildenhaus, K. J., Manlove, K., Arnold, T., and Schoener, E. P.. Effectiveness of brief interventions in reducing substance use among at- risk primary care patients in three community-based clinics. Substance Abuse 1997. 18 (3) 95-103. **Keywords:** Adolescent/Adult/article/clinical trial/community care/controlled clinical trial/controlled study/Demography/Female/follow up/high risk population/human/major clinical study/Male/primary medical care/randomized controlled trial/*substance abuse.

Protocol for a terminated study (never completed due to recruitment problems)

RefID:5692. Copeland, J., Swift, W., Howard, J., Roffman, R. A., Stephens, R. S., and Berghuis, J.. A randomized controlled trial of brief interventions for cannabis problems among young offenders [abstract]. Drug and alcohol dependence 2001. 63 Suppl 1 (#Issue#) 32-#End Page#. **Keywords:** Central.

Level 4: No response from one or more authors regarding eligibility

RefID:7035. Shetty, V., Murphy, D. A., Zigler, C., Yamashita, D. D., and Belin, T. R.. Randomized controlled trial of personalized motivational interventions in substance using patients with facial injuries. Journal of Oral & Maxillofacial Surgery (02782391) 2011. 69 (9) 2396-2411. **Keywords:** CINAHL/Facial Injuries -- Etiology/Motivation/Patient Attitudes/Substance Use Disorders -- Complications/Substance Use Disorders -- Prevention and Control/adolescence/Adult/Alcoholism -- Prevention and Control/Analysis of Variance/chi square test/Counseling/Cultural Competence/Ethnic Groups/Facial Injuries -- Prevention and Control/Feedback/Female/human/Male/Nonparametric Statistics/patient education/Prospective Studies/Psychological Tests/Randomized Controlled Trials/Self Report/Single-Blind Studies/Socioeconomic Factors/Substance Use Disorders -- Psychosocial Factors/Treatment Outcomes/Young Adult.

Level 4: Conference abstract that should be tracked for future available data

RefID:2053. Massey, L. S., Walton, M. A., Booth, B. M., Barry, K. L., Cunningham, R. M., Chermack, S. T., and Blow, F. C.. Health services interventions for patients in the emergency department with alcohol and/or drug use disorders: Findings from project health link. Alcoholism: Clinical and Experimental Research 2011. 35 (#Issue#) 144A-#End Page#. **Keywords:** *human/*society/*patient/*emergency ward/*drug use/*Alcoholism/*health/*health service/substance abuse/Case Management/city/African American/alcohol consumption/logistic regression analysis/gender/screening/United States/early

intervention/abuse/model/Male/*alcohol/Cannabis/central stimulant agent.
