# Supplementary material for: Effectiveness of brief interventions as part of the Screening, Brief Intervention and Referral to Treatment (SBIRT) model for reducing the nonmedical use of psychoactive substances: a systematic review
Source: Syst Rev. 2014 May 24;3:50. doi: 10.1186/2046-4053-3-50 (PMC4042132; doi:10.1186/2046-4053-3-50)
Supplement: Additional file 8 — Risk of bias assessments for included studies. [file 2046-4053-3-50-S8.pdf]

Additional file 8. Risk of bias assessments for included studies.

	BI vs. no BI		BI vs. written info		
	Baer 2007	Humeniuk 2008	Bernstein 2009	Zahradnik 2008	Bernstein 2005
Random sequence generation	(+)	(+)	(+)	(?)	(+)
Allocation concealment	(?)	(+)	(?)	(?)	(+)
Blinding participants and personnel	(−)	(−)	(−)	(−)	(−)
Blinding assessors – Substance use	-	-	(?)	(?)	(+)
Blinding assessors – Frequency of use	(−)	-	(?)	-	-
Blinding assessors – Quantity of use	-	-	-	(?)	(+)
Blinding assessors – Harms/negative consequences	-	-	(?)	-	-
Blinding assessors – Positive behaviour change	-	-	(?)	-	-
Blinding assessors – Decision to attend treatment	-	-	-	-	(−) [a]
Blinding assessors – Composite outcomes	-	(−)	-	-	-
Blinding assessors – Use of different substances	-	-	-	-	(+) [a]
Blinding assessors – Intention to reduce use	-	-	-	-	-
Blinding assessors – Other health measures (objective)	(+)	-	-	-	-
Blinding assessors – Other health measures (subjective)	(−)	-	(?)	-	(−)
Blinding assessors – Other adverse outcomes	-	-	-	-	-
Incomplete outcome data – Substance use	-	-	(−)	(+)	(?)
Incomplete outcome data – Frequency of use	(−)	-	(−)	-	-
Incomplete outcome data – Quantity of use	-	-	-	(?)	(?)
Incomplete outcome data – Harms/negative consequences	-	-	(−)	-	-
Incomplete outcome data – Positive behaviour change	-	-	(−)	-	-
Incomplete outcome data – Decision to attend treatment	-	-	-	-	(−) [a]
Incomplete outcome data – Composite outcomes	-	(?)	-	-	-
Incomplete outcome data – Use of different substances	-	-	-	-	(?) [a]
Incomplete outcome data – Intention to reduce use	-	-	-	-	-
Incomplete outcome data – Other health measures (objective)	(−)	-	-	-	-
Incomplete outcome data – Other health measures (subjective)	(−)	-	(−)	-	(?)
Incomplete outcome data – Other adverse outcomes	-	-	-	-	-
Selective reporting	(−)	(−)	(?)	(?)	(−)
Other bias	(−)	(?)	(+)	(?)	(−)

Abbreviations: BI=brief intervention; info=information; vs.= versus. Outcomes not reported in a study denoted by "-".
[a]Risk of bias assessed by outcome data poorly reported by authors.

Legend: (+) = low risk of bias, (?) = unclear risk of bias, (−) = high risk of bia
