# Supplementary material for: Effectiveness of brief interventions as part of the Screening, Brief Intervention and Referral to Treatment (SBIRT) model for reducing the nonmedical use of psychoactive substances: a systematic review
Source: Syst Rev. 2014 May 24;3:50. doi: 10.1186/2046-4053-3-50 (PMC4042132; doi:10.1186/2046-4053-3-50)
Supplement: Additional file 9 — Supports for risk of bias assessments for included studies. [file 2046-4053-3-50-S9.pdf]

Additional file 9. *Supports for risk of bias assessments for included studies*

| **Baer, 2007** | | | |
|---|---|---|---|
| **Risk of bias item** | **Outcome** | **Judgment** | **Support for judgment** |
| Random sequence generation | N/A | Low risk | Quote: "randomly assigned to receive either a BMI or treatment-as-usual using an urn randomization program (Stout, Wirtz, Carboni, & DelBoca, 1994) balancing for gender and ethnicity (minority vs. nonminority). Randomization was unbalanced during the course of the study to increase experimental power to evaluate differences in response within the BMI group with a final ratio of 3 to 2 (n=75 vs. n=52) receiving the intervention while maintaining the original urn variables (Berghold, 2005)." |
| Allocation concealment | N/A | Unclear risk | Quote: "randomly assigned to receive either a BMI or treatment-as-usual using an urn randomization program (Stout, Wirtz, Carboni, & DelBoca, 1994) balancing for gender and ethnicity (minority vs. nonminority). Randomization was unbalanced during the course of the study to increase experimental power to evaluate differences in response within the BMI group with a final ratio of 3 to 2 (n=75 vs. n=52) receiving the intervention while maintaining the original urn variables (Berghold, 2005)." Comment: no information on how the sequence was concealed. |
| Blinding of participants and personnel | All outcomes | High risk | Quotes (report): "unblinding the experimental condition during assessment" and "All interviews were conducted by one of three master's-level clincians".<br><br>Comment: Counsellors were aware of what they were delivering to participants. Information provided by the author via personal communication indicates that participants were made aware of the study, the intent of the study, and allocation to which groups were possible during the consent process. |
| Blinding of outcome assessors | Frequency of use - Cannabis -1 month & 3 months. | High risk | Quotes: "The follow-up interviews were conducted by a clinician or project director who did not administer the BMI and baseline interview", "Participants who reported lifetime use of any of the substance categories were asked to recall their use across the prior 30 days using a modified time line follow-back procedure", "...days of cannabis...were calculated from the calendar". Comment: self-reported. Information provided by the |

| | | | |
|---|---|---|---|
| | | | author via personal communication indicates that participants were made aware of the study, the intent of the study, and allocation to which groups were possible during the consent process |
| | Frequency of use - Drug use other than tobacco, alcohol, cannabis-1 month & 3 months | High risk | Quotes: "The follow-up interviews were conducted by a clinician or project director who did not administer the BMI and baseline interview", "Participants who reported lifetime use of any of the substance categories were asked to recall their use across the prior 30 days using a modified time line follow-back procedure", "...days of cannabis...were calculated from the calendar". Comment: self-reported. Information provided by the author via personal communication indicates that participants were made aware of the study, the intent of the study, and allocation to which groups were possible during the consent process |
| | Other health measures (Days using drop-in centre services in the past 30 days - All Substance Use) - 1 month & 3 months | Low risk | Quote: "Two variables reflecting utilization of services at the collaborating agency were computed using a database maintained by the agency. This database was based on paper sign-in sheets for attendance at the drop-in center and staff memos reporting additional services. Variables for analysis included (a) number of visits in the prior 30 days to the drop-in center". |
| | Other health measures (Days using drop-in centre additional services in past 30 days - Any Substance Use) - 1 month & 3 months | Low risk | Quote: "Two variables reflecting utilization of services at the collaborating agency were computed using a database maintained by the agency. This database was based on paper sign-in sheets for attendance at the drop-in center and staff memos reporting additional services. Variables for analysis included...(b) the number of utilizations of additional services offered by the agency". |
| | Other health measures (Use of other agency services in past 30 days - Any Substance Use) - 1 month & 3 months | High risk | Quote: "In addition, self-report data were collected from youth regarding frequency of service utilization from various other agencies over the past 30 days". Comment: Information provided by the author via personal communication indicates that participants were made aware of the study, the intent of the study, and allocation to which groups were possible during the consent process. |
| Incomplete outcome data | Frequency of use - Cannabis -1 month & 3 months | High risk | 89 participants (70%) were analyzed at 1 month. Reasons for exclusion were provided only for a subset and not reported by group. |
| | Frequency of use - Drug use other than tobacco, | High risk | 89 participants (70%) were analyzed at 1 month. Reasons for exclusion |

| | | | |
|---|---|---|---|
| | alcohol, cannabis -1 month & 3 months | | were provided only for a subset and not reported by group. |
| | Other health measures (Days using drop-in centre services in the past 30 days - All Substance Use) - 1 month & 3 months | High risk | 89 participants (70%) were analyzed at 1 month. Reasons for exclusion were provided only for a subset and not reported by group. |
| | Other health measures (Days using drop-in centre additional services in past 30 days - Any Substance Use) - 1 month & 3 months | High risk | 89 participants (70%) were analyzed at 1 month. Reasons for exclusion were provided only for a subset and not reported by group. |
| | Other health measures (Use of other agency services in past 30 days - Any Substance Use) - 1 month & 3 months | High risk | 89 participants (70%) were analyzed at 1 month. Reasons for exclusion were provided only for a subset and not reported by group. |
| Selective reporting | N/A | High risk | For all outcomes, authors do not explicitly provide participants analyzed to permit inclusion in a potential meta-analysis. In addition, other outcomes in the report (e.g., counsellor ratings of engagement) were incompletely reported. |
| Other bias | N/A | High risk | Fidelity. More than half of the participants did not attend 4 sessions, with 39% attending 2 or less.<br><br>Single-centred study. Quote: "A sample of 127 homeless youth was recruited from a nonprofit, faith-based drop-in center." Comment: It is unclear to what extent this might have affected the effect estimates.<br><br>Study sponsorship. Government-funded study. |

| **Humeniuk, 2008** | | | |
|---|---|---|---|
| **RoB item** | **Outcome** | **Judgment** | **Support for judgment** |

| | | | |
|---|---|---|---|
| Random sequence generation | | Low risk | Quote: "Eligible participants were randomized to either an intervention or wait list control group immediately following the ASSIST baseline interview. Randomization was stratified by gender, substance and level of use (high/low)...Randomization lists for each drug category and country were prepared by the coordinating centre in Australia using a web-based randomization programme (http://www.randomization.com/)". (except from Humeniuk Addiction 2011) |
| Allocation concealment | | Low risk | Quote (from reports): "Eligible participants were randomized to either an intervention or wait list control group immediately following the ASSIST baseline interview." and "Clinical interviewers were trained by the study coordinators at each site to administer the ASSIST and brief intervention."<br><br>Quote (from author correspondence): "When the randomisation (surrepticiously) occurred...interviewers had no idea, up to this point, to which group the participant would be randomised. If the participant was randomised to the intervention group, then the intervention flowed on as seemlessly as possible from the ASSIST questionnaire, without it being too obvious to the participant that they had just been randomised!"<br><br>Comment: it is reasonable to assume that a person other than the clinical interviewer allocated participants, and this was done quickly, with little opportunity to influence allocation assignment. |
| Blinding of participants and personnel | All outcomes | High risk | Quote (from report): "Clinical research staff were not blind to the intervention allocation, as they were responsible for adminstering the intervention as baseline".<br><br>Quotes (from correspondence from author regarding study information provided to participants at the time of consent): "The purpose of the research is to learn how people answer questions about their experiences with tobacco, alcohol, medicines and other drugs and how they respond to getting feedback and information about their substance use. You may or may not get information and feedback after you have answered the interviewer's questions" and "It is also concerned with how people respond to some brief information given to them about their drug use...People in one group will receive feedback...will be given some written information to take home with them. People in the other group will not receive feedback or written information." Comment: participants' self-report to answers. Participants |

| | | | |
|---|---|---|---|
| | | | were made aware of the study, the intent of the study, and allocation to which groups were possible. |
| Blinding of outcome assessors | Composite outcome - 3 months | High risk | Quotes (correspondence from author regarding study information provided to participants at the time of consent): "The purpose of the research is to learn how people answer questions about their experiences with tobacco, alcohol, medicines and other drugs and how they respond to getting feedback and information about their substance use. You may or may not get information and feedback after you have answered the interviewer's questions" and "It is also concerned with how people respond to some brief information given to them about their drug use...People in one group will receive feedback...will be given some written information to take home with them. People in the other group will not receive feedback or written information." Comment: participants' self-report to answers. Participants were made aware of the study, the intent of the study, and allocation to which groups were possible. |
| Incomplete outcome data | Composite outcome - Total illicit substance involvement - 3 months | Unclear risk | 13% of participants were lost to follow-up, and authors conducted analyses based on last outcome carried forward. Authors state reasons for lost to follow-up are unknown. |
| | Composite outcome - Cannabis - 3 months | Unclear risk | It is unclear how many lost to follow-up were with cannabis use specifically, and reasons for attrition are unknown. Authors conducted analyses based on last outcome carried forward. |
| | Composite outcome - Stimulants (amphetamine-type and cocaine) - 3 months | Unclear risk | It is unclear how many lost to follow-up were with stimulant use specifically, and reasons for attrition are unknown. Authors conducted analyses based on last outcome carried forward. |
| | Composite outcome - Opioids - 3 months | Unclear risk | It is unclear how many lost to follow-up were with opioid use specifically, and reasons for attrition are unknown. Authors conducted analyses based on last outcome carried forward. |
| | Composite outcome - Inhalants - 3 months | Unclear risk | It is unclear how many lost to follow-up were with inhalant use specifically, and reasons for attrition are unknown. Authors conducted analyses based on |

| | | | |
|---|---|---|---|
| | | | last outcome carried forward. |
| | Composite outcome - Sedatives - 3 months | Unclear risk | It is unclear how many lost to follow-up were with sedative use specifically, and reasons for attrition are unknown. Authors conducted analyses based on last outcome carried forward. |
| | Composite outcome - Hallucinogens - 3 months | Unclear risk | It is unclear how many lost to follow-up were with hallucinogen use specifically, and reasons for attrition are unknown. Authors conducted analyses based on last outcome carried forward. |
| Selective reporting | | High risk | Authors should have reported the individuals components of the composite measure. The outcome addressing general health was not specified in the methods section; the study protocol was not sufficiently detailed to assess for selective reporting. Authors should have reported a table of baseline characteristics between groups for readers to assess similarity. |
| Other bias | | Unclear risk | Fidelity. Session length varied statistically significantly among sites. Comment: Although we do not feel that the actual mean length across sites varied significantly from the original protocol, authors do not provide information as to how well they adhered to the intervention protocol in terms of content delivered.<br><br>Single-centre study. This was a multicentred study.<br><br>Study sponsorship. Government-funded study. The funder was a member of the study group and involved with coordination of the trial and report. |

| **Bernstein, 2009** | | | |
|---|---|---|---|
| **RoB item** | **Outcome** | **Judgment** | **Support for judgment** |
| Random sequence generation | | Low risk | Randomization was based on computer-generated random numbers in blocks of 100 stratified by age group (14-17 and 18-21). |
| Allocation concealment | | Unclear risk | A double opaque envelope system enabled blinding of the research assistants who performed the assessment to randomization status. It is unclear whether envelopes were also numbered and sealed. |

| | | | |
|---|---|---|---|
| Blinding of participants and personnel | All outcomes | High risk | Upon receiving the intervention, intervention providers would be aware of what was delivered. It is unclear if participants were made aware of the study, the intent of the study, or allocation to which groups were possible. |
| Blinding of outcome assessors | Substance use - abstinence - 3 months & 12 months | Unclear risk | Self-reported outcome. It is unclear if participants were made aware of the study, the intent of the study, or allocation to which groups were possible. |
| | Substance use - high on cannabis | Unclear risk | Self-reported outcome. It is unclear if participants were made aware of the study, the intent of the study, or allocation to which groups were possible. |
| | Frequency of use - days of consumption | Unclear risk | Self-reported outcome. It is unclear if participants were made aware of the study, the intent of the study, or allocation to which groups were possible. |
| | Use-related harms/consequences – carried a weapon | Unclear risk | Self-reported outcome. It is unclear if participants were made aware of the study, the intent of the study, or allocation to which groups were possible. |
| | Use-related harms/consequences – drove a car after using cannabis | Unclear risk | Self-reported outcome. It is unclear if participants were made aware of the study, the intent of the study, or allocation to which groups were possible. |
| | Use-related harms/consequences – rode in a car with a person drunk/high | Unclear risk | Self-reported outcome. It is unclear if participants were made aware of the study, the intent of the study, or allocation to which groups were possible. |
| | Positive behaviour change – tried to cut back on cannabis use | Unclear risk | Self-reported outcome. It is unclear if participants were made aware of the study, the intent of the study, or allocation to which groups were possible. |
| | Positive behaviour change – tried to stop using cannabis | Unclear risk | Self-reported outcome. It is unclear if participants were made aware of the study, the intent of the study, or allocation to which groups were possible. |
| | Positive behaviour change – tried to be careful about situations when using cannabis | Unclear risk | Self-reported outcome. It is unclear if participants were made aware of the study, the intent of the study, or allocation to which groups were possible. |
| | Other health measures – felt unsafe in the past 30 days | Unclear risk | Self-reported outcome. It is unclear if participants were made aware of the study, the intent of the study, or allocation to which groups were possible. |
| Incomplete outcome data | Substance use - abstinence - 3 months & 12 months | High risk | Differential loss-to-follow-up between groups, and proportion of missing outcomes to event risk warrants concern for bias. No reasons for loss-to-follow-up provided for groups. |
| | Substance use - high on cannabis - 3 months & 12 months | High risk | Differential loss-to-follow-up between groups, and proportion of missing outcomes to event risk warrants concern for bias. No reasons for loss-to-follow-up provided for groups. |

| | | | |
|---|---|---|---|
| | Frequency of use - days of consumption | High risk | Differential loss-to-follow-up between groups, and proportion of missing outcomes to event risk warrants concern for bias. No reasons for loss-to-follow-up provided for groups. |
| | Use-related harms/consequences – carried a weapon | High risk | Differential loss-to-follow-up between groups, and proportion of missing outcomes to event risk warrants concern for bias. No reasons for loss-to-follow-up provided for groups. |
| | Use-related harms/consequences – drove a car after using cannabis | High risk | Differential loss-to-follow-up between groups, and proportion of missing outcomes to event risk warrants concern for bias. No reasons for loss-to-follow-up provided for groups. |
| | Use-related harms/consequences – rode in a car with a person drunk/high | High risk | Differential loss-to-follow-up between groups, and proportion of missing outcomes to event risk warrants concern for bias. No reasons for loss-to-follow-up provided for groups. |
| | Positive behaviour change – tried to cut back on cannabis use | High risk | Differential loss-to-follow-up between groups, and proportion of missing outcomes to event risk warrants concern for bias. No reasons for loss-to-follow-up provided for groups. |
| | Positive behaviour change – tried to stop using cannabis | High risk | Differential loss-to-follow-up between groups, and proportion of missing outcomes to event risk warrants concern for bias. No reasons for loss-to-follow-up provided for groups. |
| | Positive behaviour change – tried to be careful about situations when using cannabis | High risk | Differential loss-to-follow-up between groups, and proportion of missing outcomes to event risk warrants concern for bias. No reasons for loss-to-follow-up provided for groups. |
| | Other health measures – felt unsafe in the past 30 days | High risk | Differential loss-to-follow-up between groups, and proportion of missing outcomes to event risk warrants concern for bias. No reasons for loss-to-follow-up provided for groups. |
| Selective reporting | | Unclear risk | No protocol or trial registry information available to check a priori methods. |

| | | | |
|---|---|---|---|
| Other bias | | Low risk | Fidelity. Quote: "All scored Adherence Checklists met the required cut-off of 80 out of 100 points for fidelity."<br><br>Single-centred trial. Quote: "The study took place in the pediatric emergency department (PED) of an inner-city, academic hospital". It is unclear to what extent this might have affected the effect estimates.<br><br>Study sponsorship. Government-funded study. |

**Zahradnik, 2009**

| RoB item | Outcome | Judgment | Support for judgment |
|---|---|---|---|
| Random sequence generation | | Unclear risk | Quotes: “Patients were randomized by ward of admission and time-frame” , “Randomized by time-frame was applied to prevent contamination due to interactions between patients belonging to IG and CG”, “Exactly half of the time each ward was included in the study, study participants from that ward were allocated to IG [intervention group]”. It is unclear what method of randomization was used. |
| Allocation concealment | | Unclear risk | Quotes: “Patients were randomized by ward of admission and time-frame” “Exactly half of the time each ward was included in the study, study participants from that ward were allocated to IG [intervention group]”. No information is provided regarding what method was used to ensure the allocation process was not altered. |
| Blinding of participants and personnel | All outcomes | High risk | Upon receiving the intervention, intervention providers would be aware of what was delivered. It is unclear if participants were made aware of the study, the intent of the study, or allocation to which groups were possible. |
| Blinding of outcome assessors | Substance use - discontinuation (any drug, sedatives/ hypnotics, opioids) – 3 months & 12 months | Unclear risk | Quotes: “The follow-ups were conducted by telephone” “In cases of non-accessibility via telephone, participants were contacted personally at their homes”. Authors did not explicitly define whether outcomes were collected by participant self-report, but this is highly likely. It is unclear if participants were made aware of the study, the intent of the study, or allocation to which groups were possible. |
| | Substance use – reducing use >25% (any drug, | Unclear risk | Quotes: “The follow-ups were conducted by telephone” “In cases of non-accessibility via telephone, participants were contacted personally at their homes”. Authors did not explicitly define whether outcomes were collected by participant self-report, but this is |

| | | | |
|---|---|---|---|
| | sedatives/hypnotics, opioids) | | highly likely. It is unclear if participants were made aware of the study, the intent of the study, or allocation to which groups were possible. |
| | Quantity of use – reduction of the defined daily dosage - 3 months & 12 months | Unclear risk | Quotes: "The follow-ups were conducted by telephone" "In cases of non-accessibility via telephone, participants were contacted personally at their homes". Authors did not explicitly define whether outcomes were collected by participant self-report, but this is highly likely. It is unclear if participants were made aware of the study, the intent of the study, or allocation to which groups were possible. |
| Incomplete outcome data | Substance use - discontinuation (any drug) - 3 months & 12 months | Low risk | Few participants were lost-to-follow-up (n=8 control, n=1 treatment), and given that authors chose to infer a worst-case scenario for those individuals, which may be realistic given that these dropouts may have been related to the true outcome, there is likely little bias occurring. |
| | Substance use - discontinuation (sedatives/ hypnotics) - 3 months & 12 months | Low risk | Although numbers for lost-to-follow-up were not provided specifically for this drug category, few participants were lost-to-follow-up overall in the study. Authors chose to infer a worst-case scenario for missing individuals, which may be realistic and likely little bias is occurring. |
| | Substance use - discontinuation (opioids) - - 3 months & 12 months | Low risk | Although numbers for lost-to-follow-up were not provided specifically for this drug category, few participants were lost-to-follow-up overall in the study. Authors chose to infer a worst-case scenario for missing individuals, which may be realistic and likely little bias is occurring. |
| | Substance use – reducing use >25% (any drug) | Low risk | Few participants were lost-to-follow-up (n=8 control, n=1 treatment), and given that authors chose to infer a worst-case scenario for those individuals, which may be realistic given that these dropouts may have been related to the true outcome, there is likely little bias occurring. |
| | Substance use – reducing use >25% (sedatives/ hypnotics) | Low risk | Although numbers for lost-to-follow-up were not provided specifically for this drug category, few participants were lost-to-follow-up overall in the study. Authors chose to infer a worst-case scenario for missing individuals, which may be realistic and likely little bias is occurring. |
| | Substance use – reducing use >25% (opioids) | Low risk | Although numbers for lost-to-follow-up were not provided specifically for this drug category, few participants were lost-to-follow-up overall in the study. Authors chose to infer a worst-case scenario for missing individuals, which may be realistic and likely little bias is occurring. |

| | | | |
|---|---|---|---|
| | Quantity of use – reduction of the defined daily dosage at<br>3 and 12 mo | Unclear risk | Authors do not provide the number of individuals analyzed. |
| Selective reporting | | Unclear risk | Consulted against trial registry information (NCT00514839). No change in listed primary outcomes but secondary outcomes were not listed. |
| Other bias | | Unclear risk | Fidelity. Despite stating procedures for addressing fidelity in the methods section, the authors provide no information regarding the results of those procedures. It is unclear to what extent the intervention and control groups were implemented as planned.<br><br>Single-centred trial. Quote: “All patients...admitted recently to an internal, surgical or gynaecological ward of either a general hospital or a university hospital”.<br><br>Recruitment bias. No information was provided regarding this item. It is unclear whether recruitment bias is a factor in the design of this study.<br>Study sponsorship. Government-funded study. |

| **Bernstein, 2005** | | | |
|---|---|---|---|
| **RoB item** | **Outcome** | **Judgment** | **Support for judgment** |
| Random sequence generation | | Low risk | Quote: "Cards generated by a computerized randomization program (in blocks of ten) were sealed in opaque envelopes and used in numerical order." |
| Allocation concealment | | Low risk | Quote: "Cards generated by a computerized randomization program (in blocks of ten) were sealed in opaque envelopes and used in numerical order." |

| | | | |
|---|---|---|---|
| Blinding of participants and personnel | All outcomes | High Risk | Quotes: "Health care providers, RAs and enrollees were all blinded to randomization status. The interventionist, who knew the enrollee's allocation, did not participate in the follow-up process. Because the intervention consisted of a conversation at the end of the assessment process, enrollees were not made explicitly aware of their own status." And “Eligible patients were offered enrollment in a study to test the value of a brief conversation about their drug use.” Comment: Inteventionists would have known what they were delivering to the individual. Participants may have known what they received. |
| Blinding of outcome assessors | Substance use - Cocaine - 6 months | Low risk | Abstinence in the previous 30 days determined through biochemical analysis and defined as <5 ng/10 mg hair for cocaine. |
| | Substance use - Opioids - 6 months | Low risk | Abstinence in the previous 30 days determined through biochemical analysis and defined as <2 ng/10 mg hair for opioids. |
| | Substance use - Cocaine and opioids - 6 months | Low risk | Abstinence in the previous 30 days determined through biochemical analysis and defined as <5 ng/10 mg hair for cocaine and <2 ng/10 mg hair for opioids. |
| | Quantity of use - Cocaine - 6 months | Low risk | Abstinence in the previous 30 days determined through biochemical analysis and defined as <5 ng/10 mg hair for cocaine. Assessed as change from baseline. |
| | Quantity of use - Opioids - 6 months | Low risk | Abstinence in the previous 30 days determined through biochemical analysis and defined as <2 ng/10 mg hair for opioids. Assessed as change from baseline. |
| | Decision to attend treatment | High Risk | Although authors state self-report results were confirmed by analysis of the Massachusetts state treatment database, this would have covered only the 'substance abuse treatment' mode and not others reported by participants, such as 'family' or 'alcoholics anonymous'. |
| | Use of different substances | Low risk | Quotes: "Because we were interested in capturing cross-over use...we assayed both drugs in all participants who were positive for either drug at baseline, and did not restrict our follow-up analysis to the drug of choice at entry", "abstinence was defined per laboratory standard as <5ng/10mg hair for cocaine and <2ng/10mg hair for opiates". |
| | Other health measures (change in ASI from baseline) - cocaine and/or opioids - 3 months & 6 months | High Risk | Authors state RAs, who administered the ASI at baseline and follow-up, were not aware of randomization status. Authors refer to this outcome in the Discussion as self-reported. Comment: RAs were probably blinded; however, participants provided answers to questions posed and they were not blinded to status. |

| | | | |
|---|---|---|---|
| Incomplete outcome data | Substance use - Cocaine - 6 months.<br><br>Substance use - Opioids - 6 months.<br><br>Substance use - Cocaine and opioids - 6 months | Unclear risk | Quotes: "Criteria for inclusion in analysis, however, included confirmation of that self-report with biochemical evidence of cocaine and heroin use...this decision was taken to assure the accuracy of the time frame for self-reported use. Furthermore, the IRB required that we explain to patients prior to screening that they might be eligible for a study about cocaine and heroin use, and there was concern that without biochemical confirmation there might be some instances of false report to obtain reimbursement" and "We were surprised to find, after enrollment and randomization, that 147 enrollees had no biochemical evidnece for use of these drugs in the past 30 days...we decided to change the eligibility criterion to presence of cocaine or heroin in hair...these subjects were dropped from the analysis strictly because of lack of eligibility, not because of their lack of compliance with intervention or because of data collected during follow-up." Comment: Probably reflective of what occurred. Just under 20% of participants of the entire randomized population were missing at follow-up, and reasons are not provided. |
| | Quantity of use - Cocaine - 6 months.<br><br>Quantity of use - Opioids - 6 months | Unclear risk | Quotes: "Criteria for inclusion in analysis, however, included confirmation of that self-report with biochemical evidence of cocaine and heroin use...this decision was taken to assure the accuracy of the time frame for self-reported use. Furthermore, the IRB required that we explain to patients prior to screening that they might be eligible for a study about cocaine and heroin use, and there was concern that without biochemical confirmation there might be some instances of false report to obtain reimbursement" and "We were surprised to find, after enrollment and randomization, that 147 enrollees had no biochemical evidnece for use of these drugs in the past 30 days...we decided to change the eligibility criterion to presence of cocaine or heroin in hair...these subjects were dropped from the analysis strictly because of lack of eligibility, not because of their lack of compliance with intervention or because of data collected during follow-up." Comment: Probably reflective of what occurred. Just under 20% of participants of the entire randomized population were missing at follow-up, and reasons are not provided. |
| | Decision to attend treatment | High risk | This outcome evaluated on only those who were abstinent. Data poorly reported. |
| | Use of different substances | Unclear risk | Incompletely reported to adjudicate. |
| | Other health measures (change in ASI from baseline) - cocaine and/or opioids - 3 months | Unclear risk | 76% of randomized participants provided data. Reasons for loss-to-follow-up were not provided. |

| | | | |
|---|---|---|---|
| | Other health measures (change in ASI from baseline) - cocaine and/or opioids - 6 months | Unclear risk | 81% of randomized participants provided data. Reasons for loss-to-follow-up were not provided. |
| Selective reporting | | High risk | Although authors state they restricted their analysis to those with biochemical confirmation, presenting the data based on self-report of cocaine or heroin use would have still been relevant to readers. For some outcomes, only p values provided. In addition, authors analyzed the ASI data base on all available participants, not just those with biochemical confirmation. |
| Other bias | | High risk | Fidelity: Although most received the complete intervention (90%), only a third received the booster call.<br><br>Single-centred trials. Quote: "The trial was conducted...in walk-in clinics at Boston Medical Center (Urgent care, Women's clinic, Homeless clinic). It is unclear to what extent this might have affected the effect estimates.<br><br>Study sponsorship. Government-funded study. |
