# Supplementary material for: Effectiveness of brief interventions as part of the Screening, Brief Intervention and Referral to Treatment (SBIRT) model for reducing the nonmedical use of psychoactive substances: a systematic review
Source: Syst Rev. 2014 May 24;3:50. doi: 10.1186/2046-4053-3-50 (PMC4042132; doi:10.1186/2046-4053-3-50)
Supplement: Additional file 10 — Additional data used for calculations or not reported in the main report. [file 2046-4053-3-50-S10.pdf]

Additional file 10. *Additional data used for calculations or not reported in the main report*

Outcome	Follow up	BI Mean (SD)		No BI Mean (SD)		Notes
		Baseline	Follow-up	Baseline	Follow-up	
PRIMARY OUTCOMES						
**Frequency of use**						
**Days of use – Cannabis** Self-report, past 30 d [1]	1 mo	17.4 (11.5)	13.7 (11.9)	19.1 (11.1)	13.0 (12.9)	Change from baseline in Table 5
	3 mo	17.4 (11.5)	14.8 (12.1)	19.1 (11.1)	13.2 (12.4)	
**Days of use - Other drugs** Self-report, past 30 d. [1]	1 mo	6.4 (8.6)	4.1 (5.9)	5.6 (8.3)	2.6 (3.8)	Change from baseline in Table 5
	3 mo	6.4 (8.6)	3.6 (5.6)	5.6 (8.3)	3.3 (5.9)	
**Days abstinent** Self-report, past 30 d. [1]	1 mo	8.7 (8.9)	12.4 (10.6)	9.2 (9.8)	15.6 (12.1)	Change from baseline in Table 5
	3 mo	8.7 (8.9)	11.4 (10.4)	9.2 (9.8)	15.2 (11.8)	
**Composite outcome**						
**ASSIST Tool Score – All substances [2]**	3 mo	36.7 (19.0)	28.9 (17.3)	36.9 (20.1)	32.3 (17.9)	Change from baseline from Table 5 of main report
**ASSIST Tool Score – Cannabis [2]**	3 mo	17.6 (7.0)	13.9 (9.0)	17.8 (6.7)	15.7 (8.1)	Additional information from original study report BI: n=176; no BI: n=152
**ASSIST Tool Score – Stimulants (amphetamine-type and cocaine) [2]**	3 mo	17.3 (7.4)	11.1 (8.6)	15.3 (7.3)	12.1 (8.6)	Additional information from original study report BI: n=110; no BI: n=119
**ASSIST Tool Score – Opioids [2]**	3 mo	22.7 (3.5)	21.9 (3.8)	13.1 (8.9)	16.3 (9.4)	Additional information from original study report BI: n=37; no BI: n=36
**ASSIST Tool Score – Inhalants [2]**	3 mo	4.9 (5.3)	2.8 (2.7)	5.7 (6.9)	3.7 (7.1)	Additional information from original study report BI: n=28; no BI: n=19
**ASSIST Tool Score – Sedatives [2]**	3 mo	8.8 (6.9)	2.9 (6.0)	10.0 (8.9)	4.3 (6.5)	Additional information from original study report

						BI: n=44; no BI: n=46
**ASSIST Tool Score – Hallucinogens [2]**	3 mo	4.7 (4.7)	2.1 (3.0)	5.2 (4.3)	2.2 (2.2)	Additional information from original study report<br>BI: n=39; no BI: n=37
**SECONDARY OUTCOMES**						
**Other health measures**						
**Use of drop-in centre services** Objective, past 30 d. [1]	1 mo	5.5 (5.4)	6.4 (6.5)	6.3 (5.4)	6.1 (5.7)	Change from baseline in Table 5
	3 mo	5.5 (5.4)	4.4 (6.1)	6.3 (5.4)	5.3 (6.8)	Change from baseline in Table 5
**Use of drop-in additional services** Objective, past 30 d. [1]	1 mo	0.5 (1.1)	0.5 (1.0)	0.5 (0.9)	1.0 (2.4)	Change from baseline in Table 5
	3 mo	0.5 (1.1)	0.6 (1.4)	0.5 (0.9)	0.4 (0.9)	Change from baseline in Table 5
**Use of other agency services** Self-report, past 30 d. [1]	1 mo	11.8 (19.2)	9.4 (12.4)	16.5 (23.3)	9.5 (14.7)	Change from baseline in Table 5
	3 mo	11.8 (19.2)	8.4 (10.7)	16.5 (23.3)	8.3 (10.5)	Change from baseline in Table 5
		**BI<br>Event rates (n)**		**Written information<br>Event rates (n)**		
**Substance use**						
Abstinence – Sedatives or hypnotics [3]	3 mo	18.5 % (27)		0% (28)		Sample sizes and group event rates provided by the author.
	12 mo	25.9% (27)		17.9% (28)		Sample sizes and group event rates provided by the author.
Abstinence – Opioids [3]	3 mo	14.7% (34)		12.0% (50)		Sample sizes and group event rates provided by the author.
	12 mo	20.6% (34)		18% (50)		Sample sizes and group event rates provided by

						the author.
**Reducing use >25% - Sedatives or hypnotics [3]**	3 mo	59.5% (27)		35.7% (28)		Sample sizes and group event rates provided by the author.
	12 mo	66.7% (27)		50.0% (28)		Sample sizes and group event rates provided by the author.
**Reducing use >25% - Opioids [3]**	3 mo	55.9% (34)		28.0% (50)		Sample sizes and group event rates provided by the author.
	12 mo	47.1% (34)		44.0% (50)		Sample sizes and group event rates provided by the author.
		**BI Mean (SD)**		**Written Information Mean (SD)**		
		Baseline	Follow-up	Baseline	Follow-up	
**Frequency of use**						
**Cannabis consumption [4]**	3 mo	19.1 (11.2)	14.2 (10.8)	14.5 (9.7)	13.7 (11.2)	Change from baseline in Table 6
	12 mo	18.1 (10.8)	11 (10.7)	15.0 (10.4)	13.2 (11.7)	Change from baseline in Table 6
**Quantity of use**						
**Defined daily dosage – Sedatives/hypnotics/opioids [3]**	3 mo	NR	NR	NR	NR	Only change from baseline reported (Table 6).
**Drug level – Cocaine (ng/10mg) [5]**	6 mo	616 (NR)	436 (NR)	485 (NR)	464 (NR)	Change from baseline in Table 6
**Drug level – Opioids (ng/10mg) [5]**	6 mo	26.4 (NR)	18.8 (NR)	30.7 (NR)	22.9 (NR)	Change from baseline in Table 6

References

1. Baer JS, Garrett SB, Beadnell B, Wells EA, Peterson PL: **Brief motivational intervention with homeless adolescents: evaluating effects on substance use and service utilization.** *Psychol Addict Behav* 2007, **21**(4):582-586.
2. Humeniuk R, Dennington V, Ali R: *The Effectiveness of a Brief Intervention for Illicit Drugs Linked to the Alcohol, Smoking and Substance Involvement Screening Test (ASSIST) in Primary Health Care Settings: A Technical Report of Phase III Findings of the WHO ASSIST Randomized Controlled Trial.* World Health Organization; 2008.
3. Zahradnik A, Otto C, Crackau B, Lohrmann I, Bischof G, John U, Rumpf HJ: **Randomized controlled trial of a brief intervention for problematic prescription drug use in non-treatment-seeking patients.** *Addiction* 2009, **104**(1):109-117.
4. Bernstein E, Edwards E, Dorfman D, Heeren T, Bliss C, Bernstein J: **Screening and brief intervention to reduce marijuana use among youth and young adults in a pediatric emergency department.** *Academic emergency medicine : official journal of the Society for Academic Emergency Medicine* 2009, **16**(11):1174-1185.
5. Bernstein J, Bernstein E, Tassiopoulos K, Heeren T, Levenson S, Hingson R: **Brief motivational intervention at a clinic visit reduces cocaine and heroin use.** *Drug and Alcohol Dependence* 2005, **77**(1):49-59.
