# Supplementary material for: Effectiveness of brief interventions as part of the Screening, Brief Intervention and Referral to Treatment (SBIRT) model for reducing the nonmedical use of psychoactive substances: a systematic review
Source: Syst Rev. 2014 May 24;3:50. doi: 10.1186/2046-4053-3-50 (PMC4042132; doi:10.1186/2046-4053-3-50)
Supplement: Additional file 11 — Quality of evidence tables. [file 2046-4053-3-50-S11.pdf]

Additional file 11: *Quality of evidence tables*

Quality of evidence domains for brief intervention **(BI) vs. no BI** in participants screened for at-risk substance use.

Outcome	Study Limitations (risk of bias)	Inconsistency	Indirectness	Imprecision	Publication bias
PRIMARY OUTCOMES					
**Substance use**	n/a	Not estimable	0 (0)	n/a	Not measured.
**Frequency of use [1]**<br>**• Use of Cannabis / Other drugs[1]**<br>**• Days abstinent**<br>Past 30-day use using a modified timeline follow-back interview.<br>**Follow-up: 1 and 3 months**	Very serious limitations (high risk of bias)	No serious limitations (1 study only)	No serious limitations	Serious limitations<br>One study assessed outcomes (lack of scientific replication). Less than 400 participants in analysis.	No serious limitations<br>This study small and NS, not likely to be other studies
**ASSIST score (composite outcome[2]) – All substances [2]** Sum score. Higher score interpreted as higher substance involvement (range 0 to 27+ points). **Follow-up: 3 months**	Serious limitations (medium risk of bias)	No serious limitations (1 study)	No serious limitations	Serious limitations<br>One study assessed this outcome (lack of scientific replication). Minimum important difference difficult to determine with the composite score.	No serious limitations<br>This study small and NS, not likely to be other studies
SECONDARY OUTCOMES					
**Other health measures**<br>• Use of drop-in services<br>• Use of additional services<br>• Use of other agency services	Very serious limitations (high risk of bias)	No serious limitations (1 study only)	No serious limitations	Serious limitations<br>One study assessed outcomes (lack of scientific replication). Less than 400 participants in analysis.	No serious limitations<br>This study small and NS, not likely to be other studies

Outcome	Study Limitations (risk of bias)	Inconsistency	Indirectness	Imprecision	Publication bias
**Follow-up: 1 and 3 mo [1]**					

*Abbreviations: ASSIST = Alcohol, Smoking and Substance Involvement Screening Test; CI = confidence interval; d=days; MD = mean difference; mo=months; RR = risk ratio.

Footnotes:

[1]Drugs other than tobacco, alcohol, cannabis were assessed.

[2]Composite outcome: substance use, frequency of use, use-related harms or negatives consequences, intention to reduce substance use, another person concerned with use, use of drug by injection.

Quality of evidence domains for **brief intervention vs. written information** in participants screened for at-risk substance use.

Outcome	Follow-up	Risk of bias	Inconsistency	Indirectness	imprecision	Publication bias
**PRIMARY OUTCOMES**						
**Substance use**						
**Abstinence – All substances**<br>Cannabis [3]; Any prescription drugs (Sedatives/hypnoti cs/opioids) [4].<br>Assessment: Self-report (past 30 days) and NR (past 3 months).	3 mo	Very serious limitations<br>[3] - medium risk<br>[4] - high risk	No serious limitations	No serious limitations	Very serious limitations<br>Total events <300 and sample size <2000. CI for one study included both appreciable harm and benefit. Most likely also the case with the unit-of-analysis error study if appropriate corrections made.	No serious limitations<br>Although a few number of studies located, mixed results in statistical significance for an important outcome (abstinence) exists among them. We feel there is a low risk of publication bias.
**Abstinence –** Cocaine/heroin [5];<br>objective	6 mo	Serious limitations	No serious limitations	No serious limitations	Serious limitations<br>CI shows both appreciable benefit and harm. One study	No serious limitations<br>Although a few number of studies located, mixed results in

Outcome	Follow-up	Risk of bias	Inconsistency	Indirectness	imprecision	Publication bias
assessment (past 30 days)					assessed outcome at 6 months.	statistical significance for an important outcome (abstinence) exists among them. We feel there is a low risk of publication bias.
**Abstinence – All Substances**<br>Cannabis [3]; any prescription drug (Sedatives/ hypnotics/opioids) [4]<br>Mix of self-report (past 30 days), and NR (past 3 months).	12 mo	Very serious limitations<br>[3] – medium risk<br>[4] - high risk	No serious limitations	No serious limitations	Serious limitations<br>Total events <300 and sample size <2000. CI for one study included both appreciable harm and benefit.	No serious limitations<br>Although a few number of studies located, mixed results in statistical significance for an important outcome (abstinence) exists among them. We feel there is a low risk of publication bias.
**High on cannabis**<br>Measured within past 30 days using Timeline Followback Calendar [3].	3 and<br>12 mo	3 mo: Serious limitations (medium)<br>12 mo: very serious limitations (high)	No serious limitations<br>(1 study only)	No serious limitations	Serious limitations<br>Total events <300 and sample size <2000. One study assessed outcome (lack of scientific replication).	No serious limitations<br>Although a few number of studies located, mixed results in statistical significance for an important outcome (abstinence) exists among them. We feel there is a low risk of publication bias.
**Reducing use >25% - Any prescription drug [4]**	3 and<br>12 mo	Very serious limitations (high)	No serious limitations<br>(1 study only)	No serious limitations	Serious limitations<br>Total events <300 and sample size <2000. One study assessed outcome (lack of scientific replication).<br>12 mo: CI includes no effect and appreciable harm.	No serious limitations<br>Although a few number of studies located, mixed results in statistical significance for an important outcome (abstinence) exists among them. We feel there is a low risk of publication bias.

Outcome	Follow-up	Risk of bias	Inconsistency	Indirectness	imprecision	Publication bias
**Frequency of use**						
**Change in cannabis consumption.** Change from baseline, measured within past 30 days using Timeline Followback Calendar [3].	3 and 12 mo	Serious limitations (medium)	No serious limitations (1 study only)	No serious limitations	Serious limitations<br>Population size <400. One study assessed outcome (lack of scientific replication).	No serious limitations<br>Although a few number of studies located, mixed results in statistical significance for an important outcome (abstinence) exists among them. We feel there is a low risk of publication bias.
**Quantity of use**						
**Reduction of defined daily dosage – Any prescription drug**<br>Patient's dose of a given prescription drug per day (in mg) divided by the product-specific WHO measure [4].	3 and 12 mo	Very serious limitations (high)	No serious limitations (1 study only)	No serious limitations	Serious limitations<br>Total population size <400. One study assessed outcome (lack of scientific replication).	No serious limitations<br>Although a few number of studies located, mixed results in statistical significance for an important outcome (abstinence) exists among them. We feel there is a low risk of publication bias.
**Change in drug level**<br>**- Cocaine**<br>**- Opioids**<br>Biochemical hair analysis [5]	6 mo	Serious limitations (medium)	No serious limitations (1 study only)	No serious limitations	Serious limitations<br>Total population size <400. One study assessed outcome (lack of scientific replication).<br>Information poorly reported – p value only provided. We do not know width of CI and therefore are uncertain how precise the data are.	No serious limitations<br>Although a few number of studies located, mixed results in statistical significance for an important outcome (abstinence) exists among them. We feel there is a low risk of publication bias.
**Use-related harms or negative consequences of use**						

Outcome	Follow-up	Risk of bias	Inconsistency	Indirectness	imprecision	Publication bias
**Carried a weapon.** Past 30 days (gun, knife, club) [3]. (330)	3 and 12 mo	Serious limitations (medium)	No serious limitations (1 study only)	No serious limitations	Serious limitations<br>Total events <300 and sample size <2000. One study assessed outcome (lack of scientific replication).<br>3 mo: CI includes no effect and appreciable benefit.<br>12 mo: CI includes no effect and both appreciable benefit and harm.	No serious limitations<br>Although a few number of studies located, mixed results in statistical significance for an important outcome (abstinence) exists among them. We feel there is a low risk of publication bias.
**Drove a car after using cannabis.** Past 30 days [3].	3 and 12 mo	Serious limitations (medium)	No serious limitations (1 study only)	No serious limitations	Very serious limitations<br>Total events <300 and sample size <2000. CI includes no effect and both appreciable benefit and harm. One study assessed outcome (lack of scientific replication).	No serious limitations<br>Although a few number of studies located, mixed results in statistical significance for an important outcome (abstinence) exists among them. We feel there is a low risk of publication bias.
**Rode in a car with a person drunk/high.** After cannabis use, past 30 days [3].	3 and 12 mo	Serious limitations (medium)	No serious limitations (1 study only)	No serious limitations	Very serious limitations<br>Total events <300 and sample size <2000. CI includes no effect and both appreciable benefit and harm. One study assessed outcome (lack of scientific replication).	No serious limitations<br>Although a few number of studies located, mixed results in statistical significance for an important outcome (abstinence) exists among them. We feel there is a low risk of publication bias.
**Physical fight.** Past 30 days [3].	3 and 12 mo	Serious limitations (medium)	No serious limitations (1 study only)	No serious limitations	Very serious limitations<br>Total events <300 and sample size <2000. One study assessed outcome (lack of scientific replication).	No serious limitations<br>Although a few number of studies located, mixed results in statistical significance for an important outcome (abstinence)

Outcome	Follow-up	Risk of bias	Inconsistency	Indirectness	imprecision	Publication bias
					3 mo: CI includes no effect and both appreciable benefit and harm.	exists among them. We feel there is a low risk of publication bias.
**Positive behaviour change**						
**Tried to cut back on cannabis use.** [3]	3 mo	Serious limitations (medium)	No serious limitations<br>(1 study only)	No serious limitations	Serious limitations<br>Total events <300 and sample size <2000. CI includes no effect and appreciable benefit.	No serious limitations<br>Although a few number of studies located, mixed results in statistical significance for an important outcome (abstinence) exists among them. We feel there is a low risk of publication bias.
	12 mo	Serious limitations (medium)	No serious limitations<br>(1 study only)	No serious limitations	Serious limitations<br>Total events <300 and sample size <2000. CI includes no effect and appreciable benefit. One study assessed outcome (lack of scientific replication).	No serious limitations<br>Although a few number of studies located, mixed results in statistical significance for an important outcome (abstinence) exists among them. We feel there is a low risk of publication bias.
**Tried to stop using cannabis.** [3]	3 and<br>12 mo	Serious limitations (medium)	No serious limitations<br>(1 study only)	No serious limitations	Serious limitations<br>Total events <300 and sample size <2000. One study assessed outcome (lack of scientific replication).<br>12 mo: CI includes no effect and appreciable benefit.	No serious limitations<br>Although a few number of studies located, mixed results in statistical significance for an important outcome (abstinence) exists among them. We feel there is a low risk of publication bias.
**"Tried to be careful about situations you got**	3 and<br>12 mo	Serious limitations (Medium)	No serious limitations<br>(1 study only)	No serious limitations	Serious limitations<br>Total events <300 and sample size <2000. One study	No serious limitations<br>Although a few number of studies located, mixed results in

Outcome	Follow-up	Risk of bias	Inconsistency	Indirectness	imprecision	Publication bias
**into when using cannabis”** [3]					assessed outcome (lack of scientific replication).<br>3 mo: CI includes no effect and appreciable benefit.	statistical significance for an important outcome (abstinence) exists among them. We feel there is a low risk of publication bias.
**Other health measures**						
**Felt unsafe in the past 30 days.** [3]	3 and<br>12 mo	Serious limitations (medium)	No serious limitations<br>(1 study only)	No serious limitations	Serious limitations<br>Total events <300 and sample size <2000. One study assessed outcome (lack of scientific replication).<br>3 mo: CI includes no effect and appreciable benefit.	No serious limitations<br>Although a few number of studies located, mixed results in statistical significance for an important outcome (abstinence) exists among them. We feel there is a low risk of publication bias.
**Change in ASI composite score from baseline – Cocaine and/or heroin**<br>Drug and medical subscales [5]	3 mo	Serious limitations (medium)	No serious limitations<br>(1 study only)	No serious limitations	Serious limitations<br>Authors only state not significant, no data are provided to calculate an effect estimate and confidence interval. Accordingly, we do not know how precise the results are.<br>One study assessed outcome (lack of scientific replication).	No serious limitations<br>Although a few number of studies located, mixed results in statistical significance for an important outcome (abstinence) exists among them. We feel there is a low risk of publication bias.
	Drug - 6 mo<br>Medical – 6 mo	Serious limitations (medium)	No serious limitations<br>(1 study only)	No serious limitations	Serious limitations<br>Information poorly reported – p value only provided. We do not know width of CI and therefore are uncertain how precise the data are.<br>One study assessed outcome	No serious limitations<br>Although a few number of studies located, mixed results in statistical significance for an important outcome (abstinence) exists among them. We feel there is a low risk of publication bias.

Outcome	Follow-up	Risk of bias	Inconsistency	Indirectness	imprecision	Publication bias
					(lack of scientific replication).	

References

1. Baer JS, Garrett SB, Beadnell B, Wells EA, Peterson PL: Brief motivational intervention with homeless adolescents: evaluating effects on substance use and service utilization. *Psychol Addict Behav* 2007, 21(4):582-586.
2. Humeniuk R, Dennington V, Ali R: *The Effectiveness of a Brief Intervention for Illicit Drugs Linked to the Alcohol, Smoking and Substance Involvement Screening Test (ASSIST) in Primary Health Care Settings: A Technical Report of Phase III Findings of the WHO ASSIST Randomized Controlled Trial.* World Health Organization; 2008.
3. Bernstein E, Edwards E, Dorfman D, Heeren T, Bliss C, Bernstein J: Screening and brief intervention to reduce marijuana use among youth and young adults in a pediatric emergency department. *Academic emergency medicine : official journal of the Society for Academic Emergency Medicine* 2009, 16(11):1174-1185.
4. Zahradnik A, Otto C, Crackau B, Lohrmann I, Bischof G, John U, Rumpf HJ: Randomized controlled trial of a brief intervention for problematic prescription drug use in non-treatment-seeking patients. *Addiction* 2009, 104(1):109-117.
5. Bernstein J, Bernstein E, Tassiopoulos K, Heeren T, Levenson S, Hingson R: Brief motivational intervention at a clinic visit reduces cocaine and heroin use. *Drug and Alcohol Dependence* 2005, 77(1):49-59.
